# RESEARCH COMMUNICATIONS OF THE 34th ECVIM‐CA CONGRESS

**DOI:** 10.1111/jvim.17231

**Published:** 2024-11-13

***5–7 September 2024***

**Cité Centre de Congrès**

**Lyon, France**

*The European College of Veterinary Internal Medicine – Companion Animals (ECVIM‐CA) Congress and the Journal of Veterinary Internal Medicine (JVIM) are not responsible for the content or dosage recommendations in the abstracts. The abstracts are not peer reviewed before publication. The opinions expressed in the abstracts are those of the author(s) and may not represent the views or position of the ECVIM‐CA. The authors are solely responsible for the content of the abstracts*.

## JVIM LIST OF ORAL RESEARCH COMMUNICATIONS

**Table:** 

ESVC – European Society of Veterinary Cardiology			
Thursday 5 September			
ESVC‐O‐1	08.15‐08.30	Gardner	Six‐lead electrocardiography in predicting subclinical hypertrophic cardiomyopathy in cats
ESVC‐O‐2	08.30‐08.45	Maffei	New electrocardiographic algorithm for the diagnosis of fast supraventricular tachycardias in dogs
ESVC‐O‐3	08.45‐09.00	Maffei	Twelve‐lead electrocardiographic differentiation of monomorphic ventricular tachycardia versus bundle branch block in dogs
ESVC‐O‐4	12.05‐12.20	Bagardi	Three step‐based electrocardiographic algorithm for the identification of accessory pathway location in dogs with ventricular pre‐excitation
ESVC‐O‐5	12.20‐12.35	Nii	Clinical utility of pharmacological defibrillation during canine mitral valve repair
ESVC‐O‐6	12.35‐12.50	Atkinson	Echocardiographic indices of reverse cardiac remodeling and outcome predictors in dogs undergoing mitral valve repair
ESVC‐O‐7	14.25‐14.40	Shinoda	Tricuspid regurgitation velocity improves after mitral valve repair in dogs but is not associated with long‐term mortality
ESVC‐O‐8	14.40‐14.55	Isayama	Trigone‐based sizing method and proposal for mitral valve annuloplasty surgery in dogs
ESVC‐O‐9	14.55‐15.10	Franchini	Three‐dimensional echocardiographic determinants of the age of onset of myxomatous mitral valve disease in Cavalier King Charles Spaniels dogs
ESVC‐O‐10	15.10‐15.25	Ferri	Assessment of Pulmonary Veins Diameter in Cavalier King Charles Spaniels with Degenerative Mitral Valve Disease
ESVC‐O‐11	15.25‐15.40	Novo Matos	Comparison of left ventricular wall measurements obtained from different echocardiographic views: a study in 408 cats
ESVC‐O‐12	15.40‐15.55	Oricco	Mitral to aortic velocity‐time integral ratio to estimate the regurgitant fraction in dogs with myxomatous mitral valve disease
ESVC‐O‐13	15.55‐16.10	Korzybska	Use of the Subcostal View for Obtaining Aortic Spectral Doppler Flow Velocities in Cats
ESVC‐O‐14	16.10‐16.25	Showers	Comparison of cardiac output measurements utilizing three‐dimensional transesophageal echocardiography and two‐dimensional transesophageal cardiography
ESVC‐O‐15	16.25‐16.40	Kennedy	Comparing pulmonic and aortic velocity‐time integral‐derived cardiac output parameters in dogs receiving intravenous fluid challenges: a preliminary study
Friday 6 September 2024			
ESVC‐O‐16	14.25‐14.40	Mazzarella	Red blood cell glycocalyx damage in canine myxomatous mitral valve disease
ESVC‐O‐17	14.40‐14.55	Patata	Echocardiographic left ventricular wall thickness in cats: the role of the breed and the body weight
ESVC‐O‐18	14.55‐15.10	Papa	Congenital cardiopathies in cats: a retrospective analysis in a single referral centre
ESVC‐O‐19	15.10‐15.25	Pierce	The BEAMS Study—Baseline Evaluation And Management of Scatter radiation using RADPAD radiation absorbing drapes during veterinary interventional cardiac procedures
ESVC‐O‐20	15.25‐15.40	Galanakis	Veterinary cardiologists’ preferences regarding interventional cardiac procedures
ESVC‐O‐21	15.40‐15.55	Dickson	Validation of a physical examination algorithm in dyspnoeic cats presenting to general practice: the RAPID CAT 2 study
ESVC‐O‐22	16.30‐16.45	Petchdee	Rivaroxaban versus enoxaparin plus clopidogrel therapy in cats with thromboembolic disease
ESVC‐O‐23	16.45‐17.00	Merveille	Observational longitudinal study of pulmonary hypertension in West Highland white terriers affected with canine idiopathic pulmonary fibrosis
ESVC‐O‐24	17.00‐17.15	Merveille	Acute and chronic pulmonary thromboembolism in dogs: a prospective clinical study
ESVC‐O‐25	17.15‐17.30	Goya	Effects of adjunct ambrisentan therapy on clinical signs, oxygenation capacity, and echocardiographic variables in dogs with pulmonary hypertension resistant to sildenafil therapy
ESVC‐O‐26	17.30‐17.45	Lombardo	Subcutaneous furosemide therapy for chronic management of refractory congestive heart failure in dogs and cats
ESVC‐O‐27	17.45‐18.00	Murphy	Management and short‐term outcome of 74 cases of acute congestive heart failure in cats (2017‐2022)
SCH – Society of Comparative Hepatology			
Thursday 5 September			
SCH‐O‐1	08.00‐08.15	Phillips	Altered serum amino acids profiles in dogs with liver disease
SCH‐O‐2	08.15‐08.30	Lopez‐gomez	Effectiveness and associated adverse effects of telmisartan in the treatment of portal hypertension secondary to liver disease in dogs
SCH‐O‐3	08.30‐08.45	Dröes	Intact thrombin generation potential in dogs with different liver diseases
SCH‐O‐4	08.45‐09.00	Habermaass	Fecal bile acids in canine chronic liver disease: results in 46 dogs
SCH‐O‐5	09.00‐09.15	Johnston	Does canine schistosomiasis promote hepatocellular carcinogenesis?
SCH‐O‐6	09.15‐09.30	Lubbers	Retrospective analysis of the incidence and clinicopathological findings associated with ammonium urate urolithiasis in dogs with congenital hepatic vascular anomalies; 378 cases (2010‐2023)
SCH‐O‐7	09.30‐09.45	Petibon	Risk factors for short‐term mortality of hepatic lipidosis: a multicentre retrospective study of 162 cats
SCH‐O‐8	09.45‐10.00	Fong	Complications associated with hepatic fine needle aspiration in cats; a retrospective study
SCH‐O‐9	10.00‐10.15	Mendoza	The effect of transdermal mirtazapine on the appetite recovery time in hospitalized cats with hepatobiliary disease: a prospective study
SCH‐O‐10	10.15‐10.30	Hernandez Perello	Feline emphysematous cholecystitis, a descriptive study
ESVNU – European Society of Veterinary Nephrology and Urology			
Thursday 5 September			
ESVNU‐O‐1	09.00‐09.15	Rossi	Exploring the impact of kidney biopsy on survival in dogs with glomerular disease: insights from the european veterinary renal pathology service (evrps)
ESVNU‐O‐2	09.15‐09.30	Man	Expression of β thymosin peptides in cat kidneys and changes associated with chronic kidney disease
ESVNU‐O‐3	09.30‐09.45	Remmel	Usefulness of parathormone, calcitriol and parathyroid glands ultrasound in differentiating acute kidney injury and acute on chronic kidney disease in dogs
ESVNU‐O‐4	09.45‐10.00	Corsaletti	Comparison of long‐term outcomes after SUB implantation in cats with and without systematic flush: a retrospective study of 128 cases (2013‐2024)
ESVNU‐O‐5	10.00‐10.15	Gravgaard	Increased Serum Amyloid A in cats with pyelonephritis
ESVNU‐O‐6	10.15‐10.30	Erika Leth Vasby	Urinary levels of interleukin‐8 in dogs with bacteriuria
ESVNU‐O‐7	11.20‐11.35	Lopez	Evaluation of probiotic supplementation in dogs with chronic kidney disease
ESVNU‐O‐8	11.35‐11.50	Sugar	Clinical Findings, Prognostic Factors and Outcomes of Feline Protein Losing Nephropathy secondary to glomerular disease: A Retrospective Study
ESVNU‐O‐9	11.50‐12.05	Bernard	Effects and remanence of short‐course of telmisartan or benazepril on renal function of healthy dogs: a prospective, cross‐over, double‐masked, placebo‐controlled study
Friday 6 September 2024			
ESVNU‐O‐10	09.00‐09.15	Gordin	Urinary Clusterin and Cystatin B in Dogs with Spontaneous Acute Kidney Injury
ESVNU‐O‐11	09.15‐09.30	Jaturunratsamee	Innovation of polycystic kidney disease test kit by loop‐mediated isothermal amplification
ESVNU‐O‐12	09.30‐09.45	Fidanzio	Evaluation of serum C‐reactive protein in dogs with urinary tract infections
ESVNU‐O‐13	09.45‐10.00	Lunet Marques	New insights into the role of adrenal function in chronic kidney disease
ESVNU‐O‐14	10.00‐10.15	Pichard	Mycoplasma‐associated urethritis in dogs: A case series
ESVNU‐O‐15	10.15‐10.30	Marynissen	Renal health in aging apparently healthy dogs: panel of serum and urinary biomarkers
ESCG – European Society of Comparative Gastroenterology			
Friday 6 September 2024			
ESCG‐O‐1	08.15‐08.30	Winston	Ursodiol Administration Modulates the Intestinal Ecosystem of Dogs and Cats
ESCG‐O‐2	08.30‐08.45	Suchodolski	Effect of metronidazole on fecal microbiome, metabolome, and serum uremic toxins in healthy adult cats
ESCG‐O‐3	08.45‐09.00	Simpson	Limited ingredient diets remodel the enteric microbiome and serum metabolome of dogs with food responsive enteropathy
ESCG‐O‐4	09.00‐09.15	Rowe	Fecal Bile Acid Dysmetabolism is Correlated with Relative Abundance of Peptacetobacter hiranonis and Oscillospirales in Cats with Chronic Kidney Disease
ESCG‐O‐5	09.15‐09.30	Winston	Multi‐omic Approach Identifies Expanded Bile Acid Pools, New Microbially Conjugated Bile Acids, and Bile Acid Biotransformation Genes in the Canine and Feline Gut Microbiota
ESCG‐O‐6	09.30‐09.45	Busch	Recovery of fecal fatty acid and sterol profiles in dogs with acute hemorrhagic diarrhea syndrome
ESCG‐O‐7	09.45‐10.00	Rowe	Metagenomic Sequencing Identifies Specific Gut Microbes Associated with Resolution of Canine Acute Noninfectious Colitis
ESCG‐O‐8	10.00‐10.15	Chen	Functional gene profile and targeted metabolome in dogs with chronic enteropathy
ESCG‐O‐9	10.15‐10.30	Barko	Disruptions in microbial tryptophan catabolism are associated with fecal abundance of Bifidobacterium and disease activity indices in cats with chronic enteropathies
ESCG‐O‐10	11.20‐11.35	Benoit	Prediction of mortality and intensive care unit length of stay in dogs with acute pancreatitis using machine learning algorithms: a pilot study on 215 dogs
ESCG‐O‐11	11.35‐11.50	Wu	Re‐evaluation of the Reference Interval and Cut‐off Value for Canine Trypsin‐like Immunoreactivity Assay
ESCG‐O‐12	11.50‐12.05	Sidler	Comparison of clinical, laboratory, and ultrasonographic findings in dogs with acute pancreatitis and acute idiopathic gastrointestinal disease
ESCG‐O‐13	12.05‐12.20	Bigay	Use of budesonide in feline chronic enteropathies: a retrospective study of 102 cases
ESCG‐O‐14	12.20‐12.35	Kaufmann	Functional dyspepsia in dogs and humans: a comparative approach and a retrospective study of 29 canine cases
ESCG‐O‐15	12.35‐12.50	Freisleben	Occurrence of clinical signs in young dogs with and without Giardia duodenalis infection
ESVIM – European Society of Veterinary Internal Medicine			
Saturday 7 September			
ESVIM‐O‐1	08.00‐08.15	Henry	Canine chronic idiopathic rhinitis, management and outcome: a single centre retrospective observational study
ESVIM‐O‐2	08.15‐08.30	Vilcot	Association between signalment, clinical signs and nasal diseases type and location in dogs and cats
ESVIM‐O‐3	08.30‐08.45	Neittaanmäki	Histopathological findings in the nasal biopsies of dogs without nasal signs compared to dogs with chronic inflammatory rhinitis
ESVIM‐O‐4	08.45‐09.00	Grobman	Evaluation of Silent Aspiration in Dogs using Videofluoroscopic Swallow Studies
ESVIM‐O‐5	14.25‐14.40	Buton	Description and comparison of efficacy and safety of blind bronchoalveolar lavage using a sterile endotracheal tube or direct laryngo‐tracheal catheterization in 51 cats
ESVIM‐O‐6	14.40‐14.55	Chang	Presence of fungi from bronchoalveolar lavage specimens in clinical cats and dogs – a retrospective evaluation over a ten‐year period
ESVIM‐O‐7	14.55‐15.10	Parreño	Comparison of cytological findings after obtaining two consecutive samples of bronchoalveolar lavages in dogs and cats: a prospective study of 21 cases
ESVIM‐O‐8	15.10‐15.25	Robin	Clinical and prognostic relevance of Mycoplasma felis PCR detection in feline lower respiratory tract diseases
ESVIM‐O‐9	15.25‐15.40	Johnston	Comparison of C‐reactive protein with other markers of inflammation in dogs with meningoencephalitis of unknown origin and steroid responsive meningitis‐arteritis
ESVIM‐O‐10	15.40‐15.55	Petitpre	Evaluation of C‐reactive protein assay in canine bronchoalveolar lavage fluid as a biomarker of bacterial bronchopneumopathy in dogs: A multicenter prospective study
ESVIM‐O‐11	16.30‐16.45	Reguera Burgos	Donor and unit‐specific factors influencing hemolysis in stored canine and feline packed red blood cells
ESVIM‐O‐12	16.45‐17.00	Broughton	Anaemic retinopathy in a referral population of anaemic cats
ESVIM‐O‐13	17.00‐17.15	Beriot	General health quality‐of‐life questionnaires reveal subtle behavioural changes in undiagnosed hypertensive older cats
ESVIM‐O‐14	17.15‐17.30	Meler	Drivers and challenges of modern clinical education in veterinary teaching hospitals: Using causal loop modelling to understand better the complexity of interactions at play within academic hospitals and within the broader University system
ESVIM‐O‐15	17.30‐17.45	Knopf	Causes and outcome of systemic inflammatory response syndrome (SIRS) in dogs – analysis of 172 cases
ESVIM‐O‐16	17.45‐18.00	Bouzouraa	Long‐term follow‐up of dogs treated with a chlorambucil‐prednisolone combination for Steroid‐Responsive Meningitis‐Arteritis: a Prospective study of 12 cases
ISCAID – International Society for Companion Animal Infectious Diseases			
Friday 6 September			
ISCAID‐O‐1	14.25‐14.40	Zwicklbauer	Characterization of the lymphocyte response in cats with feline infectious peritonitis during antiviral treatment using the SMART Tube technology for flow cytometry in feline full blood samples
ISCAID‐O‐2	14.40‐14.55	Meli	Alpha‐1‐acid glycoprotein quantification via spatial proximity analyte reagent capture luminescence assay – validation and application as diagnostic and prognostic marker in serum and effusions of cats with feline infectious peritonitis during GS‐441524 treatment
ISCAID‐O‐3	14.55‐15.10	Wenk	Feline coronavirus loads and presence of co‐infections in cats diagnosed with feline infectious peritonitis
ISCAID‐O‐4	15.10‐15.25	Cerna	Assessment of cardiac changes in cats with feline infectious peritonitis
ISCAID‐O‐5	15.25‐15.40	Schlachet	Unlicensed antiviral treatment with GS‐441524: How are clinicians approaching feline infectious peritonitis in primary care practices ?
ISCAID‐O‐6	15.40‐15.55	Buchta	Shorter treatment duration of 42 days with oral GS‐441524 also leads to complete remission in cats with feline infectious peritonitis
ISCAID‐O‐7	15.55‐16.10	Bergamo	Isolation of canine Leptospira Australis strains from the field in view of vaccine development
ISCAID‐O‐8	16.30‐16.45	Schmitt	Comparison of 5 different Leptospira spp. antibody point‐of‐care test results in healthy dogs after vaccination against leptospirosis
ISCAID‐O‐9	16.45‐17.00	Griebsch	Serovar Australis replaces serovar Copenhageni as the most common serovar causing Canine Leptospirosis in New South Wales, Australia—a case series (2021‐2023)
ISCAID‐O‐10	17.00‐17.15	Lecot	Potential leptospiral shedding in vaccinated and asymptomatic pack hounds: a prospective study in 56 dogs
ISCAID‐O‐11	17.15‐17.30	Evason	Giardia assemblages with zoonotic potential are more commonly found in cats than in dogs in fecal molecular diagnostic test samples collected from the US, Canada, Asia, and Brazil
ISCAID‐O‐12	17.30‐17.45	Sørensen	Evaluation of attitudes and behaviours related to antimicrobial prescribing in canine acute diarrhoea using discrete choice experiments
ISCAID‐O‐13	17.45‐18.00	Belshaw	Poor awareness amongst residents in the United Kingdom (UK) of the canine and human health risks associated with imported puppies carrying non‐endemic diseases
ISCAID‐O‐14	18.00‐18.15	Mauron	Effectiveness of the implementation of a revised hygiene concept on hand hygiene compliance and colonisation of hospitalized cats and dogs with multidrug‐resistant organisms
ESVONC – European Society of Veterinary Oncology			
Friday 6 September 2024			
ESVONC‐O‐1	15.10‐15.25	Faroni	A phase 2, single‐arm, open‐label clinical trial on adjuvant peptide‐based vaccination in dogs with biologically aggressive melanoma undergoing tumor excision and lymphadenectomy
ESVONC‐O‐2	15.25‐15.40	Ubiali	CD47 in canine round‐cell tumors: an explorative flow cytometric assessment
ESVONC‐O‐3	15.40‐15.55	Maga	Evaluation of risk factors for the development of canine diffuse large B‐cell lymphoma in northern Italy
ESVONC‐O‐4	16.30‐16.45	Mourou	Clinico‐hematological profile and outcomes of acute leukemias/myelodysplastic syndromes in cats: a retrospective study of 24 cases
ESVONC‐O‐5	16.45‐17.00	Vigouroux	Tolerability and short term efficacy of intra‐arterial vinblastine for the treatment of naturally occurring lower urinary tract carcinomas in dogs
ESVONC‐O‐6	17.00‐17.15	Lane	Bilateral anal sacculectomy in the management of apocrine gland anal sac adenocarcinoma: a retrospective assessment
ESVONC‐O‐7	17.15‐17.30	Coppola	Presentation and treatment of feline HSA in the UK 2009‐2024: a retrospective study of 74 cases
ESVONC‐O‐8	17.30‐17.45	Kapturska	New hope in the treatment of canine hemangiosarcoma – potentiation of doxorubicin with Poly (ADP‐ribose) polymerase inhibitors and repurposed propranolol
ESVONC‐O‐9	17.45‐18.00	Rolf	Histopathological growth pattern may predict survival in dogs with splenic haemangiosarcoma post surgery; a case‐control pilot study
ESVE – European Society of Veterinary Endocrinology			
Saturday 7 September			
ESVE‐O‐1	08.15‐08.30	Didier	Experiences with flash glucose monitoring system in dogs and cats: a global survey of veterinary practitioners
ESVE‐O‐2	08.30‐08.45	Del Baldo	Assessment of serum fructosamine and glycated hemoglobin as indicators of glycemic control in diabetic dogs: a comparative study using FreeStyle Libre‐derived metrics
ESVE‐O‐3	08.45‐09.00	Miceli	Cabergoline for the treatment of feline hypersomatotropism without concurrent diabetes mellitus
ESVE‐O‐4	09.00‐09.15	Kaemper	Does hypophysectomy cause oxytocin deficiency in cats?
ESVE‐O‐5	09.15‐09.30	Stammeleer	Is follow‐up thyroid scintigraphy useful in hyperthyroid cats treated with radioiodine?
ESVE‐O‐6	09.30‐09.45	Lin	Large‐scale screening for cases of suspected spontaneous hypothyroidism in adult cats and description of clinical, laboratory, scintigraphic features and treatment in four affected cats
ESVE‐O‐7	09.45‐10.00	Hulsebosch	Clinical and laboratory characterization of congenital hypothyroidism in 23 kittens
ESVE‐O‐8	10.00‐10.15	Schwens	Measurement of reverse triiodothyronine concentrations in healthy dogs, dogs with primary hypothyroidism and nonthyroidal illness syndrome
ESVE‐O‐9	10.15‐10.30	Miceli	Quantitative thyroid scintigraphy for the diagnosis of subclinical hypothyroidism in dogs
ESVE‐O‐10	11.20‐11.35	Rossi	Re‐evaluating diagnostic cut‐off values: impact of Immulite‐2000‐antibody change on canine hypercortisolism diagnosis using low‐dose dexamethasone suppression test
ESVE‐O‐11	11.35‐11.50	Meunier	Implementing salivary cortisol testing in a clinical setting: feasibility and usefulness of at‐home salivary sampling to monitor trilostane treatment in dogs with Cushing's syndrome
ESVE‐O‐12	11.50‐12.05	Torrano Guillamón	Performance of an in‐clinic point‐of‐care cortisol immunoassay for dogs with hypoadrenocorticism
ESVE‐O‐13	12.05‐12.20	Da Silva	Potential factors that influence adrenocorticotropic hormone stimulation test results in suspect addisonian dogs
ESVE‐O‐14	12.20‐12.35	van den Berg	Canine pheochromocytoma organoids: an in vitro model to explore new treatment options
ESVE‐O‐15	12.35‐12.50	van Bokhorst	Steroid profiling in canine adrenocortical incidentalomas
ESVCN – European Society of Veterinary Comparative Nutrition			
Saturday 7 September			
ESVCN‐O‐1	08.00‐08.15	Carciofi	Amino acid supplementation preserves protein turnover rate in obese dogs under weight loss
ESVCN‐O‐2	08.15‐08.30	Carciofi	Protein turnover of overweight and non‐overweight cats fed high starch or high protein kibble diets
ESVCN‐O‐3	08.30‐08.45	Henríquez	Environmental impacts of extruded or homemade dog foods determined by Life Cycle Assessment (LCA)
ESVCN‐O‐4	08.45‐09.00	Vecchiato	Hermetia illucens diet improved gut microbial metabolic activity in healthy dogs
ESVCN‐O‐5	09.00‐09.15	de Souza Theodoro	Urine as alternative fluid for the Doubly Labelled Water method use in dogs with Chronic Kidney Disease (preliminary evaluation)
ESVCN‐O‐6	09.15‐09.30	De Souza Theodoro	Hydrolyzed poultry byproduct meal effects on inflammatory and lipid parameters of dogs
ESVCN‐O‐7	09.30‐09.45	Kavanagh	Ultra‐low fat home cooked diets improve clinical scores and albumin in dogs with protein losing enteropathy refractory to standard management with low fat diets and immunomodulation
ESVCN‐O‐8	09.45‐10.00	Mullis	High calorie, nutrient dense diet supports body weight in dogs with malignant cancer
ESVCN‐O‐9	10.00‐10.15	Paßlack	Impact of the dietary supplementation of sunflower oil, fish oil and lard on the urine composition and selected blood values of healthy adult cats
ESVCN‐O‐10	10.15‐10.30	Pignataro	Analysis of the impact of nutritional supplementation on canine gut microbiota through an in vitro colonic fermentation model

## JVIM LIST OF POSTER RESEARCH COMMUNICATIONS

**Table:** 

ESVC – European Society of Veterinary Cardiology		
ESVC‐P‐1	Cimerman	Immune dysregulation in dogs with myxomatous mitral valve disease: insights into T lymphocyte and monocyte subtypes and natural killer cells
ESVC‐P‐2	Vezzosi	An outcome‐based severity classification of left heart enlargement in dogs with myxomatous mitral valve disease
ESVC‐P‐3	Spalla	Retrospective review of the use of hydrochlorothiazide in dogs with myxomatous mitral valve disease
ESVC‐P‐4	Mangklabruks	Blood Transcriptome profiles in healthy cats Compared to Cats with Asymptomatic and Symptomatic Hypertrophic Cardiomyopathy
ESVC‐P‐5	Dhunputh	Phonocardiographic features in cats with obstructive hypertrophic cardiomyopathy
ESVC‐P‐6	Poissonnier	Transient restrictive cardiomyopathy phenotype in cats: first reported cases (*n* = 7)
ESVC‐P‐7	Oh	Neutrophil‐to‐lymphocyte ratio and platelet‐to‐lymphocyte ratio in cats with arterial thromboembolism
ESVC‐P‐8	Mampaey	Ionized hypocalcemia as a prognostic indicator in cats with arterial thromboembolism
ESVC‐P‐9	Bichelberger	A case series of 5 cats with bacterial pericarditis with purulent pericardial effusion
ESVC‐P‐10	Feo Bernabe	Usefulness of tomographic measurements of vertebral heart volume ratio and vertebral heart scale in canine heartworm disease
ESVC‐P‐11	Feo Bernabe	Evaluation of the ratio between the thickness of the bronchial wall and the pulmonary artery using Computed Tomography in cats seropositive to Dirofilaria immitis
ESVC‐P‐12	Valente	Determination of plasmatic dimethylarginines in dogs with pulmonary hypertension
ESVC‐P‐14	Oliveira	Circadian variation in ventricular rate in dogs with atrial fibrillation
ESVC‐P‐15	Glaewketgarn	Assessment of longitudinal systolic function by using tissue motion annular displacement in hypertrophic cardiomyopathy cats
ESVC‐P‐17	Della Pina	Echocardiographic prediction intervals of the aortic and pulmonary valve annulus diameter in 1429 dogs: population‐wide and selected breed‐specific values
ESVC‐P‐18	Gheeraert	Mid‐systolic depolarization time determined by vectorcardiography as a potential new pre‐screening tool for preclinical dilated cardiomyopathy in Doberman Pinschers
ESVC‐P‐19	Grosso	Echocardiographic reference intervals of the two‐ and three‐dimensional size of the right atrium: a prospective study in 195 healthy dogs
ESVC‐P‐20	Grosso	Effect of echocardiographic imaging view and imaging mode on variability of left ventricular wall thickness measurements in normal cats and cats with hypertrophic cardiomyopathy
ESVC‐P‐21	Álamo Fernández	Validation of a four‐chamber oblique right parasternal view in the echocardiographic evaluation of transmitral flow and mitral regurgitation in dogs with cardiac pathology
ESVC‐P‐22	Dossi	Evaluation of plasma N‐terminal pro‐B‐type natriuretic peptide and urinary aldosterone‐to‐creatinine ratio in healthy Chihuahuas
ESVC‐P‐23	Manson	Dose‐exposure‐response of CARDALIS® (benazepril/spironolactone) on biomarkers of the classical and alternative arms of the renin‐angiotensin‐aldosterone system in healthy dogs
ESVC‐P‐24	Alibrandi	Content of circulating extracellular vesicles as biomarker of oxidative stress in dogs with preclinical myxomatous mitral valve disease
ESVC‐P‐25	Hardy	Left heart dimensions and cardiac troponin I concentrations in dogs presenting with diarrhoea
SCH – Society of Comparative Hepatology		
SCH‐P‐1	Di Filippo	Percutaneous endovascular embolization for treatment of single extrahepatic portosystemic shunt in four dogs
SCH‐P‐2	Mateu	Retrospective study of 15 cats with hepatic lipidosis: clinicopathological findings
SCH‐P‐3	Espenica	A retrospective, multicentre study investigating the use of C‐reactive protein in the diagnosis and monitoring of canine bacterial cholecystitis
SCH‐P‐4	Corominas	Retrospective study of 80 dogs with congenital portosystemic shunts
SCH‐P‐5	Busato	Differences in signalment and biochemical markers of hepatic function between dogs with intrahepatic and extrahepatic congenital portosystemic shunts and investigation of post‐attenuation seizures development
SCH‐P‐6	Fuentes Rodríguez	Histopathological frequency of feline hepatobiliary diseases in Ireland (2013‐2023)
ESVNU – European Society of Veterinary Nephrology and Urology		
ESVNU‐P‐1	Jessen	Increased concentrations of Kidney Injury Marker‐ 1 in urine of cats with pyelonephritis and cats with ureteral obstructions
ESVNU‐P‐2	Ruane	Aetiology of proteinuria in dogs without known pre‐renal, renal or post‐renal causes
ESVNU‐P‐3	Dorsch	Evaluation of the effect of renaltec in cats with chronic kidney disease IRIS Stage 2 and 3
ESVNU‐P‐4	Bernard	Clinical value of monitoring the urinary amino acid excretional fraction in the diagnosis and management of dogs with androgen‐dependent cystinuria
ESVNU‐P‐5	Bujok	Results of renal replacement therapy for acute kidney injury caused by Babesia canis in dogs
ESVNU‐P‐6	Rafalska	Comparison of the usefulness of cystatin C, creatinine, SDMA assays in the assessment of renal function in small and miniature dog breeds
ESVNU‐P‐7	Jousserand	Routine urinalysis predicts the results of urinary bacteriological culture in dogs and cats
ESCG – European Society of Comparative Gastroenterology		
ESCG‐P‐1	Gil	Prospective randomized controlled clinical trial of the long‐term effects of omeprazole in healthy dogs
ESCG‐P‐2	Higueras	Haematological parameters and blood cell ratios highly correlate with faecal characteristics in dogs with Food‐responsive and Immunosuppressant‐responsive enteropathies (FRE and IRE)
ESCG‐P‐3	Yu	Evaluation of three serum proteomic biomarkers in chronic enteropathy in dogs
ESCG‐P‐4	Borgonovi	Prevalence of hypocobalaminemia and hypercobalaminemia in a referral population of cats in the United Kingdom and their disease associations
ESCG‐P‐5	Maufras	Influence of breed on the composition of the fecal microbiota in healthy dogs
ESCG‐P‐6	Stringfellow	Value of routine faecal analysis and antibiotic usage in dogs with diarrhoea in general practice in the Southwest of England – a retrospective study
ESCG‐P‐7	Suchodolski	Assessing the reproducibility of shotgun metagenomic sequencing and quantitative PCR assays for evaluating the fecal microbiota in dogs
ESCG‐P‐8	Codea	Associations between ultrasonographic imaging of the gallbladder and pathogenic bacteria isolation in twenty‐two dogs with extrahepatic biliary disease
ESCG‐P‐9	Kunath	Serum transcobalamin II concentrations are decreased in cats with chronic enteropathy
ESCG‐P‐10	Fauquet	Effects of short‐term immunosuppressive corticosteroid therapy on 1,2‐o‐dilauryl‐rac‐glycero‐glutaric acid (6'‐methylresorufin)‐ester lipase activity and canine pancreatic‐specific lipase concentration in healthy dogs
ESCG‐P‐11	Berro	Comparison of clinical and paramedical abnormalities between dogs with food‐responsive enteropathy and dogs with immunosuppressant‐responsive enteropathy: A retrospective study
ESCG‐P‐12	Hernandez	Diet modulates gut microbiota composition and derived bio‐products in healthy dogs
ESCG‐P‐13	Dainese	Predisposition of Maine Coons to intestinal intussusception and clinical pathological features of these cats
ESCG‐P‐15	Del Baldo	Insect‐based (Hermetia Illucens) Diet as a Novel Protein Source for the Management of Food‐Responsive Chronic Enteropathy in Dogs
ESCG‐P‐16	Hernández	Use of a novel low–fat hydrolysed diet in dogs with chronic enteropathy
ESCG‐P‐17	Sidler	Evaluation of serum pancreatitis‐associated protein in dogs hospitalized for acute pancreatitis.
ESCG‐P‐18	Pichard	Micro‐ulcerative gastropathy in cats: A case series
ESCG‐P‐19	Benvenuti	Chronic inflammatory enteropathies in Miniature Poodle: retrospective studies in 131 dogs
ESCG‐P‐20	Hernando Montalbán	Relationship between folate and cobalamin serum concentrations and small intestinal ultrasonographic abnormalities in dogs with gastrointestinal signs
ESCG‐P‐21	Rösch	Fecal calprotectin concentrations during administration of oral cyclosporine A in healthy cats
ISCAID – International Society for Companion Animal Infectious Diseases		
ISCAID‐P‐1	Pavlin	Investigation of possible recombination between feline corona virus and SARS‐CoV‐2 in cats with feline infectious peritonitis
ISCAID‐P‐2	Monroig Carbonell	Haematological Ratios in Dogs with Leishmaniosis: Implications for Disease Classification and Survival
ISCAID‐P‐3	Monroig Carbonell	Comparison of haematological ratios (neutrophil‐to‐lymphocyte ratio, platelet‐to‐lymphocyte ratio, monocyte‐to‐lymphocyte ratio, and systemic immunity‐inflammation index) between healthy dogs and those with leishmaniosis
ISCAID‐P‐4	Loubser	Comparison of neutrophil myeloperoxidase index in dogs with canine parvoviral enteritis to a healthy control population
ISCAID‐P‐5	Dominguez	Acute kidney injury as the main cause of hospitalization and death in dogs with leishmaniosis.
ISCAID‐P‐6	Dominguez	Exploring the Impact of Initial Treatment on Clinical Outcomes in Canine Leishmaniosis: A Retrospective Analysis over 20 years (2000‐2020)
ISCAID‐P‐7	Cloete	Prevalence of co‐infections with Ehrlichia spp. or Theileria spp. in dogs naturally infected with babesiosis in the Eastern Cape province, South Africa
ISCAID‐P‐8	Bouvet	A new licensed quadrivalent antileptospiral canine vaccine prevents mortality, clinical signs, infection, bacterial excretion, renal carriage and renal lesions caused by Leptospira Australis experimental challenge
ISCAID‐P‐9	Dupuis	Molecular survey of tick‐borne pathogens in dogs and their ticks in France
ISCAID‐P‐10	Geks	Detection of S gene mutation (M 1058L) and feline coronavirus ribonucleic acid in the aqueous humour of three living cats with uveitis and three negative controls
ISCAID‐P‐11	Acke	Protothecosis as an emerging cause of colitis and multisystemic disease of dogs in temperate Europe: five cases
ISCAID‐P‐12	Nielsen	Are inflammatory biomarkers associated with bleeding diathesis in dogs with Angiostrongylus vasorum?
ISCAID‐P‐13	Sandbrink	Retrospective evaluation of positive blood cultures from dogs (2014–2022)
ISCAID‐P‐14	Sørensen	Evaluation of attitudes and behaviours related to antimicrobial prescribing in canine urinary tract infections using discrete choice experiments
ISCAID‐P‐15	Petini	The role of bone marrow parasitic load in canine leishmaniasis: correlation/agreement with clinical status, leishvet staging system, acute phase protein and hematologic alterations at the time of diagnosis
ISCAID‐P‐16	Meunier	Short‐term outcome of clinical and laboratory variables in cats with feline infectious peritonitis receiving oral treatment with GS‐441524, with and without additional corticosteroids
ISCAID‐P‐17	Ljungquist	How low can you go? Antibiotic use in Swedish dogs with gastroenteritis
ISCAID‐P‐18	Ng	Understanding the rationale for metronidazole use in dogs and cats
ISCAID‐P‐19	Prior	Encouraging safe antibiotic disposal: a One Health initiative
ISCAID‐P‐20	Schlachet	Antimicrobial susceptibility patterns in hepatobiliary bacterial infections in dogs and cats: a retrospective study about 343 isolates from 2010 to 2019
ESVIM – European Society of Veterinary Internal Medicine		
ESVIM‐P‐1	Chang	Serum total IgE levels in clinical feline patients with asthma, chronic bronchitis, and mixed‐type inflammatory lower airway disease
ESVIM‐P‐2	Viitanen	Bronchoalveolar lavage fluid hyaluronan in dogs with bronchial diseases
ESVIM‐P‐3	David	Silicone stent placement for laryngeal paralysis and other laryngeal diseases: a retrospective study of 13 cases.
ESVIM‐P‐4	Lee‐Fowler	Investigating the presence of gastroesophageal reflux disease in dogs with chronic rhinitis
ESVIM‐P‐5	Vlassenbroek	A retrospective study on the effect of packed red blood cell transfusion requirements on long term prognosis in dogs with non‐associative immune‐mediated hemolytic anemia
ESVIM‐P‐6	Vlassenbroek	What is the diagnostic yield and clinical impact of diagnostic imaging in dogs diagnosed with immune‐mediated hemolytic anemia in Belgium: A retrospective study
ESVIM‐P‐7	Pimenta da Silva	Prevalance of transmissible feline blood pathogens in a blood donor population tested on every donation
ESVIM‐P‐8	Miglio	lipidomic profiles of canine leukoreducted and non‐leukoreducted packed red blood units during storage
ESVIM‐P‐9	Menard	Systemic atherosclerosis in eurasier with presumed primary hypercholesterolemia
ESVIM‐P‐10	Gabarda	Restrospective study of 65 dogs with polyarthritis in a Mediterranean area
ESVONC – European Society of Veterinary Oncology		
ESVONC‐P‐1	Rizzoli	Unveiling the tumour microenvironment in canine pulmonary adenocarcinoma through single‐cell RNA sequencing
ESVONC‐P‐2	Brøgger	Investigating the national cause of death in dogs from 2013 to 2023: An oncological perspective
ESVONC‐P‐3	Dini	Serum amino acid profile in canine oncologic disease
ESVONC‐P‐4	Fejős	Tolerability and outcome of a higher fractionated dose of cyclophosphamide in the 19‐week cyclophosphamide, doxorubicin, vincristine, and prednisone based protocol in dogs with high‐grade multicentric B‐cell lymphoma
ESVE – European Society of Veterinary Endocrinology		
ESVE‐P‐1	Nilsen	Increased level of circulating trimethylamine N‐oxide (TMAO) in dogs with naturally occurring diabetes mellitus: a pilot study
ESVE‐P‐2	Daminet	Urinary active transforming growth factor beta 1 in cats with hyperthyroidism before and after radioiodine treatment
ESVE‐P‐3	Fowkes	An investigation into venous blood electrolytes, blood gas and markers of perfusion following transsphenoidal hypophysectomy to treat hypersomatotropism in cats
ESVE‐P‐4	Domínguez Madsen	Patterns of the low dose dexamethasone suppression test in dogs with pheochromocytoma
ESVE‐P‐6	Le Boedec	Performance of the Addison Detect Tool to screen for canine hypoadrenocorticism: external validation
ESVE‐P‐7	Lunet Marques	Characterization of hyaluronic acid concentrations in dogs with naturally‐occurring hypercortisolism and possible implications
ESVE‐P‐8	Petroff	Iodine is not increased in sera from dogs with autoimmune thyroiditis or hypothyroidism
ESVE‐P‐9	Miceli	Comparison of insulin requirements and fructosamine concentrations between diabetic dogs with naturally occurring hypercortisolism and hypothyroidism
ESVE‐P‐10	Shelton	Owner points of view and perceived quality of life of their diabetic cats following hypophysectomy
ESVE‐P‐11	Leuthard	Naturally occurring hypothyroidism in adult cats: A prospective multi‐center study in Central Europe
ESVE‐P‐12	Muthmann	Influence of the owner's personality and other owner‐, cat‐ and treatment‐related factors on the perception of the quality of life for cats with hyperthyroidism
ESVE‐P‐13	Fourie‐Viljoen	Cortisol and thyroxine changes in snake envenomation as a model of acute canine critical illness
ESVE‐P‐14	Petroff	Demographics of 4,620 cats with presumed hypersomatotropism in the USA and Canada
ESVE‐P‐15	Waite	Presenting signs, diagnostic testing and clinical management in 375 diabetic cats under UK primary veterinary care
ESVE‐P‐16	Emming	New Practical Insights in the Management of Hypoadrenocorticism in Dogs
ESVE‐P‐17	Eiermann	Hypoadrenocorticism in dogs presenting to a tertiary veterinary clinic in Switzerland: Overall prevalence, presenting complaints, laboratory changes and outcome
ESVE‐P‐18	Jaresova	Survey about management of diabetes mellitus in dogs and cats
ESVE‐P‐19	Mochel	Pharmacodynamics of Dapagliflozin in Dogs Using an Experimental Model of Metabolic Syndrome
ESVCN – European Society of Veterinary Comparative Nutrition		
ESVCN‐P‐1	Hancock	Adult dogs maintain body lean consuming food containing 0.59%/kg total sulfur amino acids
ESVCN‐P‐2	Pignataro	Modulation of the canine gut microbiota with prebiotic, probiotic, and postbiotic supplementation as assessed in the Mucosal Simulator of the Canine Intestinal Microbial Ecosystem (M‐SCIMETM)
ESVCN‐P‐3	Mullis	Quality of life supported in cats with malignant cancer with a new high calorie, nutrient dense food
ESVCN‐P‐4	Pignataro	A novel investigation into the impact of probiotic dietary supplementation on the metabolic activities of the gut microbiota of a healthy canine donor
ESVCN‐P‐5	Gonçalves Tozato	Urine dipstick tests to evaluate urine pH in dogs and cats
ESVCN‐P‐6	Bela	Effects of Limosilactobacillus reuteri DSM 32203 consumption on body weight, body condition score, faecal parameters, and intestinal microbiota of healthy dachshund dogs
ESVCN‐P‐8	Morris	Lean and fat mass changes with age in dogs and cats fed a controlled protein, amino acid‐supplemented food are comparable to a previously established baseline
ESVCN‐P‐9	Kragh Jørgensen	A cross‐sectional study of feeding practices in lean and heavy/obese cats
ESVCN‐P‐10	Navarro	Effect of a high‐protein, high‐fiber diet consumption, weight loss, and the engagement of the tutors on blood metabolite profiles of adult dogs
ESVCN‐P‐11	Zentek	Nutritional Studies on the Use of Legumes, Wheat and Quinoa in the Diet of Dogs
ESVCN‐P‐12	Corbee	Adverse reactions to food in dogs, what are possible (nutritional) risk factors?
ESVCN‐P‐13	Santos	Use of Cannabidiol (CBD) Supplements in Small Animals Practice in Portugal

## ORAL RESEARCH COMMUNICATIONS

**ESVC – European Society of Veterinary Cardiology**

## ESVC‐O‐1

## Six‐lead electrocardiography in predicting subclinical hypertrophic cardiomyopathy in cats

### L. Gardner^1^, C. Partington^1^, J. Novo Matos^1^

#### ^1^Queen's Veterinary School Hospital, Cambridge, United Kingdom

In people, electrocardiography (ECG) is recommended for screening of hypertrophic cardiomyopathy (HCM). In cats, ECG is not recommended as a screening method for HCM, but there is scarce data assessing the ability of ECG to predict HCM in feline patients. We aimed to determine whether ECG variables can predict subclinical HCM.

Single‐centre, retrospective, cross‐sectional study. Normal cats (defined as left ventricular wall thickness (LVWT) ≤5.0 mm) and cats with subclinical (asymptomatic) HCM (LVWT ≥6.0 mm) were enrolled, and blinded assessment of the following ECG variables was performed: P wave amplitude and duration, PQ interval, presence of delta, Q and S waves, R wave amplitude, QRS duration, presence of fragmented QRS, R‐to‐peak time, T wave polarity and amplitude, T/R ratio, RT concordance, QT interval and presence of ST elevation. We used the Holm‐Sidak method to adjust the *P* value for multiple comparisons. Binary logistic regression was used to identify ECG variables associated with HCM. Receiver operating characteristic curves were used to determine area under the curve (AUC) as well as sensitivity and specificity for selected ECG variables on multivariable analysis. Data are presented as median [range], odds ratio (OR) and AUC (95% confidence interval).

One‐hundred and twelve cats were enrolled, 61 normal and 51 with subclinical HCM. R wave amplitude was higher in HCM cats compared to normal cats (0.3 [0.06–0.98] mV vs. 0.2 [0.01–0.8] mV, *P* = 0.01). On multivariable logistic regression a higher R wave amplitude (OR 15.8 (2.1–153)) was the only independent predictor of HCM after adjusting for body weight, with an AUC of 0.80 (0.71–0.88). R wave amplitude as a sole screening test for HCM (AUC 0.64 (0.54–0.75)) had a 65% sensitivity and 62% specificity to detect HCM when R wave >0.26 mV (Youden's index) or 90.2% sensitivity and 11.5% specificity when R wave >0.1 mV (high sensitivity cut‐off). These results suggest that R wave amplitude has a poor ability to discriminate between normal and HCM cats.

Our results suggest that a 6‐lead ECG is a poor screening tool to predict subclinical HCM in cats.

**Disclosures**

**Source of Funding**

**ECVIM CSF Funding**

No.

**ESVC – European Society of Veterinary Cardiology**

## ESVC‐O‐2

## New electrocardiographic algorithm for the diagnosis of fast supraventricular tachycardias in dogs

### R. Santilli^1,2^, A. Maffei^1^, F.M. Colombo^3^, S. Battaia^4^, M. Bagardi^1,5^, M. Perego^1^

#### ^1^Clinica Veterinaria Malpensa Anicura Cardiovascular division, Samarate, Varese, Italy; ^2^Cornell University Cardiovascular division, Ithaca, NY, United States; ^3^University of Milan Department of Veterinary Sciences for Health,Animal production and food security, Lodi, Italy; ^4^Ospedale Veterinario I Portoni Rossi, Anicura Cardiovascular division, Zola Predosa (BO), Italy; ^5^Department of Veterinary Medicine and Animal Sciences, University of Milan Cardiovascular division, Lodi, Italy

Prompt differential diagnosis of supraventricular tachycardias (SVT) is crucial to patient management. However, differentiating sinus tachycardia (ST), focal atrial tachycardia (FAT), orthodromic atrioventricular reciprocating tachycardia (OAVRT) and atrial flutter (AFl) remains problematic.

The aim of the study is to test the accuracy and reproducibility of an electrocardiographic algorithm for the differentiation of SVT.

Ten 12‐lead ECG tracings with a confirmed SVT diagnosis via electrophysiological mapping were analyzed by two different groups, 58 cardiology specialists (CS) and 5 cardiology residents (CR), using an algorithm based on: identify the presence of SVT (heart rate > 180 bpm, QRS complex < 70ms), recognize atrioventricular conduction ratio, determine regular/irregular ventricular cycle length, recognize the atrial deflection (AD) and its polarity in lead II, determine R wave‐AD interval duration and R wave‐AD/AD‐R wave.

Accuracy and reproducibility of algorithm were estimated as follow. The accuracy estimator was the percent of patients with exact prediction of SVT type; the reproducibility estimator was the coefficient of agreement (a “Kappa‐like” coefficient) between two groups of raters. The above estimations were respectively performed with a mixed effect logistic regression and an intergroup agreement method. “Kappa‐like” coefficients (Kappa) below 0.40, from 0.40 to 0.70 and over 0.70 were considered to indicate poor, fair and excellent agreement, respectively, and p values (p) <0.05 were considered statistically significant. Below the mean estimates and their 95% confidence intervals (in brackets) are shown.

The accuracy for the diagnosis of ST in CS group was 0.97 (0.93,0.98) and in CR group was 0.89 (0.76,0.95) with a statistically significant difference between CS and CR groups (*p* = 0.001) and a Kappa of 0.62 (0.60,0.63). The accuracy for FAT in CS group was 0.75 (0.62,0.85) and in CR group was 0.79 (0.63,0.90) with no statistically significant difference between CS and CR groups (*p* = 0.47) and a Kappa of 0.303 (0.297,0.309). The accuracy for OAVRT in CS group was 0.88 (0.80,0.93) and in CR group was 0.87 (0.74,0.94) with no statistically significant difference between CS and CR groups (*p* = 0.95) and a Kappa of 0.626 (0.62,0.63). The accuracy for AFl in CS group was 0.90 (0.82,0.94) and in CR group was 0.89 (0.76,0.95) with no statistically significant difference between CS and CR groups (*p* = 0.8) and a Kappa of 0.069 (0.066,0.073).

The proposed electrocardiographic algorithm offers an accurate method to classify SVT, even if a specialty training is needed to improve knowledge to recognize AD appearance.

**Disclosures**

**Source of Funding**

**ECVIM CSF**

**ESVC**—**European Society of Veterinary Cardiology**

## ESVC‐O‐3

## Twelve‐lead electrocardiographic differentiation of monomorphic ventricular tachycardia versus bundle branch block in dogs

### A. Maffei^1^, M. Perego^1^, D. Cavallini^2^, R. Santilli^1,3^

#### ^1^Clinica Veterinaria Malpensa Anicura Cardiovascular division, Samarate, Varese, Italy; ^2^University of Bologna Department of Veterinary Medical Sciences, Bologna, Italy; ^3^Cornell University Cardiovascular division, Ithaca, NY, United States

Differential diagnosis of wide QRS complex tachycardia represents a challenge in human and veterinary medicine. Monomorphic ventricular tachycardia (MVT) must be differentiated from supraventricular tachycardia with bundle branch block (BBB). This study aims to describe the different ECG characteristics of MVT and BBB in12‐lead.

103 12‐lead‐ECG tracings were retrospectively analyzed: 18 sinus rhythm and complete right BBB (SR‐RBBB), 18 sinus rhythm and complete left BBB (SR‐LBBB), 33 MVT with RBBB appearance (MVT‐RBBB), 34 MVT with LBBB appearance (MVT‐LBBB). For each ECG the following parameters were analyzed in three consecutive beats: QRS complex duration, morphology and axis on the frontal plane and concordance in standard and precordial leads. Quantitative data distribution was assessed with the Shapiro‐Wilk test, non‐normal distributed data were normalized using box‐cox transformation and analyzed using repeated measure mixed model procedure. Qualitative data were analyzed with a logistic model and odds ratio.

Median age and weight for SR‐RBBB were 9 (1‐14) years and 28.5 (3–53) kg, for SR‐LBBB 8 (0.5–12) years and 28.5 (12–57) kg, for MVT‐RBBB were 9 (0.5–16) years and 28 (6–54) kg, for MVT‐LBBB 8 (0.4–13) years and 25.5 (3–61) kg. QRS complex in lead II was wider in SR‐RBBB than MVT‐RBBB (*p* = 0.0268), and not different between SR‐LBBB and MVT‐LBBB (*p* = 0.3718). QRS complex in V1 in SR‐RBBB had 19.3 odds ratio to show an RR’ morphology compared to MVT‐RBBB. Concordance in leads I, II, III and aVF was present in 100% of SR‐LBBB and in 88% of SR‐RBBB. Precordial leads discordance was present in 100% of SR‐RBBB and SR‐LBBB with transition zone V1‐V2 in 100% of cases, in 78.8% of MVT‐RBBB with transition zone V1‐V2 in 61.5%, and in 88.2% of MVT‐LBBB with transition zone V1‐V2 in 80%. Lead aVR and V1 concordance was present in 100% of SR‐RBBB and SR‐LBBB, in 84.8% of MVT‐RBBB and 94.1% of MVT‐LBBB. R‐Peak Time in V1 was longer in SR‐RBBB than SR‐LBBB (p<.0001) and in MVT‐RBBB than MVT‐LBBB (p<.0001). R‐peak Time in V5 was longer in SR‐LBBB than SR‐RBBB (p<.0001) and in MVT‐LBBB than MVT‐RBBB (*p* = 0.0002). Main limitations were the sample size which did not allow the thoracic morphotypes subgrouping, and MVT localization throughout endocardial mapping.

This study describes the usefulness of 12‐lead ECG to help clinicians to discern wide QRS complex in MVT and BBB in dogs.

**Disclosures**

**Source of Funding**

**ECVIM CSF Funding**

No.

**ESVC**—**European Society of Veterinary Cardiology**

## ESVC‐O‐4

## Three step‐based electrocardiographic algorithm for the identification of accessory pathway location in dogs with ventricular pre‐excitation

### M. Perego^1^, M. Bagardi^1^, F.M. Colombo^2^, R. Pariaut^3^, N.S. Moïse^3^, S.F. Lombardo^4^, R. Santilli^3,4^

#### ^1^Clinica Veterinaria Malpensa Anicura Cardiovascular Division, Samarate, Italy; ^2^University of Milan Department of Veterinary Sciences for Health, Animal Production and Food Securit, Lodi, Italy; ^3^Cornell University, College of Veterinary Medicine, Ithaca, United States; ^4^Clinica Veterinaria Malpensa Anicura, Samarate, Italy

Prior knowledge of accessory pathway (AP) location facilitates an individualized ablation strategy. Analysis of QRS complex morphology on 12‐lead ECGs can predict AP location in dogs. The aim of the study is to test the accuracy and reproducibility of a new electrocardiographic algorithm. Ten 12‐lead ECG tracings with a confirmed AP via electrophysiological mapping were analyzed by two different groups, 63 cardiology specialists (CS) and 5 cardiology residents (CR), using a new 3‐step electrocardiographic algorithm: step 1: identify presence of ventricular pre‐excitation (short PQ interval, wide QRS complex, delta wave); step 2: recognize negative polarity of the QRS complex in V1 identifying a right‐sided AP; step 3: determine mean electrical axis of the QRS complex in the frontal plane defining anterior (normal axis) or posterior (leftward deviation) APs. Accuracy and reproducibility of the algorithm were estimated as follows. The accuracy estimator was the percent of tracings with exact prediction of AP location; the reproducibility estimator was the coefficient of agreement (a “Kappa‐like” coefficient) between two groups of raters. The above estimations were respectively performed with a mixed effect logistic regression and an intergroup agreement method. “Kappa‐like” coefficients (Kappa) below 0.40, from 0.40 to 0.70 and over 0.70 indicated poor, fair and excellent agreement, respectively. P‐values (p) < 0.05 were considered statistically significant. Mean values and 95% confidence intervals (in brackets) are reported. For step 1, accuracy in CS group was 0.91 (0.84,0.95) and in CR group was 0.84 (0.70,0.92) (*p* = 0.018), and Kappa of 0.038 (0.037,0.038) indicated a poor agreement between the groups. For step 2, accuracy in CS group was 0.94 (0.89,0.96) and in CR group 0.96 (0.89,0.99) (*p* = 0.286), and Kappa of 0.72 (0.724,0.725) consistent with an excellent between group agreement. For step 3, accuracy in CS group was 0.75 (0.63,0.85) and in CR group 0.67 (0.48, 0.81) (*p* = 0.127), and there was poor between group agreement with a Kappa of 0.3481 (0.3476,0.3485). The proposed 3‐step electrocardiographic algorithm represents an accurate tool for predicting location of APs in dogs, even if a specialty training is needed to improve knowledge to recognize VPE patterns.

**Disclosures**

**Source of Funding**

**ECVIM CSF Funding**

No.

**ESVC**—**European Society of Veterinary Cardiology**

## ESVC‐O‐5

## clinical utility of pharmacological defibrillation during canine mitral valve repair

### Y. Nii^1^, K. Kurogochi^1^, M. Uechi^1^

#### ^1^JASMINE Veterinary Cardiovascular Medical Center, Yokohama, Japan

Ventricular fibrillation (VF), which commonly occurs during open‐heart surgery in dogs and humans, is associated with perioperative mortality. VF commonly requires electrical defibrillation, which carries the risk of myocardial damage. Although pharmacological defibrillation using crystalloid cardioplegic solution is a potential treatment option, its effectiveness and appropriate methods remain unclear in dogs. This study aims to examine the clinical utility of pharmacological defibrillation in dogs undergoing mitral valve repair.

We retrospectively examined the records of 402 client‐owned dogs that underwent mitral valve repairs between February and December 2021.

VF after cross‐clamp release occurred in 71 cases (17.7%). Pharmacological defibrillation was attempted by the administration of crystalloid cardioplegic solution via an aortic root catheter. If defibrillation was not achieved or VF recurred, the administration of the crystalloid cardioplegic solution was repeated. In all cases, the VF was resolved using pharmacological defibrillation alone, obviating the need for electrical defibrillation. A multiple logistic regression analysis was conducted to explore risk factors for VF. The occurrence of VF was associated with preoperative body weight (odds ratio [OR], 1.17; 95% confidence interval [CI], 1.01–1.36), preoperative normalized left ventricular internal diameter in diastole (OR, 5.01; 95% CI, 1.69–14.83), and esophageal body temperature at the time of cross‐clamp release (OR, 0.74; 95% CI, 0.59–0.92). Defibrillation was achieved with a single dose in 47 cases (median, 3.89 mL/kg [interquartile range (IQR), 2.96–4.91 mL/kg]), while multiple doses (range, 2–5) were required for the other 24 cases (median initial dose, 3.05 mL/kg [IQR, 2.07–4.14 mL/kg]). The discharge rates for VF and non‐VF cases were 98.6% (70/71) and 97.0% (321/331), respectively (*P* = 0.723).

Pharmacological defibrillation is a feasible option in dogs with intraoperative VF undergoing mitral valve repair. Single to multiple doses of crystalloid cardioplegic solution (starting at approximately 3.00 mL/kg) may replace electrical defibrillation techniques. A single administration (4.0 mL/kg) can be effective, whereas lower doses may require multiple administrations. Assessments of myocardial damage are required to determine whether pharmacological defibrillation surpasses electrical defibrillation.

**Disclosures**

**Source of Funding**

**ECVIM CSF Funding**

No.

**ESVC**—**European Society of Veterinary Cardiology**

## ESVC‐O‐6

## Echocardiographic indices of reverse cardiac remodeling and outcome predictors in dogs undergoing mitral valve repair

### B. Atkinson^1^, V. Luis Fuentes^2^, D. Brockman^2^, M. Rossanese^2^

#### ^1^Royal Veterinary College Clinical Science and Services, London, United Kingdom; ^2^Royal Veterinary College, London, United Kingdom

Myxomatous mitral valve disease is a prevalent cardiac condition in dogs. Mitral valve repair (MVR) under cardiopulmonary bypass is a well‐established procedure for this condition with prolonged survival compared to medical management alone. This study aimed (1) to assess the degree and timescale of reverse cardiac remodeling in dogs undergoing MVR; (2) to identify demographic and perioperative echocardiographic variables predictive of incomplete reverse cardiac remodeling post‐surgery.

This was a retrospective longitudinal study of dogs undergoing MVR with ≥12 months’ follow‐up at a single center. Reverse remodeling based on left atrial to aortic ratio (LA:Ao) and left ventricular diameter at end‐diastole normalized to body weight (LVIDDn) was assessed using repeated measures ANOVA. Logistic regression was applied to identify possible demographic and perioperative echocardiographic predictors of incomplete reverse remodeling.

Sixty‐six dogs that underwent MVR were included. Reduction in LA:Ao (2.46 ± 0.61 vs. 1.62 ± 0.31], *P* < 0.001) and LVIDDn (2.17 ± 1.24 vs. 1.64 ± 0.33, *P* < 0.001) was observed within 3 months after surgery, with minimal reduction thereafter. Higher body weight (*P* = 0.006) and increased pre‐operative left ventricular end‐systolic volume indexed to body surface area (ESVI) (*P* = 0.006) were associated with incomplete reverse remodeling.

In conclusion, this study suggests that most reverse remodeling occurs within the first 3 months after MVR and reduced remodeling may be expected in dogs with higher body weight and increased pre‐operative ESVI. These results may identify prognostic markers for long‐term outcome in dogs undergoing MVR.

**Disclosures**

**Source of Funding**

**ECVIM CSF Funding**

No.

**ESVC**—**European Society of Veterinary Cardiology**

## ESVC‐O‐7

## Tricuspid regurgitation velocity improves after mitral valve repair in dogs but is not associated with long‐term mortality

### A. Shinoda^1^, K. Kurogouchi^1^, M. Uechi^1^

#### ^1^JASMINE Veterinary Cardiovascular Medical Center, Yokohama, Japan

Mitral valve repair (MVR) is a potential curative treatment for myxomatous mitral valve disease (MMVD) in dogs. Although an increase in the tricuspid regurgitation velocity (TRV) is commonly observed secondary to MMVD, the prognostic relevance of this phenomenon for MVR remains unclear.

This study aimed to investigate the relationship between the degree of TRV and MVR outcomes and to provide information regarding indications for MVR.

We retrospectively analyzed data from cases of dogs with MMVD and tricuspid regurgitation with a history of congestive heart failure who underwent MVR at our hospital between February 2017 and December 2020.

We classified the cases into three groups depending on the degree of TRV: mild (<3.4 m/s), moderate (3.4–3.7 m/s), and severe (>3.7 m/s). Echocardiographic parameters were evaluated preoperatively and 6‐ and 12‐months postoperatively.

A total of 408 dogs (mild, *n* = 300; moderate, *n* = 44; severe, *n* = 62) were included in the study. In moderate and severe cases, TRV was significantly decreased after surgery, from 3.54 m/s (3.47–3.62 m/s) to 3.30 m/s (2.95–3.59 m/s), *P* < 0.01; and from 4.10 m/s (3.90–4.35 m/s) to 3.17 m/s (2.78–3.59 m/s), *P* < 0.01, respectively. The three groups did not show a difference in the three‐year survival rate for all‐cause mortality based on the Kaplan‐Meier curve: 73%, 60%, and 79%, respectively (*P* = 0.14; log‐rank test).

In this study, the degree of TRV was not associated with long‐term mortality; however, the degree of TRV improved after MVR.

**Disclosures**

**Source of Funding**

**ECVIM CSF Funding**

No.

**ESVC**—**European Society of Veterinary Cardiology**

## ESVC‐O‐8

## Trigone‐based sizing method and proposal for mitral valve annuloplasty surgery in dogs

### N. Isayama^1,2^, T. Mizuno^3,4^, S. Suzuki^5,6^, K. Sasaki^6,7^, E. Maeda^6,7^, Y. Uchimura^6,7^

#### ^1^Uenonomori Animal Hospital Cardiology, Tokyo, Japan; ^2^Tokyo Animal Cardiothoracic Surgery (TACTS) Cardiology, Tokyo, Japan; ^3^The University of Tokyo Veterinary Medical Center, Tokyo, Japan; ^4^Tokyo Animal Cardiothoracic Surgery (TACTS) Veterinary Medical Center, Tokyo, Japan; ^5^Japan Small Animal Medical Center, Saitama, Japan; ^6^Tokyo Animal Cardiothoracic Surgery (TACTS), Tokyo, Japan; ^7^Uenonomori Animal Hospital, Tokyo, Japan

In recent years, surgical treatment for mitral regurgitation in dogs has become popular and includes a combination of annuloplasty and chordae tendineae reconstruction. Although annuloplasty products designed for human patients are commercially available, replicating the surgical insertion of these in dogs is difficult because of the large size variation among dog breeds, lack of clear guidelines, and insufficient literature regarding appropriate annulus size selection.

Consequently, we adopted the trigone‐based diameter method derived from human medical practices to determine the optimum annuloplasty size, based on measurements between the trigones. A retrospective analysis of 30 surgical cases revealed a strong correlation between the measured values of trigone and middle of anterior + posterior leaflet (mA2P2) with body weight (*r* = 0.90 and 0.83, respectively). The trigone measured 58.2% (interquartile range, IQR: 53.2–64.3) of mA2P2 leading to our implementation of annuloplasty to approximately 60% (IQR: 55.6–64.6) of mA2P2. Echocardiographic assessments at 1‐, 6‐, and 12‐months post‐surgery demonstrated no increase in the mean pressure gradient or worsening of tricuspid regurgitation, and marked reductions in left atrial and ventricular dimensions were obtained due to reduced regurgitation.

Subsequently, we evaluated these measurements against those of healthy dogs and the potential predictions based on preoperative parameters. To ascertain the normal valve diameter, we examined 30 healthy dogs, focusing on parameters correlating with body weight. These parameters included the valve ring diameter (4chMV), left ventricular outflow tract (LVOT), Valsalva, and combined length of the anterior and posterior mitral leaflets as measured on echocardiography (eA2P2) (*r* = 0.93, 0.86, 0.85, and 0.89, respectively) via right parasternal long axis view. LVOT:4chMV and Valsalva:4chMV ratios were 1:1.24 (IQR 1.1–1.4) and 1:0.93 (IQR 0.9–1.0), respectively, revealing that 4chMV constituted 65.5% (IQR 60.7–69.7) of the eA2P2. Analysis of the 30 surgical cases showed that preoperative echo measurements of LVOT, Valsalva, and eA2P2 were strongly correlated with body weight (*r* = 0.90, 0.89, and 0.87, respectively). LVOT*1.24 constituted 64.5% (IQR 56.9–70.7) of mA2P2 and 63.9% (IQR 59.3–67.3) of eA2P2, whereas Valsalva*0.95 constituted 65.2% (IQR 57.1–72.3) of mA2P2 and 64.5% (IQR 59.0–70.4) of eA2P2.

Based on these positive long‐term outcomes with trigone‐based sizing and comparisons with healthy dogs, we recommend using the valve ring diameter determined from pre‐surgical estimations of LVOT*1.24, Valsalva*0.95, and eA2P2*65%. The actual ring size should be determined intraoperatively based on trigone and mA2P2 measurements to ensure optimal sizing and outcomes.

**Disclosures**

**Source of Funding**

**ECVIM CSF Funding**

No.

**ESVC**—**European Society of Veterinary Cardiology**

## ESVC‐O‐9

## Three‐dimensional echocardiographic determinants of the age of onset of myxomatous mitral valve disease in Cavalier King Charles Spaniels dogs

### A. Franchini^1^, G. Menciotti^2^, H. Jeong^3^, S. Lahmers^4^, M. Borgarelli^4^

#### ^1^Virginia Maryland College of Veterinary Medicine Small Animal Clinical Science, Blacksburg, VA, United States; ^2^Virginia Maryland College of Veterinary Medicine Small Animal Clinical Science, Blacksburg, VA, United States; ^3^NorthStar Vets, Robbinsville, NJ, United States; ^4^Virginia Maryland College of Veterinary Medicine, Blacksburg, VA, United States

This study investigated whether mitral valve (MV) morphological variables obtained by three‐dimensional transthoracic echocardiography (3DTTE) could predict the age of onset of myxomatous mitral valve disease (MMVD) in healthy Cavalier King Charles Spaniels (CKCSs).

CKCSs without echocardiographic evidence of MMVD were prospectively enrolled. Standard echocardiogram and 3DTTE were performed at enrollment and every four months until the development of MMVD or 24 months from enrollment. Dedicated software was used to analyze the 3DTTE datasets and assess MV morphologic variables. Cox's proportional hazard models were used to investigate the effect of 3DTTE MV morphologic variables at enrollment on the time to onset of MMVD. A multivariable model was also created with backward stepwise selection to identify independent predictors of the age of onset of MMVD.

MV morphologic variables of 76 CKCSs with median age of 2 (range: 1–3 years) were analyzed. Non‐planar angle (NPA) (*p*<0.001, hazard ratio (HR) 1.05, 95%, confidence interval (CI):1.02–1.09), tenting area (*p* = 0.001, HR: 0.55, 95% CI: 0.40–0.76), and tenting volume normalized by body weight (nTnV) (*p*<0.001, HR: 0.81, 95% CI: 0.69–0.94) were significant in the univariable models. NPA (*p*<0.001, HR: 1.05, 95% CI: 1.02–1.09) and nTnV (*p* = 0.009, HR: 0.82, 95% CI: 0.71–0.95) remained significant in the multivariable model.

This study demonstrated that some MV variables obtained with 3DTTE predict the early onset of MMVD in healthy CKCSs. Specifically, the lower the value of nTnV, the sooner the onset of MMVD. Therefore, 3DTTE could be used in screening and breeding programs to reduce the prevalence of MMVD in CKCSs.

**Disclosures**

M. Borgarelli and G. Menciotti receive research and travel support by Ceva Sante Animale. They are also consultants for Ceva Sante Animale. A. Franchini received salary support from Ceva Sante Animale and American Kennel Club for her PhD. She also receives travel support by Ceva Sante Animale.

**Source of Funding**

This study was fully funded by the American Kennel Club.

**ECVIM CSF Funding**

No.

**ESVC**—**European Society of Veterinary Cardiology**

## ESVC‐O‐10

## Assessment of pulmonary veins diameter in cavalier King Charles Spaniels with degenerative mitral valve disease

### C. Ferri^1^, J. Besso^2^, H. Gaillot^1^, Y. Ruel^1^, A. Agoulon^3^, C. Bourguignon^1^, C. Mey^1^, V. Gouni^1^

#### ^1^Veterinary Hospital ADVETIA, Vélizy‐Villacoublay, France; ^2^Vetradmobile, Paris, France; ^3^Oniris, INRAE, BIOEPAR, Nantes, France

In the treatment of dogs with degenerative mitral valve disease (DMVD), diuretics are crucial to prevent life‐threatening complications. The optimal time to add diuretics to the treatment is difficult to assess in early stages of left‐sided congestive heart failure (CHF). The objectives of this study are to: 1) compare pulmonary vein (PV) diameter in heathy Cavalier King Charles Spaniels (CKCS) with PV diameter in CKCS with DMVD at various stages of the ACVIM classification, 2) correlate PV diameter to several clinical and Doppler echocardiographic parameters, and 3) determine the optimal threshold PV diameter distinguishing between dogs in stage B2 and stage C of the disease.

This study includes 100 CKCS: 28 healthy controls and 72 dogs with DMVD. All dogs received a Doppler echocardiographic exam, PV diameter measurement, and 2 orthogonal thoracic radiographs. Dogs were scored as stage A, B1, B2 or C. PV diameter was measured in bidimensional (2D) mode from a left apical view at end systole using the inner edge‐to‐inner edge method. Three PVs were visible and their diameter recorded as PV1, PV2 and PV3.

Inter‐ and intra‐observer coefficients of variation of the measurement of the three veins were <10%. There was no significant correlation between PV2 and body weight in stage A (rho=0.24, *p* = 0.247); thus, PV2 was used to compare groups. There was no significant difference of PV2 between stage A (*n* = 28; median, interquartile range; 4.9 mm, 3.9‐5.2 mm), and stage B1 (*n* = 21; 5.1 mm, 4.0‐6.0 mm, *p* = 0.24). There was a significant increase of PV2 between stages B1 and B2 (*n* = 29; 9.3 mm, 7.3–11.1 mm, *p*<0.001) and between B2 and C (*n* = 22, 13.7 mm, 9.9‐15.1 mm, *p* = 0.003). A significant positive correlation was observed between PV2 and the left ventricular internal diameter normalized to body weight, the left atrium‐to‐aorta ratio, the tricuspid regurgitation gradient, the ejection fraction, the E wave velocity, and the E/A ratio. The optimal PV2 cut‐off discriminating between stages B2 and C was set at 12.8 mm (area under the curve, 0.753; 95% CI, 0.61–0.90) with sensitivity of 57% and a specificity of 93%.

In conclusion, PV2 diameter is a reproducible echocardiographic parameter, which increases with the progression of DMVD in CKCS. In this breed, it can be used as an additional parameter to identify dogs in early CHF and help in the decision‐making process for starting treatment with diuretics.

**Disclosures**

**Source of Funding**

**ECVIM CSF Funding**

No.

**ESVC**—**European Society of Veterinary Cardiology**

## ESVC‐O‐11

## Comparison of left ventricular wall measurements obtained from different echocardiographic views: A study in 408 cats

### J. Novo Matos^1^, V. Luis Fuentes^2^

#### ^1^University of Cambridge University of Cambridge, Cambridge, United Kingdom; ^2^Royal Veterinary College, London, United Kingdom

The reliability of measurements of left ventricular (LV) wall thickness (LVWT) is paramount in phenotyping cardiomyopathies in cats. However, clinicians have varied preferences for which echocardiographic views are used to measure LVWT, with a lack of data assessing whether LVWT measurements from different views are interchangeable. We aimed to assess the agreement of LVWT measurements between different echocardiographic views and techniques in cats.

Echocardiography of 408 cats was performed and measured by a single observer. LVWT of the septum and free wall were measured from two‐dimensional (2D) right parasternal long‐axis (2D‐RPLA) 4‐chamber and 5‐chamber views, and from a 2D right parasternal short‐axis view (2D‐RPSA) and M‐mode RPSA view (MMode‐RPSA). Bland‐Altman analysis was used to assess agreement of LVWT measurements between echocardiographic views in the whole cohort, and separately in cats with LVWT <6 mm (*n* = 292) and LVWT ≥6 mm (*n* = 116). Data are presented as median [range].

LVWT for both the septum and free wall was greater in 2D‐RPLA (4‐chamber and 5‐chamber) when compared to 2D‐RPSA in both study groups (MaxLVWT 6.7 [4.7–11.1] mm vs. 6.1 [3.5–12.3] mm in cats with LVWT ≥6 mm, *P* < 0.0001; 4.6 [3.3–5.9] mm vs. 4.1 [3.0–5.9] mm in cats with LVWT <6 mm; *P* < 0.0001). LVWT of the free wall was greater in MMode‐RPSA than 2D‐RPSA in the whole cohort (4.4 [2.7–9.2] mm vs. 4.2 [3.0‐9.3] mm, *P*=0.0003). Bland‐Altman analysis assessing 2D‐RPLA vs. 2D‐RPSA showed mild proportional bias with wide limits of agreement, with increased disagreement at greater LVWT (heteroscedasticity) for both septum and free wall measurements. There was a small, fixed bias (−0.04 mm) between 2D‐RPSA and MMode‐RPSA with wide 95% limits of agreement for the septum (−1.1 to 1.1 mm), and with heteroscedasticity for the free wall. Assessment of agreement between 4‐chamber and 5‐chamber 2D‐RPLA views showed a small, fixed bias for both the septum (−0.09 mm) and free wall (−0.03 mm) with wide 95% limits of agreement (septum −0.8‐0.7 mm, free wall −0.9 to 0.8 mm). Intra‐observer repeatability was excellent with low measurement variability for LVWT (ICC 0.90, %CV 5%).

Our study suggests that assessment of LVWT in different echocardiographic views is not interchangeable, as the limits of agreement between views were wide. This could affect cardiac phenotyping. Our results mirror human data and highlight the need for establishing echocardiographic guidelines in cats.

**Disclosures**

**Source of Funding**

**ECVIM CSF Funding**

No.

**ESVC**—**European Society of Veterinary Cardiology**

## ESVC‐O‐12

## Mitral to aortic velocity‐time integral ratio to estimate the regurgitant fraction in dogs with myxomatous mitral valve disease

### S. Oricco^1^, C. Quintavalla^1^, M. Poggi^2^, I. Apolloni^1^, S. Crosara^1^

#### ^1^University of Parma Department of Veterinary Sciences, Parma, Italy; ^2^Centro Veterinario Imperiese, Imperia, Italy

The regurgitant fraction (RF) magnitude is associated with the severity of myxomatous mitral valve disease (MMVD). However, echocardiographic methods to estimate RF take time and effort. In humans, the mitral to aortic velocity‐time integral ratio (VTI_Mit_/VTI_Ao_) has been used as a surrogate of RF to evaluate post‐operative follow‐up. This study aimed to assess the correlation between VTI_Mit_/VTI_Ao_ and RF, calculated by the difference between total and forward stroke volumes, in dogs. Additionally, it sought to observe how VTI_Mit_/VTI_Ao_ changes in both normal and MMVD dogs and how it correlates with some echocardiographic indexes. This retrospective study involved a review of the medical records from the digital database of two centers. All the data were analyzed with non‐parametric tests. The selected exams from normal and MMVD dogs with VTI_Mit_/VTI_Ao_ were 644: 101 normal dogs and 543 with MMVD (145 in class ACVIM B1, 176 in class ACVIM B2, 222 in class ACVIM C and D). The correlation between VTI_Mit_/VTI_Ao_ and RF, calculated using the Simpson's method and conducted on a subpopulation of 66 dogs, was moderate (rho 0.69, 95% CI: 0.53–0.80, *p* < 0.001), while the correlation between VTI_Mit_/VTI_Ao_ and RF, calculated using the Teichholz formulas and conducted on a subpopulation of 223 dogs, was strong (rho 0.8, 95% CI: 0.75–0.84, *p* < 0.001). The Kruskal‐Wallis, after Dunn post‐hoc analysis with Bonferroni correction, showed a significant difference for VTI_Mit_/VTI_Ao_ between all the ACVIM stages (*p* < 0.001). The correlations between VTI_Mit_/VTI_Ao_ and other echocardiographic indexes associated with the progression of the disease were as follows: weak for the shortening fraction (rho: 0.39, 95% CI: 0.29–0.47, *p* < 0.001), moderate for the left ventricular end‐diastolic internal diameter normalized for body weight (rho: 0.69, 95% CI: 0.61–0.73, *p* < 0.001), and strong for left atrium‐to‐aortic root ratio (rho: 0.7, 95% CI: 0.65–0.75, *p* < 0.001), left atrial anteroposterior diameter normalized on body weight (rho: 0.72, 95% CI: 0.66–0.77, *p* < 0.001), and the maximal left atrial volume (rho: 0.77, 95% CI: 0.72–0.81, *p* < 0.001). In conclusion, the VTI_Mit_/VTI_Ao_ was found to be correlated with the RF in dogs with MMVD and associated with the progression of the disease. These results suggest that it could be a useful echocardiographic index for estimating RF in dogs with MMVD, due to its simplicity.

**Disclosures**

**Source of Funding**

No funding.

**ECVIM CSF Funding**

No.

**ESVC**—**European Society of Veterinary Cardiology**

## ESVC‐O‐13

## Use of the subcostal view for obtaining aortic spectral Doppler flow velocities in cats

### E. Korzybska^1^, F. Edgerton^2^, S.A. Dickson^3^, M.I. Oliveira^3^, Y. Martinez‐Pereira ^1^, G. Culshaw^1,4^, R. Blake^1,5^

#### ^1^University of Edinburgh Cardiology, Edinburgh, United Kingdom; ^2^University of Edinburgh Cardiology, Edinburgh, United Kingdom; ^3^University of Edinburgh, Edinburgh, United Kingdom; ^4^Petsavers Cardiology, Edinburgh, United Kingdom; ^5^University College of Dublin Cardiology, Dublin, Ireland

In dogs and foals, aortic outflow spectral Doppler flow velocities (AV) obtained from the subcostal view (SC) provide higher values than the left parasternal apical five‐chamber (LAp) view. Whether cats tolerate the SC view is unknown. Usually, the LAp view is used in cats but alignment with blood flow can be challenging.

This study assessed the feasibility of obtaining SC views in cats and compared AV from SC and LAp views. We hypothesized that the SC view would be well tolerated and provide higher AV.

This was a prospective, single‐centre, observational study of cats referred for echocardiography. Standard echocardiographic studies that included LAp‐derived AV, and, where feasible, those from the SC view, were performed. Patient compliance, ease of attainment and need for sedation were recorded. Aortic velocities obtained from both views were compared. The influence of sedation, heart rate, body condition score, bodyweight and left ventricular outflow tract obstruction were determined.

Ninety‐three cats were enrolled. Eight cats were excluded due to incomplete data. Final analysis included 85 cats. Subcostal AV were greater than LAp velocities by 0.179m/s (±SD1.25m/s; *p* = 0.03). Sedation, heart rate, body condition score, bodyweight and left ventricular outflow tract obstruction did not influence the differences between views. Most cats (82%) tolerated the SC view.

In conclusion, SC‐derived AV are greater than those from the LAp view in cats. The SC view is well tolerated and may offer a more accurate assessment of left ventricular outflow tract obstruction severity in some cats.

**Disclosures**

All authors employed by the University of Edinburgh. Geoffrey Culshaw: Research funded by Kidney Research UK and Petsavers Rachel Blake: Receives consulting fees from University College Dublin and IDEXX

**Source of Funding**

**ECVIM CSF Funding**

No.

**ESVC**—**European Society of Veterinary Cardiology**

## ESVC‐O‐14

## Comparison of cardiac output measurements utilizing three‐dimensional transesophageal echocardiography and two‐dimensional transesophageal cardiography

### A. Showers^1^, G. Menciotti^2^, M. Borgarelli^2^, S. Lahmers^2^, V. Paranjape^2^

#### ^1^Virginia‐Maryland College of Veterinary Medicine Department of Small Animal Clinical Sciences, Blacksburg, United States; ^2^Virginia‐Maryland College of Veterinary Medicine, Blacksburg, United States

Transesophageal echocardiography (TEE) can be used for estimating cardiac output (CO) without the need for cardiac catheterization. Aortic area measurements are often considered one of the main sources of inaccuracy of echocardiographic estimates of CO. The objective of this study was to compare CO measurements obtained by using real‐time 3D transesophageal echocardiography to estimate the area of the left ventricular outflow tract (3DTEE_CO_), 2D transesophageal echocardiography (2DTEE_CO_), and pulmonary artery thermodilution (PATD_CO_). In this prospective diagnostic comparison study, six healthy male beagles underwent an experiment in which they were randomly and sequentially administered four different vasoactive drugs and one in which their blood volume was altered using phlebotomy, autotransfusion, and colloid administration. For each intervention, each dog also underwent a modified passive leg raise maneuver. CO was obtained by PATD_CO_ and TEE after each intervention and their respective return to baseline. Bland‐Altman analysis and linear regression were used to compare the three methods. A total of 114 CO measurements were obtained by PATD and 120 were obtained by 2DTEE and 3DTEE. A greater measurement bias was appreciated between PATD_CO_ and 3DTEE_CO_ (−1.32 *P* < 0.0001, LOA (−3.58 to 0.94)) vs. PATD_CO_ and 2DTEE_CO_ (0.27 *P* < 0.0001, LOA (‐1.04 to 1.58). The magnitude of overestimation of 3DTEE_CO_ increased at higher CO values. The relationships between 2DTEE_CO_ and PATD_CO_ and PATD_CO_ and 3DTEE_CO_ were significant (0.41, *P* < 0.0001; 0.54, *P* < 0.0001). In conclusion, while both 2DTEE_CO_ and 3DTEE_CO_ show correlation with PATD_CO_, both modalities are prone to bias with 3DTEE_CO_ significantly overestimating PATD.

**Disclosures**

M. Borgarelli and G. Menciotti receive research and travel support by Ceva Sante Animale. They are also a consultant for Ceva Sante Animale. A. Showers receives salary and travel support from Ceva Sante Animale for her PhD/Residency.

**Source of Funding**

**ECVIM CSF Funding**

No.

**ESVC**—**European Society of Veterinary Cardiology**

## ESVC‐O‐15

## Comparing pulmonic and aortic velocity‐time integral‐derived cardiac output parameters in dogs receiving intravenous fluid challenges: A preliminary study

### C. Kennedy^1^, K. Gommeren^1^, A.C. Merveille^1^

#### ^1^University of Liège, Liège, Belgium

Transaortic Doppler echocardiography may be used to assess cardiac output (CO) and fluid responsiveness (FR). Given the principle of conservation, transpulmonic‐derived parameters could be similar to transaortic‐derived parameters, providing an alternative way to measure CO. This study aimed to evaluate the agreement between transaortic and transpulmonic‐derived CO parameters before and after a fluid challenge and investigate if increases in transaortic stroke volume [SV] ≥15% (i.e. FR) after a fluid challenge are associated with similar transpulmonic measurements. A preliminary analysis was planned at 50 datapoints.

Dogs in the emergency or intensive care services receiving a fluid challenge (7.5 ml/kg of isotonic crystalloids over 5 min) were prospectively enrolled. Dogs were excluded if any arrhythmia (including sinus arrhythmia) was present or the necessary views could not be obtained. Recorded parameters before and after fluid challenge were aortic and pulmonic valve diameter; transvalvular peak flow velocity and velocity‐time integrals; heart rate, and weight. Calculated parameters included SV, SV index, minute distance, CO and cardiac index. Absolute and relative (i.e. delta) changes after the fluid challenge were calculated.

Differences between paired measurements were assessed using paired Student T‐tests. Agreement was assessed using the Intraclass Correlation Coefficient (ICC) and its 95% confidence intervals (95% CI). Variation was assessed using Coefficients of Variation (CV).

Fifty‐three paired datapoints were collected from 36 dogs. The pulmonic valve diameter was greater than the aortic valve (*p* = 0.037). The pre‐fluid SVs showed excellent agreement (ICC [95% CI] = 0.939 [0.915–0.964]) though wide CV (20%); the post‐fluid agreement was similarly excellent (ICC [95% CI] = 0.941 [0.918–0.965]) with wide CV (19.1%). Forty‐one out of fifty‐three paired datapoints demonstrated positive FR (increase in SV range 16%–81%); 12 were FR negative. With FR, the deltas for transaortic peak velocities, velocity‐time integrals, SVs, SV indexes, minute distances, COs and cardiac indexes were larger. The absolute changes in transaortic and transpulmonic‐derived SVs showed poor agreement (ICC [95% CI] = 0.431 [0.217–0.627]) with enormous CV (95.8%); the relative changes showed poor agreement (ICC [95% CI] = 0.143 [−0.129 to 0.382]) and CV (14.8%). There was no association between FR positivity and deltas for any transpulmonic‐derived parameters.

The agreement between transaortic and transpulmonic‐derived CO parameters before and after a fluid challenge was excellent, though the variation was large. Transpulmonic measured and derived CO parameters could not identify positive response to a fluid challenge in dogs in the emergency room or intensive care.

**Disclosures**

**Source of Funding**

None.

**ECVIM CSF Funding**

No.

**ESVC**—**European Society of Veterinary Cardiology**

## ESVC‐O‐16

## Red blood cell glycocalyx damage in canine myxomatous mitral valve disease

### M. Mazzarella^1,2^, M. Butler^3^, R. Foster^3^, J. Ward^1^, M. Tsakiroglou^1^, N. Finch^1,2^, M. Hezzell^1^

#### ^1^Bristol Veterinary School, Langford, United Kingdom; ^2^Bristol Medical School, Bristol, United Kingdom; ^3^Bristol Medical School Bristol Renal, Bristol, United Kingdom

Vascular endothelial glycocalyx (eGlx) damage is associated with endothelial dysfunction in people. Previous studies have shown that endothelial dysfunction is associated with myxomatous mitral valve disease (MMVD) severity in dogs; this might be secondary to eGlx damage. However, direct measurement of the eGlx is challenging. Red blood cell glycocalyx (RBCGlx) depth measured from blood smears is correlated with eGlx depth in experimental rodents and humans; therefore, it may represent an easier way to measure eGlx. We hypothesise that RBCGlx depth decreases with increasing MMVD severity.

This is a prospective, cross‐sectional, observational study. Privately‐owned dogs were enrolled; consent to use the data for research purposes was given by the owners. The following were obtained for each dog: history, physical examination, blood pressure measurement, electrocardiogram, echocardiography and blood samples. Dogs were staged according to the American College of Veterinary Internal Medicine consensus statement. Blood smears were prepared using surplus blood samples. To visualise the RBCGlx a patented method was used. The protocol included blood smears being fixed with methanol and then stained with *Lycopersicon esculentum* lectin (glycocalyx) and octadecyl rhodamine B chloride (R18) (cell membrane stain). A measure of RBCGlx depth was quantified by blinded manual measurement of the distance between the peaks of the green (lectin) and red (R18) fluorescence profiles (peak‐to‐peak). Groups were compared using Kruskall‐Wallis tests with post‐hoc Dunn's tests. Correlations between peak‐to‐peak and echocardiographic variables were assessed using Spearman's *ρ* test. Data are presented as median (minimum‐maximum).

Seventy‐four dogs (9 stage A, 27 stage B1, and 38 stage B2) were enrolled. Peak‐to‐peak measured were 0.1138 nm (0.06048–0.1349 nm), 0.08787 nm (0.005646–0.1445 nm), and 0.05584 nm (0–0.1226 nm) for stage A, B1 and B2 respectively. There was no significant difference in RBCGlx peak‐to‐peak between stage A and B1 dogs (*p* = 0.24). RBCGlx peak‐to‐peak was significantly lower in stage B2 versus both stage A and B1 (*p* < 0.0001 and 0.0006, respectively). Significant correlations were identified between RBCGlx peak‐to‐peak and left atrium‐to‐aorta ratio and end‐diastolic left ventricle internal diameter normalised for the body weight (*p* < 0.0001 for both, *ρ*=‐0.54 and ‐0.63 respectively).

RBCGlx damage correlates with progression of MMVD in dogs, and measurement may differentiate dogs with stage B2 from stage B1 disease.

**Disclosures**

Marco Mazzarella: University of Bristol, Royal Canin, The University of Edinburgh, BSAVA‐PetSavers, The Kennel Club Charitable Trust Matthew Butler: University of Bristol, BHF, Diabetes UK, Kidney Research UK, MRC IAA, MRC Rebecca Foster: University of Bristol, HFCE, BHF, Diabetes UK, Kidney Research UK, MRC IAA, MRC Jade Ward: University of Bristol Metaxia Tsakiroglou: University of Bristol, Greek Government Natalie Finch: University of Bristol, Langford Trust, BSAVA‐PetSavers, Wellcome Trust, IRIS, Ceva Animal Health Melanie Hezzell: Bristol Veterinary School, Langford Trust, PetSavers, American Kennel Club Canine Health Foundation, UKRI (BBSRC), Ceva Animal Health, Boehringer Ingelheim, Chulabhorn Royal Academy, Jean Golding Institute, Cavalier Matters, Defence Science and Technology Laboratory, ACVIM, Academy of Medical Sciences, IDEXX Laboratories Inc, Boehringer Ingelheim

**Source of Funding**

This project was funded by a University of Bristol scholarship.

**ECVIM CSF Funding**

No.

**ESVC**—**European Society of Veterinary Cardiology**

## ESVC‐O‐17

## Echocardiographic left ventricular wall thickness in cats: the role of the breed and the body weight

### V. Patata^1^, T. Vezzosi^2^, D. Caivano^3^, F. Spina^4^, P. Ferrari^4^, I. Alberti^4^, R. Ghinelli^4^, D. Lenoci^4^, P. Knafels^5^, F. Pallaro^4^, F. Porciello^6^

#### ^1^Clinica Veterinaria Privata San Marco Department of Cardiology, Padova, Italy; ^2^University of Pisa, Department of Veterinary Sciences University of Pisa, Department of Veterinary Sciences, Pisa, Italy; ^3^University of Perugia, Department of Veterinary Medicine Cardiology, Perugia, Italy; ^4^Italian Veterinary Observatory for Cardiac diseases, Bergamo, Italy; ^5^Centro Veterinario Gregorio VII, Roma, Italy; ^6^University of Perugia, Department of Veterinary Medicine, Perugia, Italy

Echocardiographic evaluation of left ventricular (LV) wall thickness in cats is essential for screening purposes and for evaluating feline cardiomyopathies. A correlation between LV wall thickness and body weight (BW) has been suggested in healthy cats by one large study using M‐mode (*n* = 19.866) and one small study (*n* = 150) using two‐dimensional (2D) echocardiography. The aim of this study was to investigate the correlation between 2D LV wall thickness and BW in a large sample of healthy purebred cats.

Clinical data were obtained from the database of the Osservatorio Veterinario Italiano Cardiopatie, retrospectively including healthy purebred cats, belonging to 7 different breeds, evaluated for cardiologic screening. The interventricular septal diastolic thickness (IVSd) and the LV posterior wall diastolic thickness (LVPWd) were assessed using 2D echocardiography. The influence of BW on echocardiographic variables was tested using a multivariate model, and the size of correlation was measured.

The study sample included 1271 cats, with 751 females and 520 males. The median age was 1 year (min‐max, 1‐15 years) and the median BW was 5 Kg (min‐max, 2‐11 kg). The breeds included were Maine Coon (*n* = 769), Norwegian Forest (*n* = 134), Siberian (*n* = 125), British Shorthair (*n* = 67), Sphynx (*n* = 67), Ragdoll (*n* = 65), and Birman (*n* = 44). Considering all cats, the LV wall thickness (IVSd and LVPWd) was independently associated with BW according to multivariable analysis (*p* < 0.001). Considering the different breeds, this was confirmed in Maine coon, Norwegian, British shorthair and Ragdoll; in Siberian and Birman cats, only the LVPWd was associated with BW; in Sphynx cats neither IVSd nor LVPWd were associated with BW. The correlation between IVSd and BW was negligible in Maine Coon (*r* = 0.28), and weak in British shorthair (*r* = 0.42), Norwegian (*r* = 0.39) and Ragdoll (*r* = 0.38). The correlation between LVPWd and BW was weak in Maine Coon (*r* = 0.33), British shorthair (*r* = 0.45), Norwegian (*r* = 43), Birman (*r* = 0.40) and Siberian (*r* = 0.32), and moderate in Ragdoll (*r* = 0.60).

Our study confirms the association of the LV wall thickness and BW in cats, but not in all breeds and with a negligible‐to‐weak correlation in most breeds. Thus, the clinical relevance of the influence of BW on LV wall thickness should be interpreted in light of the breed, and breed‐specific reference intervals should be preferred in comparison to general feline reference ranges when screening purebred cats for HCM.

**Disclosures**

**Source of Funding**

**ECVIM CSF Funding**

No.

**ESVC**—**European Society of Veterinary Cardiology**

## ESVC‐O‐18

## Congenital cardiopathies in cats: A retrospective analysis in a single referral centre

### M. Papa^1^, B. Aglieta^2^, A. Luciani^2^, M. Botto^3^, E. Boz^1^, S. Signorelli^1^, C. Rossi^1^, V. Forti^1^, M. Longobardi^1^, C. Pergamo^1^, L. Scarpellini^1^, C. Bussadori^1^

#### ^1^Clinica Veterinaria Gran Sasso, Milan, Italy; ^2^Department of Veterinary Medicine, Veterinary University Hospital, Teramo, Italy; ^3^Independent researcher, Teramo, Italy

The prevalence of congenital cardiopathies in cats is variable, from 0.5% in apparently healthy cat to 5‐15% in cat presenting for veterinary care. The aim of this study was to evaluate retrospectively the prevalence, the distribution and the association of breed and gender with congenital heart diseases (CHDs) in cats in a single referral centre, from January 1997 to June 2023.

The cardiological database was retrospectively analysed, collecting signalment and echocardiographic diagnosis for all cats.

Descriptive and inferential statistics were generated. The association between breed and CHDs, and between gender and CHDs were evaluated through binomial's and Fisher's exact tests. A *p* values < 0.05 were considered statistically significant.

A total of 5445 cats were examined during the defined period: 47.9% of cats were healthy, 47.3% were affected by acquired cardiopathies and CHDs were diagnosed in 263 cats (4.8%). A isolated cardiac disease was present in 224 cats (85.1%) and association of two or more diseases was found in 39 cats (14.9%).

The most common isolated CHD was Ventricular Septal Defect (VSD) with a prevalence of 29%, followed by Pulmonic Stenosis (PS) and Patent Ductus Arteriosus (PDA), with a prevalence of 13% and 11%, respectively. In cats with associated diseases, the association between VSD and PS (valvular or subvalvular) was the most frequently observed (12.8%).

The most represented breeds were Domestic Shorthair (58%), Main Coon (11%) and Persian cat (10%). For some breeds a predisposition to specific CHDs was evidenced: VSD and PDA in Domestic Shorthair (*p* = 0.02 and *p* = 0.001, respectively), Atrioventricular Septal Defect in Ragdoll (*p* = 0.03), Tricuspid Valve Dysplasia in Birman (*p* = 0.01), and Atrial Septal Defect in Exotic Shorthair (*p* = 0.03).

Atrioventricular Septal Defect was overrepresented in female (*p* = 0.001), while for other CHDs any significant differences have been reported for gender.

The average age at diagnosis was 33 months (1–240 months) regarding cats with single defects and 12.9 months in cats affected by associated defects (2–72 months).

This retrospective study evidenced interesting distribution of isolated and associated CHDs, and different association between breeds and gender in CHDs in a single cardiologic referral centre. The age at diagnosis may be influenced by the complexity of CHD and its cardiovascular consequences.

**Disclosures**

**Source of Funding**

**ECVIM CSF Funding**

No.

**ESVC**—**European Society of Veterinary Cardiology**

## ESVC‐O‐19

## The BEAMS Study—Baseline Evaluation And Management of Scatter radiation using RADPAD radiation absorbing drapes during veterinary interventional cardiac procedures

### K. Pierce^1^, K. Williams^2^, B. Bridges^2^

#### ^1^North Carolina State University Clinical Sciences—Cardiology, Raleigh, United States; ^2^North Carolina State University, Raleigh, United States

Ionizing radiation exposure is a notable occupational health risk for interventional cardiologists in human and veterinary medical fields. Interventionists have a significantly increased risk of cataracts, cancer, and DNA damage. Radiation exposure can be mitigated by utilizing protective equipment and limiting fluoroscopy, however, does not address exposure from scatter radiation.

RADPADs are specialized protective drapes that are sterile, disposable, lead‐free, and minimize operator‐received scatter radiation. This study evaluated the efficacy of RADPADs in reducing radiation exposure to catheterization lab personnel during interventional cardiac procedures, including patent ductus arteriosus occlusions (*n* = 9), balloon pulmonary valvuloplasties (*n* = 20) or stent (*n* = 3) and pacemaker implantations (*n* = 5). In human studies, RADPAD drapes decreased radiation exposure to the operator and personnel by up to 59%. We hypothesized that using RADPADs would reduce personnel radiation exposure during interventional cardiac procedures in veterinary patients. In addition to standard Dosimeters, individual Real‐Time Dosimetry badges were worn by residents, faculty cardiologist, transesophageal echocardiographer, anesthetist, and technician. Radiation dose was recorded for procedures with RADPAD drapes (*n* = 37) and without (NOPAD; *n* = 39) for 76 client‐owned patients over a 10‐month period. RADPADs were sterilely placed between the beam and operator. Procedure type, procedural and fluoroscopy times, fluoroscope radiation dose, patient characteristics, and personnel positions were documented.

There was approximately 50% dose reduction in the RADPAD group as compared to the NOPAD group. Given this dose reduction, radiation‐absorbing drapes could be considered part of a future standard of care for radiation safety protocols for interventional procedures and limit radiation risks to veterinary professionals.

**Disclosures**

**Source of Funding**

No funding received. RADPADs purchased with faculty startup funds.

**ECVIM CSF Funding**

No.

**ESVC**—**European Society of Veterinary Cardiology**

## ESVC‐O‐20

## Veterinary cardiologists’ preferences regarding interventional cardiac procedures

### K. Galanakis^1^, L. Locquet^1^, S. Swift^2^

#### ^1^DWR Veterinary Specialists, Six Mile Bottom, United Kingdom; ^2^HS Cardiology, Lancaster, United Kingdom

Currently there is no consensus among veterinary cardiologists on the practices involved in interventional cardiac procedures. This study investigates veterinary cardiologists' preferences in the three commonest interventional cardiology procedures: patent ductus arteriosus (PDA) occlusion, balloon valvuloplasty (BVP) for pulmonic stenosis, and pacemaker implantation. Subgroup analysis based on qualification, experience and work environment aimed to identify variations and factors influencing decision‐making.

An online survey was created, validated and distributed among Veterinary Cardiovascular Society (United Kingdom) members. The survey comprised multiple‐choice questions covering topics such as vascular access methods, antibiotic and lidocaine usage, heparin administration, temporary pacing techniques, postoperative monitoring protocols, length of hospitalisation, follow‐up evaluations and exercise restriction.

The study involved 58 participants with varied interventional case load. Most were ECVIM/ACVIM diplomates (63%), followed by residency trained/residents (25%), RCVS recognised specialists (7%) and other qualifications (5%). Most of the participants reported using antibiotics. One third of the participants administer lidocaine during BVP. Postoperative hospital stays for all three procedures were typically less than 2 days and follow‐up appointments were typically within one month. Exercise restriction recommendations varied, with most advising restriction for 2 weeks or less following PDA occlusion and BVP, and between 2 and 4 weeks following pacemaker implantation. Subgroup analysis revealed a significant (*p* < 0.05) correlation between the timing of first follow‐up appointment and the number of interventions performed annually with those performing a higher volume of interventions tending to schedule follow‐up appointments at later intervals. The number of interventions performed annually had also a significant effect on the temporary pacing method used during pacemaker implantation. Most participants used transthoracic temporary pacing, with more interventions increasing the use of transvenous temporary pacing and fewer interventions increasing the likelihood of no temporary pacing. Subgroup analysis based on work environment showed that participants working in academia were more likely to use postoperative ECG monitoring and advise for more extended hospitalisation compared to their counterparts in private practice. Moreover, academia‐based participants were more inclined to measure intracardiac pressures during BVP compared to those in private practice.

This study offers valuable insights into the preferences and practices of veterinary cardiologists regarding common interventional cardiac procedures and the significance of factors such as caseload volume and workplace environment in the decision‐making processes. The clinical significance of these differences and their effect on patient outcome remains to be assessed.

**Disclosures**

**Source of Funding**

**ECVIM CSF Funding**

No.

**ESVC**—**European Society of Veterinary Cardiology**

## ESVC‐O‐21

## Validation of a physical examination algorithm in dyspnoeic cats presenting to general practice: The RAPID CAT 2 study

### D. Dickson^1^, B. Ingram^2^, A. Cauvin^3^, L.I. Vatne^4,5^, E. Pavelková ^6^, J. Harris^7^, L. Ferasin^8^, C. Little^9^, M. Rishniw^10^

#### ^1^HeartVets, Porthcawl, United Kingdom; ^2^Alfreton Park Veterinary Hospital, Alfreton, United Kingdom; ^3^Cauvin Veterinary Consultancy Ltd, Essex, United Kingdom; ^4^Norwegian University of Life Sciences, Oslo, Norway; ^5^Empet Nydalen Small Animal Hospital, Oslo, Norway; ^6^Woodcroft Veterinary Hospital, Cheadle, United Kingdom; ^7^HeartVets, Exeter, United Kingdom; ^8^Cardiospecialist.co.uk, Hampshire, United Kingdom; ^9^Barton Veterinary Hospital, Canterbury, United Kingdom; ^10^Cornell University, Ithaca, United States

A previous study examining dyspnoeic cats presenting to general practice in the United Kingdom found that a simple diagnostic algorithm based on the presence of a gallop sound, rectal temperature, heart rate, and respiratory rate, could differentiate cardiac and non‐cardiac causes with good accuracy. We sought to validate this algorithm on a larger, naïve dataset.

We prospectively recruited cats from first‐opinion practice in the United Kingdom, Norway, Ireland and Italy. We enrolled cats with acute dyspnoea and recorded key historical and physical examination findings. First‐opinion practitioners managed these cats, with supervision from vets with advanced qualifications in cardiology, medicine or diagnostic imaging (certificate or boarded Diplomate). Cats were classified by a boarded Diplomate as having either a cardiac or non‐cardiac (respiratory, neoplastic, traumatic, or miscellaneous) cause for their dyspnoea. When possible, a final diagnosis with supporting clinical evidence was recorded. Statistical analyses comprised diagnostic test performance analysis and univariable and multivariable logistic regression, after cases were dichotomised into cardiac and non‐cardiac causes.

We enrolled 185 cats between December 2017 and February 2022 with a diagnostic classification reached for all cats: 57% cardiac (congestive heart failure, CHF), 23% respiratory, 13.5% neoplastic, 3% traumatic and 3.5% miscellaneous. 64% were male, mean age was 10 years old (ranged from 6 months to 19 years). Using the diagnostic algorithm of rectal temperature >40°C = non‐cardiac, then gallop sound or rectal temperature <37.5°C or heart rate >200bpm or respiratory rate >80bpm = cardiac (CHF); test accuracy was 76% with a positive predictive value of 77% and negative predictive value of 75%.

Multi‐variable logistic regression identified temperature and heart rate as independently associated with a diagnosis of CHF with a ROC AUC of 79.3%.

These data, obtained prospectively from multiple European locations, validate the original algorithm for differentiating cardiac and non‐cardiac dyspnoea in cats in general practice. Results are very similar to those obtained in the first study, lending veracity to the algorithm, and allow for further refinement of the system.

In conclusion, dyspnoeic cats presenting in practice with hypothermia, tachycardia, gallop sounds or extreme tachypnoea are more likely to have a cardiac cause underlying their dyspnoea and should be managed accordingly. Rapid identification of a cardiac cause for dyspnoea in cats facilitates effective management and benefits their welfare.

**Disclosures**

Honoraria for all authors from various pharma companies including Boeringher Ingelheim, CEVA, Vetoquinol, IDEXX. No honoraria are relevant to this study.

**Source of Funding**

Funding for the study was received from Pet Savers (a BSAVA charity); IDEXX and CVS Vets.

**ECVIM CSF Funding**

No.

**ESVC**—**European Society of Veterinary Cardiology**

## ESVC‐O‐22

## Rivaroxaban versus enoxaparin plus clopidogrel therapy in cats with thromboembolic disease

### S. Petchdee^1^, C. Panprom^2^

#### ^1^Faculty of Veterinary Medicine, Kasetsart University Faculty of Veterinary Medicine, Kasetsart University, Bangkok, Thailand; ^2^Department of Livestock Development, Chainat, Thailand

Thromboembolism (TE) is a common complication of feline hypertrophic cardiomyopathy, leading to severe clinical symptoms. This study compared the effects and identified the adverse impact of rivaroxaban alone or enoxaparin combined with clopidogrel on cats. Eighteen cats were enrolled in this study, seven cats were prescribed oral rivaroxaban (2.5 mg, q24h). Eleven cats received subcutaneous injections of enoxaparin (1 mg/kg, q24h) plus oral clopidogrel (3 mg/kg, PO q24h) for 2 months. Cats were treated for at least one of the following events: abnormal movement of the anterior mitral leaflet during systoles, enlargement of the left atrium, spontaneous echocardiographic contrast, or the presence of arterial thromboembolism. Complications of renal insufficiency and bleeding were observed. The cats were evaluated for plasma concentrations of D‐dimer, prothrombin time (PT), partial thromboplastin time (PTT), and international normalized ratio (INR). The relationship between echocardiography parameters and coagulation effects was analyzed. Blood samples from all cats were evaluated at baseline and 1‐ and 2 months post‐treatment. Rivaroxaban alone and cotreatment with enoxaparin and clopidogrel significantly affected PT and INR. No bleeding or recurrence of thrombus formation was investigated in cats treated with rivaroxaban. These data support the use of rivaroxaban therapy for hypertrophic cardiomyopathy‐associated thromboembolism in cats. Treatment with rivaroxaban alone for thromboembolism in cats demonstrated clinical effectiveness and no clinical complications.

**Disclosures**

**Source of Funding**

Faculty of Veterinary Medicine, Kasetsart University.

**ECVIM CSF Funding**

No.

**ESVC**—**European Society of Veterinary Cardiology**

## ESVC‐O‐23

## Observational longitudinal study of pulmonary hypertension in West Highland white terriers affected with canine idiopathic pulmonary fibrosis

### E. Roels^1^, A. Fastrès^2^, E. Rizzoli^2^, K. Mc Entee^3^, C. Clercx^2^, A.C. Merveille^2^

#### ^1^University of Liege University of Liege, Liege, Belgium; ^2^University of Liege Department of Small Animal Clinical Sciences, Liege, Belgium; ^3^University Libre of Bruxelles Laboratory of Physiology and Pharmacology, Bruxelles, Belgium

Canine idiopathic pulmonary fibrosis (CIPF) is a progressive parenchymal lung disease affecting aging West Highland white terriers (WHWTs). Pulmonary hypertension (PH) is a known co‐morbidity in WHWTs affected with CIPF necessitating close monitoring.

The aim of this descriptive study was to assess echocardiographic variations of PH parameters and the effect of treatment with sildenafil in WHWTs affected with CIPF.

WHWTs with compatible imaging and/or histopathological features of CIPF that had an echocardiography performed at diagnosis and a minimum of one echocardiographic follow‐up were retrospectively selected over an 11‐year period (2013–2024) (*n* = 28, median age 11.0 years, range 7.4–15.3). Rechecks were conducted approximately every 6 months until death or loss of follow‐up. Echocardiographic data were collected for a maximum of 4 follow‐ups for the purpose of this study. Right heart parameters were measured off‐line. The probability of PH was defined as low, intermediate, or high according to the 2020 ACVIM consensus guidelines. Linear mixed models were used to investigate the effects of sildenafil treatment, time, and their interaction with echocardiographic parameters. Statistical significance was set at *P* < 0.05.

At baseline, 11 (39.3%), 8 (28.6%), and 9 (32.1%) WHWTs affected with CIPF had low, intermediate, or high probability respectively, for PH, with none receiving sildenafil therapy. Median follow‐up time was 21.6 months (range 14.7‐35.0). At the first recheck, the probability of PH increased in 9 (31.2%), decreased in 3 (10.7%), and remained unchanged in 16 (57.1%) WHWTs, with 13 (46.4%) receiving sildenafil treatment for approximately 6 months. Sildenafil treatment significantly reduced main pulmonary artery to aorta ratio (MPA/Ao) with an estimated decrease of 0.06 units (95% CI: 0.01–0.11) in treated dogs (*P*=0.027). Time was associated with an enlargement in minimum right pulmonary artery diameter (RPADmin) and right ventricular internal dimension in diastole (RVIDd), with an estimated increase of 0.35mm (95% CI:0.09–0.62, *P*=0.013) and 0.07cm (95% CI: 0.02–0.11, *P*=0.004) respectively, every 6 months. Significant interactions between treatment and time were found for MPA/Ao (Est.:0.02, 95% CI:0–0.04, *P*=0.023), RVID_Ao (Est.: 0.04, 95% CI: 0.0–‐0.07, *P*=0.015) and right ventricle fractional area change (FAC) (Est.:−2.57, 95% CI: −4.81, −0.33, *P*=0.041), suggesting a moderating effect of sildenafil on the impact of time for these parameters. No other significant effects of treatment or time were noted on echocardiographic measurements. Over the study period, 24 WHWTs died including 17 from cardio‐respiratory causes.

In conclusion, this study highlights the importance of regular echocardiographic follow‐ups over the course of CIPF and suggests that sildenafil may mitigate the progression of specific echocardiographic parameters related to PH.

**Disclosures**

**Source of Funding**

**ECVIM CSF Funding**

No.

**ESVC**—**European Society of Veterinary Cardiology**

## ESVC‐O‐24

## Acute and chronic pulmonary thromboembolism in dogs: a prospective clinical study

### M. Lekane^1^, A.C. Merveille^1^, K. Gommeren^1^, M.M. Garigliany^2^

#### ^1^University of Liege Department of Small Animal Veterinary Clinical Sciences, Liege, Belgium; ^2^University of Liege Department of Morphology and Pathology, Liege, Belgium

Pulmonary thromboembolism (PTE) in dogs lacks an evidence‐based diagnosis. In humans acute and chronic PTE is diagnosed based on clinical probability, D‐dimers and thoracic imaging. This study describes history, clinical, laboratory and echocardiographic findings in dogs with necropsy‐confirmed PTE. Thoracic imaging findings are discussed in a separate study.

Dogs with clinical suspicion of PTE were prospectively included from 2019 until 2023. Physical examination, echocardiography, D‐dimers, cardiac troponin I (cTNi), and viscoelastography were performed. Only dogs with confirmed PTE on necropsy after fatal outcome remained included. Data are expressed as median and range. Acute PTE was histopathologically characterized by few organized thrombi in pulmonary arteries and secondary surrounding acute lung lesions, whereas chronic PTE displayed chronic, fibrotic, organized thrombi within the pulmonary vasculature.

Twenty‐four dogs were included, of which 9 were excluded (8 unconfirmed necropsy; 1 still alive). Ten had a histopathological diagnosis of acute PTE, 4 acute and chronic, 1 chronic. Chihuahuas (3) and Shih Tzus (3) were most commonly represented, median age and weight were 12 years [7–16] and 8.8 kg [2.60–31.4]. Eight had predisposing factors identified: hyperadrenocorticism (3), and severe pulmonic stenosis, pulmonary carcinoma, splenectomized hemangiosarcoma, protein losing enteropathy receiving prednisolone and acute kidney injury in individual dogs.

All dogs presented lethargy and dyspnea/tachypnea. Forty‐three percent demonstrated low cardiac output (syncope and/or systemic blood pressure ≤90 mmHg). All dogs had increased D‐dimers (2273 μg/L [388‐>6000]), whilst cTNi concentrations were increased in 7/10 dogs (443 ng/L [273.70–3542]), yet normal in 3/10 (24,8 ng/L [11.7–49]). Viscoelastography indicated hypercoagulability (5/8), hypocoagulability (3/8) and hyperfibrinolysis in a dog with hypercoagulability. Echocardiographic data were available for 13, of which 1 dog with pulmonary stenosis was excluded. Severe (6/12), moderate (4/12), mild (1/12) and absence of pulmonary hypertension (1/12) were identified based on pressure gradient and indirect echocardiographic changes. Right ventricular free wall appeared thicker and right ventricular normalized systolic area appeared enlarged in chronic PTE, yet these values did not differ significantly from acute PTE cases. Eleven dogs died during the initial PTE episode and 4 dogs died later with a median survival time of 62.5 days [12‐279] (other causes (3) or PTE relapse (1)).

This study demonstrates two coexisting forms of PTE, which appear to be associated with a high incidence of fatality. In this population, D‐Dimers were uniformly elevated whereas a majority of dogs demonstrated increased cTNi concentrations, abnormalities on viscoelastography and moderate to severe pulmonary hypertension on echocardiography.

**Disclosures**

**Source of Funding**

**ECVIM CSF Funding**

No.

**ESVC**—**European Society of Veterinary Cardiology**

## ESVC‐O‐25

## Effects of adjunct ambrisentan therapy on clinical signs, oxygenation capacity, and echocardiographic variables in dogs with pulmonary hypertension resistant to sildenafil therapy

### S. Goya^1^, N. Kusafuka^2^, H. Tani^1^, M. Ozawa^1^, T. Nakayama^1^

#### ^1^Nihon University Laboratory of Veterinary Radiology, Department of Veterinary Medicine, Fujisawa, Japan; ^2^Nihon University Nihon University Animal Medical Center, Fujisawa, Japan

Pulmonary hypertension (PH) in dogs is a highly fatal cardiovascular abnormality for which sildenafil, a phosphodiesterase‐5 inhibitor, is commonly used. However, dogs with severe PH may become resistant to sildenafil therapy and clinical signs deteriorate progressively. Ambrisentan is an endothelin receptor antagonist that dilates pulmonary vessels through a different mechanism from sildenafil. Human PH study demonstrated combined ambrisentan and phosphodiesterase‐5 inhibitors therapy is more effective than each monotherapy. This study aimed to prospectively evaluate the effects of adjunct ambrisentan therapy in dogs with severe PH resistant to sildenafil therapy.

We enrolled dogs with severe PH that exhibited syncope, dyspnea, and ascites despite receiving ≥4 mg/kg/day of sildenafil. Clinical signs were evaluated based on the FETCH and PH clinical signs scores. Conventional echocardiographic variables, arterial oxygen partial pressure, and plasma big endothelin‐1 concentrations were measured. Dogs received ambrisentan 0.1 mg/kg orally every 24 h (ambrisentan group) or 1.5–2.0 times the amount of sildenafil they are already receiving (control group). Dogs were evaluated before administration (baseline; BL) and at 2 (time 1) and 4 weeks (time 2) after administration of each drug. The BL values were compared with the values at times 1 and 2 in each group.

Nine dogs were included in the ambrisentan group, and eight dogs survived until time 2. Ten dogs were included in the control group, and six dogs survived until time 2. No significant changes were observed in all variables in the control group. The eight dogs (ACVIM classification group 1a; 6, group 3c; 2) in the ambrisentan group were 14.5 years (9–16 years), 3.7 kg (2.0–6.4 kg), with sildenafil dose of 6.7 mg/kg/day (6.0–8.7 mg/kg/day). FETCH and PH clinical signs scores significantly improved at times 1 (30±18, *p* = 0.005; 15±4, *p* = 0.002) and 2 (30±17, *p* = 0.005; 15±4, *p* = 0.003) compared with BL (50±20, 22±6). The main pulmonary artery/aorta diameter ratio measured by echocardiography significantly decreased at times 1 (1.31±0.35, *p* = 0.01) and 2 (1.33±0.28, *p* = 0.03) compared with BL (1.46±0.31). Arterial oxygen partial pressure significantly increased at times 1 (58.3±12.2 mmHg, *p* = 0.02) and 2 (58.4±14.4 mmHg, *p* = 0.02) compared with BL (49.3±12.9 mmHg). Plasma big endothelin‐1 concentrations significantly decreased at time 2 (21.7±7.2 pmol/L, *p* = 0.009) compared with BL (32.8±11.7 pmol/L, reference range; 1.1±0.5 pmol/L).

This study showed that adjunct ambrisentan therapy in dogs with severe PH refractory to sildenafil therapy improved clinical signs and oxygenation capacity by attenuating pulmonary vasoconstriction via the endothelin pathway.

**Disclosures**

**Source of Funding**

This work was supported by JSPS Grant‐in‐Aid for Young Scientists (No.23K14095).

**ECVIM CSF Funding**

No.

**ESVC**—**European Society of Veterinary Cardiology**

## ESVC‐O‐26

## Subcutaneous furosemide therapy for chronic management of refractory congestive heart failure in dogs and cats

### S. Lombardo^1,2^, H. Ferasin^1,3^, L. Ferasin^1,2^

#### ^1^The Ralph Referral Centre Cardiology, Marlow, United Kingdom; ^2^Specialist Veterinary Cardiology Consultancy Ltd Cardiology, Medstead, United Kingdom; ^3^Specialist Veterinary Cardiology Consultancy Ltd Cardiology, Medstead, United Kingdom

Loop diuretics are commonly used in veterinary cardiology due to their indisputable efficacy in relieving signs of congestion in heart failure (CHF). The diuretic dose requires regular adjustments over time to effectively control congestion. The presence of gastrointestinal oedema can reduce enteral absorption of oral diuretics in patients with active CHF (diuretic resistance). Therefore, subcutaneous administration of furosemide can be considered to overcome the reduced drug absorption. To date, there are only anecdotal reports of the utility of subcutaneous administration of furosemide in pets with resistance to oral furosemide. Therefore, the aim of this study was to determine the practicality of subcutaneous furosemide administration and its effectiveness in controlling signs of CHF in dogs and cats. For this purpose, we hypothesised that diuretic efficacy in patients with chronic CHF and unsatisfactory response to oral diuretics can be improved using subcutaneous furosemide injections. Clinical records of privately‐owned pets in CHF (9 dogs and 13 cats) with unsatisfactory response to oral diuretics were reviewed retrospectively. In all these cases, subcutaneous administration of furosemide was offered to pet owners following their request to consider euthanasia due to unsatisfactory clinical response characterised by persistent breathing effort and elevated sleeping respiratory rate (SRR > 50 breaths per minute‐bpm) at home, despite several therapeutic adjustments (refractory CHF). The mean age of dogs and cats was 8.78±1.92 and 5.22±4.02 years respectively. Average body weight was 20.5±16.1 Kg (dogs) and 4.5±1.0 Kg (cats). All dogs in CHF were receiving furosemide (6.26±2.0 mg/Kg/day), except for a single dog receiving torasemide (0.4 mg/Kg/day). Ten cats were on furosemide (5.45±1.6 mg/Kg/day) and three cats were on torasemide (0.25 [0.14‐0.44] mg/Kg/day). Oral diuretic therapy was substituted with subcutaneous furosemide injection at a dose of 6.4±2.0 mg/Kg/day in dogs and 5.0 [4.0‐7.5] mg/Kg/day in cats, which provided a stable clinical response (SRR <30 bpm) in all cases. Subcutaneous injections were well tolerated in most patients, with the only exception of two cats and one dog that experienced a mild and transitory skin reaction at the site of injection between the scapulae. Median survival time from the therapeutic modification to the endpoint (cardiac death or euthanasia) was 82 days (95% CI 6 to 171) in dogs and 90 days (95% CI 35‐749) in cats. In conclusion, subcutaneous furosemide administration appears to be a feasible therapeutic option for providing efficacious palliative care at home in dogs and cats with unsatisfactory response to oral diuresis.

**Disclosures**

**Source of Funding**

**ECVIM CSF Funding**

No.

**ESVC**—**European Society of Veterinary Cardiology**

## ESVC‐O‐27

## Management and short‐term outcome of 74 cases of acute congestive heart failure in cats (2017–2022)

### L. Murphy^1^, R. Stepien^2^, S. Tjostheim^1^, H. Kellihan^1^

#### ^1^University of Wisconsin School of Veterinary Medicine Cardiology, Madison, United States; ^2^University of Wisconsin School of Veterinary Medicine, Madison, United States

Congestive heart failure (CHF) is a common development in cats with cardiomyopathies. Optimal therapy for cats with acute CHF remains unknown but oxygen supplementation and loop diuretic therapy are frequently recommended. Pimobendan (PB) is a calcium sensitizing agent which has positive inotropic, lusitropic and vasodilating effects. Reports to date of use of PB in cats with CHF have generally revealed few side effects. Dobutamine (DB) is a β1 receptor agonist with similar positive lusitropic and inotropic effects.

The goals of this retrospective study were to describe the short‐term clinical response to CHF therapy in cats treated with furosemide and PB (standard therapy [ST] group), compared to cats treated with furosemide, PB and DB (DB group).

Sixty‐eight cats were included; the majority of cats were diagnosed with cardiomyopathic disease. Pulmonary edema score (PES) was assessed in all cats at admission and 24 hours later using a modified Murray Lung Injury Score.

Doses of furosemide and PB did not differ between treatment groups. The PES was lower on day 2 versus day 1 in both groups. The proportion of cats in each treatment group that showed improvement in their score did not differ (ST: 46/55, 84%, DB 12/16, 75%, *p* = 0.4710). Cats with a smaller change in PES score from Day 1 to Day 2 were less likely to survive to discharge (odds ratio 6.3 [95% CI 1.8 to 20.1], *p* = 0.0073). There was a significant increase in creatinine in both treatment groups (ST: *p* = 0.006, DB: *p* = 0.0037) on Day 2 versus Day 1.

Eight DB cats had adverse clinical findings attributed to DB administration noted in their records (arrhythmia: *N* = 4, neurologic signs: *N* = 4). The type of adverse sign (neurologic or arrhythmia) did not appear to be related to dose, but numbers were too small to analyze statistically. Overall survival of both groups was 76%, and there was no significant difference in survival to discharge between treatment groups. Seventy‐five percent of cats discharged alive were hospitalized for 3 days or fewer, and days of hospitalization did not differ between treatment groups.

The short‐term prognosis for cats treated for acute CHF in either treatment group was good, with most showing significant clinical and radiographic improvement in the first 24 hours of therapy. The results of this study may be useful in clinical prognostication and treatment planning for feline patients admitted with acute CHF. Further prospective study evaluating DB use in cats with CHF is warranted.

**Disclosures**

NA.

**Source of Funding**

NA.

**ECVIM CSF Funding**

No.

**SCH**—**Society of Comparative Hepatology**

## SCH‐O‐1

## Altered serum amino acids profiles in dogs with liver disease

### R.K. Phillips^1^, A. Blake^1^, A. Tinoco‐Najera ^1^, F. Dröes^1^, J. Suchodolski^1^, J. Steiner^1^, J. Lidbury^1^

#### ^1^Texas A&M University Gastrointestinal Laboratory, College Station, TX, United States

Alterations in serum amino acids (AA) profiles have been reported in different hepatopathies in people. However, studies investigating changes in AA profiles in dogs with liver disease remain limited. This cross‐sectional study aimed to compare differences in serum AA profiles in dogs with different liver disease types and with different degrees of histopathological abnormalities.

Client‐owned dogs with a definitive diagnosis of liver disease, confirmed by histology or diagnostic imaging, were prospectively enrolled between May 2019 and November 2021. Healthy control dogs (HC, *n* = 21) owned by hospital personnel were enrolled throughout 2021. Serum from HC and from dogs with chronic hepatitis (CH; *n* = 27), steroid hepatopathy (SH; *n* = 7), or congenital portosystemic shunt (CPSS; *n* = 8) were profiled using an amino acid analyzer (Biochrom 30+) for 31 nitrogenous compounds. Liver biopsies were collected (when available), processed for routine histology, scored for necroinflammatory activity, fibrosis, and copper accumulation, and submitted for tissue copper quantification. AA concentrations were compared by Kruskal‐Wallis tests followed by Dunn's multiple comparisons tests, as appropriate. The Benjamini‐Hochberg procedure was used to control the false discovery rate.

Compared with HC, dogs with CH had significantly increased ammonia (*p* = 0.0004), aspartic acid (*p* < 0.0001), glutamic acid (*p* < 0.0001), ornithine (*p* < 0.0001), phenylalanine (*p* = 0.0020), and total aromatic amino acids (AAA; *p* = 0.0272). Dogs with SH had significantly increased aspartic acid (*p* = 0.0164), glutamic acid (*p* = 0.0006), and ornithine (*p* = 0.0091). Dogs with CPSS had significantly increased ammonia (*p* < 0.0001), ornithine (*p* = 0.0170), phenylalanine (*p* < 0.0001), phosphoserine (*p* = 0.0137), serine (*p* = 0.0007), tyrosine (*p* = 0.0006), and total AAA (*p* < 0.0001), but significantly decreased carnosine (*p* = 0.0004), threonine (*p* = 0.0041), urea (*p* = 0.0012), and ratios of branched‐chain amino acids (BCAA) to AAA (*p* < 0.0001) and of BCAA to tyrosine (*p* = 0.0004). Dogs with higher necroinflammatory activity and fibrosis scores tended to have more severe AA abnormalities than dogs with lower scores; however, with the exception of glycine (*p* = 0.0006) and histidine (*p* = 0.0290), these differences did not reach the level of statistical significance (*p* > 0.05).

Among dogs with different liver diseases, distinctive serum AA concentration profiles as compared with HC were observed. The diagnostic and prognostic value of serum amino acids profiles in dogs with liver disease warrants further evaluation.

**Disclosures**

**Source of Funding**

**ECVIM CSF Funding**

No.

**SCH**—**Society of Comparative Hepatology**

## SCH‐O‐2

## Effectiveness and associated adverse effects of telmisartan in the treatment of portal hypertension secondary to liver disease in dogs

### N. Lopez‐gomez ^1^, I. Mesa^1^, Y. Fernandez^2^, C. Martinez^1^

#### ^1^AUNA Especialidades Veterinarias, Valencia, Spain; ^2^Anicura Imavet Referencia Veterinaria, La Coruña, Spain

Portal hypertension (PH) originates from an increase in resistance, in blood flow, or both in portal circulation, leading to complications such as ascites, acquired shunts (AS), and hepatic encephalopathy. The renin‐angiotensin‐aldosterone system is an important regulator of portal pressure. In human medicine, telmisartan ameliorates the cirrhosis‐induced PH by reducing liver fibrosis, inflammation, angiogenesis, and vascular remodelling. The aim of this study was to evaluate the effectiveness and adverse effects of telmisartan in the treatment of PH secondary to liver disease in dogs. Retrospectively (January 2020–February 2023), data from 12 dogs was analysed, included history, clinical signs, clinicopathological and imaging findings, histopathological diagnosis, telmisartan dosage, response to treatment and adverse effects. The degree of response to telmisartan relative to the severity of PH was classified as complete response (absence of ascites), partial response (decrease compared to the initial examination), or lack of response.

The most prevalent clinical signs at the time of diagnosis were abdominal distension (8/12 (67%)), lethargy (6/12 (50%)), diarrhoea and vomiting (5/12 (42%)) and hepatic encephalopathy (4/12 (33%)). Relevant laboratory findings included mild anaemia (7/11 (64%)) and mild elevation of ALKP and ALT activity (9/11 (82%)). Common ultrasound findings were ascites (12/12 (100%)), microhepatia (9/12 (75%)) and AS (9/12 (75%)). A definitive diagnosis was achieved in 9/12 (75%) cases, namely ductal plate malformation (3/9 (33%)), dissecting lobular hepatitis (2/9 (22%)), hypoplasia of the portal vein (2/9 (22%)), nodular hyperplasia (1/9 (11%)) and complication in congenital portosystemic shunt attenuation (1/9 (11%)). The mean initial dose of telmisartan was 0.78 mg/kg/24h (range 0.25‐1.2). With regards to the presence of ascites, the response rate was classified as complete in 7/12 dogs (58%), partial in 3/12 (25%), and no response in 2/12 (17%). Five dogs (42%) developed adverse effects (azotaemia 3/12 (25%), hypotension 2/12 (17%), vomiting 2/12 (17%) and hyperkalemia 1/12 (8%)). In all dogs with adverse effects, relapse of ascites recurrence documented after discontinuation of telmisartan. Subsequent reintroduction at lower doses led to variable responses regarding ascites. Median survival time was 181 days (range 26–1439), with 6 animals alive at present.

While further studies are needed to determine the effectiveness and safety of telmisartan in dogs with PH secondary to liver disease, these results support its use to reduce portal pressure and the degree of ascites. Due to the high rate of potential adverse effects, dose adjustement and close monitoring the animal is recommended, particularly blood pressure, renal function, and electrolytes.

**Disclosures**

**Source of Funding**

**ECVIM CSF Funding**

No.

**SCH**—**Society of Comparative Hepatology**

## SCH‐O‐3

## Intact thrombin generation potential in dogs with different liver diseases

### F. Dröes^1^, J. Adelmeijer^2^, J. Cavasin^1^, K. Phillips^1^, A. Tinoco Najera^1^, P. Manino^3^, J. Steiner^1^, C. Rutter^4^, T. Lisman^5^, J. Lidbury^1^

#### ^1^Gastrointestinal Laboratory, Texas A&M University Veterinary Small Animal Clinical Sciences, College Station, TX, United States; ^2^Surgical Research Lab, University of Groningen, UMCG, Department of Surgery, University Medical Center, Groningen, Netherlands; ^3^GulfCoast Veterinary Specialists, Houston, United States; ^4^Emergency and Critical Care Service, Texas A&M University Small Animal Veterinary Teaching Hospital, College Station, United States; ^5^Surgical Research Lab, University of Groningen, UMCG, Department of Surgery, University Medical Center Groningen, Groningen, Netherlands

The liver plays an important role in hemostasis as the majority of coagulation proteins are synthesized there. Hemostatic changes therefore are common in patients with liver disease. In humans, the simultaneous decline in pro‐ and anti‐hemostatic proteins results in a ‘rebalanced’ hemostatic system, however the hemostatic status in dogs with liver disease is understudied. The aim of this study was to assess thrombin generation potential in dogs with different liver diseases and with different hemostatic classifications as determined by viscoelastometry.

Dogs with liver disease (30 congenital portosystemic shunts [CPSS], 19 acute liver disease [ALD], 4 reactive hepatitis [RHep], 11 vacuolar hepatopathy [VacH], 22 chronic hepatitis [CH], 17 hepatic neoplasia [HNeo]), and 16 healthy control dogs were enrolled. A traditional coagulation panel and also a point‐of‐care viscoelastic coagulometric test (VCM Vet™, VCM) were evaluated at the time of enrolment. Citrated plasma was concurrently collected, stored at ‐80°C, and later evaluated with an automated thrombin generation assay (Calibrated Automated Thrombogram, CAT) with addition of thrombomodulin. Hemostasis was classified based on results of VCM as hypercoagulable, normocoagulable, or hypocoagulable. Results of CAT were compared between healthy controls and dogs with different liver diseases as well as across different hemostatic classifications using a Kruskal‐Wallis test with Dunn's correction for multiple comparisons.

Overall, 16 dogs were classified as being hypercoagulable, 78 as normocoagulable, and 21 as hypocoagulable. CAT‐derived endogenous thrombin potential (ETP) was not significantly different from healthy controls across liver disease groups: healthy controls, median: 88.5 nM thrombin * min (interquartile range [IQR]: 57.0–101.8); CPSS, 112.5 (86.8–147.8); ALD, 66.0 (34.0–85.0); RHep, 56.5 (45.5–105.0); VacH 54.0 (30.0–89.0); CH 72.5 (60.0–89.0); Neo 73.0 (46.0–86.5); *p* > 0.25. ETP was not significantly different across hemostatic classifications: hypercoagulable, median: 72.0 nM thrombin * min (IQR: 42.0–82.8); normocoagulable, 86.0 (59.8–86.0); hypocoagulable, 84.0 (45.0–140.0); >*p* > 0.1.

In dogs with different liver diseases and different hemostatic classifications, thrombin generating capacity seems to be maintained, possibly supporting the concept of rebalanced hemostasis in dogs with liver disease. These findings highlight the need for more research into the role of pro‐ and anti‐coagulant factors and their effect on hemostatic balance in dogs with liver disease.

**Disclosures**

• Entegrion (producer of VCM Vet™ cartridges) has supported this research by providing VCM Vet™ cartridges for this study. • At the time of this study: Drs. Dröes, Cavasin, Najera, Steiner, and Lidbury and Mr Phillips are employed by the Gastrointestinal Laboratory at Texas A&M University, which offers laboratory testing, including hepatic histopathology services, on a fee‐for‐service basis.

**Source of Funding**

‐ Cartridges for VCM Vet™ were provided by Entegrion.—A large part of the research was funded by the Gastrointestinal Lab at Texas A&M University.—Accommodation and travel to perform part of the work of this study at the University Medical Center Groningen was supported the Office of Graduate and Professional Studies (Texas A&M University—School of Veterinary Medicina & Biomedical Sciences)

**ECVIM CSF Funding**

No.

**SCH**—**Society of Comparative Hepatology**

## SCH‐O‐4

## Fecal bile acids in canine chronic liver disease: Results in 46 dogs

### V. Habermaass^1^, F. Bartoli^2^, E. Gori^1^, R. Dini^1^, A. Cogozzo^1^, V. Marchetti^1^

#### ^1^Department of Veterinary Sciences, University of Pisa, Pisa, Italy; ^2^Department of Translational Research Medicine, University of Pisa, Pisa, Italy

Through many pathogenetic mechanisms, both fecal and serum bile acids (BAs) concentrations are known to be altered in human patients with different kind of chronic liver diseases (CLDs), especially biliary disorders. Veterinary literature is currently mainly focused on alteration of fecal BAs during canine chronic enteropathy, but scarce literature is available regarding their potential modifications during CLDs. The present study aimed to evaluate fecal BAs pattern in dogs with CLDs according to different clinical and clinicopathological variables.

Dogs were prospectively recruited, and the diagnosis of chronic liver disease was based on the concurrent presence of persistent increased liver enzymes and ultrasound hepatobiliary alterations. Biliary tract disease (BTD) was identified in case of 2 or more between alkaline phosphatase >250 U/L, gamma‐glutamyl transferase > 11 U/L, total bilirubin > 0.3 mg/dL, cholesterol >280 mg/dL and signs of biliary tract disease at abdominal ultrasound. For each dog, accurate anamnesis, complete bloodwork and abdominal ultrasound were assessed. Canine feces were stored at ‐80°C, lyophilized, extracted, and subsequently analyzed by High Performance Liquid Chromatography technique. Cholic Acid (CA), Chenodeoxycholic Acid (KDCA), Ursodeoxycholic Acid (UDCA), Deoxycholic Acid (DCA), Lithocholic Acid (LCA), Deoxycholic Acid (DCA) were measured, Total Primary (CA+KDCA) and Secondary BAs (UDCA+DCA+LCA), Primary/Secondary BAs ratio were calculated.

Forty‐six dogs were enrolled. At the moment of inclusion, 26/46 (56%) were presenting gastrointestinal clinical signs. Total Primary BAs, CA, KDCA, P/S ratio and Total BAs were significatively increased in dogs presenting with BTD (*n* = 18) compared to dogs without significant biliary tract involvement (*n* = 28), respectively: 695 nmol/g (91–1823) vs. 289 (56–431), *p* < .0001; 407 nmol/g (27–1305) vs. 112(8‐349), *p* = .0003; 237 nmol/g(15‐861) vs. 100 (12–396), *p* = .003; .83(.06‐2.31) vs. .37(.07–.88), *p* = .0002; 1509 nmol/g (481–4824) vs. 1119 (361–3387), *p* = .005. Secondary BAs, UDCA, DCA, LCA did not differ significantly between dogs with and without BTD, however UDCA was tendentially lower in dogs with BTD, with 233 nmol/g(32‐854) vs. 262(64‐1734), *p* = .59.

The increase of fecal Primary BAs (CA, KDCA) and Total Bile acids in CLDs dogs with biliary tract involvement is consistent with the trend observed in human patients with biliary tract disorders. Since fecal BAs are dependent on the metabolic activity of the microbiota, this alteration could be mostly related to the role of Gut‐Liver axis and intestinal dysbiosis, also considering the high recurrence of gastrointestinal signs in this population.

**Disclosures**

**Source of Funding**

This research was funded by the University of Pisa (Italy).

**ECVIM CSF Funding**

No.

**SCH**—**Society of Comparative Hepatology**

## SCH‐O‐6

## Retrospective analysis of the incidence and clinicopathological findings associated with ammonium urate urolithiasis in dogs with congenital hepatic vascular anomalies; 378 cases (2010–2023)

### V. Travail^1^, M. Walton‐Clarke ^2^, C. Lubbers^1^, C. Motta^4^, P. Garcia Dominguez^5^, T. Chapman^6^, K. Peak^5^, L. Goonan^5^, K. Clarke^7^, N. Lau^7^, S. Keyte^8^, V. Coates^8^, V. Black^9^, S. Conway^9^, C. Stilwell^10^, F. Pilatti^10^, A. Holmes^11^, J. Scott^11^, C. Dye^12^, A. Davenport^12^, A. Kent^13^, C. Prior^14^, G. Ruiz^15^, G. McLauchlan^16^, T. Conley^16^, M. Rosell Garcia^17^, C. Good^17^, A. Eiras^18^, I. Bras^18^, F. Valls Sanchez^19^, M. Nicola^19^, A. Di Bella^3^

#### ^1^Southern Counties veterinary specialists Internal medicine, Ringwood, United Kingdom; ^2^Eastcott referral, Swindon, United Kingdom; ^3^Southern counties veterinary specialists Internal medicine, Ringwood, United Kingdom; ^4^Southern Counties Veterinary Specialists, Ringwood, United Kingdom; ^5^Anderson moores veterinary specialists, Winchester, United Kingdom; ^6^Southern Counties veterinary Specialists, Ringwood, United Kingdom; ^7^Davies Veterinary Specialists, Hitchin, United Kingdom; ^8^Lumbry Park Veterinary Specialists, Alton, United Kingdom; ^9^Langford Vets Small Animal Referral Hospital, Bristol, United Kingdom; ^10^The Ralph Veterinary Referral Centre, Marlow, United Kingdom; ^11^Paragon Veterinary Referrals, Wakefield, United Kingdom; ^12^Pride Veterinary Referrals, Derby, United Kingdom; ^13^Blaise Veterinary Referral Hospital, Longbridge, United Kingdom; ^14^Willows Veterinary Centre & Referral Service, Shirley, United Kingdom; ^15^Highcroft Veterinary Hospital—Whitchurch, Bristol, United Kingdom; ^16^AURA Veterinary, Guildford, United Kingdom; ^17^Wear Referrals Veterinary Specialist & Emergency Hospital, Bradbury, United Kingdom; ^18^Southfields Veterinary Specialists, Basildon, United Kingdom; ^19^Dick White Referrals, Cambridgeshire, United Kingdom

Urate urolithiasis is commonly seen in dogs with congenital hepatic vascular anomalies, with a reported prevalence of 14.3%–35.8%.

The objectives of this study were to retrospectively assess the incidence of ammonium urate urolithiasis in dogs with congenital hepatic vascular anomalies, and to investigate clinical variables associated with the presence of urate urolithiasis.

378 dogs diagnosed with congenital hepatic vascular anomalies (EHPSS, IHPSS, PVH) by CT angiography or liver biopsy in 17 referral hospitals between 2010 and 2023 were included.

Data including signalment, clinical signs, physical examination findings, and clinicopathologic test results were collected. Shunt morphology was determined from CT angiography, and the presence of ammonium urate urolithiasis was recorded. Ordinal/continuous data were summarised as median and range and compared between groups using Kruskal Wallis tests. Categorical data was summarised by frequencies and percentages and compared between groups using Fisher's Exact Tests. Nonnormally distributed data was reported as median, IQR, and range, and compared between groups using the Mann Whitney Test. Categorical significance was taken as *p* < 0.05.

The overall incidence of ammonium urate urolithiasis was 38.1%. Dogs with urolithiasis were older (45.7 months vs. 23.5 months; *p* < 0.001) and more likely to be male. While there was no significant difference in the overall incidence of urolithiasis among vascular anomalies, dogs with IHPSS were more likely to have ammonium urate crystalluria compared to those with EHPSS (87.1% vs. 42.7%; *p* < 0.001), and EHPSS dogs were more likely to have uroliths (71.8% vs. 19.4%; *p* < 0.001). Ammonia was the only biochemical variable that differed significantly between dogs with and without urolithiasis, being higher in those without urolithiasis (*p* = 0.007). Urine specific gravity was significantly higher in dogs with urolithiasis (1.027 vs. 1.020; *p* <0.001), and dogs with urolithiasis were more likely to have haematuria on dipstick analysis and sediment examination.

To conclude, no difference was found in the overall incidence of ammonium urate urolithiasis in dogs with IHPSS, EHPSS and PVH. However, dogs with IHPSS were significantly more likely to have ammonium urate crystalluria when compared to dogs with EHPSS, and those with EHPSS were more likely to have urinary calculi. Dogs with higher ammonia levels were less likely to have ammonium urate urolithiasis, and older dogs, males, or those with evidence of haematuria had an increased incidence of urolithiasis.

**Disclosures**

**Source of Funding**

**ECVIM CSF Funding**

No.

**SCH**—**Society of Comparative Hepatology**

## SCH‐O‐7

## Risk factors for short‐term mortality of hepatic lipidosis: A multicentre retrospective study of 162 cats

### M. Petibon^1^, T. Brignon^2^, C. Maurey^1^, T. Giampaolo^3^, J. Montier^4^, A. Tellier^5^, E. Krafft^3^, M. Mantelli^6^, L. Desquilbet^1,7^, G. Benchekroun^8,9^

#### ^1^Ecole Nationale Vétérinaire d'Alfort, CHUVA, Service de médecine interne 94700, Maisons‐Alfort, France; ^2^CHV ADVETIA 78140, Vélizy‐Villacoublay, France; ^3^Département des Animaux de Compagnie de Loisir et de Sport, VetAgro Sup, Marcy l'Etoile, France; ^4^Internal Medicine Department, Ecole Nationale Vétérinaire de Toulouse, Toulouse, France; ^5^Oniris, service de médecine interne 44300, Nantes, France; ^6^INTHERES, Université de Toulouse, INRAE, ENVT, Toulouse, France; ^7^Ecole nationale vétérinaire d'Alfort, Univ Paris Est Créteil, INSERM, IMRB 94700, Maisons‐Alfort, France; ^8^Ecole Nationale Vétérinaire d'Alfort, CHUVA, Service de médecine interne, Maisons‐Alfort, France; ^9^Ecole nationale vétérinaire d'Alfort, Univ Paris Est Créteil, INSERM, IMRB, Maisons‐Alfort, France

Feline hepatic lipidosis usually requires prolonged hospitalization. Data to accurately estimate the short‐term risk of death in cats with hepatic lipidosis are currently scarce.

The aim of the study was to determine at the time of diagnosis risk factors of death at 1‐month in cats diagnosed with hepatic lipidosis.

Cats diagnosed with hepatic lipidosis from 3 teaching hospitals between 2010 and 2023 were retrospectively included. Exposure variables were selected, based on the existing literature or their association with known complications of feline hepatic lipidosis.

To identify risk factors of death at 1‐month, univariate and multivariate logistic regressions using the stepwise method were performed.

Overall, 162 cats were included with 62 cats (37%) deceased at 1‐month. Median age was 7.7 years (range 1.00–18.80). Secondary hepatic lipidosis was diagnosed in 79 cats (49%), hepatobiliary diseases accounting for the most common underlying conditions (37/79, 46%).

NH3>52U/L (crude OR [cOR]=5.25 [1.18–23.22]_95%_), ALT<462U/L (cOR=4.16 [1.69–10.00]_95%_), depression (cOR=3.87 [1.46–10.23]_95%_), a BCS<2/9 (cOR=3.72 [1.32–10.52]_95%_), pH <7.35 (cOR=3.29 [1.10–9.83]_95%_), and temperature<37.8°C (cOR=2.48 [1.04–5.94]_95%_) were significantly associated with death at 1‐month. In multivariate analysis, death at 1‐month was significantly more frequent in cats with ALT<462U/L (adjusted OR [aOR]=4.48 [1.63–12.34]_95%_), depression (aOR=4.36 [1.28–14.77]_95%_), and secondary hepatic lipidosis (aOR=2.61 [1.18–5.77]_95%_).

These results indicate that presence of depression, an underlying disease and ALT<462U/L seem associated with short‐term mortality in cats with hepatic lipidosis. The higher odds of short‐term mortality associated with a decreased ALT activity had not been reported: the pathophysiological and clinical significance of this finding is yet to be determined.

**Disclosures**

**Source of Funding**

**ECVIM CSF Funding**

No.

**SCH – Society of Comparative Hepatology**

## SCH‐O‐8

## Complications associated with hepatic fine needle aspiration in cats; A retrospective study

### K. Fong^1^, S. Parton^2^, A. Boag^1^, J. Cartwright^1^

#### ^1^The Royal (Dick) School of Veterinary Studies, The University of Edinburgh The Department of Internal Medicine, Edinburgh, United Kingdom; ^2^The Royal (Dick) School of Veterinary Studies, The University of Edinburgh, Edinburgh, United Kingdom

Ultrasound‐guided fine needle aspiration biopsy (FNA) is a commonly performed first‐line diagnostic procedure when investigating liver disease. In humans, the safety of hepatic FNA is well‐documented. In dogs, retrospective studies report low complication rates; however, this has yet to be evaluated in a feline population. The primary aim of this study is to report complications in hepatic FNA in cats. The secondary objective is to assess the effects of hepatopathy and coagulopathy on complication rates.

Medical records (2018–2022) were retrospectively searched to identify cats presenting for hepatic FNA. Information on liver enzyme parameters, coagulation times, platelet counts, methods of restraint, and the presence of complications, during or within 24 h of the FNA procedure, were evaluated. Clinical hepatopathy at the time of FNA was designated if cases met two of the following six criteria (elevated alanine aminotransferase [ALT], alkaline phosphatase [ALP], gamma‐glutamyl transpeptidase [GGT], total bilirubin [tBil], bile acids [BA]), or abnormal hepatic diagnostic imaging findings) or if hepatopathy was listed as a differential on medical records.

The study population consisted of 229 cats from first opinion and referral services that presented on 269 separate occasions. Of these 269 events, hepatic FNA were completed in 262, and 241 had adequate peri‐procedural notes available. Complications were noted in 9/241 events (3.7%; 95CI: 1.7%–7%), with mild haemorrhage predominant. Most events did not require interventions (7/9) and no cats died. Clinical hepatopathy was present in 147/241 (61%; 95CI: 54%–67%) events and in most cases with complications (7/9); abnormal findings on hepatic diagnostic imaging were found in 8/9. Coagulation times were measured on 97 occasions and abnormal parameters were noted in 39 (40%; 95CI: 30%–50%). Thrombocytopaenia was recorded in 9 out of 228 events where platelet count was measured (3.9%; 95CI: 1.8%–7.4%). Coagulation parameters and platelet count were not associated with complications. No association between complications and other variables, including methods of restraint, adequacy of sedation or cytological diagnosis, were seen.

In our study population, hepatic FNA in cats were safe, and bleeding complications were uncommon. Clinical hepatopathy was frequently noted in the small number of cats that bled following liver FNA. Abnormal coagulation parameters and thrombocytopenia were also not associated with the complication rate, but the number of complications was low.

**Disclosures**

NA.

**Source of Funding**

None.

**ECVIM CSF Funding**

No.

**SCH**–**Society of Comparative Hepatology**

## SCH‐O‐9

## The effect of transdermal mirtazapine on the appetite recovery time in hospitalized cats with hepatobiliary disease: A prospective study

### S. Carvalho^3^, B. Mendoza^1,2^, P. Lunet Marques^1,4^, R. Leal^1,4^

#### ^1^Centre for Interdisciplinary Research in Animal Health, Fac.Vet.Med., ULisbon, Lisbon, Portugal; ^2^IVC Evidensia, Hospital Vet. Bom Jesus, Braga, Portugal; ^3^Vet Teaching Hospital, Fac. of Vet Med. ULisbon, Lisbon, Portugal; ^4^Associate Laboratory for Animal and Veterinary Sciences (AL4AnimalS), Lisbon, Portugal

Transdermal mirtazapine ointment is a commonly prescribed appetite stimulant. However, its effect on cats with hepatobiliary disease is scarcely documented. This study aimed to evaluate the effect of transdermal mirtazapine on the appetite recovery time in hospitalized cats with hepatobiliary disease, in which a feeding tube was placed.

A randomized controlled trial was conducted after previous evaluation and acceptance by the local Ethical Committee. Cats hospitalized with a confirmed or presumptive diagnosis of feline hepatobiliary disease (detailing cholangitis, cholangiohepatitis and/or idiopathic hepatic lipidosis) were eligible for the study after owner's consent. Cats were included if there were clinicopathological and ultrasound‐features compatible with hepatobiliary disease and had a nasal or esophagostomy feeding tube placed within the first 24h of hospitalization. Cats were excluded in case of any evidence of concurrent systemic diseases, confirmed hepatic neoplasia or previous history of appetite stimulants in the last four weeks. After placing the feeding tube, cats were randomized into one of two groups: G1 – cats treated with transdermal mirtazapine (0.1g ointment/cat, once a day starting in the first 12h after tube placement) and G2 –control group. Data concerning clinical signs, appetite recovery and length of hospitalization was analyzed. Cats of G1 were also monitored for potential side effects attributed to transdermal mirtazapine (vocalization, hyperactivity, or local erythema). Comparison between groups were conducted using nonparametric tests.

A total of thirteen cats were enrolled. All cats received standard medical care for the respective hepatobiliary disease. Seven cats were included in G1 and six cats in G2. Median anorexia time before hospital admission was similar between groups (3 and 4.5 days for G1 and G2, respectively, *p* = 0.72). No side effects were observed in cats receiving transdermal mirtazapine. Median days until spontaneous appetite recovery were 3 ± 7.25 and 11 ±4 days for G1 and G2, respectively (*p* = 0.4). Length of hospitalization was shorter for cats from G1 than G2 (3±1 days versus 12 ± 3 days; *p* = 0.057).

Since no adverse effects were identified, transdermal mirtazapine appears to be a safe appetite stimulant in hospitalized cats with hepatobiliary disease in which a feeding tube was placed. Although no significant differences were found, cats receiving mirtazapine tendentially showed a faster appetite recovery and shorter hospitalization compared to those not receiving it.

**Disclosures**

"This work was supported by FCT—Fundação para a Ciência e Tecnologia IP, grant UIDB/00276/2020 and by LA/P/0059/2020—AL4AnimalS”.

**Source of Funding**

"This work was supported by FCT—Fundação para a Ciência e Tecnologia IP, grant UIDB/00276/2020 and by LA/P/0059/2020—AL4AnimalS”.

**ECVIM CSF Funding**

No.

**SCH** – **Society of Comparative Hepatology**

## SCH‐O‐10

## Feline emphysematous cholecystitis, a descriptive study

### M. Hernandez Perello^1^, C. Prior^2^, M. Moloney^3^, C. Albuquerque^4^

#### ^1^Wear Referrals Small Animal Hospital, Bradbury, United Kingdom; ^2^Willows Veterinary Centre & Referral Service Willows Veterinary Centre & Referral Service, Solihull, United Kingdom; ^3^Lumbry Park Veterinary Specialists, Hampshire, United Kingdom; ^4^Hamilton Specialist Referrals, Buckinghamshire, United Kingdom

Emphysematous cholecystitis (EC) is a rare manifestation of acute cholecystitis complicated by gas‐producing organisms. There is no previous literature describing emphysematous cholecystitis in cats.

The aim of the study is to describe the clinical and pathological features of EC in cats.

The databases of all Diplomate‐led Small Animal Internal Medicine referral centres located in the United Kingdom and Ireland were searched. Five cases with confirmed feline EC were identified.

Three cats were Domestic Shorthair and two were Siamese. The age ranged from 6.5 to 13 years (median 8 years). The main clinical signs included vomiting (80%), hyporexia or anorexia (80%), lethargy (60%) and weight loss (40%). The main abnormalities on physical examination were jaundice (60%), abdominal discomfort and organomegaly (40%).

The most common laboratory abnormalities were lymphopenia, increased serum alanine transaminase (ALT) activity and hyperbilirubinemia.

Abdominal imaging including ultrasound or computed tomography showed dilated intra and extra‐hepatic bile ducts and intra‐luminal gas foci in all cases. Choledocoliths were identified in 2/5 cats, partially or completely obstructing the duodenal papilla. The gallbladder wall was thickened in 3/5 cases. A mild amount of peritoneal free fluid was present in 3/5 cases.

Aerobic and anaerobic bacterial culture of the bile was available in all cases. The culture identified a moderate growth of *Corynebacterium spp*. in one case and moderate growth of *Enterococcus spp*. in another case. One cat had rod‐shaped bacteria and cocci on bile cytology, however bacterial culture was negative. Choledocolith culture was available in 2 cats, with no bacterial growth. All cats had received antibiotics prior to these cultures.

The treatment of choice was surgery within 24 h of diagnosis in two cases, medical treatment including antibiotics and ursodeoxycholic acid for a number of weeks prior to surgery in two cases, and medical treatment alone in one case.

From the five cats diagnosed with EC, three survived long‐term. The cat treated medically was euthanised due to progression of clinical signs and one cat died from post‐operative complications.

This is the first time that EC has been described in a cohort of cats. EC warrants consideration as a differential diagnosis in cats presenting with vomiting, hyporexia, lethargy and increased serum ALT and bilirubin.

**Disclosures**

**Source of Funding**

**ECVIM CSF Funding**

No.

**ESVNU – European Society of Veterinary Nephrology and Urology**

## ESVNU‐O‐1

## Exploring the impact of kidney biopsy on survival in dogs with glomerular disease: Insights from the european veterinary renal pathology service (evrps)

### F. Rossi^1^, E. Zini^2,3^, A. Van Dongen^4^, S. Benali^5^, M. Massaro^6^, B. Contiero^7^, F. Dondi^8^, L. Aresu^9^

#### ^1^AniCura Istituto Veterinario di Novara Internal Medicine, Granozzo con Monticello (NO), Italy; ^2^Vetsuisse Faculty, University of Zurich Clinic for Small Animal Internal Medicine, Zurich, Switzerland; ^3^University of Padova Clinic for Small Animal Internal Medicine, Legnaro (PD), Italy; ^4^Faculty of Veterinary Medicine, Utrecht University Department of Clinical Sciences, Utrecht, Netherlands; ^5^Laboratorio di Analisi Veterinarie MYLAV, Milan, Italy; ^6^Laboratorio di Analisi Veterinarie MYLAV, Milan, Italy; ^7^University of Padova Department of Animal Medicine Production and Health, Legnaro (PD), Italy; ^8^Alma Mater Studiorum University of Bologna Department of Veterinary Medical Sciences, Ozzano dell'Emilia (BO), Italy; ^9^University of Turin Department of Veterinary Sciences, Grugliasco (TO), Italy

Kidney biopsy is not commonly performed in dogs with protein‐losing nephropathy due to the risk of bleeding and because requires anesthesia. Furthermore, its relevance to address prognosis has been poorly investigated. Hence, the European Veterinary Renal Pathology Service (https://www.evrps.net/) reviewed kidney biopsies of dogs with renal proteinuria to study survival.

Renal biopsies of dogs with protein‐losing nephropathy submitted between 2018 and 2023 were included if light microscopy (LM), transmission electron microscopy (TEM) and immunofluorescence (IF) were performed. In addition, dogs were enrolled if the veterinarian provided information about serum creatinine concentration and urine protein‐to‐creatinine ratio at the time of renal biopsy and during follow‐up. Survival analysis was studied with Kaplan–Meier followed by log‐rank tests and relative risk was calculated with contingency tables.

One‐hundred ten dogs were included. Immune‐complex glomerulonephritis (ICGN) was diagnosed in 61 dogs (56%), non‐ICGN in 39 (35%) and juvenile nephropathy in 10 (9%). Among ICGN, membranous glomerulonephropathy (MGN) was documented in 35 of 61 dogs (58%), membranoproliferative glomerulonephritis (MPGN) in 21 (34%), immune‐complex focal and segmental glomerulosclerosis in 3 (5%) and mesangioproliferative glomerulonephritis in 2 (3%). Among non‐ICGN, amyloidosis was observed in 16 of 39 dogs (41%), non‐immune‐complex focal and segmental glomerulosclerosis in 9 (23%), minimal change disease in 4 (10%), diffuse and global glomerular sclerosis in 3 (8%) and miscellaneous in 7 (18%). Survival of dogs that died due to renal disease was significantly longer for those with ICGN compared to non‐ICGN (mean±SEM: 777±56 vs. 674±88 days; *P*=0.017). Considering dogs with normal serum creatinine at renal biopsy, those with ICGN had lower risk to reach creatinine concentrations increased by 50% above normal compared to those with non‐ICGN (relative risk=0.58, 95% confidence interval=0.34–0.98; *P*=0.044). Among ICGN, survival did not differ for MGN and MPGN. Among non‐ICGN, those with amyloidosis had poorer outcome (mean±SEM: 384±100 vs. 887 ±111 days; *P*=0.003). Normalization of urine protein‐to‐creatinine ratio over time was not different between groups (ICGN 7/55=12.7%, non‐ICGN 10/39=25.6%; *P*=0.183).

In conclusion, dogs with renal proteinuria affected by ICGN may live longer compared to dogs with non‐ICGN due to less rapid progression of the underlying renal disease. Outcome of MGN and MPGN is comparable in dogs with ICGN, whereas in the non‐ICGN group amyloidosis carries the worst prognosis. Normalization of proteinuria over time does not seem different between groups. Kidney biopsy comprehensive of LM, TEM and IF is essential to characterize protein‐losing nephropathy, optimize treatment and refine prognosis in dogs.

**Disclosures**

**Source of Funding**

**ECVIM CSF Funding**

No.

**ESVNU** – **European Society of Veterinary Nephrology and Urology**

## ESVNU‐O‐2

## Expression of β thymosin peptides in cat kidneys and changes associated with chronic kidney disease

### C.Y. Man^1^, S. Spencer^1^, N. Lötter^1^, C. Brown^2^, R. Jepson^3^, J. Elliott^1^, E. Vasilopoulou^1^

#### ^1^Royal Veterinary College Department of Comparative Biomedical Sciences, London, United Kingdom; ^2^University of Georgia Athens Veterinary Diagnostic Laboratory, Athens, GA, United States; ^3^Royal Veterinary College Department of Clinical Science and Services, London, United Kingdom

Chronic kidney disease (CKD) is common in cats. Disease progression is associated with inflammation and fibrosis leading to further tubular injury. Thymosin β4 (Tβ4) reduces inflammation and fibrosis in murine CKD models, however, its role in feline CKD is unknown. This study aimed to assess the expression of Tβ4 and Tβ10 in cats with naturally occurring CKD.

Kidney samples obtained at post‐mortem from non‐azotaemic cats (*n* = 16), cats with stable CKD (plasma creatinine concentration >2.0 mg/dl; *n* = 23) and cats with progressive CKD (over 25% increase in plasma creatinine in the year since diagnosis; *n* = 11) were compared in this cross‐sectional study. Renal histology was performed by a board‐certified veterinary pathologist. Expression of TMSB4X and TMSB10, encoding Tβ4 and Tβ10, was assessed by RT‐qPCR and normalised to RPS7 followed by statistical analysis (one‐way ANOVA/Kruskal‐Wallis) and correlation (Spearman) with histopathology scores and plasma creatinine levels. Tβ4 localisation was assessed by immunohistochemistry.

Kidney TMSB4X (*P*=0.011) and TMSB10 (*P*=0.023) mRNA levels were significantly higher in progressive CKD compared with non‐azotaemic cats. TMSB4X expression was also higher in cats with progressive compared with stable CKD (P=0.015). TMSB4X and TMSB10 expression positively correlated with interstitial fibrosis (TMSB4X: *r* = 0.391, *P*=0.008; TMSB10: *r* = 0.552, *P* < 0.0001), inflammation (TMSB4X: *r* = 0.329, *P*=0.027; TMSB10: *r* = 0.570, *P* < 0.0001) and plasma creatinine (TMSB10: *r* = 0.474, *P*=0.0004). Tβ4 was localised to podocytes in the glomerulus, and macrophages, T cells and fibroblasts in the tubulointerstitium.

In conclusion, β thymosins may play a role in the pathophysiology of feline CKD. Tβ4 expression in immune cells and fibroblasts indicates a role for Tβ4 in renal inflammation and fibrosis.

**Disclosures**

This work was supported by a PhD studentship award funded by the Royal Veterinary College and The Beryl Evetts and Robert Luff Animal Welfare Trust and by a PetPlan Charitable Trust pump primer award. Sarah Spencer is in receipt of a studentship in part funded by CEVA Animal Health and the BBSRC. Jonathan Elliott receives Grant funding from CEVA Animal Health, Boehringer Ingelheim, Idexx Laboratories, Royal Canin, Waltham Petcare Science Institute and Zoetis. He is engaged as a consultant by: Boehringer Ingelheim, Elanco Animal Health, Merck‐Intervet Animal Health, Idexx Laboratories, InvetX Ltd., Purina, Variant Bio, Waltham Petcare Science Institute. Rosanne Jepson receives funding from Boehringer Ingelheim and engages as a consultant at Boehringer Ingelheim.

**Source of Funding**

**ECVIM CSF Funding**

No.

**ESVNU** – **European Society of Veterinary Nephrology and Urology**

## ESVNU‐O‐3

## Usefulness of parathormone, calcitriol and parathyroid glands ultrasound in differentiating acute kidney injury and acute on chronic kidney disease in dogs

### P. Remmel^1^, V. Leynaud^2^, M. Fenet^3^, C. Layssol‐Lamour ^4^, J. Mortier^3^, T. Buronfosse^5^, C. Trumel^4^, G. Benchekroun^1^, R. Lavoué ^6^

#### ^1^Ecole Nationale Vétérinaire d'Alfort, CHUVA, Service de médecine interne, Maisons alfort, France; ^2^Université de Toulouse, INRAE, ENVT, Toulouse, France; ^3^Ecole Nationale Vétérinaire d'Alfort, CHUVA, Service d'imagerie médicale, Maisons alfort, France; ^4^CREFRE, Université de Toulouse, INSERM, UPS, ENVT, Toulouse, France; ^5^VetAgro Sup—Service De Biologie Clinique, Marcy‐l'étoile, France; ^6^INTHERES, Université de Toulouse, INRAE, ENVT, Toulouse, France

Early differentiation of acute‐on‐chronic‐kidney‐disease (ACKD) from acute‐kidney‐injury (AKI) is important for diagnostic and therapeutic management. While clinicopathological and imaging findings might help, discriminating ACKD from AKI remains challenging in some cases.

Our objectives were to assess the utility of parathyroid glands ultrasound and biomarkers of calcium/phosphate metabolism assessment in differentiating AKI and ACKD.

Client‐owned dogs presented for recent onset of clinical azotemia were prospectively included and allocated into AKI or ACKD groups based on final diagnosis. Blood was collected within 2 days after admission and creatinine, phosphates, ionized and total calcium, calcitriol and parathormone were measured. When possible, dogs underwent parathyroid ultrasonography.

Twenty‐two AKI and 20 ACKD of various breeds were included. Groups were comparable for age, weight and sex.

Serum creatinine (*p* = 0.004, mean difference: 147 μmol/L), parathormone (*p* = 0.012, mean difference: 149 pg/mL) and parathormone/calcitriol ratio (*p* = 0.038, mean difference: 1.73) were significantly higher in ACKD. Parathormone/calcitriol had the lowest overlap between groups (median [25–75% percentile]; AKI: 0.689 [0.222–2.084] vs. ACKD 3.365 [2.075–6.275]) with an area under the Receiver‐Operating‐Characteristic (ROC) curve analysis of 0.82.

Among ultrasonographic measures, only the mean area of parathyroid glands in longitudinal axis (*p* = 0.022, mean difference: +1 mm^2^) and its ratio to ipsilateral common carotid arteries (*p* = 0.022, mean difference: +0.1) were significantly higher in AKCD. This ratio had the lowest overlap between groups (AKI: 0.349 [0.257–0.592] vs. ACKD 0.628 [0.467–0.796]) with an area under the ROC curve analysis of 0.76.

Parathormone/calcitriol and mean area of parathyroid glands/ipsilateral common carotids area ratio might help to discriminate AKI from ACKD.

**Disclosures**

**Source of Funding**

**ECVIM CSF Funding**

No.

**ESVNU – European Society of Veterinary Nephrology and Urology**

## ESVNU‐O‐4

## Comparison of long‐term outcomes after SUB implantation in cats with and without systematic flush: A retrospective study of 128 cases (2013–2024)

### A. Corsaletti^1^, E. Robin^2^, E. Darnis^2^, C. Bismuth^3^, C. Poncet^3^, E. Gomes^4^, K. Le Boedec^2^

#### ^1^Centre Hospitalier Vétérinaire Frégis—IVC Evidensia France, Gentilly, France; ^2^Centre Hospitalier Vétérinaire Frégis—IVC Evidensia France Internal medicine, Gentilly, France; ^3^Centre Hospitalier Vétérinaire Frégis—IVC Evidensia France Surgery, Gentilly, France; ^4^Centre Hospitalier Vétérinaire Frégis—IVC Evidensia France Imaging diagnostic, Gentilly, France

Subcutaneous Ureteral Bypass (SUB) devices have been developed to overcome the limitations of traditional techniques for ureteral obstruction in cats and are now widely used. The manufacturer follow‐up recommendations are to flush the device at 1 week and 1 month post‐operatively, and every 3 months thereafter, in order to prevent obstruction and biofilm formation and ensure a better prognosis. However, these procedures are time and money consuming and there is no current evidence that flush impacts the long term prognosis. The objective of this study was to compare outcomes of cats with ureteral obstruction managed with SUB with or without regular flushing procedures, and determine the effect of flushing on recurrence of obstruction, infection occurrence and cystitis rate.

Medical records of cats treated with a SUB device between 2013 and 2024, and having at least one month of follow‐up including either a regular SUB flush (group F) or no regular flush (group NF) were reviewed. Data extracted from records including type of food, biochemical data, and medical events such as recurrence of obstruction, infection or cystitis were recorded and compared between groups with univariate and multivariate Cox's proportional hazards analyses.

One hundred and twenty‐eight SUBs were implanted, with 43 in group F and 85 in group NF. Median survival time of cats was 1460 days (interquartile range of survival time: 656–2735 days). Absence of regular SUB flush was not significantly associated with time to death (median survival time: 1397 and 1460 days in the F and NF groups, respectively; *P*=0.60), even after controlling for age and creatinine at the last visit (adjusted hazard ratio: 0.69; 95% confidence interval: 0.41–1.18; *P*=0.18). The median time to the first recurrence of obstructive event after SUB placement was not statistically different between the two groups (704 and 767 days in the F and NF groups respectively; *P*= 0.60). No significant differences in time to infection and occurrence of cystitis were found between the two groups (*P*= 0.82 and *P*=0.58, respectively).

Based on these results, absence of regular SUB flush was not significantly associated with time to recurrence of obstruction. However, reducing SUB handling did not significantly reduce occurrence of cystitis and infection rate in cats treated with SUB. Managing SUB without regular flushing could be considered to reduce associated costs without negatively impacting the outcome.

**Disclosures**

**Source of Funding**

None.

**ECVIM CSF Funding**

No.

**ESVNU** – **European Society of Veterinary Nephrology and Urology**

## ESVNU‐O‐5

## Increased serum amyloid A in cats with pyelonephritis

### A.S. Gravgaard^1^, F. Moberg^1^, R. Langhorn^1^, A. Jensen^1^, N. Dupont^1^, N. Bak‐Jacobsen ^1^, B.J. Reezigt^2^, I. Kieler^1^, L.N. Nielsen^1^, L.R. Jessen^1^

#### ^1^Department of Veterinary Clinical Sciences, University of Copenhagen Department of Veterinary Clinical Sciences, University of Copenhagen, Copenhagen, Denmark; ^2^Blue Star Veterinary Hospital, Gothenburg, Sweden

Clinical signs in cats with pyelonephritis may be vague and unspecific, and the diagnosis ultimately relies on positive culture of urine obtained by pyelocentesis. This study aims to investigate serum amyloid A (SAA), a major acute phase protein, as a biomarker for feline pyelonephritis.

SAA concentrations from 68 cats were included in this prospective case‐control study (2015–2023), of which 48 were prospectively recruited to this study, and 20 were prospectively recruited to other studies with their data included retrospectively to the present study. Cats were categorized into 4 groups; healthy (*n* = 15), confirmed pyelonephritis (*n* = 14), suspected pyelonephritis (*n* = 5) and other urinary tract disorders (*n* = 34). The majority of cats with confirmed or suspected pyelonephritis had complicated pyelonephritis. Other urinary tract disorders consisted of chronic kidney disease (*n* = 10), ureteral obstruction (*n* = 13), subclinical bacteriuria (*n* = 6) and cystitis (*n* = 5). SAA was measured using an automated latex‐enhanced immunoturbidimetric assay (Eiken Chemicals Ltd, Japan) on an automated analyzer (Atellica CH, Siemens Healthineers, Germany).

SAA was significantly higher in cats with confirmed pyelonephritis (median 180.3 mg/L, 10th–90th percentile: 145.4–661.6 mg/L) and cats with suspected pyelonephritis (607.4 mg/L, 0.0–693 mg/L) when compared to healthy cats (0.0 mg/L, 0.0–0.5 mg/L, *p* < 0.0001) and cats with other urinary tract disorders (0.4 mg/L, 0.0–22.4 mg/L, *p* < 0.0001). ROC analysis revealed an optimal cut‐off value for SAA at 49.1 mg/L (SE: 1.00 [95CI 0.77–1.00], SP: 0.97 [95CI 0.85–0.99]), corresponding to a positive likelihood ratio of 34.

These results suggest that SAA reliably discriminates pyelonephritis from other urinary tract disorders in cats.

**Disclosures**

L.R. Jessen: Non‐subject matter speakers engagements with WSAVA, ACVIM, Vet Group, ECVIM and ICOHAR. R. Langhorn: Research funded by Agria and SKK's Research Fund and by the Fund of Scientific Studies in Companion Animals. Prior Speaker engagement with various larger clinical practices in Denmark. B.J. Reezigt: Occasional webinars for OrionPharma, Agria and FirstVet L.N. Nielsen: Financial relationship with Eiken Chemicals Ltd (2016–2018) and several non‐subject matter speaker engagements with medical companies (Elanco and Vet Group).

**Source of Funding**

Agria.

**ECVIM CSF Funding**

No.

**ESVNU** – **European Society of Veterinary Nephrology and Urology**

## ESVNU‐O‐6

## Urinary levels of interleukin‐8 in dogs with bacteriuria

### D. Erika Leth Vasby^1^, T. Møller Sørensen^1^, L. Rem Jessen^1^, H. Frøkiær^2^, B.J. Reezigt^3^, K. Höyer Madsbøll^1^, L. Spanggaard Kristiansen^2^

#### ^1^University of Copenhagen, Faculty of Health and Medical Sciences Department of Veterinary Clinical Sciences, Frederiksberg, Denmark; ^2^University of Copenhagen, Faculty of Health and Medical Sciences Department of Veterinary and Animal Sciences, Frederiksberg, Denmark; ^3^Blå Stjärnans Djursjukhus, Göteborg, Sweden

Subclinical Bacteriuria (SBU) is characterized by significant bacteriuria in the absence of clinical signs of urinary tract infections (UTI). The absence of clinical signs may partly be explained by the host immune response. Studies of urinary levels of interleukin‐8 (uIL‐8) in people are conflicting. However, some have shown significant higher levels in patients with cystitis compared to asymptomatic bacteriuric patients. The aim of this study was to investigate levels of uIL‐8 in bacteriuric dogs with and without clinical signs. Surplus urine samples from 53 dogs prospectively collected for other studies were included in the study. Dogs were categorized as either SBU (*n* = 14) or UTI (cystitis (*n* = 15) or pyelonephritis (*n* = 7)). Furthermore, dogs without bacteriuria (*n* = 17) served as controls. Levels of uIL‐8 were measured by sandwich ELISA and normalized to the urinary specific gravity. The median and range in the different groups were calculated and non‐parametric statistical analysis were performed. Median levels were 347.1 pg/mL (range 0.00–3228 pg/mL), 821.6 pg/mL (range 17.57–6398 pg/mL), 7497 pg/mL (range 37.94–9593 pg/mL) and 5.06 pg/mL (range 0.00–89.36) for the SBU, cystitis, pyelonephritis and control groups, respectively. The normalized uIL‐8 levels where significantly higher (*p* < 0.0001) in the bacteriuric dogs versus non‐bacteriuric dogs. In addition uIL‐8 was significantly increased (*p* = 0.012) in the pyelonephritis group compared to the SBU group. This concludes that although the levels of uIL‐8 increases with severity of the urinary disease, values are overlapping between groups. In our study population, uIL‐8 levels did not significantly differ between dogs with cystitis and SBU.

**Disclosures**

Lisbeth Rem Jessen: not‐Subject matter speakers engagements Wsava, Acvim, Vet group, VIN, Ecvim, ICOHAR.

**Source of Funding**

Peter Christian Abildgaards og overdyrlæge Hans A. Madsen og hustrus fond.

**ECVIM CSF Funding**

No.

**ESVNU** – **European Society of Veterinary Nephrology and Urology**

## ESVNU‐O‐7

## Evaluation of probiotic supplementation in dogs with chronic kidney disease

### I. Lopez^1^, E. Aguilera‐Tejero ^1^, C. Arenas^2^, G. Glorieux^3^, C. Pineda^1^

#### ^1^University Cordoba Animal Medicine and Surgery, CÓRDOBA, Spain; ^2^Hospital Veterinario Valencia Sur Anicura Internal Medicine Service, Valencia, Spain; ^3^Ghent University Hospital Internal Medicine and Pediatrics, Nephrology Section, Ghent, Belgium

Probiotic supplementation has been suggested as an adjuvant therapy to improve the balance of the gut microbiota contributing to intestinal barrier integrity and metabolic control of human patients with chronic kidney disease (CKD). However, there is no information about the potential benefits of probiotics in dogs with CKD. The aim of this study was to investigate the effects of a commercial probiotic formulation for companion animals on kidney function and uremic toxins levels in dogs with CKD.

Sixteen dogs with CKD (IRIS Stage II and III) were recruited to a double‐blind, placebo‐controlled, randomized study. At the time of inclusion, the dogs were already on a renal diet and continued with the same diet added with placebo (*n* = 8) or the commercial probiotic (Fortiflora® Nestle Purina PetCare) (*n* = 8).

All animals were subjected to a physical exam, an analytical control (complete blood count, blood biochemistry panel and urinalysis) and blood pressure measurement at the beginning of the treatment (T0), one month later (T30) and at the end of the procedure (T60). In addition, plasma uremic toxins (indoxyl sulphate (IS) and p‐cresyl sulphate) were measured at T0 and T60.

No changes in body weight or body condition score were found during the study in any of the study groups. Probiotic administration resulted in a significant decrease in plasma concentration of symmetric dimethylarginine (SDMA) both at T30 and T60 (28.0 ± 3.9 y 27.3 ± 3.2 ul/dl, *p* = 0.031, *p* =0.008 respectively) when compared with T0 (30.4 ± 3.6 ul/dl). Urine specific gravity and UPC did not change during the study. Systolic blood pressure (SBP) tended to increase in the placebo group at T30 and the differences were significant at T60 (163 ± 11 mmHg vs. 144 ± 6 mmHg, *p* = 0.033). However, no increase in SBP was found in the probiotic group. There was a significant reduction in IS in dogs on probiotics (0.27 ± 0.10 mg/dl, *p* = 0.04) at T60 when compared with T0 (0.41 ± 0.15 mg/dl). No adverse effects were registered in dog receiving probiotics.

Based on the results, it can be concluded that this probiotic is safe for dogs with CKD. In addition, when compared with the placebo group, the dogs receiving probiotic did not experience an increase in SBP. Moreover, the dogs receiving probiotic showed a significant decrease in plasma SDMA and IS. Thus, the available data support a beneficial effect of probiotic in dogs with CKD.

**Disclosures**

This study was funded by Nestle Purina PetCare Switzerland. Fortiflora® was also supplied by Nestle Purina PetCare Switzerland. Although the study was funded by Nestle Purina PetCare Switzerland and Fortiflora® was also supplied by them, the funder had no role in study design, data collection and analysis, decision to publish or preparation of the abstract. Besides that, thanks to placebo group, the study was designed as double‐blind, placebo‐controlled, randomized study to avoid any source of bias.

**Source of Funding**

This study was funded by Nestle Purina PetCare Switzerland. Fortiflora

**ECVIM CSF Funding**

No.

**ESVNU** – **European Society of Veterinary Nephrology and Urology**

## ESVNU‐O‐8

## Clinical findings, prognostic factors and outcomes of feline protein losing nephropathy secondary to glomerular disease: A retrospective study

### N. Sugar^1^, G. Segev^1^, H.H. Chen^1^

#### ^1^Koret School of Veterinary Medicine, The Hebrew University of Jerusalem Internal Medicine, Rishon Lezion, Israel

Feline protein losing nephropathy (PLN) due to glomerular disease have substantial clinical implications, however, clinical signs, clinicopathologic findings and outcome are not well described. We aimed to characterize the disease and to identify prognostic factors. PLN was diagnosed based on persistent renal proteinuria (UPC>2). Thirty‐seven cats (46% males and 54% females) were included, with a median age of 3 years (range, 1.5–11.5). The most common clinical signs at presentation were weakness/lethargy (51%) and edema (41%). Twenty‐five cats (67%) survived to discharge. Edema was the only clinical sign associated with short‐term (30‐days) survival (OR‐6, CI95:1.3–26.8, *P*=0.02). Urea (*P* < 0.001), creatinine (*P*=0.020), phosphorus (*P*=0.004), AST (*P*=0.016), and CK (*P*=0.002) at presentation were higher in non‐survivors compared with survivors, while glucose (*P*=0.009), total calcium (*P*=0.012) and MCHC (*P* =0.046) were lower. The most common treatments administered were anti‐proteinuric, antithrombotic, antibiotics, and immunosuppression. There was an association between antithrombotic administration and survival to discharge (OR‐4.2, CI95%:0.98–17.95, *P*=0.048). Complete or partial remission (UPC<0.4 or >50% reduction in UPC, respectively) were documented in 2 (5%) and 10 (33%) cats, respectively. Survival analysis revealed that complete or partial remission was associated with survival (estimated mean days±SE: 3853±936 days vs. 177±65 days, *P*=0.002), as was creatinine (OR‐1.25 CI95%: 1.03–1.52, *P* < 0.01). Follow‐up was available for 16 cats, of which 2 died within 3 months, 5 within 1 year, and 9 survived >1 year. In conclusion, short‐term prognosis is guarded, however, a third of cats achieve remission and have favorable long‐term outcome. Presentation with edema (rather than azotemia) was associated with better outcome.

**Disclosures**

**Source of Funding**

**ECVIM CSF Funding**

No.

**ESVNU – European Society of Veterinary Nephrology and Urology**

## ESVNU‐O‐9

## Effects and remanence of short‐course of telmisartan or benazepril on renal function of healthy dogs: a prospective, cross‐over, double‐masked, placebo‐controlled study

### P. Bernard^1^, M. Mantelli^1^, L. Chapuis^1^, G. Essner^1^, C. Trumel^1^, A. Diquélou^1^, R. Lavoué ^1^

#### ^1^National Veterinary School of Toulouse, Toulouse, France

Telmisartan (Telmi) and benazepril (Bena) are renin‐angiotensin‐aldosterone system blockers frequently prescribed in canine chronic kidney disease. Few data are available concerning their compared effects on canine renal function. This study aimed to evaluate renal function of healthy dogs treated with Telmi or Bena versus placebo (P).

Fifteen healthy Beagles dogs were prospectively included in this symmetric 3 × 3 cross‐over double‐masked, placebo‐controlled study and allocated to receive a 7‐day course of Telmi (1mg/kg once daily), Bena (0.5 mg/kg twice daily) or P. A 3‐weeks wash‐out period was observed between each treatment. Blood pressure, 24‐hours urine collection, glomerular filtration rate (GFR) using exogenous creatinine clearance, urinalysis, complete plasma and urine biochemistry were obtained before and after each period. A mixed general linear model and post‐hoc comparisons were used to analyze the effects of treatments.

No clinical side effects were observed. As no period nor sequence effect was found, the cross‐over was broken. GFR was not affected by treatment; however, one dog experienced a 53% decreased in GFR after Telmi. When this dog was withdrawn from analysis, GFR was significantly higher with Bena compared to P (+6.4%, *p* = 0.049), but not to Telmi. Variables which were significantly lower with Bena compared to Telmi were plasma potassium (−0.18 mmol/L, *p* = 0.045) and urine sodium (−52.39 mmol/L, *p* = 0.027).

Although neither Bena nor Telmi induced clinically relevant change in renal function, one dog experienced a marked decrease in GFR after a 7‐day course of Telmi that might have consequences in dogs with previously impaired renal function.

**Disclosures**

Residency program of the presenting author partially financed by a compagny (Royal Canin).

**Source of Funding**

**ECVIM CSF Funding**

No.

**ESVNU** – **European Society of Veterinary Nephrology and Urology**

## ESVNU‐O‐10

## Urinary Clusterin and Cystatin B in Dogs with Spontaneous Acute Kidney Injury

### E. Gordin^1^, S. Viitanen^1^, D. Gordin^2^, D. Szlosek^3^, S. Peterson^4^, T. Spillmann^1^, M. Labato^5^

#### ^1^University of Helsinki Department of Equine and Small Animal Medicine, Helsinki, Finland; ^2^Helsinki University Hospital Department of Nephrology, Helsinki, Finland; ^3^IDEXX Laboratories IDEXX Laboratories, Westbrook, Maine, United States; ^4^Idexx Laboratories, Westbrook, Maine, United States; ^5^Tufts University, North Grafton, United States

Novel biomarkers are needed to more reliably diagnose acute kidney injury (AKI) in dogs and in predicting morbidity and mortality after AKI. Our hypothesis was that two novel tubular biomarkers, urinary clusterin (uClust) and cystatin B (uCysB), are elevated in dogs with AKI of different etiologies. Serum and urine samples were collected from 18 dogs with AKI of different severity and of various etiology and from 10 healthy control dogs in a prospective, longitudinal observational study. Urinary clusterin and uCysB were measured by ELISA and compared at inclusion between dogs with AKI and healthy controls and remeasured one and three months later. Dogs with AKI had higher initial levels of uClust (median 3593 ng/mL; interquartile range [IQR]; 1489–10483 ng/mL) and uCysB (median 554 ng/mL; [IQR] 29–821 ng/mL) compared to healthy dogs (median 70 ng/mL; [IQR] 70–70 and median 15 ng/mL; [IQR]15–15; *p* < 0.001, respectively). Initial uCysB was higher in dogs that died during the one–month follow–up period (*n* = 10) (median 731 ng/mL; [IQR] 517–940), compared to survivors (*n* = 8) (median 25 ng/mL; [IQR] 15–417 (*p* = 0.009). Based on these results, uClust and especially uCysB are promising biomarkers of AKI. Further, they might reflect severity of tubular injury known to be central in the pathology of AKI.

**Disclosures**

Donald Szlosek and Sarah Peterson have an affiliation to the commercial funders of this research, as employees of IDEXX Laboratories, Inc. The work presented in this study was supported in part by IDEXX Laboratories, Inc. Westbrook, ME, USA (https://www.idexx.com/en/about-idexx/). IDEXX Laboratories, Inc. holds certain patent rights relating to assays for the detection of SDMA, clusterin and cystatin‐B and manufactures the IDEXX SDMA® Test utilized in this study. D. Szlosek and S. Peterson were employed by IDEXX Laboratories, Inc., during the time of the study. D. Szlosek and S. Peterson report ownership interest in IDEXX Laboratories, Inc. Both have stock in IDEXX. Daniel Gordin was supported by Liv och Hälsa Society, Medical Society of Finland (Finska Läkaresällskapet), Sigrid Juselius Foundation, Helsinki University Hospital, University of Helsinki, Minerva Foundation Institute for Medical Research Clinician Scientist, and Academy of Finland (UAK1021MRI). He has received Lecture or Advisory Board Honoraria: Astellas, AstraZeneca, Bayer, Boehringer Ingelheim, GE Healthcare, Fresenius, Novo Nordisk. Sanna Viitanen has received Lecture Honoraria from Fennovet Oy, Evidensia Oy, Royal Canine Scandinavia and IDEXX Europe, and holds a board member position at Veterinary Clinic Avec, Porvoo, Finland, all outside of this work. The other authors declare no conflicts of interests.

**Source of Funding**

This research was funded by IDEXX Laboratories, Inc. Westbrook, ME, USA, Orion Research Foundation; Arvid och Greta Olins fond, The Swedish Cultural Foundation in Finland; Finnish Foundation of Veterinary Research; and The Finnish Veterinary Foundation (Helvi Knuuttila Funds). Emilia Gordin received an university‐funded doctoral researcher position from the University of Helsinki for this work.

**ECVIM CSF Funding**

No.

**ESVNU** – **European Society of Veterinary Nephrology and Urology**

## ESVNU‐O‐11

## Innovation of polycystic kidney disease test kit by loop‐mediated isothermal amplification

### K. Jaturunratsamee^2^, S. Petchdee^1^

#### ^1^Faculty of Veterinary Medicine, Kasetsart University Faculty of Veterinary Medicine, Kasetsart University, Bangkok, Thailand; ^2^Faculty of veterinary Medicine, Kasetsart University, Bangkok, Thailand

The feline PKD1 gene was analyzed to identify specific mutations that cause polycystic kidney disease. Cats that carried a heterozygous CA transversion at c.10063 in exon 29, were found to have polycystic kidney disease. Loop‐mediated isothermal amplification (LAMP), which is based on advanced genetic research, is one of the most powerful methods for detecting single nucleotide polymorphisms (SNP). LAMP diagnostic tests can be performed at point‐of‐care (POC) using simple instruments like thermocyclers, heated blocks, or water baths at a constant temperature. The study included 30 cats of various breeds, with an average age of 39.74 ± 8.68 months. The results showed that PKD1 mutations were present in 23.33% of cats, with the highest prevalence found in Persian and Persian‐related breed cats. The LAMP technique can differentiate between wild‐type cats and those with a PKD1 mutant gene. The LAMP reactions could be finished at a single temperature of 61–63°C in 60 min. The novel PKD1‐LAMP with a lateral‐flow dipstick (LFD) test is an efficient and accurate assay for detecting PKD1 mutations quickly, affordably, and with minimal use of expensive, complex equipment. As a result, this assay has great potential and can be used as a new screening test for polycystic kidney disease in felines caused by PKD1 mutations.

**Disclosures**

**Source of Funding**

Faculty of Veterinary Medicine, Kasetsart University.

**ECVIM CSF Funding**

No.

**ESVNU** – **European Society of Veterinary Nephrology and Urology**

## ESVNU‐O‐12

## Evaluation of serum C‐reactive protein in dogs with urinary tract infections

### F. Fidanzio^1^, I. Tirelli^1^, G. Colombo^1^, M.C. Sabetti^1^, A. Tirolo^1^, F. Dondi^2^, A. Corsini^1^

#### ^1^University of Parma Department of Veterinary Sciences, Parma, Italy; ^2^Alma Mater Studiorum‐University of Bologna Department of Veterinary Medical Sciences, Bologna, Italy

C‐reactive protein (CRP) is an acute phase protein frequently used to diagnose and monitor systemic inflammatory state. Serum CRP concentration is increased in human patients with upper urinary tract infections (UTI) but not in patients with lower UTI; however, its role is poorly described in dogs with UTI. The aims of this retrospective cross‐sectional study were 1) to evaluate serum CRP in dogs with bacterial cystitis (BC), acute pyelonephritis (AP) and acute bacterial prostatitis (ABPR); 2) to compare serum CRP among these groups.

Thirty‐two client‐owned dogs were included:11 had BC, 11 had AP, 7 had ABPR, 3 had both AP and ABPR.

Dogs were included if they had a positive urine culture. BC was diagnosed if urological signs and active urine sediment were present. AP was diagnosed if at least 4 of these criteria were satisfied: systemic clinical signs (i.e., fever, hyporexia, vomiting, and lethargy), leukocytosis, active urine sediment, acute renal injury (defined according with IRIS guidelines), suggestive ultrasound abnormalities. ABPR was diagnosed if at least 4 of these criteria were satisfied: systemic clinical signs, disease‐related clinical signs (i.e., stranguria, tenesmus, preputial discharge, and pain at rectal palpation), leukocytosis, active urine sediment, suggestive ultrasound abnormalities. Dogs with comorbidities possibly affecting serum CRP (i.e., pancreatitis, pneumonia, immune‐mediated diseases, and Cushing's syndrome) were excluded. Serum CRP was measured at the time of diagnosis.

Kruskal–Wallis test followed by Dunn multiple comparison test was used to compare serum CRP among the three groups. Unpaired *t*‐test was used for comparison between BC and the group including dogs with AP, dogs with ABPR, and dogs with both (group APP). Receiver operating characteristic curve analysis was used to assess sensitivity and specificity. Statistical significance was set at *P* < .05.

Dogs with BC had significantly lower serum CRP (1.3 mg/dL, 0.08–3.6) compared with dogs with AP (26 mg/dL, 5.3–46, *P* < 0.0001) and ABPR (9.6 mg/dL, 6.6–46, *P*=0.01). No differences were documented between AP and ABPR (*P*=0.88). Group APP had significantly higher serum CRP compared with group BC (22.3±13.7 mg/dL vs. 1.5±1.3 mg/dL; mean difference=20.7, 95% CI: 12.2–29.3; *P* < 0.0001).

Serum CRP >4.48 mg/dL had specificity=100% (95% CI: 0.74–1.0) and sensitivity=100% (95% CI: 0.84–1.0) to discriminate dogs with BC and dogs with APP.

Serum CRP concentrations at the diagnosis accurately differentiate dogs with bacterial cystitis from those with acute pyelonephritis and/or prostatitis. In dogs with UTI and markedly elevated CRP the presence of acute pyelonephritis or acute prostatitis should be suspected.

**Disclosures**

**Source of Funding**

**ECVIM CSF Funding**

No.

**ESVNU** – **European Society of Veterinary Nephrology and Urology**

## ESVNU‐O‐13

## New insights into the role of adrenal function in chronic kidney disease

### P. Lunet Marques^1,2^, L. Mateus^1,2^, S. Galac^3^, R. Oliveira Leal^1,2^

#### ^1^Centre for Interdisciplinary Research in Animal Health, Fac.Vet.Med., ULisbon Clinical research, Lisbon, Portugal; ^2^Associate Laboratory for Animal and Veterinary Sciences (AL4AnimalS) Clinical research, Lisbon, Portugal; ^3^Utrecht University—Faculty of Veterinary Medicine Clinical Sciences, Utrecht, Netherlands

Adrenal function has been assessed in specific feline systemic diseases, with changes in cortisol reserves being reported in hyperthyroidism and cholestatic liver disease. However, the involvement of cortisol and adrenal function in the development and progression of chronic kidney disease (CKD) remains unclear. This study aims to investigate the possible link between biomarkers of CKD and basal cortisol (T0), post‐ACTH‐stimulation cortisol (T1), delta‐cortisol (T1‐T0) and endogenous ACTH (eACTH) levels in feline CKD. We hypothesize that cats with CKD may exhibit chronic relative hypercortisolism and an increased response rate to exogenous ACTH.

After ethical approval and owner's consent, a cross‐sectional study was performed including otherwise healthy cats with stable CKD (IRIS stage II–III) not receiving any anti‐hypertensive medication. All cats underwent a physical examination, blood pressure measurements, blood tests (including SDMA, BUN, creatinine, as well as others), urinalysis and an ACTH‐stimulation test (two blood samples collected 1‐hour apart after administering 125 μg tetracosactide intramuscularly). Serum samples were stored at ‐80°C until analysis. Cortisol and eACTH were assessed by chemiluminescence methodology (Immulite 1000, Siemens). Following the 3Rs policy, obtained values for T1, T0 and delta‐cortisol were compared with previous studies on healthy cats and those with chronic illness.

A total of 10 cats were enrolled. The T0 values (median [range]: 141.26 [25.59–300.73] nmol/L) were slightly higher than those reported in cats with chronic illness (121.3 [41.6200.7] nmol/L) and healthy cats (129.2 [59.2–199.2] nmol/L). The T1 values (311.77 [141.54–675.96] nmol/L) were higher than the previously reported in healthy cats (256.3 [174.7–337.9] nmol/L) and cats with chronic illness (220.6 [135.1–306.1] nmol/L). Delta‐cortisol values (216.03 [‐13.8 to 375.22] nmol/L) were higher than in healthy cats (127.1 [51.9–202.3] nmol/L) and cats with chronic illness (99.4 [25.6–173.27] nmol/L).

A significant correlation was identified between delta‐cortisol and serum BUN (*r* = 0.699, *p* = 0.024), creatinine (*r* = 0.743, *p* = 0.014) and SDMA (*r* = 0.729, *p* = 0.017). A significant correlation was also found between T1, BUN (*r* = 0.715, *p* = 0.02) and SDMA (*r* = 0.762, *p* = 0.01). Endogenous ACTH, delta‐cortisol, T0 and T1 did not correlate with any other variables (blood pressure, potassium, phosphate, urinary protein‐creatinine ratio and urine specific gravity).

This study shows that high delta‐cortisol and post‐ACTH cortisol correlate with CKD biomarkers, highlighting a possible effect of the hypothalamic‐pituitary‐adrenal axis on the pathophysiology and progression of feline CKD and offering new insights into potential prognostic markers for this disease.

**Disclosures**

**Source of Funding**

This work was supported by FCT—Fundação para a Ciência e Tecnologia IP, grant UIDB/00276/2020 and by LA/P/0059/2020—AL4AnimalS.

**ECVIM CSF Funding**

No.

**ESVNU** – **European Society of Veterinary Nephrology and Urology**

## ESVNU‐O‐14

## Mycoplasma‐associated urethritis in dogs: A case series

### D. Pichard^1^, M. Kurtz^2^, P. Remmel^2^, M. Canonne^2^, G. Benchekroun^2^, C. Maurey^2^

#### ^1^Ecole Nationale Vétérinaire d'Alfort, Maisons‐Alfort, France; ^2^Ecole Nationale Vétérinaire d'Alfort, Maisons‐Alfort, France

Canine idiopathic functional urinary outflow tract obstruction is an uncommon condition, in which urethritis is a possible underlying disease. Mycoplasma‐associated urethritis in humans is recognized as a frequent cause of dysuria. In veterinary medicine, the role of *Mycoplasma spp*. in urethritis remains poorly understood.

The objective of this study was to describe dogs with presumptive idiopathic functional urinary outflow tract obstruction in which *Mycoplasma spp*. infection was researched. Dogs with dysuria and *Mycoplasma spp*.PCR testing on urine or urethra/bladder sampling were retrospectively included. Dogs with urethral obstructions (urethrolith, neoplasia, stricture…), prostatic disease (other than benign prostatic hypertrophy), urinary tract infection (UTI) in which dysuria resolved with antimicrobial treatment, or primary neurological disease were excluded from the study.

Eighteen dogs were included between 2017 and 2024. Sixteen had negative (group M−) and 2 had a positive (group M+) *Mycoplasma spp*. PCR on urine and/or urethral swab. Median age was 3.4 [2.5–8.1] (M‐) and 7.5 [5.3–9.7] (M+) years old. Median weight was 39 kg [38.5–41.5] (M−) and 36 kg [34–38] (M+). Clinical presentations were similar in both groups with incontinence (7/16 M−, 1/2 M+), pollakiuria (7/16 M−, 1/2 M+), stranguria (2/16 M−, 1/2 M+) and hematuria (2/16 M−, 1/2 M+). Urinalysis showed negative bacterial cultures for 8/10 dogs in group M− and 1/2 in group M+. Endoscopic evaluation was performed in 16/18 dogs, with macroscopic signs of urethritis observed in 12/14 dogs M−, and marked signs in both dogs M+. In group M−, 7 dogs had *Mycoplasma spp*. testing on urine sample, 3 on urethral sample and 1 on both. In group M+, 1 dog had positive *Mycoplasma spp*. PCR on urine and 1 on urethral sample (concomitant negative PCR on urine). Treatment for dogs M− involved non‐steroidal anti‐inflammatory drugs (*n* = 12), alfusosine (*n* = 6), tamsulosine (*n* = 5), dantrolene (*n* = 5), amoxicillin (*n* = 4), cephalexin (*n* = 1), marbofloxacine (*n* = 1). All dogs had mild to moderate improvement. For the dogs M+, treatment involved meloxicam (*n* = 1), firocoxib (*n* = 1), and marbofloxacin (*n* = 2). Clinical resolution was observed in both cases, without relapse.

This case series, describes *Mycoplasma spp*. detection in dogs presented for dysuria. Incidence seems to be lower than previously reported in urine testing in dogs with UTI. It also highlights the possible necessity of urethral sampling when urine PCR is negative. Targeted antibiotic therapy allowed clinical resolution in the 2 dogs, without any relapse as opposed to dogs M‐.

**Disclosures**

Royal Canin residency funding.

**Source of Funding**

Royal Canin residency funding.

**ECVIM CSF Funding**

No.

**ESVNU** – **European Society of Veterinary Nephrology and Urology**

## ESVNU‐O‐15

## Renal health in aging apparently healthy dogs: panel of serum and urinary biomarkers

### S. Marynissen^1^, S. Daminet^1^, E. Meyer^2^, L. Duchateau^3^, K. Demeyere^2^, J. Delanghe^4^, P. Smets^1^, D. Paepe^1^

#### ^1^Small Animal Department, Faculty of Veterinary Medicine, Ghent University, Merelbeke, Belgium; ^2^Lab of Biochemistry, Department of Veterinary and Biosciences, Faculty of Veteri, Merelbeke, Belgium; ^3^Biometrics Research Group, Department of Veterinary and Biosciences, Faculty of, Merelbeke, Belgium; ^4^Department of Clinical Chemistry, Microbiology and Immunology, Faculty of Medici, Ghent, Belgium

Age‐related changes in renal function occur in people and dogs. However, combined measurement of functional, glomerular and tubular markers in aging dogs is scarce. Prospective, longitudinal study. Renal function was evaluated in apparently healthy senior and geriatric dogs at baseline (T0), 12 months (T12) and 24 months (T24), using validated serum (creatinine, symmetric dimethylarginine [SDMA], cystatin C [sCysC]) and urinary (specific gravity [USG], protein:creatinine [UPC], albumin:creatinine [uALBcr], retinol binding protein:creatinine [uRBPcr]) biomarkers. Glomerular filtration rate (GFR) was measured in a subgroup of dogs. At baseline, 122 dogs were included; follow‐up was available in 107 (T12) respectively 92 (T24) dogs; and GFR was estimated in 18 (T0), 11 (T12) and 10 (T24) dogs. Throughout the study, 25 dogs showed evidence of non‐azotemic chronic kidney disease (CKD), of which the majority (20/25) had persistent overt proteinuria. Three and 7 dogs developed CKD IRIS stage 2 at T12 and T24, respectively. Bodyweight showed a significant (*p* < 0.05) positive correlation with UPC (T24 (*ρ*=.36)) and uALBcr (T0 (*ρ*=.21), T12 (*ρ*=.48)). Age was significantly (*p*<0.05) moderately (*ρ*=.21–.38) positively correlated with both SDMA and sCysC at each timepoint, but not with creatinine. A significant (*p*<0.001) decreasing trend was observed for sCysC at T12, and SDMA at T24 compared to baseline, respectively. No other significant changes over time were observed. Almost 1/3 of older dogs developed CKD, mostly persistent renal proteinuria, over 2‐years follow‐up. Bodyweight and hence size could influence proteinuria and albuminuria. Age may affect SDMA and sCysC, emphasizing the importance of age‐specific reference intervals for each biomarker.

**Disclosures**

For the study described in the abstract material support was received.

**Source of Funding**

Ceva Belgium and Hills Pet Nutrition.

**ECVIM CSF Funding**

No.

**ESCG** – **European Society of Comparative Gastroenterology**

## ESCG‐O‐1

## Ursodiol Administration Modulates the Intestinal Ecosystem of Dogs and Cats

### A. Winston^1,2^, H. Klein^3,4^, A. Wood^3,4^, V. Parker^3,4^, A. Rudinsky^3,4^

#### ^1^The Ohio State University College of Veterinary Medicine The Ohio State University CVM Dept Vet Clinical Sci, Columbus, United States; ^2^Comparative Hepatobiliary Intestinal Research Program (CHIRP) The Ohio State University CVM Dept Vet Clinical Sci, Columbus, United States; ^3^The Ohio State University College of Veterinary Medicine, Columbus, United States; ^4^Comparative Hepatobiliary Intestinal Research Program (CHIRP), Columbus, United States

Ursodeoxycholic acid (UDCA, Ursodiol) is a naturally occurring bile acid that is used to treat a variety of hepatic and gastrointestinal diseases. Ursodiol has a multitude of beneficial therapeutic effects and can modulate bile acid pools. Bile acids can alter the gut microbiota community structure through various mechanisms. In turn, the gut microbial community can modulate bile acid pools, thus highlighting the interconnectedness of the gut microbiota‐bile acid‐host axis. Despite these interactions and its widespread use in veterinary medicine, it remains unclear if and how exogenously administered Ursodiol shapes the gut microbial community structure and fecal bile acid metabolome in dogs and cats. This study aimed to provide a comprehensive analysis of how Ursodiol alters the canine and feline intestinal ecosystem. In this study, 15 client‐owned dogs and 6 purpose‐bred research cats received 10–15 mg/kg Ursodiol orally once daily for 21 days. Naturally voided feces was collected prior to Ursodiol (baseline), at day 7, 14, 21 during treatment, and one week, 1 month, and 3 months post Ursodiol. Feces was stored at −80°C. Fecal targeted bile acid metabolomics was performed by UPLC‐MS/MS. DNA was extracted and shotgun metagenomic sequencing was performed. Metagenome‐assembled genomes (MAGs) were constructed and taxonomy assigned using the GTDB with metagenomic taxonomic profiling using MetaPhlAn4. Alterations in the fecal microbial community structures were seen in dogs and cats during Ursodiol treatment. Ursodiol administration significantly increased fecal UDCA concentrations by day 7 in dogs and by day 21 in cats (Freidman test with Dunn's multiple comparisons; all timepoints *P* < 0.05). Aside from UDCA, Ursodiol treatment altered the canine and feline fecal bile acid metabolome, including increases in the secondary bile acid murocholic acid. Ursodiol treatment also significantly altered the newly described amino acid microbial conjugated bile acids (MCBAs) in dogs, specifically histidine‐UDCA, phenylalanine‐UDCA, tyrosine‐UDCA, tryptophan‐UDCA, and methionine‐UDCA (Wilcoxon matched‐pairs signed rank test; all *P* < 0.0001). Fecal MCABs were not significantly different in cats following Ursodiol treatment. In conclusion, this study is the first to utilize a multi‐omics approach to provide a comprehensive profile of how exogenously administered Ursodiol shapes the intestinal ecosystem in dogs and cats. Further studies to investigate how these changes in turn modify the host physiologic response are required. With the widespread use of Ursodiol in veterinary medicine, ramifications of how this drug impacts the gut microbiota‐bile acid‐host axis in dogs and cats during health and disease states is warranted.

**Disclosures**

BextPetRx provided the Ursodiol used for the canine patients in this study.

**Source of Funding**

Ohio State University College of Veterinary Medicine Intramural Canine Fund; EveryCat Health Foundation.

**ECVIM CSF Funding**

No.

**ESCG**—**European Society of Comparative Gastroenterology**

## ESCG‐O‐2

## Effect of metronidazole on fecal microbiome, metabolome, and serum uremic toxins in healthy adult cats

### S. Belchik^1^, T. Schmidt^2^, A. Blake^2^, J. Cavasin^2^, J. Suchodolski^2^, K. Swanson^3^

#### ^1^University of Illinois Urbana‐Champaign Animal Sciences, Champaign, United States; ^2^Texas A&M University Small Animal Clinical Sciences, College Station, United States; ^3^University of Illinois Urbana‐Champaign Animal Sciences, Nutritional Sciences, Veterinary Clinical Medicine, Champaign, United States

Antibiotics are commonly used to aid in the remission of gastrointestinal diseases, but usage may lead to prolonged dysbiosis. In dogs, metronidazole administration reduced microbial diversity and induced prolonged functional changes in microbial function (i.e., altered bile acid (BA) profiles). Limited information is available about the effects of metronidazole on short‐chain fatty acids (SCFA) and uremic toxins in cats.

The objective of this study was to evaluate the long‐term effects of metronidazole on microbial function in healthy adult cats. Fecal and serum samples from twelve healthy cats as part of a previously conducted trial were assessed. Cats consuming the same basal diet received metronidazole (20 mg/kg BW PO q12hrs) for day 0–14, then were monitored for a follow‐up period (day 15–42). Fecal and blood samples were collected at baseline (day 0), end of metronidazole treatment (day 14), and weekly during the follow‐up period (day 21, 28, 35, and 42). Fecal samples were used for microbiome analysis using the feline dysbiosis index and 16S rRNA gene sequencing, measurement of pH, dry matter, and metabolite concentrations (SCFA, BA) using gas chromatography and mass spectrometry (GCMS), and serum samples were analyzed for the uremic toxins, p‐cresol and indoxyl‐sulfate, using LCMS.

Metronidazole significantly increased (*p* < 0.0001) dysbiosis index, and decreased SCFA and secondary BA concentrations in fecal samples, with some of these persistent over the observation period of 4 weeks. Prolonged dysbiosis and *Clostridium hiranonis* reductions were observed in 10/12 (83%) cats. Uremic toxins were strongly reduced (*p* < 0.0001) after metronidazole administration and did recover to near‐baseline measures by the end of the study. SCFA concentrations tended to increase (*p* = 0.0658) during the 4 week recovery period, but did not achieve full recovery to baseline measures.

The observed decline after metronidazole administration may be helpful in understanding microbial metabolism and its relation to disease progression. In conclusion, metronidazole is a potent bactericidal antibiotic with strong effects observed in the microbiome and metabolome with persistent effects on microbial function, even after one month of recovery.

**Disclosures**

JSS, TS, JC, ABB are employees of the Gastrointestinal Laboratory at Texas A&M which offers gastrointestinal functional testing on a fee‐for‐service basis.

**Source of Funding**

**ECVIM CSF Funding**

No.

**ESCG** – **European Society of Comparative Gastroenterology**

## ESCG‐O‐3

## Limited ingredient diets remodel the enteric microbiome and serum metabolome of dogs with food responsive enteropathy

### K. Simpson^1^, X. Morgan^2^, M. Miller^1^, J. Loftus^1^, C. Frederick^3^, J. Wakshlag^3^, S. Zhang^3^, M. Rishniw^3^

#### ^1^Cornell University Clinical Sciences, Ithaca, United States; ^2^Harvard TH Chan School of Public Health Harvard Chan Microbiome Analysis Core, Boston, United States; ^3^Cornell University, Ithaca, United States

The ability of diet to induce clinical remission in a subset of dogs with chronic enteropathy is well established. The complex interactions between diet, the microbiome, and enteric and systemic metabolism that underpin the development and remission of food‐responsive enteropathies are unresolved. We sought to determine the ability of ingredient restricted diets composed of hydrolyzed fish, rice starch and fish oil without (HF) or with prebiotics, turmeric and high cobalamin (HF+) and a diet containing mixed non‐hydrolyzed antigens and oils (non‐H) to influence the enteric microbiome and serum metabolome of dogs with food‐responsive enteropathies. Twenty‐five dogs with food‐responsive enteropathies achieved clinical remission (CCECAI ≤ 3 by 6 wks) when fed HF (11), HF+ (8) and non‐H (6) in a randomized prospective 12‐week trial. The fecal microbiomes and serum metabolomes of food‐responsive enteropathies (0, 6, 12 weeks after test diet) and 16 healthy control dogs were evaluated by metagenomic sequencing and untargeted metabolomics, respectively. We examined the effects of diet and disease status on microbiome ecology (Bray–Curtis distance), and quantified which serum metabolites, microbial species, and microbial enzymes (EC) were associated with diet and phenotype (Maaslin2). Dogs with food‐responsive enteropathies had more heterogenous microbiomes than controls, but became more similar to controls at 6 and 12 wks, corresponding with clinical remission. The food‐responsive enteropathies microbiome changed more in the first half of the study than the second half. At baseline, 5 species were depleted (all Firmicutes) and *E. coli* was enriched in food‐responsive enteropathies compared to controls. Forty‐three species were associated with remission: 34 increased (e.g. *Fusobacterium*, *Blautia*, *Peptacetobacter*) and 9 decreased (e.g. *R. gnavus*, *Enterococcus s*pp, *C. perfringens*). There were 519 ECs associated with food‐responsive enteropathies week 0 versus control, and 220 remission‐associated microbial enzymes that broadly shifted towards control. Dogs with food‐responsive enteropathies had more heterogeneous serum metabolomes at baseline than controls, which became less heterogeneous over time. Differences in lipids, amino acids, bile acids and carnitine were major contributors to disease and remission. The HF diet appeared to have the most consistent effect on the microbiome as a whole and with remission‐associated species.This study reveals substantial dysbiosis and dysmetabolism in dogs with untreated food‐responsive enteropathies, and the ability of ingredient‐restricted diets that induce clinical remission to remodel the fecal microbiome and serum metabolome of dogs with food‐responsive enteropathies.

**Disclosures**

Kenneth Simpson: Nestle Purina Advisory Council Farmina Pet Foods:Scientific Advisory Group Bactana : Senior Scientific Advisor The Kerry Health and Nutrition Institute® (KHNI) Scientific Advisory Council Grant support: Farmina Pet Foods, Intralytix, AKC‐CHF, Biostime Institute Nutrition Care Joseph Wakslag: Consultant for Ellevet Sciences, The Farmer's Dog and Annamaet Petfoods. Honorariums from Blue Buffalo, Mars and Purina. John Loftus: Vaika Foundation, Scientific Advisory Board Blue Buffalo, Veterinary Advisory Board Universal Biosensors, Veterinary Advisory Panel, Dog Watch, Advisory Board Grant support: Okava Pharmaceuticals, The Farmer's Dog, ElleVet Sciences

**Source of Funding**

Farmina Pet Foods

**ECVIM CSF Funding**

No

**ESCG** – **European Society of Comparative Gastroenterology**

## ESCG‐O‐4

## Fecal bile acid dysmetabolism is correlated with relative abundance of peptacetobacter hiranonis and oscillospirales in cats with chronic kidney disease

### J. Rowe^1,2^, S. Summers^3^, J. Quimby^1,2^, J. Winston^1,2^

#### ^1^The Ohio State University College of Veterinary Medicine Veterinary Clinical Sciences, Columbus, United States; ^2^Comparative Hepatobiliary Intestinal Research Program (CHIRP) Veterinary Clinical Sciences, Columbus, United States; ^3^Oregon State University Carlson College of Veterinary Medicine Department of Clinical Sciences, Corvallis, United States

Primary bile acids (PBAs) secreted by the host are transformed into secondary bile acids (SBAs) by gut microbiota. SBAs are reabsorbed and sensed via host receptors, modulating cellular inflammation and fibrosis. Feline chronic kidney disease (CKD) occurs with progressive renal inflammation and fibrosis. This study aimed to determine if SBA metabolism is altered in feline CKD, and if so, determine feline gut microbiota members corresponding with dysmetabolism. This prospective cross‐sectional study compared healthy cats (*n* = 6) with CKD (IRIS Stage 2 *n* = 17, IRIS Stage 3 or 4 *n* = 11). Single timepoint fecal samples from all cats underwent liquid chromatography and tandem mass spectrometry targeted metabolomics to determine concentrations of 15 PBAs and SBAs. 16S rRNA gene amplicon sequencing, generating amplicon sequence variants (ASVs) using DADA2 with SILVA taxonomy, characterized the fecal microbiota. NCBI nucleotide BLAST was used to further investigate taxonomy of 16S rRNA gene sequences. CKD cats had significantly reduced fecal concentrations (median 12.8 ng/mg, Mann–Whitney *p* = 0.0127) of the SBA ursodeoxycholic acid (UDCA) compared to healthy cats (median 39.4 ng/mg). Bile acid dysmetabolism characterized by <50% SBAs was present in 8/28 CKD and 0/6 healthy cats. Beta diversity significantly differed between cats with <50% SBAs and >50% SBAs (Bray–Curtis PERMANOVA *p* < 0.0001). 26 ASVs with >97% nucleotide identity to *Peptacetobacter hiranonis* 16S rRNA gene sequence were identified. *P. hiranonis* combined relative abundance was significantly reduced (median 2.1%) in CKD cats with <50% SBAs compared to CKD cats with >50% SBAs (median 13.9%, Kruskal–Wallis FDR adjusted *p* = 0.0002) and healthy cats with >50% SBAs (median 15.5%, Kruskal–Wallis FDR adjusted *p* = 0.0112). *P. hiranonis* combined relative abundance was significantly positively correlated with the SBAs deoxycholic acid (Spearman *r* = 0.5218, FDR adjusted *p* = 0.0407) and lithocholic acid (Spearman *r* = 0.5615, FDR adjusted *p* = 0.0156). Three *Oscillospirales* ASVs were identified as significantly negatively correlated with fecal PBA concentrations of cholic acid, chenodeoxycholic acid, and glycocholic acid. Fecal bile acid dysmetabolism characterized by <50% SBAs occurs in CKD cats in addition to reduced UDCA. The dysmetabolism is partially explained by reduced abundance of *P. hiranonis*, but other microbiota community members including *Oscillospirales* likely play a role. Metagenomic characterization of *Oscillospirales* has recently suggested a role in bile acid metabolism in people, making application of metagenomics an important next step to characterize feline microbial bile acid metabolism.

**Disclosures**

JCR has received prior travel support from Royal Canin. SCS is a research consultant for IDEXX Laboratories, Inc. and has previous work funded by Nestle Purina and IDEXX Laboratories, Inc. She has received a speaker honorarium from Royal Canin, IDEXX Laboratories, Inc., and Boehringer‐Ingelheim. JMQ's work has been funded by EveryCat Health Foundation, Morris Animal Foundation, Nestle Purina, Trivium Vet, Zoetis. She has received compensation as a member of the scientific advisory board of Nestle Purina, Elanco, Zoetis. She also has consulted or served as a key opinion leader for Boehringer Ingelheim, Dechra, Elanco, Gallant, Heska, Hill's, IDEXX, Nestle Purina, Royal Canin, SN Biomedical, Vetoquinol, Zoetis and received compensation. JAW's laboratory has been funded by EveryCat Health Foundation, Morris Animal Foundation, American Kennel Club's Canine Health Foundation, Nestle Purina, FDA, and National Institutes of Health. She has received speaker honorariums from Royal Canin, Nestle Purina, and DVM360.

**Source of Funding**

Buttons Fund For Feline Chronic Kidney Disease Research; University of Colorado Cancer Center Shared Resource Support Grant, Grant/Award Number: P30CA046934.

**ECVIM CSF Funding**

No.

**ESCG** – **European Society of Comparative Gastroenterology**

## ESCG‐O‐5

## Multi‐omic approach identifies expanded bile acid pools, new microbially conjugated bile acids, and bile acid biotransformation genes in the canine and feline gut microbiota

### A. Winston^1,2^, H. Klein^3,4^, A. Wood^3,4^, V. Parker^3,4^, A. Rudinsky^3,4^

#### ^1^The Ohio State University College of Veterinary Medicine The Ohio State University CVM Dept Vet Clinical Sci, Columbus, United States; ^2^Comparative Hepatobiliary Intestinal Research Program (CHIRP) The Ohio State University CVM Dept Vet Clinical Sci, Columbus, United States; ^3^The Ohio State University College of Veterinary Medicine, Columbus, United States; ^4^Comparative Hepatobiliary Intestinal Research Program (CHIRP), Columbus, United States

Bile acids (BAs) are dynamic signaling molecules capable of impacting host physiology. Within the gut, the microbiota metabolizes host‐derived primary BAs (PBAs) into chemically distinct microbial‐derived secondary BAs (SBAs). Bile acid biotransformations are encoded by microbial genes including for bile salt hydrolase (BSH) and bile acid‐inducible (*bai*) genes for the multi‐step 7α‐dehydroxylation reaction. In companion animals, characterization of microbial‐derived BAs in health and disease is rapidly expanding, but to date a limited number of BAs have been documented and BA biotransformation genes have yet to be described. This study aimed to define the complexity and diversity of fecal BA pools and BA biotransformation genes in companion animals, including to evaluate for the presence of newly described amino acid microbially conjugated BAs (MCBAs). In this study, naturally voided feces from 15 client‐owned dogs and 6 purpose‐bred research cats were collected and stored at −80°C. Fecal targeted BA metabolomics was performed by UPLC‐MS/MS. DNA was extracted and shotgun metagenomic sequencing was performed. Metagenome‐assembled genomes (MAGs) were constructed and taxonomy assigned using the GTDB with metagenomic taxonomic profiling using MetaPhlAn4. Comprehensive metagenomic analysis of BA biotransformation gene abundances was performed. In healthy dogs, a total of 61 fecal BAs were detected, including 3 unconjugated PBAs, 4 conjugated PBAs, 21 unconjugated SBAs, 7 conjugated SBAs, and 26 MCBAs. In healthy cats, a total of 51 fecal BAs were detected, including 3 unconjugated PBAs, 4 conjugated PBAs, 19 unconjugated SBAs, 5 conjugated SBAs, and 20 MCBAs. In both dogs and cats, these newly described MCBAs included BA conjugates with alanine, serine, leucine, threonine, lysine, methionine, histidine, phenylalanine, tyrosine, arginine, and tryptophan. In screening MAGs for BA transformation genes, we identified 14 genes (BSH, BaiA1, BaiA2, BaiB, BaiCD, BaiE, BaiF, BaiG, BaiH, BaiI, BaiJ, BaiK, BaiL, HDHA) across various species including from the family *Oscillospiraceae* and *Peptacetobacter spp*. This study provides a comprehensive overview of the diversity of *bai*‐harboring bacteria in the canine and feline gut highlighting the application of multi‐omics approaches. These results expand the scope of BA pool diversity described in dogs and cats, including the first documentation of α‐muricholic acids in companion animals. Additionally, these results are the first to document microbial BA transformation genes and the gut microbiota's capability to produce MCBAs in dogs and cats. This study opens a new perspective to investigate collaborative metabolism of BAs between companion animal and gut microbes in health and disease.

**Disclosures**

**Source of Funding**

The Ohio State University College of Veterinary Medicine Intramural Canine Grant; EveryCat Health Foundation.

**ECVIM CSF Funding**

No.

**ESCG**—**European Society of Comparative Gastroenterology**

## ESCG‐O‐6

## Recovery of fecal fatty acid and sterol profiles in dogs with acute hemorrhagic diarrhea syndrome

### K. Busch^1^, T. Schmidt^2^, W. Huang^3^, A. Reisinger^4^, J. Cavasin^3^, C.C. Chen^3^, S. Unterer^5^, J. Suchodolski^3^

#### ^1^Ludwig‐Maximilians University Small Animal Clinic, Munich, Germany; ^2^Gastrointestinal Laboratory, Department of Small Animal Clinical Sciences, Texas Department of Small Animal Clinical Sciences, Texas, College Station, TX, United States; ^3^Gastrointestinal Laboratory, Department of Small Animal Clinical Sciences, Texas, College Station, TX, United States; ^4^Ludwig‐Maximilians University Small Animal Clinic, Munich, Germany; ^5^Clinic for Small Animal Internal Medicine, Vetsuisse Faculty, Zurich, Switzerland

Changes in the fecal lipid‐related metabolites can be an indicator of intestinal damage. In dogs with chronic enteropathies, some markers are persistently altered in a subset of dogs. Acute hemorrhagic diarrhea syndrome (AHDS) is a clinically rapidly self‐limiting disease that is characterized by severe mucosal necrosis. The objective of this study was to evaluate the fecal lipid profile of AHDS dogs over time to assess the recovery of these markers of gastrointestinal mucosal damage.

Fecal samples from 29 dogs with AHDS from a previous clinical trial and 29 fecal samples from healthy control dogs (HC) were analyzed. Samples from the AHDS group were collected at five different time points (day 1 [*n* = 22], day 2 [*n* = 15], day 7 [*n* = 21], day 21 [*n* = 22], day 42 [*n* = 23]). Lipid profiles of all samples were characterized by quantifying 27 metabolites using gas chromatography‐mass spectrometry. Clinical disease severity was assessed by using the AHDS index. Statistical analysis was performed by generalized linear mixed models.

The clinical AHDS index significantly decreased within the first seven days (*p* < 0.0001). Strong alterations in the fecal fat‐related metabolites of dogs with AHDS at initial clinical presentation (day 1 and 2) were observed, followed by a rapid recovery (days 7–42). Principal component analysis revealed clustering of the lipid profiles of dogs with AHDS in samples collected on day 1 and 2 away from HC. Samples collected on day 7 or later clustered with the HC lipid profile. At days 1 and 2, fecal metabolite concentrations of arachidonic acid (*p* = 0.0003), nervonic acid (*p* < 0.0001), and cholesterol (*p* < 0.0001) were significantly increased, but decreased significantly and were closer to the HC group at days 7‐42. Total bile acid concentrations (*p* = 0.9298) and percentage of secondary bile acids (*p* = 0.8507) did not change over the first seven days in the AHDS group and was not significantly different from HC.

Results indicate highly abnormal fecal sterols and fatty acid profiles in dogs with acute enteropathy, likely reflecting extensive mucosal necrosis caused by AHDS. These changes were followed by a rapid normalization of metabolites concurrent with clinical improvement (AHDS index). This normalization contrasts with the metabolomic shift that occurs in chronic enteropathy, which persist even after clinical remission in a subset of patients.

**Disclosures**

Statement of Disclosures: JSS, JPC, CCC, WH and TS are employees of the Gastrointestinal Laboratory at Texas A&M which offers gastrointestinal functional testing on a fee‐for‐service basis.

**Source of Funding**

1 Clinic of Small Animal Internal Medicine, Centre for Clinical Veterinary Medicine, Ludwig‐Maximilians‐University, Munich, Germany 2 Gastrointestinal Laboratory, Department of Small Animal Clinical Sciences, Texas A&M University, College Station, TX, USA.

**ECVIM CSF Funding**

No.

**ESCG** – **European Society of Comparative Gastroenterology**

## ESCG‐O‐7

## Metagenomic sequencing identifies specific gut microbes associated with resolution of canine acute noninfectious colitis

### J. Rowe^1,2^, A. Rudinsky^1,2^, S. Perea^3^, V. Parker^1,2^, J. Winston^1,2^

#### ^1^The Ohio State University College of Veterinary Medicine Veterinary Clinical Sciences, Columbus, United States; ^2^Comparative Hepatobiliary Intestinal Research Program (CHIRP) Veterinary Clinical Sciences, Columbus, United States; ^3^Royal Canin Global Research and Development, Aimargues, France

A recent randomized controlled trial demonstrated superiority of dietary management alone compared to metronidazole for empiric management of canine acute noninfectious colitis. The restoration of beneficial gut microbiota through dietary management versus deleterious microbiota disturbances induced by metronidazole is a possible driver of the clinical findings. The objective of this study was to evaluate the fecal microbiota from the previously described clinical trial cohort. Dogs with acute noninfectious colitis (*n* = 59), were randomized to one of three groups: 1) easily digestible diet with placebo tablet (*n* = 19), 2) easily digestible diet with metronidazole tablet (*n* = 20), or 3) psyllium‐enhanced easily digestible diet with placebo tablet (*n* = 20). Naturally voided feces were collected and stored at −80°C at time of study enrollment, 7–10 days, and 30 days. DNA was extracted and shotgun metagenomic sequencing (target 50 million reads per sample) was performed. Metagenome‐assembled genomes (MAGs) were constructed and taxonomy assigned using the GTDB. Individual species‐level reads mapping back to MAGs from a Metaphlan4 output were analyzed with phyloseq in R Studio. Dual application of ALDEx2 and ANCOMBC2 determined differentially abundant species. At time of study enrollment, there were no significant differences in fecal microbiota alpha or beta diversity measures between groups. By days 7–10, the metronidazole group alpha diversity was significantly reduced (Kruskal‐Wallis FDR adj. *P* < 0.05 for all metrics). The metronidazole group beta diversity assessed with Bray–Curtis distances was also significantly different at days 7–10 (Pairwise PERMANOVA *P* < 0.001 compared to both other groups), while easily digestible diet and psyllium‐enhanced easily digestible diet groups did not significantly differ (Pairwise PERMANONA *P* = 0.083). At days 7–10, eight bacterial species significantly differed in relative abundance between groups: *Peptacetobacter hiranonis*, *Enterococcus faecium*, *Phocaeicola plebius*, a species within the family *Peptostreptococcaceae*, a species within the family *Oscillospiraceae*, a species within the phylum Fusobacteria, and two species within the phylum Firmicutes. By day 30, there were no differences in alpha diversity between groups, though the beta diversity between the metronidazole group and psyllium‐enhanced easily digestible diet group remained significantly different (Pairwise PERMANOVA *P* = 0.014). Future functional genetic analysis of microbial species in conjunction with metabolomics will provide mechanistic microbiome insights involved in the resolution of canine acute noninfectious colitis. Such microbial insights could identify novel members of the canine gut microbiota that mediate metabolite production important to recovery of eubiosis following states of dysbiosis.

**Disclosures**

Drs. Rudinsky, Parker, and Winston have received honoraria or are consultants for Royal Canin. Dr. Perea is an employee of Royal Canin. Dr. Rowe has received prior travel support from Royal Canin.

**Source of Funding**

This study received funding from Royal Canin.

**ECVIM CSF Funding**

No.

**ESCG** – **European Society of Comparative Gastroenterology**

## ESCG‐O‐8

## Functional gene profile and targeted metabolome in dogs with chronic enteropathy

### C.C. Chen^1^, C.H. Sung^1^, R. Pilla^1^, L. Toresson^2,3^, A. Jergens^4^, S. Summers^5^, P. Giaretta^1^, M.K. Tolbert^1^, J. Suchodolski^1^

#### ^1^Gastrointestinal Laboratory, Texas A&M University Department of Small Animal Clinical Sciences, College Station, United States; ^2^IVC Evidensia Specialist Animal Hospital Helsingborg, Helsingborg, Sweden; ^3^Faculty of Veterinary Medicine, Helsinki University, Helsingborg, Finland; ^4^Iowa State University Department of Veterinary Clinical Sciences, Ames, United States; ^5^Oregon State University Department of Clinical Sciences, Corvallis, United States

Intestinal dysbiosis has been associated with canine chronic enteropathy (CE); however, not all dogs have an increased dysbiosis index (DI). In addition, changes in fecal metabolome such as fatty acids, sterols, bile acids, and sugars have been reported in a subset of dogs with CE, suggesting host intestinal and microbial functionalities’ disturbances might play a role in CE. Previous studies have evaluated the taxonomic composition of the fecal microbiome in dogs with CE but have not investigated microbial functional gene profiles. In this study, we aimed to compare the functional gene profiles and selected fecal host and microbial metabolites between healthy control dogs (HC), dogs with CE with an abnormal DI (ADI‐CE), and dogs with CE with a normal DI (NDI‐CE).

This was a retrospective cross‐sectional study including 78 HC and 138 dogs with CE. Fecal functional gene profiles were assessed through shallow shotgun metagenomic sequencing. DI was quantified by targeted quantitative PCR. The targeted fecal metabolites including fatty acids, sterols, bile acids (BAs), and monosaccharides were assessed using gas chromatography–mass spectrometry in a subset of the study population, including 22 HC and 50 dogs with CE. ANOSIM, Maaslin2, and Kruskal–Wallis tests were used to compare functional gene profiles and fecal metabolome between groups. The correlations between DI and individual metabolites were evaluated by Spearman's correlation tests.

Based on the Bray–Curtis distance of the overall functional composition, significant differences were found in ADI‐CE when compared to NDI‐CE and HC (*p* < 0.01 for both). At the functional model level, when compared to HC, functional gene sets had greater shifts in ADI‐CE than NDI‐CE (*p* < 0.01, *p* = 0.02, respectively). Membrane transport‐related genes were upregulated, while amino acid metabolism‐related genes were downregulated in ADI‐CE, but not NDI‐CE (*p* < 0.01 for both). Similarly, fecal concentrations of unconjugated primary BAs were increased, while unconjugated secondary BAs and coprostanol were decreased in ADI‐CE when compared to other groups (*p* < 0.03 for all). As for the correlation analysis, positive correlations between DI with fecal concentrations of unconjugated primary BAs and with glucose were found (*p* < 0.01 for all). In contrast, negative correlations were observed between DI and fecal concentrations of unconjugated secondary BAs, coprostanol, and cholestenol (*p* < 0.01).

Results indicate differences in microbial gene profiles and fecal metabolome between dogs with CE and abnormal DI, compared to dogs with CE and normal DI and healthy control dogs, suggesting a different pathophysiology between the two CE groups.

**Disclosures**

The authors (Chih‐Chun Chen, Chi‐Hsuan Sung, Rachel Pilla, Paula Giaretta, M. Katherine Tolbert, Jan Suchodolski) of this abstract are employed by the gastrointestinal laboratory at Texas A&M University, which provides assays for intestinal functions and microbiota analysis on a fee‐for‐service basis. The microbiome research is supported in part by the Purina PetCare Research Excellence Fund.

**Source of Funding**

**ECVIM CSF Funding**

No.

**ESCG** – **European Society of Comparative Gastroenterology**

## ESCG‐O‐9

## Disruptions in microbial tryptophan catabolism are associated with fecal abundance of Bifidobacterium *and disease activity indices in cats with chronic enteropathies*

### P. Barko^1^, S. Marsilio^2^, J. Suchodolski^3^, A. Gal^1^, D. Williams^1^

#### ^1^University of Illinois at Urbana‐Champaign Veterinary Clinical Medicine, Urbana, United States; ^2^UC Davis School of Veterinary Medicine, Davis, United States; ^3^Texas A&M University Gastrointestinal Laboratory, College Station, United States

Microbial indole catabolites of tryptophan (MICT) are gut microbial metabolites implicated in modulating intestinal mucosal inflammation and strengthening the mucosal barrier. A previous study identified decreased MICTs, including indolepropionate, indolealdehyde, indolelactate, and indoleacrylate, in the sera of cats with chronic inflammatory enteropathy (CIE) and low‐grade intestinal T‐cell lymphoma (LGITL). We hypothesized that serum and fecal MICT concentrations would be correlated with the abundance of commensal gut microbiota and disease severity index in cats with CIE and LGITL. In this cross‐sectional, observational study, surplus serum and fecal samples from cats with CIE (*n* = 11), LGITL (*n* = 12), and healthy controls (*n* = 36) were utilized. Fecal and serum MICTs were analyzed using LCMS, fecal microbiomes were characterized using full‐length 16S‐rRNA amplicon sequencing, and the feline chronic enteropathy activity index (FCEAI) was used to assess disease activity. Differential abundance of microbiota was assessed with a negative binomial model, and the Spearman correlation coefficient (rho) was used to detect associations among MICTs, microbiota, and FCEAI. Results were corrected for multiple testing (q‐value) to minimize the risk of false discovery. Compared with healthy controls, relative abundances of *Bifidobacterium longum* and *Bifidobacteriumadolescentis* were significantly decreased in the feces of cats in the CIE and LGITL groups. Fecal tryptophan concentrations were significantly increased in the CIE (*q*=0.003) and LGITL (*q*=0.016) groups compared with healthy controls. Fecal concentrations of indoleacrylate (rho=0.54; *q*=0.001) and indolelactate (rho=0.56; *q*<0.001) were positively correlated with the relative abundance of *B. longum* while fecal indoleacrylate (rho=0.54; *q*<0.001) and indolelactate (rho=0.47; *q*=0.003) were positively correlated with the relative abundance of *B. adolescentis*. FCEAI was inversely correlated with serum concentrations of indolepropionate (rho=‐0.3; *q*=0.001), indolelactate (rho=‐0.42; q=0.003), indoleacrylate (rho=−0.47; −=0.002), and indolealdehyde (rho=‐0.46; *q*=0.003) and positively correlated with fecal concentrations of tryptophan (rho=0.4; *q*=0.003). These findings suggest that pathophysiologic consequences of enteric microbiota dysbiosis in CIE and LGITL could be mediated by lower abundance of *Bifidobacterium spp*., decreased production of MICTs, and decreased intestinal tryptophan absorption. Associations with FCEAI suggest MICTs may be promising biomarkers to further assess the influence of enteric microbiota in feline chronic enteropathies. These results emphasize the complex interplay between enteric microbiota, nutrient metabolism, and intestinal health in cats.

**Disclosures**

Patrick Barko has current research funding from American Kennel Club Charitable Health Foundation, EveryCat Health Foundation, Nestle‐Purina PetCare, and EPI4Dogs.com Foundation Inc. Sina Marsilio is a consultant for Dutch, has been a paid speaker for IDEXX and her laboratory has received funding from Inaba Foods. Jan Suchodolski is an employee of the Gastrointestinal Laboratory at Texas A&M University which offers microbiome testing on a fee‐for‐service basis. He is the Purina PetCare Endowed Chair for Microbiome Research and his work is supported in part through the Purina PetCare Research Excellence Fund. Consulting and speaking fees: Purina Petcare, Royal Canin, IDEXX Laboratories, Hill's, Nutramax Laboratories, ExeGI Pharma Arnon Gal is a consultant for TAMU GI Lab, BodiTech, Boehringer Ingelheim, and Epivara. David Williams currently receives, or recently received, financial support from Morris Animal Foundation, American Kennel Club Charitable Health Foundation, EveryCat Health Foundation, Nestle‐Purina PetCare, and EPI4Dogs.com Foundation Inc. I am also currently a consultant with Idexx Laboratories, Nestle‐Purina PetCare, and the GI Laboratory at Texas A&M University.

**Source of Funding**

This investigation was funded, in part, by a grant from the EveryCat Health Foundation Miller Trust.

**ECVIM CSF Funding**

No.

**ESCG – European Society of Comparative Gastroenterology**

## ESCG‐O‐10

## Prediction of mortality and intensive care unit length of stay in dogs with acute pancreatitis using machine learning algorithms: A pilot study on 215 dogs

### C. Benoit^1^, P. Remmel^1^, P. Verwaerde^2^, G. Benchekroun^1^, A.M. Canonne^1^

#### ^1^Ecole nationale vétérinaire d'Alfort, Univ Paris Est Créteil Unité de Médecine Interne, Maisons‐Alfort, France; ^2^Ecole nationale vétérinaire d'Alfort, Univ Paris Est Créteil Pôle Anesthésie Réanimation Soins Intensifs, Maisons‐Alfort, France

Acute pancreatitis (AP) in dogs is a frequent and life‐threatening condition. Only few prognostic factors have yet been identified with strong evidence. In human medicine, some machine learning algorithm can robustly predict the prognosis of patients with AP. This study aims at proposing machine learning models designed to predict, at admission, the occurrence of death during hospitalization (DH), and death during hospitalization or intensive care unit length of stay greater than or equal to 3 days (DH/SH).

Forty‐six clinical and clinicopathological variables measured at time of admission were retrospectively collected from dogs diagnosed with AP and hospitalized in a teaching hospital from January 2015 to May 2023. A radial kernel support vector machine algorithm was trained on 80% of the study population and tested on the remaining 20% of dogs.

A total of 215 dogs were included. The 46‐parameter model (SVM 46) could predict DH with area under the receiver‐operating characteristic curve (AUC) of 0.80 and DH/SH with AUC of 0.76. For the prediction of DH, a second model (SVM 10_D) including blood chloride, potassium, sodium, lactate, urea, creatinine, alanine aminotransferase (ALT), lymphocyte count, platelet count and rectal temperature was proposed. It was associated with an AUC of 0.84 (sensitivity of 0.86 and specificity of 0.86). For the prediction of DH/SH, a second model (SVM 10_S) including blood lactate, venous partial pressure of CO_2_, creatinine, ALT, total bilirubin, glycemia, concentration of canine‐specific pancreatic lipase (cPLi)®, white blood cell count, duration of clinical signs and rectal temperature was proposed. It was associated with an AUC of 0.78 (sensitivity of 0.90 and specificity of 0.75). The proposed models outperformed creatinine, concentration of cPLi®, C‐reactive protein (CRP) and blood lactate at admission for predicting DH (AUCs of 0.64, 0.55, 0.62 and 0.46, respectively) and DH/SH (AUCs of 0.51, 0.62, 0.65 and 0.59, respectively). SVM 10 AUCs were higher than creatinine AUCs, significantly (*p* = 0.017) to predict DH and non‐significantly (*p* = 0.089) to predict DH/SH.

This is the first study to evaluate machine learning in predicting the prognosis of AP in dogs. Proposed models showed promising performance in predicting mortality and/or a hospital stay of more than 3 days in an intensive care unit. A multicentre validation study is now needed to confirm their prognostic value on a larger scale.

**Disclosures**

**Source of Funding**

**ECVIM CSF Funding**

No.

**ESCG – European Society of Comparative Gastroenterology**

## ESCG‐O‐11

## Re‐evaluation of the Reference Interval and Cut‐off Value for Canine Trypsin‐like Immunoreactivity Assay

### Y.A. Wu^1^, W.H. Chuang^1^, J. Lidbury^1^, G. Fosgate^1,2^, C.H. Chang^1^, E. Gould^1^, M.K. Tolbert^1^, J. Gookin^1,3^, J. Steiner^1^, K. Aicher^1^

#### ^1^Gastrointestinal Laboratory, Texas A&M University, College Station, Texas, United States; ^2^Faculty of Veterinary Science, University of Pretoria, Onderstepoort, South Africa; ^3^Department of Clinical Sciences, North Carolina State University, Raleigh, North Carolina, United States

Laboratory data and clinical experience suggest that there has been an upward drift in the measurements of serum canine trypsin‐like immunoreactivity (cTLI) concentration for a widely used commercial chemiluminescent assay. Consequently, the aims of this study were to (1) re‐evaluate the reference interval (RI) for serum cTLI concentration, and (2) determine a cut‐off value for diagnosis of exocrine pancreatic insufficiency (EPI).

The lower limit of the RI was calculated based on the 5th percentile of serum cTLI concentrations measured in surplus samples from 97 healthy dogs enrolled in unrelated studies. To determine a new cut‐off value, questionnaires were sent to veterinarians who had submitted samples for measurement of serum cTLI concentration during the second half of 2023 and in which the results were ≤ 10.0 μg/L. Collected information included clinical signs, treatment(s), response to treatment(s), and concurrent diseases. Five board‐certified internists, blinded to case demographics, serum cTLI concentration, and the classifications of other evaluators, reviewed the questionnaires and classified cases as “EPI,” “not EPI” or “maybe EPI.” The final classification of a case as “EPI” or “not EPI” required at least 4/5 internists to assign that classification. Results where such agreement was not reached were excluded from further analyses. A cTLI cut‐off value for differentiation of “EPI” and “Not EPI” cases was determined using a weighted Youden index. Sensitivity was prioritized because (1) false negatives were considered more serious, and (2) the specificity was likely underestimated as dogs without EPI but cTLI values > 10 (μg/L) were not enrolled.

The new RI was determined to be ≥ 10.9 (μg/L). Forty‐seven dogs were consensually classified into each of the “EPI” or “Not EPI” groups for determination of the cut‐off value. The median (min–max) serum cTLI concentrations were 1.5 μg/L (<1.0–7.2 μg/L) for the “EPI” dogs, and 8.3 μg/L (2.2–10.0 μg/L) for the “Not EPI” dogs. The cut‐off value for diagnosis of EPI was determined to be 5.5 (μg/L) and had a sensitivity of 95.7% and a specificity of 83.0%.

Consistent with clinical observations, there has been an upward shift in the measurement of serum cTLI concentration using this commercial assay. Use of the RI and cut‐off value that we established will reduce the rate of false negative results for EPI when using this assay.

**Disclosures**

The authors of this study are all affiliated with the Gastrointestinal Laboratory at Texas A&M University, which offers laboratory testing, including measurement of serum cTLI concentrations, on a fee‐for‐service basis.

**Source of Funding**

None.

**ECVIM CSF Funding**

No.

**ESCG – European Society of Comparative Gastroenterology**

## ESCG‐O‐12

## Comparison of clinical, laboratory, and ultrasonographic findings in dogs with acute pancreatitis and acute idiopathic gastrointestinal disease

### M. Sidler^1^, D. Brugger^2^, B. Riond^3^, M.M. Dennler^4^, S. Unterer^1^, P.H. Kook^1^

#### ^1^Clinic for Small Animal Internal Medicine, University of Zurich, Zurich, Switzerland; ^2^Institute for Animal Nutrition and Dietetics, University of Zurich, Zurich, Switzerland; ^3^Veterinary Laboratory and Center for Clinical Studies, University of Zurich, Zurich, Switzerland; ^4^Clinic for Diagnostic Imaging, University of Zurich, Zurich, Switzerland

The clinical picture of dogs with acute pancreatitis (AP) has never been compared with acute idiopathic gastrointestinal (GI) disease (aiGId). Therefore, we prospectively compared clinical signs, CBC, biochemistry, C‐reactive protein concentrations (CRP), and ultrasonographic (US) findings in dogs with increased (AP) and normal/minimally increased lipase activity (aiGId).

Twenty‐seven dogs with lipase activity (LIPC Roche, substrate DGGR) > 400 U/L (RI, 17–156 U/L) and acute (<7d) GI signs and 49 dogs with lipase activity within/minimally (20 U/L) > RI and acute (<7d) GI signs were included. As we also wanted to compare US findings between groups, lipase activity was the decisive criterion for distinguishing AP from aiGId.

Standardized medical histories were taken in all dogs. Appetite, vomiting, diarrhea, abdominal pain, hydration, and lethargy at admission were graded using the modified clinical activity index (MCAI). CBC, biochemistry, CRP (Gentian canine CRP) results, and pancreatic as well as selected GI US findings were compared between groups.

Statistics comprised fixed effect models (group, sex, age, weight) and chi‐square tests. Median (range) duration of disease before presentation (AP 38.5h (3–96h), aiGID 53.9h (3–168h)) did not differ between groups. Diarrhea was significantly (*p* = 0.03) more often the first clinical sign observed in aiGId dogs. Neither frequency of individual clinical signs nor MCAI differed between groups. Median lipase activity and CRP (range) in AP and aiGId dogs were 1862 U/L (441–6712), 80.73 mg/L (6.4–370), and 68 U/L (14–176), 95.7 mg/L (6.4–368), respectively. CRP did not differ between groups. Of all laboratory values, only alkaline phosphatase activity (*p* = 0.005), bilirubin (*p* < 0.0001), and glucose (*p* < 0.0001) concentrations were significantly higher in AP. All but one AP dog (96%) and 12/49 (24%) aiGId dogs had US evidence of pancreatitis. In aiGId dogs, a heteroechoic (17/45; 38%), hyperechoic (9/45; 20 %), and hypoechoic pancreas (4/45; 9%) was found; 9/45 (20 %) aiGId dogs had a hyperechoic mesentery. All pancreatic US variables were significantly (*p* < 0.0001) more common in AP. Frequencies of a distended stomach, corrugated duodenum, and aperistaltic small bowel loops were not different between groups. Duration of hospitalization did not differ between groups.

In conclusion, dogs with AP do not differ clinically from aiGId except the first clinical sign was more often diarrhea in aiGId. Laboratory differences indicate that cholestasis and hyperglycemia is more common in AP dogs. Pancreatic US changes in aiGId are mostly indicative of chronic lesions, while pancreatic changes suggestive of AP are infrequent in dogs with normal/minimally increased lipase activity.

**Disclosures**

**Source of Funding**

**ECVIM CSF Funding**

No.

**ESCG – European Society of Comparative Gastroenterology**

## ESCG‐O‐13

## Use of budesonide in feline chronic enteropathies: A retrospective study of 102 cases

### M. Bigay^1^, E. Bourassi^2^, R. Javard^3^

#### ^1^VetAgroSup, Campus vétérinaire de Lyon, Marcy l'Etoile, France; ^2^Atlantic Veterinary College, University of Prince Edward Island Department of Companion Animals, Charlottetown, Canada; ^3^Centre DMVet, Montréal, Canada

Chronic enteropathy is a common disorder in feline patients with a rising prevalence. It is commonly treated with prednisolone but concurrent diseases or systemic side effects may prevent its use. Budesonide is a corticosteroid analogue developed for use in human patients with Crohn's disease to avoid systemic adverse effects. It has also been used with success in dogs with inflammatory bowel disease. Despite anecdotal reports of its use in feline chronic enteropathies, there is no objective clinical data available. The objective of this study was to evaluate the efficacy and safety of the use of budesonide in cats with chronic enteropathies.

A retrospective review of medical records from 2013 to 2022 from a referral hospital was used to identify cats with chronic enteropathy treated with budesonide. To be included in the study, cats had to meet the following criteria: clinical signs consistent with chronic enteropathy, thorough diagnostic evaluation with exclusion of other causes of chronic gastrointestinal signs, no administration of corticosteroids in the previous 3 weeks, treatment with budesonide (start of treatment T0) and at least one follow‐up between 1 and 3 months after start of treatment (T1). Clinical and biological side effects were recorded. The feline chronic enteropathy activity index (FCEAI) was calculated in all cats at T0, T1, and T2 (follow‐up between 6 and 12 months) if available.

One hundred and two cats were included in the study. At T0, T1 and T2, the mean FCEAI score were 4.7 (range 1–11), 1.0 (range 0–4) and 0.86 (range 0–5) respectively. Full clinical remission (defined as 75% or greater reduction in FCEAI score) was observed in 70% and 72% of cats at T1 and T2, respectively. In a subset of cats with confirmed inflammatory bowel disease by biopsies, the mean FCEAI score was 4.6 (range 1–11), 0.6 (range 0–4) and 0.5 (range 0–3). Full clinical remission in this group was observed in 90% and 78% of cats at T1 and T2, respectively.

Clinical and biological side effects were observed in 9% and 15% cats, respectively. Reported clinical effects were mild and included polyuro‐polydipsia (4%), lethargy (4%), dysorexia (2%), polyphagia (2%), alopecia (1%) and weight gain (1%). Biological effects were hyperglycemia (10%), leukocytosis (5%), increased ALP (1%), proteinuria (1%), arterial hypertension (1%), subclinical bacteriuria (1%).

Budesonide appears to be an effective treatment in cats with chronic enteropathy. It also seems to be well tolerated with infrequent and mild side effects.

**Disclosures**

**Source of Funding**

**ECVIM CSF Funding**

No.

**ESCG – European Society of Comparative Gastroenterology**

## ESCG‐O‐14

## Functional dyspepsia in dogs and humans a comparative approach and a retrospective study of 29 canine cases

### H. Kaufmann^1^, H. Duboc^2^, V. Freiche^3^

#### ^1^Centre Hospitalier Vétérinaire Frégis—IVC Evidensia France, Gentilly, France; ^2^Hôpital Louis‐Mourier Service d'Hépato‐gastro‐entérologie, Colombes, France; ^3^Ecole Nationale Vétérinaire d'Alfort, Maisons‐Alfort, France

Functional gastrointestinal disorders (FGID), including mostly functional dyspepsia (FD) and irritable bowel syndrome (IBS), are highly prevalent in humans. FD is a major and complex condition in human gastroenterology with female predominance, based on abdominal discomfort, epigastric pain or burning, postprandial fullness, or early satiety. Its pathophysiology is still not fully elucidated but included impaired gastric accommodation, *H. pylori*‐infection or brain‐gut axis dysfunction. To our knowledge, such clinical signs in the absence of an organic, metabolic or systemic cause, have not been reported in dogs.

The aim of the current study was to provide a comprehensive description of the presentation of suspected canine functional dyspepsia (CFD) in a retrospective case series.

Dogs presenting chronic gastro‐intestinal discomfort and nausea in the absence of structural disease that is likely to explain the clinical signs were reviewed between 2012 and 2023. Unremarkable laboratory data, imaging results and gastroscopy findings were mandatory to be included.

A total of 29 dogs were retrospectively enrolled. All dogs presented with gastrointestinal discomfort without notable biological or histological abnormalities, following exclusion criteria. CFD affected predominantly female (66%) and caused vomiting (97%), abdominal pain (58%), intermittent diarrhea (52%), pica (52%), chewing (48%), belching/yawning (41%), and other upper digestive discomfort. Duration of clinical signs was often prolonged, with a median of 1.5 years, and the median age of onset of 1.5 years. Anxiety of the owner was commonly reported and general quality of life of dogs was altered. Various treatments were attempted with varying success, including dietary changes, gastric acid reducers, prokinetics and corticosteroids. Follow‐up information was available for 21 dogs, with 76% showing improvement over a median follow‐up period of 12 months.

Functional dyspepsia is probably an emerging entity in dogs, especially in toy and small breed, middle age, female dogs, characterized by chronic gastrointestinal discomfort and vomiting in the absence of structural disease. Anxiety could also be a triggering factor of CFD onset. Overlap with food sensitivity, digestive dysbiosis, bilious vomiting syndrome, gastroesophageal reflux, or gastroparesis is still of concern. CFD should be included in differential diagnosis of canine upper digestive disorders and individual management is mandatory to allow improvement.

**Disclosures**

**Source of Funding**

Retrospective study, no source of funding.

**ECVIM CSF Funding**

No.

**ESCG – European Society of Comparative Gastroenterology**

## ESCG‐O‐15

## Occurrence of clinical signs in young dogs with and without *Giardia duodenalis* infection

### A. F. Freisleben^1^, K. Busch‐Hahn ^1^, S. Kanski^1^, R. Hailmann^1^, K. Weber^1^, J. Suchodolski^2^, R. Mueller^1^, U. Truyen^3^, K. Hartmann^1^

#### ^1^Centre for Clinical Veterinary Medicine, Ludwig‐Maximilians‐University Clinic of Small Animal Medicine, Munich, Germany; ^2^Department of Small Animal Clinical Sciences, Texas A&M University Gastrointestinal Laboratory, Texas, United States; ^3^Institute for Animal Hygiene an Veterinary Public Health, University of Leipzig, Leipzig, Germany

*Giardia duodenalis* is one of the most prevalent endoparasites affecting young dogs and can be associated with gastrointestinal signs or asymptomatic, and elimination of *Giardia* does not always improve clinical signs. The aim of this study was to assess association between gastrointestinal signs and canine *Giardia* infection.

Faecal samples from 142 dogs aged 1–18 months were tested for *Giardia* infection using immunofluorescence assay (IFA). Cyst counts were quantified as low (1–50), moderate (51–500), and high (>500 cysts per slide). Other causes for diarrhoea, such as endoparasites and parvovirus infection, were excluded. An overall clinical score was evaluated including faecal consistency (using the Purina faecal score), defaecation frequency, mucous admixtures, vomiting, appetite, activity, and abdominal pain. Clinical signs were compared between infected and non‐infected dogs with multiple regression analysis.

Thirty‐nine of 142 dogs (27%) tested positive for *Giardia*. Infected dogs did not show a higher overall clinical score (*p* = 0.6) than uninfected dogs. There was also no difference in number of dogs (43/103 uninfected; 22/39 infected) with increased faecal Purina score (≥4) (*p* = 0.53) or in the occurrence of other gastrointestinal signs (mucous admixtures (*p* = 0.74), defaecation frequency (*p* = 0.94), vomiting (*p* = 0.8), overall activity score (*p* = 0,6)). Also, the presence of moderate or high cyst numbers showed no correlation with presence or severity of clinical signs.

Therefore, other factors beyond *Giardia* infection play a role in gastrointestinal signs in young, infected dogs. Consequently, other gastrointestinal diseases should be ruled out, and elimination of *Giardia* cysts should not be the sole aim of treatment.

**Disclosures**

A. Freisleben Employee/salary: Clinic of Small Animal Internal Medicine, Centre for Clinical Veterinary Medicine, Ludwig‐Maximilians‐University, Munich, Germany Grants/research: none Speaking & consultancies: none Investments/commercial interests: none Gifts, hospitality, travel support: none S. Kanski Employee/salary: Clinic of Small Animal Internal Medicine, Centre for Clinical Veterinary Medicine, Ludwig‐Maximilians‐University, Munich, Germany Grants/research: positions was supported by the Federal Ministry for Economic Affairs and Climate Action (BMWK) on the basis of a decision by the German Bundestag Speaking & consultancies: none Investments/commercial interests: none Gifts, hospitality, travel support: none R. Hailmann Employee/salary: Clinic of Small Animal Internal Medicine, Centre for Clinical Veterinary Medicine, Ludwig‐Maximilians‐University, Munich, Germany Grants/research: positions was supported by the Federal Ministry for Economic Affairs and Climate Action (BMWK) on the basis of a decision by the German Bundestag Speaking & consultancies: none Investments/commercial interests: none Gifts, hospitality, travel support: none K. Busch Employee/salary: Clinic of Small Animal Internal Medicine, Centre for Clinical Veterinary Medicine, Ludwig‐Maximilians‐University, Munich, Germany Grants/research: ECVIM Endocrinology, CGS, Tierschutzstiftung Heidrun und Dr. Ulrich Bergmann Speaking & consultancies: Purina, Royal canin, Hill's, Vetconcept, Dechra, Laboklin, VetWebinar, cpPharma, Investments/commercial interests: none Gifts, hospitality, travel support: none R. Mueller Employee/salary: Clinic of Small Animal Internal Medicine, Centre for Clinical Veterinary Medicine, Ludwig‐Maximilians‐University, Munich, Germany Speaking & consultancies: Bayer Animal Health, Ceva Animal Health, Elanco Animal Health, Hill's, Royal Canin, MSD Animal Health, Synlab, and Zoetis Investments/commercial interests: none Gifts, hospitality, travel support: none.

**Source of Funding**

Positions of S. Kanski and R.Hailmann supported were supported by the Federal Ministry for Economic Affairs and Climate Action (BMWK) on the basis of a decision by the German Bundestag.

**ECVIM CSF Funding**

No.

**ESVIM – European Society of Veterinary Internal Medicine**

## ESVIM‐O‐1

## Canine chronic idiopathic rhinitis, management and outcome: A single centre retrospective observational study

### P. Henry^1^, A. Boag^1^, G. Woods^1^

#### ^1^Hospital for Small Animal, The Royal (Dick) School of Veterinary Studies Department of Internal Medicine, Edinburgh, United Kingdom

Canine chronic idiopathic rhinitis (CCIR) is one of the most common canine nasal diseases. Response to therapy is variable, and in the absence of consensus, empirical treatment trials are often undertaken to control clinical signs. The aim of this study was to describe the management and outcome of a cohort of 76 dogs diagnosed with CCIR.

Signalment, clinical signs and treatments prior to referral were retrospectively retrieved from files of dogs investigated for nasal disease between December 2018 and July 2023, in addition to therapy and outcomes when available (resolved, improved, static or worse). CCIR was defined according to previous literature as the presence of chronic inflammatory changes on histopathology without identifiable cause. Patients without tomography and/or endoscopy of the nasal cavities were excluded. Outcomes were compared between treatments using non‐parametric statistics. Data are presented as median [range].

Thirty‐four females and 42 males were included, aged 7.8 years‐old [0.5–14] and weighing 16.1kg [1.6–73]. Duration of clinical signs prior to referral was 184 days [3–1954]; bilateral purulent nasal discharge and sneezing were the most common. Only 12/76 dogs did not receive antibiotics prior to referral. Clinicopathological and rhinoscopy findings (74/76) were unspecific. C‐Reactive Protein was mildly elevated in 15/27 cases. Tomography revealed mild to moderate destructive rhinitis in 40/75 dogs, and concurrent sinusitis in 32/75.

After referral, the most common first‐line treatment was non‐steroidal anti‐inflammatory (NSAIDs, 46/76). Sixty‐two cases were followed‐up for 257 days [14–1701]. Forty‐seven, 24 and 13 dogs underwent a second, third and fourth treatment trial respectively. Antibiotics were the most common second trial (25/47, of which 18 received doxycycline), corticosteroids the most common third trial (15/24) and repeated antibiotics the most common fourth (7/13 repeated antibiotics and 6/13 corticosteroids).

Outcome was available for 62 dogs after initial treatment, 39 after second, 20 after third and 13 after fourth trial. Overall, 14/62 cases were cured, 8/62 static, and a majority (37/62) improved. Three cases were euthanized due to their disease. NSAIDs were significantly associated with clinical improvement after initial treatment trial (OR: 3.3, 95% CI: 1.2–9.6; *p* = 0.023) but there was no significant association between outcomes and later trials.

Most dogs diagnosed with CCIR are treated with antibiotics. Dogs receive variable therapies with unpredictable responses, but outcomes are largely favourable. A consensus on the treatment of CCIR is lacking but administration of NSAIDs as first‐line treatment was associated with improved outcome.

**Disclosures**

**Source of Funding**

**ECVIM CSF Funding**

No.

**ESVIM – European Society of Veterinary Internal Medicine**

## ESVIM‐O‐2

## Association between signalment, clinical signs and nasal diseases type and location in dogs and cats

### M. Vilcot^1^, E. Roels^1^, C. Clercx^1^, F. Billen^1^

#### ^1^Faculty of Veterinary Medicine, FARAH, University of Liege Department of Small Animal Clinical Sciences, Liège, Belgium

Nasal and nasopharyngeal diseases manifest through a wide variety of clinical signs, with certain manifestations being more frequently associated with specific diseases. Large‐scale respiratory semiological studies are needed to assist clinicians in disease localisation and prioritisation.

The aim of this retrospective cross‐sectional observational study was to explore the associations between signalment, clinical signs, and the location and type of nasal diseases in dogs and cats.

Medical data for a 4‐year period (2018–2022), from dogs and cats that underwent a rhinoscopy and received a definitive diagnosis, were extracted from our medical records database. Univariate and multivariate logistic regression models were employed for statistical analysis, with a significance level set at *P*‐value <0.05. For readability, adjusted odds ratios and their corresponding 95% CIs have been omitted from this abstract.

A total of 167 cats and 229 dogs were included. Rhinitis (40.1%), tumours or polyps (32.3%), and nasopharyngeal stenosis (31%) were the most common diagnoses in cats, whilst dogs frequently presented with fungal rhinitis (30.6%), lymphoplasmacytic rhinitis (26.6%), foreign bodies (25.8%), and tumours (12.2%).

Nasal localisation in cats was associated with sneezing, nasal discharge, and epistaxis, without stertor or dysphagia. In dogs, it was primarily associated with sneezing and retching. Nasopharyngeal disorders in cats presented with stertor, stridor, and dyspnoea without sneezing or nasal discharge, whereas in dogs, they manifested with reverse sneezing and decreased nasal airflow without sneezing.

Clinical variables significantly associated with rhinitis in cats included bilateral nasal discharge, particularly without stertor, dyspnoea, and systemic signs. Similarly, in dogs, bilateral nasal discharge was significantly associated with rhinitis, especially without epistaxis. Obstructive nasal/nasopharyngeal disease in cats due to tumours or polyps was associated with stertor, decreased nasal airflow, facial deformation, and systemic signs. Nasal tumour in dogs was associated with decreased nasal airflow, epistaxis, and none or serous nasal discharge. Fungal rhinitis was associated with chronicity, muco‐purulent discharge, epistaxis, nose discoloration and systemic signs, without decreased nasal permeability; while foreign bodies were associated with acute signs, without nasal discharge or systemic signs. Nasopharyngeal stenosis in cats was linked to a chronic duration of clinical signs and stertor without sneezing.

In conclusion, this study emphasizes the role of clinical presentation of dogs and cats with nasal or nasopharyngeal disorders as they can help in localising the disease and refining the differential diagnosis.

**Disclosures**

**Source of Funding**

**ECVIM CSF Funding**

No.

**ESVIM – European Society of Veterinary Internal Medicine**

## ESVIM‐O‐3

## Histopathological findings in the nasal biopsies of dogs without nasal signs compared to dogs with chronic inflammatory rhinitis

### H. Neittaanmäki^1^, S. Viitanen^2^, H.M. Javela^3^, E. Kuningas^4^, K. Koskinen^2^, M. Rajamäki^2^, N. Airas^3^

#### ^1^University of Helsinki University of Helsinki, Helsinki, Finland; ^2^University of Helsinki Department of Equine and Small Animal Medicine, Helsinki, Finland; ^3^University of Helsinki Department of Veterinary Biosciences, Helsinki, Finland; ^4^University of Helsinki Department of Equine and Small Animal Medicine, Helsinki, Finland

Chronic inflammatory rhinitis (CIR) is among the most common causes of chronic nasal signs in dogs. The diagnosis of CIR is obtained by excluding other causes for chronic nasal signs, such as dental disease, aspergillosis and nasal tumours, and histopathologic analysis of nasal biopsies. To our knowledge, there are no studies reporting the histopathological changes in the nasal mucosa of healthy dogs. Since the nasal cavity is in contact with inhaled microbes and particles, it is indicated to compare the histopathological findings in dogs without nasal signs to those with CIR to better understand the clinical relevance of inflammatory changes.

The purpose of this study was to describe the histopathological findings and inflammation scores in the nasal biopsies of dogs without signs of nasal disease compared to dogs with CIR. Study groups included client‐owned dogs without nasal signs euthanized for reasons unrelated to this study and donated for research purposes (*n* = 20) and previously collected archived nasal biopsies from dogs diagnosed with CIR (*n* = 20). The diagnosis of CIR was based on typical clinical presentation and computed tomography, rhinoscopy and histopathology findings indicative of CIR.

Nasal biopsy specimens were stained with H&E, Masson, PAS and Gram and evaluated with light microscopy. Inflammatory cell count was calculated for three random high‐power fields (2.37 mm^2^). The number of lymphocytes and plasma cells was increased (*P* <0.001) in dogs with CIR (median 87, range 7–658) when compared to dogs without nasal signs (median 19, range 1–57). The number of neutrophilic granulocytes (median 9, range 1–82; median 1, range 0–3; *P* <0.001) and intraepithelial inflammatory cells (median 8, range 0–96; median 1, range 0–3; *P* <0.001) was increased in dogs with CIR. On the contrary, the number of goblet cells was decreased in dogs with CIR. In addition, there was increased mucosal oedema and fibrosis in the lamina propria of the nasal mucosa in dogs with CIR. In conclusion, many factors in the nasal mucosa are altered in CIR and assessing all these factors will further help to understand the clinical relevance of the histopathological changes.

**Disclosures**

No disclosures to report.

**Disclosures**

**Source of Funding**

H. Neittaanmäki has received research grants from the Finnish Foundation of Veterinary Research (Suomen Eläinlääketieteen säätiö, SELS) and the Finnish Veterinary Research Foundation (Eläinlääketieteen tutkimuksen tukisäätiö, ETTS).

**ECVIM CSF Funding**

No.

**ESVIM – European Society of Veterinary Internal Medicine**

## ESVIM‐O‐4

## Evaluation of silent aspiration in dogs using videofluoroscopic swallow studies

### M. Grobman^1^, C. Reinero^2^, T. Lee‐Fowler ^3^, T. Lever^4^

#### ^1^Auburn University College of Veterinary Medicine Department of Clinical Sciences, Auburn, United States; ^2^University of Missouri College of Veterinary Medicine, Columbia, United States; ^3^Auburn University College of Veterinary Medicine, Auburn, United States; ^4^University of Missouri School of Medicine, Columbia, United States

In people, silent aspiration (SA) occurs with the entry of food or oral secretions below the vocal folds in the absence of cough. Detection of SA requires visualization of aspiration events as clinical markers of aspiration (i.e., cough) are absent. Silent aspiration has been identified in dogs using videofluoroscopic swallow studies (VFSS); however, the clinical and VFSS factors associated with SA in dogs are unknown. The objectives of this study were to compare demographic, clinical, and swallowing features between dogs with SA (penetration‐aspiration [PA] score 7), pathologic PA (pPA; PA score 3–6), and without pPA (npPA; PA score 1–2) on VFSS. VFSS and medical records from dogs with SA (*n* = 40), pPA (*n* = 17), and npPA (*n* = 20) presenting to 2 veterinary teaching hospitals were retrospectively reviewed. Statistical comparisons were made by one‐way analysis of variance (ANOVA), one‐way ANOVA on ranks, and Chi squared tests (*p* < .05). Mixed clinical signs (CS; GI and respiratory) were more likely in dogs with SA compared to npPA (*p* = .003). Exclusively GI CS were less likely in dogs with SA compared to pPA and npPA (*p* < .001). Gagging was more common in dogs with SA compared to pPA and npPA (*p* < .001). Pharyngeal swallow dysfunction was more common in dogs with SA compared to npPA (*p* < .001). SA was most likely to occur in dogs exhibiting mixed CS and clinical and VFSS features of pharyngeal dysfunction. VFSS should be performed in dogs with clinical signs of pharyngeal dysfunction because of the increased likelihood of SA.

**Disclosures**

Teresa Lever is a patent holder for the kennels used to perform the swallow studies. Other authors have nothing to disclose.

**Source of Funding**

None.

**ECVIM CSF Funding**

No.

**ESVIM – European Society of Veterinary Internal Medicine**

## ESVIM‐O‐5

## Description and comparison of efficacy and safety of blind bronchoalveolar lavage using a sterile endotracheal tube or direct laryngo‐tracheal catheterization in 51 cats

### J. Buton^1^, C. Didier^1^, M. Sarra Ferrer^1^, S. Faucher^1^, F. Granat^2^, B. Reynolds^3^, R. Lavoué ^3^, M. Mantelli^3^

#### ^1^Université de Toulouse, ENVT, Toulouse, France; ^2^Laboratoire central de biologie médicale, ENVT, Toulouse, France; ^3^INTHERES, Université de Toulouse, INRAE, ENVT, Toulouse, France

Bronchoalveolar lavage (BAL) fluid analysis is indicated in cats presented for lower respiratory tract diseases but the procedure is associated with a high anaesthetic risk. Several techniques are described, with or without endoscopic guidance. Blind BAL include catheterization through a sterile endotracheal tube, or direct laryngo‐tracheal catheterization. Data regarding the anaesthetic risk and the quality of the specimen obtained with blind BAL are currently lacking.

The aims were: (1) to describe two techniques of blind BAL; (2) to compare safety and quality of BAL accomplished by use of direct laryngo‐tracheal catheterization (D‐BAL) or sterile endotracheal tube (ED‐BAL) technique in cats.

51 cats (D‐BAL; *n* = 34 and ED‐BAL; *n* = 17) presented with diffuse lower respiratory tract diseases between 2016 and 2024 were retrospectively included. Signalment, clinical presentation, imaging and clinicopathological findings and final diagnosis, if achieved, were recorded.

Cats were anesthetized, and 8F sterile catheter was wedged in a bronchus. Two 5‐mL aliquots of warmed saline (0.9% NaCl) solution were supposedly infused into the left and right lung fields and aspirated manually. Cytological analysis and aero‐anaerobic bacterial cultures were performed for each obtained sample. Respiratory and heart rates after BAL, procedure duration, percentage of BAL fluid retrieved, lowest saturation of peripheral oxygen obtained by pulse oximetry, duration of oxygen supplementation and oropharyngeal contamination were compared between D‐BAL and ED‐BAL groups.

Procedure duration, respiratory and heart rates or average yield were not different between groups. Minimal saturation was significantly lower in the D‐BAL group (−6%; *p* = 0.044). Longer procedure duration was associated with longer need for oxygen supplementation in both groups (+5 min; *p* < 0.001). Intubation increases the risk of oropharyngeal contamination (OR=4.2 [1.02–22.3]_95%_; *p* = 0.044). A diagnosis could be established in all cats but 1.

Blind BAL is a moderately invasive procedure, that requires little equipment and can be performed in general practice. Accordingly to the present study, using a sterile endotracheal tube yields similar results to BAL without intubation, and might results in fewer per‐anaesthetic complications.

**Disclosures**

**Source of Funding**

**ECVIM CSF Funding**

No.

**ESVIM – European Society of Veterinary Internal Medicine**

## ESVIM‐O‐6

## Presence of fungi from bronchoalveolar lavage specimens in clinical cats and dogs – A retrospective evaluation over a ten‐year period

### W.T. Chang^1^, H.W. Chen^2^, Y.C. Wu^3^, P.Y. Lo^4^, C.H. Lin^5^

#### ^1^1.National Taiwan University, 2.TACS‐Alliance Research Center Lab of Small Animal Respiratory and Cardiovascular Medicine, Taipei, Taiwan; ^2^National Taiwan University 1.Department of Veterinary Medicine, 2.Animal Resource Center, Taipei, Taiwan; ^3^National Chung Hsing University Research Center for Animal Medicine, Taichung, Taiwan; ^4^TACS‐Alliance Research Center Lab of Small Animal Respiratory and Cardiovascular Medicine, Taipei, Taiwan; ^5^1.National Taiwan University, 2.TACS‐Alliance Research Center 1.VH & Graduate Institute Vet Clin Sci; 2.Lab of Small Anim Resp & CV Medicine, Taipei, Taiwan

Fungal lung diseases typically occur in endemic areas or among immunocompromised patients. However, their role in triggering immune responses or serving as lower airway flora in cats and dogs remains poorly understood. This study aimed to assess fungal isolation from bronchoalveolar lavage fluid (BALF).

A total of 246 cases with investigations of fungal organisms in BALF were included. Fungal culture has been performed as part of the microbiological workup of BALF since 2013, while panfungal PCR with sequencing has been used since 2021 for cases considering fungal infection among differential diagnoses.

Fungal culture yielded positive growth in 6.5% (10/155) of feline samples and 4.4% (4/91) of canine samples, but none showed evidence suggestive of fungal infection at follow‐up, except for one cat diagnosed with cryptococcosis. Panfungal PCR was performed on 29 cats and one dog to investigate fungal pathogens, showing positive results in 9 cats and none in the dog. All cats with a positive panfungal PCR result had no growth on fungal culture. *Cladosporium* was the most frequent genus identified (3/29). While the presence of lymphocytosis (56% vs. 45%, *P*=0.70) or hemosiderosis (78% vs. 45%, *P*=0.13) was not statistically different, multinucleated giant cells in BALF cytology were more common in cats with positive panfungal PCR results than in those with negative results (56% vs. 10%, *P*=0.02).

In conclusion, investigating fungi in BALF might be considered in cats with unexplained pulmonary hemorrhage and granulomatous inflammation. The role of fungi in eliciting immune responses in feline lungs warrants further study.

**Disclosures**

**Source of Funding**

NSTC 111‐2313‐B‐002‐062‐/TACS‐A 2024 Award/Support from National Taiwan University.

**ECVIM CSF Funding**

No.

**ESVIM – European Society of Veterinary Internal Medicine**

## ESVIM‐O‐7

## Comparison of cytological findings after obtaining two consecutive samples of bronchoalveolar lavages in dogs and cats: A prospective study of 21 cases

### M. Parreño^1^, S. Atencia^2^, A. Hernández^3^, E. Martínez De Merlo^4^, A. Caro^4^, A. Rodriguez^4^, E. González^5^

#### ^1^Anicura Vetsia Veterinary Hospital Internal Medicine, Madrid, Spain; ^2^Anicura Vetsia Veterinary Hospital Internal Medicine, Madrid, Spain; ^3^Anicura Vetsia Veterinary Hospital Oncology, Madrid, Spain; ^4^Complutense University Animal Medicine and Surgery, Madrid, Spain; ^5^Complutense University Animal Surgery and Medicine, Madrid, Spain

Bronchoalveolar lavage fluid (BALF) examination has become an important technique in the diagnosis of bronchopulmonary disease in small animal medicine. In human medicine there are guidelines that allow a standardized protocol for BALF to be carried out. However, this does not occur in veterinary medicine, where there is not standardized method for obtaining these BALF samples.

The aim of this prospective study was to compare the cytology quality between two BALF samples obtained consecutively in the same patient.

A total of twenty‐one client‐owned dogs and cats referred to investigate respiratory signs in a referral hospital in Spain were included.The inclusion criteria were dogs and cats that presented with lower respiratory signs and underwent two consecutive BALFs under the same anesthesia.

All patients underwent a complete physical examination. Complementary tests included haematology, biochemistry and thoracic imaging: radiographs, computed tomography scan or pulmonary ultrasound were performed depending on the individual case. Bronchoscopy and two consecutive BALFs were performed under general anesthesia. A cytology exam was performed on both BALF samples separately. Cellular conservation as well as mucus contamination were compared.

Sample conservation differed in 76.1% (16/21) of the cases. The second BALF had better cellular conservation in 62.5% (10/16) of the samples. In the other 5 cases cellular conservation was similar in both BALF. Regarding the amount of mucus, a difference was observed in 47.6% (10/21) of the samples. In 60% (6/10) of the cases the second BALF had less amount of mucus.

When two consecutive BALFs are performed in the same patient, the second BALF sample obtained seems to offer better quality cytology, showing less mucus and detritus.

**Disclosures**

**Source of Funding**

**ECVIM CSF Funding**

No.

**ESVIM – European Society of Veterinary Internal Medicine**

## ESVIM‐O‐8

## Clinical and prognostic relevance of Mycoplasma felis PCR detection in feline lower respiratory tract diseases

### T. Robin^1^, M. Bigay^1^, C. Touzet^2^, K. Le Boedec^1^

#### ^1^Centre Hospitalier Vétérinaire Fregis—IVC Evidensia France, Paris, France; ^2^Clinique vétérinaire Olliolis, Ollioules, France

In humans, *Mycoplasma pneumoniae* contributes to the development of chronic lower respiratory tract (LRT) signs. Similarly, *Mycoplasma felis* is considered a commensal of feline upper airways and is assumed pathogenic in the LRT. However, a meta‐analysis failed to show a significant association between *Mycoplasma felis* detection in bronchoalveolar lavage fluid (BALF) and presence of LRT disease in cats, suggesting that detecting *Mycoplasma felis* in LRT could be incidental.

This retrospective cohort study aimed to compare two groups of cats with LRT diseases (one with *Mycoplasma felis* detected in the BALF [M+] and the other without [M−]), in terms of signalment, clinical signs, diagnostics results, response to treatment, and survival.

All cats for which *Mycoplasma felis* was searched by PCR in BALF between 2016 and 2023 at our hospital were included. Cats with evidence of oropharyngeal contamination, for which PCR results were under the quantification level, and without follow‐up information were excluded. Cats who had received antibiotics effective against *Mycoplasma felis* before BALF collection were excluded if the PCR results were negative. Follow‐up information was retrieved from medical records and by contacting referring veterinarians and owners.

Fifty‐five cats were included, including 19 in the M+ group and 36 in the M‐ group. Significant differences were detected between the two groups in prevalence of systemic signs (M+: 0%, M−: 28%, P=0.01), prevalence of bronchial collapse on bronchoscopy (M+: 28%, M−: 6%, *P*=0.03), prevalence of alveolar lesions on thoracic radiography (M+: 57%, M‐: 24%, *P*=0.04), and percentage of neutrophils (average values in M+: 65% and in M−: 35%, *P*=0.002) and eosinophils (average values in M+: 9% and in M−: 25%, *P*=0.03) in the BALF.

Antibiotics were used significantly more frequently to treat M+ cats (M+: 89%, M−: 42%, *P*=0.001) than M− cats, often treated with steroids. No significant difference was found in treatment response (short term: *P*=0.86 and long term: *P*=0.37) and survival time (*P*=0.75) between the two groups.

This study revealed that presence of alveolar lesions on thoracic radiographs and pronounced neutrophilic inflammation in BALF were significantly associated with detection of *Mycoplasma felis* in BALF. However, it was not possible to determine if this association was causal, consequential, or contextual (ie, sharing the same cause). Detection of *Mycoplasma felis* in BALF did not negatively impact prognosis. However, as most M+ cats received antibiotics, the necessity of treating *Mycoplasma felis* when isolated from feline LRT could not be evaluated.

**Disclosures**

None.

**Source of Funding**

None.

**ECVIM CSF Funding**

No.

**ESVIM – European Society of Veterinary Internal Medicine**

## ESVIM‐O‐9

## Comparison of C‐reactive protein with other markers of inflammation in dogs with meningoencephalitis of unknown origin and steroid responsive meningitis‐arteritis

### T. Johnston^1^, A. Tebb^1^, K. Langner^1^

#### ^1^Western Australian Veterinary Emergency and Specialty Internal Medicine, Perth, Australia

C‐reactive protein (CRP) is an acute phase protein used for diagnosing and monitoring canine inflammatory diseases. Serum CRP concentrations have been evaluated in dogs with steroid‐responsive meningitis‐arteritis (SRMA) and recently in dogs with meningoencephalitis of unknown origin (MUO). Data regarding the assessment of CRP concentrations in comparison with other markers of inflammation for the purpose of differentiating MUO and SRMA are lacking.

The aim of this study was to analyze serum CRP concentrations and other clinical markers of inflammation in a cohort of dogs with confirmed MUO and SRMA and to evaluate their potential to aid in the differentiation of the 2 disease entities.

Medical records from a referral hospital were evaluated between 2018 and 2023 for dogs with serum CRP concentrations, cerebrospinal fluid analysis and where performed, advanced imaging of the central nervous system. Dogs were allocated to MUO and SRMA groups following review of the records with signalment, clinical and clinicopathological details recorded for each patient. Descriptive statistics were used to compare parameters between MUO and SRMA groups. Associations of selected systemic inflammatory markers between MUO and SRMA were assessed by odd ratios (OR). An area under the curve receiver operating characteristic (AUROC) and Youden's index were used to calculate the optimal cut‐off for CRP concentration to differentiate MUO and SRMA.

Twenty‐nine and 26 dogs were assigned to the MUO and SRMA groups, respectively. Dogs with MUO compared to dogs with SRMA had a significantly lower body temperature (median 38.4^o^C, range 37–39.8 vs. 39.2, 37.6–40.5, *p* = 0.0007), serum CRP concentration (8 mg/L, 3–95.7 versus 143.1, 8–333, *p* = <0.0001), leukocyte count (9.4 ×10^9^/L, 5.5–19.2 vs. 22.15, 11.6–35, *p* = <0.0001), neutrophil count (7.6 ×10^9^/L, 5.5‐19.2 vs. 20.36, 9.16‐27.3, *p* = <0.0001) and monocyte count (0.56 ×10^9^/L, range 0.1–1.38 versus 2.55, 0.55–4.37, *p* ≤ 0.0001). When compared to the MUO group, dogs with SRMA had higher odds to have an increased body temperature (OR 76.6; 95% Cl: 10.16–837), increased serum CRP concentration (OR 20.5; 95% Cl: 2.8–227.9), leukocytosis (OR 113.4; 95% Cl 13.06–1203), neutrophilia (OR 103.5; 95% Cl: 15.28–512.1) and monocytosis (OR 90; 95% Cl 11–958.6). The AUROC when discriminating MUO and SRMA was 0.94 (95% CI: 0.92–1) and the best cut–off value was 50 mg/L with a sensitivity and specificity of 0.96 (95% Cl: 0.81–1) and 0.97 (95% Cl: 0.83–1), respectively.

This study shows that serum CRP concentrations, body temperature and cellular markers of inflammation can aid in the differentiation of MUO and SRMA in dogs.

**Disclosures**

**Source of Funding**

**ECVIM CSF Funding**

No.

**ESVIM – European Society of Veterinary Internal Medicine**

## ESVIM‐O‐10

## Evaluation of C‐reactive protein assay in canine bronchoalveolar lavage fluid as a biomarker of bacterial bronchopneumopathy in dogs: A multicenter prospective study

### A. Petitpre^1^, D. Pichard^2^, M. Canonne^2^, T. Giampaolo^3^, E. Krafft^3^, A. Lamoureux^1^, M. Cervone^4,5^

#### ^1^Centre hospitalier vétérinaire Anicura Nordvet, La madeleine, France; ^2^Ecole Nationale Vétérinaire de Maisons Alfort Unité Pédagogique de Médecine, Maison Alfort, France; ^3^Université de Lyon, VetAgro Sup Département des Animaux de Compagnie de Loisir et de Sport, Marcy L'Etoile, France; ^4^Université de Lyon, VetAgro Sup, Marcy L'Etoile, France; ^5^Clinique vétérinaire Sevetys Crozatier, Paris, France

Diagnosis of bacterial bronchopneumopathy (bBP) in dogs requires bronchoalveolar lavage fluid (BALF) analysis. However, results are delayed, and antimicrobials are commonly blindly prescribed during this time. An unpublished pilot study demonstrated that CRP can be detected in canine BALF (BALF‐CRP) mainly in case of bBP.

This multicenter prospective study aimed to evaluate if BALF‐CRP can differentiate bBP from non‐bBP in dogs.

Eighty BALF from dogs were collected between 2022 and 2024. The final diagnosis of bBP versus non‐bBP was achieved by 3 board‐certified internists. BALF‐CRP concentration was assessed using the Konelab Termofisher® analyzer (limit of detection 5mg/L). Sensitivity and specificity of BALF‐CRP in distinguishing dogs with BP and non‐BP were calculated.

Thirty dogs with bBP and 50 dogs with non‐bBP were included. BALF‐CRP >5mg/L had a sensitivity of 30% (14.7%–49.4%), a specificity of 80% (66.3%–90%), a positive predictive value (PPV) of 47% (29%–66%) and a negative predictive value (NPV) of 65% (59%–71%) in differentiating dogs with bBP from dogs with non–bBP. When a cut‐off value of >11mg/L was applied, specificity increased to 94% (83.5%–98.8%), with a sensitivity of 20% (7.7%–38.6%), a PPV of 67% (35%–88%) and a NPV of 66% (62%–70%). The association of BALF–CRP >11mg/L and plasma‐CRP ≥50 mg/L yielded a specificity of 100% (79%–100%), a sensitivity of 18% (5%–40%), a PPV of 100% (40%–100%) and a NPV of 47% (42%–52%).

BALF‐CRP is a specific but insensitive biomarker of bBP in dogs. BALF‐CRP concentration >11 mg/L could support antibiotic administration while awaiting results, particularly if plasma‐CRP concentration is concomitantly increased (≥50 mg/L).

**Disclosures**

**Source of Funding**

**ECVIM CSF Funding**

No.

**ESVIM – European Society of Veterinary Internal Medicine**

## ESVIM‐O‐11

## Donor and unit‐specific factors influencing hemolysis in stored canine and feline packed red blood cells

### P. Reguera Burgos^1^, R.R.F. Ferreira^2^, C. Martínez Gil^1^, I. Mesa Sánchez^3^

#### ^1^AÚNA Especialidades Veterinarias—IVC Evidensia, Valencia, Spain; ^2^Banco de Sangue Animal, Porto, Portugal; ^3^Banco de Sangre Animal, Barcelona, Spain

Quality control analysis of packed red blood cells (pRBCs) is becoming a routine procedure in pets blood banks. Hemolysis, as an indicator of storage lesions, is a limiting factor for the unit's storage time. Previous research has shown a progressive increase in hemolysis percentage over time in stored pRBCs in both dogs and cats. In human medicine, genetic polymorphisms in blood donors may contribute to donor‐specific differences in the survival of red blood cells (RBCs) during storage. The purpose of this study was to test the potential impact of donor and unit‐specific characteristics on pRBCs storage lesions.

A total of 7461 canine and 3392 feline pRBC units were collected between 2021 and 2023. The hemolysis percentage was evaluated at different storage times: respectively in canine and feline units, 2105 and 2405 analyses were performed pre‐storage, 57 and 52 in units stored <1 week, 51 and 12 between 1–2 weeks, 130 and 56 between 2–3 weeks, 3,076 and 648 between 3–4 weeks, 1,838 and 201 between 4–5 weeks, and 204 and 18 between 5–6 weeks. The impact of donor and unit‐specific factors (gender, age, blood group, hematocrit and unit volume) on hemolysis was statistically evaluated.

In canine units, the hemolysis percentage significantly increased from 0.07 (± 0.13) at pre‐storage time to 0.85 (± 0.57) after 6 weeks (*p* < 0.001). Combined analysis of every hemolysis measurements revealed that units from female donors (0.59% vs. 0.57% in males; *p* < 0.032), and DEA 1 positive units (0.64% vs. 0.49% in DEA 1 negative; *p* < 0.001) were significantly associated with a higher hemolysis percentage. Canine units with higher hematocrit levels (*p* < 0.001) and larger volumes (*p* < 0.001) exhibited significantly decreased hemolysis percentage. In feline units, the hemolysis percentage significantly increased from 0.06 (± 0.08) at pre‐storage time to 1.08 (± 0.69) after 6 weeks (*p* < 0.001). Feline units with B blood type (0.34%; *p* < 0.001) and AB blood type (0.96%; *p* < 0.001) were significantly associated with a higher hemolysis percentage compared to A blood type units (0.22%). Feline units with higher hematocrit levels (*p* < 0.001) and larger volumes (*p* < 0.001) exhibited significantly decreased hemolysis percentage. An increase in age of the feline donor (only for units stored < 2 weeks) was associated with a significant increase in hemolysis (*p* < 0.001).

These findings highlight the impact of donor and unit‐specific factors on measures of RBCs hemolysis during routine pRBCs storage.

**Disclosures**

Rui R. F. Ferreira and I. Mesa Sánchez are employees of the Banco de Sangue Animal (Porto, Portugal) and Banco de Sangre Animal (Barcelona, Spain), respectively. The study has not received financial support from any other company. P. Reguera Burgos and C. Martínez Gil are not affiliated with the Banco de Sangue Animal (Porto, Portugal) or the Banco de Sangre Animal (Barcelona, Spain). The study has been conducted in collaboration with the Banco de Sangue Animal (Porto, Portugal) and the Banco de Sangre Animal (Barcelona, Spain). P. Reguera Burgos and C. Martínez Gil have not received financial benefit for conducting this study.

**Source of Funding**

**ECVIM CSF Funding**

No.

**ESVIM – European Society of Veterinary Internal Medicine**

## ESVIM‐O‐12

## Anaemic retinopathy in a referral population of anaemic cats

### S. Broughton^1^, J. Preston^1^, M.C. Fischer^1^, B. Glanemann^1^

#### ^1^Royal Veterinary College Department of Clinical Science and Services, London, United Kingdom

Anaemic retinopathy (AR) is described in humans, especially those with a haemoglobin concentration (hgb) of <8g/dL, with a prevalence of 20%–28.3%. These present as preretinal, retinal, and subretinal haemorrhages and have been reported to affect vision in humans. A single study on this condition is available in cats and reported retinal lesions in 20 cats with a hgb <5g/dL (equivalent to a packed cell volume (PCV) of <15%), however no exclusion criteria were applied for potential confounders. We aimed to describe the fundic findings of all anaemic cats, excluding those with possible confounding factors to retinal haemorrhage, presenting to a referral hospital over a one‐year period.

Ninety‐two cats met the inclusion criteria (PCV <24% and fundic examination performed), and were not hypertensive, thrombocytopenic or had evidence of a bleeding disorder.

Seventeen cases (18.4%) had changes consistent with AR, and of the 58 cats whose PCV was below 15%, 16 (27.6%) had evidence of AR. The lowest PCV of cats with AR was median 7% (range 5%–17%) which was significantly lower (*P* < 0.001) than cats without anaemic retinopathy (non‐AR) (median 13% (range 4%–23%)). Sixteen cases with AR required at least 1 blood transfusion for their systemic disease (range 1–3; median 2 transfusions). Of the non‐AR cats, 41/75 required blood transfusion(s) (range 1–3; median 1 transfusion). Cats with AR were unwell for a median of 21 days (range 1–121 days) at the time of presentation. Only one cat with AR had a normal PCV recorded at some point whilst they were unwell (PCV 26%). Of the 75 non‐AR cats, 30 had a normal PCV recorded when they were unwell (24%–43%; median 27%). Fifteen of the AR cats had a non‐regenerative anaemia, with the most common disease process (12 cases; 70.6%) being suspected or proven precursor‐targeted immune mediated anaemia(PIMA)/pure red cell aplasia(PRCA). Thirty‐nine (52%) of the non‐AR cases were anaemic due to blood loss or haemolysis, and only nine (12%) had PIMA or PRCA suspected or proven. Four cats with AR had a reassessment at the referral hospital and were clinically improved. Three had resolution of the retinopathy and 1 was showing improvement 5 days after initial discharge.

AR was present in cats at a similar incidence to anaemic human patients. The vast majority of cats with AR had a non‐regenerative anaemia, and required at least one blood transfusion. AR appeared to resolve with improvement in anaemia.

**Disclosures**

S. Broughton has held a role part funded by Royal Canin B. Glanemann has received funding from ECVIM clinical fund, the Kennel club, SAMSOC and the Morris Animal Foundation.

**Source of Funding**

This study was not funded.

**ECVIM CSF Funding**

No.

**ESVIM – European Society of Veterinary Internal Medicine**

## ESVIM‐O‐13

## General health quality‐of‐life questionnaires reveal subtle behavioural changes in undiagnosed hypertensive older cats

### E. Beriot^1^, J. Bestwick^2^, Y.M. Chang^3^, R. Geddes^2^, J. Elliott^1^

#### ^1^Royal Veterinary College Comparative Biomedical Sciences, London, United Kingdom; ^2^Royal Veterinary College Clinical Science and Services, London, United Kingdom; ^3^Royal Veterinary College Research Support Office, London, United Kingdom

Hypertension is common in older cats. Early clinical signs may be non‐specific whilst consequences of target organ damage can be severe. Owner‐reported early indicators of disease would be beneficial for targeted screening of at‐risk cats.

During a 52‐month period, owners of older cats (≥8 years old), without prior medical diagnoses and undergoing routine health screening (history, physical examination, serum biochemistry ±TT4, systolic blood pressure (SBP) [Doppler method] ±fundic examination, ±urinalysis), were asked to complete a general‐health/quality‐of‐life questionnaire (QoLQ). This included 4 domains (general health, behaviour, eating and management) with both numerical‐ and descriptive‐style frequency/severity scoring questions. Hypertension was diagnosed based on mean SBP >160 mmHg on ≥2 occasions or the presence of hypertensive retinopathy. Individual question scores from QoLQ of cats diagnosed as hypertensive were compared using Wilcoxon matched‐pairs signed rank test to question scores from age‐matched cats considered healthy.

Five‐hundred and forty‐nine cats were health screened of which 37 were diagnosed as hypertensive. QoLQs available from 33/37 hypertensive cats (SBP 191±21 mmHg; age 16±3 years) were compared to 33 age‐matched healthy cats (SBP 128±19 mmHg; age 16±3 years). Owners reported hypertensive cats to be significantly less interactive (*p* = 0.01) and playful (*p* = 0.02) and perform less frequent natural scratching behaviour (*p* = 0.02), during the week prior to routine health screening, compared to their age‐matched counterparts.

An owner‐perceived lower degree of interaction, playfulness and scratching behaviour in older cats could indicate the presence of hypertension and should prompt veterinarians to measure blood pressure.

**Disclosures**

R. Geddes has received funding from Petplan, Royal Canin, an RVC Internal Grant, The Academy of Medical Sciences and The Everycat Foundation; has previously had a consultancy agreement with Boehringer Ingelheim; has received speaking honoraria from Boehringer Ingelheim, Idexx and Royal Canin. Jonathan Elliott receives Grant funding from CEVA Animal Health, Boehringer Ingelheim, Idexx Laboratories, Royal Canin, Waltham Petcare Science Institute and Zoetis. He is engaged as a consultant by: Boehringer Ingelheim, Elanco Animal Health, Merck‐Intervet Animal Health, Idexx Laboratories, InvetX Ltd., Purina, Variant Bio, Waltham Petcare Science Institute. J.P. Bestwick is currently undertaking a PhD studentship funded by Royal Canin SAS and previously completed an MPhil studentship funded by BSAVA PetSavers.

**Source of Funding**

**ECVIM CSF Funding**

No.

**ESVIM – European Society of Veterinary Internal Medicine**

## ESVIM‐O‐14

## Drivers and challenges of modern clinical education in veterinary teaching hospitals: Using causal loop modelling to understand better the complexity of interactions at play within academic hospitals and within the broader university system

### E. Meler^1^, R. Richards^2^

#### ^1^The University of Queensland School of Veterinary Science, Brisbane, Australia; ^2^The University of Queensland Business School, Brisbane, Australia

Clinical practice‐based training has been traditionally delivered in Veterinary Teaching Hospitals (VTHs) and is a crucial part of veterinary education. However, with declining government funding for universities and growing competition from the private sector, VTHs are placed under increased pressure to be financially sustainable which can compete with their primary educational role. Recent papers have brought to light the resulting tensions experienced by clinical teachers as well as the mounting trend of academic clinical staff attrition.

In this research, our goal is to explore the interactions between patient management and clinical teaching and to understand the challenges it triggers for clinical teachers within VTHs and more broadly within their academic environment. By using a system thinking tool such as a Causal Loop Diagram (CLD), we aim to reveal important often hidden feedback loops that drive the behaviour of the system and to improve the clarity of the mental representation of academic clinical teaching in veterinary medicine and its challenges. The longer‐term goal is to unveil meaningful and long‐lasting strategic solutions to allow VTHs to retain competent staff and continue to thrive.

The CLD was built based on a mixed method approach encompassing a thorough literature review across both human and veterinary literature, an online survey of veterinary clinical teachers, and observations within an AVMA accredited VTH. The CLD was then reviewed by experts to ensure the authentic representation of the main variables and of their respective interactions.

The building of the CLD model confirmed that the academic hospital environment was incredibly complex and that it was governed by 5 major entities: 1) patient caseload and fluidity of workflow within the VTH, 2) quality of the clinical learning student experience, 3) funding available, 4) staff recruitment and retention, and 5) academic progression and promotion. Increasing clinical workload was related to negative reinforcing feedback loops including increased work stress, difficulty to cope with the competing demands of the job, imbalanced work/life and lesser job satisfaction. Improved access to resources, funding, and hospital culture were related to positive reinforcing feedback loops. It was also the case for an improved preparedness and mindset of students training in the VTH and an improved curriculum design.

To the authors’ knowledge, this is the first time that a CLD is attempted to try unravelling the complexity of the work environment within VTHs and to understand the drivers of tensions experienced by clinical teachers.

**Disclosures**

**Source of Funding**

**ECVIM CSF Funding**

No.

**ESVIM – European Society of Veterinary Internal Medicine**

## ESVIM‐O‐15

## Causes and outcome of systemic inflammatory response syndrome (SIRS) in dogs – Analysis of 172 cases

### M. Knopf^1^, C. Weingart^1^, M. Fulde^2,3^, A. Lübke‐Becker ^2,3^, R. Merle^4^, C. Robé ^2,5^, U. Rösler^2,5^, T. Sandbrink^1,2^, S. Schwarz^2,3^, M. Skrodzki^1^, B. Kohn^1,2^

#### ^1^Small Animal Clinic, Freie Universität Berlin, Berlin, Germany; ^2^Veterinary Center for Resistance Research, Freie Universität Berlin, Berlin, Germany; ^3^Institute of Microbiology and Epizootics, Freie Universität Berlin, Berlin, Germany; ^4^Institute for Veterinary Epidemiology and Biostatistics Freie Universität Berlin, Berlin, Germany; ^5^Institute for Animal Hygiene and Environmental Health, Freie Universität Berlin, Berlin, Germany

SIRS and sepsis can lead to organ dysfunctions and are associated with high mortality rates. The aim of this study was to evaluate dogs with SIRS or sepsis regarding the underlying diseases, organ dysfunctions, and outcome.

Dogs were included in this prospective observational study if they met at least two out of four SIRS‐criteria: 1) hypo‐/hyperthermia, 2) tachycardia, 3) tachypnea, 4) leukocytosis/leukopenia/left shift. They were categorized as “non‐septic SIRS” (G1), “sepsis” (G2) (“sepsis without organ dysfunction” [G2a], “severe sepsis” [G2b] [dysfunction of at least one organ system], “septic shock” [G2c] [hypotension despite adequate fluid resuscitation]) and “SIRS with suspected infection” (G3). Additional blood smears and/or lactate measurements were performed, using surplus material. Statistical analysis was performed with SPSS®.

From 01/2023 until 12/2023, 172 cases were included (G1: 76; G2: 68 [G2a: 43, G2b: 21, G2c: 4]; G3: 28), six dogs were presented twice with SIRS of different origins. Most common origin of SIRS/sepsis was the gastrointestinal tract (*n* = 61, 35%; e.g. pancreatitis, parvovirosis) followed by vector‐borne diseases (*n* = 28, 16%) and (suspected) neoplasia (*n* = 23, 13%). There was a dysfunction of at least one organ system in 68 (40%) cases (G1: 32/76, 42%; G2: 25/68, 37%; G3: 11/28, 39%) (most frequently cardiovascular system, followed by respiratory tract, kidney and liver). Overall, 128 (74%) cases survived until discharge (G1: 52/76, 68%; G2: 59/68, 87%; G3: 17/28, 61%).

The best survival rate was found in dogs with sepsis. The survival rate for patients with SIRS in this study was similar to that in other studies.

**Disclosures**

Myriam Knopf declares that she is supported by a scholarship from the Ernst Reuter Foundation. Christiane Weingart declares that she has given lectures for veterinary pharmaceutical and diagnostics companies. Marcus Fulde, Antina Lübke‐Beker, Roswitha Merle, Caroline Robé, Uwe Rösler, Teresa Sandbrink, Stefan Schwarz and Marianne Skrodzki declare that they have no conflict of interest. Barbara Kohn has held lectures, had research collaborations, and acted as a consultant for veterinary pharmaceutical and diagnostic companies.

**Source of Funding**

Myriam Knopf declares that she is supported by a scholarship from the Ernst Reuter Foundation.

**ECVIM CSF Funding**

No.

**ESVIM – European Society of Veterinary Internal Medicine**

## ESVIM‐O‐16

## Long‐term follow‐up of dogs treated with a chlorambucil‐prednisolone combination for steroid‐responsive meningitis‐arteritis: a prospective study of 12 cases

### T. Bouzouraa^1^, J.L. Cadoré ^2^, L. Chabanne^2^

#### ^1^VETALPHA, Dommartin, France; ^2^VetAgro Sup Lyon, Marcy l'Étoile, France

Steroid‐Reponsive Meningitis Arteritis (SRMA) is a systemic immune‐mediated disease that mainly affects large‐breed, young adult dogs. Diagnosis relies on a combination of epidemiologic, phenotypic and clinocopathological findings. Treatment involves long‐term immunosuppression, primarily with corticosteroids. Second‐line immunosuppressors might improve treatment efficacy and help reducing the dosage and adverse effects of steroids. We previously documented the usefulness of chlorambucil, as a potential therapy for SRMA.

Here, we prospectively describe long‐term follow‐up of 12 dogs diagnosed with SRMA, and treated with a combination of chlorambucil and prednisolone.

Client‐owned dogs presented between January 1st 2021 and December 31st 2023, were prospectively included if complete data was available. Diagnosis was based on associations of the following: predisposed breed, fever, neck pain, leukocytosis/neutrophilia, increased CRP (>30mg/L), absent CT and/or MRI lesions, CSF pleocytosis and/or proteinorrachia, negative PCR‐screening and bacterial culture, obvious and sustained improvement with immunosuppression. Categorical and continuous variables were estimated with percentages and medians (min–max).

Twelve dogs were diagnosed with SRMA (8 males, 3 males). Brain and cervical spinal MRI (7/12 cases) and CT (5/12 cases) were unremarkable. CSF count was increased in all cases (116–390 cells/μL). Concurrent immune‐mediated polyarthritis was diagnosed in 1/12 case. Median age was 22 months (7–66). Median weight was 25.6 kg (14.3–46.6). There were 3 Bernese Mountain Dogs and Golden Retrievers, then 1 of each (Weimaraner, Boxer, Irish Setter, Whippet, Nova Scotia, American Staffordshire Terrier).

Chlorambucil was prescribed at 0.2 mg/kg q24h PO for 4 months, then reduced at 0.1 mg/kg for 2 months. Prednisolone was prescribed at 2 mg/kg daily IV or PO for the first 2 days, and reduced at 1.5 mg/kg PO for 1 week, then tapered every 2 weeks at 1.25 mg/kg, 1 mg/kg, 0.75 mg/kg, 0.5 mg/kg, and 0.25 mg/kg and stopped.

Median follow‐up was 89 weeks (26–102). Complete resolution of clinical signs was observed in 12/12 dogs (100%, 95% CI [71.13; 100.00]) by a median of 1 days (1–3), and 3/12 dogs relapsed (25%, 95% CI [0.50;49.50]). Adverse effects were reported in 1/12 dog (8.33%, 95% CI [0.00;23.97]) and consisted in moderate normocytic, normochromic non‐regenerative anemia (Hemoglobin: 8.7 g/dl) that responded to chlorambucil withdrawal.

We proposed here a treatment protocol for dogs with SRMA. Long‐term follow‐up revealed that chlorambucil‐prednisolone was effective and safe for dogs with SRMA.

**Disclosures**

**Source of Funding**

**ECVIM CSF Funding**

No.

**ISCAID – International Society for Companion Animal Infectious Diseases**

## ISCAID‐O‐1

## Characterization of the lymphocyte response in cats with feline infectious peritonitis during antiviral treatment using the SMART tube technology for flow cytometry in feline full blood samples

### K. Zwicklbauer^1^, D. von La Roche^2^, D. Krentz^1^, L. Kolberg^3^, M. Alberer^3^, K. Hartmann^1^, UU. von Both^3,4^, S. Härtle^5^

#### ^1^LMU Small Animal Clinic, Centre for Clinical Veterinary Medicine, LMU Munich LMU Small Animal Clinic, Centre for Clinical Veterinary Medicine, Munich, Germany; ^2^Department of Veterinary Sciences, AG Immunology, LMU Munich Department of Veterinary Sciences, AG Immunology, Planegg, Germany; ^3^Division of Paediatric Infectious Diseases, Dr. von Hauner Children's Hospital Division of Paediatric Infectious Diseases, Dr. von Hauner Children's Hospital, Munich, Germany; ^4^German Center for Infection Research (DZIF), partner site Munich Division of Paediatric Infectious Diseases, Dr. von Hauner Children's Hospital, Munich, Germany; ^5^Department of Veterinary Sciences, AG Immunology, LMU Munich Department of Veterinary Sciences, AG Immunology, LMU Munich, Planegg, Germany

Feline infectious peritonitis (FIP) caused by feline coronavirus (FCoV) is an immune‐mediated and, if untreated, fatal disease in cats. A common feature of FIP is moderate to severe lymphopenia most likely due to the release of pro‐apoptotic factors from phagocytes that develops into lymphocytosis when treated successfully with oral GS‐441524. This study aimed to further characterize the lymphocytosis occurring during antiviral treatment using flow cytometric quantification of T‐helper cells, cytotoxic T‐cells, and B‐cells with a newly established conservation technology. First, the SMART Tube (SMART TUBE Inc.; SMT) system developed to store human blood samples for later analysis by flow cytometry was adapted for cats. Then, blood samples from 5 cats with FIP showing lymphocytosis during treatment (days 14, 28, 56, and 83 after treatment initiation) and from 5 healthy, FCoV‐negative control cats without lymphocytosis were collected and stored using SMTs. Treatment‐induced differences in lymphocyte subpopulations were analyzed by one‐way ANOVA (Bonferroni's Multiple Comparison). It was demonstrated that fixation and long‐term preservation of feline blood samples at −80°C using SMT was possible. Flow cytometry revealed that lymphocytosis during treatment was mainly attributed to a significant increase in T‐helper cells (*p* < 0.05) and B‐cells (*p* < 0.0005), but not in cytotoxic T‐cells. An increase in T‐cells is likely caused by regulatory T‐cells, which control the excessive immune response. Conversely, the increase in B‐cells suggests a period of excessive production during reduction of FCoV‐induced apoptosis. The cytotoxic T‐cell pool likely reflects the physiological distribution after recovery. Thus, the detected immune response indicates efficacy of antiviral treatment.

**Disclosures**

**Source of Funding**

**ECVIM CSF Funding**

No.

**ISCAID – International Society for Companion Animal Infectious Diseases**

## ISCAID‐O‐2

## Alpha‐1‐acid glycoprotein quantification via spatial proximity analyte reagent capture luminescence assay – Validation and application as diagnostic and prognostic marker in serum and effusions of cats with feline infectious peritonitis during GS‐441524 treatment

### M. Meli^1^, A.K. Helfer‐Hungerbuehler ^1^, A. Spiri^1^, T. Meili^1^, B. Riond^1^, D. Krentz^2^, K. Zwicklbauer^2^, K. Buchta^2^, A.M. Zuzzi‐Krebitz ^2^, K. Hartmann^2^, R. Hofmann‐Lehmann ^1^

#### ^1^University of Zurich, Clinical Laboratory Department of Clinical Diagnostics and Services, Zurich, Switzerland; ^2^LMU Munich, LMU Small Animal Clinic Centre for Clinical Veterinary Medicine, Munich, Germany

This study aimed to establish and validate a novel method to quantify alpha‐1‐acid glycoprotein (AGP) in serum/effusion samples from cats with feline infectious peritonitis (FIP) (*n* = 54/49) and compare them to sick cats with inflammatory diseases other than FIP (*n* = 39/37) and healthy cats (*n* = 17). Furthermore, serum samples from 18 FIP‐cats during GS‐441524 treatment were evaluated. AGP was measured by a spatial proximity analyte reagent capture luminescence (SPARCL^TM^) assay on the VetBio‐1 analyzer. Significant differences were assessed by non‐parametric tests: Mann–Whitney U (between 2 groups); Kruskal–Wallis one‐way ANOVA (between 3 groups) and Friedman (time course).

The assay was linear over 5 logs of two‐fold serum serial dilutions. Intra‐ and inter‐run precision was <5% and <15%, respectively. AGP was stable in serum stored for at least 8 days at room temperature, at 4°C and at ‐20°C. FIP‐cats had significantly higher AGP serum concentrations (median: 2954, range: 200–5861 μg/mL) than those with other inflammatory diseases (median: 1734, range: 305–3449 μg/mL; *p* = 0.0001) and healthy cats (median: 157, range: 78–616 μg/mL; *p* < 0.0001). AGP in effusions was significantly higher in FIP‐cats (median: 2425, range: 343–5611 μg/mL) than sick cats without FIP (median: 560, range: 83–3950 μg/mL; *p* < 0.0001). AGP concentrations in serum of GS‐441524 treated cats showed a significant decrease within the first 7 days and reached normal levels after 14 days.

The SPARCL^TM^assay offers a reliable, fast, and cost‐effective method to measure AGP concentrations in single feline serum/effusion samples. This is pivotal for diagnostics and treatment monitoring of highly sick cats.

**Disclosures**

**Source of Funding**

**ECVIM CSF Funding**

No.

**ISCAID – International Society for Companion Animal Infectious Diseases**

## ISCAID‐O‐3

## Feline coronavirus loads and presence of co‐infections in cats diagnosed with feline infectious peritonitis

### J.M. Wenk^1,2^, M.L. Meli^1,2^, C. C. De Witt^1,2^, A.B. Bouzon^1,2^, B. Pineroli^1,2^, S. Felten^3^, S.M. Meunier^3^, K. Zwicklbauer^4^, K. Hartmann^4^

#### ^1^Clinical Laboratory, Department of Clinical Diagnostics and Services, University of Zurich, Switzerland; ^2^Center for Clinical Studies, Vetsuisse Faculty, University of Zurich, Switzerland; ^3^Clinic for Small Animal Internal Medicine, Vetsuisse Faculty, University of Zurich, Switzerland; ^4^LMU Small Animal Clinic, Centre for Clinical Veterinary Medicine, Ludwig‐Maximilians University of Munich, Germany

Recent achievements allow managing feline infectious peritonitis (FIP) caused by feline coronavirus (FCoV). This study aimed to assess clinical presentation, FCoV loads, and co‐infections in 100 cats diagnosed with FIP before enrollment in a GS‐441524 treatment trial.

FCoV loads in effusion, blood, cerebrospinal fluid (CSF), organ fine‐needle aspirate (FNA), and oropharyngeal swabs were determined by reverse‐transcription quantitative polymerase chain reaction (RT‐qPCR). Cats were tested for feline leukemia virus (FeLV) antigen by point‐of‐care tests and qPCR detection, for feline immunodeficiency virus (FIV) antibodies using point‐of‐care tests and FIV Western blot. Feline calicivirus (FCV) was detected in oropharyngeal swabs by RT‐qPCR. Differences in disease severity between cats with/without co‐infection were evaluated using Mann–Whitney‐*U* test.

Of the 100 cats, 91 cats showed effusions (79 abdominal, 5 pleural, 7 bicavitary) of which 76 were procurable. Ocular and neurological signs were present in 24 and 17 cats. Effusion tested FCoV‐positive in 91/91 (median virus load (MVL) 8,162,400 copies/mL), blood in 57/91 (MVL 4275 copies/mL), FNAs in 14/19 (MVL 1,401 copies/FNA), CSF in 5/5 (MVL 522,100 copies/mL), and oropharyngeal swabs in 35/97 cats (MVL 100 copies/swab). Viral loads in blood were significantly lower than in effusion (*p* < 0.0001). Twenty‐seven cats tested FCV‐positive, 4 FIV‐positive, and 2 had progressive FeLV infection. Disease severity (Karnofsky‐Score) did not significantly differ in cats with/without FIV, progressive FeLV, or FCV infection.

In conclusion, FCoV loads vary significantly among different compartments in FIP‐cats, with highest loads in effusions. Co‐infections, e.g., with FCV infection, are frequent, and should be considered in management of FIP‐cats.

**Disclosures**

**Source of Funding**

1. Schweizerische Vereinigung für Kleintiermedizin (Swiss Association for Small Animal Medicine (SVK)) 2. Stiftung für Kleintiere der Vetsuisse Fakultät Zürich (Foundation for Small Animals of the Vetsuisse Faculty Zurich) 3. UZH Global Strategy and Partnerships Funding.

**ECVIM CSF Funding**

No.

**ISCAID – International Society for Companion Animal Infectious Diseases**

## ISCAID‐O‐4

## Assessment of cardiac changes in cats with feline infectious peritonitis

### P. Cerna^1^, E. Ross^1^, M. Willis^1^, L. Visser^1^, J. Hawley^1^, M. Lappin^1^

#### ^1^Colorado State University Department of Clinical Sciences, Fort Collins, United States

Antiviral drugs such as GS‐441524 and EIDD‐2801 have been successful in treatment of feline infectious peritonitis (FIP). As more cats are being treated with these new antiviral drugs instead of being euthanized, new disease processes that may be associated with FIP are being recognized. This includes the development myocarditis and myocardial injury, which have also been documented in humans and animals with SARS‐CoV‐2.

The objective of this study was to prospectively evaluate echocardiography and cardiac troponin I (cTnI) in cats diagnosed with FIP to better understand the prevalence of cardiac changes associated with this disease. All cats were also evaluated for other concurrent infections associated with myocarditis by testing serum for FIV antibodies, FeLV antigen, *Bartonella* spp. IgG, and *Toxoplasma gondii* IgG and IgM as well as by testing blood for nucleic acids of *Bartonella* spp. and SARS‐COV‐2.

A total of 18 client owned cats with FIP were evaluated. Evidence for co‐infections was documented in 1 cat that was *Bartonella* seropositive (1:128) but PCR negative. Assessment of chamber size from echocardiography was based on published allometric 2‐D reference intervals for cats. Increased wall thickness or chamber dilation was based on exceeding the 95% prediction intervals. The left ventricular free wall from right parasternal long axis and short axis was thickened in 5/18 and 4/18 cats, respectively. The interventricular septum from right parasternal short axis was thickened in 3/19 cats and no cats had a thickened interventricular septum from right parasternal long axis. The left atrium from the right parasternal long axis was not enlarged in any cat. The left atrium to aorta ratio from the right from the right parasternal short axis basilar view was greater than 1.6 in 3/19 cats. Pleural effusion was present in 7/18 cats (all echocardiograms were performed following thoracocentesis) and pericardial effusion in 5/18 cats (moderate in 1 cat and scant in 4 cats). The median cTnI at initial evaluation was 0.37 ng/ml (IQR 0.20–1.02; upper reference range 0.20 ng/ml). Recheck cTnI was performed at 12 weeks following institution of EIDD‐2801 therapy in 6 cats that initially had elevated cTnI; in all 6 cats, cTnI normalized to <0.20 ng/ml.

This study suggests that cats with FIP can present with elevations in cTnI and wall thickening, both of which could be suggestive of myocarditis and/or myocardial injury. Additionally, antiviral therapy may reduce active myocarditis in patients with FIP.

**Disclosures**

**Source of Funding**

**ECVIM CSF Funding**

No.

**ISCAID – International Society for Companion Animal Infectious Diseases**

## ISCAID‐O‐5

## Unlicensed antiviral treatment with GS‐441524: How are clinicians approaching feline infectious peritonitis in primary care practices?

### A. Schlachet^1^, S. Schuller^1,2^, A. Carranza Valencia^1^

#### ^1^Department of Clinical Veterinary Science, Vetsuisse Faculty, University of Bern Division of Small Animal Internal Medicine, Bern, Switzerland; ^2^Multidisciplinary Center for Infectious Diseases (MCID), University of Bern Division of Small Animal Internal Medicine, Bern, Switzerland

Feline infectious peritonitis (FIP) is now treatable, yet effective antiviral drugs remain legally unavailable in many countries. This has led to the widespread distribution of unlicensed antiviral products, mainly through Facebook groups, which also provide guidance on treatment protocols.

The aim of this retrospective study was to describe the diagnostic approach and outcome of cats with suspected FIP treated with unlicensed GS‐441524 in Western Switzerland. The data set was generously provided by a Facebook group and included signalment, clinical findings, FIP type, treatment protocol, and assessment at 4, 8, 12, 18, and 24 weeks. Additionally, each cat had a Facebook link where further information was posted.

Data from 344 cats treated with GS‐441524 between January 2021 and July 2023 were analyzed. 251 cats successfully completed the treatment (cured cats), 35 cats were still undergoing treatment, and 58 cats had died. For survival analysis, cured and dead cats were considered. Six cured cats with relevant missing data and seven dead cats with a cause of death other than FIP were excluded. The final population consisted of 296 cats: 245 were cured and 51 died, resulting in a survival rate of 82.8%.

Due to limited data on deceased cats, subsequent analysis was performed on the cured cats group. Median age was 12 months (range: 2–240), with 62% (76/123) being male. More than half were non‐pedigree cats. Cats were further categorized based on their FIP type: wet (42.4%), dry (29.4%), neurological (3.3%), ocular (2.9%), and mixed (22%). The most common clinical signs were hyporexia, weight loss, lethargy, and fever. Abdominal effusion, pleural effusion, neurological signs, and ocular signs were reported in 40% (92/230), 9.6% (22/229), 11.9% (28/236), and 9.4% (22/234) of cats, respectively.

Available workup data included hematology (92%), biochemistry profile (90%), FCoV PCR (30%), FCoV serology (22%), serum electrophoresis (18%), abdominal ultrasound (19%), and radiographs (5%). None of the cats underwent CT/MRI, CSF, or aqueous humor analysis. The most common laboratory abnormalities were hyperglobulinemia, hyperproteinemia, anemia, and hyperbilirubinemia.

The survival rate in this study is comparable to rates reported for licensed products. Most diagnoses were presumptive, indicating that clinicians rely on signalment, clinical, and clinicopathological findings rather than confirmatory tests. Given the serious implications of misdiagnosing FIP, which can lead to a patient's death or inappropriate use of antivirals, emphasizing the exclusion of differential diagnoses becomes crucial.

**Disclosures**

No disclosure.

**Source of Funding**

No funding.

**ECVIM CSF Funding**

No.

**ISCAID – International Society for Companion Animal Infectious Diseases**

## ISCAID‐O‐6

## Shorter treatment duration of 42 days with oral GS‐441524 also leads to complete remission in cats with feline infectious peritonitis

### K. Buchta^1^, A.M. Zuzzi‐Krebitz ^2^, M. Bergmann^1^, D. Krentz^1^, K. Zwicklbauer^1^, R. Dorsch^1^, G. Wess^1^, A. Fischer^1^, R. Hofmann‐Lehmann ^3^, M.L. Meli^3^, A.M. Spiri^3^, A. Hönl^1,4^, K. Matiasek^5^, Y. Zablotski^1^, L. Kolberg^6^, M. Alberer^6^, U. von Both^6^, K. Hartmann^1^

#### ^1^LMU Small Animal Clinic, LMU Munich LMU Small Animal Clinic, LMU Munich, Munich, Germany; ^2^LMU Small Animal Clinic, LMU Munich LMU, Munich, Germany; ^3^Department of Clinical Diagnostics and Services, Vetsuisse Faculty, University Department of Clinical Diagnostics and Services, Vetsuisse Faculty, University, Zurich, Switzerland; ^4^Institute of VeterinaryPathology, Section of Clinical/Comparative Neuropathology LMU Small Animal Clinic, LMU Munich, Munich, Germany; ^5^Institute of VeterinaryPathology, Section of Clinical/Comparative Neuropathology Institute of VeterinaryPathology, Section of Clinical/Comparative Neuropathology, Munich, Germany; ^6^Division of Paediatric Infectious Diseases, Dr. von Hauner Children's Hospital Division of Paediatric Infectious Diseases, Dr. von Hauner Children's Hospital, Munich, Germany

The antiviral nucleoside analogue GS‐441524 is currently the most promising feline infectious peritonitis (FIP) treatment option. So far, the recommended treatment duration is 84 days.

This study aimed to evaluate whether a shorter treatment of 42 days with oral GS‐441524 is equally effective as the 84‐day protocol.

Forty cats with FIP, diagnosed by quantitative reverse transcription polymerase reaction (RT‐qPCR) in effusion, were prospectively included and treated with 15 mg/kg GS‐441524 (BOVA Specials UK) PO q24h. The cats were randomly divided into two groups; 20/40 were treated for 84 days and 20 for 42 days. Cats were followed over 365 days with several variables routinely rechecked. Due to repeated measurements, variables were analyzed using linear mixed‐effects models, with an individual cat serving as random effect.

Of the 40 cats, 38 recovered quickly with rapid improvement in clinical and laboratory parameters, and a remarkable reduction of viral loads in effusions, blood, and feces. No significant differences with clinical relevance between the two treatment options were observed. Two cats had to be euthanized due to advanced FIP stage: one due to cardiopulmonary arrest on day 3 of treatment; the other on day 31 due to massive dyspnea caused by pleural adhesions, in this case RT‐qPCR was negative in all tissues post‐mortem.

All cats were in complete remission and remained healthy (> 1 year after the end of treatment). Orally administered GS‐441524 is highly effective, even with a shorter 42‐day treatment, thus demonstrating that a long 84‐day treatment duration is not necessary.

**Disclosures**

**Source of Funding**

**ECVIM CSF Funding**

No.

**ISCAID – International Society for Companion Animal Infectious Diseases**

## ISCAID‐O‐7

## Isolation of canine *Leptospira australis* strains from the field in view of vaccine development

### P. Bergamo^1^, L. Cupillard^2^

#### ^1^Boehringer Ingelheim Animal Health Centre de Recherche de Saint‐Vulbas, LYON, France; ^2^Boehringer Ingelheim Animal Health Laboratoire Lyon Porte des Alpes, LYON, France

Leptospirosis is a widespread zoonosis caused by spirochaetes belonging to the pathogenic species of *Leptospira* which are classified in more than 25 serogroups and more than 300 serovars. Etiological diagnosis of the disease relies principally on micro‐agglutination test (MAT), polymerase chain reaction (PCR) and molecular typing of the causative strain. Canine leptospirosis can be prevented by vaccination, with a spectrum limited to serogroups contained in the vaccine.

The aim of this field study was to set‐up and validate a robust recruitment and sampling process for optimizing the probability of isolating *Leptospira* strains, followed by serological and molecular typing of the resulting isolates, in order to select field strains of proven pathogenicity and virulence for vaccine development.

Dogs were enrolled by vet practitioners, based on clinical profiles compatible with acute leptospirosis, such as acute renal failure, acute hepatitis (both of recent and sudden onset), glucosuria without hyperglycemia, acute hemorrhagic gastro‐enteritis (non‐parvoviral), hemorrhagic syndrome. Blood and urine samples were sterilely collected and transported to a reference laboratory for MAT serology, PCR, bacterial isolation, RNA16S sequencing. Typing of the isolated strains was performed using sero‐agglutination and several molecular techniques such as variable number tandem repeat (VNTR). Of note, blood and urine were sampled for diagnostic purpose (MAT serology and PCR). The other analysis were performed on the surplus material.

Sixty four (64) sick dogs from 22 veterinary practices were included among which 46 cases had MAT and/or PCR results compatible with *Leptospira* infection, which represents a success rate close to 72%. Acute renal failure was the most commonly inclusion criterion observed in these dogs, followed by acute hepatitis. Fourteen (14) *Leptospira* field strains could be isolated, representing 22% over all included cases and 30% of the positive cases. Based on molecular typing by VNTR, 11 of the isolates were classified as serogroup Australis (serovar Bratislava) and 3 as serogroup Icterohaemorrhagiae (serovar Icterohaemorrhagiae/Verdun).

This study confirmed that implementing strict clinical case selection and sampling procedures allow high isolation rates of *Leptospira* field strains. It also outlined the value of combining different testing methods when facing a suspicion of leptospirosis and the usefulness of implementing molecular typing methods for epidemiological purposes. Finally, this kind of study is of utmost importance when it comes to identifying and isolating relevant strains circulating in the field, before considering to include them in vaccines and to use them for challenge studies.

**Disclosures**

Pierre BERGAMO and Lionel CUPILLARD are Boehringer Ingelheim Animal Health employees.

**Source of Funding**

Boehringer Ingelheim Animal Health.

**ECVIM CSF Funding**

No

**ISCAID – International Society for Companion Animal Infectious Diseases**

## ISCAID‐O‐8

## Comparison of 5 different Leptospira spp. antibody point‐of‐care test results in healthy dogs after vaccination against leptospirosis

### K.G. Schmitt^1^, M. Bergmann^1^, Y. Zablotski^1^, K. Hartmann^1^

#### ^1^LMU Small Animal Clinic, Munich, Germany

Canine leptospirosis is a serious disease with zoonotic potential. Early diagnosis is therefore important. Point‐of‐care (POC) tests provide fast results; however, their outcome after *Leptospira* vaccination is unclear. This study compared results of 5 POC tests detecting *Leptospira* antibodies after vaccination.

Five POC tests were evaluated in 582 serum samples from 97 healthy dogs (immunized with 4‐serovar‐vaccines 1 year previously) before revaccination and after 2, 4, 12, 26, 52 weeks. Three tests detected immunoglobulin M (IgM) antibodies ((1) WITNESS® Lepto, Zoetis; (2) FASTest® Leptospira IgM, MegaCor; (3) Test‐it^TM^Leptospirosis, Life Assay), one immunoglobulin G (IgG) antibodies ((4) ImmunoComb® Canine Leptospira Test Kit, Biogal) and one both ((5) SNAP® Lepto, IDEXX). Logistic regression and Tukey's correction for multiple testing were applied.

Before vaccination, few IgM‐POC tests were positive ((1) 1/97; (2) 1/97; (3) 0/97)), but 32/97 IgG‐POC (4) and 13/97 IgM/IgG‐POC (5) tests. More dogs became positive after vaccination with increasing numbers until week 4 (week 2: (1) 18/97, (2) 13/97, (3) 1/97, (4) 81/97, (5) 43/97; week 4: (1) 10/97, (2) 7/97, (3) 1/97, (4) 78/97, (5) 34/97), and declined thereafter. Most IgM‐POC tests were negative by week 12. Significant differences were found between IgM‐POC and the other 2 POC tests (*p*‐values ≤ 0.0085).

IgM‐POC tests become positive in only some dogs (1% to 19%) after vaccination and might therefore be more useful than other tests for diagnosing *Leptospira* infection in not recently vaccinated dogs. Test (3) did not react after vaccination and further studies of its sensitivity are required.

**Disclosures**

The leptospirosis vaccine (Nobivac® L4) was provided by MSD Animal Health/Intervet International B.V.

**Source of Funding**

MSD Animal Health/Intervet International B.V., Boxmeer, The Netherlands.

**ECVIM CSF Funding**

No.

**ISCAID – International Society for Companion Animal Infectious Diseases**

## ISCAID‐O‐9

## Serovar Australis replaces serovar Copenhageni as the most common serovar causing canine leptospirosis in New South Wales, Australia: A case series (2021–2023)

### C. Griebsch^1^, N. Kirkwood^1^, M. Ward^1^, J. Norris^1^

#### ^1^Sydney School of Veterinary Science, The University of Sydney, Sydney, Australia

After apparent absent of disease, highly fatal canine leptospirosis emerged in New South Wales (NSW), Australia in 2017 with 17 cases diagnosed in urban Sydney dogs between 2017 and 2020. Based on results of microscopic agglutination testing (MAT), serovar Copenhageni appeared to be the causative serovar in most however not all clinical cases. If deemed necessary, dogs are vaccinated with the registered monovalent vaccine in Australia containing serovar Copenhageni.

Aims of the study were to determine the geospatial distribution, clinicopathologic and serological characteristics of canine leptospirosis in NSW from January 2021 to December 2023 to establish if the currently available vaccine offers sufficient protection.

Cases were identified following referral or direct contact from referring veterinarians. Dogs were included if clinical and clinicopathological findings confirmed leptospirosis.

Between 2021 and 2023 leptospirosis has been confirmed in 61 dogs in NSW. While cases continued to be identified in greater Sydney, in 2022 there were two major outbreaks in the LGA of Shoalhaven (*n* = 23) and in the LGA of Lake Macquarie (*n* = 7) located approximately 140km South and North of Sydney respectively. The most common serovar identified was Australis (*n* = 23) followed by Copenhageni (*n* = 8), Pomona (*n* = 2), Robinsoni (*n* = 2) and Bratislava (*n* = 1). In all cases from the LGA of Shoalhaven in which serology was performed serovar Australis was identified (*n* = 16/18), two dogs tested negative. Overall, 95% of dogs had renal, 63% hepatic, 8% haemorrhagic and 5% pulmonary involvement. Three dogs had non azotaemic glucosuria. The case fatality rate was 40%.

The apparent emergence of serovar Australis as the main causative serovar in dogs in NSW highlights the need for bi‐ or multivalent vaccines. Ongoing case surveillance is needed to determine if other serovars are of clinical importance. The presence of glucosuria should prompt leptospirosis testing in endemic areas.

**Disclosures**

Acknowledgements This study received philanthropic funding from the University of Sydney (Betty and Keith Cook Bequest, Schnakenberg Bequest) and the Sydney Institute for Infectious Diseases (Sydney ID). Conflict of Interest The authors do not have any conflicts of interest to declare.

**Source of Funding**

This study received philanthropic funding from the University of Sydney (Betty and Keith Cook Bequest, Schnakenberg Bequest) and the Sydney Institute for Infectious Diseases (Sydney ID).

**ECVIM CSF Funding**

No.

**ISCAID**—**International Society for Companion Animal Infectious Diseases**

## ISCAID‐O‐10

## Potential leptospiral shedding in vaccinated and asymptomatic pack hounds: A prospective study in 56 dogs

### L. Lecot^1^, J. Gros^2^, F. Ayral^3^, Z. Djelouadji^3^, M. Le Guyader^3^, M. Hugonnard^1^

#### ^1^VetAgro Sup Département des animaux de compagnie de loisir et de sport, Marcy‐l'Étoile, France; ^2^VetAgro Sup, Marcy‐l'Étoile, France; ^3^VetAgro Sup Département élevage et santé publique vétérinaire, Marcy‐l'Étoile, France

Asymptomatic carriage of leptospires in dogs living in a pack may pose a risk of contamination for humans and congeners. The aim of this prospective study was to determine the rate of asymptomatic carriage of pathogenic leptospires in vaccinated pack hounds and to monitor their carriage status over time.

A total of 56 apparently healthy adult dogs were enrolled including 55 Grand Anglo Français and 1 Fox Hound. The fox‐hunting dogs were living in a kennel that was open to a grassy yard, next to forest and ponds, in direct contact to wildlife. All dogs had received a quadrivalent vaccine preventing urinary shedding after contamination by Icterohaemorrhagiae, Canicola and Australis. Micro‐Agglutination Test (MAT) including 12 serogroups and real time polymerase chain reaction (PCR) analysis on blood were performed for each dog. Real time PCR was performed on urine from a subset of dogs for which urine could be obtained by free catch (*n* = 32). After eleven months, PCR analyses were repeated on blood and urine from PCR‐positive dogs still present in the pack. All positive PCR results were confirmed by conventional 16s rRNA PCR and typing was performed using 16s gene sequencing.

Ten of 32 dogs tested (31%) had positive PCR results on urine (2 with maximal MAT titers of 1:1600 for Australis and 8 with all MAT titers < 1:200). One of 56 dogs (2%) had positive PCR results on blood and undetectable MAT titers. Genomic species could be identified in 9/11 cases (*L. borgpetersenii* in 5 cases; *L. interrogans*, including leptospires of serogroup Australis, in 4 cases). Two of 4 dogs positive for *L. interrogans* were seropositive for Australis. Among the whole population, the seroreactivities were mainly directed against *Leptospira* serogroups Australis and Sejroe. Eleven months later, 8/11 PCR‐positive dogs were still in the pack and 2 of them were still PCR‐positive on their urine. The dog with a blood‐positive PCR at first sampling had left the pack.

This study suggests that the pack hounds although healthy may carry and shed pathogenic leptospires, including those belonging to Australis, despite appropriate vaccination with a tetravalent vaccine. The protection offered by the tetravalent vaccine against urinary shedding is therefore uncertain. The pathogenic leptospires DNA found in urine was not an evidence of the excretion of viable bacteria. Further studies are needed including leptospiral urine cultures to clarify the signification of these preliminary results.

**Disclosures**

**Source of Funding**

**ECVIM CSF Funding**

No.

**ISCAID – International Society for Companion Animal Infectious Diseases**

## ISCAID‐O‐11

## Giardia assemblages with zoonotic potential are more commonly found in cats than in dogs in fecal molecular diagnostic test samples collected from the US, Canada, Asia, and Brazil

### M. Evason^1^, P. Jimenez Castro^1^, S. Miyashiro^2^, O. Valerio^2^, M. Baranger‐Ete ^1^, C. Savard^1^, C. Leutenegger^1^

#### ^1^MARS Petcare Science & Diagnostics, Fountain Valley, United States; ^2^Technology in Animal Health, Belo Horizonte, Brazil

*Giardia* is one of the most prevalent gastro‐intestinal (GI) parasites. The common dog and cat assemblages C, D, and F are not associated with human illness, but *Giardia* assemblages A and B have zoonotic potential. Routine GI parasite screening with ova and parasite identification, or ELISA technology for *Giardia* antigen detection, are used in the reference laboratory setting; however, neither test differentiates *Giardia* at the assemblage level. In contrast, molecular methods such as real‐time PCR (qPCR) can be used for assemblage and genotype identification, allowing for determination of *Giardia* zoonotic potential.

The aim of the study was to describe *G. duodenalis* screening test and assemblage A&B identification, as detected by a broad (20 GI parasites), commercially available, qPCR panel in dog and cat populations from the United States (US), Canada, Asia, and Brazil.

A selection of dog and cat fecal samples submitted from the United States (US) (*n* = 770,250), Canada (*n* = 5,818), Asia (*n* = 1,673), and Brazil (*n* = 333) were evaluated by qPCR following specimen retention release. *G. duodenalis* and assemblages A&B prevalence were calculated for both dogs and cats.

Overall, qPCR detected *Giardia duodenalis* at higher prevalence in dogs than in cats in all geographies. In the US, *G. duodenalis* prevalence in dogs was 13.8% (94,343/653,714), and 7.1% (8,837/116,536) in cats. In Canada, prevalence in dogs was 18.1% (697/3,846) and 8.2% (162/1972) in cats. In Asia, prevalence in dogs was 10.6% (57/539) and 10.1% (114/1,134) in cats. In Brazil, prevalence in dogs was 27.0% (55/204) and 33.3% (43/129) in cats. When *Giardia* assemblages with zoonotic potential were evaluated, the prevalence in cats was higher than in dogs in all regions. In US dogs, the *Giardia* assemblage A&B prevalence was 2.0% (1,873/94,343), and 17.9% (1,584/8,837) in cats. In Canada, prevalence in dogs was 1.9% (13/697) and 27.8% (45/162) in cats. In Asia, prevalence in dogs was 1.8% (1/57) and 13.2% (15/114) in cats. Prevalence in dogs was 0.0% (0/55) and 18.6% (8/43) in cats from Brazil.

These data describe a lower *Giardia duodenalis* prevalence in cats as compared to dogs in all geographies. In all regions, a higher prevalence of *Giardia* assemblages with zoonotic potential was observed in cats, with detection as much as 14.6 times higher (Canada). Molecular diagnostics possess the ability to identify key genetic differences. This study highlights the utility of zoonotic risk potential detection that may influence clinical decision‐making, antimicrobial stewardship, and as part of One Health.

**Disclosures**

Michelle D. Evason1 Pablo D. Jimenez Castro1 Samtha Miyashiro2 Otavio Valerio2 Myriam Baranger‐Ete1 Christian Savard1 Christian M. Leutenegger1 Authors employed by, (1) Antech Diagnostics, Inc., a part of the Mars Petcare Science & Diagnostics, or (2) TECSA Technology in Animal Health, Belo Horizonte, Brazil.

**Source of Funding**

**ECVIM CSF Funding**

No.

**ISCAID – International Society for Companion Animal Infectious Diseases**

## ISCAID‐O‐12

## Evaluation of attitudes and behaviours related to antimicrobial prescribing in canine acute diarrhoea using discrete choice experiments

### T. Sørensen^1^, F. Allerton^2^, S. Weese^3^, L. Jessen^1^, D. Timofte^4^, F. Bagcigil^5^, E. Broens^6^, M. Nolff^7^, D. Singleton^8^, D. Verwilghen^9^, J. Buckell^10^, K. Powels^10^

#### ^1^University of Copenhagen Dept. of Veterinary Clinical Sciences, Frederiksberg, Denmark; ^2^Willows Veterinary Centre & Referral Service, West Midlands, United Kingdom; ^3^University of Guelph Dept of Pathobiology and Centre for Public Health and Zoonoses, Guelph, Canada; ^4^University of Liverpool Department of Veterinary Anatomy, Physiology and Pathology, Liverpool, United Kingdom; ^5^Istanbul University‐Cerrahpaşa Department: Department of Microbiology, Istanbul, Turkey; ^6^Utrecht University Department of Biomolecular Health Sciences, Utrecht, Netherlands; ^7^University of Zürich Clinic for Small Animal Surgery, Zürich, Switzerland; ^8^IVC Evidensia, Bristol, United Kingdom; ^9^University of Sydney School of Veterinary Sciences, Camperdown, Australia; ^10^University of Oxford Nuffield Department of Population Health, Oxford, United Kingdom

Prescribing behaviour affects antimicrobial resistance (AMR) levels and in order to avoid unnecessary prescriptions we need to understand the drivers for prescribing antimicrobials among first opinion veterinarians. The aim of this study was to investigate veterinarians’ attitudes and behaviours related to antimicrobial treatment in canine acute diarrhoea.

An online discrete choice experiment (DCE) was developed in seven languages. The background questions related to the practice, attitudes and behaviours related to AMR. The experiment consisted of six experimental scenarios choosing between prescribing antibiotics or not. The scenarios were described by several varying attributes: clinical signs, expected duration of diarrhoea after consultation and client pressure.

In total, 853 veterinarians from 62 countries across all five World Organisation for Animal Health (WOAH) regions participated. The choice not to prescribe antibiotics was most common. Prescription of antibiotics was more likely when: 1) diarrhoea was haemorrhagic; 2) expecting longer duration of diarrhoea without antibiotics and 3) the anticipated client pressure for antibiotics. Antibiotic prescription was also linked to veterinarians’ demographics with antimicrobial prescription associated with male gender, more years in practice, lower knowledge or concern for AMR, or if veterinarians were from Asia, South America, and Africa compared to Europe. No differences in antibiotic prescription probability were observed between Europe, North America, and Australasia.

The study shows that both geographical, client‐related and patient‐related factors drive antimicrobial prescribing choices and highlights the need for multifaceted stewardship interventions.

**Disclosures**

**Source of Funding**

WALTHAM™ Foundation Research Award.

**ECVIM CSF Funding**

No.

**ISCAID – International Society for Companion Animal Infectious Diseases**

## ISCAID‐O‐13

## Poor awareness amongst residents in the United Kingdom (UK) of the canine and human health risks associated with imported puppies carrying non‐endemic diseases

### Z. Belshaw^1^, M. Lord^2^, C. Whelan^3^, R. Packer^4^

#### ^1^EviVet Research Consultancy, Nottingham, United Kingdom; ^2^Co‐Evolve, Bristol, United Kingdom; ^3^Merkle EMEA Merkle EMEA, London, United Kingdom; ^4^Royal Veterinary College Clinical Sciences and Services, London, United Kingdom

Over 250,000 dogs entered the United Kingdom (UK) from mainland Europe in 2022, driven by residents' wishes to own or “rescue” European dogs, and illegal puppy trading. There is mounting evidence that these dogs are importing non‐endemic diseases, but little research has been conducted into UK residents’ awareness of this. This study aimed to assess UK residents’ concerns, knowledge and information sourcing regarding diseases linked to canine importation.

A 40‐question, five‐part questionnaire was administered via SurveyMonkey during May–June 2023. All UK residents aged 18 years and over were eligible to participate. Data were collected on participants’ demographics, dog ownership history, and canine‐related employment. Multiple‐choice questions ascertained their concern level, and information sources, about canine imported diseases. Participants were then asked if they had heard of seven non‐endemic diseases: rabies, brucellosis, leishmaniasis, babesiosis, ehrlichiosis, hepatozoonosis and dirofilarial heartworm. For each disease they had heard of, participants were asked whether an infected imported puppy could potentially infect a UK‐based dog, or human. Descriptive data were analysed in Microsoft Excel and linear regression was performed in R Studio (version 4.3.2) with the outcome the number of correct responses. Significance was set at 5%. No questions were mandatory so *n* = varies.

Useable responses were collected from *n* = 7184 people, with a 48.0% completion rate. Many participants (*n* = 3375/5455; 62.0%) were very concerned about canine imported diseases, with only *n* = 110/5455 (2.0%) reporting no concerns. However, *n* = 491/5455 (9.0%) reported no awareness. Animal charities (*n* = 2019/4562; 44.2%) and social media posts (*n* = 1879/4562; 41.2%) were the commonest information sources where participants recalled learning about imported diseases. Most had heard of rabies (*n* = 5350/5292; 99.2%) and dirofilarial heartworm (*n* = 4587/5395; 85.0%). Over half had heard of brucellosis (*n* = 3490/5374; 64.9%) and leishmaniasis (*n* = 2908/5381; 54.0%). Fewer had heard of babesiosis (*n* = 1828/5351; 34.2%), ehrlichiosis (*n* = 1427/5348; 26.7%) or hepatozoonosis (*n* = 1235/5354; 23.1%). Respondents consistently demonstrated greater awareness of transmission risks to UK‐resident dogs than zoonotic risks to humans, for example, rabies risk was correctly answered for dogs: *n* = 5061/5256 (96.3%) vs. humans: *n* = 4693/5264 (89.2%); ehrlichiosis risk was correctly answered for dogs: *n* = 918/1383 (66.4%), three‐times more than for humans: 296/1371 (21.5%). Multivariable linear regression identified higher odds of answering disease‐risk questions correctly if respondents had received job‐specific training in imported diseases, worked with imported dogs, had learnt about imported diseases through UK Government information or via friends, or had taken a dog abroad.

This study demonstrates worrying knowledge gaps relating to awareness of, and risks from, non‐endemic canine diseases amongst UK residents.

**Disclosures**

Our research group has received funding for related research into the pandemic puppy phenomenon from Battersea Dogs and Cats Home, the British Veterinary Association and the Society of Companion Animal studies. Dr Belshaw has independently received funding in the past for research and consultancy work from Hills, Dechra, Boehringer Ingelheim, Zoetis, Clinician's Brief, Veterinary Record and the BBSRC. We do not believe these disclosures are relevant to the abstract submitted.

**Source of Funding**

Research England Policy Support Fund (PSF), administered by the Royal Veterinary College, London.

**ECVIM CSF Funding**

No.

**ISCAID – International Society for Companion Animal Infectious Diseases**

## ISCAID‐O‐14

## Effectiveness of the implementation of a revised hygiene concept on hand hygiene compliance and colonisation of hospitalized cats and dogs with multidrug‐resistant organisms

### C. Mauron^1^, J. Horlbog^2^, A. Collaud^3^, A. Young^4^, R. Stephan^2^, M. Biggel^2^, H. Rohrbach^5^, B. Willi^6^, V. Perreten^3^, S. Schuller^1^

#### ^1^Vetsuisse Faculty, University of Bern Department of Clinical Veterinary Medicine, Bern, Switzerland; ^2^Vetsuisse Faculty, University of Zürich Institute for Food Safety and Hygiene, Zürich, Switzerland; ^3^Vetsuisse Faculty, University of Bern Institute of Veterinary Bacteriology, Bern, Switzerland; ^4^School of Agricultural, Forest and Food Sciences, Bern University of Applied Sci Food Business and Marketing Division, Zollikofen, Switzerland; ^5^Vetsuisse Faculty, University of Bern, Bern, Switzerland; ^6^Vetsuisse Faculty, University of Zürich Clinic for Small Animal Internal Medicine, Zürich, Switzerland

The emergence and spread of multidrug‐resistant organisms (MDRO) represent a threat to human and animal health. The aims of this study were to assess the effectiveness of improved infection prevention and control (IPC) measures in a small animal referral hospital on staff hand hygiene compliance (HHC) and MDRO colonisation in dogs and cats.

A structured IPC audit including direct observation of staff hand hygiene compliance (HHC) were conducted in 2018 (pre‐IPC implementation) and again in 2021, after implementation of infrastructural, procedural, and educational measures to improve IPC. Oronasal and rectal swabs were collected at admission form 147 dogs and 63 cats of which 105 dogs and 33 cats (65%) were sampled again at discharge from the hospital. Methicillin‐resistant Staphylococcaceae (MRS), 3rd generation cephalosporin‐resistant (3GCR) and carbapenemase‐producing Enterobacterales (CPE) were isolated using selective media. Species identification was performed by MALDI‐TOF mass spectrometry, antimicrobial resistance was determined by disk diffusion and CPE‐resistance gene were detected by PCR. Whole genome sequencing (WGS) was performed to confirm resistance genes and determine genetic relatedness of CPE isolates.

HHC of 190 employees was observed (645 HH opportunities) in 4 hospital areas. Overall HHC remained low at 30.7% (CI 27.1–34.3). HHC was higher in veterinarians compared to nurses and students (*p* = 0.021), and in the emergency room compared to general examination or pre‐operation rooms (*p* = 0.003). The admission prevalence of MDRO carriage in pets was 12.3% (95% CI, 7.8–16.8). The discharge prevalence and acquisition rates were 35.5% (49/138; 95% CI 27.4–43.6) and 33.3% (46/138, 95% CI 25.4–41.3), respectively. There was no significant difference in colonisation rates pre‐ and post IPC implementation.

Predominant hospital‐acquired isolates were *Escherichia coli* (*n* = 33, 36%)*, Enterobacter hormaechei/cloacae* (*n* = 25, 27%) and *Klebsiella variicola* (*n* = 14, 15%). Of these, 80% were resistant to ≥3 classes of antimicrobials, 63% were 3GCR and 47% carbapenemase‐producing. WGS revealed 14 CPE clusters, the largest being *E. hormaechei* ST418 *bla*_OXA‐48_ (*n* = 17/97, 17.5%) and *K. variicola* ST1270 *bla*_OXA‐48_ (*n* = 18/97, 18.5%). Six dogs acquired MDR gram‐positive bacteria (7%) including 2 MRSA. The majority of the isolates in the clusters were acquired by patients during hospitalization, indicating in‐hospital transmission rather than *de novo* selection.

Despite advances in IPC implementation, veterinary hospitals may play a significant role in the selection and transmission of MDROs amongst veterinary patients. Measures to increase HHC require a continuous effort to be effective.

**Disclosures**

**Source of Funding**

This study was financed by the Swiss Federal Food Safety and Veterinary Office (FSVOGrant no. 1.21.q “Effect of the implementation of infection prevention and control concepts and hand hygiene campaigns in companion animal clinics in Switzerland”).

**ECVIM CSF Funding**

No.

**ESVONC – European Society of Veterinary Oncology**

## ESVONC‐O‐1

## A phase 2, single‐arm, open‐label clinical trial on adjuvant peptide‐based vaccination in dogs with biologically aggressive melanoma undergoing tumor excision and lymphadenectomy

### E. Faroni^1^, S. Sabattini^1^, L. Tiraboschi^2^, M. Aralla^3^, I. Maga^1^, F. Massari^4^, M.L. Bianchi^4^, M. Rescigno^2^, L. Marconato^1^

#### ^1^Department of Veterinary Medical Sciences, University of Bologna, Ozzano dell'Emilia, Italy; ^2^IRCCS Humanitas Research Hospital, Rozzano, Italy; ^3^Pronto Soccorso Veterinario Laudense, Lodi, Italy; ^4^Clinica Veterinaria Nervianese, Nerviano, Italy

Melanoma is common in dogs, and its biological behavior depends on the primary tumor site and histologic features. For biologically aggressive melanoma, surgery, including excision of the tumor and lymphadenectomy, is not curative due to the rapid development of distant metastases. Conventional chemotherapy has shown limited efficacy, prompting the need for new anticancer strategies. Immunotherapy has recently gathered attention in veterinary medicine, showing promising results in several studies involving various tumors.

In this trial, dogs with biologically aggressive melanoma of any anatomical site undergoing surgery were administered a heterologous vaccine derived from the secretome of canine melanoma cells, enriched in ER‐stress peptides, to assess its efficacy and tolerability.

Dogs underwent complete staging, and those without distant metastases were considered for enrollment. Following tumor excision and regional/sentinel lymphadenectomy, dogs were included only after confirming the diagnosis of a biologically aggressive melanoma, defined as the presence of nodal metastasis and/or a Ki‐67 index of ≥15% or ≥19.5 positive nuclei per 1 cm^2^grid area for cutaneous or oral/lip melanoma, respectively. The included dogs received a monthly vaccination for a total of 6 doses and were followed‐up with serial physical exams and imaging. A historical group of dogs undergoing excision of a biologically aggressive melanoma and regional/sentinel lymphadenectomy, whether treated with adjuvant chemotherapy or not, served as control.

Twenty‐four dogs were vaccinated, and 23 were included in the control group. The two groups were well‐balanced regarding signalment, anatomic site, imaging method used for staging, completeness of surgical resection, and presence of nodal metastasis at surgery. Among vaccinated dogs, 22 (91.7%) completed immunotherapy, while 2 did not due to cancer‐related death. No adverse events were registered. Median time to metastasis (TTM) was 312 days (95% CI, 1–717) and 167 days (95% CI, 59–275) for vaccinated and control dogs, respectively (*P*=0.026). At data analysis closure, 9 (37.5%) vaccinated dogs were still alive with a median follow‐up of 539 days (range, 239–1029), while 15 (62.5%) were dead for tumor‐related (*n* = 13) or unrelated (*n* = 2) causes. One (4.3%) unvaccinated dog was alive at 704 days from surgery, while 22 (95.7%) were dead for tumor‐related (*n* = 21) or unrelated (*n* = 1) causes. Median overall survival (OS) was 505 days (95% CI, 323–627) for vaccinated dogs and 190 days (95% CI, 148–232) for control dogs (*P* < 0.001).

This peptide‐based vaccine against canine melanoma was well‐tolerated, and significantly increased TTM and OS. Active immunotherapy shows promise for changing and improving melanoma management in dogs.

**Disclosures**

**Source of Funding**

**ECVIM CSF Funding**

No.

**ESVONC – European Society of Veterinary Oncology**

## ESVONC‐O‐2

## CD47 in canine round‐cell tumors: an explorative flow cytometric assessment

### A. Ubiali^1^, P. Moretti^2^, M. Luciani^3^, F. Montesi^3^, L. Marconato^4^, D. Stefanello^2^, V. Martini^2^

#### ^1^Department of Veterinary Medicine and Animal Sciences, University of Milan Department of Veterinary Medicine and Animal Sciences, Lodi, Italy; ^2^Department of Veterinary Medicine and Animal Sciences, University of Milan, Lodi, Italy; ^3^MYLAV Veterinary Diagnostic Laboratory, Rho, Italy; ^4^Department of Veterinary Medical Sciences, University of Bologna, Ozzano Dell'Emilia (BO), Italy

It is nowadays well established that CD47 protein, a molecule involved in the "don't eat me" signal inhibiting phagocytosis by macrophages, is expressed by various human cells. Several studies reported increased expression in hematological malignancies, and its role in immunotherapy is well known. However, there is currently no information available on its presence in canine neoplasms. The aim of this study is to assess the presence of CD47 on the surface of neoplastic cells in canine round cell tumors using flow cytometry (FC).

Samples obtained for diagnostic purposes from dogs diagnosed with mast cell tumors (MCTs), lymphomas or leukemia were included. The surplus material of cellular suspension (from enlarged lymph nodes for lymphoma cases and from the nodule for MCTs) or peripheral blood (for leukemia cases) was tested for CD47 expression via FC. Samples were considered positive if ≥20% of neoplastic cells stained positive for CD47.

A total of 120 neoplasms were analyzed, including 85 lymphomas (53 B‐cell, 20 T‐cell, 12 T‐zone Lymphoma), 15 MCTs (12 cutaneous, 2 subcutaneous, 1 not reported) and 20 leukemias (8 chronic lymphocytic, 1 acute lymphoblastic, 1 acute myeloid, 1 chronic myeloid, 9 acute undifferentiated). Positive samples accounted for 27/85 (32%) lymphomas, 15/20 (75%) leukemias, and 12/15 (80%) MCTs. A significant difference in the prevalence of positive samples was found among different diagnoses, with leukemias and MCTs reporting a larger number of positive samples than lymphomas. Conversely, no statistical differences in CD47 expression were found among lymphoma subtypes, between acute and chronic leukemias and among MCT based on grading according to Patnaik/Kiupel system, nor based on lymph node metastatic status.

Based on our results, CD47 is expressed by canine round cell tumors and can be accessed via FC, with higher rates observed in leukemias and MCTs compared to lymphomas. The lack of significant differences among subclasses within each diagnostic group should be interpreted with caution, given the limited number of samples included in some groups, possibly influencing the results. As CD47 is widely expressed, it may be included in various panels for immune‐checkpoint assessment in round‐cell neoplasms, and its prognostic value warrants further investigation.

**Disclosures**

**Source of Funding**

**ECVIM CSF Funding**

No.

**ESVONC – European Society of Veterinary Oncology**

## ESVONC‐O‐3

## Evaluation of risk factors for the development of canine Diffuse Large B‐Cell Lymphoma in northern Italy

### I. Maga^1^, E. Faroni^1^, L. Marconato^1^, R. Zaccone^1^, A. Rubini^1^, S. Sabattini^1^

#### ^1^Department of Veterinary Medical Sciences, University of Bologna, Ozzano dell'Emilia, Italy

Canine lymphoma is a widely recognized comparative model for human non‐Hodgkin lymphoma, with Diffuse Large B‐Cell Lymphoma (DLBCL) being the most frequent histotype. In humans, several environmental risk factors have been recognized, including exposure to chemical agents and ionizing radiation. In dogs, few epidemiologic studies have been conducted, with breed, exposure to chemical agents, and proximity to industrial areas reported as potential contributing factors. However, studies mostly focused on demographic features alone or a restricted number of factors, and they assessed heterogeneous groups of dogs with different lymphoma histotypes.

The aim of this study was to explore the influence of a broad spectrum of demographic and environmental factors on the development of canine DLBCL in the highly industrialized regions of northern Italy.

A retrospective, observational, case‐control study was conducted. Owners of dogs with DLBCL were asked to complete an anonymous questionnaire including socio‐demographic, environmental and lifestyle information. The same questionnaire was distributed to the control group, consisting of owners of dogs aged at least 8 years old and with no history of lymphoma. The association between DLBCL and potential risk factors was assessed through logistic regression analysis.

Questionnaires were retrieved from the owners of 72 dogs with DLBCL and 200 controls. The two groups were well balanced in terms of owners’ socio‐demographic characteristics. Weight >10 kg, intact reproductive status, exposure to electromagnetic fields, drinking non‐mineral water, exposure to second‐hand smoke, and living in rural areas were associated with an increased risk of DLBCL at univariable analysis. Risk factors significantly associated with an increased risk of DLBCL at multivariable analysis included weight >10 kg (OR, 5.02; 95% CI, 1.87–13.48; *P* < 0.001), exposure to second‐hand smoke (OR, 1.96; 95% CI, 1.01–3.77; *P*=0.045), and exposure to electromagnetic fields (OR, 1.80; 95% CI 1.01–3.21; *P*=0.048).

Weight emerged as the strongest risk factor, likely related to the well‐documented prevalence of lymphoma among medium to large‐sized dog breeds. Exposure to electromagnetic fields has been linked to human lymphoma and childhood leukaemia, and it was also suggested as a risk factor in a previous study in dogs. In contrast, second‐hand smoke has not been previously associated with canine DLBCL, despite already being recognized as a known risk factor for several tumour types in both dogs and cats.

This is the first study investigating the potential risk factors specifically associated to canine DLBCL. Data analysis confirms some of the previously reported risk factors and suggests the potential role of new ones.

**Disclosures**

**Source of Funding**

**ECVIM CSF Funding**

No.

**ESVONC – European Society of Veterinary Oncology**

## ESVONC‐O‐4

## Clinico‐hematological profile and outcomes of acute leukemias/myelodysplastic syndromes in cats: A retrospective study of 24 cases

### K. Mourou^1^, C. Duperrier‐Simond ^1,2^, L. Barrett^3^, J.L. Cadoré ^1^, M. Hugonnard^1^, L. Lecot^1^, E. Ramery^4^, E. Krafft^1^

#### ^1^VetAgro Sup Internal Medicine, Marcy‐l'Etoile, France; ^2^Oniris Internal Medicine, Nantes, France; ^3^VetAgro Sup Oncology, Marcy‐l'Etoile, France; ^4^VetAgro Sup Clinical Pathology, Marcy‐l'Etoile, France

Acute leukemias (AL) and myelodysplastic syndromes (MDS) represent a heterogeneous group of rare hematological malignancies, with little existing literature in cats.

The objectives were to describe the clinicopathologic features and outcomes of feline AL and MDS diagnosed between 2009 and 2023. Survival analysis was conducted using Kaplan–Meier curves.

Twenty‐four cats were included (median age 6.5 years [0.8–18], males 67%): 19 diagnosed with AL (myeloid [*n* = 16], lymphoid [*n* = 3]) and 5 with MDS. Clinical signs were mostly non‐specific; half had fever. Chief hematological abnormalities included anemia in 23/24 (non‐regenerative [*n* = 20], macrocytic [*n* = 8]), thrombocytopenia in 17/24, circulating nucleated red blood cells in 17/24, and circulating blast cells in 15/19 cats with AL (often counted as monocytes by the analyzers). Bi‐ and pancytopenia were present in 58 and 13% of cases, respectively. FeLV infection was documented in 5/21 cats. Four cases met the diagnostic criteria for IMHA. Extramedullary leukemic infiltration was confirmed in 9 cases (spleen [*n* = 7], liver [*n* = 4], lymph nodes [*n* = 3], joints, central nervous system, and skin [*n* = 1, each]). Median survival time was 6 days [1–40]. Treatments included blood transfusions (16/24), corticosteroids (17/24) and/or chemotherapy (6/24), without significant effect on survival.

This study confirmed that AL/MDS present with vague clinical signs and carry a poor prognosis in cats. FeLV infection was less common than previously reported. While easily suspected in cases with multiple cytopenias and/or circulating blast cells, AL/MDS may present with inconspicuous hematological abnormalities. In such cases, investigation of extramedullary organ involvement, frequently observed in this population, may aid in diagnosis.

**Disclosures**

**Source of Funding**

**ECVIM CSF Funding**

No.

**ESVONC – European Society of Veterinary Oncology**

## ESVONC‐O‐5

## Tolerability and short term efficacy of intra‐arterial vinblastine for the treatment of naturally occurring lower urinary tract carcinomas in dogs

### O. Vigouroux^1^, A. Horton^2^, J. O'Keeffe^3^, G. McLauchlan^3^

#### ^1^AURA Veterinary Internal medicine, Guildford, United Kingdom; ^2^Royal Surrey Hospital Interventional Radiology, Guildford, United Kingdom; ^3^AURA Veterinary, Guildford, United Kingdom

Lower urinary tract (LUT) carcinomas account for >2% of all canine tumours and are associated with a guarded prognosis. Standard treatment includes intravenous chemotherapy, nonsteroidal anti‐inflammatory drugs (NSAIDS) and, when possible, surgery.

Targeted intra‐arterial (IA) chemotherapy involves delivery of a cytotoxic agent directly to the arterial supply of the tumour with the aim of increasing local drug delivery to the neoplasm while potentially sparing healthy tissue. The use of IA chemotherapy has been reported in both human and veterinary patients with LUT carcinomas.

The aim of this study was to report the tolerability (<7 days) and short‐term efficacy of IA vinblastine for the treatment of naturally occurring LUT carcinomas in dogs.

Dogs diagnosed with LUT carcinomas that received IA vinblastine between 2016 and 2023 were included. Fourteen dogs met the inclusion criteria and fifteen procedures were performed. Adverse events were recorded during the peri‐operative period and a time up to first review (day 7) and classified according to the Veterinary Cooperative Oncology Group criteria (VCOG‐CTCAE V2). Clinical response was evaluated at day 14 before administration of the second dose of vinblastine. When available, diagnostic imaging restaging was reviewed and dogs were classified as having progressive disease (PD), stable disease (SD), partial remission (PR) and complete remission (CR) according to the Response Evaluation Criteria for Solid Tumour (RECIST).

Of the patients included 8/14 had a urinary bladder carcinoma and 6/14 a prostatic carcinoma. Vinblastine was successfully administered via the internal iliac arteries in all cases. Peri‐operative complications included the development of ventricular premature complexes in 2/15 and mild hypotension due to blood loss in 1/15. Post‐procedural adverse events included neutropenia (4/15, two grade I, one grade II, one grade III) diarrhea (3/15, two grade I, one grade II), lethargy (3/15, grade I), vomiting (2/15 grade II) and mild hindlimb lameness (2/15).

Clinical response was evaluated in ten dogs. An improvement was reported in 9/10 dogs and stable clinical signs in 1 dog.

Restaging was available for eight dogs. Time of restaging varied from 14 to 165 days. 4/8 had PR, 2/8 SD and 2/8 PD.

Despite the small sample size and retrospective nature of this study the result suggest that IA vinblastine is well tolerated in dogs. In many cases it was associated with a rapid clinical improvement.

**Disclosures**

**Source of Funding**

**ECVIM CSF Funding**

No.

**ESVONC – European Society of Veterinary Oncology**

## ESVONC‐O‐6

## Bilateral anal sacculectomy in the management of apocrine gland anal sac adenocarcinoma: A retrospective assessment

### A. Lane^1^, N. Milevoj^1^, P. Cazzini^2^, A. Malbon^3^, K. Pratschke^1^, L. Blackwood^1^

#### ^1^Hospital for Small Animals, University of Edinburgh, Edinburgh, United Kingdom; ^2^Easter Bush Pathology, University of Edinburgh Easter Bush Pathology, Edinburgh, United Kingdom; ^3^Easter Bush Pathology, University of Edinburgh, Edinburgh, United Kingdom

Apocrine gland anal sac adenocarcinoma (AGASACA) is a common malignant perianal neoplasm in the dog. Typically, it is unilateral, however bilateral AGASACA, where both anal sacs contain separate neoplasms, have been described in 4 to 14% of AGASACA cases in multiple studies. The treatment of choice for localised AGASACA is closed anal sacculectomy to remove the primary, with excision of metastatic lymph nodes as appropriate. Unilateral sacculectomy is still commonly performed, with intraoperative and postoperative complication rates of 7% and 17% (respectively) reported in a recent publication.

The aim of this study was to describe the incidence of undetected contralateral AGASACA, in a population of dogs undergoing routine bilateral anal sacculectomy for management of AGASACA. A secondary aim was to describe the surgical complication rate.

This was a single‐centre, retrospective study, assessing dogs who had bilateral anal sacculectomy performed as the standard of care surgical approach for the management of AGASACA of any stage, between June 1996 and September 2022. Dogs undergoing lymphadenectomy were not excluded. AGASACA was histologically confirmed.

Clinical data retrieved from the clinical records included signalment, tumour stage, intraoperative and postoperative complications, and additional treatments. Clavien–Dindo classification was used to grade post‐operative complications. Referring veterinarians were contacted for follow up information.

Sixty‐four dogs met the inclusion criteria. Of these, 15 dogs (23.4%) had bilateral AGASACA. In eight dogs (12.5%), the contralateral tumour was not detected on pre‐operative examination or imaging. Two dogs (3.1%) had dysplastic changes identified in the contralateral anal sac.

Seven dogs (10.9%) had intraoperative complications. All seven had mild to moderate haemorrhage not requiring transfusion of blood products for management, and in four cases this was associated with lymphadenectomy. Twelve dogs (18.8%) experienced a total of 14 post‐operative complications. Of these, 8 were grade I, 4 were grade II, and 2 were grade III. No grade IV or V complications were observed. These results are comparable to reported complication rates with unilateral surgery.

A significant minority of dogs with AGASACA have a contralateral neoplasm, undetectable with pre‐operative imaging or examination. Performing bilateral anal sacculectomy should be considered for all AGASACA patients, even those cases presenting with apparent unilateral disease. This study also suggests that bilateral anal sacculectomy is not associated with a higher frequency or severity of post‐operative complications than unilateral sacculectomy.

**Disclosures**

**Source of Funding**

No funding.

**ECVIM CSF Funding**

No.

**ESVONC**—**European Society of Veterinary Oncology**

## ESVONC‐O‐7

## Presentation and treatment of feline HSA in the UK 2009‐2024: A retrospective study of 74 cases

### G. Coppola^1^, B. Rulf^2^, O.D. Davies^3^, I. Amores‐Fuster ^4^, I. Gramer^5^, M. Macfarlane^6^, K. Purzycka^7^, J. Morris^8^, S. Mason^1^

#### ^1^Southfields Veterinary Specialists, Basildon, United Kingdom; ^2^Bristol Vet Specialists, Bristol, United Kingdom; ^3^Bristol Vet Specialists, Bristol, United Kingdom; ^4^Univeristy of Liverpool, Liverpool, United Kingdom; ^5^Royal Veterinary College, RVC, Hawkshead, United Kingdom; ^6^North Downs Specialist Referrals, Bletchingley, United Kingdom; ^7^Anderson Moores Veterinary Specialists, Winchester, United Kingdom; ^8^University of Glasgow, Glasgow, United Kingdom

Feline Haemangiosarcoma (fHSA) is considered to be a rare highly malignant tumour. Elder cats are the most affected without any sex or breed predisposition. Primary sites are spleen and skin/subcutaneous tissues with liver and lungs as high metastatic sites. Cats with cutaneous and subcutaneous (sc‐)fHSA typically exhibit firm or soft swelling. Visceral tumours may cause different signs; mostly related to tumour rupture. Surgery is the ideal first treatment. Chemotherapy is often recommended following surgery and is usually the first primary therapy when surgery is not a feasible option. Prognosis of visceral HSA is poor: median survival time (MST) is generally short (2.5 months). Cutaneous HSAs treated with aggressive surgery have long reported MSTs (9 months to 4 years).

This retrospective multicentre case‐series study aims to describe clinical presentation, response to treatment and metastatic patterns of fHSA in UK cats.

Clinical records from seven UK‐based referral hospitals were searched. Cats were included if they had a histological diagnosis of fHSA. The following information was reviewed from medical records: signalment, clinical signs, organs involved, laboratory results, diagnostic imaging, treatments and date of treatment/death.

Seventy‐four cats were included: forty‐three with sc‐HSA and thirty‐one with visceral HSA. Abdominal distention and weakness due to intraperitoneal effusion were the most common presenting clinical signs for visceral HSA, as was a visible mass for the sc‐fHSA. Anaemia (53% of fHSA with 85% visceral fHAS and 35% sc‐fHSA) and azotaemia (44% of fHSA with 38% visceral fHAS and 48% sc‐fHSA) were the most common abnormalities on laboratory testing. Spleen (90%) and liver (19%) were the most involved abdominal organs. Patients required stabilisation and blood transfusion in most cases of visceral‐HSA. 24% of cats presented metastasis with lungs and regional lymph nodes the most detected metastatic sites (both 33%). Only abdominal ultrasound was the most commonly performed test (51%), followed by abdominal ultrasound and thoracic radiographs (29%) and thoracic and abdominal computer tomography (26%). Fifty‐six patients had surgery with complete excision. Eighteen were followed with systemic chemotherapy. Doxorubicin‐based protocol was the most used. Electrochemotherapy and radiotherapy were less‐described treatments. MST was around 98 days: MST for visceral fHSA was shorter (49 days) than for sc‐fHSA (193 days).

This study confirms that fHSA is associated with a poor long‐term prognosis and relapse of the initial clinical signs should be expected.

**Disclosures**

**Source of Funding**

**ECVIM CSF Funding**

No.

**ESVONC**—**European Society of Veterinary Oncology**

## ESVONC‐O‐8

## New hope in the treatment of canine hemangiosarcoma – Potentiation of doxorubicin with Poly (ADP‐ribose) polymerase inhibitors and repurposed propranolol

### K. Kapturska^1^, A. Pawlak^2^

#### ^1^University of Environmental and Life Sciences Department of the Pharmacology and Toxicology, Wroclaw, Poland; ^2^University of Environmental and Life Sciences Department of the Pharmacology and Toxicology UPWr, Wroclaw, Poland

Canine hemangiosarcoma (HSA) is a devastating, highly aggressive malignant tumor with a poor prognosis, mainly managed with surgery and adjuvant chemotherapy. Besides many studies effective treatment is still needed, recently focusing on small molecular compounds. Thus, doxorubicin (DOX) covers the majority of chemotherapy protocols used in HSA therapy, we attempt to potentiate its effectiveness with the use of Poly (ADP‐ribose) polymerase (PARP) inhibitors (olaparib, niraparib) together with non‐selective β‐blocker, propranolol (PRO). Using culture of two of the commercially purchased HSA cell lines, DD‐1 and DHSA‐1426 we conduct cell proliferation and clonogenic assay together with subsequent western blot analysis of changes in the protein expression involved in DNA damage response, cellular stress induction and apoptosis, as well as detection of the pathways involved in mechanisms underlying antiproliferative activity of tested compounds that we have observed. After incubation for 72h with increasing concentrations of DOX, niraparib (NIR), olaparib (OLA) and PRO as well as their combinations (constant rate), antiproliferative activity has been obtained on both cell lines. All of the tested compounds separately were able to inhibit proliferation DHSA‐1426 cell line was significantly more sensitive than DD‐1 for DOX and OLA, but NIR and PRO show similar activity in both canine HSA cell lines. The combination treatment significantly decreased cell viability compared to a single agent in both cell lines. Calculation of the combination index (CI) showed synergy (CI <1.0) for most of the tested pairs of drugs, as well as for triple therapy, with the lowest values for DOX+OLA (ratio 1:50) and DOX+NIR (1:12.5) in DD‐1 cell line and DOX+OLA+PRO (1:500:1000) in the DHSA‐1426 cell line, in which it has been reported as strong synergy (CI between 0.1 and 0.3). Dose Reduction Index (DRI) estimation shows preferable dose reduction, especially for the highest concentrations of the DOX and OLA‐2.0 and 100 μM with DRI >30 and >11 for both drugs, respectively. Moreover, OLA‐potentiated DOX has been effective in weakening the ability to form colonies by the DHSA‐1426 cell line. A significant decrease in survival fraction (SF)% has been noted (48%, 58% vs. 28%). Based on these results, designed and broad clinical studies should be conducted to estimate the clinical relevance of using PARPi and PRO in chemotherapeutic protocol in canine HSA, both of which are easily achievable on the market nowadays.

**Disclosures**

**Source of Funding**

The author of this publication receives research funding from the Implementation Project for Doctoral Studies, which, following the arrangements of the tripartite agreement numbered WOI.NI.4211.IN.6/WOI/2020, dated 2/11/2020, takes care of product development and search for solutions for the introduction of an innovative business solution based on the results described in this publication. In addition, the first author and her company are a partner of Wroclaw University of Environmental and Life Sciences and are cooperating in the development of the implementation. The terms of this agreement have been reviewed and approved by the Ministry of Education and Science as part of its policy to support scientific research among businesses.

**ECVIM CSF Funding**

No.

**ESVONC**—**European Society of Veterinary Oncology**

## ESVONC‐O‐9

## Histopathological growth pattern may predict survival in dogs with splenic haemangiosarcoma post surgery; A case‐control pilot study

### B. Rolf^1^, D. Wasques^2^, I. Schofield^3^, O. Davies^3^

#### ^1^Bristol Vet Specialists Oncology, Bristol, United Kingdom; ^2^Finn Pathologists, Harleston, United Kingdom; ^3^Bristol Vet Specialists, Bristol, United Kingdom

Canine splenic haemangiosarcoma (CSH) is associated with median survival time (MST) of 19–86 days with splenectomy alone, yet a minority of dogs experience prolonged survival (PS). The aim of this study was to identify histopathological features which may predict PS.

Records of a UK commercial histopathological laboratory were retrospectively searched for CSH cases treated by their first opinion practices; follow‐up was obtained from searching clinical records. Signalment, presentation, diagnostic tests, treatment and outcome were recorded. Cases were included if they underwent splenectomy, had a clear CSH histopathological diagnosis and complete follow‐up until death or the date of data collection. Cases were excluded if the diagnosis was questionable, medical anticancer treatment was given (excluding non‐steroidal anti‐inflammatory drugs), or were lost to follow‐up. After a group of 10 dogs with PS was formed (>6 month survival after surgery), a group of 20 control dogs (survival between 2 weeks and 6 months) was assembled, matched for age, breed and date of diagnosis.

PS group had a MST of 384 days (195–898), mean bodyweight 27.2kg and mean age 110.3 months. Control group had a MST of 60.5 days (16–136), mean bodyweight 28.7kg and mean age 124.8 months. Acute haemoabdomen was the most common presentation (90% of PS cases, 85% of control cases). Stage 3 was suspected in 3 control cases; all other cases were stage 2. Stage was not statistically different between groups (*P*=0.53).

Histopathology of all cases were reviewed by one pathologist who was blinded to which group cases belonged. Growth patterns (GP) were defined as: (I) vascular; (II) papillary with dense collagenous stroma; (III) papillary with scant stroma and plump neoplastic cells; (IV) solid with spindloid appearance; (V) solid with epithelioid/plump appearance and (VI) red pulp‐like pattern. The proportion of each GP within each case was scored semi‐quantitatively: (0) absent; (1) <25%; (2) 25%–50% and (3) >50% of tumour area. Mitotic count (per 2.37 mm^2^) was assessed in areas of highest mitotic activity.

PS and control groups were compared using Fisher's exact and Wilcoxon Rank Sum tests. GP classification VI was significantly more prevalent in dogs with PS (median score 1) when compared to controls (median score 0; *P* = 0.01). Mitotic count, haemoabdodomen and anaemia at diagnosis were not significantly different between groups.

GP classification VI (red pulp‐like pattern) might have prognostic significance in CSH, and further survival analysis in a larger dataset is ongoing.

**Disclosures**

**Source of Funding**

CVS resident fund.

**ECVIM CSF Funding**

No.

**ESVE**—**European Society of Veterinary Endocrinology**

## ESVE‐O‐1

## Experiences with flash glucose monitoring system in dogs and cats: A global survey of veterinary practitioners

### M. Didier^1^, M. Re^1^, F. Fracassi^1^, A.M. Tardo^1^, C. Scudder^2^, N. Stijn^2^, C. Gilor^3^, L. Fleeman^4^, O.L. Rodolfo^5^, A. Carolina^6^, F. Zeugswetter^7^, F. Del Baldo^1^

#### ^1^University of Bologna Department of Veterinary Medical Science, Ozzano dell'Emilia, Bologna, Italy; ^2^Royal Veterinary College Department of Clinical Science and Sevices, London, United Kingdom; ^3^University of Florida Department of Small Animal Clinical Sciences, Gainesville, United States; ^4^Animal Diabetes Australia, Victoria, Australia; ^5^University of Lisbon Centre for Interdisciplinary Research in Animal Health, Lisbon, Portugal; ^6^AniCura Hospital Veterinario Valencia Sur, Valencia, Spain; ^7^University of Veterinary Medicine Department of Small Animal and Horses Division of Small Animal Internal Medicine, Vienna, Austria

The flash glucose monitoring system (FGMS) has revolutionized the management of diabetic cats and dogs. The study aim was to investigate the use of FGMS by veterinarians and its perceived effectiveness in monitoring and managing dogs and cats, and compare its use with other monitoring methods. An online English survey translated into four different languages (Portuguese, Spanish, Italian and German) was developed. Respondents were recruited through the social network of veterinary groups, mailing lists and presentations. Overall, 363 responses from 24 countries were included. The median (range) number of cats and dogs monitored per year was 5.5 (1–10); 56.5% of respondents used the FGMS more than five times. Seventy‐six percent of respondents requested a set fee for sensor application and glucose data interpretation, while 24% did not. The cloud‐based Libreview diabetes data‐sharing system was used by 52.7% of veterinarians, while 44.3% assessed glucose trends through screenshots of graphs sent by owners. FGMS was employed in various clinical settings, including poor diabetic control (24.7%), newly diagnosed patients (23.2%), suspected hypoglycemic events (17.7%), diabetic ketoacidosis (DKA) (15.7%), routine diabetic monitoring (14.8%), and insulinoma (3.9%). The application of the sensor, its removal, or both procedures were considered challenging by 61.5%, 28.9%, and 9.6% of respondents in dogs, and by 65.2%, 24.6%, and 10% of respondents in cats, respectively. Compared to blood glucose curves, FGMS was considered to allow easier therapeutic decisions (73.5%), result in better glycemic control (58%), and less stressful for the patient (92.5%). Sensor malfunction (53.9%), difficulty in managing owners’ anxiety (43.7%), and premature sensor detachment (41.7%) were identified as main drawbacks. When considering other monitoring methods (clinical signs, urine dipstick and glycated proteins), FGMS advantages included easier therapeutic decisions (73.3%), reduced patient stress (67%), and better glycemic control (66.5%). According to 50.2% of respondents, FGMS was considered more expensive than other monitoring methods. Since using FGMS, 51.2% and 36.7% of respondents feel a lower incidence of hypoglycemic and DKA events, respectively. Additionally, 84.4% of respondents noted an improvement in successfully managing diabetic dogs and cats, while 79% reported increased confidence in making therapeutic decisions. The use of FGMS generated a sense of confidence in 72.3% of respondents. In conclusion, the use of FGMS positively influenced the management of dogs and cats by veterinarians. However, sensor malfunction, its costs and the necessity to address owner anxiety were identified as the primary drawbacks.

**Disclosures**

Francesca Del Baldo Speaking & consultancies: MYLAV Veterinary Laboratory, Boehringer Ingelheim, Dechra, Royal Canin. Federico Fracassi Financial support: Dechra, MSD, Monge. Speaking & consultancies: Boehringer Ingelheim, Dechra, MSD, Royal Canin, Hill's, Nestlé Purina, MYLAV Veterinary Laboratory. Antonio Maria Tardo Financial support: Monge. Speaking & consultancies: Dechra, MYLAV Veterinary Laboratory. Mariachiara Re Speaking & consultancies: Dechra Linda Fleeman: Speaking & consultancies: MSD Animal Health, Zoetis, Royal Canin, Nestle Purina, Dechra, Boehringer‐Ingelheim Carolina Arenas: Speaking & consultancies: Dechra, Boehringer. Rodolfo Oliveira Leal: Speaking & consultancies: Dechra, Boehringer Ingelheim, VIN Stijn Niessen Consultant / funding from Boehringer Ingelheim, Dechra, MSD, Purina, Hill's, Zoetis

**Source of Funding**

**ECVIM CSF Funding**

No.

**ESVE**—**European Society of Veterinary Endocrinology**

## ESVE‐O‐2

## Assessment of serum fructosamine and glycated hemoglobin as indicators of glycemic control in diabetic dogs: A comparative study using FreeStyle Libre‐derived metrics

### F. Del Baldo^1^, M. Re^1^, A. Rossi^1^, A. Corsini^2^, A.M. Tardo^3^, F. Fracassi^3^

#### ^1^University of Bologna Department of veterinary medical sciences, Ozzano dell'Emilia, Italy; ^2^University of Parma Department of Veterinary Science, Parma, Italy; ^3^University of Bologna Department of veterinary medical sciences, Ozzano dell'Emilia, Italy

Serum fructosamine (SF) and glycated hemoglobin (HbA1c) are commonly used to monitor diabetic dogs (DD) although their use was recently questioned because of the poor performances in reflecting the glycemic control (GC). However, previous studies assessed the correlation between SF and HbA1c concentrations and clinical signs, often in conjunction with blood glucose curves, which may not be optimal parameters for comparison. The objective of this study was to investigate the accuracy of SF and HbA1c to categorize glycaemic control in comparison to FreeStyle Libre‐derived metrics (FSLDM) in DD. Nineteen DD on insulin treatment were prospectively enrolled in the study. SF and HbA1c concentrations were measured and FreeStyle Libre was applied at the time of inclusion (T0) and thereafter every two weeks (T2, T4, T6, and T8). At each time point, the ALIVE Diabetic Clinical Score was recorded and the following FSLDM were extrapolated: percentage of time‐in‐range (TIR%, 70–250 mg/dL), time above range (TAR%, >250mg/dL), time below range (TBR%, <70 mg/dL), mean glucose (MG), glucose management indicator (GMI) and percent coefficient of variation (CV). Correlations between SF and the FSLDM at T2, T4, T6 and T8 were evaluated, while HbA1c correlations were evaluated only with the FSLD at T8. Mann–Whitney test was used to compare SF in dogs with good GC (defined as >75% TIR) and dogs with poor GC (defined as <75% TIR). The ROC‐curve analysis was performed to determine the best cut‐off of SF to classify GC. These last analyses were not performed for HbA1c due to the small sample size. SF were significantly correlated with TIR% (rs=−0.51, *P* < 0.0001), TAR% (rs = 0.25, *P* = 0.027), MG (rs=0.50, *P* < 0.0001) and GMI (rs = 0.58, *P*<0.0001). HbA1c was significantly correlated with TAR% (*r* = 0.49, *P*=0.04). The correlation between HbA1c and TIR%, MG and GMI was not significant. Additionally, the correlation between SF and HbA1c with CV%, ALIVE score and TBR% was not significant. Median SF was 412 μmol/L and 494 μmol/L in dogs with good GC and poor GC, respectively (*P* = 0.001). ROC‐curves analysis of SF to distinguish poor GC from good GC dogs showed an AUC of 0.78. SF>460 μmol/L had Sp=85%, Se=70% in discriminating dogs with poor GC from dogs with good GC. In conclusion, HbA1c showed no correlation with the majority of the FSLDM over a 2‐month period. SF had moderate accuracy in classifying the GC. The glycated proteins should not be used as the sole indicator of GC, and can only complement clinical signs and FreeStyle Libre glucose data.

**Disclosures**

Francesca Del Baldo Speaking & consultancies: MYLAV Veterinary Laboratory, Boehringer Ingelheim, Dechra, Royal Canin. Federico Fracassi Financial support: Dechra, MSD, Monge. Speaking & consultancies: Boehringer Ingelheim, Dechra, MSD, Royal Canin, Hill's, Nestlé Purina, MYLAV Veterinary Laboratory. Antonio Maria Tardo Financial support: Monge. Speaking & consultancies: Dechra, MYLAV Veterinary Laboratory. Andrea Corsini Speaking & consultancies: MYLAV Veterinary Laboratory, Boehringer Ingelheim, Dechra, Royal Canin. Mariachiara Re Speaking & consultancies: Dechra.

**Source of Funding**

**ECVIM CSF Funding**

No.

**ESVE**—**European Society of Veterinary Endocrinology**

## ESVE‐O‐3

## Cabergoline for the treatment of feline hypersomatotropism without concurrent diabetes mellitus

### D. Miceli^1^, J. Rey Amunategui^2^, G. Pompili^2^, I. Espiñeira^3^, O. Pignataro^4^

#### ^1^IBYME CONICET, Buenos Aires, Argentina; ^2^MAIMONIDES UNIVERSITY, Buenos Aires, Argentina; ^3^Private practice Private practice, Buenos Aires, Argentina; ^4^IBYME CONICET Ibyme, Buenos Aires, Argentina

Feline hypersomatotropism (HST) is typically associated with diabetes mellitus (DM), and only a few case reports have described HST without concurrent DM. Studies on the treatment of this subpopulation of cats with HST alone are lacking. Cabergoline is a long‐acting dopamine agonist with high affinity for dopamine receptor D2. In approximately one third of diabetic cats with HST, cabergoline effectively reduced insulin‐like growth factor 1 (IGF‐1) concentrations and achieved DM remission. The aim of this study was to evaluate the safety and efficacy of cabergoline monotherapy for the treatment of HST in cats without concurrent DM. This prospective study included nine (*n* = 9) non‐diabetic cats with HST. The diagnosis of HST was based on (1) clinical signs compatible with HST/acromegaly (acromegalic features: enlargement of the head, abdomen and paws; prognathia inferior; respiratory stridor; polyphagia), (2) markedly increased serum IGF‐1 concentration (>1000 ng/ml) and (3) presence of pituitary enlargemen> (>4 mm dorsoventral height on computed tomography [CT]). Cats received an oral dose of cabergoline (10 μg/kg every 48 hours) for six months. Serum IGF‐1 and fructosamine concentrations, complete blood count, serum biochemistry, and urinalysis were assessed at the time of diagnosis of HST (t0), after 3 (t1) and 6 months (t2) of cabergoline treatment. Six cats (*n* = 6) completed the study. The median dorsoventral height of the pituitary gland on CT at enrolment was 4.2 mm (range 4–8.1). The median serum IGF‐1 concentration was 1236 ng/ml (range 1009‐2501 ng/ml) at t0, 1100 ng/ml (range 251–2455 ng/ml) at t1, and 864 ng/ml (range 369–1275 ng/ml) at t2. Serum IGF‐1 concentrations showed significant differences when comparing t0 and t1 (*P*=0.02), with normalization observed in 4/9 of the cases. No significant differences were observed between IGF‐1 concentrations at t1 and t2 (*P*=0.06), and between t0 and t2 (*P*=0.06), although a normalization was observed at t2 in 3/6 of the cases. No significant differences in serum fructosamine concentration were observed and none of the cats showed hyperglycemia/glycosuria or developed DM during the study. One cat experienced vomiting after cabergoline administration. No other side effects were observed. This study suggests that cabergoline monotherapy, though it does not revert IGF‐1 increase at 6 months, substantially reduces IGF‐1 concentration in 44% and 33% of non‐diabetic cats with HST at 3 and 6 months, respectively. Therefore, cabergoline could be considered as a possible pharmacological treatment option for non‐diabetic cats with HST.

**Disclosures**

**Source of Funding**

**ECVIM CSF Funding**

No.

**ESVE**—**European Society of Veterinary Endocrinology**

## ESVE‐O‐4

## Does hypophysectomy cause oxytocin deficiency in cats?

### G. Kaemper^1^, J. Fenn^1^, R. Fowkes^2^, D. Church^1^, C. Scudder^1^

#### ^1^Royal Veterinary College Department of Clinical Science and Services, Hatfield, United Kingdom; ^2^College of Veterinary Medicine, Michigan State University Small Animal Clinical Sciences, Michigan, United States

Roughly two thirds of cats experience persistent diabetes insipidus following hypophysectomy but the effects of hypophysectomy on serum oxytocin concentrations have not been described. Oxytocin deficiency has been associated with hyperphagia, and oxytocin supplementation reduces appetite and increases lean body tissue in rodents. A median weight gain of 1.2 kg by 12 months post‐hypophysectomy has been reported in cats. The aim of this study was to evaluate the effect of hypophysectomy on serum oxytocin concentration in cats treated for hypersomatotropism (HST) and diabetes mellitus (DM).

A database of paired serum samples stored at −80^o^C from cats with HST and DM pre‐ and post‐hypophysectomy was searched. A commercial ELISA validated for the measurement of feline oxytocin was used and serum samples were measured following solid‐phase extraction. Pre‐ and post‐hypophysectomy serum samples were each measured in duplicate. Patient descriptive and outcome data were recorded, including serum IGF1 concentration and resolution of DM. Statistical analysis comparing serum oxytocin concentration pre‐ and post‐hypophysectomy using Wilcoxon matched pairs signed‐rank test was performed. The relationships between oxytocin and DDAVP requirement, and oxytocin and percent bodyweight change were statistically analysed using Mann–Whitney U test and Spearman's correlation respectively.

Twelve paired samples were included with 11/12 cats achieving normalisation of serum IGF1 concentration and resolution of DM following hypophysectomy, and 1 cat had persistently elevated IGF1 and insulin‐resistant DM. There was no difference between pre‐ and post‐hypophysectomy oxytocin concentrations (pre‐hypophysectomy median 16 pg/mL, range 0–61 and post‐hypophysectomy median 22 pg/mL, range 0–42, *P*=.65). There was no relationship between post‐hypophysectomy oxytocin concentration and requirement for long‐term DDAVP supplementation (P=0.81). There was no correlation between post‐hypophysectomy oxytocin concentration and the percentage change in bodyweight at 3 to 6 months (P=0.70).

This study suggests that cats continue to produce and secrete oxytocin following hypophysectomy, and oxytocin deficiency is not a contributor to post‐operative weight gain. Further study with a larger cohort of cats would be indicated, as serum oxytocin concentrations were at the lower limit of the ELISA detection range.

**Disclosures**

**Source of Funding**

**ECVIM CSF Funding**

No.

**ESVE**—**European Society of Veterinary Endocrinology**

## ESVE‐O‐5

## Is follow‐up thyroid scintigraphy useful in hyperthyroid cats treated with radioiodine?

### L. Stammeleer^1^, P. Xifra^2^, S.I. Serrano^2^, E. Vandermeulen^3^, S. Daminet^1^, M. Peterson^4,5^

#### ^1^Ghent University, Merelbeke, Belgium; ^2^Iodocat, Madrid, Spain; ^3^DAC Hond & Kat, Deinze, Belgium; ^4^Animal Endocrine Clinic, New York, United States; ^5^Cornell University, Ithaca, United States

Qualitative/quantitative thyroid scintigraphy is commonly used for diagnosis and staging of hyperthyroid cats. Little information exists about scintigraphic changes after 131I treatment. The objective of this study was to describe follow‐up scintigraphy and to compare these results with associated serum thyroid values for determination of thyroid status post radioiodine therapy.

Two‐hundred‐and‐three client‐owned hyperthyroid cats were included in this study. Technetium‐pertechnetate thyroid scintigraphy (qualitative and quantitative) was performed at time of 131I treatment (T0) and six months thereafter (T6). Serum total thyroxine (TT4) and thyroid‐stimulating hormone (TSH) were assessed at both timepoints. Background‐corrected thyroid/salivary ratio was calculated as followed: (thyroid counts‐tracheal background counts) / (salivary counts‐tracheal background counts).

After radioiodine treatment, all 203 cats showed a reduction in size and brightness of their “hot” tumor nodule(s). Fifty‐four of 86 (63%) cats with unilateral disease had recovery of the suppressed thyroid lobe, whereas it remained undetectable in 32 (37%) cats. Of 117 cats with bilateral disease, follow‐up scintigraphy revealed two lobes in 97/117 (83%), one lobe in 11/117 (9%), and none in 9/117 (8%). After 131I treatment, cats were classified for their thyroid status based on serum TT4 and TSH levels: 141/203 (69.4%) cats were euthyroid (normal TT4 and TSH), 55/203 (27.1%) cats developed subclinical iatrogenic hypothyroidism (normal TT4 and high TSH), 5/203 (2.5%) cats developed overt iatrogenic hypothyroidism (low TT4 and high TSH) and 2/203 (1%) cats remained hyperthyroid (high TT4 and low TSH). In 32/141 (23%) of these euthyroid cats a persistent “hot” nodule was noted, with increased values for percentage thyroidal uptake of pertechnetate (%TcTU) or thyroid/salivary ratio (T/S). For the 60 cats with either subclinical or overt iatrogenic hypothyroidism, only 18/60 (30%) had low %TcTU, 15/60 (25%) had low background‐corrected T/S, and none had low standard T/S.

After 131I treatment, cats show a reduction in size and brightness of their thyroid tumor nodule(s). Some retain a small “hot” nodule, which only rarely indicates persistent hyperthyroidism and thus need for retreatment. Suspected hypothyroid cats show poor agreement between serum thyroid values and scintigraphic variables. Thyroid scintigraphy is a very useful diagnostic test for hyperthyroid cats but is of less value for diagnosing iatrogenic hypothyroidism or persistent hyperthyroidism after 131I treatment.

**Disclosures**

Two of the authors received funding for another project by the EveryCat Health Foundation. Two of the authors are associated with Boehringer Ingelheim for scientific work (lectures, product development), not for this project.

**Source of Funding**

This study was supported by AML‐Medvet Antwerp and Idexx USA/Europe.

**ECVIM CSF Funding**

No.

**ESVE**—**European Society of Veterinary Endocrinology**

## ESVE‐O‐6

## Large‐scale screening for cases of suspected spontaneous hypothyroidism in adult cats and description of clinical, laboratory, scintigraphic features and treatment in four affected cats

### J. Lin^1^, C. Schwens^2^, S. Schaub^3^, N. Bauer^4^, K. Hazuchova^5^

#### ^1^Clinic for Small Animals, Internal Medicine, Justus‐Liebig‐University Clinic for Small Animals, Internal Medicine, Giessen, Germany; ^2^Antech Lab Germany GmbH Companion animals, Augsburg, Germany; ^3^Clinic for Small Animals, Diagnostic Imaging, Justus‐Liebig‐University Diagnostic Imaging, Giessen, Germany; ^4^Central Laboratory, Internal Medicine, Justus‐Liebig‐University Veterinary Clinical Pathology and Pathophysiology, Giessen, Germany; ^5^Clinic for Small Animals, Internal Medicine, Justus‐Liebig‐University Internal Medicine, Giessen, Germany

Because clinical and clinico‐pathological variables have only been described in a few adult cats with spontaneous hypothyroidism, it is currently unknown what findings should raise suspicion and prompt screening.

The aim of this study was to screen laboratory submissions for cats with suspected spontaneous hypothyroidism (SSH), perform confirmatory tests and describe treatment response.

Laboratory submissions (geriatric profile, total thyroxine [TT4]) were prospectively screened for cats with low TT4 aged 3–12 years (January 2022–April 2023). TSH was measured; iatrogenic hypothyroidism was excluded by contacting submitting veterinarians. Creatinine, cholesterol, red blood cell count (RBC) of cats with SSH (low TT4, TSH>0.53 ng/mL [total allowable error of the upper reference range (RR)]) was compared to cats with TT4 within RR and cats with low TT4 but normal TSH (<0.3 ng/mL) by non‐parametric tests. Significant was *P* < 0.05. Hypothyroidism was confirmed based on technetium‐99m thyroid scintigraphy. History, physical examination, haematology, biochemistry, urinalysis, TT4, free thyroxine (fT4), TSH, thyroid ultrasound, TSH‐stimulation test, thyroid scintigraphy, and response to L‐thyroxine therapy over 6‐months follow‐up are described.

31572 submissions were included. Cats with SSH (*n* = 53/31572; 0.17%) had higher creatinine (median 149 vs. 129 μmol/L, P=0.002) and lower RBC (7.9 vs. 9 ×10^12^/L, P=0.0001) than age‐matched cats with TT4 within RR (*n* = 25274) as well as higher creatinine (149 vs. 122 μmol/L, *P*=0.004) than cats with low TT4 but normal TSH (*n* = 3290). 23/41 (56%) cats with SSH with known creatinine were azotaemic (creat>>ni>e >140 μmol/L).

4/53 cats with SSH presented for further work‐up. All had body condition score (BCS) 6‐7/9, and scrappy, thin hair coat; 3/4 were lethargic and 2/4 were diabetic. All ha> l>w TT4> TSH > RR, 3/4 had low fT4. 3/4 cats were azotaemic, 1/4 had urine specific gravity (USG) <1035. TSH‐stimulation test indicated hypothyroidism in 4/4; ultrasound revealed small thyroid glands in 2/4 and bilaterally enlarged thyroid glands in 2/4 cats; 2/4 had no functional thyroid tissue and 2/4 had severely enlarged thyroidea on scintigraphy. TT4 normalised 4 weeks and TSH 12‐20 weeks after starting L‐thyroxine (final dose 200 μg/kg/day). Hair coat and BCS improved in 4/4; creatinine normalised after 4–24 weeks in 3/3 and USG in 1/1 cats with initial abnormalities. 1/2 diabetic cats went into remission.

SSH was suspected in 0.17% cats. Increased creatinine might indicate the need for TSH testing in cats with low TT4. Treatment leads to improvement of BCS and azotaemia. Normalisation of TSH might take several months.

**Disclosures**

Katarina Hazuchova is a consultant for Boehringer Ingelheim. Christina Schwens is an employee of Antech Lab Germany GmbH. Antech Lab Germany GmbH performed TSH measurements for a reduced price.

**Source of Funding**

This study received a grant from ECVIM‐CA Clinical Studies Fund (CSF) (ESVE Pilot Research Award), which was used to cover the costs of TSH measurements.

**ECVIM CSF Funding**

Yes.

**ESVE**—**European Society of Veterinary Endocrinology**

## ESVE‐O‐7

## Clinical and laboratory characterization of congenital hypothyroidism in 23 kittens

### S. Hulsebosch^1^, W. Vernau^2^, S. Marks^2^, K. Vernau^2^

#### ^1^UC Davis UC Davis, Davis, United States; ^2^UC Davis, Davis, United States

Naturally‐occurring hypothyroidism is uncommon in cats; however, it is more commonly reported in kittens than adult cats. Congenital hypothyroidism may be an underrecognized cause of “failure‐to‐thrive” in kittens. We aimed to prospectively assess “failure‐to‐thrive” kittens for congenital hypothyroidism and describe patient characteristics and outcome.

Unwell kittens with small stature were prospectively recruited from 2019 to 2023 and, if history and physical examination were consistent with congenital hypothyroidism, they were screened for enrollment via CBC, serum chemistry panel, total thyroxine (T4), free thyroxine (fT4), and canine thyroid‐stimulating hormone (cTSH) measurement.

Forty‐eight unwell kittens were screened: 25 were excluded due to alternate diagnoses; 23 were diagnosed with congenital hypothyroidism. Median age at diagnosis was 60.5 days (range 21–150), median weight was 474 g (range 155–1000), 12 were intact females, and 11 were intact males. Most common clinical signs included poor growth, short and disproportionate stature, dull mentation, and constipation. At diagnosis, median T4 was 0.5 μg/dL (range 0–0.93; RI 0.69–3.50), median fT4 was 0.3 pmol/L (range 0.3–15; RI 10–53), median cTSH was 8.38 ng/mL (range 0.13–12; RI 0–0.38). Primary hypothyroidism was diagnosed in 21 kittens, while secondary hypothyroidism was suspected in 2 kittens.

Median dose of levo‐thyroxine to first achieve clinical and biochemical control was 35.3 mcg/kg PO q12h (range 20.8–48.2), which is higher than typically used in adult dogs/cats. Eighteen of 23 kittens are still alive. Congenital hypothyroidism appears to be more common than previously reported and is a treatable cause of poor growth in kittens.

**Disclosures**

**Source of Funding**

EveryCat Foundation.

**ECVIM CSF Funding**

No.

**ESVE**—**European Society of Veterinary Endocrinology**

## ESVE‐O‐8

## Measurement of reverse triiodothyronine concentrations in healthy dogs, dogs with primary hypothyroidism and nonthyroidal illness syndrome

### J. Russ^4^, C. Schwens^1^, N. Glos^2^, S. Ritz^3^, C. Meindl^3^, R. Müller^5^

#### ^1^Antech Lab Germany Antech Lab Germany, Augsburg, Germany, Augsburg, Germany; ^2^Dermatologie Kleintierzentrum Germering, Germering, Germany; ^3^Anicura Tierklinik Haar, Haar, Germany; ^4^Mars GmbH, Verden (Aller), Germany; ^5^Kleintierklinik LMU München, München, Germany

In clinical practice, dogs with primary hypothyroidism are typically diagnosed by thyroxine (TT4) measurement. Unfortunately, TT4 is also low in 31%–40% of dogs with nonthyroidal illness syndrome (NTIS). Additional analysis of TSH increases diagnostic specificity, but not sensitivity. In human medicine, reverse triiodothyronine (rT3) concentration can differentiate NTIS from hypothyroidism.

The aim of this prospective study was to establish a reference range for rT3 using HPLC‐MS/MS in healthy dogs (HD) and to evaluate rT3 in dogs with hypothyroidism and NTIS (group 1 + 2).

All dogs included in the study were adult and received a complete blood testing and clinical examination. Furthermore, a premium thyroid profile (TT4, fT4 (ED), TT3, TSH, thyroglobuline autoantibodies, and T3/T4‐ autoantibodies) was initiated in groups 1 and 2. Hypothyroidism was diagnosed based on typical clinical signs with compatible laboratory testing and improvement with thyroxine therapy. Dogs with severe underlying diseases and low TT4 levels, but no clinical signs consistent with hypothyroidism were included in the NTIS group.

The study included 47 HD, 18 hypothyroid dogs and 15 dogs with NTIS. The reference interval for rT3 was 109–435 pg/ml using a robust, untransformed method. rT3 in dogs with hypothyroidism (<5–106 pg/ml) was lower than rT3 in dogs with NTIS (52.2–700 pg/ml; *P* = 0.004) and lower than in HD (111–817 pg/ml; *P* < 0.0001). Using a cut‐off value of <50 pg/ml specificity and sensitivity in detecting hypothyroidism was 100% and 83% rT3 seems to be useful for diagnosing canine hypothyroidism. Furthermore, a reference interval for rT3 (using HPLC‐MS/MS) in the dog was established.

**Disclosures**

Christina Schwens is employed by Antech Lab Germany which offers rT3 testing on a fee‐for‐service basis. The relationship does not have any impact of the data presented.

**Source of Funding**

This study received financial support through a research grand from DGVD (Deutsche Gesellschaft für Veterinärdermatologie = German Society for Veterinary Dermatology).

**ECVIM CSF Funding**

No.

**ESVE**—**European Society of Veterinary Endocrinology**

## ESVE‐O‐9

## Quantitative thyroid scintigraphy for the diagnosis of subclinical hypothyroidism in dogs

### D. Miceli^1^, J. Garcia^2^, M. Scarpa^3^

#### ^1^IBYME CONICET, Buenos Aires, Argentina; ^2^Department of Nuclear Veterinary Diagnostics, Western Veterinary Hospital, Bueno, Buenos Aires, Argentina; ^3^Private practice, Buenos Aires, Argentina

Subclinical hypothyroidism characteristically presents with increased serum thyroid stimulating hormone (TSH) and normal thyroxine (TT4) concentrations. In several conditions in dogs, it has been described that an increase in TSH does not necessarily represent thyroid dysfunction. Thyroid scintigraphy is one of the gold standards for the diagnosis of canine hypothyroidism and could potentially serve as a diagnostic tool for dogs with thyroid hypofunction showing increased TSH and normal TT4. The aim of this study was to assess whether quantitative thyroid scintigraphy could be used for the identification of dogs with subclinical hypothyroidism. In this study, quantitative thyroid scintigraphy was performed in 25 dogs with markedly increased TSH (>1 ng/ml) and TT4 within the reference interval (1–3 μg/dl), 10 dogs with clinical hypothyroidism (increased TSH and decreased TT4), 9 dogs with non‐thyroidal illness (NTI), and 12 healthy control dogs. The scintigraphy was performed 20 min after the administration of 0.5 mCi/kg IV (18.5 MBq/kg) sodium pertechnetate (Na^99m^TcO4‐), and the thyroid‐to‐salivary (T/S) ratio was calculated. Median T/S ratio was 0.3 (range 0.1–0.41) in hypothyroid dogs, 0.7 (range 0.12–2) in dogs with increased TSH and normal TT4, 1.52 (range 0.92–3.4) in dogs with NTI, and 1.1 (range 0.9–2) in healthy dogs. Significant differences were observed between dogs with increased TSH/normal TT4 and dogs with clinical hypothyroidism, dogs with NTI and healthy dogs (P=0.007, P=0.006, P=0.047, respectively). Among the dogs with increased TSH/normal TT4, 36% (9/25 cases) had a T/S ratio (median 0.37, range 0.12–0.49) in the same range as dogs with clinical hypothyroidism. These dogs had mild clinical signs compatible with hypothyroidism (lethargy, overweight, and dermatological problems). Likewise, 20% (5/25 cases) of dogs with increased TSH and normal TT4 had a T/S ratio in a “grey zone” between the upper limit of the range for dogs with clinical hypothyroidism and the lower limit of the range for healthy dogs. And the remaining 44% (5/25 cases) had a T/S ratio in the same range as healthy dogs. This study suggests that quantitative thyroid scintigraphy may help identify dogs that present thyroid hypofunction with increased TSH and normal TT4. Similarly, this study provides evidence that not all dogs with increased TSH and normal TT4 have thyroid hypofunction, highlighting the relevance of performing quantitative thyroid scintigraphy to assess thyroid function in dogs with equivocal thyroid profile results.

**Disclosures**

**Source of Funding**

**ECVIM CSF Funding**

No.

**ESVE**—**European Society of Veterinary Endocrinology**

## ESVE‐O‐10

## Re‐evaluating diagnostic cut‐off values: Impact of Immulite‐2000‐antibody change on canine hypercortisolism diagnosis using low‐dose dexamethasone suppression test

### G. Rossi^1^, F. Del Baldo^1^, A.M. Tardo^1^, S. Golinelli^1^, F. Fracassi^1^

#### ^1^University of Bologna Department of Veterinary Medical Sciences, Ozzano dell'Emilia, Italy

The low‐dose dexamethasone suppression test (LDDST) is a widely used test for canine hypercortisolism (HC). In 2020, there was a change in the Immulite‐2000‐antibody used for cortisol measurement and new investigations of LDDST are required. This study aimed to re‐evaluate the LDDST 8‐hour cortisol cut‐point for the diagnosis of canine HC using a chemiluminescent enzyme immunoassay (Veterinary Cortisol, Siemens, IMMULITE 2000 XPi) and to compare the diagnostic performance of LDDST before and after the change of Immulite‐2000‐antibody. The performance of LDDST was retrospectively evaluated comparing the results in dogs with HC and dogs with disease mimicking HC (DMHC). The diagnosis of HC was based on the presence of compatible clinical signs and at least one of the following criteria: post‐ACTH cortisol value >22 μg/dL; undetectable ACTH associated with an adrenal mass; pituitary‐to‐brain ratio >0.33; clear response to medical treatment. The diagnostic performances of the test were evaluated before (T1, January 2016–October 2020) and after (T2, November 2020–January 2024) the change in the Immulite‐2000‐antibody. Dogs that received glucocorticoids in the previous 90 days or lacked data for diagnosis confirmation were excluded. The difference between 8‐hour cortisol at T1 and T2 in HC dogs was assessed using the Mann–Whitney test. Performance of the LDDST was assessed using sensitivity, specificity and a receiver operating characteristic (ROC) curve. The LDDST was performed on 63 dogs with HC and 32 dogs with DMHC at T1, and 40 dogs with HC and 40 dogs with DMHC at T2. The median (range) 8‐hour cortisol value in HC dogs was 3.35 μg/dL (0.5–25 μg/dL) at T1 and 2.4 μg/dL (0.3–8.9 μg/dL) at T2 (*p* = 0.02). At T1 and T2, the area under the curve for 8‐hour cortisol to differentiate HC from DMHC dogs was 0.95 (95% CI 0.91–0.99) and 0.97 (95% CI 0.94–1.0), respectively. The cut‐point associated with the best sensitivity and specificity to diagnose HC was >1.4 μg/dL (Se=88%, 95% CI 77%–94%; Sp=97%, 95% CI 84%–100%) at T1 and >1.2 μg/dL (Se=88%, 95% CI 73%–96%; Sp=98%, 95% CI 87%–100%) at T2. In conclusion, when using IMMULITE 2000 XPi, the optimal cut‐point of LDDST 8‐hour cortisol for HC diagnosis was >1.2 μg/dL, which is lower than the currently accepted cut‐point of >1.4 μg/dL. Clinicians should consider this updated cut‐off value when performing an LDDST for the diagnosis of HC.

**Disclosures**

Francesca Del Baldo: Speaking & consultancies: MYLAV Veterinary Laboratory, Boheringer Ingelheim, Dechra, Royal Canin; Antonio Maria Tardo: Financial support: Ph.D. scholarship funded by Monge. Speaking & consultancies: Dechra, MYLAV Veterinary Laboratory; Stefania Golinelli: Financial support: Ph.D. scholarship funded by Dechra Speaking & consultancies: Dechra; Federico Fracassi: Financial support: Dechra, MSD, Monge. Speaking & consultancies: Boehringer Ingelheim, Dechra, MSD, Royal Canin, Hill's, Nestlé Purina, MYLAV Veterinary Laboratory.

**Source of Funding**

**ECVIM CSF Funding**

No.

**ESVE**—**European Society of Veterinary Endocrinology**

## ESVE‐O‐11

## Implementing salivary cortisol testing in a clinical setting: Feasibility and usefulness of at‐home salivary sampling to monitor trilostane treatment in dogs with Cushing's syndrome

### S.M. Meunier^1^, E. Neubert^2^, L.A. Harburger^2^, C.E. Reusch^2^, C. D. Voegel^3^, F.S. Boretti^2^, N.S. Sieber‐Ruckstuhl ^2^

#### ^1^Clinic for Small Animal Internal Medicine, Vetsuisse Faculty, University of Zurich, Switzerland; ^2^Clinic for Small Animal Internal Medicine, University of Zurich, Zurich, Switzerland; ^3^Department of Nephrology and Hypertension, Inselspital, Bern University Hospital, Bern, Switzerland

At‐home salivary cortisol testing, proven useful in humans, could also benefit dogs by reducing the impact of in‐hospital fear on cortisol measurements. Sampling with a ginger powder embedded cotton swab and measuring salivary cortisol concentrations by liquid chromatography tandem mass spectrometry (LC‐MS) has shown promising results in experimental dogs and requires validation in clinical settings.

Therefore, the aims of this study were to evaluate practical feasibility of salivary sampling in client‐owned dogs, and to compare the performance of at‐home and in‐hospital salivary cortisol concentrations for monitoring trilostane treatment.

Saliva was collected from healthy staff‐owned dogs (controls) at the hospital, and at home by the owners. For trilostane‐treated dogs, saliva was collected at the hospital immediately before blood sampling for prepill serum cortisol measurements. Owners were instructed to collect saliva at home. Saliva sampling involved holding a Salimetrics® cotton swab dipped in ginger powder in the dogs' mouths for 30 seconds. Salivary cortisol concentrations were measured using LC‐MS, while serum cortisol was assessed using chemiluminescence immunoassay. Dogs were considered well‐controlled based on the resolution of clinical signs. The target range of serum prepill cortisol concentration was set at 28–140 nmol/L. Statistical analyses were conducted in GraphPad Prism® 10, using non‐parametric tests. Significance was defined as a *p*‐value < 0.05.

Sufficient saliva volume was retrieved at both locations from all 10 controls. No significant difference in salivary cortisol concentrations between the two settings was observed (*p* = 0.541).

Thirty‐eight trilostane‐treated dogs were included. In‐hospital sampling failed in 20 (due to defensive movements or attempts to bite) but was successful in the remaining 18. In 7 of the 18, owners could collect saliva at home. Salivary cortisol concentrations were significantly lower at home compared to in‐hospital (*p* = 0.016).

Six of those 7 dogs were clinically well‐controlled; the uncontrolled one had the highest at‐home salivary cortisol concentration. Three of the 6 well‐controlled dogs had serum cortisol pre‐pill values above the target range. At‐home salivary cortisol concentration was below the detection limit in 2 of them and lower that for the uncontrolled dog in the third one.

Implementing this saliva sampling technique in client‐owned trilostane‐treated dogs was proven to be challenging. Nevertheless, the higher in‐hospital salivary cortisol concentrations in these dogs and inappropriately high pre‐pill values in well‐controlled dogs underscore the limitations of in‐hospital sampling. If owners can collect saliva at home, it might present a promising approach to monitor trilostane treatment in dogs with Cushing's syndrome.

**Disclosures**

The authors have the following disclosures related to their presentation Current/past employee/salary: none Investments/commercial interests: none Patents: none Speaking engagements (last 5y): Dechra Veterinary Products Ltd, Unisvet, Eickemeyer Veterinary Technology for life, Hippiatrika Verlag Baden‐Baden Consultancies (last 5y): Dechra Veterinary Products Ltd, Boehringer Ingelheim, Idexxs Grants/research (last 5y): Swiss National Foundation; Novartis Foundation for Medical biological Research; Albert‐Heim Foundation, Bern, Switzerland; Foundation for Research in Science and the Humanities at the University of Zurich; Dr. Gerber‐ten‐Bosch Foundation, Zurich, Switzerland Gifts, hospitality, travel support: Dechra Veterinary Products Ltd, Hill's Pet Nutrition, Nestle Purina, Scil animal care Memberships: ECVIM‐CA, ESVE, ACVIM

**Source of Funding**

Albert‐Heim Foundation, Bern, Switzerland.

**ECVIM CSF Funding**

No.

**ESVE**—**European Society of Veterinary Endocrinology**

## ESVE‐O‐12

## Performance of an in‐clinic point‐of‐care cortisol immunoassay for dogs with hypoadrenocorticism

### A. Torrano Guillamón^1^, C. Juárez Sarrión^1^, E. Prosper Asensi^1^, J. Castro López^1^, P. García San José ^2^, C. Arenas Bermejo^1^

#### ^1^Anicura Valencia Sur Veterinary Hospital, Valencia, Spain; ^2^Complutense University. Department of Animal Medicine and Surgery., Madrid, Spain

Dogs with hypoadrenocorticism (HA) can present with a wide range of clinical signs, some presenting as an emergency. Therefore, a simple, quick turn‐around test for diagnosis is desirable. Although in‐clinic cortisol measurement is commonly used, the precision of certain analyzers remains questionable.

The purpose of this study was to compare cortisol measurements obtained by a rapid point‐of‐care ELISA (Idexx) (SNAP) with those obtained by a veterinary reference laboratory (chemiluminescent enzyme immunoassay‐Immulite 2000 (VRL)).

A prospective study was conducted including dogs for which HA was a differential diagnosis. Dogs were allocated into either the HA group (post‐ACTH (VRL) cortisol <2 μg/dL) or to the non‐HA group (NHA) (basal serum cortisol (BSC) >2μg/dL or post‐ACTH cortisol ≥ 2 μg/dL). Each serum sample was analyzed in‐house using the SNAP test and an aliquot was submitted to the VRL for blinded analysis. Left‐over blood was used for the study. The lower limit of quantification for both assays was 0.5μg/dL.

In total 121 cortisol paired‐samples were analyzed (HA group=19 dogs (3 eukaliemic‐eunatriemic); NHA group 59 dogs). 8 dogs couldn't be allocated to either group but BSC was used for correlation analysis. The SNAP showed good correlation to the VRL method (Spearman's rho=0.924; *p* < 0.001). 35 ACTH‐stimulation tests (ACTHst) were performed. Median BSC and post‐ACTH cortisol concentrations measured by both methods were not significantly different (p ≥ 0.05; Mann–Whitney U test).

A cut‐off value of <2μg/dL BSC was 100% sensitive to detect HA with both methods. Specificity was 72,9% (SNAP) and 67,8% (VRL). A cut‐off value of <2μg/dL post‐ACTH cortisol was 100% specific for both methods; however, sensitivity was 89.5% for the SNAP (2/19 dogs with HA had post‐ACTH cortisol ≥2μg/dL; post‐ACTH cortisol concentrations for these dogs were 2.0μg/dL and 2.2μg/dL; one was hyperkalemic/hyponatremic and the other was hyponatremic). If the dog with post‐ACTH cortisol concentration of 2.0μg/dL would have been allocated into the HA group, sensitivity for the SNAP would be 94.7%

A cut‐off value of BSC ≤1,4 μg/dl (SNAP) maintained 100% sensitivity and improved specificity for detecting HA up to 78% (area under the ROC curve= 0.926; 95% CI= 0.870–0.983; *p* = 0.000).

In conclusion, the SNAP assay exhibited good correlation with VRL method. Despite the high sensitivity for HA, caution is advised due to the possibility of misdiagnosis. Dogs with SNAP ACTHst results near decision thresholds for HA should undergo further testing with the VRL. Nevertheless, these results suggest that BSC measured by SNAP allows HA to be ruled out in more patients, compared to the VRL method.

**Disclosures**

‐Carolina Arenas: Speaking & consultancies: Dechra consulting; Boehringer Ingelheim. ‐Jorge Castro: Speaking & consultancies: Zoetis consulting; Boehringer Ingelheim. ‐Paula García San Jose: Funding, Purina and Royal canin. Idexx provided the SNAPs.

**Source of Funding**

Idexx provided the SNAPs.

**ECVIM CSF Funding**

No.

**ESVE**—**European Society of Veterinary Endocrinology**

## ESVE‐O‐13

## Potential factors that influence adrenocorticotropic hormone stimulation test results in suspect addisonian dogs

### A. J. Da Silva^1^, M. Oliveira^2^, C. T. Mooney^2^

#### ^1^University College Dublin Veterinary Hospital University College Dublin, Dublin, Ireland; ^2^University College Dublin Veterinary Hospital, Dublin, Ireland

Both basal and adrenocorticotropic hormone (ACTH) stimulated cortisol concentrations are frequently used for ruling out canine hypoadrenocorticism but published studies exclude cases where prior drug therapies/non‐adrenal illness that could potentially influence results exist. This does not represent field conditions where these factors cannot easily be excluded.

The aim of this study was to evaluate both basal and ACTH stimulated cortisol concentrations in dogs in which these results were required to rule out hypoadrenocorticism.

The hospital database was interrogated for dogs in which an ACTH stimulation test was performed but where an alternative diagnosis of non‐adrenal illness was made and treatment for hypoadrenocorticism considered unwarranted. Administration of corticosteroids within the previous 6 weeks was recorded.

In total, 115 dogs were identified. Corticosteroids had been administered in 45 (39.1%) dogs. Median basal, ACTH‐stimulated and delta cortisol were significantly (*P* < 0.001, *P* < 0.001 and *P*=0.005, respectively) lower in dogs that had received corticosteroids (78.4, interquartile range 27.6–194.0 nmol/L; 159.0, 73.1–320.0 nmol/L; and 53.8, 17.4–105.0 nmol/L; respectively) compared to dogs that had not (192.5, 103.0–326.8 nmol/L; 451.0, 312.0–661.3 nmol/L; and 160.0, 22.5–413.1 nmol/L).

Twenty (17.4 %) dogs had either a flat‐line (*n* = 11) or minimal (*n* = 8) cortisol (<100 nmol/L) response to ACTH; 17 (85.0%) had received corticosteroids. Three dogs had a repeat ACTH stimulation test ruling out hypoadrenocorticism. Of the remaining 28 (62.2%) dogs that had received corticosteroids, the results helped rule out hypoadrenocorticism. Thirty‐four (29.7%) dogs had a basal cortisol concentration >250 nmol/L, of which 8 (23.5%) had received corticosteroids. Median delta cortisol was not significantly (*P*=0.735) different between dogs that had received corticosteroids (65.0, –7.8 to 102.0 nmol/L) and those that had not (16.5, –68.3 to 132.8 nmol/L). There was a moderate negative correlation between basal cortisol and delta cortisol in these dogs (*r* = ‐0.452; P=0.007). Twenty‐one of these 34 (61.7%) dogs had flat‐line results despite high basal cortisol concentrations. Seventeen (81.0%) were presented in shock of which 7 were eventually euthanased or died.

Prior corticosteroid use, often unavoidable in clinical practice, has a potentially significant effect on both basal and stimulated cortisol results and must always be taken into consideration when such results suggest hypoadrenocorticism. Nevertheless, the results may help rule out hypoadrenocorticism in a good proportion of these dogs. Prior steroid use appears to have a less significant effect in dogs with higher basal cortisol concentrations. In accordance with previous studies, delta cortisol is a potential prognostic marker.

**Disclosures**

**Source of Funding**

**ECVIM CSF Funding**

No.

**ESVE**—**European Society of Veterinary Endocrinology**

## ESVE‐O‐14

## Canine pheochromocytoma organoids: An in vitro model to explore new treatment options

### M. van den Berg^1^, E. Timmermans‐Sprang ^1^, F. Viets^1^, M. van Hoef^1^, M. van Wolferen^1^, H. Kooistra^1^, S. Galac^1^

#### ^1^Faculty of Veterinary Medicine, Utrecht University Department of Clinical Sciences, Utrecht, Netherlands

Given the current lack of effective medical treatment for canine pheochromocytomas (PCCs), there is a clear need for an *in vitro* model to explore new treatment options. Organoids are self‐organizing, self‐renewing three‐dimensional cellular structures that closely resemble the organ or tumor they originate from. As such, organoid cultures can constitute a valuable disease modeling and drug screening platform. We aimed to establish and characterize organoid cultures of canine PCCs.

Tumor tissue was obtained from client‐owned dogs with PCC following surgical removal. The diagnosis of PCC was based on clinical presentation, presence of an adrenal mass, and increased plasma free metanephrines. The diagnosis was confirmed by histopathology and immunohistochemistry (IHC) using the adrenomedullary markers synaptophysin and chromogranin A. Primary cell suspensions were seeded in a 3D matrix (basement membrane extract) and cells were cultured with medium containing specific growth factors. Organoids were characterized using histology, IHC, immunofluorescence (IF), and qPCR analysis. Catecholamine and metanephrine production in the cell culture supernatant was measured by liquid chromatography‐tandem mass spectrometry.

Six PCC organoid lines were successfully established, which could initially be passaged after 3–6 weeks but exhibited decreased expansion after passage 1. Primary cell suspensions exhibited mRNA expression of adrenomedullary markers tyrosine hydroxylase and chromogranin A, while organoids had notably lower expression. Organoids also showed decreased immunostaining for chromogranin A and synaptophysin over time. Instead, organoids displayed increased mRNA expression of adrenomedullary progenitor markers such as nestin and vimentin, accompanied by immunostaining for these markers in both IHC and IF assays. Measurement of catecholamines and metanephrines in the cell culture supernatant showed a pattern that matched that of the primary tumors.

The expression profiles of these organoids, particularly the downregulation of adrenomedullary markers coupled with increased expression of progenitor markers, indicate a transition towards a progenitor phenotype. Ongoing research aims to optimize culture conditions to maintain organoid expansion over extended passages. In addition, research efforts towards differentiation of these organoids towards a chromaffin phenotype are ongoing.

The successful establishment of PCC organoid lines marks a significant advancement in the field. These organoid lines have great potential as an *in vitro* research tool, paving the way towards identification of new treatment modalities for this difficult‐to‐treat tumor type.

**Disclosures**

Marit van den Berg: no disclosures. Elpetra Timmermans‐Sprang: no disclosures. Fleur Viets: no disclosures. Marleen van Hoef: no disclosures. Monique van Wolferen: no disclosures. Hans Kooistra: member of the Global Diabetes Mellitus Advisory Board of Boehringer Ingelheim. Sara Galac: no disclosures.

**Source of Funding**

This study was supported by a donation from Friends of VetMed.

**ECVIM CSF Funding**

No.

**ESVE**—**European Society of Veterinary Endocrinology**

## ESVE‐O‐15

## Steroid profiling in canine adrenocortical incidentalomas

### K. van Bokhorst^1,2^, M. van den Berg^1^, H. Kooistra^1^, S. Galac^1^

#### ^1^Faculty of Veterinary Medicine, Utrecht University Clinical Sciences, Utrecht, Netherlands; ^2^IVC Evidensia Clinical Sciences, Vleuten, Netherlands

Seemingly hormonally silent adrenocortical tumors (SATs) are typically diagnosed incidentally during diagnostic imaging in dogs with either no or only mild clinical features suggestive of adrenal disease. While treatment options are clear for hormonally active adrenal tumors, SATs present a dilemma for the clinician. The aim of this study was to evaluate the production of adrenocortical steroids including those not typically included in standard endocrine tests in canine SATs, to investigate if these are truly hormonally silent.

Diagnosis of SAT was based on presence of an adrenal mass on diagnostic imaging and exclusion of naturally occurring hypercortisolism, hyperaldosteronism and pheochromocytoma through endocrine testing. Dogs with SATs underwent adrenalectomy and histopathology with immunohistochemistry was performed to confirm the tumor's adrenocortical origin. In total, 12 unilateral and two bilateral SAT cases were included. Metabolomic profiling was performed on fresh frozen tumor tissue, preserved after surgery. Steroid metabolites (*n* = 17) originating from all three adrenocortical zones were analyzed by liquid chromatography with mass spectrometry. Results were compared with metabolomic profiling of canine cortisol‐secreting adrenocortical tumors (csACTs, *n* = 10) and normal adrenal cortices (NACs, *n* = 10) from dogs euthanized for reasons unrelated to the present study. The Kruskal–Wallis test was used to compare results among the three groups, followed by Dunn–Bonferroni post‐hoc tests to assess differences between individual groups.

The results indicate that SATs exhibited significantly higher relative tissue cortisol concentrations than NACs (*p* = 0.036), and these concentrations were not different from those in csACTs (*p* = 0.322). Furthermore, SAT's tissue concentrations of mineralocorticoid precursors corticosterone and 18‐hydroxy‐corticosterone were significantly higher than those of NACs (*p* = 0.010 and *p* = 0.015, respectively) and not different when compared to csACTs (*p* = 0.348 and *p* = 0.999, respectively). No significant differences were found in progesterone and 17‐hydroxyprogesterone tissue concentrations between SATs and NACs.

This is the first study using advanced analysis of steroid hormone production in canine adrenal tumors not falling into disease categories of hypercortisolism, hyperaldosteronism and pheochromocytoma. Results of this study reveal steroid and steroid‐precursor production in SATs, which might be analogous to ‘mild autonomous cortisol secretion’ as described in humans. The finding that these tumors may not be as hormonally silent as thought before, prompts further studies on treatment and monitoring recommendations in affected dogs.

**Disclosures**

‐ K.L. van Bokhorst: national expert panel diabetes mellitus Boehringer Ingelheim—H.S. Kooistra: global diabetes advisory board Boehringer Ingelheim.

**Source of Funding**

Grant Friends of Veterinary Medicine.

**ECVIM CSF Funding**

No.

**ESVCN**—**European society of Veterinary & Comparative Nutrition**

## ESVCN‐O‐1

## Amino acid supplementation preserves protein turnover rate in obese dogs under weight loss

### L. Luis^1^, L. Pacheco^1^, C. Goloni^1^, S. Theodoro^1^, M.E. Gonçalves^1^, M. Monti^2^, A. Cavalieri Carciofi^1^

#### ^1^FCAV‐UNESP, Jaboticabal, Brazil; ^2^Special Dog Company, Santa Cruz do Rio Pardo, Brazil

Given the impact of calorie restriction on protein metabolism, it is necessary to investigate the requirements of amino acids during weight loss (WL) helping to mitigate lean mass loss during regimen. The study compared protein turnover rates (PTR) of client‐owned non‐obese and obese dogs, before and after loss of 20% of body weight, fed a control (CO) obesity diet or a treatment diet (AA) similar in composition, supplemented with methionine, tryptophan, valine, and threonine.

Twenty obese dogs (BCS 7–9/9; 43.1±1.3% fatty mass) and twenty non‐obese dogs (BCS 4–5/9; 23.4±1.4%) were used. At begining, 10 dogs of each body condition were fed CO (12.5 kJ/g; 32%CP; 25%dietary fiber) or AA diet to maintain constant weight for 15 days (Approval 07196/19). After, 13C‐Leucine method (13C‐Leu) was used to access PTR. Obese dogs were then subjected to a loss of 20% of weight, 10 with CO and 10 with AA treatment (BCS 5.6/9; 29.7±1.6% fatty mass; *P* < 0.01) and were tested again with 13C‐Leu. No losses of lean mass were observed (P=0.342). In 13C‐Leu test, dogs were fasted and the isotopes (2mg.kg^−1^of L‐[1‐13C] Leucine and 0.1mg.kg‐1 of [13C]NaH_13_CO_3_) infused. After 5 min, infusions of 0.7mg.kg^−1^of L‐[1‐13C] Leucine were given 7 times every 20min. Samples of expired gas and blood plasma were collected before, and 80, 100, 120, 140, 160, 180 and 200 min after enrichment. 13C was analysed (ABCA2, SerCon, UK), results used to calculate protein synthesis (S) and breakdown (B) and submitted to ANOVA considering effects of body composition (BC), diet, and interactions. For obese dogs, time was also considered inside each diet (*P* < 0.05).

Diet effect was observed, with higher S and B for AA dogs regardless of BC (*P* < 0.05). Dogs BC also influence PTR, with tendency for higher S and B for non‐obese (*P* = 0.058). Although time effect was not observed for PTR (*P* = 0.26), reduced S and B were observed for obese after loss when fed CO (*P* < 0.05). It suggests that AA supplementation increased PTR in non‐obese and obese dogs, and it supported PTR during regiment, as after weight loss protein S and B was similar with initial values in obese dogs fed AA but was lower with the CO diet.

Obese dogs presented lower PTR. The loss of weight reduced PTR in CO animals, but not in AA, which presented similar values than initial moment.

**Disclosures**

**Source of Funding**

**ECVIM CSF Funding**

No.

**ESVCN**—**European society of Veterinary & Comparative Nutrition**

## ESVCN‐O‐2

## Protein turnover of overweight and non‐overweight cats fed high starch or high protein kibble diets

### C. Goloni^1^, L. Pacheco^1^, L. Scarpim^1^, L. Luis^1^, S. Theodoro^1^, A. Cavalieri Carciofi^1^

#### ^1^São Paulo State University (UNESP), FCAV, Jaboticabal, Brazil

Optimum protein intake for adult cats is little investigated with protein turnover (PT), as the impact of body composition on protein requirements. It is known that obesity promotes insulin resistance might also induce alterations in amino acid uptake by peripheral tissues. The study evaluated PT in non‐overweight (NO) and overweight (OW) cats fed a high‐starch (HS) or high‐protein diet (HP) diet.

Castrated NO (*n* = 10; 3.9 ± 0.8kg; 4.1 ± 0.9 years; BCS: 5.0 ± 0.0/9; 16 ± 4% fatty mass [FM]) and OW cats (*n* = 6; 4.6 ± 0.8kg; 5.8 ± 1.2 years; BCS: 7.2 ± 0.1/9; 27 ± 3% FM) were used. In a crossover design, cats were fed for a constant body weight diets with similar fat and fibre contents: HS (32% Starch; 38% CP, 16.6 kJ/g ME, 1.5% phenylalanine [Phe] in dry matter [DM]) or HP (19% Starch; 55% CP; 17.1 kJ/g ME; 2.2% Phe in DM). After 21 days of diet intake PT was evaluated by^13^C‐Phe method. Cats were fed and after 60 min a primming dose (PD) of 0.18 mg/kg/^13^C‐Bicarbonate and 0.66 mg/kg/^13^C‐Phe was oral administered, followed by 25, 50, 75, 100, 125, 150 min, doses of 1.33 mg/kg/^13^C‐Phe. Expired air were collected using an adapted mask. After 50 min of PD, eight samples of expired air were collected at each 25 min. Breathed air samples were analysed for^13^CO_2_ (ABCA2, SerCon, Cheshire, UK). The enrichment of^13^C‐Phe was used to determine protein synthesis (S) and breakdown (B; g protein/kg^0.67^/day). Due to the different Phe contents of the HS and HP diet, a direct comparison between diets is not possible. Results were subjected to ANOVA considering NO versus OW cats, and their effect inside each diet (*P*<0.05).

Protein intake was lower for cats fed HS (5.9 ± 1.0 g/kg^0.67^/d) than HP diet (8.8 ± 1.4; *P*<0.05), and lower for OW than NO inside each diet (P<0.01). The general rates of protein S and B was similar between OW and NO cats (*P*=0.53; Table 1). When body composition was compared inside each diet, PT was similar for NO and OW cats fed the HS or HP diets (*P* > 0.05). OW cats fed the HP diet had 14.8% higher PT than NO animals, although not significant (*P*=0.32) this can be attributed to the higher lean body mass of OW cats (350g higher than of the NO; P=0.22).

OW and NO cats have similar PT, even when fed diets with different proportions of protein or starch. Further studies are required to better apply the^13^C‐Phe method in cat nutrition.

**Disclosures**

**Source of Funding**

BRF Ingredients; ADIMAX Pet Food; BRF Pet Food; Affinity PetCare Spain.

**ECVIM CSF Funding**

No.

**ESVCN**—**European society of Veterinary & Comparative Nutrition**

## ESVCN‐O‐3

## Environmental impacts of extruded or homemade dog foods determined by Life Cycle Assessment (LCA)

### A. Martins^1^, L. Henríquez^1^, J. Costa^1^, M. Monti^2^, R. Vasconcellos^1^

#### ^1^State University of Maringá Department of Animal Science, Maringá, Brazil; ^2^Special Dog Company, Santa Cruz do Rio Pardo, Brazil

Given the wide range of pet food products, that apply food or feed grade ingredients in the formulation, the objective of this study was to quantify by LCA, the environmental impacts related to the production of two extruded dry foods (Premium (PD) and Super Premium (SPD)) and a homemade diet (HMD) for adult dogs.

One food of each category (PD, SPD and HMD) was formulated based on a study of the ingredient and nutritional composition of products sold in the Brazilian pet food market. For the formulation of each food, the two most common ingredients by nutrient class (starch, fiber, protein, fat, and additives), and mean of nutrient concentration declared in the label were used. The system boundaries were “cradle to gate” (all impacts in the production and processing of ingredients and foods were considered, excluding the packaging, distribution, and use steps). The Functional Unit (FU) was considered the daily energy requirement of a dog with 10 kg, and the Reference Flux (RF) was the amount of food to supply the FU (PD = 160.9; SPD = 181.4; HMD = 463.6 g/day). The Ecoinvent and Agri‐footprint databases, available in the Simapro, were used for modelling the ingredients (secondary data). The primary data for PD and SPD were obtained from a Brazilian pet food company. For the HMD, primary data of the processing step were obtained during the preparation of the diet in a laboratory environment.

HMD presented the highest values for the 14 impact categories analysed. It was observed that the five most relevant impact categories for PD and SPD were freshwater ecotoxicity, terrestrial and marine eutrophication, acidification, and particulate matter, which together accounted, respectively, for 83.7% and 86.9% of the total impact. For HMD, the main categories were non‐cancerous human toxicity, freshwater ecotoxicity, terrestrial eutrophication, acidification, and particulate matter, which together represented 84.8% of the impacts. For the Climate Change category, it was estimated an emission of 50.92 for PD, 37.64 for SPD, and 1,058.26 kg of CO_2_ Eq‐/year for HD.

This study shows the high relevance of the formulation step in the eco‐design of pet food. The inclusion of vegetable and animal byproducts in the SPD and PD contributes for a lower impact than the HMD that was formulated with human grade ingredients. Regardless the niche of food production, the formulation based on the LCA is essential for the development of more sustainable foods for pets.

**Disclosures**

In this study, co‐author Mariana Monti works for a Pet Food company (Special Dog). However, the partnership with the company only occurred to obtain Simapro software and primary data (regarding transport, processing, distribution, electric and thermal energy, water use, and waste) for LCA analysis of extruded dry foods evaluated in this study. To obtain data from the software and presentation of these in the summary, there was no interference from the company.

**Source of Funding**

Special Dog Company (MANFRIM INDL E COML LTDA).

**ECVIM CSF Funding**

No.

**ESVCN**—**European society of Veterinary & Comparative Nutrition**

## ESVCN‐O‐4

## Hermetia illucens diet improved gut microbial metabolic activity in healthy dogs

### C.G. Vecchiato^1^, F. Sportelli^1^, C. Pinna^1^, C. Delsante^1^, B. Di Gregorio^1^, G. Biagi^1^, F. Del Baldo^1^

#### ^1^Department of Veterinary Medical Sciences, University of Bologna, Ozzano dell'Emilia, Italy

Insects represent an alternative and sustainable protein source for petfood, and *H. illucens* larvae (HI) is one of the most promising species for monogastric animal nutrition in terms of nutrient profile and sustainability.

This study aimed to compare two diets different in protein source, containing either HI or poultry (CON) meal with regard to nutrient digestibility and faecal microbial parameters in healthy dogs. Twelve adult dogs (8 females and 4 males, aged 70 ± 37 months) were involved in a cross‐over study and assigned to receive either the HI (on dry matter (DM) basis: crude protein (CP) 25.7%, fat (EE) 13.7%, starch 33.1%, crude fiber (CF) 4.8%, ash 8.8%) or the CON (CP 26.9%, EE 13.6%, starch 37.6%, CF 2.9%, ash 8.2%) in a 2×4‐week period (HI‐CON or CON‐HI), with 7‐day transition between periods. Silica was included in both diets (1%) to assess the apparent total tract digestibility (ATTD, %) for DM and CP. On the last 5 days of each feeding period, faecal aliquots were sampled and pooled for ATTD, while a single sample was analysed for moisture, pH, NH_3_, volatile fatty acids (VFA), and selected bacterial populations by qPCR. Differences between values were tested by a GLM model fitting a cross‐over design with diet (HI and CON) and period (HI‐CON or CON‐HI) as fixed factors, and subjects as random factors; period and diet x period interaction were tested to exclude any carry‐over effect. Data about HI vs. CON (mean ± SD) are reported. Statistical significance was set at p<0.05. The ATTD (%) of DM and CP did not differ (*p* > 0.05) when dogs were fed HI compared to CON (DM: 78 ± 4 vs. 79 ± 3; CP: 79 ± 5 vs. 78 ± 3). When dogs were fed HI compared to CON, moisture was +3% (67 ± 3 vs. 65 ± 3%, *p* = 0.02) and NH_3_ was ‐55% (30 ± 28 vs. 67 ± 28 μmol/g). Among faecal VFA (%), propionate was +30% (35 ± 6 vs. 27 ± 6 p<0.01), n‐butyrate was ‐18% (9 ± 2 vs. 11 ± 2), iso‐butyrate was ‐36% (1.6 ± 1 vs. 2.5 ± 1) and iso‐valerate was ‐12% (2.2 ± 1.6 vs. 3.7 ± 1.7) for HI compared to CON (*p*<0.01). The qPCR revealed that HI compared to CON enriched the concentration of *Bifidobacterium* spp. (4.0 ± 1.4 vs. 4.7 ± 1.6 Log DNA, *p* = 0.045). HI diet did not affect nutrient digestibility compared to CON diet when fed to healthy dogs. Moreover, findings from this study suggest a change in dogs’ gut microbial metabolic activity, with lower bacterial protein catabolism and potential prebiotic effects exerted by the insect meal.

**Disclosures**

Carla Giuditta Vecchiato, Speaking & consultancies: Monge, MSD, Prolife‐Zodiaco, Royal Canin. Sportelli Federica, consultancies: Necon Pinna Carlo: no disclosures to report Delsante Costanza, consultancies: Amusi Di Gregorio Barbara: no disclosures to report Biagi Giacomo, Speaking & consultancies: Candioli, Innovet, Aurora biofarma, Purina, CIAM, Ecuphar, Elanco, Prolife‐Zoodiaco. Del Baldo Francesca, Speaking & consultancies: MYLAV Veterinary Laboratory, Boheringer Ingelheim, Dechra, Royal Canin.

**Source of Funding**

This study was funded by Dorado srl, a pet food company which provided the diets used in the study.

**ECVIM CSF Funding**

No.

**ESVCN**—**European society of Veterinary & Comparative Nutrition**

## ESVCN‐O‐5

## Urine as alternative fluid for the doubly labelled water method use in dogs with chronic kidney disease (preliminary evaluation)

### A. de Castro^1^, T.G. Bitencourt Freire^1^, J. de Castro Fenerick^1^, L. Araújo Silva^1^, M.E. Gonçalves Tozato^1^, S. de Souza Theodoro^1^, L.G. Pacheco^1^, C. Goloni^1^, L. Mantovani Volpe^2^, V. Eliodoro Costa^3^, A. Cavalieri Carciofi^1^

#### ^1^São Paulo State University (UNESP), Jaboticabal, Brazil; ^2^Adimax Pet, Salto de Pirapora, Brazil; ^3^São Paulo State University (UNESP), Botucatu, Brazil

Stable isotopes are promising metabolic tracers for studying animals affected by various clinical conditions. In the doubly labelled water (DLW) method, the body water is enriched with deuterium (^2^H) and oxygen‐18 (^18^O), and its concentrations are measured in blood, saliva, or urine to estimate energy expenditure (EE), body composition, and water turnover rate (WTR). Dogs with chronic kidney disease (CKD) commonly have polyuria, what could affect the results when using this fluid. This study aimed to compare urine to standard blood to measure isotopes concentration to apply DLW method in CKD dogs.

Five dogs (5.2 ± 1.4 years old) with CDK stage 2 and 3 (creatinine: 2.3 ± 0.3 mg/dL; SDMA: 17.4 ± 2.7ug/dL; urine specific gravity: 1.010 ± 0) received a dose of 0.12g of^2^H_2_O at 99.9 atm% and 2g of H_2_^18^O at 10 atm% per Kg of body water subcutaneously after 12 h of food and 1 h of water fasting. Blood and urine samples were collected before (basal) and after 3 h (enrichment), 2, 4, and 6 days (elimination) and analyzed using isotope ratio mass spectrometry (IRMS‐EA, Thermo Fisher Scientific, Waltham, MA, USA). Means were compared using ANOVA and Student's t‐test, and the agreement between fluids using Bland–Altman and Pearson correlation (P<0.05).

The isotopic concentrations at basal and elimination moments were similar in blood and urine fluids (*P* > 0.05). The^2^H and^18^O concentration in plasma and urine during the basal and elimination periods presented a low bias (0.2 SD 3.7 and −0.1 SD 6.0 ppm, respectively), high precision (1.0), mean absolute error of 1.91 and 3.4, root mean square error of 3.6 and 5.8, high concordance correlation coefficient (0.99), and very high Pearson correlation (*ρ* 0.99; *P*<0.001). However, during the enrichment period, isotopes concentration was lower in urine than blood (*P*<0.05), probably requiring more time to equilibrate isotopes, as the initial samples may include urine previously stored in the bladder. In comparison with the use of blood, adopting urine results for basal and elimination measurements, and blood to access enrichment resulted in similar calculations of the water space pool, lean and fat body mass (kg), EE (404.6 ± 63.7 and 382.9 ± 68.7 kJ/kg^0.75^/d) and WTR (110.6 ± 17 and 114.0 ± 33.6 mL/kg^0.75^/d) for the CDK dogs>(*P* > 0.05).

More investigations to establish the optimal time for urine collection during enrichment are required. Urine can be safely used for measuring the concentration of ²H and^18^O pre‐application and during elimination as a replacement for blood while using DLW in dogs with CKD.

**Disclosures**

**Source of Funding**

**ECVIM CSF Funding**

No.

**ESVCN**—**European society of Veterinary & Comparative Nutrition**

## ESVCN‐O‐6

## Hydrolyzed poultry byproduct meal effects on inflammatory and lipid parameters of dogs

### T. De Souza Avida^1^, L.B.S. Bassi Scarpim^2^, C. Cristina de Oliveira^2^, A.P. Garcia Gonçalves^2^, P. Ricardo^2^, S. De Souza Theodoro^2^, C. Goloni^2^, E. Cristina de Ramos^2^, A. Cavalieri Carciofi^2^

#### ^1^São Paulo State University—UNESP, Jaboticabal, Brazil; ^2^São Paulo State University, Jaboticabal, Brazil

Protein hydrolysates have low molecular weight, high digestibility and may have functional bioactive peptides that modify metabolic and physiological responses. Our aim was to compare the effects of diets with increasing amounts of hydrolysed poultry by‐product meal (HPM) replacing conventional low ash poultry by‐product meal (PBM) as protein source, verifying effects on serum concentrations of total cholesterol, high‐density lipoprotein (HDL), low‐density lipoprotein (LDL), triglycerides and cytokines of dogs.

Four extruded diets with similar chemical compositions were used: Control (PBM), and the replacement of 25%, 50% and 100% of PBM by HPM. Thirty‐two health Beagle dogs were distributed in a randomized block design (13.3 ± 0.4 kg; 4.1 ± 1.5 years), 8 dogs per food. After 30 days of food intake, blood was collected from the jugular vein (fasting 8h) and total cholesterol, HDL, LDL and triglycerides were analysed (Labtest Diagnóstica S.A, Lagoa Santa, Brazil). The cytokines TNF‐a, IL‐2, IL‐6, IL‐10 were analysed using a commercial kit (MILLIPLEX MAP Canine Cytokine/Chemokine Magnetic Bead Panel, Merck Millipore). Data were submitted to variance analysis and means compared by polynomial contrasts according to the HPM inclusion level. Serum cytokine and LDL did not comply with ANOVA assumptions and were compared using Kruskal–Wallis test (*P*<0.05). Approval number: 7511/2022.

The 100% HPM diet reduced LDL by 44.6% compared to the control diet (PBM) (*P*=0.03). Other differences in lipid parameters were not observed (*P* > 0.05). Decreased LDL concentration was also observed in mice fed chicken liver hydrolysate, although the mechanisms are still unknown. IL‐2 and TNF‐α were lower in the 100% HPM diet than in the other diets (*P*<0.05), and IL‐6 and IL‐10 were lower in the 100% HPM diet than in the 25% HPM and 50% HPM diets (*p*<0.05), with the control diet presenting intermediate values. In dogs with suspected or confirmed chronic enteropathy, lower *ex vivo* production of IL‐10 and TNF‐α was demonstrated in animals fed hydrolysed protein compared to intact protein. Hydrolysed protein contains peptides that are small enough to prevent IgE‐mediated food allergy by preventing recognition of two IgE antibody receptors on a mast cell. The present study opened a potential mechanism of action of hydrolysed proteins, since the replacement of PBM (intact protein) by HPM induced a significant reduction in systemic inflammation in health dogs.

Health dogs fed 100% HPM as protein source presented lower serum LDL, IL‐2, TNF‐α, IL‐6 and IL‐10 concentrations, which justifies further studies in animals with food allergy.

**Disclosures**

**Source of Funding**

**ECVIM CSF Funding**

No.

**ESVCN**—**European society of Veterinary & Comparative Nutrition**

## ESVCN‐O‐7

## Ultra‐low fat home cooked diets improve clinical scores and albumin in dogs with protein losing enteropathy refractory to standard management with low fat diets and immunomodulation

### A. Kavanagh^1,2^, J. Rudinsky^1,2^, A. Winston^1,2^, P. Parker^1,2^

#### ^1^Veterinary Clinical Sciences, Ohio State University College of Veterinary Med Department of Veterinary Clinical Sciences, Columbus, Ohio, United States; ^2^Comparative Hepatobiliary Intestinal Research Program (CHIRP) Department of Veterinary Clinical Sciences, Columbus, Ohio, United States

Protein‐losing enteropathy (PLE) is a chronic, life‐threatening gastrointestinal disease. Treatment consists of nutritional management, anti‐coagulants, dietary supplements (e.g. cobalamin), and immunosuppressive medications. Dietary fat restriction is the mainstay of nutritional management; however some dogs may require even more fat restriction than is available in commercial dog foods formulated for fat‐responsive disease. The aim of this retrospective study was to evaluate the response to ultra‐low‐fat home‐cooked diets (ULFD) in dogs with PLE that had failed to respond to standard low‐fat diets in conjunction with other standard treatments. Nutrition service records were searched for dogs with a clinical diagnosis of PLE who at time of evaluation had failed low fat diet trials using commercial diets at the same time as standard immunomodulation protocols. Cases were then considered eligible for inclusion if the only treatment change initiated was transitioning to a ULFD. Cases in which other supplements or medications were changed in addition to the ULFD were excluded. Eleven dogs were identified (5 male castrated, 6 female spayed). Median body weight was 15.3 kg (range, 5.9‐33.6). Clinical signs were variable: diarrhea (72%), weight loss (45%), lethargy (45%), effusions (36%), vomiting (9%), pruritus (9%). Immunosuppresant medications included corticosteroids (82%), cyclosporine (36%), and alkylating agents (27%). Commerical low fat diets included Purina EN Low Fat (27%), Royal Canin Gastrointestinal Low Fat (27%), Hill's Prescription Diet i/d Low Fat (27%), and Purina HA Vegetarian (18%). Dietary fat concentration was higher in commercial low‐fat diets fed at enrollement (median 1.9 g/100 kcal; range 1.7–2.7 g/100kcal) compared to home cooked ULFDs (median 1.4 g/100kcal; range 1.1–1.6 g/100 kcal) (P=0.001). Besides fat concentration, ULFDs were formulated to meet the Association of American Feed Control Officials minimum requirements for canine adult maintenance. CCECAI scores at baseline (median 3.5, range 1–11) were higher than after ULFD management (median 2.5, range 0–8) (P=0.04). After switching to ULFDs, clinical signs completely resolved in 4 dogs, partially resolved in 4 dogs, and failed to respond in 3 dogs. Albumin significantly improved from baseline (median 2.1 mg/dl, range 1.4–3.4) to first recheck on ULFDs (median 2.4 mg/dl, range 1.7–2.8) (P=0.04). Reducing dietary fat intake beyond what can be achieved with commercial low‐fat diets marketed for use in fat‐responsive diseases improves outcome in some dogs with PLE. Randomized clinical trials are necessary to further compare the response between ULFD diets and other treatments in dogs with PLE.

**Disclosures**

Drs. Rudinsky, Winston, and Parker receive consulting fees and/or speaker honoraria from multiple pet food companies including Purina, Hill's, and Royal Canin.

**Source of Funding**

This study was not funded.

**ECVIM CSF Funding**

No.

**ESVCN**—**European society of Veterinary & Comparative Nutrition**

## ESVCN‐O‐8

## High calorie, nutrient dense diet supports body weight in dogs with malignant cancer

### M. Amundson^1^, I. Becvarova^1^, J. Brejda^2^, M.L. Higginbotham^3^, P. Macias^3^

#### ^1^Hill's Pet Nutrition, Inc., Topeka, United States; ^2^Alpha Statistical Consulting, Lincoln, United States; ^3^Kansas State University, Manhattan, United States

Anorexia, weight loss, and decreased body condition scores have been associated with reduced quality of life (QoL) and survival in dogs with cancer. The study objective was to evaluate feeding a new diet to support dogs with malignant cancer.

The test group consisted of 34 dogs fed Hill's Prescription Diet ONC Care Canine dry for a 56‐day period with a 7‐day transition, and the control group consisted of 26 dogs fed their original food. All dogs were diagnosed with malignant cancer and underwent concurrent cancer treatment. From the test group, 21 dogs completed the 56‐day feeding period. Intake (grams and calories), body weight (BW), body condition score (BCS; 1=very thin, 5=obese), muscle condition score (MCS; 1=normal, 4=severe loss), body fat index (BFI; 20=low risk, 70=extreme risk), eating enthusiasm (1=lowest, 7=highest), stool quality (1=liquid, 6=firm), blood laboratory tests, and (QoL) were recorded. Only BW and BCS on day 0 and 56 ± 3 were collected for the control group. Average age, BW and BCS of the control group at day 0 was 8.4 y, 24.9 kg, and 3.7, respectively. Average age, BW, BCS, MCS, and BFI of the test group at day 0 was 9.2 y, 24.9 kg, 3.6, 1.1, and 27.1%, respectively. This study was approved by the Institutional Animal Care and Use Committee.

Gram intake was unchanged (*P*=0.5513), but caloric intake tended to decrease (*P*=0.0532) from day 8 to 56. In both groups, BW and BCS increased from day 0 to 56, but BW response was two‐fold larger in the test group (1.2 kg, SE 0.3, *P*=0.0008) compared to the control group (0.6 kg, SE 0.3, *P*=0.0700). MCS and BFI remained unchanged, and normal stool quality was observed throughout the study. Owners reported dogs in the test group maintained a high (6.0) average eating enthusiasm and QoL throughout the study. From the QoL questionnaires, a decrease in wanting to be near the owner (*P*=0.0320) and enjoyment of affection from family (*P*=0.0253) was observed from day 0. Canine specific pancreatic lipase decreased (*P*=0.0619), and serum C‐reactive protein and 25 OH‐vitamin D were unchanged from day 0 to 56 in the test group.

Dogs fed the test food gained BW, while maintaining BCS, normal stools, and a high QoL. Additionally, dogs in the test group showed no evidence of fat intolerance or pancreatic disease. Overall, this study demonstrated that the test food may be an appropriate nutritional support for dogs with malignant cancer.

**Disclosures**

Madison D. Amundson and Iveta Becvarova are employees of Hill's Pet Nutrition, Inc. John Brejda is a subcontractor of Hill's Pet Nutrition, Inc. Mary Lynn Higginbotham and Paulina Macias were employees at Kansas State University at the time this research was conducted.

**Source of Funding**

This study was funded by Hill's Pet Nutrition, Inc.

**ECVIM CSF Funding**

No.

**ESVCN**—**European society of Veterinary & Comparative Nutrition**

## ESVCN‐O‐9

## Impact of the dietary supplementation of sunflower oil, fish oil and lard on the urine composition and selected blood values of healthy adult cats

### N. Paßlack^1,2^, S. Müller^3^, K. Büttner^4^, J. Zentek^5^

#### ^1^Small Animal Clinic, Justus‐Liebig‐University Giessen, Giessen, Germany; ^2^Chair for Animal Nutrition and Dietetics, Ludwig‐Maximilians‐Universität München, Munich, Germany; ^3^LABOKLIN GmbH & Co. KG, Bad Kissingen, Germany; ^4^Unit for Biomathematics and Data Processing, Justus‐Liebig‐University Giessen, Giessen, Germany; ^5^Institute of Animal Nutrition, Freie Universität Berlin, Berlin, Germany

Data from human medicine indicate that the dietary fat concentration or specific fatty acids, like arachidonic acid, might impact the risk for the development of calcium oxalate uroliths. In addition, it has been described that the supplementation of n‐3‐fatty acids can lower blood triglycerides and alter blood cholesterol levels in human subjects.

In the present study, the relevance of the dietary fat supply was investigated in healthy adult cats. Ten cats received a commercial low fat basic diet without or with the addition of sunflower oil, fish oil or lard in a randomized cross‐over design. The fat supplements were added daily to the basic diet at 0.5 g or 1 g per kg body weight (BW) of the cats. By this, varying fat concentrations and fatty acid patterns were achieved. Each dietary treatment was applied for 3 weeks, where fasting blood as well as urine samples were collected in the last week each.

The results demonstrated that neither the dietary fat concentration nor the dietary fatty acid pattern influenced the urinary calcium and oxalate concentrations and the relative supersaturation of the urine with calcium oxalate. All blood values were within the reference range. However, the comparison of the three dietary fat sources revealed that only fish oil affected indicators of the feline lipid metabolism. Plasma total cholesterol and low density lipoprotein cholesterol concentrations were increased, when the cats received 1 g fish oil/kg BW/day (*P* = 0.0186 and *P* = 0.0167), while plasma triglycerides were lowered by the supplementation of 0.5 g fish oil/kg BW/day (*P* = 0.0268).

The present data indicate that the dietary fat concentration and fatty acid pattern might not be a specific risk factor for the development of calcium oxalate stones in cats. The observed effects of the fish oil supplementation on markers of the feline lipid metabolism are in accordance with data from human medicine. Their clinical relevance, however, warrants further investigation.

**Disclosures**

The study was partly supported by the Waltham Foundation Grant. One of the authors (S.F.M.) is an employee of the laboratory Laboklin, Bad Kissingen, Germany, where some of the analyses of the present study were performed.

**Source of Funding**

The study was partly supported by the Waltham Foundation Grant.

**ECVIM CSF Funding**

No.

**ESVCN**—**European society of Veterinary & Comparative Nutrition**

## ESVCN‐O‐10

## Analysis of the impact of nutritional supplementation on canine gut microbiota through an in vitro colonic fermentation model

### G. Pignataro^1^, L. Clerico^2^, B. Belà ^3^, A. Gramenzi^3^

#### ^1^Department of Veterinary Medicine, University of Teramo Department of Veterinary Medicine, University of Teramo, Italy, Teramo, Italy; ^2^Consultant in medical writing, Savona, Italy; ^3^Department of Veterinary Medicine, University of Teramo, Teramo, Italy

Modern science states that improving gut health is essential to guarantee dogs an optimal quality of life. The Simulator of the Canine Intestinal Microbial Ecosystem (SCIME^TM^) was recently developed and validated to offer a proper platform to analyze the effects of nutritional interventions on the canine gut microbiota. Given the high similarity between the human and dog intestinal conditions, the general reactor (SHIME®) was adapted to simulate the canine intestinal ecosystem. The study aims to evaluate the impact of a feed product on the composition and metabolic activity of healthy canine donors gut microbiota. The nutritional supplementation is Microbiotal (NBF Lanes), a complementary feed that includes in its composition dried oligofructose, microencapsulated tributyrate, inulin, and inactivated *Lactobacillus reuteri*.

The properties of Microbiotal were evaluated using the microbiota of a healthy ± 20 kg canine donor using the SCIME^TM^platform. After inoculation of the colon reactors with the fecal sample, the experiment followed three steps: a stabilization period that allowed the microbial community to differentiate (3 weeks); a control period (3 weeks) during which an analysis of the sample determined the baseline microbial community composition and activity; a treatment period of two weeks where the SCIME^TM^was operated with a diet supplemented with Microbiotal on top of the normal composition. Dietary supplementation effects were evaluated at the level of the composition (using the 16S‐targeted Illumina sequencing) and metabolic activity (through the continuous monitoring of the acid/base consumption, the concentration of short‐chain fatty acids—SCFA ‐, lactate, ammonium, and branched SCFA) of the luminal and mucosal gut microbiota.

The supplementation of the Microbiotal product enhanced acidification in both the proximal and distal colon, which was linked with significant stimulation of acetate production in the proximal colon (*p*<0.05). Concerning markers for proteolytic fermentation, a trend towards increased ammonium and branched SCFA levels was observed in the distal colon. Repeated administration of Microbiotal significantly enriched different bacterial groups involved in primary substrate degradation (*Bifidobacterium*, *Limosilactobacillus*, *Prevotella*, *Bacteroides*, and *Enterococcus*) in the proximal colon, indicating a boost of saccharolytic fermentation within the canine microbial community.

Acetate is one of the critical metabolites formed during primary substrate fermentation that a wide range of gut microbes can produce. Its significant increase in the proximal colon confirms the Microbiotal beneficial effect. This work highlights how a feed containing prebiotics and bacterial fermentation products can diversify the bacterial flora and enhance the modulatory effects on the gut microbiota.

**Disclosures**

**Source of Funding**

**ECVIM CSF Funding**

No.

## ECVIM_Poster_sorted

**ESVC**—**European Society of Veterinary Cardiology**

## ESVC‐P‐1

## Immune dysregulation in dogs with myxomatous mitral valve disease: Insights into T lymphocyte and monocyte subtypes and natural killer cells

### M. Cimerman^1^, A. Nemec Svete^1^, S. Petek^2^, M. Bajc^2^, K. Pohar^2^, A. Ihan^2^, A. Domanjko Petrič ^1^

#### ^1^Small Animal Clinic, Veterinary faculty, University of Ljubljana, Ljubljana, Slovenia; ^2^Institute of Microbiology and Immunology, Faculty of Medicine University of Ljubljana, Ljubljana, Slovenia

Dogs with myxomatous mitral valve disease (MMVD) show alterations in T lymphocyte (CD3+) subpopulations. Recent studies in humans with congestive heart failure (CHF) have additionally shown a reduction in natural killer (NK) cells and differences in monocyte subtypes compared to healthy subjects.

Our study aimed to investigate the immune status of dogs with MMVD at different stages of the disease compared to healthy dogs, with a particular interest in monocyte subtypes and NK cells, previously unstudied, and CD3+ lymphocyte subtypes.

Eighty‐one client‐owned dogs were included in this prospective cross‐sectional study: 64 dogs with MMVD at various stages of the disease and 17 healthy dogs. The dogs with MMVD were categorized into three groups according to the American College of Veterinary Internal Medicine (ACVIM) classification: ACVIM B2 (preclinical; 23), ACVIM C (stable CHF; 22) and ACVIM C and D (unstable CHF; 19). Flow cytometry was performed on the blood samples to determine the percentage of total and activated CD3+ lymphocytes and their subpopulations (T helper lymphocytes (CD3+CD4+), cytotoxic T lymphocytes (CD3+CD8+), regulatory T lymphocytes (Treg; CD3+CD4+FOXP3+), double positive T lymphocytes (DPT; CD3+CD4+CD8+), double negative T lymphocytes (DNT; CD3+CD4‐CD8‐)), B lymphocytes (CD45+CD21+), NK cells (CD3‐CD21‐CD5‐/dim) and total monocytes (CD11b+), subdivided into three subtypes (CD14+MHCII‐, CD14+MHCII+, and CD14‐MHCII+).

Kruskal‐Wallis analysis with pairwise comparisons and Bonferroni correction was used to compare immune variables between groups. P values <0.05 were considered significant.

Our results showed that the percentage of total monocytes was significantly higher in dogs with stable and unstable CHF compared to preclinical dogs. The dogs with stable CHF had a significantly higher percentage of CD14+MHCII+ monocytes compared to the healthy dogs. The percentage of activated CD3+CD4+ lymphocytes was significantly lower in the stable CHF group compared to all other groups. No significant differences were found between the dog groups for CD3+CD8+, DPT, DNT, Treg, B lymphocytes or NK cells.

In conclusion, the alteration of CD14+MHCII+ monocytes indicates a possible role of these cells in promoting chronic inflammation in MMVD. The low percentage of activated CD3+CD4+ lymphocytes suggests a reduced activation or functional response of T helper cells, which play an important role in the pathophysiology of chronic CHF in MMVD dogs. Contrary to our expectations, NK cells showed no direct role in the pathophysiology of MMVD in dogs. The results of this study may contribute to a better understanding of the immune dynamics in MMVD‐affected dogs.

**Disclosures**

**Source of Funding**

Slovenian research and innovation agency (research program P4‐0053).

**ECVIM CSF Funding**

No.

**ESVC**—**European Society of Veterinary Cardiology**

## ESVC‐P‐2

## An outcome‐based severity classification of left heart enlargement in dogs with myxomatous mitral valve disease

### T. Vezzosi^1^, G. Grosso^1^, A. Della Pina^1^, M. Croce^2^, H. Broch^3^, R. Tognetti^1^, O. Domenech^2,3^

#### ^1^Department of Veterinary Sciences, University of Pisa, Pisa, Italy; ^2^Anicura Istituto Veterinario di Novara, Novara, Italy; ^3^Anicura Clinica Veterinaria CMV Varese, Varese, Italy

The echocardiographic assessment of left heart enlargement plays a key role in the management of dog with myxomatous mitral valve disease (MMVD). The left atrium‐to‐aorta ratio (LA/Ao), the left atrial diameter normalized for body weight (LADn) and the left ventricular internal diastolic diameter normalized for body weight (LVIDDn) are the most used parameters of left heart enlargement in both clinical research and practice. The aim of this study was to propose an outcome‐based severity classification of left atrial and ventricular enlargement based on these three parameters.

This retrospective, multicenter, cohort study included dogs with MMVD imaged between 2011 and 2021. The LA/Ao was measured in 2D from the right‐parasternal short‐axis view. The LADn was measured in 2D from the right‐parasternal long‐axis view and calculated as LAD(mm)/kg^0.324^. The LVIDDn was measured in M‐mode from the right‐parasternal short‐axis view and calculated as LVIDD(mm)/kg^0.294^/10. The prognostic value of LA/Ao, LADn and LVIDDn was tested by evaluating their association with the median time to cardiac event, defined with the occurrence of congestive heart failure or cardiac death. Long‐term outcome was assessed by telephone interviews with the owners. Median time to cardiac event was analysed using Kaplan–Meier curves and logrank tests.

A total of 386 dogs with MMVD were included, with 209 in stage ACVIM B1, 90 in stage ACVIM B2 and 87 in stage ACVIM C, of which 136 (35%) reached the cardiac endpoint according to an updated follow‐up in December 2023. A Kaplan‐Meier curve was drawn for each value of LA/Ao, LADn and LVIDDn, and based on the cardiac outcome three severity classes were defined for each parameter. Regarding LA/Ao: <1.70 normal‐to‐mild, 1.70–2.00 moderate, >2.00 severe. Regarding LADn: <17.0 normal‐to‐mild, 17.0–20.0 moderate, >20.0 severe. Regarding LVIDDn: <1.7 normal‐to‐mild, 1.70–2.00 moderate, >2.00 severe. The median time to cardiac event was significantly different (P<0.0001) between all the severity classes: LA/Ao, normal‐to‐mild [2502 days, 95% confidence interval (CI) 2344–2604 days), moderate (1588 days, 95% CI 1176–1725 days), severe (343 days, 95% CI 229–488 days); LADn, normal‐to‐mild (2502 days, 95% CI 2085–2604 days), moderate (1341 days, 95% CI 810–1610 days), severe (324 days, 95% CI 181–514 days); and LVIDDn, normal‐to‐mild (2604 days, 95% CI 2344–2604 days), moderate (1341 days, 95% CI 1139–2085 days), severe (314 days, 95% CI 198–429 days).

In conclusion, the proposed outcome‐based severity classification of left heart enlargement gives the possibility to objectively standardize the definition of the degree of cardiac remodeling in dogs with MMVD.

**Disclosures**

**Source of Funding**

**ECVIM CSF Funding**

No.

**ESVC**—**European Society of Veterinary Cardiology**

## ESVC‐P‐3

## Retrospective review of the use of hydrochlorothiazide in dogs with myxomatous mitral valve disease

### A. Boscia^1^, R. Toschi Corneliani^1^, M. Caccia^1^, V. Caroli^1^, A. Galizzi^1^, I. Spalla^1^

#### ^1^Ospedale Veterinario San Francesco Cardiology Department, Milan, Italy

The use of hydrochlorothiazide (HTCZ) is recommended in dogs with myxomatous mitral valve disease (MMVD) refractory to congestive heart failure management (American College of Veterinary Internal Medicine, Stage D). Hydrochlorothiazide is prescribed in addition to loop diuretics to achieve sequential nephron blockade in order to enhance diuresis. Although its use is common in this group of patients, information on outcome, survival and complications are not widely available, hence the aim of the present retrospective study.

Over the last 3 years, 24 dogs were prescribed and received HTCZ as part of multimodal treatment of refractory congestive heart failure. All dogs were diagnosed with MMVD stage D from a single referral centre. Data retrieved included treatment, available bloodwork before and after starting HTCZ treatment, echocardiographic assessment, additional diagnostics (ECG and/or thoracic radiographs) and survival data.

Twenty‐one dogs were receiving torasemide at a mean dose of 0.6mg/kg/day and three dogs received furosemide at a mean dose of 8mg/kg/day alongside with HTCZ. All dogs were also receiving pimobendan (0.7mg/kg/day), benazepril (0.45mg/kg/day) and spironolactone (4mg/kg/day). Four dogs were receiving antiarrhythmic treatment for concurrent supraventricular arrhythmias. Hydrochlorothiazide was prescribed at a median starting dose of 1.2mg/kg every 48hrs (range: 0.4–2mg/kg q24–48h PO). The latest median recorded HTCZ dose was 1.6mg/kg/day (range: 0.5–3mg/kg q12–24h PO). Seventeen dogs died, all of which due to cardiac disease. Median survival time in the overall population was 229 ± 113 days. Blood venous gas data was available in 21 dogs prior to and 18 dogs following HTCZ administration. Mean creatinine value was 1.10mg/dl prior HTCZ and 1.96mg/dl at the latest available control (*p* = 0.040). Azotaemia was identified in 3 dogs prior to HTCZ treatment, and in 10 dogs following HTCZ addition. An overall reduction of serum electrolytes concentration was noted, yet it did not reach statistical significance (*p* = 0.08 for potassium, 0.489 for sodium and 0.653 for chloride difference). Only one dog had clinically relevant side effects (severe azotaemia and inappetence) requiring discontinuation of the drug.

In conclusion, HTCZ was a feasible addition in dogs with advanced MMVD, improving clinical signs and outcome. Although it affected renal values, negligible clinical side effects were observed in the vast majority of cases.

**Disclosures**

**Source of Funding**

No funding used.

**ECVIM CSF Funding**

No.

**ESVC**—**European Society of Veterinary Cardiology**

## ESVC‐P‐4

## Blood transcriptome profiles in healthy cats compared to cats with asymptomatic and symptomatic hypertrophic cardiomyopathy

### T. Mangklabruks^1^, P. Chuammitri^2^, S.D. Surachetpong^1^

#### ^1^Faculty of Veterinary Science, Chulalongkorn University, Bangkok, Thailand; ^2^Faculty of Veterinary Medicine, Chiang Mai University Faculty of Veterinary Medicine, Chiang Mai University, ChiangMai, Thailand, ChiangMai, Thailand

Next generation sequencing (NGS) technology offers a deeper understanding of mRNAs profiles, or transcriptome, associated with diseases of interest. Hypertrophic cardiomyopathy (HCM) is a hereditary disease in human and cats. Extensive studies on mRNAs have significantly advanced our understanding, diagnosis and treatment of this condition. Feline HCM is the most prevalent heart disease in cats, while it shares similar structural and histopathological features with human HCM, our understanding of the feline transcriptome in this context remains limited.

This study aimed to utilize NGS technology to define the transcriptome profiles of healthy cats and those with HCM. We further sought to identify differences in circulating gene expression associated with various stages of the disease. This study included ten cats classified according to the ACVIM staging system for HCM: stage A (*n* = 4), stage B (*n* = 3), and stage C (*n* = 3). White blood cell samples were collected from these cats. NGS data was then used to identify differentially expressed genes (DEGs) between the different HCM stages. Cats with HCM stages A/C exhibited the greatest number of DEGs, with 832 genes were down‐regulated, and 898 genes were up‐regulated. Key DEGs (fold change > 2; FDR < 0.01), potentially involved in HCM pathophysiology were identified. These DEGs including *RAB25, CYTB, MMP8, EDNRB, ND1, GYG1, EGR1*, and *COL1A1*. HCM stages B/C showed 228 down‐regulated, 108 up‐regulated genes. Key DEGs included *HTRA3*, *PITPNM3*, *IL13*, *CDK4*, and *CACNG5*. Stages A/B of HCM showed minimal expression changes (25 down‐, 29 up‐regulation), but key DEGs (*RAB25*, *CYTB*, *LRRC71*, *CALR3*, *EGFR*, *COL4A4*, and *KCNB1*) were linked to HCM.

Among DEGs identified between healthy (A) and HCM cats (B and C), Cytochrome b (*CYTB*) emerged as particularly interesting. Notably, *CYTB* exhibits similar dysregulation in human HCM, suggesting its potential as a circulating biomarker for feline HCM. This study pioneers the use of RNA‐seq to identify blood‐based gene expression differences between healthy cats and those with various stages of HCM. Notably, we identified novel genes not previously associated with the heart or HCM in either humans or cats. Further investigation is warranted to elucidate their roles in cardiac function and the pathogenesis of feline HCM.

**Disclosures**

**Source of Funding**

**ECVIM CSF Funding**

No.

**ESVC**—**European Society of Veterinary Cardiology**

## ESVC‐P‐5

## Phonocardiographic features in cats with obstructive hypertrophic cardiomyopathy

### M. Dhunputh^1^, L. Desquilbet^2^, V. Saponaro^1^

#### ^1^National Veterinary School of Alfort Cardiology, Maisons‐Alfort, France; ^2^National Veterinary School of Alfort Biostatistics, Maisons‐Alfort, France

Phonocardiography (PCG) intervals reflect the mechanical activity of the heart. Our hypothesis is that obstructive hypertrophic cardiomyopathy (OHCM), cause of obstruction, has a different mechanical activity with respect to the other feline population and that this could be shown by PCG.

The aim of this study was to compare the phonocardiograms of OHCM cats to those of non OHCM cats.

We retrospectively searched our phonocardiographic database for cats undergoing a full echocardiography and good quality phonocardiograms at the same time.

Based on echocardiography, cats were grouped according to 4 different phenotypes: HCM, OHCM, mitral regurgitation (MR) and healthy (H). Clinical data were also collected including signalment, auscultation findings (murmur, third sound) and presence of congestive heart failure. Cats were excluded if they received betablockers. Phonocardiography and echocardiographic examinations were performed by a senior cardiologist, phonocardiographic measurements by a junior clinician supervised by the senior.

Phonocardiographic measurements included: amplitude(_A_) and duration(_D_) of S1, S2, S3, S4, S1' (in case of SAM: systolic anterior motion of the mitral valve); S1_A_/S2_A_ ratio; S1–S2 interval (systole duration: Sys), S2–S1 interval (diastole duration: Dia); Sys/Dia ratio. Sys and Dia were normalized (nSys, nDia) by dividing to S1–S1 interval (cardiac cycle). Presence of simultaneous ECG on phonocardiograms was also reported.

Median and interquartile range were measured for all metric variables and comparison was done between groups with Kruskal–Wallis test.

Among 247 phonocardiograms, 128 were included: OHCM (79), HCM (16), H (24) and MR (9). Simultaneous ECG was available for 41 cats.

Main results are as follows: for OHCM with a left ventricular outflow tract peak gradient > 50 mmHg, S1' was found on phonocardiograms in 93 % of these cats. It was always absent in non OHCM cats.

Normalized Sys and S1_A_/S2_A_ were significantly higher in OHCM (median and range 0.49 [0.46;0.54] and 3.02 [2.38;3.66] respectively) compared to cats without OHCM (median and range 0.45 [0.42;0.48] and 2.33 [1.72;3.13] respectively) (*p* ≤.0001 and *p* = 0.0004). Compared to HCM (median and range 0.45 [0.42; 0.47]), nSys was significantly higher in OHCM (*p* = 0.002) but no significant difference was found for S1_A_/S2_A_ ratio.

In conclusion, this study shows that OHCM cats have different phonocardiographic features from the other feline population, probably reflecting a specific mechanical activity in this disease.

**Disclosures**

**Source of Funding**

**ECVIM CSF Funding**

No.

**ESVC**—**European Society of Veterinary Cardiology**

## ESVC‐P‐6

## Transient restrictive cardiomyopathy phenotype in cats: first reported cases (*n* = 7)

### C. Poissonnier^1,2^, P. Guigo^2^, P. Foulex^3^, P. Passavin^4^, A. Fouhety^2^, T. Douay^2^, C. Le Gall^2^, V. Chetboul^1,5^

#### ^1^Ecole Nationale Vétérinaire d'Alfort, Maisons‐Alfort, France; ^2^Clinique Vétérinaire Tourainevet, Rochecorbon, France; ^3^Sudvetia, Aix‐en‐Provence, France; ^4^Centre Hospitalier Vétérinaire Atlantia, Nantes, France; ^5^U955—IMRB Inserm, École Nationale Vétérinaire d'Alfort, UPEC, Maisons‐Alfort, France

Transient myocardial thickening has been well described in cats. This clinical entity is characterized by left ventricular (LV) hypertrophy initially mimicking hypertrophic cardiomyopathy, often associated with left atrial (LA) dilation and congestive heart failure (CHF), that progressively resolves during follow‐up with a good long‐term prognosis. To the best of our knowledge, no transient restrictive cardiomyopathy (TRCM) phenotype has been described in cats so far. The aim of this retrospective case series was therefore to describe the historical, epidemiological, clinical, echocardiographic, and outcome of cats diagnosed with a TRCM phenotype identified by echocardiography. Cats diagnosed with a reversible RCM phenotype were searched in the databases of three referral centers and reviewed by a board‐certified cardiology specialist. As proposed by the ACVIM consensus statement on feline cardiomyopathies, the RCM phenotype was defined by normal LV dimensions (including wall thickness) with LA or biatrial enlargement. Seven cats were included. The median (interquartile range;minimum‐maximum) age and body weight at diagnosis were 1.6 years (0.7–8.1;0.5–11.0) and 3.5 kg (3.2–4.4;2.5–7.5), respectively. An antecedent event was identified 24 h to 17 days (median=7 days) before the TRCM phenotype diagnosis in 6/7 cats, i.e., neutering (*n* = 3), respiratory diseases (rhinitis and bronchopneumonia, *n* = 2), and glucocorticoid injection for feline eosinophilic granuloma treatment (*n* = 1). Thoracic radiographs and echocardiography confirmed signs of CHF in 6/7 cats, i.e., pulmonary edema (*n* = 5) and/or pleural effusion (*n* = 3). The TRCM phenotype was characterized by normal LV dimensions, with LA and biatrial enlargement in 6/7 and 1/7 cats respectively. The median end‐diastolic LA:Aorta ratio at diagnosis was 1.8 (1.5–1.8;1.4–1.9), LA enlargement being defined as an end‐diastolic LA:Aorta >1.2. A restrictive mitral inflow Doppler pattern characterized by a mitral E:A rat>o >2 (median=3.4 [2.8–3.7]) was observed in the 4/7 cats for which mitral E and A diastolic waves were not fused. The median time from diagnosis of the RCM phenotype to evidence of its echocardiographic resolution was 189 days (80–200; 14–422), with a final median end‐diastolic LA:Aorta ratio of 1.0 (0.98–1.03;0.86–1.09) and E:A ratio of 1.1 (0.5–1.9). When prescribed, antithrombotic treatment was discontinued for all cats (*n* = 4), and furosemide treatment was discontinued for 5/7 cats. Serum ultrasensitive cardiac troponin I was available in 2 cats. Values were markedly increased at diagnosis (18.3 and 4.3 ng/mL) and normalized during follow‐up (0.01 and 0.06 ng/mL, respectively). This case series shows that TRCM phenotype occurs in cats, with a good prognosis, and is often associated with an antecedent event.

**Disclosures**

Camille Poissonnier: speaking and consultancy for Vetoquinol, Dechra, Boeringher Ingelheim unrelated with the subject of the study.

**Source of Funding**

no funding was needed for this study.

**ECVIM CSF Funding**

No.

**ESVC**—**European Society of Veterinary Cardiology**

## ESVC‐P‐7

## Neutrophil‐to‐lymphocyte ratio and platelet‐to‐lymphocyte ratio in cats with arterial thromboembolism

### S. Oh^1^, J. Park^1^, Y.J. Hong^2^

#### ^1^Western Referral Animal Medical Center Department of Veterinary Internal Medicine, Seoul, South Korea; ^2^Western Referral Animal Medical Center Department of Veterinary Surgery, Seoul, South Korea

Feline arterial thromboembolism (FATE) is a life‐threatening condition frequently associated with underlying cardiac diseases, such as hypertrophic cardiomyopathy, and is characterized by its sudden onset and high mortality rate. The Neutrophil to Lymphocyte Ratio (NLR) and Platelet to Lymphocyte Ratio (PLR) serve as biomarkers for systemic inflammation and the balance of immune response. This study aimed to assess the prognostic value of NLR and PLR in predicting survival outcomes for cats diagnosed with FATE, alongside evaluating the effects of successful blood flow restoration and the impact of altered physiological parameters.

In our retrospective review, we analyzed the clinical records of forty‐eight client‐owned cats treated for FATE, dividing them into two groups based on post‐initial hospitalization outcomes: a survival group (18) and a non‐survival group (30).

Neither the NLR (*p* = 0.41) nor the PLR (*p* = 0.24) significantly differentiated between the groups, indicating their limited utility as prognostic markers in FATE cases. However, the analysis revealed that the restoration of blood flow significantly correlated with increased survival rates (p<0.0001). Additionally, cats that did not survive exhibited significantly higher respiratory rates (*p* = 0.046) and blood pressures (*p* = 0.018) compared to the survivors. Factors such as age, body weight, breed, sex, heart rate, white blood cell count, hematocrit, platelet count, creatinine, or blood urea nitrogen values showed no significant differences between the two groups.

These findings highlight the challenges in predicting outcomes for FATE, indicating that a multifactorial approach to prognostication is essential. Although markers such as NLR and PLR may not independently predict outcomes in this feline population, the study suggests the significance of effective reperfusion. Furthermore, it identifies elevated respiratory rates and blood pressures as critical physiological parameters that negatively affect survival chances.

**Disclosures**

**Source of Funding**

**ECVIM CSF Funding**

No.

**ESVC**—**European Society of Veterinary Cardiology**

## ESVC‐P‐8

## Ionized hypocalcemia as a prognostic indicator in cats with arterial thromboembolism

### G. Mampaey^1^, M. Rysenaer^1^, B. Broeckx^2^, P. Smets^1^

#### ^1^Ghent University, Faculty of Veterinary Medicine, Small Animal Department, Merelbeke, Belgium; ^2^Ghent University, Department of Veterinary and Biosciences, Merelbeke, Belgium

Arterial thromboembolism (ATE) is a common complication of feline cardiomyopathy. Prognosis is poor with 30%–45% of cats surviving to discharge and worsens when cats develop hyperkalemia (reperfusion injury). Hypocalcemia is reported in 20%–38% of cats presented with ATE and it has been suggested to be associated with development of hyperkalemia. However, no data on the prognostic ability of hypocalcemia are currently available.

The first aim of this study was to investigate the association between hypocalcemia and survival in cats with ATE. The second aim was to determine if development of hypocalcemia during hospitalization could predict development of reperfusion injury and death.

Twenty‐eight client‐owned cats presented with ATE were included in a prospective cohort study. Upon admission, results from physical examination, hematology, biochemistry, and serum electrolytes including potassium, sodium, chloride and ionized calcium (iCa) were collected. Electrolyte measurements were repeated at 12, 24 and 48 h after admission and/or when signs of reperfusion injury were suspected (according to the monitoring protocol for ATE cats in our hospital). All iCa measurements were performed on leftover samples. Cats were divided into two groups: cats that survived until discharge without development of reperfusion injury (survivors) and cats that died from or were euthanized after development of reperfusion injury (non‐survivors). To assess the association between outcome (“survivor” or “non‐survivor”) and various factors at admission, a Wilcoxon rank sum test (continuous variable) or Fisher exact test (categorical variable) was used.

Nine cats (9/28) had normal iCa upon admission, of which eight survived until discharge (88.9%) and one developed reperfusion injury during hospitalization (11.1%). Nineteen cats (19/28) had mild to severe ionized hypocalcemia upon admission (67.9%) of which 13 developed reperfusion injury (68.4%) and six (31,6%) survived until discharge. In all cats developing reperfusion injury, treatment with intravenous glucose, insulin and calcium gluconate was initiated. However, all died or were euthanized. Decreased iCa upon admission, presence of hind limb paralysis, decreased rectal temperature and presence of congestive heart failure were significantly associated with decreased survival (*P* = 0.02; *P* < 0.01; *P* < 0.01; *P* = 0.01, respectively). A significant decrease in iCa concentration during hospitalisation was present in non‐survivors (*P* < 0.001), which was not detected in survivors.

In conclusion, ionized hypocalcemia is common in cats with ATE. Ionized hypocalcemia upon admission or hypocalcemia that develops during hospitalization was associated with development of reperfusion injury and death.

**Disclosures**

**Source of Funding**

**ECVIM CSF Funding**

No.

**ESVC**—**European Society of Veterinary Cardiology**

## ESVC‐P‐9

## A case series of 5 cats with bacterial pericarditis with purulent pericardial effusion

### B. Bichelberger^1^, A.C. Merveille^1^, K. Gommeren^2^, G. Guyon^3^, C. Damoiseaux^3^

#### ^1^Department of clinical science University of Liège Department of clinical science University of Liège, Liège, Belgium; ^2^Department of clinical science University of Liège, Liège, Belgium; ^3^Frégis Veterinary Hospital Center, Paris, France

Bacterial pericarditis is a rare cause of pericardial effusion in cats, with only 6 case reports published in the literature. Only one had an identifiable cause (a grass awn foreign body) and bacteremia was suspected in 4 cats. Treatment including antibiotics, pericardiocentesis and pericardectomy has been reported, with 4 out of 6 cats surviving to discharge. This retrospective case series describes the clinical presentation, management, and outcome of 5 cats with bacterial pericarditis. Cases were 3 domestic cats, 1 maine coon, and 1 chartreux, including 3 males and 2 females. The median (range) age and weight at presentation were 6.5 years (0.4–10) and 2.5 kg (1.9–4.8). Main presenting complaints were anorexia (3), lethargy (3), and dyspnea (2). On physical examination, muffled heart sounds (4), hypothermia (3) and respiratory discordance (2) were the most common abnormalities. Initially 2 cats had neutropenia and 3 cats had a severe left shift confirmed on blood smear. Point‐of‐care ultrasound revealed pericardial and pleural effusion in all cats, associated with abdominal effusion in 2. Cardiac tamponade was identified in only a single case. Initial pericardiocentesis revealed an exudate (5) with intracellular bacteria (4). Pericardial drainage was performed using a single pericardiocentesis (1) or placement of a pericardial drain (4), after which cats were hospitalized (duration 4–9 days), receiving antibiotic mono‐ (2) or combination therapy (3), including amoxicillin‐clavulanic acid, enrofloxacin, penicillin, marbofloxacin, and trimethoprim/sulfamethoxazole. Echocardiography was performed on all cats and revealed valve abnormalities suggestive of endocarditis and suspicion of myocarditis in one cat. Thoracic CT scan showed no visualization of infectious foci or foreign bodies (2). Culture and sensitivity testing of pericardial effusion revealed Neisseria animaloris (3) and Streptococcus canis (2), which all were sensitive to the initial antibiotic(s). Pericardectomy was performed in one cat. One cat died during hospitalization secondary to septic shock. Histopathological examination of the pericardium of these two cats confirmed pyogranulomatous pericarditis and fibropurulent pericarditis, respectively. Four cats survived to discharge, with 3 cats fully recovered with a survival time of 25, 93 and 121 days after diagnosis and 1 cat lost to follow‐up. This case series described 5 cats with bacterial pericarditis, of which 4 survived until discharge. The origin of the bacterial pericarditis was unclear in all cases, however Neisseria animaloris, a common pathogen of the oral cavity, was isolated in 3 cats, suggesting a hematogenous spread secondary to periodontal disease, as described in endocarditis.

**Disclosures**

**Source of Funding**

**ECVIM CSF Funding**

No.

**ESVC**—**European Society of Veterinary Cardiology**

## ESVC‐P‐10

## Usefulness of tomographic measurements of vertebral heart volume ratio and vertebral heart scale in canine heartworm disease

### J. Matos^1^, S. García‐Rodríguez^1^, E. Mohr^1^, D. Vera^1^, E. Carretón^1^, J. Montoya‐Alonso ^1^

#### ^1^Internal Medicine, Faculty of Veterinary Medicine, Institute for Research in Bio, Las Palmas de Gran Canaria, Spain

Currently, computed tomography (CT) plays an important role in the diagnosis of heart disease and can provide prognostic information when echocardiography is not available. Tomographic measurements of vertebral heart volume ratio (VCVR) and vertebral heart scale (VHS) have previously shown high correlation with their respective measurements by radiological study, and have been especially accurate in the diagnosis of cardiac diseases that produce a not radiographically evident cardiomegaly. The present study aimed to evaluate the usefulness of VCVR and VHS in the diagnosis of pulmonary hypertension (PH) in dogs suffering from heartworm disease (HWD).

A total of 31 dogs diagnosed with HWD by antigen detection test and 30 healthy cardiorespiratory dogs were analyzed using the same CT equipment and following the same anesthetic protocol. Through echocardiography, the presence of PH was diagnosed in 13 dogs with HWD (Tricuspid regurgitation pressure gradient >3.4m/s and right pulmonary artery distensibility index <30%). Dogs were kept in sternal recumbency and post‐contrast positive pressure CT scans were performed after administration of 2.0 ml/kg iobitridol (300 mgI/ml). Helical cross‐sectional images were acquired using a slice thickness of 1.0 mm at 120 kVp, 200 Ma, and a tube rotation time of 0.6 s. For optimal CT images and better evaluation of the analyzed structures, a soft tissue window configuration was used (WW = 360; WL = 60). The tomographic measurements of VHS and VCVR were carried out by analyzing the cardiac dimensions and the vertebral bodies following guidelines established in previous studies carried out in dogs, using sagittal and transverse CT plane images respectively.

The results showed that the average values of VHS and VCVR were significantly higher (Mann–Whitney U test <0.5) in the animals suffering from HWD and PH (11.19 and 54.02 cm^2^), with respect to healthy dogs (8.31 and 39.23 cm^2^) and dogs with HWD and without PH (8.92 and 43.33 cm^2^). On the other hand, no significant differences were observed (Mann–Whitney U te>t >0.5) between healthy animals and dogs with HWD and without PH.

Tomographic determinations of VHS and VCVR have proven to be useful in the diagnosis of myxomatous mitral valve disease and canine dilated cardiomyopathy. However, its use in the diagnosis of canine PH has not been implemented. In this study, the results show that cardiovascular changes secondary to the presence of HWD generate cardiomegaly and both VHS and VCVR can be useful measurements in the analysis of PH caused by HWD.

**Disclosures**

**Source of Funding**

**ECVIM CSF Funding**

No.

**ESVC**—**European Society of Veterinary Cardiology**

## ESVC‐P‐11

## Evaluation of the ratio between the thickness of the bronchial wall and the pulmonary artery using Computed Tomography in cats seropositive to Dirofilaria immitis

### S. García‐Rodríguez^1^, S. Falcón‐Cordón^2^, N. Costa‐Rodríguez^1^, D. Vera‐Rodríguez^1^, E. Carretón^2^, A. Montoya‐Alonso ^2^

#### ^1^Internal Medicine, Faculty of Veterinary Medicine, Research Institute of Biomedi Internal Medicine, Faculty of Veterinary Medicine, Research Institute of Biomedi, Las Palmas, Spain; ^2^Internal Medicine, Faculty of Veterinary Medicine, Research Institute of Biomedi, Las Palmas, Spain

Computed tomography (CT) has been used for the assessment of the feline bronchial structures, damaged by inflammatory lung pathologies. The quantification of bronchial wall thickness has previously been described as an effective parameter to detect bronchiectasis in veterinary medicine. Cats with Heartworm‐Associated Respiratory Disease (HARD) often suffer from dilated bronchial lumen because of the potent inflammatory response resulting from exposure to *Dirofilaria immitis*.

The aim of this study was to compare, using CT, the relationship obtained between the thickness of the bronchial wall (BW) and the adjacent pulmonary artery (PA) in healthy cats and cats with HARD in the cranial and caudal lung lobes.

A total of 12 cats seropositive to specific IgG against *D. immitis* and with clinical signs compatible with HARD (group A) and 7 healthy and seronegative cats (group B) were included. The same CT scanner was used to evaluate the diameter of the bronchial wall and the adjacent pulmonary artery of the right cranial lobe, cranial and caudal aspect of the left cranial lobe, right caudal lobe, and left caudal lobe, following protocols established in previous studies. Statistical study was carried out using SPSS Version 25.0 software, through the Mann–Whitney U method.

The BW/PA ratio was significantly higher for all lung lobes in seropositive cats (0.37 ± 0.04) compared to seronegative cats (0.20 ± 0.01) (p<0.000). The right cranial lobe showed an average of 0.40 ± 0.15 in cats with HARD, being 0.21 ± 0.03 in healthy cats. Similarly, the cranial and caudal aspects of the left cranial lobe exhibited ratios of 0.42 ± 0.11 and 0.41 ± 0.06 in cats from group A, respectively, compared to 0.23 ± 0.03 and 0.20 ± 0.05 in group B. In the right caudal lobe, an average of 0.30 ± 0.08 was observed in seropositive felines, contrasting with 0.17 ± 0.03 in healthy cats. Finally, the left caudal lobe obtained an average of 0.31 ± 0.08 in group A and 0.18 ± 0.02 in group B.

Previous research compared the relationship between BW and PA in healthy dogs and dogs with chronic bronchitis using CT, revealing a higher ratio in the latter. Likewise, cats infected by immature forms of *D. immitis* have chronic bronchial lesions and restrictive lung disease. The results of this study showed that cats with HARD may have a larger bronchial wall than healthy cats, contributing to the development of respiratory signs. These results display the usefulness of CT to detect bronchiectasis in cats with *D. immitis*; nonetheless, more studies with a larger sample number are required.

**Disclosures**

**Source of Funding**

**ECVIM CSF Funding**

No.

**ESVC**—**European Society of Veterinary Cardiology**

## ESVC‐P‐12

## Determination of plasmatic dimethylarginines in dogs with pulmonary hypertension

### C. Valente^1^, C. Guglielmini^1^, G. Romito^2^, C. Mazzoldi^2^, C. Artusi^3^, B. Contiero^1^, H. Poser^1^

#### ^1^University of Padova Animal Medicine, Production and Health, Legnaro, Italy; ^2^University of Bologna Veterinary Medical Sciences, Ozzano dell'Emilia, Italy; ^3^University‐Hospital of Padova Laboratory Medicine, Padova, Italy

Pulmonary hypertension (PH) is a condition characterized by endothelial dysfunction and increased pulmonary artery pressure. Endothelial dysfunction is linked to a decrease production of nitric oxide (NO), which is derived from L‐arginine. Dimethylarginines, byproducts of protein metabolism resulting from L‐arginine methylation, play a role in endothelial dysfunction. Among them, asymmetric dimethylarginine (ADMA) and symmetric dimethylarginine (SDMA) act as endogenous NO synthase inhibitor and compete with L‐arginine cellular uptake, respectively.

The aim of this study was to evaluate the plasma concentration of ADMA, SDMA, their precursor L‐arginine, and L‐arginine/ADMA in dogs with pre‐capillary and post‐capillary PH.

We conducted a retrospective search of clinical records of dogs visited at two Veterinary Teaching Hospitals, selecting those animals with echocardiographic diagnosis of PH. Each dog underwent complete clinical examination, thoracic radiographs, echocardiography, CBC, biochemical profile, and NT‐proBNP quantification. A diagnosis of PH was considered when tricuspid regurgitation velocity was > 3.4 m/s. Additionally, a control group of clinically healthy dogs was included. Plasma concentration of L‐arginine, ADMA and SDMA were determined using high–performance liquid chromatography from plasma samples stored at ‐80°C.

Statistical differences among healthy dogs, dogs with pre‐capillary PH or those with post‐capillary PH were analyzed using a Kruskal–Wallis’ test. The association between dimethylarginines and laboratory and echocardiographic parameters was assessed using the Spearman's rank correlation index. A P value <0.05 was considered statistically significant.

The study included forty‐three dogs, comprising 14 control dogs and 29 dogs with PH, among which 16 had pre‐capillary PH and 13 had post‐capillary PH (all with myxomatous mitral valve disease). The median plasma concentration of SDMA was significantly higher in dogs with post‐capillary PH (0.53 μmol/L [0.48–0.86]) compared to healthy dogs (0.43 μmol/L [0.37–0.47]) (*P* = 0.04). However, no differences among groups of dogs with PH were detected for L‐arginine, ADMA and L‐arginine/ADMA (*P* = 0.23, *P* = 0.11, *P* = 0.12, respectively). Furthermore, SDMA and ADMA showed positive correlation with NT‐proBNP (*r* = 0.64; *P* < 0.0001; *r* = 0.41; *P* = 0.02; respectively), while a negative correlation was found for L‐arginine/ADMA (*r* = ‐0.45; *P* = 0.01).

These preliminary results suggest that SDMA levels might be influenced by the presence of PH. However, further studies involving a larger number of dogs are necessary to confirm this hypothesis.

**Disclosures**

**Source of Funding**

This work was supported by a grant of University of Padua, Italy, to Dr. Poser (SID Year: 2017‐ Protocol number: BIRD173881).

**ECVIM CSF Funding**

No.

**ESVC**—**European Society of Veterinary Cardiology**

## ESVC‐P‐14

## Circadian variation in ventricular rate in dogs with atrial fibrillation

### M.I. Oliveira^1^, G. Culshaw^1^, S.A. Dickson^1^, R. Blake^1^, Y. Martinez Pereira^1^

#### ^1^University of Edinburgh, Edinburgh, United Kingdom

In people, disruption of cardiovascular circadian rhythm is used for stratification of cardiovascular risk. Circadian variation (CV) of ventricular rate (VR) in ambulatory ECG (aECG) has retrospectively been demonstrated in healthy pet dogs and dogs with atrial fibrillation (AF), defined by sinusoidal variation of heart rate with a defined wave amplitude and mesor. In dogs with AF, the mean VR recorded from aECG has prognostic value. This study prospectively investigates presence and features of CV in aECG of dogs with AF. It is hypothesised that in presence of CV, the values of mesor and amplitude will differ from those in healthy dogs and correlate well with mean/median VR.

Eighteen dogs with AF undergoing aECG were prospectively recruited. Cosinor analysis was used to determine presence of CV in aECG recordings, describing values for amplitude and mesor when sinusoidal variation was found. Descriptors of CV were compared with values from healthy dogs. Correlation and Bland–Altman analysis were used to compare mean and median VR with mesor.

Hourly VR varied sinusoidally in 17/18 dogs with AF (mesor 143.8 ± 36.04 bpm, amplitude 11.48 ± 4.76 bpm). Mesor of CV was different in healthy and AF dogs, however the amplitudes did not differ significantly (*p* = <0.0001 mesor; *p* = 0.2224 amplitude). Both median (*r* = 0.76, *p*<0.0001, bias ‐0.7931, SD 7.36) and mean (0.71, *p*<0.0001, ‐0.3078, SD 6.73) had a strong correlation with the mesor.

In this study, CV was documented in nearly all dogs with AF, although significant differences were found in their mesors compared to healthy dogs. In dogs with AF, values of mesor differed from mean/median VR but a strong correlation between them was found. Longitudinal studies should investigate the prognostic value of presence of CV and its parameters in dogs with cardiovascular disease.

**Disclosures**

This abstract is part of a research project with the title ‘Use of heart rate spot‐check protocol to assess 24‐hour average heart rate in canine patients with atrial fibrillation.’ The ECVIM‐CA and Purina Institute Resident Research Awards have awarded us with £8000 to fund this project.

**Source of Funding**

ECVIM‐CA and Purina Institute Resident Research Awards.

**ECVIM CSF Funding**

Yes.

**ESVC**—**European Society of Veterinary Cardiology**

## ESVC‐P‐15

## Assessment of longitudinal systolic function by using tissue motion annular displacement in hypertrophic cardiomyopathy cats

### N. Glaewketgarn^1^, S.D. Surachetpong^1^

#### ^1^Chulalongkorn University Department of Veterinary Medicine, Bangkok, Thailand

Cardiac systolic function is the sum of longitudinal, radial, and circumferential myocardial deformation. In HCM cats, global systolic function assessed by conventional echocardiography is still preserved while longitudinal systolic function assessed by global longitudinal strain (GLS) has already been reduced. In human medicine, tissue motion annular displacement (TMAD) provides information on systolic function by assessing the degree of longitudinal deformation of the mitral annulus toward cardiac apex. TMAD is considered a sensitive and accurate estimation of LV longitudinal systolic function. The aim of this study is to investigate the LV longitudinal systolic function in HCM and healthy cats by using TMAD.

The study population includes 26 healthy cats and 21 HCM cats. According to ACVIM staging guideline, HCM cats were divided into B1, B2 and C subgroups. All cats underwent echocardiographic examinations without sedation, which included conventional echocardiographic parameters, GLS and TMAD. The result of TMAD was presented as global TMAD in millimeters and percentage.

Global TMAD were not influence by breed, sex, age, and heart rate. Global TMAD (mm and %) and GLS significantly decreased in HCM stage C (2.42 mm, 10.49% and −14.4) when compared to control group (3.95 mm, 16.12% and −19.43) (*p*‐value < 0.001, <0.001 and 0.043), suggesting LV longitudinal systolic dysfunction in HCM C. LV fractional shortening (LVFS) showed no difference between the control (52.76%) and HCM stage C group (54.64%) (*p*‐value = 0.972). TMAD was reduced with preserved LVFS due to a compensatory effect from circumferential systolic function and the greater susceptibility of longitudinal myocardial fibers. Global TMAD (mm and %) had a significant correlation with GLS (*r* = ‐0.573, CI −0.739 to 0.342; *r* = ‐0.75, CI = −0.853 to ‐0.590). Because TMAD and GLS are using the same technique, and both represent longitudinal myocardial displacement. Therefore, TMAD can be used to evaluate longitudinal systolic function in HCM. Global TMAD (mm and %) and GLS had moderate correlation with LV posterior wall thickness (*r* = ‐0.554, CI = −0.727 to −0.314; *r* = ‐0.631, CI = −0.779 to‐4.18; GLS *r* = 0.562, CI = 0.325–0.733) indicating the more progressive the disease, the more impaired longitudinal systolic function. In conclusion, TMAD was significantly decreased in cats with HCM stage C compared with the control group and was considered as sensitive technique for assessing LV longitudinal systolic dysfunction in cats with HCM.

**Disclosures**

**Source of Funding**

**ECVIM CSF Funding**

No.

**ESVC**—**European Society of Veterinary Cardiology**

## ESVC‐P‐17

## Echocardiographic prediction intervals of the aortic and pulmonary valve annulus diameter in 1429 dogs: Population‐wide and selected breed‐specific values

### T. Vezzosi^1^, A. Della Pina^1^, G. Grosso^1^, R. Tognetti^1^, H. Broch^2^, O. Domenech^3^

#### ^1^Department of Veterinary Siences, University of Pisa Department of Veterinary Sciences, Pisa, Italy; ^2^Anicura Clinica Veterinaria CMV Varese, Varese, Italy; ^3^Anicura Istituto Veterinario Novara, Novara, Italy

Two‐dimensional echocardiographic evaluation of the diameter of the aortic and pulmonary valve annulus (AV_a_ and PV_a_) plays a key role in the screening and assessment of different congenital heart disease in dogs and it is also critical for decision making when planning interventional or surgical correction of congenital defects. In addition, the AV_a_ is echocardiographically used for indexing different cardiac dimensions to an unchanging structure such as the aorta, but also for hemodynamic calculations. However, no studies described the body weight‐prediction intervals (BW‐PIs) of the AV_a_ and PV_a_ in a large sample of healthy dogs. Therefore, the aim of the present study was to describe BW‐PIs of AV_a_ and PV_a_ according to allometric scales in dogs, and also to describe relative reference intervals (RI) in specific canine breeds.

This retrospective, observational, multicenter study included a total of 1429 healthy dogs. The AV_a_ and PV_a_ were acquired from the right parasternal long‐axis view optimized for the left ventricular outflow tract and the right parasternal short‐axis view optimized for the right ventricular outflow tract, respectively. Body weight‐PIs of AV_a_ and PV_a_ were generated using allometric scales. In addition, breed‐specific RIs for those breeds represented by 70 individuals at least were defined. Finally, a multiple regression analysis was performed to verify the possible influence of age, sex and heart rate on AV_a_ and PV_a_.

Strong positive linear relationships between AV_a_, PV_a_ and BW were evident after logarithmic transformation (R^2^=0.843 and R^2^=0.853, respectively; *P*<0.0001). According to allometric scales, the 95% PI of the AV_a_ normalized to BW [AV_a__N=AV_a_(mm)/BW(kg)^0.325^] was between 4.99 and 7.62, whilst the 95% PI of the PV_a_ normalized to BW [PV_a__N=PV_a_(mm)/BW(kg)^0.326^] was between 4.98 and 7.50. Among specific breeds, AV_a_ and PV_a_ RIs were described among Labradors, Golden Retrievers, Dobermanns, French Bulldogs, English Bulldogs and Boxers. To multivariable analysis, AV_a_ and PV_a_ were independent of age, sex and heart rate.

Generally applicable BW‐dependent PIs and breed‐specific RIs were generated for AV_a_ and PV_a_ in a large sample of dogs, made available for clinical use, especially useful for the echocardiographic assessment of valvular diseases in the general canine population but also in specific breeds predisposed to congenital heart defect.

**Disclosures**

**Source of Funding**

**ECVIM CSF Funding**

No.

**ESVC**—**European Society of Veterinary Cardiology**

## ESVC‐P‐18

## Mid‐systolic depolarization time determined by vectorcardiography as a potential new pre‐screening tool for preclinical dilated cardiomyopathy in Doberman Pinschers

### M. Gheeraert^1^, P. Smets^2^, G. Mampaey^2^, G. Wess^3^, P. Gheeraert^4^, J. Eberhard^3^, J. De Pooter^4^, L. Duchateau^2^

#### ^1^University of Ghent University of Ghent, Merelbeke, Belgium; ^2^University of Ghent, Merelbeke, Belgium; ^3^LMU Munich, Munich, Germany; ^4^University of Ghent, Ghent, Belgium

Early diagnosis of preclinical dilated cardiomyopathy (DCM) remains challenging in primary veterinary medicine due to the need for echocardiography and 24‐h Holter electrocardiogram (ECG) recording. A cost‐effective and readily available diagnostic tool to identify dogs at high risk of preclinical DCM could optimize current screening practice. Vectorcardiographic (VCG) methods have not been investigated for this purpose. We hypothesize that mid‐systolic depolarization time determined by a new VCG method is a sensitive parameter to identify preclinical DCM and correlates with left ventricular size & function in Doberman Pinschers. In this prospective cross‐sectional study, 129 Doberman Pinschers (106 healthy controls and 23 with preclinical DCM) were included. All dogs underwent echocardiography, three‐minute six or 12‐lead ECG and VCG. Diagnostic accuracy of mid‐systolic depolarization time for detection of preclinical DCM was determined using ROC‐analysis. Its correlation to echocardiographic left ventricular size and function was assessed by calculating Spearman's correlation coefficient. A mid‐systolic depolarization time of ≥33.5 ms has a sensitivity of 94.4% and specificity of 78.9% for the detection of preclinical DCM (AUC 0.922, SD 0.028). Furthermore, mid‐systolic depolarization time is strongly correlated with normalized systolic left ventricular diameter (*r*_s_ = 0.77, *p* < 0.001), systolic left ventricular two‐dimensional volume (*r*_s_ = 0.70, *p* < 0.001), and moderately inversely correlated with ejection fraction (*r*_s_ = −0.60). In conclusion, mid‐systolic depolarization time is a sensitive parameter to detect preclinical DCM. Further investigation of its diagnostic potential compared to or in combination with other tools in a primary care veterinary setting is warranted.

**Disclosures**

**Source of Funding**

**ECVIM CSF Funding**

No.

**ESVC**—**European Society of Veterinary Cardiology**

## ESVC‐P‐19

## Echocardiographic reference intervals of the two‐ and three‐dimensional size of the right atrium: A prospective study in 195 healthy dogs

### G. Grosso^1^, G. Menciotti^2^, T. Vezzosi^1^, R. Tognetti^1^, M. Borgarelli^2^

#### ^1^Department of Veterinary Sciences, University of Pisa, Pisa, Italy; ^2^Department of Cardiology, Virginia‐Maryland College of Veterinary Medicine, Blacksburg, United States

Right atrial (RA) size has important diagnostic and prognostic roles for the non‐invasive assessment of right heart diseases and left heart diseases with right‐sided involvement. Body weight (BW)‐prediction intervals (PI; BW‐PIs) of the RA diameter (RAD) and the RA area (RAA) were described in relatively small samples of dogs; furthermore, BW‐PIs of the RA volume (RAV) in dogs are lacking. Therefore, the aim of this study was to describe BW‐PIs of the RAD, RAA and two‐ and three‐dimensional (2D and 3D) RAV in a large sample of dogs.

This prospective, single‐centre and observational study enrolled a total of 195 healthy dogs. The RAD was measured from the right parasternal long‐axis (RPLA) view and the left apical four‐chamber (LA4C) view optimized for the right heart. The RAA and the 2D‐RAV were measured from the LA4C optimized for the right heart, the latter using both the Simpson method of discs (SMOD) and the area‐length (A‐L) method. The 3D‐RAV was obtained from the same view. After investigating relationship to body sizes, the BW‐PIs of RAD, RAA and RAV were generated using allometric scaling. In addition, the reference intervals (RIs) of the RAD indexed to the aortic annulus (RAD_RPLA_/Ao and RAD_LA4C_/Ao, respectively), the RAA indexed to the body surface area (RAAi), and 2D‐RAV_SMOD_, 2D‐RAV_A‐L_ and 3D‐RAV indexed to BW (2D‐RAVi_SMOD_, 2D‐RAVi_A‐L_ and 3D‐RAVi) were defined. Finally, intra and interobserver measurement variability were determined using the coefficient of variation.

Strong positive linear relationships between RA variables and BW were evident after logarithmic transformation (*P*<0.0001) suggesting an allometric relationship. The 95%PI of RAD_RPLA_ normalized for BW [RAD_RPLA__N=RAD_RPLA_(mm)/BW(kg)^0.429^] was between 5.12 and 9.04, the 95%PI of RAD_LA4C_ normalized for BW [RAD_LA4C__N=RAD_LA4C_(mm)/BW(kg)^0.406^] was between 5.29 and 8.79, the 95%PI of RAA normalized for BW [RAA_N=RAA(cm^2^)/BW(kg)^0.832^] was between 0.26 and 0.58, the 95%PI of 2D‐RAV_SMOD_ and 2D‐RAV_A‐L_ normalized for BW [2D‐RAV_SMOD__N and 2D‐RAV_A‐L__N, respectively=RAV(ml)/BW(kg)^1.265^] was between 0.09 and 0.32, whilst the 95%PI of 3D‐RAV normalized for BW [3D‐RAV_N=3D‐RAV(ml)/BW(kg)^1.199^] was between 0.18 and 0.56. Reference intervals for RAD_RPLA_/Ao, RAD_LA4C_/Ao, RAAi, 2D‐RAVi_SMOD_, 2D‐RAVi_A‐L_ and 3D‐RAVi were described. Finally, all RA variables showed good to excellent intra and interobserver measurement variability.

Applicable BW‐PIs of RA size were described in a large sample of dogs and made available for clinical use. The quantification of RA size, especially with novel technologies, could be useful in the echocardiographic screening and severity assessment of congenital and acquired right and left heart diseases in dogs.

**Disclosures**

**Source of Funding**

**ECVIM CSF Funding**

No.

**ESVC**—**European Society of Veterinary Cardiology**

## ESVC‐P‐20

## Effect of echocardiographic imaging view and imaging mode on variability of left ventricular wall thickness measurements in normal cats and cats with hypertrophic cardiomyopathy

### G. Grosso^1^, K. Schober^2^

#### ^1^Department of Veterinary Sciences, University of Pisa, Pisa, Italy; ^2^College of Veterinary Medicine, The Ohio State University Department of Veterinary Clinical Sciences, Columbus, United States

Echocardiography is the clinical gold standard test for diagnosing hypertrophic cardiomyopathy (HCM) in cats. Left ventricular (LV) wall thickness can be measured by two‐dimensional (2D) and M‐mode techniques and from short‐axis (Sax) and long‐axis (Lax) images, but results may differ. Current ACVIM guidelines on feline cardiomyopathies have not recommended a preferred imaging mode and view. The aim of this study was to evaluate agreement between different imaging views and modes in the assessment of LV wall thickness in cats. We hypothesized that there is clinically

relevant bias between methods, and that results cannot be used interchangeably.

Echocardiographic images of 280 cats (140 healthy and 140 with HCM) from the clinical database were reviewed in this retrospective, single‐centre, observational study. End‐diastolic thickness of the interventricular septum (IVS) and LV free wall (LVFW) was evaluated by 2D (Method [M] 1 and M3) and M‐mode (M2 and M4) and from right parasternal (RP) Sax and Lax imaging views ([M1, from a RPLax view using 2D; M2, from a RPLax view using M‐mode; M3, from a RPSax view using 2D, and M4, from a RPSax view using M‐mode) by a single investigator. Using 2D images the thickest portion

of the wall in both M1 and M3 was measured. M‐mode measurements were done according to accepted guidelines. Methods were compared using RM ANOVA on ranks and the Bland‐Altman method with bias and 95% limits of

agreement (95%LOA).

In control cats, measurements of the IVS were not different amongst the 4 methods (*P* = .67) with bias and 95%LOA between 0.04 to 0.10 mm and −1.09 to 1.28 mm, respectively. In contrast, LVFW thickness was higher (*P*<.001) using Mmode

compared to 2D, and bias and 95%LOA were 0.22 to 0.30 mm and −0.81 to 1.23 mm, respectively. In HCM cats, measurements of both IVS and LVFW were different (*P*<.05) amongst methods. Comparing M1 versus M2 and M3 versus M4 for IVS, bias was ‐1.04 to ‐1.13 mm with 95%LOAs between −3.04 and 0.77 mm, respectively. Comparing M1 versus M2 and

M3 versus M4 for LVFW, bias was 0.04 to 0.14 mm with 95%LOAs between ‐1.75 and 2.03 mm, respectively. Observer measurement variability was good (coefficients of variation <10%). Echocardiographic assessment of LV wall thickness with 2D and M‐mode or Lax and Sax views cannot be used interchangeably, particularly in cats with HCM.

**Disclosures**

**Source of Funding**

**ECVIM CSF Funding**

No.

**ESVC**—**European Society of Veterinary Cardiology**

## ESVC‐P‐21

## Validation of a four‐chamber oblique right parasternal view in the echocardiographic evaluation of transmitral flow and mitral regurgitation in dogs with cardiac pathology

### L.F. Álamo Fernández^1^

#### ^1^Vet‐ECODiagnóstico, Molina de Segura‐Murcia, Spain

The recommendations for the echocardiographic exam of the Echocardiography Committee of the Cardiology Specialty of the ACVIM are still widely used. Transmitral flow and mitral regurgitation are classically evaluated from the left apical four‐chamber view, but complete bilateral transthoracic echocardiographic evaluation is not possible in some patients. We questioned whether the assessment of transmitral flow and mitral regurgitation was reliable from an optimized right paraesternal view in the dog.

Complete echocardiographic exam using a Mindray M9 Vet was performed in 85 dogs, 48 males and 37 females (Mean age 10.9 years, range 1‐17. Weight 8.6 kg, range 2‐41), with mitral cardiac pathology or mitral repercussion (73 dogs with mitral degeneration, 7 dilated cardiomyopathy, 2 subaortic stenosis and 3 ductus arteriosus). A four‐chamber oblique right parasternal view was added. It was obtained from a classic four‐chamber right parasternal long axis image, moving the probe caudally and towards the sternum, obtaining an oblique image of the left atrium and ventricle. This image was optimized, with slight movements, to be as longitudinal as possible. The results of maximun velocity and duration of the transmitral E and A waves, E wave deceleration time, diastolic pattern/type of flow, velocity and size (area in left atrium) of the regurgitation jet and dP/dt were compared between this view and the classic left apical four chamber view (Wilcoxon test).

For the right oblique view, median and interquartile range were 68.50/18.16 and 66.50/14.56 cm/s (maximun velocity of E and A), 130/22.61 and 89/18.53 msec (duration), 101/28.16 msec (E deceleration time), 5.39/0.80 m/s (regurgitation vel) and 1693/781.33 mmHg (dP/dt). For the left apical view, results were 68.50/22.42 and 62/17.23 cm/s, 128.5/23.9 and 84/22.23 msec, 101/17.79 msec, 5.30/0 .66 m/s and 1693/781.33 mmHg/s, respectively. Regarding transmitral flow, 13 dogs were excluded (6 dogs presented marked changes in heart rate associated with stress, 4 supraventricular arrhythmias and 3 second‐degree atrioventricular blocks). No statistically significant differences (α=0.001) were detected between the variables for each view. Flow type classification was the same in 66 dogs (92%). In 6 dogs, the difference was between normal flow and abnormal relaxation diastolic pattern. All dogs were used in mitral regurgitation and there were no significant differences (*α*=0.001). The jet size classification was the same.

The evaluation of the variables under study from this right oblique view is reliable, but the classification between normal and abnormal relaxation transmitral flow can generate confusion in a low percentage of dogs.

**Disclosures**

**Source of Funding**

**ECVIM CSF Funding**

No.

**ESVC**—**European Society of Veterinary Cardiology**

## ESVC‐P‐22

## Evaluation of plasma N‐terminal pro‐B‐type natriuretic peptide and urinary aldosterone‐to‐creatinine ratio in healthy Chihuahuas

### G. Dossi^1^, A. Galizzi^1^, C. Locatelli^1^, V. Borromeo^1^, P. Pocar^1^

#### ^1^University of Milan Department of Veterinary Medicine and Animal Science, Milan, Italy

Chihuahua represents an increasingly widespread breed predisposed to cardiac disease. N‐terminal pro‐B‐type natriuretic peptide (NT‐proBNP) might be a useful point of care biomarker for dogs suspected of heart diseases but breed differences have been reported. Urinary aldosterone‐to‐creatinine ratio (UAldo:C) appeared to be a good indicator of the renin‐angiotensin‐aldosterone system activity in dogs, but Chihuahuas showed significantly higher UAldo:C compared to other breeds. The objective of this study was to assess preliminary breed‐specific reference intervals for NT‐proBNP and UAldo:C in healthy Chihuahuas and evaluate sex differences in these parameters.

Chihuahuas were recruited among private‐owned dogs referred for cardiological screening. Dogs had to be healthy and >12 months. The following procedures were performed: collection of medical history (including diet data), blood pressure measurement, physical examination, echocardiography, complete blood count, biochemistry profile, urinalysis (urine was collected by spontaneous micturition), plasma NT‐proBNP and urinary aldosterone assessment.

Forty‐three healthy Chihuahuas were enrolled. Twenty‐six were intact females (anoestrus), 14 were intact males and 3 were neutered males. The median age and body weight were 2.32 (1.19–4.11) years and 2.71 (2.39–3.10) kg respectively. Median NT‐proBNP was 347 (125–515) pmol/L and the calculated reference interval was 125 (90% CI 125–125)–2121 (90% CI 942–2248) pmol/L. Ninety‐one percent (39/43) of Chihuahuas had an NT‐proBNP below the currently available non‐breed specific cut‐off (900 pmol/L). The median UAldo:C was 2.59 (1.57–4.61) μg/g and the calculated reference interval was 0.6 (90% CI 0.5–0.9)–16.8 (90% CI 10.9–27.4) μg/g. No sex differences in NT‐proBNP (*P* = 0.408) and UAldo:C (*P* = 0.124) were found.

Median value and reference interval of NT‐proBNP were in line with other small‐size breeds. The proposed non‐breed specific cut‐off of 900 pmol/L seems to be valid for most healthy Chihuahuas. In contrast to previous studies, no sex differences in NT‐proBNP were detected. As previously suggested, Chihuahuas seem to be characterized by higher values of UAldo:C compared to other breeds.

**Disclosures**

**Source of Funding**

This study was supported by Piano di Sostegno alla Ricerca 2020, Linea 2, University of Milan, Italy.

**ECVIM CSF Funding**

No.

**ESVC**—**European Society of Veterinary Cardiology**

## ESVC‐P‐23

## Dose‐exposure‐response of CARDALIS® (benazepril/spironolactone) on biomarkers of the classical and alternative arms of the renin‐angiotensin‐aldosterone system in healthy dogs

### E. Manson^1^, J. Ward^1^, M. Merodio^1^, E. Guillot^2^, T. Blondel^3^, O. Domenig^4^, J. Mochel^5^

#### ^1^Iowa State University Department of Veterinary Clinical Sciences, Ames, Iowa, United States; ^2^Ceva Sante Animale Companion Animal Franchise, Libourne, France; ^3^Ceva Sante Animale Pharma Research & Development, Libourne, France; ^4^Attoquant Diagnostics, Viena, Austria; ^5^College of Veterinary Medicine, The University of Georgia Department of Veterinary Pathology, Athens, GA, United States

Previous studies have demonstrated a dose‐dependent effect of the angiotensin‐converting enzyme (ACE) inhibitor benazepril on biomarkers of circulating renin‐angiotensin‐aldosterone system (RAAS). A combination product including benazepril and the mineralocorticoid‐receptor antagonist spironolactone (CARDALIS®) is approved for use in dogs with congestive heart failure at a fixed dose of benazepril hydrochloride 0.25 mg/kg and spironolactone 2 mg/kg every 24 hours. The objective of this study was to characterize the dose‐exposure‐response relationship of three different doses of CARDALIS® on biomarkers of RAAS in dogs.

In this study, 18 purpose‐bred healthy beagle dogs were randomly assigned to 3 dosage groups (n = 6 dogs each). After induction of RAAS activation by feeding a low‐sodium diet, dogs received CARDALIS® at one of 3 dosages for 14 days: (1) benazepril 0.25mg/kg + spironolactone 2 mg/kg PO q24hr (labeled dose); (2) benazepril 0.25mg/kg + spironolactone 2 mg/kg PO q12hr (labeled dose given twice daily); and (3) benazepril 0.5mg/kg + spironolactone 4 mg/kg PO q12hr (2 x labeled dose given twice daily). Blood samples were collected at baseline and serial time intervals after CARDALIS® dosing to measure serum RAAS biomarkers and plasma concentrations of active metabolites of benazepril and spironolactone. Noncompartmental pharmacokinetic analyses were performed for each drug metabolite. Time‐weighted averages for serum RAAS biomarkers for 24 hours after CARDALIS® dosing at steady state were compared between dose groups using Wilcoxon rank‐sum testing.

Compared to the lowest (labeled) dose of CARDALIS®, the highest dose (0.5 mg/kg benazepril and 4 mg/kg spironolactone PO q12hr) was associated with significant differences in all RAAS biomarkers, including decreased concentration of angiotensin II (*P* = 0.026), increased concentration of angiotensin 1–7 (*P* = 0.026), decreased surrogate activity of ACE (*P* = 0.0022), and higher concentration of circulating aldosterone (*P* = 0.015). Compared to the intermediate dose of CARDALIS® (0.25 mg/kg benazepril and 2 mg/kg spironolactone PO q12hr), the highest dose was associated with higher markers of renin (*P* = 0.041) and angiotensin I (*P* = 0.026) and decreased surrogate activity of ACE (*P* = 0.0022). Pharmacokinetic data revealed dose‐proportionality for all metabolites. CARDALIS® was well‐tolerated at all doses with no clinically relevant bloodwork changes.

These findings suggest a dose‐dependency of alterations to RAAS metabolites reflecting both ACE inhibition and mineralocorticoid receptor antagonism from the combined CARDALIS® product. This dose‐dependent alteration in classical and alternative RAAS pathways could help inform clinical trials and dosing decisions for dogs with heart disease.

**Disclosures**

Funding for this study was provided by Ceva Sante Animale. Authors EG and TB are employees of Ceva Sante Animale. Authors JLW and JPM have served as consultants for Ceva Sante Animale and have received reimbursement and honoraria for consulting, expert testimony, travel, and service as key opinion leaders (KOLs). Author OD is an employee of Attoquant Diagnostics. Author EM has no relevant competing interests.

**Source of Funding**

Funding for this study was provided by Ceva Sante Animale.

**ECVIM CSF Funding**

No.

**ESVC**—**European Society of Veterinary Cardiology**

## ESVC‐P‐24

## Content of circulating extracellular vesicles as biomarker of oxidative stress in dogs with preclinical myxomatous mitral valve disease

### L. Alibrandi^1^, T. Vezzosi^2^, F. Scebba^1^, R. Tognetti^2^, V. Lionetti^1^

#### ^1^Sant'Anna School of Advanced Studies Unit of Translational Critical Care Medicine, Institute of Health Science, Pisa, Italy; ^2^University of Pisa Department of Veterinary Science, Pisa, Italy

Extracellular vesicles (EVs) are a heterogeneous group of large cell‐derived membrane lipid vesicles that act as signal carriers for cell‐cell communication. In cardiovascular disease, oxidative stress can damage mitochondria causing cellular senescence and disease progression, with an increase in circulating EVs and mitochondria‐containing EVs (mt‐EVs). Thus, circulating EVs are attracting for their potential role as biomarkers of oxidative stress in cardiovascular medicine. Recently, veterinary studies evaluated changes in EVs storage in various diseases, such as leishmaniasis, neoplasms or doxorubicin cardiotoxicity in dogs. However, studies on circulating EVs in small animal cardiovascular diseases are lacking. Thus, the present study aimed to preliminary evaluate changes in the number of circulating EVs, as well as their lipofuscin content and the percentage of circulating mt‐EVs in dogs with preclinical MMVD. This was a prospective observational study enrolling client‐owned healthy dogs and dogs with preclinical MMVD. Extracellular vesicles were isolated from plasma via serial centrifugations. The presence of mitochondria inside the EVs was evaluated using electron and confocal microscopy. We determined the percentage of plasma mt‐EVs using MitoBrilliant™fluorescent probe and we measured lipofuscin content of mt‐EVs by assessing red autofluorescence (PC5.5A) with flow cytometry. The study included 15 dogs, of which 5 healthy dogs (stage ACVIM A—control group), 5 stage ACVIM B1 dogs and 5 stage ACVIM B2 dogs. The median number of plasma EVs was not different among the groups (*P* = 0.19). The median percentage of plasma mt‐EVs was lower in preclinical dogs [B1, 55.8%, interquartile range (IQR) 36.6‐59.5%); B2, 44.7%, IQR 13.4–59.0%] in comparison to stage A (79.3%, IQR 77.2–90.6%) (*P* = 0.035 and *P* = 0.041, respectively). Moreover, dogs in stage B2 showed a higher lipofuscin content (median signal intensity 6528, IQR 6293–7350 signal intensity) in comparison to stage A (median signal intensity 2101, IQR 155‐2884 signal intensity) (*P* = 0.014). To our preliminary results, dogs with preclinical MMVD have enhanced oxidative stress levels compared to healthy dogs predisposed to MMVD in terms of lower percentage of circulating mt‐EVs and higher lipofuscin content in stage B2. Thus, mitochondria and lipofuscin carried by circulating EVs seems to be a novel potential biomarker of oxidative stress in canine preclinical MMVD.

**Disclosures**

**Source of Funding**

**ECVIM CSF Funding**

No.

**ESVC**—**European Society of Veterinary Cardiology**

## ESVC‐P‐25

## Left heart dimensions and cardiac troponin I concentrations in dogs presenting with diarrhoea

### L. Hardy^1,2^, I. Spalla^3^, J. Munday^4^, J. Seo^5,6^

#### ^1^Bristol Veterinary Specialists Internal Medicine., Bristol, United Kingdom; ^2^Animal Referral Centre Internal Medicine., Auckland, New Zealand; ^3^San Francesco Veterinary Hospital Cardiology, Milan, Italy; ^4^School of Veterinary Science, Massey University Veterinary Pathobiology and Research Director, Palmerston North, New Zealand; ^5^Animal Referral Centre Cardiology, Auckland, New Zealand; ^6^School of Veterinary Science, Massey University Cardiology, Palmerston North, New Zealand

Diarrhoea is a common clinical sign of underlying gastrointestinal disease. Studies in dogs and humans suggest compromised gastrointestinal health can negatively affect cardiovascular function by an altered autonomic tone, hypovolaemia, acid‐base disturbances, electrolyte abnormalities, and circulating inflammatory cytokines and gut microbial metabolites. The aim of this study was to describe the left heart dimensions and circulating cardiac troponin I (cTnI) concentration in dogs presenting for evaluation of diarrhoea. We hypothesised that dogs presenting with diarrhoea have increased cTnI concentration and that the increased cTnI concentration is associated with left ventricular systolic dysfunction.

This prospective observational dual‐centre study included 55 client‐owned dogs which were presented for evaluation of diarrhoea. Exclusion criteria included previous diagnosis of cardiac disease, current treatment with corticosteroids, diuretics, cardiac drugs, and the use of intravenous fluids within 24 hours of presentation. Each dog underwent a complete physical examination, haematology, biochemistry and blood pressure measurement. A focused cardiac ultrasound was performed by trained observers. Serum cTnI measurement was performed using a validated assay (Siemens ADVIA Centaur Tn‐I Ultra assay, Siemens Healthcare Diagnostics Inc, Germany), which had a reference value of <0.06 ng/mL.

Eleven of 55 dogs (20%) with diarrhoea had an increased cTnI concentration compared to the reference interval (mean cTnI 0.05 ng/mL [±0.78]). Of these dogs, 5 were diagnosed with acute haemorrhagic diarrhoea syndrome, 2 had a chronic enteropathy, 2 had acute idiopathic diarrhoea, and 1 had anaphylaxis. No cause of the diarrhea was determined in 1 dog. On focused cardiac ultrasound, 11 of the 55 dogs (20%) had echocardiographic signs of hypovolaemia, 5 (9%) had reduced left ventricular systolic function, and 9 (16%) had hyperdynamic systolic function. The dog with diarrhoea of unknown aetiology had increased cTnI (0.1 ng/mL) and evidence of hypovolaemia on focused cardiac ultrasound and subsequently died suddenly of a cardiopulmonary arrest during stabilisation. No association was identified between left heart dimensions and cTnI concentration within the 55 dogs in this study. CTnI concentration was not associated with baseline heart rate, blood lactate, Purina faecal score, blood pressure measurements, or cause of diarrhoea.

In this cohort of dogs presenting to emergency centres with diarrhoea, 20% had increased cTnI concentration. Echocardiographic evidence of hypovolaemia and hyperdynamic state were common. Five dogs (9%) had left ventricular systolic dysfunction but no association with cTnI concentration was identified. Clinical significance of the increased cTnI and left ventricular systolic dysfunction in dogs presenting with diarrhoea warrants further studies.

**Disclosures**

**Source of Funding**

Healthy Pets New Zealand IDEXX assisted with the shipping of samples and subsidised the cardiac troponin I testing.

**ECVIM CSF Funding**

No.

**SCH**—**Society of Comparative Hepatology**

## SCH‐P‐1

## Percutaneous endovascular embolization for treatment of single extrahepatic portosystemic shunt in four dogs

### L. Di Filippo^1^, C. Olmedo^2^, R. Santiago^3^

#### ^1^Hospital Veterinari del Mar IVC Evidensia Internal Medicine, Barcelona, Spain; ^2^Hospital Veterinari del Mar IVC Evidensia Surgery, Barcelona, Spain; ^3^Hospital Veterinari de Molins IVC Evidensia Internal Medicine, Barcelona, Spain

Percutaneous endovascular therapy (PET) has been employed in dogs for the occlusion of intrahepatic portosystemic shunts since the 2000s, with lower morbidity and mortality compared to traditional surgery. There is limited information regarding PET for extrahepatic portosystemic shunts (EPSS). The aim of this case series was to describe the procedure and outcome of PET in four dogs with a single EPSS. Four privately owned dogs were included. All dogs were diagnosed with EPSS by compatible clinical signs, abnormal dynamic bile acid test, and computed tomography (CT). The median age was 3 years [2–5 years], two males and two females. The median body weight was 8.1 kg [5–11 kg]. The main clinical signs were associated with hepatic encephalopathy in 2 dogs, polyuria/polydipsia in 1 dog, and gastrointestinal signs in 2 dogs. Prior to surgical treatment, all patients were stabilized with medical treatment for three weeks with high‐quality plant‐based protein diet and lactulose. CT findings were consistent with portocaval shunt (*n* = 1), left gastrophrenic shunt (*n* = 1), left gastrocaval shunt (*n* = 1) and splenoazygos shunt (*n* = 1). Direct measurement of portal blood pressure was performed with a multipurpose catheter (10.1 mmHg [8–11.5 mmHg]). The increase in portal pressure was evaluated simulating a temporary occlusion through Fogarty ballon inflation and was within the acceptable range to complete the shunt closure (16.5 mmHg [14–18.0 mmHg]). Based on preoperative CT angiography and intraoperative contrast examination, vascular plugs were selected for three dogs (Amplatzer Vascular Plug II Abbott; Cera Vascular Plug) and a cover stent (Advanta V12 Getinge) was selected for one dog. Under fluoroscopy, the device was deployed into the shunting vessel (*n* = 3) or vena cava (*n* = 1). Selective angiography was performed to confirm the closure of the malformation. In all cases complete occlusion of blood flow was possible with a single device. There were no major procedure‐related complications. No dogs developed post‐ligation signs of portal hypertension. The median time of hospitalization was 1.5 days [1–3 days]. In the long‐term follow‐up (day 30‐90‐120), clinical signs resolution was achieved in all patients. Fasting and postprandial bile acid were in reference range (median 2,9 μmol/L and 4,9 μmol/L; reference range 0–29.9 μmol/L) in all patients, approximately one month after surgery. PET was clinically effective in the treatment of single EPSS in dogs. Preoperative CT and intraoperative contrast examination is essential for device selection based on shunt morphology. PET is a promising alternative to conventional surgical procedures for single EPSS in dogs.

**Disclosures**

**Source of Funding**

**ECVIM CSF Funding**

No.

**SCH**—**Society of Comparative Hepatology**

## SCH‐P‐2

## Retrospective study of 15 cats with hepatic lipidosis: Clinicopathological findings

### N. Mateu^1^, S. Atencia^2^

#### ^1^AniCura Vetsia Veterinary Hospital Internal Medicine, Madrid, Spain; ^2^AniCura VETSIA Veterinary Hospital Internal Medicine, Madrid, Spain

Feline hepatic lipidosis (FHL) is the most common hepatobiliary disease in the cat. It is characterised by the accumulation of triglycerides in more than 80% of hepatocytes, resulting in impaired liver function as a consequence of a negative energy balance.

The aim of this retrospective study was to describe the most relevant clinicopathological findings in a population of 15 cats diagnosed with FHL in a referral hospital in Spain.

A total of 15 cats with liver cytology consistent with FHL were included. Haematology, biochemistry, feline pancreatic specific lipase (fPLI), coagulation times and cobalamin results were reviewed. Additionally, ultrasound, cytology and bile culture results were evaluated.

Hyperbilirubinaemia was the most common clinicopathological finding (100%), followed by a mild to moderate increase in alkaline phosphatase (ALKP) (13/15) and alanine aminotransferase (ALT) (13/15). In one case, the increase in ALT was much more severe (>x10) than ALKP. In this patient liver cytology revealed a lymphoma.

Only in 13% (2/15) the fPLI was consistent with pancreatitis.

Cobalamin was measured in 8 cases. One cat showed hypocobalaminemia (287 ng/L). In the remainder, values were within the range (423–762 ng/L) (63%), or severely increased (>1200 ng/L) (25%). Among the two patients whose cobalamin levels were out of range, one was diagnosed with multiple myeloma.

Clotting times were found to be more than 25% above the upper limit in 60% (6/10) of the cats in which they were measured. These patients were treated with vitamin K and underwent plasma transfusion after which clotting times normalised and no bleeding was observed following hepatic fine needle aspiration.

The most common ultrasound finding was an increased liver echogenicity 93% (14/15).

Liver cytology was consistent with FHL in all cases and in two of the patients it was used to diagnose lymphoma. Bactibilia was observed in only 3/15, however, 100% of the bile cultures were negative. Two out of the 3 patients with bactibilia had previously received antibiotics, which could explain the absence of bacterial growth in the culture.

Following the diagnosis of FHL, an oesophagostomy tube was placed to initiate early feeding. The average time before patients started eating willingly was two and a half weeks.

The most frequent cause for the development of FHL was exposure to stressful situations (33%).

Regarding prognosis, 75% of the patients were discharged, so, despite being a life‐threatening disease, the prognosis improves if an early diagnosis and appropriate treatment is established.

**Disclosures**

**Source of Funding**

**ECVIM CSF Funding**

No.

**SCH**—**Society of Comparative Hepatology**

## SCH‐P‐3

## A retrospective, multicentre study investigating the use of C‐reactive protein in the diagnosis and monitoring of canine bacterial cholecystitis

### S. Espenica^1^, M. Rodgers^2^, M. Targett^3^, W. Bayton^1^

#### ^1^Dick White Referrals Internal Medicine, Cambridgeshire, United Kingdom; ^2^University of Cambridge Pathology, Cambridge, United Kingdom; ^3^University of Nottingham Neurology (Lecturer), Nottingham, United Kingdom

Evaluate if C‐reactive protein (CRP) has diagnostic significance in canine bacterial cholecystitis, to aid clinicians in diagnosis and appropriate treatment. Determine if CRP has prognostic significance in canine bacterial cholecystitis.

This was a retrospective case‐control study based in two UK referral centres. Signalment, history, clinicopathological findings, ultrasonographic findings, bile cytology and microbiological results, treatments and outcomes of dogs, between January 2016 and March 2023, were reviewed.

A total of 94 dogs met the inclusion criteria; this included 25 case dogs with confirmed bacterial cholecystitis on bile cytology or culture. Twelve dogs with a suspected bacterial cholangiohepatopathy that were negative on culture but improved with antibiotic treatment were excluded from statistical analysis. The most reported history findings were vomiting (68%), lethargy (60%), painful abdomen (40%) and anorexia (36%). A patient presenting with a painful abdomen was the only statistically significant finding between groups (T‐test, *P* = 0.018). The clinicopathological variables with significant differences between the case and control group included: neutrophil count (13 × 10^9^/L; T‐test, *P* = 0.001), ALP‐activity (Median 1581 U/L; T‐test, *P* = 0.002), haematocrit (Median 42%; T‐test, *P* = 0.029), AST‐activity (Median 71 U/L; T‐test, *P* = 0.035), cholesterol (Median 10 mmol/L; T‐test, *P* = 0.03). In the present study CRP was significant in diagnosis of bacterial cholecystitis (Median 31 mg/L; T‐test, *P* ≤ 0.001). The most common isolate cultured was *Escherichia coli* (62%) and 4/29 isolates were defined as MDR organisms. The median length of hospitalisation was 3 days and all patients survived until discharge. CRP had a weak positive correlation with length of hospitalisation; a spearman rank correlation 0.212 (*P* = 0.05).

CRP, alongside expected clinicopathological abnormalities, was statistically significant in the diagnosis of bacterial cholecystitis. If patients presented with suspected hepatobiliary disease, using an ALP/AST ratio cut‐off value of >10, gave a 61% sensitivity (95% CI, 39%–80%) and 94% specificity (95% CI, 82%–99%) for presence of bacterial cholecystitis. CRP at admission weakly correlated with duration of hospitalisation. In total 14% of cultured isolates were MDR organisms, and this should highlight the need for appropriate antibiotic prescribing.

**Disclosures**

**Source of Funding**

**ECVIM CSF Funding**

No.

**SCH**—**Society of Comparative Hepatology**

## SCH‐P‐4

## Retrospective study of 80 dogs with congenital portosystemic shunts

### P. Corominas^1^, P. Gabarda^2^, C. Bertolani^1^, L. Tabar^2^, E. Pujol^3^, J. Tabar^4^

#### ^1^Canis Veterinary Hospital IVC Evidensia Internal Medicine, Palma de Mallorca, Spain; ^2^Anicura San Vicente Veterinary Hospital Internal Medicine, San Vicente del Raspeig, Alicante, Spain; ^3^Canis Veterinary Hospital IVC Evidensia Soft Tissue Surgery and Traumathology, Palma de Mallorca, Spain; ^4^Anicura San Vicente Veterinary Hospital Soft Tissue Surgery and Traumathology, San Vicente del Raspeig, Alicante, Spain

The evidence base for the treatment of congenital portosystemic shunt (CPSS) is weak, with some studies pointing to a higher survival of surgically treated dogs over medically treated dogs. Moreover, there is no clear superiority of one surgical technique over others.

The aim of the present study was to retrospectively review clinical records from dogs diagnosed with CPSS from two referral Hospitals.

80 dogs were evaluated, including 40 males and 40 females, from 4 months to 7 years (10 dogs aged ≥5 years and 70 <5 years) and 21 different breeds. Main clinical signs were digestive (55%), neurological (65%) and urinary (35%). Analytical alterations included hypoalbuminemia (38/80), anemia (26/80), abnormal bile acids (78/78), hypoglycemia (23/80), elevated liver enzymes (50/80), hypocholesterolemia (23/43) and hyperammonemia (12/20). Diagnosis was confirmed by mesenteric portography (6), computed tomography (71) and exploratory laparotomy (3), with 11 intrahepatic CPSS and 69 extrahepatic CPSS. Medical and surgical treatment was performed in 14 and 66 dogs, respectively. Ameroid constrictor was used in 45 dogs and cellophane band in 21 dogs.

Follow‐up was available for a period of more than 3 months in 48.8% of dogs, more than 6 months in 46.3% and more than 1 year in 41.3%. The median follow‐up time was 664 days (1–4745 days).

Perioperative survival was 93.3% when ameroid technique was used and 90.5% for cellophane band, with no statistical difference between them. All the dogs that died did it in the perioperative period (5/6), except for one dog that died one month after surgery. Recurrence rate of clinical signs was 21.6% for surgically treated dogs (15% for ameroid and 40% for cellophane, with no statistical difference between them). The 8 cases with clinical recurrence did it in the first 6 months after surgery and all presented with neurological signs at recurrence.

The information about outcome in medically treated dogs was limited.

Type of clinical signs, analytical abnormalities and age at surgical time (less or more than 5 years) were not associated with survival.

The findings from this study agree with the previous literature. Survival after surgical treatment of CPSS is high, regardless of the technique employed. In fact, if a dog survives the perioperative period, the probability of dying due to the disease is low, reflecting the exit of surgical closure of the shunt. Moreover, age at diagnosis should not influence treatment choice.

**Disclosures**

**Source of Funding**

**ECVIM CSF Funding**

No.

**SCH**—**Society of Comparative Hepatology**

## SCH‐P‐5

## Differences in signalment and biochemical markers of hepatic function between dogs with intrahepatic and extrahepatic congenital portosystemic shunts and investigation of post‐attenuation seizures development

### F. Busato^1^, A. Zoia^1^

#### ^1^San Marco Veterinary Clinic Internal Medicine, Veggiano, Italy

Congenital portosystemic shunts (cPSSs) can be intrahepatic or extrahepatic and the latter recognize different morphologies. The two aims of this study were to identify, at diagnosis, differences in signalment and markers of hepatic function in dogs with diverse type/morphology of cPSS and to identify predisposing factors for development of post‐attenuation seizures (PAS).

Cross‐sectional study (first aim) and retrospective cohort study (second aim) including dogs with cPSSs presented between 2019 and 2023. Variables studied were: age, sex, body weight (BW), body condition score (BCS), breed, shunt type/morphology, episodes of hepatic encephalopathy (HE) before surgery, and markers of hepatic function (i.e., albumin, cholesterol, glucose, urea, bilirubin, fibrinogen, APTT, PT, and urinary bile acids/creatinine ratio [uBA/crea]). Extrahepatic cPSSs were attenuated through cellophane banding and intrahepatic cPSSs via percutaneous transvenous coil embolization. Post‐attenuation follow‐up for PAS development lasted 3 months.

Seventy‐two dogs entered the study: 42 females and 30 males. Sixteen were Maltese dogs, 9 mongrels, 8 Yorkshire terries, 4 chihuahuas, 4 miniature poodles, 4 shih‐tzu, 3 pugs, 3 border collies, 3 jack russel terriers, and 18 dogs of other 15 different breeds. There were 14 intrahepatic cPSSs and 58 extrahepatic cPSSs (i.e., 31 portocaval, 15 portophrenic, and 12 portoazygos). Episodes of HE before surgery occurred in 43 (60%) dogs.

Intrahepatic shunts were significantly younger (median=6.5 months), at diagnosis, than portophrenic (median=32 months; *P* = 0.029) and portoazygos (median=47.5 months; *P* = 0.004) shunts and were also significantly heavier (median=13.1 kg) than portocaval (median=3.6 kg; *P*<0.001), portophrenic (median=5.0 kg; *P*<0.001) and portoazygos (median=5.2 kg; *P* = 0.008) shunts. Intrahepatic shunts had a significantly lower serum albumin concentration (median=2.2 g/dL) than portophrenic shunts (median=2.7, g/dL; *P* = 0.042); while, portocaval shunts had a significantly higher PT (median=8.7 sec.) than portophrenic shunts (median=7.9 sec.; *P* = 0.035). No differences between cPSSs types/morphology were found for sex, BCS, episodes of HE, and the other markers studied.

Twelve dogs (17%) developed PAS, within 3 days of surgery. PAS– dogs were significantly younger (median=17.5 months) than PAS+ dogs (median=55.5 months; *P* = 0.016). There was also a trend for higher uBA/crea in PAS+ (median=429.65) in comparison to PAS– dogs (median=2021.35; *P* = 0.085). No differences between PAS+ and PAS– dogs were found for sex, BW, BCS, breed, shunt type/morphology, episodes of HE, and the other markers studied.

Signalment and laboratory differences were present between cPSSs type/morphology. As previously demonstrated dogs PAS+ were significantly older than dogs that remained seizure free. None of the other variables analyzed showed a potential role in identify this possible complication.

**Disclosures**

**Source of Funding**

**ECVIM CSF Funding**

No.

**SCH**—**Society of Comparative Hepatology**

## SCH‐P‐6

## Histopathological frequency of feline hepatobiliary diseases in Ireland (2013–2023)

### J.A. Fuentes Rodríguez^1^, C. Broadbridge^1^, P. Kelly^1^, E. O'Neill^1^

#### ^1^School of Veterinary Medicine, University College Dublin, Dublin, Ireland

Hepatobiliary diseases are a common cause of morbidity and mortality in cats, with histopathology an essential step for reaching a diagnosis and guiding appropriate therapy. This retrospective study aimed to review the relative frequency of diseases identified in liver biopsy reports from a university referral hospital over the study period. A secondary aim was to determine the most common clinical signs and clinicopathological abnormalities identified at the time of presentation.

Liver biopsies performed from 2013 to 2023 were identified; those with evidence of hepatobiliary disease included, those unremarkable or non‐diagnostic excluded. Signalment and clinical signs at presentation and duration were recorded, along with haematology and biochemistry. Reported histopathology findings were categorised based on WSAVA Liver Standardization Project guidelines. Where more than one disease category was identified, multiple were selected.

Seventy‐nine histopathology reports were identified. Six did not meet the criteria and were excluded (3 normal, 3 non‐diagnostic). The remaining 73 comprised four post‐mortem, four tru‐cut and sixty‐five exploratory laparotomy samples from 32 females (28 neutered) and 41 males (36 neutered), and a variety of breeds, including 58 domestic shorthair cats. Median age was 7.5 years.

Forty‐two cats had inflammatory liver disease: 16 cholangitis (2 neutrophilic, 2 lymphocytic, and 12 mixed), 19 reactive hepatitis, 3 acute hepatitis, 1 chronic hepatitis and 3 pyogranulomatous hepatitis. The most common disease category was vacuolar hepatopathy (total 22), occurring secondary to other conditions in 14 (cholangitis, congenital portosystemic shunt (CPSS) and reactive hepatitis). Non‐inflammatory diseases included cholestasis (15), neoplasia (9), and CPSS (8). Five cats presented with perfusion‐related disorders, 3 cats had secondary lipogranuloma, and 1 each, reversible hepatocellular injury, cholecystitis and a biliary cyst.

The most common presenting clinical signs were weight loss (34/72) and inappetence (31/72), with vomiting (26/72) and jaundice (14/72) also common. Only 6 cats presented with pyrexia. The median duration of clinical signs was 65 days (data available in 62 cats). Clinical pathology findings (available in 72 cats) showed 19 with leukocytosis and neutrophilia, 28 lymphopenia, 10 monocytosis, and 4 lymphocytosis. 64% cats had hyperbilirubinemia (>5.1 mmol/L) and 41% elevated liver enzyme activit> (>3× upper limit); increased ALT (30/72), ALP (21/72), AST (9/72) and GLDH (14/72).

Vacuolar hepatopathy was the most reported single disease category, however overall inflammatory liver diseases were most common, making up 57% of cases. Almost 50% of cats presented with inappetence and weight loss, 41% of them having significant elevation of at least one liver enzyme activity.

**Disclosures**

**Source of Funding**

**ECVIM CSF Funding**

No.

**ESVNU**—**European Society of Veterinary Nephrology and Urology**

## ESVNU‐P‐1

## Increased concentrations of Kidney Injury Marker‐1 in urine of cats with pyelonephritis and cats with ureteral obstructions

### L. Jessen^1^, L.N. Nielsen^1^, R. Langhorn^1^, A. Jensen^1^, F.S. Moberg^1^, N.H. Dupont^1^, A.S. Gravgaard^1^, N. Bak‐Jakobsen ^1^, I.N. Kieler^1^, B.J. Reezigt^2^, D. Bienzle^3^

#### ^1^University of Copenhagen Department of Veterinary Clinical Sciences, FREDERIKSBERG C, Denmark; ^2^Blue Star Veterinary Hospital, Gothenburg, Sweden; ^3^Ontario Veterinary College, University of Guelph Department of Pathobiology, Guelph, Canada

Pyelonephritis may be associated with tubular damage, in particular if concomitant obstructive uropathy is present. Kidney Injury Marker 1 (KIM‐1) is a scavenger receptor of the proximal tubular epithelial cells and a highly specific marker of acute kidney injury. This study aimed to investigate the degree of tubular injury in cats with pyelonephritis and assess the discriminatory potential of KIM‐1. Sixty‐three urine samples, of which 20 were left‐over‐samples from previous studies, were included in this prospective case‐control study from 2015 to 2024. Cats were categorized as pyelonephritis (*n* = 20, of which 19 were complicated), ureteral obstruction (UO, *n* = 12), chronic kidney disease (CKD, *n* = 9), subclinical bacteriuria (SBU)/cystitis; *n* = 11) or healthy (*n* = 11). Urinary KIM‐1 (UKIM‐1) was measured by lateral flow immunoassay. A positive control was measured with each test sample, and the ratio was normalized to a urine specific gravity of 1,035. Mild to marked UKIM‐1 elevations were detected in 12/20 and 7/12 cats with pyelonephritis and UO, respectively. UKIM‐1 was significantly higher in cats with pyelonephritis (median 0.73, range 0.19–1.26) compared to healthy cats (0.36, range 0.25–0.61, *p* = 0.004) and cats with SBU/cystitis (0.33, range 0.07–0.71, *p* = 0.0002). In cats with UO (0.76, range 0.19–1.07) UKIM‐1 was significantly higher compared to healthy cats (*p* = 0.01) and cats with SBU/cystitis (*p* = 0.01). There was no statistically significant difference between UKIM‐1 in cats with CKD (0.62, range 0.40–0.94) and any other group. This study suggests that tubular injury is common in cats with complicated pyelonephritis and cats with UO. High UKIM‐1 in cats with bacteriuria may prompt suspicion of pyelonephritis.

**Disclosures**

Dr. Bienzle is a co‐inventor of the feline KIM‐1 assay. Dr. Rem Jessen has received speakers honorarium from VIN, ACVIM, ECVIM, the VET GROUP for not‐subject‐matter talks. Dr. Langhorn has received speaker honorarium from several private clinics in Denmark for not‐subject‐matter talks. Dr. Nielsen has financial relationship with Eiken Chemicals Ltd (2016–2018) and several non‐subject matter speaker engagements with medical companies (Elanco and Vet Group). Dr Reezigt has received speakers honorarium for non‐subject‐matter webinars for OrionPharma, Agria, FirstVet.

**Source of Funding**

This study was funded by a grant from the Agria Research Foundation.

**ECVIM CSF Funding**

No.

**ESVNU**—**European Society of Veterinary Nephrology and Urology**

## ESVNU‐P‐2

## Aetiology of proteinuria in dogs without known pre‐renal, renal or post‐renal causes

### E. Ruane^1^, M. Rodgers^1^, C. Hare^1^, K. McCallum^1^, T. Williams^1^

#### ^1^University of Cambridge Veterinary Medicine, Cambridge, United Kingdom

Proteinuria is classified as pathological, encompassing pre‐renal, renal and post‐renal causes, or physiological. Physiological proteinuria is described in both humans and dogs; however, the mechanism is incompletely understood. Anecdotally, we have observed that proteinuria is a common finding in dogs without known causes and we hypothesised that proteinuria would be associated with systemic inflammation. This study aimed to evaluate the aetiology of proteinuria in dogs without known pre‐renal, renal or post‐renal causes and to report its magnitude in these cases.

Dogs with available serum C‐reactive protein (sCRP) concentrations and urine protein: creatinine ratio (UPC) data over a 2‐year period were included. Dogs were excluded if they lacked biochemistry data, or if known causes of proteinuria were identified from their records. Cases were defined as having systemic inflammation based on elevated sCRP concentrations (>8.2 mg/L), then classified by their final diagnosis. Continuous and categorical variables were compared between groups using non‐parametric statistics and associations between disease categories and proteinuria were evaluated using multivariable logistic regression analyses. Data are presented as median [minimum‐maximum].

74 proteinuric and 313 non‐proteinuric dogs were included. The UPC was significantly higher in dogs with systemic inflammation than those with sCRP ≤8.2 mg/L (0.26 [0.05–4.63], *n* = 100 vs. 0.14 [<0.01–7.32], *n* = 287; *P*<0.001). Mild (UPC 0.5–1), moderate (UPC 1‐2) and marked (UPC>2) proteinuria were observed in 21, 6, and 5 dogs with systemic inflammation, and in 19, 8 and 15 dogs without systemic inflammation, respectively. Proteinuria (U>C >0.5) was more prevalent in dogs with pancreatitis (52% vs. 17%; *P*<0.001) and less prevalent in non‐pancreatic gastrointestinal disease (2% vs. 22%; *P*<0.001) compared to the rest of the study population. Pancreatitis remained independently associated with increased odds of proteinuria (odds ratio [OR] = 4.5 [95% CI: 1.8–11.3]; *P*<0.001), and non‐pancreatic gastrointestinal disease remained independently associated with decreased odds of proteinuria ( in multivariable logistic regression analyses.

This study demonstrates a positive association between systemic inflammation and proteinuria in dogs without known pre‐renal, renal or post‐renal causes, suggesting that systemic inflammation should be considered as a differential diagnosis for proteinuria in dogs. Although in most cases proteinuria was mild (UPC <1), moderate to marked proteinuria was frequently observed. This study also found pancreatitis to be an independent risk factor for proteinuria in dogs.

**Disclosures**

**Source of Funding**

**ECVIM CSF Funding**

No.

**ESVNU**—**European Society of Veterinary Nephrology and Urology**

## ESVNU‐P‐3

## Evaluation of the effect of renaltec in cats with chronic kidney disease IRIS stage 2 and 3

### I. Elzenbeck^1^, S. Teichmann‐Knorrn ^2^, K. Hartmann^1^, Y. Zablotski^1^, R. Dorsch^1^, J.S. Suchodolski^3^

#### ^1^LMU Small Animal Clinic, Centre for Clinical Veterinary Medicine, Munich, Germany; ^2^Tierklinik Oberhaching, Oberhaching, Germany; ^3^College of Veterinary Medicine and Biomedical Sciences, Texas A&M University Gastrointestinal Laboratory, Department of Small Animal Clinical Sciences, College Station, United States

Chronic kidney disease (CKD) is associated with accumulation of uremic toxins such as indoxyl sulfate (IS) in blood and tubular epithelial cells which can contribute to progression of CKD.

Aim of this prospective, randomized clinical pilot study was to evaluate effect of oral spherical carbonaceous adsorbent renaltec (PorusOne, Porus) on IS plasma concentration, progression of CKD (increase in IRIS stage or >25% creatinine increase), parameters of calcium/phosphate homeostasis (tCa, phosphorus, FGF23), urine protein/creatinine ratio (UP/C), and fecal dysbiosis index (FDI) in cats with stable CKD IRIS Stage 2 and 3.

Nineteen cats with CKD were randomly assigned to the control group (*n* = 9, 8 stage 2, 1 Stage 3) or study group (*n* = 10, 8 stage 2, 2 Stage 3). All cats received treatment according to IRIS‐Guidelines. Study group cats received in addition 500 mg renaltec PO q24h. Cats were examined on day 0 (t0), after 3 months (t1), and after 6 months (t2). Statistical analysis was performed using generalized linear mixed models.

On t0, study group cats had significantly lower tCa (*p* = 0.026) than control group cats. On t1, there were no differences between groups, while on t2, study group cats had significantly lower IS (*p* = 0.045) and UP/C (*p* = 0.001). Progression of CKD was noted in 2 cats of each group. Study group cats had a significantly lower FDI on t2 compared to t1 (*p* = 0.023) and t0 (*p* = 0.009).

Administration of renaltec over 6 months has a positive influence on parameters that might help to delay progression of CKD.

**Disclosures**

**Source of Funding**

**ECVIM CSF Funding**

No.

**ESVNU**—**European Society of Veterinary Nephrology and Urology**

## ESVNU‐P‐4

## Clinical value of monitoring the urinary amino acid excretional fraction in the diagnosis and management of dogs with androgen‐dependent cystinuria

### P. Bernard^1^, F. Sabourdy^2,3^, R. Lavoué ^1^

#### ^1^National Veterinary School of Toulouse, Toulouse, France; ^2^Unité Mixte de Recherche INSERM 1037, CNRS 5071, Toulouse III Paul Sabatier, Toulouse, France; ^3^Laboratoire de Biochimie, Institut Fédératif de Biologie, CHU Purpan, Toulouse, France

Androgen‐dependent cystinuria is a defect in tubular reabsorption of cystine and dibasic amino acids associated with remission following castration. A low‐purine diet is often recommended in this context but has been associated with severe complications. Accurate diagnosis and follow‐up of this condition might therefore prevent unnecessary prescription of specific diet and its associated risks.

Five cystinuric entire male dogs were prospectively included. Urine and plasma amino‐acid profiles were obtained by liquid chromatography coupled with mass spectrometry at first presentation (M0), then 1 (M1) and 3 (M3) months after castration.

Two Staffordshire Bull Terriers, a Dachshund, a Basenji, and a Swiss Mountain Dog were included. Cystine excretional fraction decreased during follow‐up and normalized in 4 dogs (M0 median: 21,4% [9.9–77.4]; M1 median: 2% [0.8–7.7]; M3 median: 1.6% [1–2,3]). Ornithine, Lysine and Arginine excretional fractions showed similar evolution. No recurrence was observed over a median follow‐up period of 128 days [29–253] despite discontinuation of the low‐purine diet in 4 dogs. During follow‐up, marked amyotrophy (*n* = 3), digestive disorders (*n* = 1), excessive urine alkalinization (*n* = 3) and dilated cardiomyopathy secondary to suspected amino acid deficiencies (*n* = 1) were observed with low‐purine diet.

Monitoring of amino acid profiles might be a useful tool in the diagnosis and management of androgen‐dependent cystinuria and could limit the need to prescribe a low‐purine diet, which can be associated with serious complications in some patients. Low stability of sulphur‐containing amino acid could limit its usefulness and should be further documented in dogs to adjust guidelines recommendation.

**Disclosures**

Residency program of the presenting author partially financed by a compagny (Royal Canin)–non funding received for the current study

**Source of Funding**

**ECVIM CSF Funding**

No.

**ESVNU**—**European Society of Veterinary Nephrology and Urology**

## ESVNU‐P‐5

## Results of renal replacement therapy for acute kidney injury caused by *Babesia canis in dogs*

### J. Bujok^1,2^, J. Rafalska^3^, M. Czechowska^4^

#### ^1^Przychodnia Weterynaryjna Wrocławska Wroclaw University of Environmental and Life Sciences, Bielany Wrocławskie, Poland; ^2^Wroclaw University of Environmental and Life Sciences Wroclaw University of Environmental and Life Sciences, Wrocław, Poland; ^3^Wroclaw University of Environmental and Life Sciences, Wrocław, Poland; ^4^Przychodnia Weterynaryjna Wrocławska, Bielany Wrocławskie, Poland

Babesiosis in dogs is a common disease in Poland caused mainly by *Babesia canis* parasites. This acute disease is characterized by systemic inflammatory response syndrome and may result in multi organ dysfunction syndrome (MODS). Acute kidney injury (AKI) due to babesiosis may be severe necessitating initiation of renal replacement therapy (RRT). Since RRT is a very expensive therapy, it is important to estimate the prognosis for survival and recovery after AKI. The aim of this study was to retrospectively determine the survival of patients treated with RTT for AKI caused by *Babesia canis* in a single RRT center in Poland.

Between 2019 and 2024, 15 dogs (six females and nine males) were treated with intermittent hemodialysis using a Fresenius 4008 machine for AKI secondary to babesiosis. The dogs were between 1 and 11 years old (mean 5.2 ± 3.08 years) and weighed between 10.6 and 60.0 kg (mean 26.7 ± 13.8 kg). Serum creatinine concentration on admission was from 10.0 to 23.0 mg/dl (mean 14.8 ± 3.6 mg/dl), blood urea nitrogen was from 136 to 503 mg/dl (254 ± 87), and a mean hematocrit was 26.3 ± 4.0%. Nine dogs had oliguria or anuria at the presentation, and 7 of them were clinically overhydrated. Six dogs had hypocalcemia. Eight patients had MODS involving two organs and in two dogs three organs were involved.

13 (86.6%) dogs survived to the completion of renal replacement therapy. Surviving dogs required from 2 to 6 hemodialysis sessions (mean 4 ± 1). In eight patients, creatinine concentration remained mildly elevated (up to 2.2 mg/dl) and stable for up to 3 months of observation. Both non‐surviving dogs had long‐standing oligoanuria, a positive test result for *Borrelia burgdorferi*, MODS affecting 2 or more organs, and were older. One dog was euthanized after 13 hemodialysis sessions due to persistent severe azotemia, and the second patient after 5 treatments due to lack of diuresis and poor general condition.

RRT for AKI in the course of babesiosis provides a good prognosis in most patients undergoing intermittent hemodialysis.

**Disclosures**

**Source of Funding**

No funding, retrospective study.

**ECVIM CSF Funding**

No.

**ESVNU**—**European Society of Veterinary Nephrology and Urology**

## ESVNU‐P‐6

## Comparison of the usefulness of cystatin C, creatinine, SDMA assays in the assessment of renal function in small and miniature dog breeds

### J. Rafalska^1^, J. Bujok^2^

#### ^1^University of Environmental and Life Sciences Faculty of Veterinary Medicine, Wrocław, Poland; ^2^University of Environmental and Life Sciences Division of Animal Physiology, Faculty of Veterinary Medicine, Wrocław, Poland

According to IRIS recommendations, creatinine in dogs should be lower than 125 μmol/l. Cystatin C (CysC) and symmetric dimethylarginine (SDMA), which has also been recently added to the guidelines (should be lower than 0,89 μmol/l) can also be used to assess kidney function. Creatinine depends on muscle mass, which is lower in small dogs, therefore a patient with a GFR decrease <75% may still be within the reference range. The aim of the study was to determine and compare available markers of GFR in healthy dogs with a body weight up to 5 kg.

The study included 39 privately owned dogs which had blood analysis done before castration or teeth scaling. The dogs were Yorkshire Terrier (YST), Maltese, Chihuahua, Jack Russel Terrier, Shih Tzu and mixed breeds. The patients were between 4 and 180 months old and weighed between 1.3 kg and 5.3 kg. The average BCS was 3.5/5. Each dog was examined (clinical examination, blood pressure measurement, kidney ultrasound, urine specific gravity, urine protein:creatinine ratio) to rule out any conditions affecting GFR. Crea, SDMA and CysC were marked in the same commercial laboratory from the same serum sample. The laboratory reference ranges were: Crea 35.0–132.0 μmol/l, SDMA 0.000–0.700 μmol/l, CysC 0.300–1.30 mg/l.

The mean Crea, SDMA and CysC in the examined dogs were 63,49 ± 13,09 μmol/l, 0.5 ± 0,13 μmol/l, 0.58 ± 0.12 mg/l respectively. 95% confidence intervals were as follows: 60.9–71.21 μmol/l, 0.46–0.58 μmol/l, and 0.56–0.66 mg/l for Crea, SDMA, and CysC respectively. These results deviate from the laboratory's reference ranges. There was no direct correlation between any of the parameters and the animal's body weight. Creatinine correlated moderately with CysC (*r* = 0,48, *p*<0.05), while no correlation was found between SDMA and other markers. There were also no significant differences in markers between young, adult and senior dogs (<1.5, 1.5–11, >11 years old respectively). Maltese breed (80.64 ± 14,41 μmol/l) had significantly higher creatinine levels compared to YST (56.28 ± 10,66 μmol/l) and Chihuahuas (61.74 ± 11.07 μmol/l), while SDMA levels did not differ between breeds (0,45 ± 0,1, 0.59 ± 0,14 and 0.49 ± 0.11 for YST, Chihuahua and Maltese respectively).

A common creatinine reference range may lead to delayed renal disease diagnosis in small dogs. The ranges should be established based on the weight but also the dog's breed, because as initially found in our study‐ Maltese have naturally higher creatinine levels compared to other miniature breeds. In these breeds, simultaneous assay of SDMA may provide a more accurate assessment of renal function.

**Disclosures**

**Source of Funding**

Young Minds Project funded by Wroclaw University of Environmental and Life Sciences, Poland.

**ECVIM CSF Funding**

No.

**ESVNU**—**European Society of Veterinary Nephrology and Urology**

## ESVNU‐P‐7

## Routine urinalysis predicts the results of urinary bacteriological culture in dogs and cats

### N. Jousserand^1^, C. Trumel^2^, M. Dalla Nave^3^, M. Mantelli^4^, V. Leynaud^3^, B. Reynolds^4^, A. Diquélou^1^, E. Oswald^1^, R. Lavoué ^4^

#### ^1^IRSD, Université de Toulouse, INSERM, INRAE, ENVT, UPS, Toulouse, France; ^2^CREFRE, Université de Toulouse, INSERM, UPS, ENVT, Toulouse, France; ^3^Université de Toulouse, ENVT, Toulouse, France; ^4^InTheRes, Université de Toulouse, ENVT, INRAE, Toulouse, France

Urine bacterial culture is an important part of the diagnostic work‐up of urinary tract diseases. Time lag between urine collection and obtention of bacterial culture might be associated with inadequate management.

The aim of this prospective observational study was to establish the diagnostic performances of routine urinalysis to predict significant bacteriuria. Every dog and cat requiring complete urinalysis during the diagnostic work‐up was included. Negative and positive predictive values (NPV, PPV), sensitivities and specificities of urine dipstick results, direct visualization of bacteria or image of phagocytosis were determined using urine culture as the reference method.

222 dogs (83 positive cultures, 139 negative) and 205 cats (56 positive, 149 negative) were included. Efficient variables in dogs to predict a positive culture (PPV [95% CI], specificity [95% CI]) were nitrites ≥1+ (100% [100–100], 100% [100–100]), leukocytes ≥3+ (92% [91–93], 99% [98–100]), observation of bacteria (93% [90–95], 97% [95–100]), or phagocytosis (93% [91–95], 98% [96–100]); and in cats observation of bacteria (97% [95–98], 99% [98–100]) or phagocytosis (95% [94–96], 99% [98–100]). Efficient variables in dogs and cats to rule‐out a significant bacteriuria (NPV [95% CI], sensitivity [95% CI]) were proteins≥1+ (76% [71–82], 81% [76–86] in dogs; 96% [93–98], 98% [96–100] in cats), observation of bacteria (81% [75–87], 59% [51–66] in dog; 92% [88–96], 75% [68–82] in cats), or phagocytosis (75% [69–82], 41% [34–49] in dogs; 85% [80–91], 48% [40–55] in cats.

Routine urinalysis is useful to confirm or rule‐out significant bacteriuria in dogs and cats and might prevent useless antimicrobial prescription.

**Disclosures**

**Source of Funding**

French ministry for agriculture and food.

**ECVIM CSF Funding**

No.

**ESCG**—**European Society of Comparative Gastroenterology**

## ESCG‐P‐1

## Prospective randomized controlled clinical trial of the long‐term effects of omeprazole in healthy dogs

### L. Gil^1,2^, P.F. Navarro^3^, G. Martín^3^, A. Vila^3,4^, R. Sáiz^3,4^, S. Soler^3,4^

#### ^1^Universidad Católica de Valencia UCV Department of Animal Medicine and Surgery, Valencia, Spain; ^2^Hospital Veterinario UCV Department of Animal Medicine and Surgery, Valencia, Spain; ^3^Universidad Católica de Valencia UCV, Valencia, Spain; ^4^Hospital Veterinario UCV, Valencia, Spain

The use of omeprazole in veterinary medicine has been questioned in recent years. This study aimed to assess the long‐term effects of omeprazole on cobalamin and serum gastrin levels in healthy dogs. Eighteen dogs were included, divided into a control group (*n* = 10) and an omeprazole group (*n* = 8). Blood samples were collected at three‐time points: before treatment (T_0_), 30 days (T_1_), and 60 days (T_2_). The mean cobalamin value (ng/L) in the control group was 481.4 ( ± 293.70) at T_0_, 481.4 ( ± 170.21) at T_1_, and 513.2 ( ± 174.50) at T_2_. In the omeprazole group, the values were 424.62 ( ± 161.57) at T_0_, 454.5 ( ± 160.96) at T_1_, and 414,87 ( ± 127.90) at T_2_. The median gastrin values (pg/mL) in the control group were 62.45 [30.17–218.75] at T0, 76.06 [30.67–199.87] at T1, and 63.02 [35.81–176.06] at T_2_. The median gastrin value in the omeprazole group was 67.59 [55.96–101.60] at T_0_, 191.77 [75.31–1901.77] at T_1_, and 128.16 [43.62–1066.46] at T_2_. No significant changes were observed in cobalamin levels between the two groups throughout the study period. These findings align with previous studies in felines but contrast with human medicine, where a decrease in serum cobalamine has been described as associated with omeprazole long‐term use. However, omeprazole administration significantly increased serum gastrin levels compared to the control group (*p* = 0.008), indicating an effect on gastric acid secretion. Gastrin is a hormone involved in regulating gastric acid production, and its elevation suggests a potential risk of gastric hyperplasia or cancer, as has been described in humans. Adverse effects, such as diarrhea or vomiting, were reported in 18% of dogs in the omeprazole group, potentially associated with gastrointestinal dysbiosis. These findings align with previous research indicating that omeprazole can disrupt the gastrointestinal microbiome, potentially leading to digestive disturbances. While short‐term use of omeprazole is generally well‐tolerated in dogs, caution is warranted regarding its prolonged administration due to the risk of gastrointestinal complications. Further studies are needed to explore the long‐term effects of omeprazole and its implications for canine health.

**Disclosures**

**Source of Funding**

“This research was funded by Universidad Católica de Valencia San Vicente Mártir, grant num‐ber 2019‐205‐001.”

**ECVIM CSF Funding**

No.

**ESCG**—**European Society of Comparative Gastroenterology**

## ESCG‐P‐2

## Haematological parameters and blood cell ratios highly correlate with faecal characteristics in dogs with food‐responsive and Immunosuppressant‐responsive enteropathies (FRE and IRE)

### C. Higueras^1^, A. Sainz^2^, M. García‐Sancho ^2^, F. Rodríguez‐Franco ^2^, D. Díaz‐Regañón^2^, A. Rey^1^

#### ^1^University Complutense de Madrid Department of Animal Production, Madrid, Spain; ^2^University Complutense de Madrid Department of Animal Medicine and Surgery, Madrid, Spain

Chronic enteropathies (CEs) are classified retrospectively based on treatment response. In human medicine, the complete blood count of patients with inflammatory bowel disease (IBD) has been widely studied. Thus, increased platelet count is a widely recognised marker of IBD activity and other blood cell ratios such as neutrophil‐to‐lymphocyte (NLR), monocyte‐to‐lymphocyte (MLR) and platelet‐to‐lymphocyte (PLR) ratios have been shown to have diagnostic and prognostic potential. A limited number of reports exists regarding the haematological aspects of canine CEs. One of the few existing studies in Immunosuppressant‐responsive enteropathy (IRE) dogs observed higher NLR values than in Food‐responsive enteropathy (FRE).

The aim of this study was to evaluate the differences in haemogram and haematological inflammatory indices between dogs with FRE and those with IRE, and to evaluate possible correlations of these parameters with faecal fat and moisture percentages and canine IBD activity index (CIBDAI) as indicators of illness severity.

Forty‐two dogs were included in this prospective study. Twenty‐six dogs were diagnosed with FRE based on the good response to an elimination diet. Sixteen dogs were diagnosed with IRE based on the poor response to diet, the good response to immunosuppressants, and the evidence of an inflammatory process on histologic duodenal samples, after a complete differential diagnosis workup. Biochemistry was performed to exclude systemic diseases that may present similar symptomatology, such as hypoadrenocorticism or exocrine pancreatic insufficiency. Leftover blood samples were used for haemogram. Faecal samples were collected for parasite detection, (such as *Giardia duodenalis*) as well as assessment of fat and moisture content. Finally, endoscopy and biopsy samples were necessary for the final diagnosis of IRE. NLR, MLR and PLR were determined by dividing the absolute neutrophil, monocyte and platelet counts by the absolute lymphocyte count, respectively.

IRE dogs presented higher platelet count (*p* = 0.0020), plateletcrit (PCT; *p* = 0.0204) and PLR (*p* = 0.0138) compared to FRE dogs. Faecal fat correlated positively with platelets (*r* = 0.648, *p* = 0), PCT (*r* = 0.340, *p* = 0.0315), NLR (*r* = 0.496, *p* = 0.0021) and MLR (*r* = 0.504, *p* = 0.0024) respectively. Moisture percentage correlated positively with platelets (*r* = 0.468, *p* = 0.0069) and PCT (*r* = 0.393, *p* = 0.0195). Monocytes count also correlated positively with CIBDAI (*r* = 0.418, *p* = 0.008).

This study suggests that platelet count and blood cell ratios such as PLR, NLR and MLR could be clinically useful in the evaluation of dogs with CEs. Furthermore, they may serve as predictive indices of disease severity, as indicated by their correlation with worsened faecal characteristics.

**Disclosures**

**Source of Funding**

**ECVIM CSF Funding**

No.

**ESCG**—**European Society of Comparative Gastroenterology**

## ESCG‐P‐3

## Evaluation of three serum proteomic biomarkers in chronic enteropathy in dogs

### J. Yu^1^, V. Wasinger^2^, P. Bennett^3^, C. Ruaux^1^

#### ^1^Sydney School of Veterinary Science, University of Sydney, Sydney, Australia; ^2^Bioanalytical Mass Spectrometry Facility, University of New South Wales, Sydney, Australia; ^3^University of Melbourne, Melbourne, Australia

Chronic enteropathy (CE) is common in dogs and can be classified based on response to treatment as food‐responsive enteropathy (FRE), antibiotic‐responsive enteropathy and idiopathic inflammatory bowel disease (IBD). Dogs that do not respond to immunosuppressive therapies are classified as non‐responsive enteropathy (NRE). The aim of this study is to further characterise serum proteomic biomarkers that differentiate between dogs with FRE and IBD or NRE.

A cross‐sectional multicentre study was performed. Dogs with clinical signs of chronic enteropathy, such as vomiting, diarrhoea, weight loss or inappetence for at least three weeks were recruited. Clinicopathological diagnostic tests, faecal tests and diagnostic imaging were performed to rule out extra‐gastrointestinal diseases and infectious causes. All dogs had at least one diet trial with a hydrolysed protein or a novel protein diet. Five dogs diagnosed with FRE and nine dogs presumptively diagnosed with IBD or NRE were enrolled. Serum samples were collected and analysed using mass spectrometry‐based proteomic techniques. Protein abundances were analysed using a logistic regression model.

The median age was 5 years (range 1–10 years). There were three intact males, seven neutered males and four neutered females. Body condition scores ranged from 3 to 6 out of 9, with a median score of 5. CCECAI scores ranged from 3 to 16, with a median score of 7. Histopathological diagnoses were available in nine dogs. Significant increases in gelsolin (*p* < 0.001), transferrin receptor protein 1 (TRP1) (*p* = 0.001) and fibronectin (*p* < 0.001) were identified in the IBD/ NRE group compared to FRE dogs. Receiver operator curves (ROC) were calculated for discrimination between dogs with FRE and IBD/ NRE using these proteins, with gelsolin showing an AUC of 1.00, specificity and sensitivity of 100% at an optimal cut off relative abundance of 14. Similarly, for TRP1 and fibronectin, the ROC curves had AUC of 0.92 and 0.96 respectively, with optimum cut‐off relative abundances of 8.5 and 26, specificities of 100% and 80%, and sensitivities of 89% and 100%, respectively. The combined used of these three proteins differentiates between dogs with FRE and IBD/ NRE.

These data support the potential use of gelsolin, TRP1 and fibronectin as biomarkers to differentiate between dogs with FRE and IBD/ NRE. We are currently recruiting more cases to further validate the utility of these three proteins.

**Disclosures**

**Source of Funding**

This project is funded by ESCG‐Purina Institute Trainee Research Grant by ESCG and Purina institute.

**ECVIM CSF Funding**

No.

**ESCG**—**European Society of Comparative Gastroenterology**

## ESCG‐P‐4

## Prevalence of hypocobalaminemia and hypercobalaminemia in a referral population of cats in the United Kingdom and their disease associations

### S. Borgonovi^1^, W. Bayton^1^

#### ^1^DWR Veterinary Specialists, Six Mile Bottom, United Kingdom

Cobalamin (vitamin B12) is a water‐soluble vitamin that plays an essential role in many metabolic intracellular reactions. The prevalence of hypocobalaminemia in cats was reported to occur in up to 43% of cats with chronic enteropathy and up to 78% of cats with alimentary lymphoma. Hypercobalaminemia has been previously associated with liver disease or solid neoplasms.

The aim of the study was to assess the prevalence of hypocobalaminemia (B12 <400 pg/ml) and hypercobalaminemia (B12 >1000 pg/ml); to describe the clinicopathological abnormalities and the diagnostic imaging findings in a referral population of cats in the UK and to identify the underlying disease processes associated with both conditions.

Two hundred sixteen cats were included between December 2016 and December 2023 in this retrospective single centre study. 76 cats (35.18%) had hypocobalaminemia, 67 cats (31.01%) had hypercobalaminemia and the remaining 73 cats (33.79%) had normal cobalamin.

In the hypocobalaminemic group the most common diagnosis were chronic enteropathy in 31/76 cats (40.79%), high grade lymphoma in 14/76 (18.42%) and small cell alimentary lymphoma in 8/76 (10.53%). In the hypercobalaminemic group the most common diagnosis were chronic enteropathy in 38/67 cats (56.72%), high grade lymphoma in 11/67 (16.42%), chronic kidney disease in 10/67 (14.93%) and pancreatitis in 7/67 (10.45%).

The most common clinical signs were chronic vomiting in 36/76 (47.37%) hypocobalaminemic cats and in 24/67 (35.82%) hypercobalaminemic cats (*p* = 0.005); hyporexia in 40/76 (52.63%) hypocobalaminemic cats and in 21/67 (31.34%) hypercobalaminemic cats (*p*<0.001) and chronic diarrhoea in 12/76 (15.79%) hypocobalaminemic cats and in 21/67 (31.34%) hypercobalaminemic cats (*p* = 0.001).

The most common abnormalities identified on abdominal ultrasound were lymphadenomegaly in 49/76 (64.47%) hypocobalaminemic cats and 28/67 (41.79%) hypercobalaminemic cats (*p* = 0.0025) and thickened intestines in 49/76 (64.47%) hypocobalaminemic cats and in 28/67 (41.79%) hypercobalaminemic cats (*p* = 0.025).

Median survival time was 274 days in the hypocobalaminemic group and 711 days in the hypercobalaminemic group (*p* = 0.001). The hypocobalaminemic cats had an increased risk of early death compared to normocobalaminemic cats (odds ratio 2.4 vs. 0.4 for hypercobalaminemic cats) (*p*<0.001).

This study suggests that chronic vomiting is seen in nearly half of hypocobalaminemic cats; chronic diarrhoea and chronic enteropathy are more common in hypercobalaminemic cats in contrast with previous literature. None of the hypercobalaminemic cats in our study had a diagnosis of liver neoplasia and <3% were diagnosed with hepatopathy. Hypocobalaminemia is associated with reduced survival in this cohort of cats, therefore early cobalamin supplementation is recommended.

**Disclosures**

**Source of Funding**

No funding required.

**ECVIM CSF Funding**

No.

**ESCG**—**European Society of Comparative Gastroenterology**

## ESCG‐P‐5

## Influence of breed on the composition of the fecal microbiota in healthy dogs

### J. Hernandez^1^, A. Jablaoui^2^, T. Méric^3^, V. Mariaule^2^, H. Mkaoua^2^, N. Akermi^2^, T. Maufras^3^, A. Drut^3^, O. Sénécat^3^, M. Lomet^3^, E. Maguin^2^, M. Rhimi^2^

#### ^1^Oniris VetAgroBio Nantes Clinical Sciences, Nantes, France; ^2^INRAE, Jouy‐en‐Josas, France; ^3^Oniris VetAgroBio Nantes, Nantes, France

The composition of the intestinal microbiota is influenced by many factors such as diet, physical activity, age, environment, medications, etc. The role of genetics is not completely understood. In humans, some genes involved in olfaction, metabolism, or immunity seem to influence the composition of the intestinal microbiota. The objective of this study is to compare the intestinal composition in the fecal samples of healthy Yorkshire terriers (YT), Australian shepherds (AS) and German shepherds (GS).

Samples were selected from a fecal biobank of 400 healthy dogs. Breeds were matched in terms of age, sex, body condition score and diet (kibble with identical macronutrient analysis). After extraction of the metagenomic DNA, the V3‐V4 regions were amplified with designed primer pairs of the 16S ribosomal RNA locus. To analyze the raw sequences, we used the bioinformatics pipeline FROGS (Find Rapidly OTU with Galaxy Solution). Statistical analyses were carried out using the R software (R Core Team, 2019; R Foundation for Statistical Computing, Vienna, Austria). The Mann–Whitney test was applied to analyze the differences in phyla, families, and genera between the three groups. Benjamini–Hochberg corrections (BH) were used to avoid false positives (significance threshold = 0.05).

Twenty‐eight YT, 15 GS and 11 AS were included in the study. Firmicutes, Bacteroidetes and Fusobacteria were the most abundant phyla in all breeds without significant differences between them. The richness index (Observed) and the alpha‐diversity indices (Shannon and Simpson) were significantly reduced in YT compared to the other two breeds. Beta‐diversity was significantly different in YT compared to the other two breeds. At the family level, Bacteroidaceae and Campylobacteraceae were significantly more represented, while Christensenellaceae, Enterobacteriaceae, Erysipelotrichaceae, Enterococcaceae, Peptococcaceae, and Peptostreptococcaceae were significantly less represented in YT compared to the other two breeds. At the genus level, 10 genera were differentially abundant, including *Bacteroides* and *Faecalibacterium*, which were overrepresented, and *Butyricicoccus*, *Peptococcus* and *Ruminococcus* which were under‐represented in the YT compared to the other 2 breeds.

This study suggests that differences in fecal microbiota composition might be related to breed, particularly in Yorkshire terriers compared to German shepherds and Australian shepherds.

**Disclosures**

**Source of Funding**

Financial support of Agria & Société Centrale Canine (France).

**ECVIM CSF Funding**

No.

**ESCG**—**European Society of Comparative Gastroenterology**

## ESCG‐P‐6

## Value of routine faecal analysis and antibiotic usage in dogs with diarrhoea in general practice in the Southwest of England: A retrospective study

### H. Stringfellow^1^, L. Fortuna^2^, I. Schofield^3^, G. Ruiz^4^

#### ^1^Bristol Vet Specialists, Bristol, United Kingdom; ^2^Bristol Vet Specialists Resident of Internal Medicine, Bristol, United Kingdom; ^3^CVS Director of Clinical Research, London, United Kingdom; ^4^Bristol Vet Specialists Head of Internal Medicine, Bristol, United Kingdom

The objectives of this study were to describe the treatments prescribed to dogs presented for diarrhoea to first opinion practices, their associated outcome, and to evaluate the benefits of performing faecal analysis in these animals.

This was a retrospective study of dogs presented for diarrhoea to 12 first opinion practices in Southwest England between 2019 and 2021 which had faecal analysis performed. Signalment, presenting clinical signs, initial treatments and responses, faecal analysis results, secondary treatments and responses, and further analyses and outcomes, when available, were recorded and assessed. The benefit and relevance of the use of antimicrobials were also reviewed. Univariable and multivariable binary logistic regression modelling were used to assess associations between demographic and management risk factors and a resolution of diarrhoea.

The study included 403 dogs, 45% were less than 1 year old. Reasons for presentation included acute diarrhoea (63%), intermittent diarrhoea (20%), and chronic diarrhoea (16%); 1% presented with haematochezia alone. Initial treatments included diet change (91%), probiotics ±associated with prebiotics (81%), wormer (29%), and antibiotics (17%). After initial treatment, full resolution occurred in 53%, 14% had a partial response and 34% did not respond. Most common pathogens identified on faecal analysis were *Campylobacter spp*. (28%) and *Giardia spp*. (15%); *Salmonella spp*. was identified in 5 cases. In 59% of the dogs, no bacteria or parasite could be identified. In multivariable analysis, dogs with non‐haemorrhagic diarrhoea (*p* = 0.017), and those with chronic diarrhoea at presentation (*p* < 0.001), were less likely to resolve despite treatments. Dogs for which *Campylobacter spp*. (odds ratio = 0.37; 95% confidence interval 0.22–0.61) or *Giardia spp*. (odds ratio = 0.14; 95% confidence interval 0.07–0.27) were identified were also less likely to have resolution of diarrhoea. There was no impact of the diet change or use of antibiotic on the likelihood of resolution of the diarrhoea following multivariable analysis.

This study suggests that antibiotics are fairly frequently prescribed to dogs with diarrhoea in first opinion practice (approx. 20%), although it does not appear that their use increases the likelihood of diarrhoea resolution.

**Disclosures**

**Source of Funding**

No funding.

**ECVIM CSF Funding**

No.

**ESCG**—**European Society of Comparative Gastroenterology**

## ESCG‐P‐7

## Assessing the reproducibility of shotgun metagenomic sequencing and quantitative PCR assays for evaluating the fecal microbiota in dogs

### C.H. Sung^1^, M.P. Hong^1^, P. Giaretta^1^, R. Pilla^1^, J. Suchodolski^1^

#### ^1^Gastrointestinal Laboratory, Texas A&M University, College Station, United States

Changes in intestinal microbiota have been linked to chronic enteropathy in dogs and cats. Next‐generation sequencing (NGS) is commonly utilized to describe the intestinal microbiota in clinical studies. However, studies on inter‐assay variation to evaluate the reproducibility of NGS methods are limited. This study aimed to assess the reproducibility of targeted quantitative PCR (qPCR) assays and untargeted DNA shotgun sequencing in evaluating the fecal abundances of the core microbiota, previously identified by shotgun metagenomic sequencing in clinically healthy dogs.

The study analyzed eight fecal samples from dogs. To evaluate inter‐assay variation, fecal DNA was extracted at the same time, divided into multiple aliquots, and then submitted for shallow metagenomic shotgun sequencing on four separate occasions using the same sequencing platform (Illumina NovaSeq 6000), alongside qPCR assays performed eight times on separate days. Downstream analysis of sequencing data was performed using identical bioinformatic pipelines, including the same reference database and rarefaction depth. The percentage of coefficient of variation (CV%) for the abundance of the core bacterial taxa and alpha diversity metrics across multiple batches was calculated.

The median% (range) CVs of the relative abundances of the core microbiota varied for shotgun metagenomic sequencing, such as *Bacteroides* (120%, 67%–200%), *Bifidobacterium* (27%, 12%–72%), *Blautia* (45%, 14%–136%), *Faecalibacterium* (99%, 65%–200%), *Prevotella copri* (26%, 10%–53%), *Ruminococcus gnavus* (38%, 7%–100%), *Streptococcus* (79%, 73%–115%), and *Turicibacter* (83%, 26%–106%). The median CVs for alpha diversity (observed features, Chao1, and Shannon) were 25%, 23%, and 11%, respectively. For the qPCR assays, the median CVs of the absolute abundances of *Bacteroides, Bifidobacterium, Blautia, Clostridium hiranonis, E. coli, Faecalibacterium, Streptococcus*, and *Turicibacter* ranged from 0.8% to 5.3%. *Fusobacterium* was not detected in 72.8% (23/32) samples by shallow shotgun metagenomic sequencing, whereas qPCR assay detected *Fusobacterium* in 100% of samples analyzed.

Targeted qPCR assays showed acceptable inter‐assay variation. In contrast, despite using the same sequencing platform, a high CV% across batches indicated low reproducibility in assessing the relative abundances of the core microbiota through DNA shotgun sequencing. This emphasizes the need for caution when interpreting sequencing data across different batches or studies, as distinguishing between alterations in bacterial abundance due to treatment effects versus technical variability may be difficult.

**Disclosures**

All authors are employers by the Gastrointestinal Laboratory at Texas A&M University, which provides assays for intestinal function and microbiota analysis on a fee‐for‐service basis.

**Source of Funding**

Microbiome Research at the GI Laboratory is in part supported through the Purina PetCare Research Excellence Fund.

**ECVIM CSF Funding**

No.

314

**ESCG**—**European Society of Comparative Gastroenterology**

## ESCG‐P‐8

## Associations between ultrasonographic imaging of the gallbladder and pathogenic bacteria isolation in twenty‐two dogs with extrahepatic biliary disease

### A.R. Codea^1^, M. Niculae^2^, D. Neagu^3^, C.P. Popovici^3^, A. Biris^3^, S.P. Codorean^3^, M. Mircean^3^

#### ^1^University of Agricultural Sciences and Veterinary Medicine Internal Medicine, Cluj‐Napoca, Romania; ^2^University of Agricultural Sciences and Veterinary Medicine, Faculty of Veterina Infectious Diseases, Cluj‐Napoca, Romania; ^3^University of Agricultural Sciences and Veterinary Medicine, Faculty of Veterina Internal Medicine, Cluj‐Napoca, Romania

Bacterial involvement in canine extrahepatic biliary pathologies is documented by a relatively small number of studies. Gallbladder ultrasound examination combined with bile culture and in *vitro* sensitivity tests facilitate accurate diagnosis and treatment.

This study aimed to identify the most common bacterial species isolated from bile samples and to assess their association with the most frequent gallbladder pathological ultrasonographic findings in dogs.

Twenty‐two client‐owned dogs, 12 males and 10 females of various breeds and age groups (mean 10.8 years), were included in this retrospective case series study. Dogs were selected based on their medical history, clinical signs, abdominal ultrasonography, and laboratory findings suggestive of gallbladder disease. All dogs underwent a complete abdominal ultrasound examination, and 18 dogs underwent ultrasound‐guided cholecystocentesis under sedation and/or general anesthesia. Four dogs were diagnosed with biliary mucocele during an abdominal ultrasound examination with an absolute contraindication for cholecystocentesis. Bile samples from these patients were collected postoperatively from the gallbladder. Aerobic and anaerobic bacterial bile culture tests were performed on the collected samples.

Abdominal ultrasound examination revealed the following diagnostic findings: cholecystitis in 10 dogs (45.45%), cholelithiasis in 8 dogs (36.36%), and biliary mucocoele in 4 dogs (18.18%).

Out of the 22 bile samples evaluated, 17 (77%) samples tested microbiologically positive, while 5 (23%) samples yielded negative results. Positive cultures yielded a single bacterial isolate in 82% of cases, and bacterial coinfection was confirmed in 14% of cases. *Escherichia coli* (39%) and *Staphylococcus spp*. (28%) were the most commonly identified bacteria. The remaining positive isolates included *Enterococcus spp*. (16%) and *Streptococcus spp*. (17%).

From the cholelithiasis group, a total of 6 (75%) bile samples were microbiologically positive for: *Escherichia coli n* = 2 (33.33%), *Streptococcus spp. n* = 3 (50%), and *Enterococcus spp*. n = 1 (16.6%). From the cholecystitis group, a total of 8 (80%) bile samples were microbiologically positive for: *Enterococcus spp*. *n* = 2 (25%), *Escherichia coli n* = 1 (12.5%), *Staphylococcus spp*. n = 2 (25%), *Streptococcus spp. n* = 1 (12.5%), *Escherichia coli* and *Staphylococcus spp*. coinfection n = 1 (12.5%), and Escherichia coli and Staphylococcus spp. coinfection *n* = 1 (12.5%). From the biliary mucocoele group, all 4 (100%) samples were microbiologically positive for *Escherichia coli* n = 2 (50%) and *Staphylococcus spp*. *n* = 2 (50%).

This study highlights the significant involvement of bacterial pathogens in canine gallbladder disease, emphasizing the importance of bile culture in tailoring treatment protocols.

**Disclosures**

**Source of Funding**

**ECVIM CSF Funding**

No.

**ESCG**—**European Society of Comparative Gastroenterology**

## ESCG‐P‐9

## Serum transcobalamin II concentrations are decreased in cats with chronic enteropathy

### T. Kunath^1^, S. Kather^1^, F. Dengler^2^, H. Pfannkuche^3^, E. Nexo^4^, R. Heilmann^1^

#### ^1^College of Veterinary Medicine, University of Leipzig Department for Small Animals, Leipzig, Germany; ^2^Institute of Physiology, Pathophysiology and Biophysics, University of Veterinar, Vienna, Austria; ^3^College of Veterinary Medicine, University of Leipzig Institute of Veterinary Physiology, Leipzig, Germany; ^4^Aarhus University Hospital, Aarhus, Denmark Department of Clinical Biochemistry, Aarhus, Denmark

Measuring the serum cobalamin (vitamin B_12_) concentration has become routine in the diagnostic evaluation of small animal patients with suspected chronic enteropathy (CE) and is used to assess the cobalamin status. Abnormal serum cobalamin concentrations can be detected in cats, dogs, and humans with gastrointestinal diseases but the derangements in cobalamin metabolism are incompletely understood and might be species–specific. Cobalamin is distributed to target tissues bound to transcobalamin II (TCbl II) in blood, which has not been evaluated in cats due to the lack of a TCbl II test for feline samples. This study aimed to establish an in‐house enzyme‐linked immunosorbent assay (ELISA) to quantify serum total TCbl II concentrations in cats with CE and in relation to other conditions and serum cobalamin states.

Surplus feline sera served to validate a sandwich‐ELISA using serially diluted (surplus) human serum as a calibrator and a rabbit polyclonal antibody against recombinant human TCbl II as capturing and detecting reagent. Analytical validation of the assay comprised determining the lower detection limit (LOD), dilutional linearity, spiking‐and‐recovery, intra‐ and inter‐assay variability, and a preliminary reference interval. Serum TCbl II concentrations were measured and compared among 147 cats categorized as either CE, gastrointestinal neoplasia, cholangiohepatopathy, other neoplastic or non‐neoplastic conditions, or healthy controls.

The assay LOD was 160 aU/L. Observed‐to‐expected ratios for five serial two‐fold dilutions ranged from 72.4% to 145.6% and were 75.1% to 126.7% for four different serum spikes. Intra‐ and inter‐assay coefficients of variation were 1.8–17.7% (*n* = 4 samples) and 7.3%–17.2% (*n* = 3). The preliminary reference interval for serum TCbl II was 160–2795 aU/L. Serum TCbl II concentrations were significantly reduced and showed a trend for an inverse association with serum cobalamin concentrations in cats with CE (*P* = 0.0800). Increased serum TCbl II concentrations (>2795 aU/L) were exclusively seen in hypercobalaminemic cats, and the highest TCbl II concentrations were detected in cats with advanced‐stage chronic kidney disease (CKD).

The TCbl II‐ELISA was linear, accurate, precise, reproducible, and sufficiently analytically sensitive, making it suitable for measuring TCbl II in feline samples. TCbl II variation associated with cobalamin deficiency states in feline CE (similar to cases of cholangiohepatopathy) could result from the inflammatory response or anti‐TCbl II‐autoantibodies as described in humans. Further studies are warranted to determine whether serum TCbl II variation in cats with CE contributes to reduced intracellular availability of cobalamin, changes with supplementation, and is affected (and to what extent) by concurrent CKD.

**Disclosures**

N/A.

**Source of Funding**

Purina PetCare Germany generously supported part of the study.

**ECVIM CSF Funding**

No.

**ESCG**—**European Society of Comparative Gastroenterology**

## ESCG‐P‐10

## Effects of short‐term immunosuppressive corticosteroid therapy on 1,2‐o‐dilauryl‐rac‐glycero‐glutaric acid (6'‐methylresorufin)‐ester lipase activity and canine pancreatic‐specific lipase concentration in healthy dogs

### M. Mantelli^1^, C. Fauquet^1^, B. Roques^2^, M. Quincey^1^, A. Marchand^1^, F. Jolivet^3^, N. Jousserand^4^, A. Diquelou^4^, B. Reynolds^5^, T. Buronfosse^6^, R. Lavoué ^5^

#### ^1^Université de Toulouse, ENVT, Toulouse, France; ^2^Department of Physiology & Therapeutics, Ecole Nationale Veterinaire de Toulouse, Toulouse, France; ^3^Centre Hospitalier Vétérinaire Languedocia, Montpellier, France; ^4^IRSD, INSERM U 1220 UMR INRA – ENVT; Ecole Nationale Vétérinaire de Toulouse, Toulouse, France; ^5^INTHERES, Université de Toulouse, INRAE, ENVT, Toulouse, France; ^6^Université de Lyon, VetAgro Sup, Laboratoire de Biologie Médicale, Marcy L'Etoile, France

The effect of corticosteroids on serum canine pancreatic lipase concentration (Spec cPL) and plasma 1,2‐o‐dilauryl‐rac‐glycero‐glutaric acid (6'‐methylresorufin)‐ester lipase (DGGR) activity is unclear. The objective was to determine the effects of prednisolone administered at immune‐suppressing dosage on canine pancreatic lipase Spec cPL concentrations and DGGR lipase activity in healthy dogs.

Fourteen healthy beagle females were enrolled in this prospective, randomized, double‐blind, placebo‐controlled study. They received either an immune‐suppressive dosage of prednisolone (1.5 mg/kg BID) or a placebo during 7 days. Clinical examinations and blood tests were performed before treatment (D0), at the end of treatment (D7), and 2 weeks (D21) and 4 weeks (D35) after the end of treatment. All serum and plasma samples (stored at −80°C) used in the present study were surplus specimen from a previous study which was approved by the local Animal Welfare Body under an Ethical license provided by the national competent authority (Ethical Committee No. 086 of the Ministry of National Education, Universities and Science). No further procedure performed on animal research was conducted.

In the present study, the left‐over samples were used to evaluated Spec cPL concentration, DGGR lipase activity, cholesterol and triglyceride concentrations at each timepoints.

None of the dogs showed clinical signs of pancreatitis throughout the study. On day 7, DGGR lipase activity was significantly higher in the prednisolone group (+76 IU/L, *P*<0.05) and was above reference intervals in 3 dogs. No significant change remained after day 21. No significant effect was observed for Spec cPL (*p* = 0.08), cholesterol (*p* = 0.21) and triglycerides (*p* = 0.94) concentrations. Strong correlation between DGGR lipase and Spec cPL (*r* = 0.986) was observed throughout the study.

Immunosuppressive treatment with prednisolone for 7 days immediately and reversibly increased DGGR lipase activity in healthy dogs. No significant effect on Spec cPL was observed in this small population. The origin of this increase is unclear and may reflect transient or subclinical pancreatitis or extrapancreatic factors. During corticosteroid therapy, canine DGGR lipase levels should be interpreted with caution.

**Disclosures**

**Source of Funding**

**ECVIM CSF Funding**

No.

**ESCG**—**European Society of Comparative Gastroenterology**

## ESCG‐P‐11

## Comparison of clinical and paramedical abnormalities between dogs with food‐responsive enteropathy and dogs with immunosuppressant‐responsive enteropathy: A retrospective study

### E. Berro^1^, F. Lodarski^2^, A. Lamoureux^1^, M. Cervone^2,3^

#### ^1^Centre Hospitalier Vétérinaire Anicura Nordvet, La Madeleine, France; ^2^Clinique Vétérinaire Sevetys Crozatier, Paris, France; ^3^Université de Lyon, VetAgro Sup, Lyon, France

Food‐responsive enteropathy (FRE) and immunosuppressant‐responsive enteropathy (IRE) are commonly diagnosed in dogs, with no clearly identified factors predicting which category dogs might belong to. Moreover, dogs suffering from IRE could be believed to have worse clinical signs and outcome than dogs with FRE.

The aim of this study was to compare clinical and paramedical data, and outcome between dogs having either FRE or IRE.

Multicenter retrospective medical record review was performed, between 2020 and 2023. Signalment, clinical signs, diagnostic findings, and outcome were compared between dogs with FRE and IRE (level of significance set at *p*<0.05).

Overall, 116 dogs were included: 61 FRE‐dogs and 55 IRE‐dogs. FRE‐dogs were significantly younger (*p* = 0.03) than IRE‐dogs (median of 4 and 5 years, respectively). Frequent clinical signs included vomiting (72%), hyporexia (59%), and diarrhea (51%), without difference between groups. The median CIBDAI score was significantly lower (*p* = 0.04) in FRE‐dogs compared to IRE‐dogs (5 and 6, respectively). No difference between groups was found for biological data. Abdominal effusion was more frequent (*p* = 0.049) in IRE‐dogs (21%) compared to FRE‐dogs (7%), and endoscopic macroscopic lymphangiectasia was more frequently observed (*p* = 0.002) in IRE‐dogs (67%) compared to FRE‐dogs (25%). Median time to complete remission (CR) was significantly shorter (*p* = 0.0009) in FRE‐dogs (28 days) compared to IRE‐dogs (40 days), but the frequency of CR was similar (65% and 79%, respectively).

Neither clinical signs nor diagnostic findings are useful to differentiate FRE‐dogs from IRE‐dogs. Outcome was also similar for both groups, although the time to achieve CR was shorter for FRE‐dogs.

**Disclosures**

**Source of Funding**

**ECVIM CSF Funding**

No.

**ESCG**—**European Society of Comparative Gastroenterology**

## ESCG‐P‐12

## Diet modulates gut microbiota composition and derived bio‐products in healthy dogs

### J. Hernandez^1^, S. Rhimi^2^, A. Jablaoui^2^, V. Mariaule^2^, N. Akermi^2^, T. Méric^3^, H. Mkaoua^2^, M. Wysocka^4^, A. Lesner^4^, M.A. Borgi^5^, E. Maguin^2^, M. Rhimi^2^

#### ^1^Oniris VetAgroBio Nantes Clinical Sciences, Nantes, France; ^2^INRAE, Jouy‐en‐Josas, France; ^3^Oniris VetAgroBio Nantes, Nantes, France; ^4^University of Gdansk, Gdansk, Poland; ^5^Faculty of Sciences of Gafsa, Gafsa, Tunisia

The role of the intestinal microbiota in health and disease is now well‐established in humans and animals. Among the most important gut microbiota bio‐products, short‐chain fatty acids (SCFA) and bile acids (BAs) are known to modulate homeostasis. Diet is identified as a key regulatory factor of the microbiota composition and functions. The objective of the present study was to investigate the impact of a dietary change on the modulation of the fecal microbiota composition and the host response using African semi‐stray dogs.

Eighteen healthy semi‐stray dogs were recruited from several farms in North Africa. The selected dogs were self‐nourished by hunting and human food waste. The 18 dogs were randomly divided into 2 groups. Group 1 (*n* = 6) served as a control and maintained their eating habits. Group 2 (*n* = 12) underwent a dietary transition to a kibble industrial food. Fecal samples were collected at D0 and D40. Metagenomic DNA was extracted, V3‐V4 was amplified then sequenced and analysed. Fecal BAs profiling was assessed by liquid chromatography‐tandem mass spectrometry as described. Short‐chain fatty acids were quantified by gas chromatography‐mass spectrometry as described.

At the phylum level, an increase in the abundance of Bacteroidota and Fusobacteriota along with a decrease in the abundance of Actinobacteria and Proteobacteria was observed. A significant increase in Shannon and Simpson indexes (*p* < 0.001) was observed in group 2 dogs at D40 compared to D0. At the genus level, an enrichment of Bacteroides (*p* < 0.05) and particularly of Butyricicoccus (*p* < 0.001), Erysipelatoclostridium (*p* < 0.001) and Lachnospiraceae NK4A136 (*p* < 0.05) was observed. A decrease in bacterial abundance of Parabacteroides (*p* < 0.001), Enterococcus (*p* < 0.01), and Streptococcus (*p* < 0.001) genera was detected.

The analysis of BAs profiles revealed a significant decrease of primary BAs levels, namely cholic acid and chenodeoxycholic acid after diet change (*p* < 0.01). An increase in two secondary BAs namely hyodeoxycholic acid (HDCA) and lithocholic acid (LCA) was observed at D40 compared to D0. The fecal concentration of isobutyrate, acetate and isovalerate increased significantly (*p*<0.01) after the diet change.

Based on our results, the transition from a natural diet in African semi‐stray dogs to an industrial kibble diet was accompanied by beneficial changes in the composition of the microbiota and its impact on the host homeostasis.

**Disclosures**

**Source of Funding**

**ECVIM CSF Funding**

No.

**ESCG**—**European Society of Comparative Gastroenterology**

## ESCG‐P‐13

## Predisposition of Maine Coons to intestinal intussusception and clinical pathological features of these cats

### M. Dainese^1^, M. Petini^1^, A. Zoia^1^

#### ^1^San Marco Veterinary Clinic Internal Medicine, Veggiano, Italy

Maine Coons (MCs) seem overrepresented for intestinal intussusception in some studies. Aims of the study were to calculate the odd ratio (OR) for this condition in MCs and to evaluate differences in signalment, FCEAI score (made only by biochemical paraments [i.e., serum total protein, phosphate, ALT and ALP]) and cholesterol concentration between sick MCs and sick non‐MC cats and between intussuscepted MCs and non‐MC cats.

Cross‐sectional study including sick MCs and a comparative group of sick cats of other breeds individually matched (1:1 ratio) for age (±6 month), sex, and body weight (±2Kg), randomly extracted from the hospital data base, presented between 2004 and 2023. A third group of other non‐MC cats admitted for intestinal intussusception was also included.

In the study entered 371 sick MCs, 16 with intestinal intussusception (4.31%, 95% CI=2.49–6.91), and 371 control sick cats, none with intestinal intussusception (OR=34.48, 95% CI=2.06–576.99, *P* = 0.0138). Due to the matching strategies no differences were found for age and sex between MCs and control sick cats. Nevertheless, MCs were significantly heavier (median, 5.7Kg; IQR, 4.45–7.15) than control sick cats (median, 5.0Kg; IQR, 3.9–6.15; *P*<0.001), but they had a significantly lower BCS (median 3; IQR, 2–3) than control sick cats (median 3; IQR, 3–4; *P*<0.001). No significant difference was found in FCEAI score between MCs and control sick cats. Finally, cholesterol concentration was significantly lower in MCs (median 119mg/dL; IQR, 96–145) compared to control sick cats (median, 130 mg/dL; IQR, 101–165; *P* = 0.001).

Fifteen other non‐MC cats were hospitalised for intestinal intussusception. Intussuscepted MCs were significantly younger (median, 36 months; IQR, 11.25–58.5) than the other intussuscepted cats (median, 125 months; IQR, 57–143; *P* = 0.0114). There were less neutered female MCs (*n* = 0) than in the other intussuscepted cats (*n* = 5; *P* = 0.0191). Intussuscepted MCs were significantly heavier (median 4.85Kg; IQR, 3.87–6.9) than the other intussuscepted cats (median 3.0Kg; IQR, 2.75–4.15; *P* = 0.0058). No significant differences were found in BCS and FCEAI score between intussuscepted MCs and the other intussuscepted cats. Finally, cholesterol concentration was significantly lower in intussuscepted MCs (median 113mg/dL; IQR, 91.75–124.75) compared to the other intussuscepted cats (median, 156mg/dL; IQR, 141–187; *P* = 0.015).

MCs are predisposed to intestinal intussusception. Their lower BCS in comparison to control sick cats and their lower cholesterol concentration in comparison to control sick cats and to the other intussuscepted cats may suggest the presence of an underlying intestinal malfunction in this breed.

**Disclosures**

**Source of Funding**

**ECVIM CSF Funding**

No.

**ESCG**—**European Society of Comparative Gastroenterology**

## ESCG‐P‐15

## Insect‐based (*Hermetia illucens*) diet as a novel protein source for the management of food‐responsive chronic enteropathy in dogs

### F. Del Baldo^1^, F. Sportelli^2^, B. Di Gregorio^2^, A. Costa^2^, M. Crisonà ^2^, A. Corsini^3^, J.S. Suchodolski^4^, L.F. Da Costa Medina^4^, M. Pietra^2^, C.G. Vecchiato^2^

#### ^1^University of Bologna Department of veterinary medical sciences, Ozzano dell'Emilia, Italy; ^2^University of Bologna, Ozzano dell'Emilia, Italy; ^3^University of Parma, Parma, Italy; ^4^Texas A&M University Gastrointestinal Laboratory, Texas, United States

Insects represent a novel and sustainable protein source for pet food, potentially serving as an alternative option in the nutritional management of chronic enteropathies (CE) in dogs. Additionally, in monogastric animals insect proteins have been proven to be effective in modulating fecal microbiota (FM). The aim of this study was to evaluate the clinical response and changes in FM in dogs with food‐responsive enteropathy (FRE) before and after an elimination dietary trial with an *H. Illucens*‐based‐diet (INS). The same diet was fed to healthy control dogs (HC) for comparison with FRE. Twelve HC and twenty FRE were prospectively enrolled. HC and FRE were fed the INS for 30 and 60 days, respectively. In FRE, clinical improvement was evaluated by comparing body weight (BW) and clinical scores (Canine Inflammatory Bowel Disease Activity Index [CIBDAI], Fecal Score [FS]) before (T0) and after 1 (T1) and 2 months (T2) the dietary trial. Differences in FM between HC and FRE at T0 and T1 were compared. Moreover, changes in FM (analyzed by qPCR) and fecal bacterial metabolites (NH_3_, iso‐acids), in FRE at T1 and T2 were assessed. BW (range) improved from T0 (15.4 kg; 4.1–37) to T1 (16.0 kg; 4.1–35.5) (*P* = 0.02). CIBDAI (range) and FS (range) improved from T0 (4; 2–11 and 5; 2–7, respectively) to both T1 (2;0–6 and 2.5;2–5, respectively) and T2 (1; 0–4 and 2; 2–5, respectively) (*P*<0.001). There was a significant decrease of NH_3_ (μmol/g ± SD) from T0 (47 ± 22) to both T1 (24 ± 10) and T2 (24 ± 13). Also, a significant decrease of iso–butyrate (% ± SD) (1.6 ± 0.9 to 0.74 ± 0.52), and iso‐valerate (% ± SD) (2.36 ± 1.5 to 1.01 ± 0.85) from T0 to T2 was detected (P<0.01). Dysbiosis index (range) was not different between FRE and HC at T0 (−2.6; −4.9 to 3.5 vs. −3.8; −6.1 to −1.7; *P* = 0.08) and T1 (−2.6;‐6.1 to 4.5 vs. −4.6;−6.9 to −0,5; *P* = 0.06) and in FRE between T0 and both T1 and T2 (T0:−2.6;−4.9 to 3.5; T1:−2.7;−6.1 to 4.5; T2:‐2.7;‐5.8 to 3.6; *p* > 0.05). *Faecalibacterium* spp. was lower in FRE compared with HC at T0 (*P* = 0.016). At T1, *Bacteroides* spp. (*P*<0.001), *Fusobacterium* spp. (*P* = 0.008) and *Faecalibacterium* spp. (*P*<0.001) were decreased in FRE compared with HC. *Bifidobacterium* spp. was higher (*P*<0.01) and *C. hiranonis* was lower (*P*<0.001) in FRE from T0 to both T1 and T2. In conclusion, our results suggest that in FRE treatment with the INS led to clinical improvement. However, the impact on FM changes should be further evaluated.

**Disclosures**

Francesca Del Baldo Speaking & consultancies: MYLAV Veterinary Laboratory, Boheringer Ingelheim, Dechra, Royal Canin. Carla Giuditta Vecchiato Speaking & consultancies: Monge, MSD, Prolife‐Zodiaco, Royal Canin Federica Sportelli Speaking & consultancies: Necon Andrea Corsini Speaking & consultancies: MYLAV Veterinary Laboratory, Boheringer Ingelheim, Dechra, Royal Canin. Luis Fernando da Costa Medina Speaking & consultancies: Gastrointestinal Laboratory at Texas A&M University Jan Suchodolski Speaking & consultancies: JSS is an employee of the Gastrointestinal Laboratory at Texas A&M University which offers microbiome testing on a fee‐for‐service basis. Purina Petcare, Royal Canin, IDEXX Laboratories, Hill's, Nutramax Laboratories

**Source of Funding**

This study was funded by Dorado srl, a pet food company which provided the diets used in the study.

**ECVIM CSF Funding**

No.

**ESCG**—**European Society of Comparative Gastroenterology**

## ESCG‐P‐16

## Use of a novel low–fat hydrolysed diet in dogs with chronic enteropathy

### C. Hernández^1^, L. Feo^1^, J. Puig^1^

#### ^1^Anicura Ars Veterinary Hospital, Barcelona, Spain

Diet plays a crucial role in both the development and management of chronic enteropathies. Dietary interventions such as novel or hydrolysed proteins, low‐fat content, high fibre or increased digestibility can be implemented This study aimed to investigate the use of a novel low‐fat, hydrolysed diet as a strategy for managing patients with chronic enteropathy.

This descriptive study included sixteen dogs with chronic enteropathy divided into two groups. Group A (6/16) never received a hydrolysed or novel‐protein diet. Group B (10/16) included dogs that lacked a complete response following at least one previous hydrolysed or novel‐protein trial. The prescription diet contained 20.1 g fat/Mcal metabolizable energy with hydrolysed salmon. Canine Inflammatory Bowel Disease Activity Index (CIBDAI) score and a palatability assessment were registered at each time‐point: before starting the diet (T0), at one month (T1) and at three months (T2). The outcome was categorised as complete response (CR), partial response (PR), and no response (NR), according to the improvement of the CIBDAI score.

The overall CR, PR and NR in the total population were 63.6%, 18.2% and 18.2%, respectively. A total of 4/6 (66.7%) of patients in group A and 6/10 (60%) in group B experienced a CR, 2/6 (33.3%) in group A and 1/10 (10%) in group B experienced PR and 0/6 in group A and 3/10 in group B (30%) experienced NR. No statistically significant differences (*p* = 0.47) were observed in the CIBDAI score between groups A and B at T0. All patients that experienced a CR achieved it at T1, with maintenance of the CIBDAI improvement at T2. The diet was well‐accepted and highly palatable to most dogs (12/16; 75%).

This study suggests that a low‐fat, hydrolysed protein diet may be a suitable option for managing diet‐responsive enteropathies in dogs. In this population, the diet led to an overall response of 81 % (CR and PR patients), emphasising the importance of nutrition management as a cornerstone of the therapeutic approach to chronic enteropathies. Additionally, the high proportion of dogs who experienced a CR suggests this dietary option could be offered as an alternative when other dietary strategies have proven ineffective.

**Disclosures**

This study was supported by Dechra Pharmaceutical, which provided free diet to some clients. L. Feo and J. Puig are employed as consultants by Dechra Pharmaceuticals.

**Source of Funding**

**ECVIM CSF Funding**

No.

**ESCG**—**European Society of Comparative Gastroenterology**

## ESCG‐P‐17

## Evaluation of serum pancreatitis‐associated protein in dogs hospitalized for acute pancreatitis

### M. Sidler^1^, D. Brugger^2^, A.K. Helfer‐Hungerbuehler ^3^, B. Riond^3^, M. Dennler^4^, S. Unterer^1^, P.H. Kook^1^

#### ^1^Clinic for Small Animal Internal Medicine, University of Zurich, Zurich, Switzerland; ^2^Institute for Animal Nutrition and Dietetics, University of Zurich, Zurich, Switzerland; ^3^Veterinary Laboratory and Center for Clinical Studies, University of Zurich, Zurich, Switzerland; ^4^Clinic for Diagnostic Imaging, University of Zurich, Zurich, Switzerland

Pancreatitis‐associated protein (PAP) is secreted from acinar cells during acute pancreatitis (AP) and was found useful when assessing AP disease severity in people. PAP expression has also been observed in chronic intestinal disease. Serum PAP has not been measured in dogs so far. The aim of this prospective study was to evaluate serum PAP concentrations in dogs hospitalized for AP and acute idiopathic gastrointestinal (GI) disease (aiGId).

Forty healthy dogs, 27 dogs with AP, and 49 dogs with aiGId were included. Clinical diagnosis of AP was based on lipase activity (LIPC Roche; substrate DGGR) > 400 U/L (RI, 17–156 U/L) and acute onset (<7 d) of GI signs (vomiting, abdominal pain, diarrhea, lethargy, inappetence). Dogs with acute (<7 d) GI signs, lipase activity within or minimally (20 U/L) above RI, were allocated to the aiGId group. All dogs received standard supportive care. During hospitalization, dogs were daily assessed clinically using the modified canine activity index (MCAI) score. Serum PAP levels (DOG PAP‐1VetBio‐1 SPARCL assay on VetBio‐1 luminometer), lipase activity and CRP (Gentian canine CRP) concentration were measured daily. The PAP assay was internally validated. Data analysis comprised general linear models (group sex) assuming log‐link functions over gamma distributions and spearman correlation coefficients due to the non‐normality of analyzed datasets. A PAP RI was calculated using the 5th and 95th percentiles.

The newly established RI for PAP was 0.35–0.96 mcg/mL. Median (range) lipase activity, CRP, and PAP at admission (d1) was 1862 U/L (441–6712), 80.73 mg/L (6.4–370), 2.48 mcg/mL (0.07–6.5) in AP dogs, and 68 U/L (14–176), 95.7 mg/L (6.4–368), and 2.3 mcg/mL (0.17–6.5) in aiGId dogs. AP and aiGId dogs were hospitalized for a median (range) of 3d (1–5 d; 1–8 d). PAP concentration did not differ significantly between both groups (*p* = 0.08) but decreased significantly over time (*p* = 0.0003). PAP correlated significantly with CRP in both groups irrespective of time points of observation (AP r_s_ 0.616, *p*<0.0001; aiGId r_s_ 0.482, *p*<0.0001). PAP correlated significantly with lipase activity (r_s_ 0.473, *p*<0.0001) in AP dogs irrespective of time points of observation. Only in the aiGId group did PAP (r_s_ 0.342) and CRP (r_s_ 0.4) correlate significantly (p<0.0001) with MCAI irrespective of time points of observation.

In conclusion, serum PAP concentration did not differ between dogs with AP and aiGId, and serum PAP concentration did not correlate with disease severity in AP. Our results suggest that PAP behaves like an acute phase protein, especially in dogs with aiGId.

**Disclosures**

**Source of Funding**

**ECVIM CSF Funding**

No.

**ESCG**—**European Society of Comparative Gastroenterology**

## ESCG‐P‐18

## Micro‐ulcerative gastropathy in cats: A case series

### D. Pichard^1^, C. Liwszyc^1^, M. Le Dudal^1^, V. Freiche^1^

#### ^1^Ecole Nationale Vétérinaire d'Alfort, Maisons‐Alfort, France

Gastric ulceration can result from gastric foreign bodies, neoplasia, gastrointestinal eosinophilic sclerosing fibroplasia and less commonly dietary indiscretion in cats. The role of *Helicobacter* species as gastric pathogens is less recognized. Macroscopic multifocal mucosal micro‐ulcerative gastropathy is still an unknown condition in cats.

The aim of this study was to describe the endoscopic appearance of micro‐ulcerative gastric lesions, and to assess signalment, clinical, paraclinical and histologic data in cats. Cats with endoscopic description of more than 5 millimetric ulcers on gastric wall were retrospectively included between 2016 and 2024. Continuous data were presented as median [interquartile range].

Nineteen cats were included between 2016 and 2024. Median age at presentation was 11 years old [5.5–13]. Median weight was 4.5 kg [4.0–5.0]. Breeds included were Domestic shorthair (*n* = 16), Burmese (*n* = 2) and Ragdoll (*n* = 1). Clinical presentation included chronic vomiting (*n* = 16), weight loss (*n* = 9), hyporexia (*n* = 6), diarrhea (*n* = 5). Gastric ultrasound examination revealed gastritis signs (*n* = 8) with median gastric wall thickness of 3.2 mm [2.1–4.8], proeminent gastric mucosa (*n* = 3) and gastric adenopathy (*n* = 5). Ultrasound signs of enteropathy (*n* = 9) and mesenteric adenopathy (*n* = 2) were associated. Hypocobalaminemia was detected in 3 cases. On endoscopic evaluation, all cases showed more than 5 millimetric ulcers on gastric mucosa with multifocal distribution. An acquired pyloric stenosis was associated in 11 cases (pyloric measurement 8 mm [7.8–8]). Histologic evaluation showed fibrosis (*n* = 11), chronic inflammation (*n* = 9), ulcerative lesions (*n* = 5), lymphoplasmocytic infiltration (*n* = 4), a gastric lymphoma (*n* = 1) and no histologic patent lesion (*n* = 3). *Helicobacter* species were detected in 2 cases. Small intestine biopsies were performed as well in 18 cases and histologic features showed lymphoplasmocytic (*n* = 6) and/or eosinophilic (*n* = 4) infiltration, fibrosis (*n* = 4), feline eosinophilic sclerosing fibroplasia (*n* = 1), ulcerative enteritis (*n* = 1), and no evident histologic lesion (*n* = 1). Treatment included omeprazole (*n* = 14), sucralfate (*n* = 5), hypoallergenic or hyperdigestible diet and specific treatment for associated diseases, and allowed clinical improvement in all cases.

This study describes feline micro‐ulcerative gastropathy, characterized by diffuse multifocal millimetric ulcers on gastric mucosa, associated with chronic vomiting and in 50% of the cases with an acquired pyloric stenosis in cats. Generally, macroscopic lesions were more severe than histologic features.

**Disclosures**

Royal Canin residency funding.

**Source of Funding**

Royal Canin residency funding.

**ECVIM CSF Funding**

No.

**ESCG**—**European Society of Comparative Gastroenterology**

## ESCG‐P‐19

## Chronic inflammatory enteropathies in miniature poodle: Retrospective studies in 131 dogs

### E. Benvenuti^1^, C. Menicocci^1^, P. Ruggiero^1^, E. Bottero^1^

#### ^1^Endovet Professional Association, Rome, Italy

Chronic inflammatory enteropathies (CIE) are commonly classified according to treatment response as food responsive enteropathy (FRE), microbiota‐related modulation‐responsive enteropathies (MrMREs), immunosuppressive or steroid responsive enteropathy (IRE/SRE) and non‐responsive enteropathies (NRE). CIE has been reported in various canine breeds (Weimaraner, Rottweiler, German shepherd dog, Border collie, Boxer, Norwegian Lundehund, West Highland White Terrier) including Miniature Poodle.

The aim of this retrospective study was to analyse CIE in Miniature Poodle and compare FRE/MrMREs to IRE.

Data from a gastroenterology referral veterinary center database were evaluated between January 2020 and December 2023. One hundred and thirty‐one Miniature Poodles were included in this study. In all dogs age, clinical signs, diet, endoscopy and therapy were evaluated after exclusion of non‐gastrointestinal causes. FRE dogs were categorized based an adequate response after at least two diet changes and no additional famacological treatments were required. Pre/probiotic, postbiotics or symbiotics therapy was performed in dogs that had a partial response to diet (MrMREs). Dogs that didn't respond to diet or microbiota‐related modulation and showed an adequate response to immunosuppressant treatment were classified as IRE. In 79 of 131 FRE/MrMREs cases (60%) the median age was 2 years (range: 1–12); while in 52 of 131 cases (40%) of IRE dogs, the median age was 3 years (range: 1–15). In FRE dogs, borborygmi was the main symptom, observed in 67 out of 79 cases (85%), followed by vomiting in 59 cases (75%), diarrhea in 56 cases (70%), dysorexia in 45 cases (57%), weight loss in 37 cases (75%), sickness in 36 cases (45%) and abdominal pain in 22 cases (28%). In IRE dogs, vomiting was the main symptom observed in 42 out of 52 cases (81%), followed by diarrhea in 40 cases (77%), dysorexia in 32 cases (62%), sickness in 30 cases (58%), borborygmi in 27 cases (52%), weight loss in 22 cases (42%), and abdominal pain in 12 cases (23%). The hydrolysed diet was the most common used (64/131) followed by homecooked diets (55/131), easily digestible diets (5/131), low‐fat diets (5/131), and single‐protein ones (2/131). Endoscopy evaluation was performed in 61 dogs (46,5%) and immunosuppressive treatment, consisting in prednisolone or methylprednisolone, was used in 52 dogs (40%).

In conclusion, in this study, Miniature Poodles showed a poorer response to diet. IRE was present in 40% of cases and vomiting was the main symptom. Furthermore, IRE may be present in young dogs (<4 years)

**Disclosures**

**Source of Funding**

**ECVIM CSF Funding**

No.

**ESCG**—**European Society of Comparative Gastroenterology**

## ESCG‐P‐20

## Relationship between folate and cobalamin serum concentrations and small intestinal ultrasonographic abnormalities in dogs with gastrointestinal signs

### E. Hernando Montalbán^1^, B. Benages Saura^2^, V. Herrería Bustillo^1^, A. Vila Soriano^1^, R. Saiz Álvarez^1^

#### ^1^Hospital Veterinario de la Universidad Católica de Valencia, Valencia, Spain; ^2^Hospital Veterinario Puchol, Madrid, Spain

Abdominal ultrasound is commonly included in the diagnostic approach of gastrointestinal diseases. However, it is unclear if ultrasonographic findings can help to identify dogs with folate or cobalamin deficiency. The main objective of the study was to determine the relationship between serum folate/cobalamin concentrations and small intestinal ultrasonographic abnormalities in dogs with gastrointestinal clinical signs. Secondary aims were to assess the potential correlation between both vitamin concentrations and the duration of clinical signs and serum albumin and cholesterol concentrations.

Records for client‐owned dogs presenting with gastrointestinal signs and in which serum folate and cobalamin were measured from 2022 to 2023 were reviewed. Inclusion criteria comprised dogs with acute (<3 weeks) or chronic (>3 weeks) gastrointestinal signs who underwent abdominal ultrasound within a month of folate/cobalamin determination. Dogs with exocrine pancreatic insufficiency and those previously supplemented with folate or cobalamin were excluded.

Dogs were classified based on its serum folate and cobalamin concentrations: hyper/normocobalaminemia (>400 μg/ml), suboptimal cobalaminemia (400–275 μg/ml), hypocobalaminemia (<275 μg/ml), hyper/normofolatemia (>8.2 ng/ml), hypofolatemia (<8.2 ng/ml) and hyper/normofolatemia (>8.2ng/ml). Small intestinal ultrasound abnormalities were categorized as wall thickening, loss of layer differentiation, mucosal and functional alteration.

A total of 105 dogs were included in this study. Among them, 68/105 (64.8%) dogs exhibited chronic and 37/105 (35.2%) acute clinical signs. Median folate and cobalamin concentrations were 9.25ng/ml (2.94–24) and 538ng/dl (150–1200) respectively. Thirty‐six dogs (34.3%) had hypofolatemia and 69/105 (65.7%) hyper/normofolatemia. In the cobalamin group, 67/105 dogs (63.8%) were hyper/normocobalaminemic, 20/105 (19%) suboptimal cobalaminemic and 18/105 (17.1%) hypocobalaminemic. Median plasmatic albumin and cholesterol concentrations were 2.96g/dl (1.3–5) and 178.54mg/dl (39–340) respectively.

Seventy‐three (69.5%) dogs showed intestinal ultrasound abnormalities. The most common findings were mucosal alterations (4.3%), followed by wall thickening (26.7%), functional alterations (23.8%) and loss of layer differentiation (6.7%). 41/67 (61.2%) normo/hypercobalaminemic, 19/20 (95%) suboptimal cobalaminemic, and 13/18 (72.2%) hypocobalaminemic, 31/36 (86.1%) hypofolatemic and 55/69 (79.7%) normo/hyperfolatemic dogs exhibited ultrasonographic abnormalities.

Statistical analysis revealed no significant differences in folate (*p* = 0.964) and cobalamin (*p* = 0.223) concentrations between dogs with or without small intestinal ultrasound abnormalities. None of the ultrasonographic abnormalities correlated with folate (*R* ^2^=0.73, *F*=1.964, *p* = 0.106) and cobalamin (*R* ^2^=0.21, *F*=0.536, *p* = 0.709) in the multiple linear regression. Duration of clinical signs, plasmatic albumin and cholesterol did not correlate with folate (*p* = 0.557, *p* = 0.481, *p* = 0.222) and cobalamin (*p* = 0.562, *p* = 0.840, *p* = 0.315) concentrations.

This study suggests that neither intestinal ultrasonographic findings, duration of clinical signs nor albumin/cholesterol concentrations should be considered predictors of folate and cobalamin status in dogs with gastrointestinal signs.

**Disclosures**

**Source of Funding**

**ECVIM CSF Funding**

No.

**ESCG**—**European Society of Comparative Gastroenterology**

## ESCG‐P‐21

## Fecal calprotectin concentrations during administration of oral cyclosporine A in healthy cats

### S. Rösch^1^, J. Suchodolski^2^, J. Cavasin^2^, R. Mischke^1^

#### ^1^Small Animal Clinic, University of Veterinary Medicine Hannover, Foundation Internal Medicine, Hannover, Germany; ^2^Gastrointestinal Laboratory, Texas A&M University, College Station, Texas, United States

Fecal calprotectin (S100A8/A9 protein complex, fCal) has utility as a non‐invasive biomarker for assessment of inflammation in inflammatory bowel disease in humans. In cats, fCal has been shown to be increased in a subset of animals with chronic inflammatory enteropathies (CIE). Cyclosporine A (CsA) is recommended as one of the treatment options for feline CIE, despite CsA itself is possibly initially leading to gastrointestinal side effects and maybe worsening clinical signs in the beginning of CIE‐treatment. Therefore, it was investigated as a secondary aspect in a pharmacokinetic study with a high‐dose CsA administration regimen in healthy cats whether CsA application per se has an influence on the fCal concentration.

In this prospective study, six healthy domestic shorthair cats were orally administered 7 mg/kg CsA q 12 hours over 10 days under well‐defined experimental conditions. Fecal samples were analyzed by an immunoturbidimetric assay (fCal turbo, Bühlmann Laboratories, Switzerland) verified for feline samples before, during 10 days of CsA administration as well as up to 3 days after administration of CsA. For statistical analyses of fCal concentrations, Kruskal Wallis test was performed due to not normally distributed data with *p* < 0.05 considered to be significant.

In 6/6 cats, baseline fCal concentrations (median: 23.6 μg/g feces, minimum–maximum: 23.6–80.1 μg/g feces) were within the reference interval (<140 μg/g feces). During the experimental trial and despite mild gastrointestinal side effects (2/6 cats once vomiting and 5/6 cats softer feces), fCal concentrations stayed within the reference interval except in two samples. Statistical analysis revealed no significant changes of fCal concentration during the trial.

In conclusion, even if gastrointestinal signs are observed, high‐dose application of CsA has no impact on fCal concentrations, which is of clinical relevance when interpreting fCal concentrations in monitoring treatment response to CsA in CIE cats.

**Disclosures**

None.

**Source of Funding**

None.

**ECVIM CSF Funding**

No.

**ISCAID**—**International Society for Companion Animal Infectious Diseases**

## ISCAID‐P‐1

## Investigation of possible recombination between feline corona virus and SARS‐CoV‐2 in cats with feline infectious peritonitis

### D. Pavlin^1^, G. Grubelnik^2^, R. Kogoj^2^, A. Suljič ^2^, N. Tozon^1^, A.Ž. Tatjana^2^, M. Korva^2^

#### ^1^University of Ljubljana, Veterinary faculty, Clinic for small animals, Ljubljana, Slovenia; ^2^University of Ljubljana, Faculty of medicine Institute of Microbiology and Immunology, Ljubljana, Slovenia

During and after the COVID‐19 epidemic, we have seen a significant increase in the number of cats with feline infectious peritonitis (FIP). It has already been reported that humans are susceptible to feline coronavirus (FCoV) infection, and during the epidemic it has been shown that cats can be infected with SARS‐CoV‐2, suggesting that interspecies transmission is possible. Based on these facts, we aimed to investigate possible recombination event between FCoV and SARS‐CoV‐2 that could provide an explanation for the increase in FIP cases during the epidemic.

A total of 6 cats with clinically confirmed FIP according to the European Advisory Board on Cat Diseases diagnostic tool were included in the study, ranging in age from 3 months to 12 years. 4/6 cats had effusive FIP and 2/6 had non‐effusive FIP. In 5/6 cats, the owners opted for euthanasia and one cat was treated. One set of samples was taken as part of either the diagnostic or palliative procedure (thoracocentesis, abdominocentesis, fine needle biopsy of lymph nodes) and the leftover samples were used with the owners' consent. Another set of samples was taken immediately post mortem (tissue samples from spleen, lymph nodes and liver). The samples were analysed using metagenomics next generation sequencing (NGS). The Pan‐Coronavirus Enrichment Panel with Illumina RNA Prep was used for the NGS application. A custom in‐house bioinformatics pipeline was used to analyse the results.

The presence of FCoV was confirmed in various samples from five clinically affected cats, as the highest proportion of both raw reads and contigs corresponding to FCoV was detected among other putative viral reads. It was possible to assemble 47.5%–91.7% of FCoV genomes in individual cats. A phylogenetic analysis of the obtained FCoV genomes for these five cats was performed using the reference sequence of the SARS‐CoV‐2 virus (Wuhan‐Hu‐1_NC_045512.2) and the FIPV reference sequence (NC_002306.3). The results of the phylogenetic differences showed that all five FCoV genomes analysed were relatively similar to each other, but differed strongly from the SARS‐CoV‐2 reference sequence.

From the results of our study, we can conclude that a recombination event between FCoV and SARS‐CoV‐2 is highly unlikely, as we could not detect any genetic elements of SARS‐CoV‐2 genetic elements in the FCoV isolated from cats diagnosed with FIP.

**Disclosures**

**Source of Funding**

**ECVIM CSF Funding**

No.

**ISCAID**—**International Society for Companion Animal Infectious Diseases**

## ISCAID‐P‐2

## Haematological ratios in dogs with leishmaniosis: Implications for disease classification and survival

### M. Monroig Carbonell^1,2^, P.F. Navarro Martinez^2^, R. Saiz Álvarez^2,3^, A. Vila Soriano^2,3^, J. Castro López^4^, I. Mesa Sánchez^5^, L. Gil Vicente^2,3^

#### ^1^Doctoral School, Universidad Católica de Valencia San Vicente Mártir, Valencia, Spain; ^2^Department of Animal Medicine and Surgery, Universidad Católica de Valencia, Valencia, Spain; ^3^Internal Medicine Department, Hospital Veterinario de Referencia UCV, Valencia, Spain; ^4^Internal Medicine Department, AniCura Valencia Sur, Valencia, Spain; ^5^Internal Medicine Department, IVC Evidensia Aúna Especialidades Veterinarias, Valencia, Spain

Different leukocyte markers such as neutrophil‐to‐lymphocyte ratio (NLR), platelet‐to‐lymphocyte ratio (PLR), monocyte‐to‐lymphocyte ratio (MLR) and the systemic immunity‐inflammation index (SII), have been studied in veterinary medicine as prognostic factors in various diseases, including acute diarrhoea, pancreatitis, chronic enteropathies, meningoencephalitis of unknown origin, pneumonia and parvovirus. However, there is limited research evaluating these ratios in dogs with leishmaniosis. To the authors' knowledge, no study has specifically investigated the MLR in this disease.

The objective of this study was to compare haematological ratios among patients with leishmaniosis across different disease stages and to examine their association with patient survival.

Of the 185 samples initially analysed from dogs with leishmaniosis, 59 were excluded due to concurrent diseases or the administration of immunosuppressive therapy or NSAIDs at the time of sampling. Finally, 126 dogs were analysed from three veterinary hospitals between 2018 and 2024.

The NLR, MLR, and PLR were calculated by dividing the total parameter count by the total lymphocyte count, while SII was calculated by dividing the total neutrophil count by the total lymphocyte count and multiplying the result by the total platelet count. The normality assumption was assessed using the Shapiro–Wilk test. Given the population's non‐normal distribution, disparities among disease stages were assessed using the Kruskal–Wallis rank sum test. Logistic regression was used to examine the correlation between ratios and patient prognosis.

The comparison of haematological ratios between the stages of leishmaniasis did not show statistically significant differences in any parameter NLR (*p* = 0.243), PLR (*p* = 0.173), MLR (*p* = 0.140) and SII (*p* = 0.085). Haematological ratios and the probability of death in dogs with leishmaniosis were assessed using logistic regression analysis. No significant differences were observed in the NLR (*p* = 0.138), PLR (*p* = 0.931), and SII (*p* = 0.602). Nonetheless, a trend towards decreased MLR was noted, supported by a negative estimated regression coefficient variable (‐1.543), suggesting an inverse relationship with the probability of death (*p* = 0.069).

In conclusion, this study provides valuable insights into haematological ratios in dogs with leishmaniosis. It suggests further research to fully understand their role in the disease and their association with patient survival. Therefore, expanding the study population and assessing the evolution of these ratios throughout leishmaniosis treatment would be beneficial. This could help determine whether these ratios work as reliable indicators of disease progression and aid in predicting the increased risk of mortality based on their outcomes.

**Disclosures**

**Source of Funding**

Study carried out without funding.

**ECVIM CSF Funding**

No.

**ISCAID**—**International Society for Companion Animal Infectious Diseases**

## ISCAID‐P‐3

## Comparison of haematological ratios (neutrophil‐to‐lymphocyte ratio, platelet‐to‐lymphocyte ratio, monocyte‐to‐lymphocyte ratio, and systemic immunity‐inflammation index) between healthy dogs and those with leishmaniosis

### M. Monroig Carbonell^1,2^, P.F. Navarro Martinez^2^, R. Saiz Álvarez^2,3^, A. Vila Soriano^2,3^, J. Castro López^4^, I. Mesa Sánchez^5^, L. Gil Vicente^2,3^

#### ^1^Doctoral School, Universidad Católica de Valencia San Vicente Mártir, Valencia, Spain; ^2^Department of Animal Medicine and Surgery, Universidad Católica de Valencia, Valencia, Spain; ^3^Internal Medicine Department, Hospital Veterinario de Referencia UCV, Valencia, Spain; ^4^Internal Medicine Department, AniCura Valencia Sur, Valencia, Spain; ^5^Internal Medicine Department, IVC Evidensia Aúna Especialidades Veterinarias, Valencia, Spain

Different leukocyte markers such as neutrophil‐to‐lymphocyte ratio (NLR), platelet‐to‐lymphocyte ratio (PLR), monocyte‐to‐lymphocyte ratio (MLR) and the systemic immunity‐inflammation index (SII), they have been studied in veterinary medicine as prognostic factors. Consequently, several studies have compared these haematological ratios between healthy subjects and those with specific diseases, including pancreatitis, myxomatous mitral valve disease, and chronic enteropathy, revealing notable differences between the two groups. Additionally, to the authors' knowledge, no study has specifically assessed the MLR in dogs with leishmaniosis.

The aim of this study was to compare these haematological ratios between healthy dogs (control group) and dogs with leishmaniosis.

From the initial 185 leishmaniosis blood samples, 59 were excluded due to concurrent pathologies such as cancer, systemic infections, or concurrent anti‐inflammatory drug administration at the time of sampling. A total of 126 samples from dogs with leishmaniosis were finally included and compared with 120 samples from healthy dogs to assess the NLR, MLR and SII, and 90 samples for PLR (dogs with platelet aggregates were excluded). All samples were obtained from patients from three veterinary hospitals between 2018 and 2024. NLR, MLR, and PLR were calculated by dividing the total parameter count by the total lymphocyte count, while SII was calculated by dividing the total neutrophil count by the total lymphocyte count and multiplying the result by the total platelet count. The normality assumption was assessed using the Shapiro–Wilk test. Since the population did not exhibit a normal distribution, differences between the groups were evaluated using the Wilcoxon‐Mann–Whitney test.

The comparison of haematological ratios between both groups showed statistically significant differences in NLR and MLR ratios, except for PLR (*p* = 0.909) and SII (*p* = 0.975). The median NLR in seropositive dogs (3.790 [2.303–6.560] was significantly higher than in healthy dogs (2.703 [2.055–3.474]) (*p* = 5.565e^−05^). The median MLR in seropositive dogs (0.355 [0.230–0.575] was significantly higher than the control group (0.143 [0.118–0.190]) (*p* = 2.2e^−16^).

Regarding NLR and MLR, it showed that patients with leishmaniosis had a higher ratio than healthy ones, which could be due to an increase in leukocytes in animals infected by *Leishmania infantum*. These results point out significant differences between different leukocytes proportions from healthy dogs and those infected by Leishmania infantum. Therefore, they could be used to aid in the diagnosis and monitoring seropositive patients.

**Disclosures**

**Source of Funding**

Study carried out without funding.

**ECVIM CSF Funding**

No.

**ISCAID**—**International Society for Companion Animal Infectious Diseases**

## ISCAID‐P‐4

## Comparison of neutrophil myeloperoxidase index in dogs with canine parvoviral enteritis to a healthy control population

### L. Loubser^1^, G. Fosgate^2^, W. Botha^1^, Y. Rautenbach^1^, A. Celliers^3^

#### ^1^University of Pretoria Department of Companion Animal Studies, Faculty of Veterinary Science, Pretoria, South Africa; ^2^University of Pretoria Department of Production Animal Studies, Faculty of Veterinary Science, Pretoria, South Africa; ^3^Kansas State University Department of Clinical Sciences, College of Veterinary Medicine, Manhattan, Kansas, United States

The myeloperoxidase index (MPXI) is a value attributed to the average myeloperoxidase (MPO) content of circulating neutrophils, determined by the balance between MPO synthesis and release during neutrophil maturation and activation/degranulation respectively. In human and veterinary medicine, MPXI is utilised as an indicator of neutrophil activity and systemic inflammation, which may provide prognosticatory information in certain conditions. Canine parvovirus (CPV) often results in a marked systemic inflammatory response but is also associated with a marked neutropaenia through massive neutrophil consumption or destruction of precursors in the bone marrow and lymphoid organs. Investigation of neutrophil counts and associated changes in MPXI may provide further information on the immune response to this disease. This study aimed to describe the neutrophil MPXI measured at presentation in dogs diagnosed with parvoviral enteritis compared to a group of healthy age‐matched control dogs.

Thirty‐two dogs diagnosed with parvoviral enteritis, and 12 healthy age‐matched controls were included in this retrospective study. Affected dogs were between 6 weeks and 12 months of age, of any breed or sex, weighed at least 3 kg, did not receive antibiotic/anti‐inflammatory treatment in the preceding two weeks and were diagnosed CPV positive using a validated commercial antigen ELISA with the infection confirmed via faecal electron microscopy. MPXI and neutrophil counts were measured on blood collected in an EDTA tube at presentation, using the ADVIA 2120 haematology analyser.

The mean (± standard deviation) and median (minimum–maximum) neutrophil count and MPXI of the affected and control dogs were calculated and compared using an independent *t*‐test. There was no significant difference in the mean (5.2 × 10^9^/L ± 4.4) and median (4.0 × 10^9^/L; 0.1–14.6) neutrophil count in dogs with parvoviral enteritis compared to the mean (6.7 × 10^9^/L ± 1.6) and median (7.0 × 10^9^/L; 3.9–9.0) count in the control dogs (*P* = 0.305). There was also no significant difference in the mean (17.7 ± 5.9) and median (18.8; 9.3–30.3) MPXI in dogs with parvoviral enteritis compared to the mean (20.9 ± 7.9) and median (19.9; 8.2–35.6) MPXI in control dogs (*P* = 0.357).

This study showed that at presentation, the MPXI and neutrophil count were similar between parvoviral enteritis and healthy control dogs. Prospective studies in dogs with parvoviral enteritis are needed to determine if, and how, the MPXI changes during the course of the disease, along with its potential use in prognostication.

**Disclosures**

**Source of Funding**

Department of Veterinary Tropical Diseases (DVTD) Postgraduate Scholarship (Masters or Doctoral Study Programmes); Faculty of Veterinary Science; University of Pretoria (UP)

**ECVIM CSF Funding**

No.

**ISCAID**—**International Society for Companion Animal Infectious Diseases**

## ISCAID‐P‐5

## Acute kidney injury as the main cause of hospitalization and death in dogs with leishmaniosis

### M.A. Daza^1^, C. Garrido^2^, L. Alaman^1^, V. Dominguez^3^, G. Miró ^4^

#### ^1^Veterinary Clinical Hospital. Veterinary Faculty. Universidad Complutense, Madrid, Spain; ^2^Veterinary Clinical Hospital. Veterinary Faculty. Universidad Complutense, Madrid, Spain; ^3^Animal Health Department. Veterinary Faculty. Universidad Complutense de Madrid, Madrid, Spain; ^4^Veterinary Hospital Animal Health Department Universidad Complutense Madrid, Madrid, Spain

Canine leishmaniosis is a vector borne disease caused by Leishmania infantum in Southern Europe. The clinical manifestations in dogs are variable but cutaneous, ocular, orthopedical and/or renal injuries are the most severe clinical picture of bad prognosis.

The aim of this study was to determine the principal causes of hospitalization in dogs with leishmaniosis in a referral Hospital. Medical records of dogs presenting at the Veterinary Teaching Hospital from January 2013 to December 2023 were retrospectively evaluated.

A total of 141 patients, previously diagnosed with leishmaniosis, went to the emergency room: 58.2% were males and 41.8% females, with a mean age of 6.7 ± 3.5 years old. There were dogs from different breeds, being the most prevalent ones: crossbreed dogs (22%) and Spanish Greyhounds (10,6%). According to the LeishVet classification, 29.1% were in stage 2; 20.6% in Stage 3 and 50,4% in stage 4. The reasons for consultation were renal disease (58.5%); haemostatic disorders [epistaxis (12%), thrombosis (2%)]; digestive (6.3%), haematological (5%); orthopedic (3,5%); dermatologic (2,1%); endocrine (2.1%); oncologic (2.1%); neurologic disorders (2.1%); and/or other concurrent infectious (2,1%); reproductive (0.7%) or hepatic diseases (0.7%). Mean hospitalization time was 2.7 ± 2,9 days; with a mortality rate of 31,2% (while the mortality of 7.1% of the population was not determined on the medical records). On the other hand, the 61.7% of the population survived to the hospitalization. The relapse rate of CanL along the study period was 20.6%.

As the most frequent reason for hospitalization was renal disease following IRIS acute kidney injury classification, we can describe our population as: 1.5% in stage 1; 14.5% in stage 2; 41% in Stage 3; 28.9% in stage 4; 10.8% in stage 5 and 3.6% in unknown stage. Mean serum creatinine in the renal disease group was 5 ± 3,1 mg/dL. The 98.8% (82/83 animals) of the patients with renal disease had an acute on chronic kidney disease injury (they were previously diagnosed with chronic kidney disease) and 1.2% (1/83 animal) had Shar Pei autoinflammatory disease.

In conclusion, the most frequent cause of hospitalization in dogs with leishmaniosis in the study population was acute kidney injury, followed by haemostatic and digestive disorders.

An early diagnosis of renal disease in dogs with clinical leishmaniosis may be crucial to avoid or reduce mortality rate.

**Disclosures**

**Source of Funding**

**ECVIM CSF Funding**

No.

**ISCAID**—**International Society for Companion Animal Infectious Diseases**

## ISCAID‐P‐6

## Exploring the impact of initial treatment on clinical outcomes in canine leishmaniosis: A retrospective analysis over 20 years (2000–2020)

### J. Sarquis^1^, C. Rodríguez Sanz^2^, V. Domínguez^1^, J.P. Barrera^1^, A. Montoya^1^, G. Miró ^1^

#### ^1^Universidad Complutense de Madrid Animal Health Department, Madrid, Spain; ^2^Spanish Agency for Medicines and Medical Devices Department of Veterinary Medicines, Madrid, Spain

The standard treatment for canine leishmaniosis (CanL) in Europe is based on a combination of meglumine antimoniate and allopurinol, although treatment protocols may vary. In this retrospective study, we aimed to investigate if the first round of treatment after diagnosis could impact the clinical outcome of dogs with CanL.

Data from 1146 dogs diagnosed with CanL between 2000 and 2020 was used to perform this study. Information about the first round of treatment and corresponding outcomes was available for 563 of these patients. A favourable outcome was defined as dogs showing improvement in their clinical stage following treatment, with no recurrence of clinical signs. An unfavourable outcome was characterized by the progression of clinical signs and clinicopathological disorders despite treatment, or eventual natural death/euthanasia due to CanL. Dogs with unchanged clinical stages were categorised as stable.

Statistical analyses included Fisher's exact test to assess associations between treatment and outcomes across all combinations and a chi‐square test limited to combinations with adequate representation (>5 observations). Standardized residuals from the chi‐square statistic were utilized to interpret associations, highlighting deviations from expected frequencies. All statistical analyses were performed using R software. The threshold of significance was set at *p*<0.05.

Twenty‐one different combinations of treatments were identified, with meglumine antimoniate plus allopurinol (*n* = 543, 47.38%) and allopurinol plus miltefosine (*n* = 137, 11.95%) being the most common. Allopurinol as a monotherapy was the second most common first‐course treatment reported (*n* = 290, 25.31%). Data about treatment was missing in 42 dogs (3.66%) and 52 (4.54%) received no treatment. Regarding outcome, 224 dogs (19.55%) had a favourable outcome, 194 (16.93%) had an unfavourable outcome and 145 (12.65%) remained clinically stable.

Our results indicate a significant association between the first course of treatment and clinical outcome (*p* = 0.0005, Fisher's exact test). Our second analysis also revealed significant associations between treatment and outcomes (Chi‐square statistic = 18.018, df = 4, *p* = 0.001). Specifically, meglumine antimoniate plus allopurinol (*n* = 285) demonstrated a higher‐than‐expected frequency of favourable outcomes, while miltefosine plus allopurinol exhibited a higher‐than‐expected frequency of unfavourable outcomes. Conversely, allopurinol monotherapy was associated with a higher‐than‐expected frequency of acceptable outcomes. In conclusion, our study underscores the importance of considering treatment regimens in managing CanL, with potential implications for clinical decision‐making.

**Disclosures**

**Source of Funding**

**ECVIM CSF Funding**

No.

**ISCAID**—**International Society for Companion Animal Infectious Diseases**

## ISCAID‐P‐7

## Prevalence of co‐infections with *Ehrlichia* spp. or *Theileria* spp. in dogs naturally infected with babesiosis in the Eastern Cape province, South Africa

### H. Cloete^1,2^, Y. Rautenbach^1^, A. Leisewitz^1,3^, R. Mellanby^4^, P. Thompson^5^, J. Schoeman^1^

#### ^1^University of Pretoria Companion Animal Clinical Studies, Pretoria, South Africa; ^2^University of Liverpool Companion Animal Clinical Studies, Liverpool, United Kingdom; ^3^Auburn University College of Veterinary Medicine Companion Animal Clinical Studies, Auburn, United States; ^4^University of Edinburgh, Edinburgh, United Kingdom; ^5^University of Pretoria Production Animal Studies, Pretoria, South Africa

Canine babesiosis and ehrlichiosis are tick‐borne infections of great significance in South Africa. Theileriosis in dogs in South Africa is still poorly understood. Co‐infection with multiple tick‐borne diseases has been documented and is perceived as a common occurrence in South Africa.

The main objective of this study was to determine the prevalence of co‐infections with *Ehrlichia* spp. or *Theileria* spp. in dogs with babesiosis in the Eastern Cape province. There is a lack of data on canine tick‐borne disease distribution in this region. Possible associations of population characteristics and haematological and biochemistry measures with a co‐infection of *Ehrlichia* spp. or *Theileria* spp. in these dogs were also investigated.

The study population included 150 dogs naturally infected with babesiosis presented to the Mdantsane State Veterinary Clinic between January 2021 and November 2021. Quantitative polymerase chain reaction was used to confirm the *Babesia* spp. that the dogs were infected with and to identify co‐infections. Association with co‐infection for the following parameters were evaluated: sex, breed, age, duration of illness, band neutrophil count, and serum globulin concentration. Positive and negative predictive values of monocytosis, leukopenia, band neutrophilia, thrombocytopenia, and non‐regenerative absolute reticulocyte count for co‐infection were also calculated.

A co‐infection prevalence of 2.0% (95% CI: 0.4–5.7) with *B. rossi* and *E. canis* was found. No other co‐infections were reported. No investigated variables showed significant associations with co‐infections. Monocytosis, in particular, was not associated with co‐infection.

Co‐infection with other tick‐borne diseases in dogs with babesiosis is uncommon in the Eastern Cape province. These findings raise the possibility that *B. rossi* may have a protective effect against other tick‐borne diseases.

**Disclosures**

**Source of Funding**

The Health and Welfare Sector Education and Training Authority, and the University of Pretoria Pathobiology Research Fund and Department of Companion Animal Clinical Studies provided funding for the research. The funding sources had no involvement in the conduct of the research and/or preparation of the article.

**ECVIM CSF Funding**

No.

**ISCAID**—**International Society for Companion Animal Infectious Diseases**

## ISCAID‐P‐8

## A new licensed quadrivalent antileptospiral canine vaccine prevents mortality, clinical signs, infection, bacterial excretion, renal carriage and renal lesions caused by *Leptospira australis* experimental challenge

### J. Bouvet^1^, C. Cariou^2^, F. Oberli^1^, L. Cupillard^2^

#### ^1^Boehringer Ingelheim, Saint‐Vulbas, France; ^2^Boehringer Ingelheim, Saint‐Priest, France

The objective of the studies was to evaluate onset and duration of immunity (OOI and DOI) induced by a new licensed quadrivalent antileptospiral vaccine including four *Leptospira* components (Canicola, Icterohaemorrhagiae, Grippotyphosa, Australis) against a virulent canine strain of L. Australis recently isolated from the field. In vaccinated group (OOI: 6 animals, DOI: 7 animals), seven to 10‐week‐old puppies received 2 doses of the vaccine, 4 weeks apart. Control puppies (OOI: 6 animals, DOI: 7 animals) were not vaccinated against *Leptospira*. All animals were challenged 2 weeks (OOI) and 12 months (DOI) after the 2nd vaccine injection. Mortality, clinical signs, leptospiremia, leptospiruria, renal carriage and renal lesions were assessed after challenge. In control group, the challenge induced severe clinical signs leading to death or euthanasia of 83% of puppies and 57% of adults. Leptospiremia was detected in all animals, leptospiruria in 67% of puppies and 86% of adults, kidneys were *Leptospira* positive in 83% of puppies and 71% of adults, kidney lesions were observed in 100% of puppies and 86% of adults. A thrombocytopenia associated with increased concentrations of urea, creatinine, and aspartate aminotransferase was recorded in controls displaying severe clinical signs. In both OOI and DOI vaccinated groups, no animal had clinical signs, no *Leptospira* were detected in blood, urine, and kidney. No kidney lesions were observed. No significant changes in hematological and biochemical parameters were recorded.

We also tested, in a separated OOI study (6 animals per group), another licensed quadrivalent antileptospiral non adjuvanted vaccine including four Leptospira components (Canicola, Icterohaemorrhagiae, Grippotyphosa, Australis). In vaccinated group, seven to 9‐week‐old puppies received 2 doses of the vaccine, 4 weeks apart. Control puppies remained unvaccinated. All animals were challenged with our L. Australis isolate 3 weeks after the 2nd vaccine injection. In control group, challenge induced severe clinical signs leading to euthanasia of 57% of puppies, leptospiremia was detected in all animals, leptospiruria in 71% of puppies, kidneys were *Leptospira* positive and kidney lesions in 71% of puppies. In vaccinated group, two dogs were euthanized due to severe clinical signs. Leptospiremia was detected in all animals, and 57% of the animals had leptospiruria, positive kidneys and kidney lesions. Significant changes in hematological and biochemical parameters were also recorded.

The new licensed quadrivalent antileptospiral vaccine was shown to induce a quick and long‐lasting protection against a severe *L. australis* infectious challenge, preventing mortality, clinical signs, infection, bacterial excretion, renal lesions and renal carriage.

**Disclosures**

All authors are employees of Boehringer Ingelheim, the manufacturer of the antileptospiral vaccine tested in the studies

**Source of Funding**

**ECVIM CSF Funding**

No.

**ISCAID**—**International Society for Companion Animal Infectious Diseases**

## ISCAID‐P‐9

## Molecular survey of tick‐borne pathogens in dogs and their ticks in France

### D. Tahir^1^, V. Geolier^1^, C. Collignon^2^, S. Dupuis^2^, S. Favy^3^, T. Blondel^2^, A. Sibari^2^, A. Crippa^2^, E. Ferquel^1^, M. Varloud^2^, V. Choumet^1^

#### ^1^Institut Pasteur, Paris, France; ^2^Ceva Santé Animale, Libourne, France; ^3^Mon Véto, Rouen, France

Tick‐borne diseases (TBDs) pose significant threats to both human and animal health worldwide. In France, the incidence of tick‐borne illnesses in humans and dogs is growing, thus requiring a comprehension of the diversity of tick‐borne pathogens circulating in both canine hosts and tick vectors. In this study, we aimed to characterize the molecular diversity of tick‐borne pathogens infecting dogs and their ticks and investigate the current tick infestations in dogs in France.

Overall, 39 veterinary practices were involved in blood and tick collections from dogs from different geographical locations in France between October 2022 and December 2023. Dogs infested with one or more ticks were recruited. A consent form was filled by their owner.

Dogs were screened using a rapid test (SNAP 4Dx Plus® IDEXX) targeting 4 pathogens: *Ehrlichia* spp.*, Borrelia burgdorferi, Anaplasma* spp*., Dirofilaria immitis*. Clinical case forms were available to fill by the practitioners for all dogs. Ticks were morphologically identified and categorized according to their stage, sex, and engorgement status. DNA extracted from canine blood samples and tick specimens was subjected to PCR amplification targeting pathogens (*Borrelia burgdorferi* sensu lato., *Anaplasma* spp., *Babesia* spp, *Ehrlichia* spp) followed by sequencing and phylogenetic analysis.

A total of 82 dogs and 154 ticks were sampled. The most common tick species collected from dogs were *Dermacentor reticulatus* (35%), *I. ricinus* (26.6%), *Rhipicephalus sanguineus* (21.4%), *I. hexagonus* (7.1%). The infestation intensity varied between 1 and 15 ticks/dog. Blood engorgement of *D. reticulatus*, *I. ricinus, Rh. sanguineus* and *I. hexagonus* was observed in 48.1%, 82.9%, 54.5% and 100% of specimens, respectively. Although the tick infestation cannot be directly linked to the concomitant TBD states of the dog, it is considered as a risk factor. Four dogs were positive on the rapid test (1 *Ehrlichia*, 3 *Anaplasma* spp.). More dogs were positive when their sera were analysed by PCR for at least one of the pathogen tested. Ten dogs exhibited at least one clinical sign (e.g., hyperthermia, anorexia, and haematuria) of infection potentially linked to TBDs.

This study provided valuable insights into the epidemiology of TBDs in France and emphasizes the importance of continued surveillance of these pathogens in dogs for prevention and early treatment. Our data support the role of dogs as sentinels to assess the risk of human TBD infection in a given area and demonstrate the importance of veterinarians in this One health approach.

**Disclosures**

This study was funded by Ceva Santé Animale and performed in collaboration with the Pasteur Institute of Paris and French veterinary clinics including Mon Veto. Sophie Dupuis, Cécile Collignon, Alessia Crippa, Ambre Sibari, Thomas Blondel and Marie Varloud are employees of Ceva Santé Animale.

**Source of Funding**

Ceva Santé Animale

**ECVIM CSF Funding**

No.

**ISCAID**—**International Society for Companion Animal Infectious Diseases**

## ISCAID‐P‐10

## Detection of S gene mutation (M 1058L and feline coronavirus ribonucleic acid in the aqueous humour of three living cats with uveitis and three negative controls

### A.K. Geks^1^, H. Walter^1^, N. Steffensen^1^, H. Volk^1^, J.C. Rieder^1^, C. Busse^1^

#### ^1^University of Veterinary Medicine Hannover Department of Small Animal Medicine and Surgery, Hannover, Germany

Feline uveitis can be caused by many different etiologies and requires a complex workup to detect the underlying pathogenesis. In case of highly suspected feline infectious peritonitis (FIP), with uveitis as an only clinical finding, the final diagnosis is challenging. The examination of aqueous humor by paracentesis offers the possibility of strengthening the suspected diagnosis.

In total six cats were included in the study. All were presented with ophthalmologic disorders. In 3 cats (*n* = 2 British Shorthair, *n* = 1 European Shorthair), with a median age of 11 months, a uveitis without any body cavity effusions or other organ changes was diagnosed. Infectious diseases, such as FIV/FeLV and toxoplasmosis, were ruled out before aqueous humor was collected by paracentesis and real‐time PCR was performed targeting feline coronavirus (FCoV) RNA and mutations in the S gene. The other 3 cats (*n* = 2 European Shorthair, *n* = 1 Persian cat), with a median age of 74 months, were used as a control group.

In the 3 cats with suspected FIP infection, FCoV RNA and the mutation (M1058L) in the spike gene were detected using real‐time PCR. All 3 cats were negative for the S1060A mutation. The control group was negative for FCoV RNA, M1058L and S1060A.

This case series shows that in cats with uveitis and clinical suspicions of FIP, aqueous humor paracentesis is a pre‐mortem diagnostic which should be considered to detect FCoV‐RNA and S‐gene mutations in cats.

**Disclosures**

**Source of Funding**

**ECVIM CSF Funding**

No.

**ISCAID**—**International Society for Companion Animal Infectious Diseases**

## ISCAID‐P‐11

## Protothecosis as an emerging cause of colitis and multisystemic disease of dogs in temperate Europe: Five cases

### E. Acke^1^, N. Pantchev^2^, A. Margull^2^, M. Klingler^2^, M. Somerkoski^2^, G. Stock^2^, A. Lehmbecker^2^, S. Kimpfler^2^, G. Danneels^3^, A. Meyer^4^, K.O. Söhl^5^, N. Decaro^6^, P. Capozza^6^

#### ^1^IDEXX Laboratories Vet Med Labor GmbH IDEXX Laboratories Vet Med Labor GmbH, Kornwestheim, Germany; ^2^IDEXX Laboratories Vet Med Labor GmbH, Kornwestheim, Germany; ^3^Dierenkliniek Tussen Mark en Amer, Hooge Zwaluwe, Netherlands; ^4^Tierklinik Schwarzmann, Rankweil, Austria; ^5^Tiergesundheitszentrum Söhl GmbH Tiergesundheitszentrum Söhl GmbH, Lichtenau, Germany; ^6^Department of Veterinary Medicine University of Bari, Bari, Italy

*Prototheca* spp. are ubiquitous achlorophyllous microalgae, that occur mostly in warmer aquatic environments with high organic content and can cause opportunistic infections in humans and animals. Protothecosis in dogs with disseminated disease responds poorly to treatment, thus optimizing early diagnosis and treatment strategies could be pivotal for improving survival. In Europe, cases have mainly been reported in Mediterranean countries. This report describes protothecosis in 5 dogs from countries with temperate climate conditions, with the aim to increase awareness and understanding of this frequently fatal disease.

Protothecosis was diagnosed in 5 large‐breed dogs (one mixed breed, 3 dogs were female) aged from one to 13 years. One dog was from the Netherlands, 2 from Germany and 2 from Austria. All dogs presented with colitis (hemorrhagic in 4 dogs). Neurological examination confirmed central vestibular signs in one dog. Two dogs had a history of chronic enteropathy, the dog with vestibular signs had been receiving steroid therapy. One dog was pregnant and developed mastitis. Diagnostics included complete blood count and serum clinical chemistry; fecal sample flotation, bacterial and fungal culture, *Giardia*, *Cryptosporidium*, *Clostridia* spp., and canine Parvovirus testing; serum CRP, vitamin B12, folic acid, basal cortisol/ACTH stimulation, canine pancreatic lipase and trypsin‐like immunoreactivity. Abdominal imaging was performed, and chronic gastrointestinal signs had been treated supportively as well as with antibiotic and steroid therapy in some cases. Endoscopic intestinal biopsies were collected in 3 cases, surgical biopsies in one case, and cytology was done in the mastitis patient.

Histopathology revealed lymphoplasmacytic and eosinophilic enteritis, and granulomatous colitis with intralesional and intracellular Periodic Acid‐Schiff positive structures. Culture, Maldi‐Tof‐MS and/or microscopy, and PCR confirmed a diagnosis of *Prototheca* spp. infection. Other diagnostic workup findings were non‐specific, fitting with dehydration, inflammation, intestinal blood loss and chronic enteritis. Oral or parenteral antifungal treatment was attempted in all cases, 2 cases deteriorated with multifocal signs and were euthanized within 3 months. Rectal scraping cytology was found to be a useful and rapid non‐invasive method for screening, and monitoring of *Prototheca* spp. colitis therapy.

These cases, combined with findings from previous reports, highlight the need for recognizing protothecosis as a cause of enteritis and multisystemic disease. Most dogs present with hemorrhagic colitis and rapid, non‐invasive diagnosis via rectal scraping cytology is possible, which should enable earlier treatment with better response rates. A predisposition in purebred, female and immunocompromised dogs may exist.

**Disclosures**

**Source of Funding**

**ECVIM CSF Funding**

No.

**ISCAID**—**International Society for Companion Animal Infectious Diseases**

## ISCAID‐P‐12

## Are inflammatory biomarkers associated with bleeding diathesis in dogs with *Angiostrongylus vasorum*?

### L.N. Nielsen^1^, A.K. Krogh^1^, M. Bak^1^, C. Soendergaard^1^, R. Langhorn^1^, J. Willesen^1^

#### ^1^University of Copenhagen University Hospital for Companion Animals, Frederiksberg C, Denmark

*Angiostrongylus (A.) vasorum* is a common infection in dogs and a subgroup presents primarily with bleeding diathesis. The pathophysiology of this bleeding phenotype remains obscure; however, subclinical disseminated intravascular coagulation (DIC) has been suggested. While eosinophilia is common in dogs with *A. vasorum*, activation of other parts of the immune system could induce subclinical DIC. Larvae in the pulmonary vessels may provoke the development of neutrophil extracellular traps (NETs), a potential trigger for DIC.

The aim of this study was to examine if parts of the innate and adaptive immune system in *A. vasorum* positive dogs were associated with clinical bleeding.

This design was a retrospective cross sectional study in a single veterinary hospital examining client‐owned *A. vasorum*‐positive dogs from 2005 to 2023. Diagnosis was reached by Baermann sedimentation technique or antigen testing. Dogs were grouped as a bleeding or non‐bleeding phenotype based on clinical signs of bleeding diathesis. Difference in inflammation parameters (neutrophils, neutrophil‐to‐lymphocyte ratio (NLR), lymphocytes, eosinophils and, C‐reactive protein (CRP)) was examined between the groups. In addition, nucleic acid‐containing units (NACU), a novel biomarker of cell free DNA and RNA, was analyzed as a potential indirect biomarker of NETs and measured in a subset of dogs as part of their routine haematology. No extra material was collected from the dogs.

The study included 200 *A. vasorum‐*positive dogs, with 77 dogs presented with bleeding diathesis. Bleeding and non‐bleeding dogs differed significantly (median and interquartile range) in NLR (3.3 (1.8–4.7), 2.3 (1.5–3.5), *p* = 0.01) and eosinophils (×10^9^cells/L) (0.6 (0.1–1.0), 1.1 (0.5–1.6), *p* = 0.0002) although substantial overlap between groups was seen. Neither the neutrophils, lymphocytes, NACU count nor CRP differed significantly.

Although it was confirmed that dogs with *A. vasorum* and concurrent bleeding diathesis have inflammatory changes, these were subtle. In depth inflammatory pathways needs investigated to uncover, if subclinical DIC is caused by inflammation in these dogs.

**Disclosures**

Some of the co‐authors have had speaker engagements, and (unrelated subject matter) financial relationship with medical companies.

**Source of Funding**

The Danish Kennel Club association.

**ECVIM CSF Funding**

No.

**ISCAID**—**International Society for Companion Animal Infectious Diseases**

## ISCAID‐P‐13

## Retrospective evaluation of positive blood cultures from dogs (2014–2022)

### T. Sandbrink^1,2^, A. Lübke‐Becker ^3,4^, C. Robé ^3,5^, M. Fulde^3,4^, M. Knopf^6^, R. Merle^3,7^, C. Weingart^6^, U. Rösler^3,5^, S. Schwarz^3,4^, B. Kohn^3,6^

#### ^1^Small Animal Clinic, Freie Universität Berlin, Germany Small Animal Clinic, Freie Universität Berlin, Germany, Berlin, Germany; ^2^Veterinary Centre for Resistance Research, Freie Universität Berlin Small Animal Clinic, Freie Universität Berlin, Germany, Berlin, Germany; ^3^Veterinary Centre for Resistance Research, Freie Universität Berlin, Berlin, Germany; ^4^Institute of Microbiology and Epizootics, Freie Universität Berlin, Berlin, Germany; ^5^Institute for Animal Hygiene and Environmental Health, Freie Universität Berlin, Berlin, Germany; ^6^Small Animal Clinic, Freie Universität Berlin, Germany, Berlin, Germany; ^7^Institute for Veterinary Epidemiology and Biostatistics Freie Universität Berlin, Berlin, Germany

Blood culture (BC) results are imperative for the selection of an optimized antimicrobial treatment of patients with bloodstream infections. This evaluation of BC results aimed at identifying opportunities to optimize BC management in small animal medicine. Therefore, the most frequently isolated bacterial pathogens, their antimicrobial susceptibility profiles, the positivity and contamination rates, and durations of processing steps were determined.

Microbiology results of canine BC from 01/2014 to 9/2022 were evaluated retrospectively. Data was processed using Microsoft Excel®, IBM SPSS Statistics®, and GraphPad Prism®.

Clinically relevant bacterial pathogens were detected in 103 of the 751 BCs (13.7%). The rate of false‐positive BCs due to contamination was 1.9%. The most common bacteria from monomicrobial‐positive BCs were Enterobacterales [*Escherichia coli* (*n* = 18), *Klebsiella pneumoniae* (*n* = 2), *Enterobacter cloacae* complex (*n* = 1), *Salmonella enterica* (*n* = 1), 13 (59%) thereof multidrug‐resistant]; coagulase‐positive staphylococci [*Staphylococcus pseudintermedius* (*n* = 16), *Staphylococcus aureus* (*n* = 4), 3 (15%) thereof methicillin‐resistant], beta‐hemolytic streptococci [*Streptococcus canis* (*n* = 15), *Streptococcus equi* subsp. *zooepidemicus* (*n* = 2)] and obligate anaerobic species. Nine BCs showed polymicrobial growth. The duration of processing from receipt in the laboratory until feedback of pathogen identification was 1–6 days (median 1) and 1–7 days (median 2) until feedback of resistance profiles.

BC yield was comparable to other veterinary studies. The contamination rate is acceptable according to the performance standard in human medicine. The detection of multidrug‐resistant Enterobacterales and methicillin‐resistant coagulase‐positive staphylococci underscores the need for shorter cultivation and antimicrobial susceptibility testing durations to minimize the time to optimized antimicrobial therapy.

**Disclosures**

Teresa Sandbrink declares that she is supported by a scholarship from the Jutta und Georg Bruns‐Stiftung and the Ernst Reuter Foundation. Antina Lübke‐Beker, Marcus Fulde, Roswitha Merle, Caroline Robé, Uwe Rösler, Myriam Knopf and Stefan Schwarz declare that they have no conflict of interest. Christiane Weingart declares that she has given lectures for veterinary pharmaceutical and diagnostics companies. Barbara Kohn has held lectures, had research collaborations, and acted as a consultant for veterinary pharmaceutical and diagnostic companies.

**Source of Funding**

Teresa Sandbrink declares that she is supported by a scholarship from the Jutta und Georg Bruns‐Stiftung and the Ernst Reuter Foundation.

**ECVIM CSF Funding**

No.

**ISCAID**—**International Society for Companion Animal Infectious Diseases**

## ISCAID‐P‐14

## Evaluation of attitudes and behaviours related to antimicrobial prescribing in canine urinary tract infections using discrete choice experiments

### T. Sørensen^1^, F. Allerton^2^, S. Weese^3^, L. Jessen^1^, D. Timofte^4^, F. Bagcigil^5^, E. Broens^6^, M. Nolff^7^, D. Singleton^8^, D. Verwilghen^9^, J. Buckell^10^, K. Powels^10^

#### ^1^University of Copenhagen Dept. of Veterinary Clinical Sciences, Frederiksberg, Denmark; ^2^Willows Veterinary Centre & Referral Service, West Midlands, United Kingdom; ^3^University of Guelph Dept of Pathobiology and Centre for Public Health and Zoonoses, Guelph, Canada; ^4^University of Liverpool Department of Veterinary Anatomy, Physiology and Pathology, Liverpool, United Kingdom; ^5^Istanbul University‐Cerrahpaşa Department: Department of Microbiology, Istanbul, Turkey; ^6^Utrecht University Department of Biomolecular Health Sciences, Utrecht, Netherlands; ^7^University of Zürich Clinic for Small Animal Surgery, Zürich, Switzerland; ^8^IVC Evidensia, Bristol, United Kingdom; ^9^University of Sydney School of Veterinary Sciences, Camperdown, Australia; ^10^University of Oxford Nuffield Department of Population Health, Oxford, United Kingdom

Short duration of antimicrobial therapy for canine lower urinary tract infections (UTI) has been shown to be effective in randomized controlled trials and is believed to be associated with fewer adverse effects, less selection for antimicrobial resistance (AMR), and lower costs. The aim of this study was to investigate veterinarians’ attitudes and behaviours related to short duration antimicrobial prescribing in canine UTI.

An online discrete choice experiment (DCE) was developed in seven languages. The background questions related to the practice, attitudes and behaviours towards AMR. The experiment consisted of six experimental scenarios choosing between a 3‐day, 5‐day or 7‐day antimicrobial prescription. The scenarios were described by several varying attributes: clinical signs, adverse effects and client pressure.

In total, 853 veterinarians from 62 countries across all five World Organisation for Animal Health (WOAH) regions participated. The choice to prescribe seven days was more common than five days; and prescribing five days was more common than three days. The longer course prescription was more likely when: 1) the clinical signs were more severe; 2) the risk of adverse effects was higher; and 3) the anticipated client pressure for the longest duration of treatment. Longer course duration was also linked to veterinarians’ demographics with longer course durations prescribed if veterinarians had lower knowledge or concern for AMR, or if veterinarians were from South America compared to Europe.

The study shows that both geographic, client‐related and patient‐related factors drive antimicrobial prescribing choices and underscores the need for multifaceted stewardship interventions.

**Disclosures**

**Source of Funding**

Waltham Foundation Research Award.

**ECVIM CSF Funding**

No.

**ISCAID**—**International Society for Companion Animal Infectious Diseases**

## ISCAID‐P‐15

## The role of bone marrow parasitic load in canine leishmaniasis: Correlation/agreement with clinical status, leishvet staging system, acute phase protein and hematologic alterations at the time of diagnosis

### M. Petini^1^, T. Furlanello^2^, M. Drigo^3^, A. Zoia^1^

#### ^1^San Marco Veterinary Clinic Internal Medicine, Veggiano, Italy; ^2^Laboratorio d'Analisi San Marco Division of Clinical Pathology, Veggiano, Italy; ^3^University of Padova Department of Animal Medicine, Production and Health, Legnaro, Italy

Serological diagnosis of Canine leishmaniosis (CanL) can be difficult in endemic area. Molecular techniques such as quantitative PCR (qPCR) have high sensitivity and specificity. Bone marrow (BM) sampling is one of the most suitable substrates due to the high concentration of the protozoa in systemic disease, and BM‐qPCR can be important to elucidate the status of seropositive dogs and to monitor treatment response. The first aim of this study was: to investigate whether there is a correlation/agreement between BM‐qPCR parasitic load and clinical status score, Leishvet staging system and acute phase proteins (APPs) score in dogs with CanL at the time of diagnosis. The secondary aim was to evaluate correlation/agreement between BM parasitic load detected by qPCR and degree of haematological alterations on CBC.

Cross‐sectional study including 289 dogs with CanL presented between 2009 and 2023. All eligible dogs had serology for CanL, BM‐qPCR, and sometime direct cytological/histopathological identification of the parasite. All dogs had a complete medical record including anamnesis, physical examination, CBC, biochemistry, urinary creatinine to protein ratio, BM qPCR for Leishmania with concurrent cytological BM evaluation. Dogs treated, at any time, with anti‐leishmania drugs before diagnosis were excluded. Published clinical and APPs scores were used for this study. A scoring system based on degree of peripheral cytopenia (CBC score) was also developed. All scores were on 4 levels; therefore, also BM‐qPCR was arbitrarily categorized in 4 levels. The correlation/agreement between BM‐qPCR parasitic load, clinical score, Leishvet stage, and APPs score were assessed by Spearman's correlation test and Weighted Cohen's kappa test, respectively. The same tests were used to assess correlation/agreement between BM‐qPCR parasitic load and CBC score.

Correlation between BM‐qPCR parasitic load and clinical score, APPs score, and Leishvet stage were Rho=0.18 (*p* = 0.003), Rho=0.27 (*p*<0.001), and Rho=0.37 (*p*<0.001), respectively. Agreement between BM‐qPCR parasitic load and clinical score, APPs score, and Leishvet stage were: *k*=0.07 (95% CI, 0.03─0.11; *p*<0.001), *k*=0.21 (95% CI, 0.12─0.30; *p*<0.001), and *k*=0.24 (95% CI, 0.67─0.31; *p*<0.001), respectively. No correlation/agreement was found between BM‐qPCR parasitic load and CBC score.

Strength of correlation and agreement between BM‐qPCR parasitic load and clinical score, APPs score, and Leishvet stage, despite significant, ranged from negligible to weak and from slight to fair, while no correlation/agreement was present with CBC score. BM‐qPCR parasitic load, at diagnosis, in untreated dogs, may not be so impactful on clinical signs, acute phase response and disease severity in dogs with CanL and it is not related to haematological alterations.

**Disclosures**

**Source of Funding**

**ECVIM CSF Funding**

No.

**ISCAID**—**International Society for Companion Animal Infectious Diseases**

## ISCAID‐P‐16

## Short‐term outcome of clinical and laboratory variables in cats with feline infectious peritonitis receiving oral treatment with GS‐441524, with and without additional corticosteroids

### S. M. Meunier^1^, S. Felten^1^, A. M. Spiri^2,3^, J. M. Wenk^4,5^, C. C. De Witt Curtius^4,5^, A. B. Bouzon^6,7^, M. L. Meli^4,5^, I. Cerchiaro^4,5^, B. Pineroli^4,5^, A. K. Kipar^8^, K. Hartmann^9^, R. Hofmann‐Lehmann ^4,5^

#### ^1^Clinic for Small Animal Internal Medicine, Vetsuisse Faculty, University of Zurich, Switzerland; ^2^Clinical Laboratory, Department of Clinical Diagnostics and Services Center for Clinical Studies, University of Zurich, Switzerland; ^3^Center for Clinical Studies, Vetsuisse Faculty Center for Clinical Studies, University of Zurich, Switzerland; ^4^Clinical Laboratory, Department of Clinical Diagnostics and Services, University of Zurich, Switzerland; ^5^Center for Clinical Studies, Vetsuisse Faculty, University of Zurich, Switzerland; ^6^Clinical Laboratory, Department of Clinical Diagnostics and Services Department of Clinical Diagnostics and Services, and Center for Clinical Studies, University of Zurich, Switzerland; ^7^Center for Clinical Studies, Vetsuisse Faculty Department of Clinical Diagnostics and Services, and Center for Clinical Studies, University of Zurich, Switzerland; ^8^Institute of Veterinary Pathology, Vetsuisse Faculty, University of Zurich, Switzerland; ^9^Clinic of Small Animal Medicine, Center for Clinical Veterinary Medicine, Ludwig‐Maximilians‐Universitaet Munich, Germany

Corticosteroids are often prescribed to alleviate feline infectious peritonitis (FIP) associated neurological or immune‐mediated manifestations. Anecdotal evidence suggests caution when combining them with antivirals, but conclusive data on negative impact are lacking.

The present study aimed to determine the effect of prednisolone on survival until end of treatment, and viral loads during the first week of oral GS‐441524 treatment for FIP.

One hundred cats with effusive FIP were prospectively treated with GS‐441524 (15 mg/kg PO q24h, BOVA UK). Four already received prednisolone for small cell lymphoma (1: 2 mg/kg PO q24h), IBD (1: 0.5 mg/kg PO q24h), or unknown reason (2: 0.5 mg/kg PO q12h and 24h, respectively). Prednisolone treatment was initiated within first 5 treatment days in 10/100 cats (9: 1mg/kg and 1: 2 mg/kg PO q24h) due to neurologic signs and associative immune‐mediated hemolytic anemia, respectively. Viral loads were determined in blood and effusion (when sampling was feasible) by quantitative 7b‐Gen‐RT‐qPCR on treatment days 1 (D1) and 7 (D7). Statistical analyses were performed in GraphPad Prism® 10, using non‐parametric tests.

Survival rate until end of treatment did not differ significantly (100% (13/13) *versus* 93% (74/80), *p* = 0.31; 7 still under treatment), neither did viral loads on D1 (blood: median 5678 *versus* 392 copies/mL (*p* = 0.46); effusion: median 6,548,598 versus 8,891,000 copies/mL (*p* = 0.79)) and on D7 (blood: median 0 copies/mL, both groups (*p* = 0.34); effusion: median 1100 *versus* 200 copies/mL (*p* = 0.09)).

In conclusion, prednisolone does not impede short‐term recovery in cats receiving oral GS‐441524 FIP‐treatment and can therefore be used safely with appropriate indication.

**Disclosures**

**Source of Funding**

‐Schweizerische Vereinigung für Kleintiermedizin (SVK) ‐Stiftung für Kleintiere der Vetsuisse Fakultät Zürich ‐UZH Global Strategy and Partnerships Funding.

**ECVIM CSF Funding**

No.

**ISCAID**—**International Society for Companion Animal Infectious Diseases**

## ISCAID‐P‐17

## How low can you go? Antibiotic use in Swedish dogs with gastroenteritis

### D. Ljungquist^1^, A.M. Andersson^2^, E. Johansson^1^, J. Tham^3^, L. Toresson^4^

#### ^1^IVC Evidensia SE IVC Evidensia, Stockholm, Sweden; ^2^IVC Evidensia, Bristol, United Kingdom; ^3^Department of translational medicine, Lund University, Malmö, Sweden; ^4^Faculty of Veterinary Medicine, University of Helsinki, Helsinki, Finland

Prioritising antibiotic stewardship (ASP) efforts to areas with a high level of antibiotic use, where evidence support non‐antibiotic treatment regimens, is efficient to reduce overall antibiotic prescriptions. The European Network for Optimization of Veterinary Antimicrobial Treatment (ENOVAT) has assessed canine gastroenteritis (CGE) to be an area of special interest, with high prescription rates of antibiotics. Previous international studies have indicated high levels of antibiotic use for CGE, ranging from 40% to 71%, despite international and national antibiotic guidelines advocating rational and restrictive use.

In Sweden, half a century of efforts to decrease antibiotic use within veterinary and human care have paved the way for a low national level of antimicrobial resistance (AMR). This makes Swedish data valuable when defining international benchmarks. The aim of this study is to describe the rate of systemic antibiotic use and the temporal trend for CGE in Sweden between 2020 and 2023.

In this retrospective observational multicentre cohort study, data from 93,641 CGE consultations from a veterinary corporate group between 2020 and 2023 was analysed using Microsoft Power BI linked to the medical database. Diagnoses coded as CGE were categorised as either acute or chronic CGE. The acute CGE were further subcategorised as diarrhoetic (DCGE) or non‐diarrhoetic (NDCGE), as well as haemorrhagic or non‐haemorrhagic DCGE. All CGE consultations with or without systemic antibiotic treatment were included irrespective of age, breed, severity of disease and level of care. Local antibiotic treatments were excluded from the treatment data.

The yearly rate of antibiotic use for CGE between 2020 and 2023 was 8.1%, 6.1%, 5.0% and 3.9%, respectively, with a mean of 5.7%. Hence, a more than fifty percent reduction in antibiotic prescription was observed between 2020 and 2023. Specifying acute CGE as DCGE or NDCGE yielded a 7.6% and 3.5% average rate of antibiotic use, respectively, for the entire study period. For non‐haemorrhagic DCGE, the average rate of antibiotic use was 5.5%, whereas 21.0% of the haemorrhagic DCGE received antibiotics. For chronic CGE, the rate of antibiotic use declined from 2.4% in 2020 to 1.1% in 2023.

This study demonstrates considerably lower rates of antibiotic use for canine gastroenteritis in Sweden compared to international studies. The declining rate of antibiotic use is most likely reflecting educational and informative national efforts as well as the group's ASP strategies. The results verify prudence in antibiotic use for canine gastroenteritis amongst Swedish veterinarians and can serve as an international benchmark.

**Disclosures**

**Source of Funding**

**ECVIM CSF Funding**

No.

**ISCAID**—**International Society for Companion Animal Infectious Diseases**

## ISCAID‐P‐18

## Understanding the rationale for metronidazole use in dogs and cats

### J. Ng^1^, N. Steffensen^2^, I. Battersby^3^, J. Weese^4^, D. Timofte^5^, P. L. Toutain^6^, J. Granick^7^, J. Elliott^6^, S.Y. Choi^1^, S. Tavener^8^, F. Allerton^1^

#### ^1^Willows Veterinary Centre & Referral Service Department of Internal Medicine, Solihull, United Kingdom; ^2^University of Veterinary Medicine Hannover, Foundation Department of Internal Medicine, Hannover, Germany; ^3^Mars Veterinary Health 18101 SE 6th Way, Vancouver, Canada; ^4^Ontario Veterinary College—University of Guelph Centre for Public Health and Zoonoses, Guelph, Canada; ^5^University of Liverpool Institute of Infection, Veterinary and Ecological Sciences, Liverpool, United Kingdom; ^6^The Royal Veterinary College Department of Comparative Biomedical Sciences, London, United Kingdom; ^7^University of Minnesota College of Veterinary Medicine Department of Veterinary Clinical Sciences, Minnesota, United States; ^8^Highcliff Veterinary Practice Limited, Suffolk, United Kingdom

Metronidazole is commonly used in dogs and cats. The aim of this study was to characterise how and why metronidazole is used.

Veterinarians were invited to input data relating to animals they had treated with metronidazole in the last year into an electronic data capture platform (CastorEDC). In phase I, reasons for metronidazole selection were reported via open questioning until theoretical saturation was reached (*n* = 91). In phase II, a list of options was provided for the rationale question based on answers from phase I.

There were 137 respondents and 333 cases, reporting metronidazole use in 47 cats and 286 dogs.

Metronidazole was most often prescribed to treat acute diarrhoea (155/333, 47%), chronic diarrhoea (80/333, 24%), or giardiasis (36/333, 11%).

Veterinarians reported selecting metronidazole for non‐antibacterial reasons in 68% of cases (227/333). Of these 227 uses of metronidazole, 49 % were for a putative anti‐diarrhoeal action, 37% for anti‐inflammatory/immunomodulatory properties and 23 % to manage patients that were systemically unwell or had severe disease.

In phase II of the study, respondents that cited the anti‐inflammatory / immunomodulatory properties were asked to provide a source of this mechanism of action. Educational resources (41/92, 45%), team‐based collaboration (29/92, 32%) and specialist consultation (10/92, 11%) were most commonly referenced.

Among animals receiving metronidazole, faecal testing (82/333, 25 %), culture of non‐faecal samples (34/333, 10%), cytologic evaluation (21/333, 6%), histopathologic evaluation (19/333, 6%), polymerase chain reaction (6/333, 2%), and faecal enterotoxin assays (4/333, 1%) were performed. Respondents performed no diagnostic tests in 27% of cases (91/333).

This study suggests that veterinarians are using metronidazole predominantly for non‐antibacterial properties, and frequently in contradiction to antimicrobial use guidelines. Evidence of the anti‐inflammatory or immunomodulatory effect in dogs and cats with diarrhoea is lacking although this putative mechanism seems well‐established (without traceable backing) among veterinarians in practice. Future stewardship programs should adapt guidance specifically to counter this prescribing behaviour.

**Disclosures**

**Source of Funding**

Funding for the survey platform (CastorEDC) comes from UK Small Animal Medicine Society (SAMSoc).

**ECVIM CSF Funding**

No.

**ISCAID**—**International Society for Companion Animal Infectious Diseases**

## ISCAID‐P‐19

## Encouraging safe antibiotic disposal: A One Health initiative

### C. Prior^1^, F. Allerton^1^, C. Jamieson^2^, R. Aggarwal^3^, J. Granick^4^, D. Cooper^2^, M. Bawn^5^, I. Ramsey^6^

#### ^1^Willows Veterinary Centre and Referral Service Internal Medicine Service, Birmingham, United Kingdom; ^2^NHS England Regional Antimicrobial Stewardship Lead Midlands Region, Birmingham, United Kingdom; ^3^NHS England Medicines Management and Optimisation, Birmingham, United Kingdom; ^4^College of Veterinary Medicine, University of Minnesota Internal Medicine Service, Minnesota, United States; ^5^The Launch Box RUMA CA&E Communications Manager and Antibiotic Amnesty Project, Cheshire, United Kingdom; ^6^University of Glasgow,School of Biodiversity, One Health and Veterinary Medicine Internal Medicine Service, Glasgow, United Kingdom

The safe disposal of unused or unwanted antibiotics to prevent environmental contamination and antimicrobial resistance represents a missing link in antimicrobial stewardship efforts. A campaign, called the Antibiotic Amnesty, aimed at encouraging the return of leftover antibiotics was adopted as a One Health initiative in 2022 and 2023 to address this deficit.

During November 2022 and 2023, UK veterinary practices and local community pharmacies in the Midlands promoted the return of unused (or partially unused) packs of antibiotics for safe disposal. Messaging was shared with health professionals and the general public (especially pet owners) around the environmental impact of inappropriate disposal and the dangers of using leftover antibiotics on others (including pets). The impact of the Antibiotic Amnesty was monitored by measuring antibiotic returns and by surveying veterinary and health professionals. To gauge the outcomes of the amnesty in the veterinary sector, an on‐line practice survey was used.

Both amnesties and public education campaigns witnessed significant engagement from the veterinary community, resulting in a year on year increase in returned antibiotics. Over the two campaign periods, 369 full and 886 part‐packs of antibiotics were returned to community pharmacies. In 2023, in the veterinary arm at least 2458 antibiotic tablets were collected (a threefold increase from the previous year), 160 oral antibiotic suspensions (with no comparative data from the previous year), 119 topical antibiotic preparations (more than double the previous year), and 11 antibiotic injections (a slight decrease from the previous year). Among these returns were highest priority critically important antibiotics (fluoroquinolones). Furthermore, 302 practices and veterinary organizations participated, representing a 70% increase from the previous year. In contrast, decreased engagement was observed across the human sector, attributed to factors such as campaign repetition, workforce issues in community pharmacy, and financial constraints impeding capacity for unfunded public health initiatives.

The amnesty represents a low input (resources readily available), high impact initiative that can support a One Health collaboration between different professions with particular potential in the veterinary sector. This study underscores the multifaceted approach required to address AMR effectively, emphasising the importance of collaborative efforts and targeted communication strategies in promoting responsible antibiotic use and disposal practices.

**Disclosures**

**Source of Funding**

None.

**ECVIM CSF Funding**

No.

**ISCAID**—**International Society for Companion Animal Infectious Diseases**

## ISCAID‐P‐20

## Antimicrobial susceptibility patterns in hepatobiliary bacterial infections in dogs and cats: A retrospective study about 343 isolates from 2010 to 2019

### A. Schlachet^1^, S. Beurlet‐Lafarge ^2^, H.J. Boulouis^3^, M. Canonne‐Guibert ^4^

#### ^1^Department of Clinical Veterinary Science, Vetsuisse Faculty, University of Bern Division of Small Animal Internal Medicine, Bern, Switzerland; ^2^Cerbavet Laboratory, Massy, France; ^3^École nationale vétérinaire d'Alfort, Université Est Créteil Parasitic and Fungal Molecular Biology and Immunology Unit, Maisons‐Alfort, France; ^4^École nationale vétérinaire d'Alfort, Université Est Créteil Department of Small Animal Internal Medicine, Maisons‐Alfort, France

Hepatobiliary infections are not uncommon in dogs and cats, and the widespread availability and improvement of diagnostic tools have led to increased recognition of these diseases. Antibiotic treatment is classically prescribed for an extended period of 4–6 weeks and choosing the right antimicrobial drug is crucial. However, recent description of antibiotic susceptibility of hepatobiliary pathogens from dogs and cats is lacking. The aim of this study was to describe antibiotic susceptibilities of hepatobiliary bacteria collected from dogs and cats over a 10 year‐period.

A total of 343 bacterial pathogens isolated on bile and liver tissue and collected from 309 dogs and cats were retrospectively recorded. Cultures were analyzed by a Veterinary Teaching Hospital and an additional private laboratory. Prevalence of multidrug resistant (MDR) bacteria and association with prior antibiotic administration were evaluated.

Monobacterial cultures were more frequent (91%). Gram‐negative bacteria were predominant (67%), with *Escherichia coli*, *Staphylococcus* spp., and *Enterococcus* spp. being the most common pathogens.

Resistance of gram‐negative bacteria to amoxicillin‐clavulanic acid, aminopenicillins and fluoroquinolones was respectively observed for 32%, 48% and 16% of isolates. Resistance of gram‐positive bacteria to amoxicillin‐clavulanic acid, aminopenicillins and fluoroquinolones was respectively observed for 12%, 16% and 17% of isolates.

During the period from 2010 to 2014, pathogens exhibited higher rates of resistance to aminopenicillins (*p* = .004) and first‐generation cephalosporins (*p*<.001) compared to the subsequent period of 2015 to 2019. Forty percent of isolated bacteria were MDR. One‐third of *E.coli* isolates and half of *Enterococcus* spp. isolates were MDR. MDR bacteria were isolated more frequently in dogs and cats that received antibiotic treatment in the three months prior to sampling (81%, *p*<.001).

This study represents the largest examination of antibiotic susceptibilities among hepatobiliary bacteria in dogs and cats. A worrying proportion of MDR bacteria was observed, particularly in animals that had received antibiotic therapy within the 3 months preceding the infection. Performing culture and antimicrobial testing on bile or liver tissue is thus highly warranted in dogs and cats suspected to have hepatobiliary infection.

**Disclosures**

**Source of Funding**

No funding source.

**ECVIM CSF Funding**

No.

**ESVIM**—**European Society of Veterinary Internal Medicine**

## ESVIM‐P‐1

## Serum total IgE levels in clinical feline patients with asthma, chronic bronchitis, and mixed‐type inflammatory lower airway disease

### F.Y. Lin^1^, W.T. Chang^1^, H.W. Chen^2^, H.F. Chuang^3^, Y.Y. Chen^3^, P.Y. Lo^4^, H.D. Wu^5^, C.H. Lin^6^

#### ^1^National Taiwan University, TACS‐Alliance Research Center Lab of Small Animal Respiratory and Cardiovascular Medicine, Taipei, Taiwan; ^2^National Taiwan University Department of Veterinary Medicine, Animal Resource Center, Taipei, Taiwan; ^3^National Taiwan University Department of Veterinary Medicine, Taipei, Taiwan; ^4^TACS‐Alliance Research Center Lab of Small Animal Respiratory and Cardiovascular Medicine, Taipei, Taiwan; ^5^National Taiwan University National Taiwan University Hospital, Taipei, Taiwan; ^6^National Taiwan University, TACS‐Alliance Research Center VH & Graduate Institute Vet Clin Sci; Lab of Small Anim Resp & CV Medicine, Taipei, Taiwan

Feline lower airway disease (FLAD) comprises asthma, chronic bronchitis (CB), and mixed inflammation (MI), differentiated by bronchoalveolar lavage (BAL) analysis. Although asthma is conventionally defined by BAL eosinophilic inflammation and believed to be an allergic disease, allergen‐specific IgE is not always detectable. Considering total IgE concentration in blood (T‐IgE) has shown relevance in asthma phenotyping in humans, this study aimed to investigate T‐IgE levels in FLAD patients.

Inclusion criteria included a definitive diagnosis of asthma, CB, or MI by BAL, availability of frozen leftover serum obtained without corticosteroid use within 4 weeks, and absence of identifiable parasite infestation. Feline‐specific T‐IgE was measured by ELISA and compared with BAL cytology and clinical data.

A total of 53 cats (32 asthma, 7 CB, 14 MI; median age 5.2 years, range 1.3–15.5) were enrolled. T‐IgE ranged from 0.0 to 522.8 μg/mL in study cats. Cats with T‐IgE below the first quartile exhibited milder inflammation (*P* = 0.045), but BAL eosinophil percentage showed no correlation with T‐IgE (r_s_=0.07). Furthermore, median T‐IgE (μg/mL) was not statistically different among cats with asthma (15.8; range 0.0–522.8), CB (13.1; 0.0–36.1), and MI (42.2; 0.0–294.8) (*P* = 0.12). Cats with bronchospasm or profound yet reversible bronchoconstriction exhibited significantly higher T‐IgE levels than cats without (91.7 vs. 14.4 μg/mL, *P* = 0.01).

In conclusion, T‐IgE was not specifically high in cats with BAL eosinophilic inflammation, suggesting heterogeneous immune responses among BAL inflammatory subtypes in FLAD. Cats with bronchospasm‐related features appear to have higher T‐IgE levels, indicating a need for future investigations.

**Disclosures**

**Source of Funding**

NSTC 111‐2313‐B‐002‐062–/TACS‐A 2024 Award/Support from National Taiwan University.

**ECVIM CSF Funding**

No.

**ESVIM**—**European Society of Veterinary Internal Medicine**

## ESVIM‐P‐2

## Bronchoalveolar lavage fluid hyaluronan in dogs with bronchial diseases

### S. Viitanen^1^, N. Höglund^2^, A.M. Mustonen^3^, S. Oikari^4^, P. Nieminen^4^

#### ^1^University of Helsinki Department of Equine and Small Animal Medicine, Helsinki, Finland; ^2^University of Helsinki Department of Equine and Small Animal Medicine, Helsinki, Finland; ^3^University of Eastern Finland Department of Environmental and Biological Sciences, Faculty of Science, Kuopio, Finland; ^4^University of Eastern Finland Institute of Biomedicine, School of Medicine, Kuopio, Finland

Hyaluronan (HA) is a non‐sulfated glycosaminoglycan, which is major component of extracellular matrix. During lung injury, low‐molecular‐weight HA is produced and, contrary to physiological HA, it exhibits pro‐inflammatory properties. Deposition of HA has been documented in several lung diseases in humans and experimental animal models, but role of HA in canine lung diseases is yet undiscovered. Our hypothesis was that bronchoalveolar lavage fluid (BALF) HA concentrations would be modified in canine inflammatory bronchial diseases.

Surplus samples from dogs diagnosed with chronic bronchitis (CB, *n* = 12) and eosinophilic bronchopneumopathy (EBP, *n* = 13), as well as from healthy control dogs (*n* = 11) were included. Diagnosis was based on thoracic imaging, bronchoscopy and BALF cytology and culture. HA was measured from BALF samples using a sandwich‐like ELISA. HA concentrations between groups were compared with Kruskal–Wallis test and correlations were assessed using Spearman's correlation coefficients.

BALF HA was significantly (*P*<0.001) increased in dogs with EBP (median 42.9 ng/ml, interquartile range [IQR] 31.4–186.7 ng/ml) when compared to dogs with CB (median 6.2 ng/ml, IQR 3.4–16.6 ng/ml) or healthy dogs (median 3.9 ng/ml, IQR 3.2–5.6 ng/ml). HA was positively correlated to both blood eosinophil count (*P* = 0.002, *r* = 0.451) and BALF eosinophil percentage (*P* = 0.001, *r* = 0.471).

These results show that HA concentration in BALF increases in canine eosinophilic lung disease, similarly, as reported in human allergic asthma. This indicates that HA has a role in EBP pathophysiology and allows further studies investigating the potential of HA as a diagnostic biomarker or as a therapeutic target.

**Disclosures**

SJ Viitanen has received speaking honoraria from Fennovet Oy, Evidensia Oy, Royal Canine, IDEXX and Dechra Finland and travel support from the Finnish Foundation of Veterinary Research. None of these have influenced study design or interpretation of the results in this study.

**Source of Funding**

This study was supported by a grant from the Research Council of Finland (grant number 338469).

**ECVIM CSF Funding**

No.

**ESVIM**—**European Society of Veterinary Internal Medicine**

## ESVIM‐P‐3

## Silicone stent placement for laryngeal paralysis and other laryngeal diseases: A retrospective study of 13 cases

### M. David^1^, L. Giraud^1^, R. Dumont^1^, S. Gibert^2^, A. Dunié‐Mérigot^2^, P. Sériot^2^, F. Jolivet^1^

#### ^1^CHV Languedocia Internal Medicine, Montpellier, France; ^2^CHV Languedocia Surgery, Montpellier, France

In dogs with idiopathic laryngeal paralysis, surgical treatment with unilateral arytenoid lateralization remains the gold standard, but can be declined for financial concerns and its invasive nature. Laryngeal silicone stent placement for laryngeal paralysis has recently been described in two small case series. Data regarding the use of this device in other laryngeal diseases are scarce.

The aim of this study was to evaluate the outcome of silicone stents placement for laryngeal paralysis and other laryngeal diseases in dogs.

This retrospective study included dogs treated with a laryngeal silicone stent in our veterinary hospital between September 2022 and March 2024. Complete blood count, biochemistry panel, thoracic imaging (radiography or computed tomography) and laryngoscopy were required for inclusion. Response to treatment, complications and survival time were recorded.

Thirteen dogs were included, 9 with laryngeal paralysis, 1 with laryngeal collapse, 1 with a laryngeal granuloma and 2 with both laryngeal collapse and granulomas. Inspiratory dyspnea (*n* = 10), stridor (*n* = 7), stertor (*n* = 5) and exercise intolerance (*n* = 5) were the main clinical signs at presentation. Median duration of clinical signs before presentation was 4 months [10–912 days]. Complete resolution of clinical signs was observed for all dogs before discharge. After stent placement, clinical and radiological findings consistent with aspiration pneumonia were reported in 3 dogs. Two dogs underwent definitive stent removal after 3 weeks due to recurrent aspiration pneumonia or bacterial infection of the device associated with severe cough and retching. Six dogs died within the study period, including 2 dogs with laryngeal paralysis and all 4 dogs without laryngeal paralysis. Dogs with laryngeal paralysis died from causes unrelated to stent placement. In the others, reported causes of death were obstruction of the stent due to granulomas (*n* = 2) at 45 and 419 days following its placement, aspiration pneumonia (*n* = 1) and respiratory distress in a dog with concomitant nasal carcinoma. The remaining 5 dogs are still alive at the time of writing, from 77 to 428 days after the procedure. Their quality of life is deemed good by their owners. Reported side effects include mild cough (*n* = 4), halitosis (*n* = 4), retching (*n* = 1) and mild dysphagia (*n* = 1) in these dogs.

Laryngeal silicone stent placement appears well tolerated and offers an alternative for treatment of idiopathic laryngeal paralysis in dogs. The procedure is quick, cost‐efficient and noninvasive. However, complication rates seem higher in other laryngeal diseases, for which stenting might not be indicated.

**Disclosures**

**Source of Funding**

**ECVIM CSF Funding**

No.

**ESVIM**—**European Society of Veterinary Internal Medicine**

## ESVIM‐P‐4

## Investigating the presence of gastroesophageal reflux disease in dogs with chronic rhinitis

### T. Lee‐Fowler ^1^, M. Grobman^1^

#### ^1^Auburn University CVM, Auburn, AL, United States

A link between gastroesophageal reflux disease (GERD) and chronic rhinitis has been established in humans. Though suspected in dogs, this has not been thoroughly investigated. This retrospective study aimed to evaluate the prevalence of GERD in a population of dogs with chronic rhinitis.

The database was searched for dogs diagnosed with nasal disease or GERD between August 2021 and March 2024. Dogs with nasal diseases other than rhinitis were excluded. GERD diagnosis was made either by video fluoroscopic swallow study or resolution of clinical signs with GERD therapy. Included dogs were categorized into GERD and non‐GERD groups. Age, sex, weight, BCS, respiratory rate (RR), duration of clinical signs (DCS), conformation, clinical signs (sneezing, reverse sneezing, coughing, gagging, epistaxis, nasal discharge [ND]), and character of the ND, serous or mucopurulent (MP), were compared between groups. Statistical analysis was completed using *t*‐test, Mann–Whitney rank sum, and Fisher's exact test, respective to the distribution of the data (*p*<0.05).

One hundred and nineteen cases were identified. Eighty‐four dogs were excluded: mass (*n* = 67), fungal (*n* = 8), foreign body (*n* = 6), oronasal fistula (*n* = 3). Thirty‐five dogs were further evaluated and categorized as: lymphoplasmacytic (LP) rhinitis (*n* = 20), GERD (*n* = 10), neutrophilic rhinitis (*n* = 6), eosinophilic rhinitis (*n* = 2), open diagnosis (*n* = 2), and histiocytic rhinitis (*n* = 1). Two of the GERD dogs had concurrent LP rhinitis (5.7% of dogs evaluated). GERD dogs weighed less than non‐GERD dogs (*p*<0.001). No difference between groups was identified for the following: Age (*p* = 0.987), sex (*p* = 0.794), BCS (*p* = 0.712), RR (*n* = 0.228), DCS (*p* = 0.317), conformation (*p* = 0.479), sneezing (*p* = 0.434), reverse sneezing (*p* = 1), coughing (*p* = 0.434), gagging (*p* = 1), epistaxis (*p* = 0.292), ND (*p* = 0.053), serous ND (*p* = 1), MP ND (*p* = 0.686).

Chronic rhinitis and GERD may overlap in a subpopulation of dogs with nasal disease. Targeted questioning regarding GERD symptoms in dogs with chronic rhinits is warranted.

**Disclosures**

**Source of Funding**

no funding was required.

**ECVIM CSF Funding**

No.

**ESVIM**—**European Society of Veterinary Internal Medicine**

## ESVIM‐P‐5

## A retrospective study on the effect of packed red blood cell transfusion requirements on long term prognosis in dogs with non‐associative immune‐mediated hemolytic anemia

### R. Vlassenbroek^1^, D. Peeters^1^, L. Matthewman^1^, G. Bolen^1^, J. Ficheroulle^1^, K. Gommeren^1^

#### ^1^University of Liège Department of Companion Animal Clinical Sciences, Liège, Belgium

Immune‐mediated hemolytic anemia (IMHA) is common in dogs and according to the ACVIM consensus guidelines, is described as associative (aIMHA) or non‐associative (naIMHA) depending on whether an underlying cause is identified or not. IMHA patients often require blood transfusions during the initial treatment phase, which can be subject to financial constraints. Patients with more severe hemolysis or patients responding more slowly to therapy are expected to require more packed red blood cell (pRBC) transfusions during initial hospitalization but associations between pRBC transfusion requirements and long‐term prognosis of naIMHA in dogs surviving to discharge has not been investigated.

This study aimed to investigate whether there is an association between pRBC transfusion requirements and the chance of being alive in hospital discharged dogs with naIMHA.

Medical records from dogs presented to a university teaching hospital between 2017 and 2023, and diagnosed with IMHA, were retrospectively reviewed. Two blinded board‐certified internal medicine specialists individually reviewed records and classified patients as aIMHA or naIMHA. A third internist reviewed the case if the classification was unequivocal. For patients with a diagnosis of naIMHA that survived to discharge and had a minimum follow‐up of 3 months, the chances of being alive were assessed at 3, 6, and 12 months and the number of pRBC transfusions administered to each dog was recorded. Multivariable logistic regression was applied to identify a possible association between the number of pRBC transfusions requirements and the chance of being alive.

Seventy‐three dogs discharged with naIMHA and at least 3 months of follow‐up were identified. Further follow‐up was available for 67 and 59 dogs, at 6 and 12 months, respectively. The chances of being alive were 50.7%, 43.3% and 30.5% at 3, 6 and 12 months, respectively. Dogs received a median of 2 (range 0–4) pRBC transfusions. For each additional pRBC transfusion the chance of being alive at 3 months decreased by 56% (adjusted Odds Ratio (aOR), 0.44; 95% confidence interval (CI), 0.19–0.87). However, additional pRBC transfusions did not affect the chance of being alive at 6 and 12 months.

In conclusion, increased pRBC transfusion requirements were associated with a decreased chance of being alive at 3 months but not at the later time points. This may suggest that animals with higher transfusion requirements may benefit from closer monitoring and follow‐up during these first 3 months to ensure proper disease control.

**Disclosures**

None.

**Source of Funding**

None.

**ECVIM CSF Funding**

No.

**ESVIM**—**European Society of Veterinary Internal Medicine**

## ESVIM‐P‐6

## What is the diagnostic yield and clinical impact of diagnostic imaging in dogs diagnosed with immune‐mediated hemolytic anemia in Belgium: A retrospective study

### R. Vlassenbroek^1^, D. Peeters^1^, L. Matthewman^1^, G. Bolen^1^, J. Ficheroulle^1^, K. Gommeren^1^

#### ^1^University of Liège Department of Companion Animal Clinical Sciences, Liège, Belgium

Immune‐mediated hemolytic anemia (IMHA) is common in dogs and according to the ACVIM consensus guidelines is described as associative (aIMHA) or non‐associative (naIMHA) depending on whether an underlying cause is identified. The utility of diagnostic imaging (DI: abdominal ultrasound and thoracic radiographs) to screen for underlying causes has been questioned.

This study aimed to report the prevalence of aIMHA in dogs as well as the outcome of suspected naIMHA in dogs with or without DI.

Medical records from dogs, presented to a university teaching hospital between 2017 and 2023, and diagnosed with IMHA were retrospectively reviewed. Two blinded board‐certified internal medicine specialists individually reviewed records and classified patients as aIMHA or naIMHA. If classification was unequivocal, a third internist reviewed the case. For dogs with naIMHA and a minimum follow‐up period of 3 months, the chances of being alive were assessed at 3, 6, and 12 months. These dogs were further divided into a first group that received ultrasound and radiographs (DI) and a second group that either received partial (ultrasound or radiographs) or absolutely no DI (noDI). Multivariable logistic regression was applied to compare groups.

One hundred and twenty‐two dogs with IMHA were identified. The reviewers unanimously classified 7 (5.7%) as aIMHA and 115 (94.3%) as naIMHA. Of the dogs with naIMHA, Shih Tzus were overrepresented (*n* = 15, 13.0%), and follow‐up was available for 73 (DI = 27; noDI = 46), 67 (DI = 23; noDI = 44) and 59 (DI = 20; noDI = 39) dogs, at 3, 6 and 12 months, respectively. The chances of being alive were 50.7%, 43.3% and 30.5% at 3, 6 and 12 months, respectively. DI did not significantly affect the chance of being alive at 3, 6 and 12 months (*p* = 0.682, 0.554, 0.522, respectively).

In conclusion, Shih Tzus were overrepresented in this study. Performing DI in dogs with naIMHA did not affect the chance of being alive in this cohort of dogs in Belgium. A diagnosis of aIMHA was uncommon in this cohort, which may be due to regional differences.

**Disclosures**

None.

**Source of Funding**

None.

**ECVIM CSF Funding**

No.

**ESVIM**—**European Society of Veterinary Internal Medicine**

## ESVIM‐P‐7

## Prevelance of transmissible feline blood pathogens in a blood donor population tested on every donation

### R.M. Pimenta da Silva^1^, R. Ferreira^1^, I. Mesa Sanchez^2^, I. Cardoso^1^, L. Rocha^1^, S. Alves^1^, B. Correira^1^

#### ^1^Banco de Sangue animal, Porto, Portugal; ^2^Banco de Sangre animal, Barcelona, Spain

This study aimed to evaluate the prevalence of blood infectious agents in healthy, client‐owned cats from a blood donor population in Portugal and Spain, and to address the importance of a screening protocol on every donation, instead of annual testing, as is commonly performed in veterinary medicine.

Client‐owned healthy cats were tested before each donation on a veterinary blood bank. A FIV‐FeLV rapid test (WITNESS, Zoetis or SNAP Combo Test, IDEXX) was used before the first donation for quick screening, and positive cats were automatically excluded from the donor program. After each donation blood samples were tested by real time PCR (LightCycler 480ll, Roche) for *Mycoplasma spp*., provirus *FeLV (Feline Leukemia Virus) and Bartonella henselae*. Serological tests by ELISA were also performed for *FIV* and *FeLV p27 antigen* (Gemini Stratec, Novatec). On each donation cats were tested for every agent. Some donors donated more than once. Animals tested positive for PCR and/or ELISA were excluded from the donor program.

7,040 cats were tested between January 2022 and January 2024. A total of 7,040 FIV‐FeLV rapid tests revealed 0.86% (*n* = 61/7040) positive FIV results and 1.10% (77/7040) FeLV. A total of 18528 donations were carried out by the 6902 donors who were negative to the rapid test. By PCR, the overall prevalence of subclinical infectious agents in FeLV and FIV rapid test‐negative donors was 8.02% (554/6902); 5.56% (384/6902) tested positive for *Mycoplasma spp*.; 2.32% (160/6902) for *FeLV* provirus, and 0.14% (10/6902) for *Bartonella henselae*. ELISA serologies revealed positive results in 1.34% (93/6902) of donations: 0.62% (43/6902) to *FIV* antibodies, and 0.72% (50/6902) to *FeLV* p27 antigens. A total of 174 of positive results were from cats previously integrated in the blood donor program with negative results in previous donations less than a year before: 79 positive results for *Mycoplasma spp*., 73 for *FeLV*, 21 for FIV, and 1 for *Bartonella henselae*.

Obtained results suggest a relatively high prevalence of infectious agents in healthy, client‐owned feline blood donors in the Iberian Peninsula. This study emphasises the importance of a regular screening protocol for every donation instead of annual testing, as is commonly performed in veterinary medicine.

**Disclosures**

**Source of Funding**

**ECVIM CSF Funding**

No.

**ESVIM**—**European Society of Veterinary Internal Medicine**

## ESVIM‐P‐8

## Lipidomic profiles of canine leukoreducted packed red blood units during storage

### A. Miglio^1^, H. Alabed^2^, D. Frongia Mancini^2^, R. Pellegrino^2^, L. Leonardi^3^, O. Barbato^3^, M. Antognoni^3^

#### ^1^Department of Veterinary Medicine, University of Perugia, Italy Dept. Veterinary Medicine, University of Perugia, Via San Costanzo 4, 06126‐Per, Perugia, Italy; ^2^Dept. Chemistry, Biology and Biotechnology, University of Perugia, Perugia, Italy; ^3^Department of Veterinary Medicine, University of Perugia, Italy, Perugia, Italy

The application of omics technology in human medicine has improved the understanding of red blood cell (RBC) storage lesions (SLs) of blood components for transfusion use, but no studies have extensively characterized the SLs with lipidomic analysis in dogs.

The study aimed to evaluate the lipidomic profiles in canine units of leukoreduced (LR) and non‐leukoreduced (nLR) packed RBC (pRBC) during storage. Packed RBC were obtained from 6 adult blood‐donor dogs.

Whole blood was collected using CPD‐SAGM‐transfusion bags with a Leukoreduction filter to produce 2 pRBC for each donor, before (nLR‐pRBC) and after (LR‐pRBC) leukoreduction, and stored at 4°C. Sterile sampling from each pRBC unit was obtained during storage (days 0, 7, 14, 21, 28, 35, and 42), and centrifuged to obtain RBCs pellet for untargeted lipidomic analyses (Liquid Chromatography/Mass Spectrometry). Statistical analyses were performed at the level of Lipid Classes, Lipid Molecular Species and Lipid Building Blocks using the web tool LipidOne 2.0.

In both nLR and LR groups, 156 lipid molecular species belonging to 16 lipid classes were annotated. The most abundant were glycerophospholipids (particularly Diacylglycerophosphoinositols‐PI and 1‐alkyl,2‐acylglycerophosphoinositols‐PI‐O), sphingomyelins (SM), and Glycosphingolipid Sulfates (SHexCer).

Fresh pRBC samples (day 0) showed a significant quantitative diversity between nLR and LR groups due to the glycerophospholipid classes. Indeed, 50/156 lipid molecular species were significantly increased in LR‐pRBC (*p* < 0.001).

During storage time, PI and PI‐O tended to constantly increase over time in both groups nLR‐pRBC and LR‐pRBC (p< 0.001). Conversely, SHexCer lipids significantly decrease, but only in LR‐pRBC.

The explored lipidomic changes induced by storage time and LR in blood units for transfusion purposes may serve as targets for storage strategies. LR has an impact to the lipid profile of blood units that differs quantitatively but not qualitatively for most lipid classes both in nLR‐pRBC and LR‐pRBC samples.

During storage, the constant increase observed for the lipid classes PI and PI‐O could be explained as possible hydrolysis of Phosphatidylinositol (3,4,5)‐trisphosphate‐PIP3 or Phosphatidylinositol (3,4)‐Disphosphate‐PIP2 to glycerophosphoinositols. The increase of these lipids could be used as biomarkers of SLs and aging of pRBC units related to anerobic conditions. Interestingly, the decrease of SHexCer only in the LR‐pRBC group could be attributable to the absence of leukocytes in these units. Further studies focusing on RBC morphology and extracellular vesiculation during storage‐time, by using scanning electron microscopy, could be useful to improve knowledge on this matter.

**Disclosures**

**Source of Funding**

None.

**ECVIM CSF Funding**

No.

**ESVIM**—**European Society of Veterinary Internal Medicine**

## ESVIM‐P‐9

## Systemic atherosclerosis in eurasier with presumed primary hypercholesterolemia

### M. Menard^1^, R. Lavoué ^2^, O. Dossin^3^, A. Duclos^4^, I. Testault^5^, L. Giraud^6^, J. Gallay^7^, N. Gaide^8^, C. Laprie^9^, N. Soetart^10^, C. Trumel^11^, S. Piazza^12^, M. Debreuque^4^, C. Musso^13^, L. Blond^6^, F. Jolivet^14^

#### ^1^Clinique Vétérinaire Olliolis Internal Medicine Department, Ollioules, France; ^2^Ecole Nationale Vétérinaire de Toulouse Internal Medicine Department, Toulouse, France; ^3^Centre Hospitalier Vétérinaire Advetia Internal Medicine Department, Vélizy‐Villacoublay, France; ^4^Ecole Nationale Vétérinaire de Toulouse, Toulouse, France; ^5^Centre Hospitalier Vétérinaire Atlantia, Nantes, France; ^6^Centre Hospitalier Vétérinaire Languedocia, Montpellier, France; ^7^Clinique Vétérinaire Olliolis, Ollioules, France; ^8^Ecole Nationale Vétérinaire de Toulouse Anatomic Pathology Department, Toulouse, France; ^9^VetHisto, Marseille, France; ^10^LabOniris Clinical Pathology, Nantes, France; ^11^Ecole Nationale Vétérinaire de Toulouse Clinical Pathology Department, Toulouse, France; ^12^Centre Hospitalier Vétérinaire Languedocia Department of Neurology, Montpellier, France; ^13^Clinique Vétérinaire Olliolis Department of Neurology, Ollioules, France; ^14^Centre Hospitalier Vétérinaire Languedocia Internal Medicine Department, Montpellier, France

Primary hypercholesterolemia is a rare disorder in dogs. We describe here seven Eurasier including 6 clinical patients and one dog with no clinical signs (a sibling of one of the affected patients). These dogs (4 males, 3 females) were median age of 23 months (range: 19–38 months) and were presented in 5 different referral centers in France. These dogs presented with neurological signs (5/6) (central vestibular (2/5) or forebrain (3/5) signs), weight loss (5/6), decreased appetite (4/6), sneezing without nasal discharge (3/6), vomiting and/or diarrhea (3/6), lipid keratopathy (2/6), and diffuse pain (2/6). Biochemistry profile revealed severe hypercholesterolemia in 7/7 (median 26 mmol/L; range: 20–38 mmol/L) and mild to moderate hypertriglyceridemia in 6/7 (median 1.95 mmol/L; range: 1.16–3.74 mmol/L). The remaining of the bloodwork and urinalysis including screening for endocrine disease, pancreatitis, and kidney disease were unremarkable. Central nervous system imaging (MRI performed in 2/6 and CT‐Scan in 1/6) revealed no abnormality. Serum lipoprotein electrophoresis was performed in 3/7 cases and showed marked increase in percentage of low‐density lipoprotein (63.2%–75.1%). Treatment included anticonvulsant drugs and/or low‐fat diet with omega‐3 supplementation and lipid‐lowering drug (bezafibrate, atorvastatin and/or niacin). Despite treatment, 5/6 symptomatic dogs died within a median of 11 months (range: 1‐25 months) after diagnosis (1/5 sudden death and 4/5 euthanasia). Post‐mortem examination was performed in 3 cases. Gross lesions were restricted to the vascular system including at least aorta, coronary arteries, and cerebral arterial circle. Wall of the arteries appeared diffusely white–yellow, thickened, firm, with severe reduction of luminal diameter sometimes leading to complete luminal obstruction. Histopathology confirmed severe, multicentric, and chronic atherosclerosis.

In these cases, no predisposing diseases were found and therefore hyperlipidemia was considered to be primary. Considering the close genetic background of these dogs, a hereditary disorder was suspected. A causal relationship between hypercholesterolemia and clinical signs was difficult to confirm before post‐mortem examination. Serum lipoprotein electrophoresis was useful by showing high percentage of LDL, which is an uncommon lipoprotein profile in dogs and a well‐known predisposing factor of atherosclerosis in people. To our knowledge, this is the first report of systemic atherosclerosis due to presumed primary hypercholesterolemia in Eurasier dogs.

**Disclosures**

**Source of Funding**

**ECVIM CSF Funding**

No.

**ESVIM**—**European Society of Veterinary Internal Medicine**

## ESVIM‐P‐10

## Restrospective study of 65 dogs with polyarthritis in a Mediterranean area

### G. Paula^1^, T. Maria Dolores^2^, T. Javier^1^

#### ^1^Anicura San Vicente Hospital Veterinario, San Vicente‐Alicante, Spain; ^2^Anicura San Vicente Hospital Veterinario Internal Medicine, San Vicente‐Alicante, Spain

Idiopathic primary immune‐mediated polyarthritis (IMPA) is the most common cause of canine polyarthritis. Recently imaging screening has been shown to have low yield in identifying a potential cause of associative polyarthritis. However, the same may not be true regarding utility of screening for infectious disease.

The aim of this study was to retrospectively review clinical records from dogs with polyarthritis seen in a Mediterranean area. Degenerative and septic causes of arthritis were excluded.

65 dogs were evaluated (32 males and 33 females, 3 months to 13 years). All dogs have one or more manifestations of joint disease including lameness (50.8%), articular pain (70.8%), reluctance walking (27.7%) and stiffness (12.3%). Other clinical signs were fever (47.7%), spinal pain (27.7%), digestive (23.1%), PU/PD (7.7%), lymphadenomegaly (24.6%), skin alterations (15.4%) and non‐specific (73.8%‐lethargy, weakness, hyporexia, weight loss‐). Analytical alterations included anemia (60%), leukocytosis (38.5%), leukopenia (3.1%), thrombocytopenia (16.9%), azotemia (7.7%), elevated liver enzymes (13.8%), hypoalbuminemia (12.3%), hyperglobulinemia (20%) and ANA positive titer (2/29). Thorax radiographs were abnormal in 4/30, abdominal ultrasound in 24/33 and echocardiography in 1/10 dogs. Erosive arthropathy was detected in 5/37 dogs with joint radiographs. Infectious work‐up consisted on L.infantum

serology (positive in 20/62) or PCR (positive in 4/7), 4DX Idexx test (positive in 2/40, Ehrlichia canis/ewingii and Borrelia burgdorferi), bone marrow/spleen/lymph‐node cytology (Leishmania spp. positive in 13/34), Babesia spp. PCR (4 negative) and Bartonella PCR (positive for B koehlerae in 1/2).

Dogs were classified as primary (50.8%, including 3 “Shar‐pei fever” and 30 IMPA), reactive polyarthritis (46.2%, including 28 Type II PA and 2 Type IV PA) and unknown (2 dogs). Causes of Type‐II PA were leishmaniosis (20), ehrlichiosis (1), Bartonella‐endocarditis (1) and doxycycline‐response PA (6). Type IV PA was secondary to splenic carcinoma (1) and osteosarcoma (1).

Signalment, clinical signs and analytical alterations were not related to type of PA, except for leukocytosis, most likely in dogs with primary PA (*P* = 0.011).

Among dogs with known long‐term outcome, complete remission was attained in 12/27 primary PA dogs and 15/24 secondary PA dogs, partial remission in 13/27 primary PA dogs and 2/7 secondary PA dogs, and bad response was observed in 2/27 primary PA dogs and 7/24 secondary PA dogs.

In this study secondary PA was more common than previously reported and leishmaniosis was the most frequent disease encountered, highlighting the need for infectious diseases investigation in dogs with polyarthritis living in a Mediterranean area.

**Disclosures**

**Source of Funding**

**ECVIM CSF Funding**

No.

**ESVONC**—**European Society of Veterinary Oncology**

## ESVONC‐P‐1

## Unveiling the tumour microenvironment in canine pulmonary adenocarcinoma through single‐cell RNA sequencing

### E. Rizzoli^1^, A. Fastrès^1^, L. Fievez^2,3^, E. Roels^1^, T. Marichal^2,3,4^, C. Clercx^1^

#### ^1^Faculty of Veterinary Medicine, University of Liège Department of Companion Animal Clinical Sciences, Liège, Belgium; ^2^Faculty of Veterinary Medicine, University of Liège Department of Functional Sciences, Liège, Belgium; ^3^GIGA Institute, University of Liège Department of Functional Sciences, Liège, Belgium; ^4^WEL Research Institute Department of Functional Sciences, Wavre, Belgium

Primary pulmonary carcinoma, a prevalent malignancy in humans, is relatively rare in dogs. When possible, surgical resection is the treatment of choice, but the prognosis remains poor in advanced stages. Deciphering the complex tumour microenvironment is pivotal as it may reveal targets for adjuvant therapies. This study aimed to investigate specific cancer‐associated cell types and gene expression profiles in canine pulmonary adenocarcinoma (PAC), the most common histologic subtype of canine primary pulmonary carcinoma.

Fresh leftover biopsy samples from three histologically confirmed primary PAC cases, surgically excised via curative‐intent lobectomy, were obtained. Tissues were dissociated into single‐cell suspensions and droplet based single‐cell RNA sequencing (10× Genomics) was conducted. The data from PAC samples were integrated with previously analysed datasets of four healthy lung samples for downstream analysis. Cell subpopulations were identified based on their gene expression and their relative abundances in both conditions were statistically compared. Differentially expressed genes between PAC and healthy samples were calculated and gene set enrichment analyses were conducted to better characterise cancer‐related heterogeneity within cell types.

In PAC samples, four main cell compartments were observed, as in healthy lungs: mesenchymal, immune, epithelial, and endothelial. Most cells subpopulations identified in healthy lungs were also present in tumours. PAC samples exhibited a higher relative proportion of mature dendritic cells and lymphoid cells, particularly regulatory T lymphocytes, B lymphocytes, and plasma cells. Additional immune cell subtypes were even exclusively found in PAC samples. As expected, PAC epithelial cells were dominated by multiple clusters identified as cancer cells clusters, each exhibiting a particular gene expression signature. Differential gene expression analyses revealed distinct gene expression profiles between PAC and healthy lung tissues. For instance, cancer‐associated fibroblasts (CAFs) overexpressed genes associated with contractility, inflammation, matrix remodelling, and collagen synthesis. Additionally, gene set enrichment analysis of CAFs revealed significant enrichment of pathways associated with epithelial‐to‐mesenchymal transition.

This study expands our understanding of the cellular and molecular heterogeneity of the canine pulmonary adenocarcinoma microenvironment, identifying molecular signatures of cancer‐associated cell types. The gained insights carry significant implications for the development of new strategies for the treatment of this condition, especially in advanced stages or unresectable cancers.

**Disclosures**

**Source of Funding**

National Funds for Scientific Research and Special Research Funds from the Faculty of Veterinary Medicine of the University of Liège.

**ECVIM CSF Funding**

No.

**ESVONC**—**European Society of Veterinary Oncology**

## ESVONC‐P‐2

## Investigating the national cause of death in dogs from 2013 to 2023: An oncological perspective

### H. Brøgger^1^, B. Børresen^2^, M. Arendt^2^, A. Müller^2^, H. Proschowsky^2^

#### ^1^Department of Veterinary Clinical Sciences, University of Copenhagen University of Copenhagen, Frederiksberg, Denmark; ^2^Department of Veterinary Clinical Sciences, University of Copenhagen, Frederiksberg, Denmark

A previous study including owner‐reported data more than 20 years ago showed that old age was the leading cause of death among dogs nationally. This was followed by cancer, which was also the leading disease‐specific cause of death. We wanted to establish if this distribution has changed over time. In addition, from an oncological perspective, we wanted to investigate if we could get more comprehensive information regarding the location and knowledge on breed specific differences on age and cause of death.

We designed an online questionnaire which was generated and validated through an iterative process. The questionnaire focused on collecting owner‐reported data on cause of death for dogs who had died during a ten‐year period from 2013 to 2023. The questionnaire was distributed by the University and the national kennel club's social media platforms (Facebook) and Instagram.

In total we had 24630 respondents of which 21157 completed the questionnaire. The questionnaire was subject to recall bias with an increase in the number of reported deaths from 2013 to 2023. Labrador Retrievers were the most common breed reported in this study followed by German Shepherds and Golden Retrievers, which is in alignment with the popularity of these breeds according to the national kennel club. In total 92% of dogs were reported to have been euthanized while 8% died spontaneously. The leading cause of death was cancer with 23% of owners reporting that their dog died due to cancer. This was followed by 14% of owners reporting that their dogs died due to old age. The average age of dogs dying from cancer was 10 compared to dogs dying from age at 13. Abdominal cancer was the most commonly reported cancer location. There were clear differences between the age at death and cause of death between dog breeds and dog sizes. As an example, 43% of Flat Coated Retrievers died of cancer versus only 23% of Labrador retrievers and 11% of Cavalier King Charles Spaniels. Likewise, the mean age at death was significantly different between dogs when grouped by weight.

In conclusion, our study shows that cancer has now exceeded old age as the most common cause of death. This large comprehensive death registry study shows differences between individual breeds and highlights breed predispositions to cancer as well as other diseases.

**Disclosures**

**Source of Funding**

**ECVIM CSF Funding**

No.

**ESVONC**—**European Society of Veterinary Oncology**

## ESVONC‐P‐3

## Serum amino acid profile in canine oncologic disease

### R. Dini^1^, F. Bartoli^2^, V. Habermaass^3^, E. Gori^3^, V. Marchetti^3^, A. Pierini^3^

#### ^1^Veterinary Sciences University of Pisa Veterinary Sciences University of Pisa, Pisa, Italy; ^2^Department of Translational Research University of Pisa, Pisa, Italy; ^3^Veterinary Sciences University of Pisa, Pisa, Italy

In humans, different oncologic diseases may result in alterations in amino acids (AAs) metabolism, with several mechanisms involved and they currently also represent a prognostic and therapeutic target. Scarce literature is available in veterinary medicine, with few case‐control studies focused on lymphoma, mammary gland tumor, brain tumor, hepatocellular carcinoma, and oral melanoma.

The aim of this study was to evaluate serum AAs in dogs with cancer compared to a healthy control group.

Leftover serum samples of 50 client‐owned dogs with cytological or histological diagnosis of malignant tumor and 25 healthy blood‐donor dogs were included in this case‐control study. Dogs receiving AAs supplementation were excluded. Dogs were classified as having local (19/49, 39%) or metastatic/diffuse disease (30/49, 61%) based on the cancer staging. Glycine(GLY), alanine(ALA), valine(VAL), leucine(LEU), isoleucine(ILE), proline(PRO), serine(SER), threonine(THR), cysteine(CYS), methionine(MET), phenylalanine(PHE), tyrosine(TYR), tryptophan(TRP), aspartate(ASP), glutamic acid(GLU), histidine(HIS), lysine(LYS) and arginine(ARG) were measured with High‐Performance Liquid Chromatography (HPLC), with the Waters AccQ Tag method employed for sample preparation. Essential (EAAs), non‐essential (NEAAs), branched‐chain (BCAAs) and aromatic (AAAs) amino acids together with EAA/NEAA and BCAA/AAA ratios were calculated. All continuous data were non‐normally distributed and then reported as median and range in square brackets. Mann–Whitney U‐test was used to compare serum AAs and ratios between groups.

The following AAs resulted significantly lower in the oncologic group compared to controls: HIS (100.9 [6.9–324.6] vs. 197.4 [24.9–323.4] nmol/mL; *p*<0.0001), ARG (38.6 [4–79] vs. 59.2 [0,1–172.5] nmol/mL; *p* = 0.001), ALA (68.8 [7.7–149.1] vs. 94.5 [56.6–226] nmol/ml; *p* = 0.003), PRO (4.1 [0.05–21.9] vs. 6.3 [4–67.5] nmol/ml; *p*<0.0001), TYR (6.0 [1.1–56.5] vs. 11.3[6.3–54] nmol/ml; p<0.0001], ILE (20.1 [2–87.5] vs. 24.3 [12.7–45.4] nmol/ml; *p* = 0.013). Whereas GLU (16.8 [.9–49.3] vs. 8.8 [4.4–20.6] nmol/ml; *p*<0.0001) and MET (42.2 [3.9–98.4] vs. 4.8 [.3–58.5] nmol/ml; p<0.0001) resulted significantly higher in the study group than healthy dogs. No serum AAs were found different between dogs with local or metastatic/diffuse disease. Both EAAs/NEAAs and BCAAs/AAAs ratios did not significantly differ between healthy vs. oncologic group.

Many serum AAs resulted significantly different between cancer and healthy dogs. Our results confirm higher concentration of GLU in dogs with cancer as previously reported in dogs. Therefore, GLU may be used as a biomarker in dogs with cancer and their follow‐up.

**Disclosures**

**Source of Funding**

**ECVIM CSF Funding**

No.

**ESVONC**—**European Society of Veterinary Oncology**

## ESVONC‐P‐4

## Tolerability and outcome of a higher fractionated dose of cyclophosphamide in the 19‐week cyclophosphamide, doxorubicin, vincristine, and prednisone based protocol in dogs with high‐grade multicentric B‐cell lymphoma

### C. Fejős^1^, V. Meier^2^, L. Beatrice^3^

#### ^1^Vetsuisse Faculty, University of Zurich Department for Small Animals, Division of Radiation Oncology, Zurich, Switzerland; ^2^Vetsuisse Faculty, University of Zurich, Switzerland Department of Small Animals, Division of Radiation Oncology, Zurich, Switzerland; ^3^Vetsuisse Faculty, University of Zurich, Switzerland Department of Small Animals, Diviosn of Radiation Oncology, Zurich, Switzerland

The standard of care for canine intermediate‐ and high‐grade lymphoma is the administration of multiagent protocols containing cyclophosphamide, vincristine, and doxorubicin, with 19‐week CHOP being one of the most commonly used regimens. As in humans, more dose‐intense protocols have been associated with longer remission and survival times. Although these patients may experience survival benefits, in dogs, increased toxicity, hospitalization, and frequent dose delays can lead to owner dissatisfaction and even treatment discontinuation.

The aim of this study was to describe the clinical characteristics, adverse events (AEs), and outcome in dogs with high‐grade lymphoma receiving a 300mg/m^2^fractionated dose of oral cyclophosphamide in the 19‐week CHOP protocol.

Dogs diagnosed with high‐grade, B‐cell multicentric lymphoma treated at least in one cycle with the intended dose of 300 mg/m^2^of cyclophosphamide in the 19‐week CHOP protocol as first‐line treatment were included in this retrospective study. Cyclophosphamide was administered orally at a fractionated dose of 75 mg/m² over 4 days without furosemide. Data were collected on diagnosis, staging, AEs graded according to VCOG‐CTCAEv2, and outcome. The Kaplan‐Meier method was used to calculate progression‐free survival (PFS) and overall survival (OS).

Twenty‐four dogs fulfilled the inclusion criteria. Nine (38%) dogs had stage V disease, and 14 (58%) were clinically unwell (substage b). The overall response rate was 100%, with 22 (92%) patients achieving complete remission and two (8%) dogs experiencing a partial response. Seventy‐nine percent (19/24) of patients had at least one dose reduction, and 83% (20/24) had at least one dose delay due to toxicity caused by ≥ grade 2 neutropenia or grade 3 gastrointestinal toxicity. Six dose‐delaying AEs occurred in five dogs following cyclophosphamide administration, with two (8%) developing hemorrhagic cystitis and three having grade 2 neutropenia. The majority of dose‐delaying toxicities were vincristine related, with 17 AEs occurring in 13 (54%) dogs after the first dose and 23 in 16 (67%) after the second dose of vincristine in the cycle. The dose of vincristine was reduced in 79% (19/24) of patients. In total, Ffve dogs required hospitalization, and the protocol was discontinued in one patient due to AEs. The median PFS was 266 days, and the median OS was 401 days, respectively.

The results indicate that although the fractionated higher dose of cyclophosphamide is well tolerated, the protocol caused a high number of AEs, leading to frequent dose reductions and delays. PFS and OS were similar to previously reported outcomes.

**Disclosures**

**Source of Funding**

No funding.

**ECVIM CSF Funding**

No.

**ESVE**—**European Society of Veterinary Endocrinology**

## ESVE‐P‐1

## Increased level of circulating trimethylamine N‐oxide (TMAO) in dogs with naturally occurring diabetes mellitus: a pilot study

### H.Y. Nilsen^1,2^, R. Landberg^3^, T. Fall^4^, J. Häggström^5^, T. Falk^1,6^, F.M. Panah^7^, L. Krych^7^, C. Hartmann Friis Hansen^1^, L. Hoier Olsen^1^

#### ^1^University of Copenhagen Department for Veterinary and Animal Sciences, Copenhagen, Denmark; ^2^Blue Star Animal Hospital Department for Veterinary and Animal Sciences, Gothenburg, Sweden; ^3^Chalmers University of Technology Department of Biology and Biological Engineering, Food and Nutrition Science, Gothenburg, Sweden; ^4^Uppsala University Department of Medical Sciences, Uppsala, Sweden; ^5^Swedish University of Agricultural Sciences Department of Clinical Sciences, Uppsala, Sweden; ^6^Din Veterinaer Department for Veterinary and Animal Sciences, Helsingborg, Sweden; ^7^University of Copenhagen Department of Food Science, Copenhagen, Denmark

Trimethylamine N‐oxide (TMAO) is a gut microbiota‐derived metabolite produced from dietary nutrients. In humans, high levels of circulating TMAO have been linked to hypertension, cardiovascular disease, diabetes mellitus (DM), cancer, kidney function as well as all‐cause mortality. Dogs with congestive heart failure secondary to myxomatous mitral valve disease (MMVD) had higher concentrations of TMAO compared to asymptomatic MMVD‐dogs as well as healthy controls.

The aim of this study was to determine whether dogs with naturally occurring diabetes mellitus and healthy controls have different levels of circulating gut microbiota‐derived metabolites.

18 dogs were included in this prospective cohort study, of which 10 dogs had a diagnosis of naturally occurring diabetes mellitus (DM) and 8 dogs were healthy controls (weight‐/age‐/gender‐matched). Diabetic dogs received insulin treatment by the owners. Exclusion criteria included treatment with systemic antibiotics or probiotics for a period of six months prior to inclusion, dogs receiving a raw food diet during the last six months, repeated episodes of vomiting or diarrhea for the last 3 months and a diagnosis or clinical signs of other endocrinopathies or systemic disease.

Blood analysis included circulating trimethylamine N‐oxide (TMAO) and short‐chain fatty acids (SCFAs).

TMAO was significantly higher in dogs with DM (median 23.8 (IQR 7.3–38.1)) compared to healthy controls (6.8 (IQR 3.8–12.3)), *P* = 0.037. Regarding SCFAs there were no statistically significant changes between the groups.

In conclusion, a higher circulating level of TMAO was found in dogs with naturally occurring DM compared to controls. This study suggests that even in dogs, TMAO is a biomarker associated with DM. Further studies are needed to investigate the potential of TMAO as prognostic biomarker as well as intervention target.

**Disclosures**

**Source of Funding**

Marianne Thedes foundation (Sweden) Blå Stjärnans Djursjukhus personalutvecklingsfond (Sweden).

**ECVIM CSF Funding**

No.

**ESVE**—**European Society of Veterinary Endocrinology**

## ESVE‐P‐2

## Urinary active transforming growth factor beta 1 in cats with hyperthyroidism before and after radioiodine treatment

### L. Pérez‐López^1^, L. Stammeleer^2^, L. Duchateau^3^, E. van den Broecke^2^, L. van Mulders^2^, E. Meyer^4^, S. Daminet^2^

#### ^1^Institute of Biomedical and Health Research, University of Las Palmas, Las Palmas de Gran Canaria, Spain; ^2^Faculty of Veterinary Medicine, Ghent University Small Animal, Merelbeke, Belgium; ^3^Biometrics Research Centre, Faculty of Veterinary Medicine, Ghent University, Merelbeke, Belgium; ^4^Faculty of Veterinary Medicine, Ghent University Veterinary and Biosciences, Merelbeke, Belgium

Novel renal markers allowing early diagnosis of feline chronic kidney disease and prediction of post‐treatment renal azotemia in hyperthyroid cats are highly anticipated. The urinary active transforming growth factor beta 1 – creatinine ratio (uaTGFβ1/Cr) may be a useful marker of early kidney impairment in cats and its ability to predict development of azotemia in hyperthyroid cats remains to be assessed.

Therefore, the aim of this prospective study was to assess uaTGFβ1/Cr in cats with hyperthyroidism before (T0) and 3 (T3), 6 (T6), and 12 (T12) months after radioiodine (^131^I) treatment and to evaluate the utility of this normalized marker to predict renal azotemia. In addition, a pilot cross‐sectional study was included to compare uaTGFβ1/Cr at baseline in hyperthyroid cats with healthy senior cats (>10 years) and CKD cats (serum Cr > 2.0 mg/dL and urinary specific gravity <1.035 or urinary protein/Cr >0.4).

Sixty‐two hyperthyroid cats, 30 healthy senior cats, and 21 CKD cats were included in this cross‐sectional study. Median and interquartile range (Q1–Q3) uaTGFβ1/Cr values were 17.7 (1.9–38.5) pg/mg in hyperthyroid cats, 17.0 (12.6–22.3) pg/mg in healthy senior cats, and 22.4 (16.1–34.9) pg/mg in CKD cats. No statistical differences were found between these 3 groups using the Kruskal Wallis test (*p* = 0.240).

A total of 61 hyperthyroid cats were available for follow‐up, of these *n* = 54 were assessed at T3, *n* = 46 at T6 and *n* = 35 at T12. Median and Q1–Q3 of uaTGFβ1/Cr were 17.9 (1.8–36.4) pg/mg at T0, 8.6 (1.8–29.5) pg/mg at T3, 13.1 (3.3–28.4) pg/mg at T6, and 17.9 (11.0–26.6) pg/mg at T12. No statistical differences were found between the different time points. Post‐treatment renal azotemia developed in 18 out 61cats (29.5 %). No differences were found for uaTGFβ1/Cr between cats that developed azotemia after^131^I treatment and non‐azotemic cats at T3 [17.4 (2.0–23.9) vs. 8.45 (2.0–26.6) pg/mg; *p* = 0.569], at T6 [18.9 (10.8–25.7) vs. 11.3 (3.3–26.4)], or T12 [24.9 (18.4–63.2) vs. 15.7 (9.4‐37.6), *p* = 0.138].

Our data indicate that feline uaTGFβ1/Cr before treatment of hyperthyroidism was not useful to predict post‐treatment renal azotemia. In addition, increased TT4 levels in hyperthyroid cats did not seem to affect uaTGFβ1/Cr.

**Disclosures**

This study was funded by EveryCat Health Foundation (EC23‐069).

**Source of Funding**

This study was funded by EveryCat Health Foundation (EC23‐069). The first author received the Margarita Salas postdoctoral research contract given by University of Las Palmas de Gran Canaria and the Spanish Ministry of Universities granted by Order UNI/501/2021 of May 26, as well as financing by the European Union‐Next Generation EU Funds.

**ECVIM CSF Funding**

No.

**ESVE**—**European Society of Veterinary Endocrinology**

## ESVE‐P‐3

## An investigation into venous blood electrolytes, blood gas and markers of perfusion following transsphenoidal hypophysectomy to treat hypersomatotropism in cats

### M. Drapeau^1^, C. Scudder^2^, D. Church^2^, J. Fenn^2^

#### ^1^Royal Veterinary College, Hatfield, United Kingdom; ^2^Department of Clinical Science and Services, Royal Veterinary College Department of Clinical Science and Services, Hatfield, United Kingdom

Cats with hypersomatotropism (HST) and diabetes mellitus (DM) can be successfully treated with transsphenoidal hypophysectomy. Numerous electrolyte derangements are possible following hypophysectomy due to iatrogenic diabetes insipidus (DI) and changing insulin requirement for glycaemic control. This study describes blood electrolyte abnormalities within the first 48 hours post‐hypophysectomy and investigates associations with clinical variables and outcome.

Medical records of cats with HST and DM treated by hypophysectomy between 2012 and 2023 were eligible for inclusion. Preoperative descriptive and surgical variables, venous blood gas, electrolyte and metabolite results (preoperatively and +6, +12, +24, +48 hours postoperatively) and outcome data (persistent DI, DM remission, peri‐operative death and survival time) were recorded. Data are presented as median and range. Wilcoxon Signed Rank Tests and Friedman tests were performed where appropriate with Bonferroni correction for multiple comparisons.

Ninety cats were included. Hypernatremia (>153 mmol/L) developed postoperatively in 80.2% and hypocalcaemia (<1.28 mmol/L) in 76.5% of cats. Median plasma sodium concentrations (mmol/L) were significantly higher at all time points post‐operatively (+6h was 154 [145–166], P<.001; +12h was 154 [140–183], *P*<.001; +24h was 152 [135–191], *P*<.001; and +48h was 151 [134–165], *P* = .01) compared to baseline (148 [142–165]). Median plasma calcium concentrations (mmol/L) were significantly lower at all timepoints (+6 h was 1.23 [.85–2.46], *P*<.001; +12h was 1.25 [.93–1.47], P<.001; +24h was 1.24 (.96–2.13), P.001; and +48 h was 1.25 (.95–2.08) compared to baseline. There was no significant association between postoperative sodium or calcium plasma concentrations and clinical or surgical variables, or patient outcome.

Post‐operative hypernatremia and hypocalcaemia were common changes not associated with survival or long‐term endocrine outcome.

**Disclosures**

**Source of Funding**

**ECVIM CSF Funding**

No.

**ESVE**—**European Society of Veterinary Endocrinology**

## ESVE‐P‐4

## Patterns of the low dose dexamethasone suppression test in dogs with pheochromocytoma

### A. Domínguez Madsen^1^, M.I. Rodríguez Piñeiro^1^, R. Movilla Fernández^1^, R. Paniagua Jiménez^2^

#### ^1^Hospital Veterinario Puchol Internal Medicine, Hospital Veterinario Puchol, Madrid, Spain; ^2^Universidad Europea Internal Medicine, Universidad Europea, Madrid, Spain

Functional adrenocortical tumors (FATs) and pheochromocytomas (PCCs) may share clinical and clinicopathological features (PU/PD, panting, hypertension‐associated target‐organ damages, increased liver enzymes, hypercholesterolemia). Imaging techniques generally show a unilateral adrenal enlargement (nodule/mass) in both cases. Furthermore, some cases of pituitary‐dependant hyperadrenocorticism (PDH) can also cause nodular hyperplasia of adrenals and, sometimes, a single nodule without contralateral atrophy can be visualized. FATs are much more common than PCCs, therefore, in questionable cases, hyperadrenocorticism (HAC) should be ruled out first by the appropriate screening test, ideally using a low dose dexamethasone suppression test (LDDST).

Different LDDST response patterns with variable positive predictive values (PPV) for the diagnosis of HAC have been described. Furthermore, non‐invasive presurgical PCC diagnosis has become more attainable with the use of cytology and plasmatic/urinary free metanephrines.

The aim of this retrospective study is to describe the different LDDST patterns in dogs diagnosed with PCC.

Included were dogs diagnosed with PCC (compatible clinical signs + unilateral adrenal lesion + cytology/histology results compatible with a medullary tumour and/or clearly increased plasma/urine free normetanephrine and/or metanephrine concentrations), with a LDDST performed. Other data (ACTH‐stimulation test, endogenous ACTH (e‐ACTH) results, response to trilostane) were recorded when available.

Fifteen dogs met inclusion criteria. PCC diagnosis was confirmed by cytology in 6 dogs, histopathology in 2, plasma or urinary free metanephrines in 9 and 1, respectively, and by several methods in 3. The next patterns of LDDST were observed: 8/15 complete suppression, 2/15 partial suppression, 1/15 lack of suppression, and 4/15 escape.

In this study, 3/15 dogs with PCC showed partial or lack of suppression LDDST patterns. Partial and, especially, lack suppression have been recently described to have a good to excellent PPV for the diagnosis of HAC. In all 3 cases contralateral adrenal gland was not atrophied, and eACTH was in the normal range in the only dog measured, however PDH could still be possible in these dogs. A false positive result for HAC was suspected in one dog in the absence of response to trilostane treatment. Despite its low PPV, it is worth mentioning that 4 out of 15 dogs showed escape pattern.

The results obtained here suggest that, in the presence of an adrenal nodule, obtaining a LDDST compatible with HAC should not preclude a diagnosis of PCC, which should also be investigated (cytology, free metanephrines). The proportion of dogs with PCC and concurrent PDH is currently unknown and difficult to determine.

**Disclosures**

**Source of Funding**

**ECVIM CSF Funding**

No.

**ESVE**—**European Society of Veterinary Endocrinology**

## ESVE‐P‐6

## Performance of the Addison Detect Tool to screen for canine hypoadrenocorticism: External validation

### K. Le Boedec^1^, A. Da Silva^2^, A. Briend‐Marchal ^3^, C. Mooney^2^

#### ^1^Centre Hospitalier Veterinaire Fregis—IVC Evidensia France, Paris, France; ^2^School of Veterinary Medicine | University College Dublin, Dublin, Ireland; ^3^Anydiag laboratory, Paris, France

The Addison Detect Tool (ADT) is a web app based on a machine learning algorithm designed to assist clinicians in decision making when canine hypoadrenocorticism is suspected. Although it has been externally validated, the sample contained relatively few addisonian dogs (*n* = 17) and only 6 (35.3%) with a Na:K ratio >27.

The aim of this study was to further investigate the performance of the ADT using a larger external validation sample population.

Data were collected retrospectively from two referral centers in Ireland and France. Dogs were excluded if they had received glucocorticoids within 30 days of testing or if mandatory clinicopathological data were missing. Dogs were included if they could be reliably classified as addisonian or control. The diagnosis of hypoadrenocorticism was based on ACTH stimulation test (ACTHST) results and consistent clinical features. Hypoadrenocorticism was suspected in all control dogs but subsequently excluded based on baseline cortisol concentration (BCC) or ACTHST results.

Ninety‐nine control and 99 addisonian dogs (including 28 with a Na:K ratio >27) were included. The area under the receiver‐operating‐characteristic curve of the ADT was 0.94 (95% confidence interval [CI]: 0.91–0.97), which was similar to that of BCC (0.94 [95% CI: 0.90–0.98]). Using predicted probability thresholds of 10%, 50%, and 80%, sensitivity and specificity were 92% (95% CI: 85–96) and 80% (95% CI: 71–87), 60% (95% CI: 49–69) and 99% (95% CI: 95–100), and 34% (95% CI: 25–45) and 100% (95% CI: 96–100), respectively. All the addisonian dogs rejected by the ADT (*n* = 8) had a Na:K ratio >27.

In conclusion, although overall diagnostic performance of the ADT is similar to that of BCC, the results should be interpreted cautiously if the Na:K ratio is >27.

**Disclosures**

The first author (KLB) owned the Addison Detect Tool website that allows calculation of the predicted probability of canine hypoadrenocorticism but the website is completely free and is used without registration (so no personal data are entered and therefore can be collected)

**Source of Funding**

None.

**ECVIM CSF Funding**

No.

**ESVE**—**European Society of Veterinary Endocrinology**

## ESVE‐P‐7

## Characterization of hyaluronic acid concentrations in dogs with naturally‐occurring hypercortisolism and possible implications

### P. Lunet Marques^1,2^, D. Silva^1,2^, I. Oliveira^1,2^, T. Domingues^3^, M. Re^4^, L. Mateus^1,2^, F. del Baldo^4^, R. Oliveira Leal^1,2^

#### ^1^Centre for Interdisciplinary Research in Animal Health, Fac.Vet.Med., ULisbon Clinical research, Lisbon, Portugal; ^2^Associate Laboratory for Animal and Veterinary Sciences (AL4AnimalS) Clinical research, Lisbon, Portugal; ^3^FCiências.ID—Associação para a Investigação e Desenvolvimento de Ciências Statistics and operational research, Lisbon, Portugal; ^4^Department of Veterinary Medical Science, University of Bologna Veterinary Medical Science, Bologna, Italy

Proteinuria is a common finding in dogs with naturally‐occurring hypercortisolism, with an estimated incidence as high as 75%. Although hemodynamic factors such as hypertension and changes on glomerular filtration rate may contribute to proteinuria, its etiology is still poorly understood. Emerging studies have revealed a compelling correlation between endothelial glycocalyx (EG) breakdown and proteinuria in humans. Serum circulating hyaluronan has been considered a reliable biomarker of EG shedding not only in humans but in various animal models. Despite the increasing evidence that supports the role of EG shedding in the pathophysiology of several diseases, little is known about its change in canine hypercortisolism. This study aims to evaluate serum hyaluronan concentration (SHC) in dogs with naturally‐occurring hypercortisolism and its possible relationship with proteinuria.

A cross‐sectional multicentric study was conducted. SHC was measured on leftover samples from dogs with naturally‐occurring hypercortisolism (diagnosed from 2022 to 2024) before starting the Trilostane treatment. All serum samples were stored within 24 h of collection, stored and frozen at ‐20 or ‐80°C until analysis. Serum hyaluronan was measured using a kit previously validated in dogs (Hyaluronan Quantikine ELISA, R&D Systems). Paired urinary protein‐creatinine ratio (UPCR) and urine specific gravity (USG) values for each animal were obtained from medical records. Obtained values were compared with historic controls from previous studies, including healthy dogs and dogs in hypercoagulable states. Dogs were grouped regarding their degree of proteinuria: proteinuric (0.5 < UPCR < 2), non‐proteinuric (UPCR < 0.5) and overtly proteinuric (UPCR > 2).

The SHC (median [range]) in dogs with hypercortisolism was 45.94 [9.70–265.07] ng/mL. The median value was similar to those previously obtained healthy dogs (45.7 [8.7–80.2] ng/mL) and the range was similar to those previously obtained in dogs in hypercoagulable states (92.40 [16.9–247.6] ng/mL) No correlation between SHC and UPCR (*p* = 0.119) was found. SHC between the 3 group of proteinuria was not different (*p* = 0.062).

Although no apparent association with proteinuric state was found, to the author's best knowledge, this is the first study assessing SHC in dogs with naturally occurring hypercortisolism. Obtained values were similar, in range, to those previously identified in dogs with a hypercoagulable state, pointing towards a potential future role of this biomarker on the individual identification of hypercoagulability.

**Disclosures**

**Source of Funding**

This work was supported by FCT—Fundação para a Ciência e Tecnologia IP, grant UIDB/00276/2020 and by LA/P/0059/2020—AL4AnimalS.

**ECVIM CSF Funding**

No.

**ESVE**—**European Society of Veterinary Endocrinology**

## ESVE‐P‐8

## Iodine is not increased in sera from dogs with autoimmune thyroiditis or hypothyroidism&nbsp;

### B. Petroff^1^, R. Egbert^1^, J. Buchweitz^1^, M. Godfrey^1^

#### ^1^Michigan State University PDI, CVM, Michigan State University, East Lansing Michigan, USA, Lansing, United States

Increased iodine intake is an established risk factor for autoimmune thyroiditis in rodents and humans. The relationship between iodine and canine autoimmune thyroiditis is unclear. In this study, we hypothesized that serum iodine concentrations are greater in dogs with autoimmune thyroid disease. We measured total and inorganic iodine concentrations in surplus sera from dogs with autoimmune thyroiditis but normal thyroid hormones (AIT; *n* = 94) or hypothyroidism (HT; *n* = 96) and in dogs with normal thyroid function and negative autoantibodies (NML; *n* = 84). Study samples were obtained through thyroid panel submission to the Michigan State University Veterinary Diagnostic Laboratory and only surplus sera were used for study. Thyroglobulin autoantibody was used as a marker for autoimmune thyroiditis. Total T4 and free T4 were measured using validated immunoassay while TSH (thyroid stimulating hormone) was assayed via automated chemiluminescent reagents for canine TSH. Total serum iodine in normal dogs ranged from 56.7 to 1526 ppb while inorganic iodine ranged from 32.1 to 1199 ppb. Total and inorganic iodine were significantly decreased in sera from hypothyroid dogs in comparison to normal dogs (NML: 167.8 ± 17.9 ppb total iodine vs. HT: 104.1 ± 5.3 ppb total iodine; *p* ≤ 0.001; NML: 123.8 ± 14.2 ppb inorganic iodine vs. HT: 71.2 ± 3.9 ppb inorganic iodine; *p* ≤ 0.001). Mean serum iodine concentrations did not differ significantly between normal dogs and dogs with AIT alone (NML: 167.8 ± 17.9 ppb total iodine vs. AIT: 158.7 ± 7.9 ppb total iodine; *p* = 0.84; NML: 123.8 ± 14.2 ppb inorganic iodine versus AIT:131.2 ± 6.8 ppb inorganic iodine; *p* = 0.82). Neither autoimmune thyroiditis nor hypothyroidism was associated with increased serum iodine in this study.

**Disclosures**

Authors Egbert, Petroff and Buchweitz are employed at the MSU Veterinary Diagnostic Laboratory where the testing was performed.

**Source of Funding**

Michigan State University College of Veterinary Medicine.

**ECVIM CSF Funding**

No.

**ESVE**—**European Society of Veterinary Endocrinology**

## ESVE‐P‐9

## Comparison of insulin requirements and fructosamine concentrations between diabetic dogs with naturally occurring hypercortisolism and hypothyroidism

### D. Miceli^1^, O. Pignataro^2^

#### ^1^IBYME CONICET, Buenos Aires, Argentina; ^2^IBYME CONICET Ibyme, Buenos Aires, Argentina

Hormonal insulin antagonism may induce insulin resistance in dogs. Naturally occurring hypercortisolism and hypothyroidism are endocrinopathies that reduce insulin sensitivity, potentially predisposing to the development of diabetes mellitus (DM) and leading to instability in diabetic control. Studies in dogs with hypothyroidism and concurrent DM are lacking. The aim of this study was to compare insulin requirements and serum fructosamine concentrations in diabetic dogs with naturally‐occurring hypercortisolism and hypothyroidism. A total of 29 diabetic dogs were included in this retrospective study: 11 dogs with concurrent hypercortisolism, 8 dogs with concurrent hypothyroidism, and 10 dogs with DM only. Serum fructosamine concentration, insulin dose and insulin resistance index (IRI) were assessed before the diagnosis (t0) of the concurrent disease (hypercortisolism or hypothyroidism), and after 1 month (t1) and 3 months (t2) of trilostane or levothyroxine treatment. Dogs with hypercortisolism and hypothyroidism received fixed doses of trilostane (1 mg/kg q12h) or levothyroxine (10 μg/kg q12h) during the study period. All diabetic dogs received the same commercial diabetic food and insulin therapy (NPH or Detemir). In dogs with hypercortisolism and concurrent DM, the median insulin dose increased significantly at t1 (*P*<0.05) and t2 (*P*<0.05) during trilostane treatment, compared to t0. No significant differences were observed in serum fructosamine concentration and IRI during trilostane treatment. In dogs with hypothyroidism and concurrent DM, the median insulin dose did not differ significantly during levothyroxine treatment. Median serum fructosamine concentration and IRI decreased significantly during levothyroxine treatment at t1 (*P*<0.05) and t2 (*P*<0.05), compared to t0, and between t1 and t2 (*P*<0.05). Significant differences were observed when comparing insulin doses between diabetic dogs with hypercortisolism and hypothyroidism at t1 and t2 (*P*<0.01). Serum fructosamine concentration in hypothyroid dogs before treatment with levothyroxine was significantly higher than in dogs with hypercortisolism before treatment with trilostane (*P*<0.01), but significantly lower after trilostane treatment at t2 (P<0.05). IRI in diabetic dogs with hypothyroidism was significantly lower than in diabetic dogs with hypercortisolism at t1 and t2 (P<0.05). This study suggests that levothyroxine treatment of diabetic dogs with naturally occurring hypothyroidism would be more effective than trilostane treatment of diabetic dogs with naturally occurring hypercortisolism in achieving adequate diabetic control, as it improves serum fructosamine concentrations and IRI at 1 month and 3 months after treatment initiation.

**Disclosures**

**Source of Funding**

**ECVIM CSF Funding**

No.

**ESVE**—**European Society of Veterinary Endocrinology**

## ESVE‐P‐10

## Owner points of view and perceived quality of life of their diabetic cats following hypophysectomy

### E. Shelton^1^, R. Jepson^1^, D. Church^1^, J. Fenn^1^, C. Scudder^1^

#### ^1^Department of Clinical Science and Services, Royal Veterinary College, Hatfield, United Kingdom

Hypophysectomy provides the most favourable long‐term outcome for cats with hypersomatotropism and concurrent diabetes mellitus (DM). This study aimed to describe owner perception of hypophysectomy on quality of life (QoL).

Owners whose cats had undergone hypophysectomy between 2015 and 2022 for the management of hypersomatotropism with DM were identified. Telephone interviews were performed with a subset of owners to formulate questions addressing points of view regarding hypersomatotropism and hypophysectomy until response saturation occurred. The DIAQoL‐Pet questionnaire was adjusted by including newly formulated questions developed following telephone interviews. The questionnaire was then distributed to owners of cats with hypersomatotropism with DM pre‐hypophysectomy (group A) from March 2023 to 2024 and to the retrospectively recruited owners (group B) post‐hypophysectomy. Item‐weighted impact scores (IWIS) and an average‐weighted impact score (AWIS) were calculated. AWIS and IWIS were compared between groups using Mann–Whitney *U* tests.

There were nine owners in group A and 27 in group B. The IWIS scores were significantly more negative in group A compared to B for “worry” (*P* = .039), “no treats” (*P* = .029), “medication worries” (*P* =.039), “pet unwell” (*P* = .024), “future care” (*P* = 026), “worry hypoglycaemia” (*P* = .005), “worry DKA” (*P* = <.001), “worry vision” (*P* = .039) and “special bond” (*P* = .003). In contrast, median AWIS was not significantly different before (‐1.46, range ‐5.11 to ‐0.36) and after (1.11, range ‐5.00 to 0.25) hypophysectomy (*P* = .136). Eighty‐eight percent of respondents would definitely request hypophysectomy again.

Most owners perceive hypophysectomy to be a beneficial intervention for the quality of life of themselves and their cat.

**Disclosures**

All authors are employed at the Royal Veterinary College where feline hypophysectomy is offered as a treatment option for hypersomatotropism.

**Source of Funding**

**ECVIM CSF Funding**

No.

**ESVE**—**European Society of Veterinary Endocrinology**

## ESVE‐P‐11

## Naturally occurring hypothyroidism in adult cats: A prospective multi‐center study in Central Europe

### F. Leuthard^1^, A. Carranza Valencia^2^, M. Wenger^1^, F. Tschuor^1^, F. Zeugswetter^3^

#### ^1^Tierklinik Mittelland, Oftringen‐Zofingen, Switzerland; ^2^University of Bern Department of Clinical Veterinary Science, Bern, Switzerland; ^3^University of Veterinary Medicine Vienna, Vienna, Austria

Research on the prevalence of naturally occurring hypothyroidism in adult cats is limited to 12 reported cases in the last three decades, highlighting the need for more comprehensive studies. Diagnosis is difficult, given that clinical signs are unspecific and thyroxine concentration may be in the low normal reference range. This prospective multicenter study was conducted to determine the prevalence of naturally occurring feline hypothyroidism in adult cats presented over a one‐year period to three small‐animal clinics.

All staff members of two university hospitals and one private referral clinic were asked to search for cats aged older than one year with clinical signs suggestive of feline hypothyroidism (obstipation, obesity/weight gain, lethargy, skin changes) or azotemia without evident underlying cause and to proceed with endocrine work‐up in case of low or low normal total T4 (TT4) concentrations. Additionally, all TT4 measurements performed during the one‐year study period were monthly screened for low TT4 blood levels to identify possible additional patients. The continuative diagnostic work‐up consisted primarily of thyroid‐stimulating hormone (TSH) measurements using leftover samples and TT4 reevaluation. The TSH stimulation test or thyroid scintigraphy was recommended in doubtful cases.

A low to low‐normal TT4 was observed in 46% of 1382 cats. Of these, 40% had a TT4 concentration <15 nmol/L and 60% between 15 and 24 nmol/L. Of all cats, we identified 44 cats that were suspicious of hypothyroidism. Furthermore, 53 cats with azotemia had low or low‐normal TT4 concentration measurements and were further evaluated for hypothyroidism. Two cats with high suspicion of hypothyroidism underwent a TSH stimulation test or scintigraphy as part of their diagnostic work‐up. Hypothyroidism was excluded in both cats. Common underlying causes for low or low‐normal TT4 included acute non‐thyroidal illness (35.1%), hyperthyroidism under treatment (24.6%), and treatment with drugs known to reduce TT4 concentrations (15.1%). 22.4% of cats showed neither clinical signs suspicious for hypothyroidism nor azotemia and were excluded from further work‐up. In 17 cats (2.7%), no follow‐up was possible due to euthanasia or owners rejecting further work‐up. Hypothyroidism was diagnosed in one cat with a low TT4 and in none of the cats with a TT4 within the reference limits.

The results of this study suggest that naturally occurring spontaneous hypothyroidism is extremely rare in the Central European cat population. Supplementary TSH measurements can thus be restricted to cases with high clinical suspicion.

**Disclosures**

**Source of Funding**

**ECVIM CSF Funding**

No.

**ESVE**—**European Society of Veterinary Endocrinology**

## ESVE‐P‐12

## Influence of the owner's personality and other owner‐, cat‐ and treatment‐related factors on the perception of the quality of life for cats with hyperthyroidism

### S. Muthmann^1^, I. Schofield^2^, M. Kersting^3^, F. Blunschi^4^, N. Bauer^4^, K. Hazuchova^4^

#### ^1^Small Animal Clinic Justus‐Liebig University Giessen Departement of Veterinary Clinical Sciences, Giessen, Germany; ^2^CVS (UK) Ltd, Norfolk, United Kingdom; ^3^Department of Psychology, Giessen, Germany; ^4^Small Animal Clinic Justus‐Liebig University Giessen, Giessen, Germany

Assessment of quality‐of‐life (QoL) is becoming increasingly important in veterinary medicine. In human medicine, it is known, that QoL assessment might be affected by assessor's personality. The aim of this study was to evaluate the impact of owner personality and other owner‐, cat‐ and treatment‐related factors on the health‐related QoL (HRQoL) of hyperthyroid cats, assessed using a previously validated HyperthyroidismQoL‐cat tool.

The HyperthyroidismQoL‐cat was made available online (April 2023–February 2024) in German and English alongside validated questionnaire Big Five Inventory‐2 (BFI‐2) used to measure 5 personality domains (extraversion, agreeableness, conscientiousness, negative emotionality, open‐mindedness; scores ranging 1–5) to be completed by owners of hyperthyroid cats. Socio‐demographic information about the owner, cat's signalment and treatment‐related data were collected. Univariable and multivariable linear regression modelling were used to assess associations between owner personality scores and other factors and cat's HRQoL score. Risk factors with a broad association with HRQoL during univariable analysis (*p*<0.2) were considered for multivariable evaluation. Significance was *p*<0.05.

500 owner‐cat pairs were available for analysis. 470/500 (94%) of the owners were female, most common age category was >50 years old (220/500; 44%). The personality scores (mean ± standard deviation) were 3.1 ( ± 0.69), 3.79 ( ± 0.48), 3.54 ( ± 0.6), 2.91 ( ± 0.66) and 3.63 ( ± 0.72) for extraversion, agreeableness, conscientiousness, negative emotionality and open‐mindedness, respectively, and were in line with the published norm values for BFI‐2. 250/500 (50%) cats were 11–14 years old, 245/500 (49%) were female, 338/500 (68%) were Domestic Shorthair, 345/500 (69%) were treated with oral antithyroid drugs, 55/500 (11%) underwent radioiodine therapy (RAIT), 6/500 (1%) were fed low‐iodine diet (LID), and 20/500 (4%) received no treatment. The median HRQoL score was 90 (range 3–278).

Univariable modelling revealed conscientiousness (*p* = 0.052), owner ag> (>50 years old; *p* = 0.019) and RAIT (p<0.001) to be negatively associated (meaning lower HRQoL and better QoL) and negative owner emotionality (p<0.001), no treatment (*p* = 0.002) and LID (*p* = 0.015) to be positively associated with HRQoL (meaning higher HRQoL and poorer QoL). The association remained significant for negative owner emotionality (p<0.001), and with cats receiving RAIT (*p* = 0.001), no treatment (*p* = 0.001) and low‐iodine diet (*p* = 0.023) in the multivariable analysis.

This study indicates that negative emotionality (describing the tendency to experience anxiety, fear and other negative emotions) of an owner has an effect on their assessment of their hyperthyroid cat's QoL. RAIT was associated with better QoL, while LID or lack of treatment were associated with poorer HRQoL.

**Disclosures**

Kararina Hazuchova is consultant for Boehringer Ingelheim.

**Source of Funding**

**ECVIM CSF Funding**

No.

**ESVE**—**European Society of Veterinary Endocrinology**

## ESVE‐P‐13

## Cortisol and thyroxine changes in snake envenomation as a model of acute canine critical illness

### L. Fourie‐Viljoen ^1^, A. Goddard^1^, P. Thompson^2^, S. Daminet^3^, J. Schoeman^1^

#### ^1^Faculty of Veterinary Science, University of Pretoria Department of Companion Animal Clinical Studies, Onderstepoort, South Africa; ^2^Faculty of Veterinary Science, University of Pretoria Department of Production Animal Studies, Onderstepoort, South Africa; ^3^Faculteit Diergeneeskunde, Universiteit Gent Vakgroep Kleine Huisdieren, Merelbeke, Belgium

The Hypothalamic‐Pituitary‐Adrenal (HPA) and Hypothalamic‐Pituitary‐Thyroidal (HPT) axes, surrounded by controversy in critical illness endocrinology, are central in the pursuit of homeostasis in critical illness. The changes seen in these axes are archetypal, especially of acute critical illness, and constitute the central and peripheral mechanisms of the “stress response” as physiological entity. Snake envenomations are paradigmatic models of acute inflammatory illness, hallmarked by inflammatory and acute phase responses in murine models. To the authors’ knowledge, no prior veterinary research had been performed on endocrine changes in canine snake envenomation. The goal of this study was to investigate the host endocrine response in the snake‐envenomed canine patient, both as critically ill patient and as distinct clinical grouping.

This prospective, cross‐sectional, observational study consisted of 18 client‐owned dogs naturally envenomed by either a snouted cobra (Naja annulifera) (9 dogs) or a puffadder (Bitis arietans) (9 dogs), that presented within 6 hours of envenomation. Serum samples were collected at admission, and thereafter at 12, 24, and 36 hours post‐envenomation. At each time point, total thyroxine (TT4), thyrotropin (TSH), and cortisol were measured.

The cortisol concentrations of both groups were at their highest at admission. A significantly (P<0.05) higher serum cortisol was noted at admission than at each individual time point thereafter. Interestingly, the rate of increase in cortisol was substantially higher than the rate of decrease. Regarding the HPT‐axis, both the puffadder (*P* = 0.014) and snouted cobra (*P* = 0.002) groups displayed a significant decrease in serum TT4 between admission and 12 hours post‐envenomation, with the suppression being sustained until 24 hours post‐envenomation in puffadder‐envenomed dogs (*P*< 0.001). The patterns in TSH failed to reach statistical significance, indicating possible peripheral mechanisms of thyroid gland suppression. The observed serum cortisol peak was markedly acute when compared to prior studies on experimental canine babesiosis, where the peak occurred at 96 hours post infection, or experimental pneumonia, where peak levels were reached at 24 hours post pulmonary challenge. The TT4 nadir observed was also earlier than that of experimental babesiosis, but later than the milder T4 nadir seen in dog bite wound cases.

This study provides novel insights into the temporal relationship between HPA and HPT perturbations in snake envenomation as a model of acute inflammatory illness. It further contextualises these changes in the broader study of the endocrinology of critical illness, and allows a more granular understanding of the endocrine pathophysiology involved.

**Disclosures**

**Source of Funding**

No additional funding was obtained for this study, as the data was collected for a study performed on other parts of the dataset.

**ECVIM CSF Funding**

No.

**ESVE**—**European Society of Veterinary Endocrinology**

## ESVE‐P‐14

## Demographics of 4,620 cats with presumed hypersomatotropism in the USA and Canada

### B. Petroff^1^, R. Egbert^1^, S.S. Niessen^2^, R. Fowkes^3^

#### ^1^Michigan State University PDI, CVM, Michigan State University, East Lansing Michigan, USA, Lansing, United States; ^2^Royal Veterinary College Royal Veterinary College – Veterinary Clinical Sciences, North Mimms, Herts, Uni, Herts, United Kingdom; ^3^Michigan State University SCS, CVM, Michigan State University, East Lansing Michigan, USA, Lansing, United States

Hypersomatotropism in cats is a disorder of excessive growth hormone and increased IGF‐1 (Insulin like Growth Factor 1). It is usually caused by hyperplasia or neuroendocrine tumors of the pars distalis of the pituitary gland. This retrospective study used serum IGF‐1 results to investigate the population characteristics for domestic cats with hypersomatotropism in the United States and Canada. Laboratory records from 1 Jan 06 to 31 Dec 22 were reviewed and animals with a circulating IGF‐1 hormone concentration of 190 nmol/L (1,450 ng/mL) or greater were presumed to have hypersomatotropism (acromegaly). This review identified 4,620 such cats with a mean age (*n* = 4,351) of 11.19 years (median: 11.2 y, range: .5–20.3 yr) and mean weight (*n* = 2,086) of 6.03 Kg (median: 5.91 Kg, range: 2.27–15.0 Kg). Males outnumbered females in the database, which included 3,048 males and 1,181 females. Domestic Short/Medium/Longhair cats were the most prevalent breed (*n* = 3,563; 77%), followed by Maine Coon cats (*n* = 120; 2.6%) and Siamese cats (*n* = 111, 2.4%). Just under half of the animals (*n* = 2,037, 44%) had a history of diabetes mellitus on the submission form. Most submissions were from the US (*n* = 4,347, 94%) with the most hypersomatotropic cats from California (*n* = 841, 18.2%), New York (*n* = 604, 13.1%), Massachusetts (*n* = 402, 8.7%), Colorado (225, 4.9%), and Texas (200, 4.3%). The mean serum IGF‐1 concentration of these animals was 358 nmol/mL (median: 345, range 190–761 nmol/mL). For comparison, an age and sex matched control group of 95 animals with residual samples previously submitted for unrelated testing was selected. The mean IGF‐1 concentration for the control group was 97 nmol/mL (median: 85, range 0–311 nmol/mL). In North America, hypersomatotropism is diagnosed most frequently in older male cats.

**Disclosures**

Egbert and Petroff are both employees of the MSU Veterinary Diagnostic Laboratory where testing was performed.

**Source of Funding**

MSU College of Veterinary Medicine.

**ECVIM CSF Funding**

No.

**ESVE**—**European Society of Veterinary Endocrinology**

## ESVE‐P‐15

## Presenting signs, diagnostic testing and clinical management in 375 diabetic cats under UK primary veterinary care

### O. Waite^1^, E. Wright^1^, R. Gostelow^1^, R. Jepson^1^, D. O'Neill^1^

#### ^1^Royal Veterinary College, London, United Kingdom

Approximately 1‐in‐200 cats in the UK are living with diabetes mellitus (DM) but limited information describes their initial assessment, clinicopathology and management under primary veterinary care (PVC). This study aimed to describe presenting clinical signs, diagnostic testing and management strategies in cats following diagnosis of DM under UK PVC between 1st January and 31st December 2019.

From a VetCompass™ sampling frame of 1,255,130 cats under PVC in 2019, automated searching of electronic health records (EHR) identified 14,321 candidate cases. Manual EHR review of a random selection of 3,047 candidate case confirmed 375 DM cases first diagnosed during 2019. Presenting clinical signs reported ≤ 30‐days prior to diagnosis, laboratory diagnostic tests performed ≤ 7‐days either side of diagnosis and all antihyperglycemic treatments prescribed were extracted until the study period end.

Mean age at diagnosis was 11.8 years (standard deviation [SD] ± 3.5, *n* = 371); 61.9% (232/375) were male cats. Median adult bodyweight was 5.9 kg (interquartile range [IQR]; 4.6–7.1; range: 1.5–14.5 kg). The most commonly reported clinical signs were: polydipsia (63.5%, 238/375), weight loss (58.9%, 221/375) and polyuria (39.5%, 148/375). Hindlimb weakness (6.4%, 24/375) and collapse (1/1%, 4/375) were the least frequently reported signs.

At least one laboratory diagnostic test was performed in 98.7% (370/375) of cases before antihyperglycemic treatment. Mean blood glucose concentration was 25.0mmol/L (SD ± 5.24; *n* = 268). Median fructosamine concentration was 523.μmol/l (IQR: 447.0, 599.5; range: 329.0–909.0; *n* = 92). Urinalysis results were available to review (57.6%, 216/375); glucosuria was recorded (89.3%, 193/261) of cases. Insulin like growth factor‐1 (3.2%, 12/375) and serum ketones (1.2%, 13/375) were measured.

Antihyperglycemic medications were prescribed (82.7%, 310/375) of cases. Insulin was prescribed (96.8%, 300/310) to treated cats including lente insulin (63.2%, 196/310), protamine zinc insulin (PZI [31.9%, 99/310]) and glargine (0.3%, 1/300); insulin was administered (1.7%, 5/300) but the type was unrecorded. Glipizide was prescribed (3.3%, 10,310) to treated cats. Antihyperglycemic medications were not prescribed (17.3%, 65/375) to diabetic cats.

Diet was changed (28.0%, 105/375) in cats following DM diagnosis; 66.7% (70/105) of cats received prescription diabetic diets.

Euthanasia occurred within 72 h of first DM diagnosis in 12.0% (45/375) of cats; 84.4% (38/45) of these cats had not received any DM‐specific therapy.

Euthanasia without treatment occurs frequently in cats following diagnosis of DM. Lente insulin was most commonly prescribed, despite a long‐acting insulin being licenced for use in diabetic cats in the UK.

**Disclosures**

**Source of Funding**

**ECVIM CSF Funding**

No.

**ESVE**—**European Society of Veterinary Endocrinology**

## ESVE‐P‐16

## New practical insights in the management of hypoadrenocorticism in dogs

### C. Emming^1^, A. Geks^2^, S. Sajadihezaveh^3^, S. Rösch^1^, T. Rieker^4^, H. Volk^5^, J. Rieder^5^

#### ^1^Small Animal Clinic, University of Veterinary Medicine Hannover, Foundation Internal Medicine, Hannover, Germany; ^2^Small Animal Clinic, University of Veterinary Medicine Hannover, Foundation Internal Medicine, Hannover, Germany; ^3^AniCura small animal specialists Augsburg GmbH Internal Medicine, Augsburg, Germany; ^4^AniCura Germany GmbH, Ravensburg, Germany; ^5^Small Animal Clinic, University of Veterinary Medicine Hannover, Foundation, Hannover, Germany

In canine hypoadrenocorticism (HA), comprehensive data regarding various glucocorticoid types and dosages for acute and long‐term therapy remains scarce. To address this gap, we developed an online questionnaire tailored for veterinarians, centering on diverse dosing protocols. This questionnaire encompassed multiple‐choice and open‐ended inquiries, targeting cases of HA diagnosed in dogs between 2016 and 2023.

The results of 103 completed questionnaires were analysed, accounting for 54 male and 49 female dogs (median age 5.4 years; range 0.4–13.3). 14 of 103 dogs were initially eunatremic and eukalemic. 82.5% (85/103) of the dogs were hospitalised for at least 24 hours. During hospitalisation, 45 dogs (52.9%) received a prednisolone derivative, 26 dogs (31.8%) dexamethasone, and 10 dogs (11.8%) hydrocortisone. Dogs treated with hydrocortisone had a statistically significant shorter hospitalization time (*p* = 0,0115). After discharge, all dogs with available follow‐up data (85/103) who were further treated with a glucocorticoid, received prednisolone. Single (18.9%) or repeated (78.4%) prednisolone dosage reductions were adjusted to the patients’ need. The median prednisolone dose was 0.13 mg/kg (range: 0–1 mg/kg, *n* = 79). There was no significant difference for prednisolone dose in regards to receiving mineralocorticoids, nor to the initial type of glucocorticoid. Overall, 47.6% of dogs with HA were optimally adjusted according to their quality of life and side‐effects’ profile. One dog died due to an Addisonian crisis during long‐term treatment.

In conclusion, HA is associated with a good prognosis in this study population. Treatment with hydrocortisone appears to lead to shorter hospitalisation times and should be investigated in future studies.

**Disclosures**

**Source of Funding**

**ECVIM CSF Funding**

No.

**ESVE**—**European Society of Veterinary Endocrinology**

## ESVE‐P‐17

## Hypoadrenocorticism in dogs presenting to a tertiary veterinary clinic in Switzerland: Overall prevalence, presenting complaints, laboratory changes and outcome

### J. Eiermann^1^, B. Lutz^1^

#### ^1^University of Bern—Small Animal Internal Medicine, Bern, Switzerland

Hypoadrenocorticism is a relatively uncommon endocrinological disease and dogs often present with non‐specific clinical signs.

Medical records were retrospectively searched for dogs diagnosed with hypoadrenocorticism (HAD) between March 2015 and March 2023. Diagnosis of HAD was based on abnormal results of an adrenocorticotropic hormone stimulation test (ACTH‐ST). Dogs with resting serum cortisol values ≥2 ug/dl or stimulated cortisol values ≥2 ug/dl were categorized as not suffering from HAD.

A total of 54 dogs were diagnosed with HAD, which corresponds to 9.6% of all dogs screened for HAD (*n* = 564). Dogs without HAD had significantly higher sodium (*p*<0.001), lower creatinine (*p* = 0.007), and lower phosphorus (*p*<0.001) concentrations than dogs with HAD. Most common dog breeds diagnosed with HAD were mix breeds (*n* = 16), Jack Russel Terriers (*n* = 5), Bernese Mountain Dogs (*n* = 3), followed by Cairn Terrier, Poodle, Chihuahua, Brittany, Irish Soft Coated and West Highland White Terriers (*n* = 2 each). Median age at diagnosis was 5 years (IQR: 3 months–14 years). Twenty‐nine were male, 26 dogs were female. Twenty‐four of 54 dogs (44.4%) initially presented with clinical signs compatible with an HAD crisis. Only two of 55 dogs (3.7%) had resting cortisol values > 0.5ug/dl (max. 0.63) and six of 54 dogs (11.1%) had post‐ACTH‐ST cortisol values > 0.5ug/dl (median: 0.82ug/dl, IQR: 0.51–0.96). Forty‐three dogs (79.6%) had typical HAD (concurrent mineralo‐ and glucocorticoid‐deficiency) and the remaining 11 dogs had eunatremic, eukalemic hypoadrenocorticism (EEH). Most common presenting complaints were combinations of hypo‐/anorexia (*n* = 37, 68.5%), apathy (*n* = 35, 64.8%), vomiting (*n* = 31, 57.4%), followed by diarrhea (*n* = 12, 22.2%), weight loss (*n* = 10, 18.5%) and polyuria and polydipsia (*n* = 9, 16.7%). Dogs with EEH had significantly lower phosphorus (p<0.001), calcium (*p* = 0.005), albumin (*p* = 0.002), urea (*p* = 0.003), and creatinine concentrations (*p* = 0.001). Dogs with EEH also had significantly lower hemoglobin (*p* = 0.045), mean corpuscular hemoglobin concentration (MCHC) (p<0.001), and higher red cell distribution width (RDW) (*p* = 0.008). Of the HAD dogs, two were euthanized and one dog died during treatment (in‐house mortality of 5%). One dog with EEH developed typical HAD seven weeks after initial diagnosis, representing a prevalence of nine percent.

This study describes prevalence, typical presenting complaints, laboratory changes, and short‐term outcome of dogs diagnosed with HAD in a tertiary veterinary clinic in Switzerland. The prevalence of EEH was similar to those previously reported literature. In contrast to previous reports, females were not overrepresented compared to males, and there was no significant difference in age at presentation between typical HAD and EEH.

**Disclosures**

**Source of Funding**

**ECVIM CSF Funding**

No.

**ESVE**—**European Society of Veterinary Endocrinology**

## ESVE‐P‐18

## Survey about management of diabetes mellitus in dogs and cats

### T. Jaresova^1^, N. Bauer^2^, K. Hazuchova^1^

#### ^1^Small animal clinic – internal medicine, Justus‐Liebig‐University, Giessen, Germany; ^2^Central laboratory, Justus‐Liebig‐University, Giessen, Germany

Diabetes mellitus (DM) is one of the most common endocrinopathies presented to veterinarians in first opinion or referral practices. Although guidelines for DM management exist, a number of factors might influence their implementation in the practice. The aim of this study was to obtain information on veterinarians' experience regarding management of diabetic pets across Europe.

A questionnaire consisting of 2 parts (part 1 – DM management [28 questions]; part 2—demographic information about the veterinarian [practice type, location, etc.; 9 questions]) was made available online in 3 languages (English, German, Czech) to be completed by veterinarians from May 2022 to February 2024. The data is presented as the most frequent response to each questionnaire item, with the number (%) of veterinarians in brackets.

In total, 488 veterinarians completed the questionnaire; 256 in English, 176 in German and 56 in Czech language. Most respondents worked in first opinion practice (*n* = 396/488, 81.1%) and their caseload consisted of over 90 % dogs and cats (*n* = 402/488, 82.4%).

Most veterinarians diagnosed DM in 2–4 dogs (*n* = 206/488, 42.2%) and 2–4 cats (*n* = 250/488, 51.2%) per year. In both dogs and cats, most respondents recommended twice daily insulin injections (*n* = 398/488 [81.6%] and *n* = 440/448 [90.2%], respectively) alongside weight reduction (*n* = 348/488 [71.3%] and *n* = 373/488 [76.4%], respectively). The preferred first‐line insulin was Caninsulin in dogs (*n* = 455/488, 93.2%) and Prozinc in cats (*n* = 203/488, 41.6%). The preferred monitoring method were blood glucose curves (*n* = 216/488, 44.2%). The first re‐check following insulin treatment start was mostly done after 1–2 weeks (*n* = 313/488, 64.1%), well‐controlled patients were usually re‐checked every 3 months (*n* = 225/488, 46.1%). Most veterinarians reported remission in <1 of 10 cats (*n* = 160/488, 32.8%) and <1 of 10 female dogs following castration (*n* = 251/488, 51.4%). The most common comorbidity was urinary tract infection in both dogs and cats (*n* = 285/488 [58.4%] and *n* = 255/488 [52.2%], respectively). Poor owner compliance and the presence of comorbidities were considered the main challenges in DM management by 220/488 (45.1%) and 179/488 (36.7%) veterinarians, respectively. Difficulty in keeping regular daily schedule was considered the main hurdle for diabetic pet owners by 234/488 (48%) veterinarians. Nevertheless, most owners were willing to start treatment and only 80/488 (16.4%) respondents for dogs and 104/488 (23.4%) for cats reported, that >1 in 10 patients are euthanised after DM is diagnosed.

Our results suggest that management of diabetic pets in Europe mostly corresponds with DM management guidelines, although challenges preventing their full implementation exist.

**Disclosures**

Katarina Hazuchova is consultant for Boehringer Ingelheim.

**Source of Funding**

No funding.

**ECVIM CSF Funding**

No.

**ESVE**—**European Society of Veterinary Endocrinology**

## ESVE‐P‐19

## Pharmacodynamics of dapagliflozin in dogs using an experimental model of metabolic syndrome

### J. Mochel^1^, T. Blondel^2^, D. Kundu^3^, E. Guillot^2^, J. Ward^3^, K. Allenspach^4^

#### ^1^The University of Georgia Pathology, Athens, United States; ^2^Ceva Santé Animale, Libourne, France; ^3^Iowa State University, Ames, United States; ^4^The University of Georgia, Athens, United States

Evidence gathered from multiple placebo‐controlled studies suggests that sodium‐glucose transporter 2 (SGLT‐2) inhibitors have a favorable influence on systemic metabolism, regardless of the patient's diabetic status. This experimental study was divided into two phases. The first phase aimed to demonstrate the ability of a Western diet (WD) model to emulate key biological characteristics of Metabolic Syndrome (MetS) in canines. The second phase aimed to examine the pharmacodynamic effects of administering a low dose (LD) of 0.3 mg/kg and a high dose (HD) of 1.0 mg/kg of the SGLT‐2 inhibitor dapagliflozin (DAPA) to dogs with WD‐induced MetS.

In a first study period, MetS was initiated in eighteen healthy adult Beagle dogs who were fed an isocaloric WD for ten weeks. Biological specimens were collected at the beginning (BAS1) and the end (BAS2) of the ten‐week feeding period. The WD model resulted in significant changes in various markers of MetS, such as elevated blood pressure (*P* = 0.017), increased fasting glucose levels (*P*<0.001), and decreased HDL‐cholesterol levels (*P*<0.001). Additionally, the model induced an increase in circulating NT‐proBNP levels (*P*<0.001) and significant changes in general metabolic processes, lipids, and biogenic amine levels.

In a subsequent period, dogs were randomly assigned to three study groups: (1) Control (CTRL, *n* = 6), (2) DAPA LD (*n* = 6), and (3) DAPA HD (*n* = 6) (Phase II). DAPA tablets were administered orally once a day. Blood samples were taken at the first dose (DOS1) of DAPA and at steady state (DOS21). Urinary catheterization was also performed to determine the total volume of urine produced and the amount of glucose excreted over a 24‐hour period. Variations from BAS2 among the treatment groups were analyzed using non‐parametric statistics.

Overall, DAPA caused an increase in circulating insulin levels compared to CTRL after DOS1 (LD, *P* = 0.026; HD, *P* = 0.061), along with a decrease in fasting blood glucose levels at DOS21 (LD, *P* = 0.005; HD, *P* = 0.018). Additionally, 24‐h urine volume significantly increased in DAPA‐treated dogs compared to CTRL (LD, *P* = 0.008; HD, *P* = 0.047), as did the amount of glucose excreted in urine over 24 hours (LD, *P* = 0.007; HD, *P* = 0.07). No significant differences were observed between the effects of the two doses of dapagliflozin.

In conclusion, feeding of WD successfully induced evidence of MetS in healthy dogs. Our findings demonstrated a significant impact of DAPA on glucose homeostasis, resulting in reduced levels of circulating blood glucose and increased urinary glucose excretion and 24‐hour urine production.

**Disclosures**

Mochel, Ward, and Allenspach are consultant for Ceva Sante Animale. Blondel and Guillot are employees of Ceva Sante Animale.

**Source of Funding**

Ceva Sante Animale.

**ECVIM CSF Funding**

No.

**ESVCN**—**European Society of Veterinary & Comparative Nutrition**

## ESVCN‐P‐1

## Adult dogs maintain body lean consuming food containing 0.59%/kg total sulfur amino acids

### D. Jewell^1^, E. Morris^2^, J. Pezzali^3^, L. Hancock^4^

#### ^1^Kansas State University, Manhattan, United States; ^2^Hill's Pet Nutrition Clinical Studies and Claims, Topeka, United States; ^3^Kansas State University Department of Grain Science and Industry, Manhattan, KS, United States; ^4^Hill's Pet Nutrition Clinical Studies and Claims, Topeka, KS, United States

Dietary protein is often oversupplied in commercial dog foods partially because of limited information of protein and indispensable amino acid requirements for adult dogs. However, this oversupply of protein is not only environmentally expensive but can lead to an imbalance and/or excess of certain amino acids, which may be detrimental to health. Therefore, this study aimed to evaluate the minimum concentration of indispensable amino acids necessary to maintain health and lean body mass in adult dogs. Seventy Beagles were used in a completely randomized design where the effects of five dietary levels of protein (16.0%, 18.9%, 22.54%, 24.3% and 30.2% dry matter basis (DMB)) with or without synthetic amino acid supplementation (methionine, lysine, tryptophan and threonine) on body composition and selected blood metabolites were tested. Experimental diets had approximately 4,000 kcal/kg, calculated using modified Atwater factors. All food contained non‐amino acid nutrients in excess of minimum requirements. The non‐supplemented experimental diet with 16% CP was probably most limiting in total sulfur amino acids (TSAA; 0.59% DMB) followed by lysine (0.63% DMB). Blood analysis for urea nitrogen, albumin, hematocrit and hemoglobin, and body composition from dual energy x‐ray absorptiometry (DXA) were performed at baseline and at the end of the 6 months feeding trial, where dogs were fed to maintain body weight. Data were analyzed with the mixed procedure in SAS 9.4 using treatment as a fixed variable and animal ID as a random variable. Blood values were maintained within reference ranges regardless of dietary treatment. There was a quadratic change (*P* =0.06) in the concentration of blood urea nitrogen with increasing (*P* < 0.05) concentration in the dogs consuming the 30.2% protein food. There was no effect (*P* > 0.05) of dietary protein level or amino acid supplementation on circulating concentrations of hemoglobin, hematocrit or albumin. There were no differences in initial or final body lean or fat mass as measured by DXA among dietary treatments, and no linear or quadratic effect of dietary protein concentration on initial or final body lean or fat mass (*P* > 0.05). However, there was a numeric average increase in body lean mass in all treatments. In conclusion, all dietary treatments supplied sufficient dietary intake of indispensable amino acids for maintaining lean body mass in adult dogs, with the diet most limiting in protein and amino acids having a concentration of 16% protein, 0.59% TSAA and 0.63% lysine on DMB.

**Disclosures**

DEJ and JGP have grant proposals pending with Hill's Pet Nutrition, Inc. DEJ has previously been employed by Purina and Hill's Pet Nutrition, Inc. EMM and LBH are current employees of Hill's Pet Nutrition, Inc.

**Source of Funding**

Hill's Pet Nutrition, Inc.

**ECVIM CSF Funding**

No.

**ESVCN**—**European society of Veterinary & Comparative Nutrition**

## ESVCN‐P‐2

## Modulation of the canine gut microbiota with prebiotic, probiotic, and postbiotic supplementation as assessed in the Mucosal Simulator of the Canine Intestinal Microbial Ecosystem

### G. Pignataro^1^, L. Clerico^2^, B. Belà ^3^, A. Gramenzi^3^

#### ^1^Department of Veterinary Medicine, University of Teramo Department of Veterinary Medicine, University of Teramo, Italy, Teramo, Italy; ^2^Consultant in medical writing, Savona, Italy; ^3^Department of Veterinary Medicine, University of Teramo, Teramo, Italy

The Simulator of the Canine Intestinal Microbial Ecosystem (SCIME^TM^) was recently developed and validated to provide a proper platform to analyze the effects of nutritional interventions on the canine gut microbiota.The present study aims to evaluate the impact of *Lactobacillus reuteri* probiotic supplementation (NBF1), alone or in combination with Microbiotal (prebiotics and bacterial fermentation products), on the composition and metabolic activity of healthy canine donors' luminal and mucosal gut microbiota.

The experiments were conducted using the SCIME^TM^platform.The effect of the supplementations was evaluated using the microbiota of a healthy ± 20 kg canine donor. After inoculation of the reactors with the fecal sample, the experiment followed three steps where the SCIME^TM^was supplemented with *L. reuteri* or *L. reuteri* and Microbiotal. Both the effects of the supplementations were evaluated using the 16S‐targeted Illumina sequencing and metabolic activity (acid/base consumption and concentration of SCFA, lactate, ammonium, and branched SCFA) of the luminal and mucosal gut microbiota.

The treatment with *L. reuteri* stimulated lactate production, alone or in combination with Microbiotal. In contrast with the results previously observed with Microbiotal treatment alone, probiotic supplementation reduced acetate and propionate levels compared to controls. Concerning markers for proteolytic fermentation, a trend towards increased ammonium and branched SCFA levels was observed in the distal colon following *L. reuteri* treatment, both in the presence or absence of Microbiotal. The *Limosilactobacillus* genus was strongly enriched following supplementation with the probiotic, independently of co‐supplementation with Microbiotal, especially in the proximal colon.Furthermore, treatment with *L. reuteri* significantly enriched the *Pseudomonas*, *Stenotrophomonas*, and *Faecalibacterium* genera in the proximal colon compared to the control. Interestingly, next to the stimulation of *Limosilactobacillus*, significant enrichment of *Bifidobacterium* was observed in the distal colon upon treatment with the combinatory supplementation.

The SCIME^TM^experiment displayed that *L. reuteri* supplementation promoted significant changes in microbial community fermentation patterns. Following probiotic treatment, the RNA gene profiling highlighted the activation of cross‐feeding interactions in the canine microbiome. Moreover, significant metabolic differences were observed between the proximal and distal colon regions. In the proximal colon, a significant metabolic shift was due to treatment with pure *L. reuteri* and in co‐supplementation with Microbiotal. In the distal colon, a significant impact was revealed upon supplementation with the probiotic engrafted with Microbiotal. This work points out the ability of a feed containing probiotics to improve the modulatory effects on the canine gut microbiota, especially when coauthored with prebiotics and postbiotics products.

**Disclosures**

**Source of Funding**

**ECVIM CSF Funding**

No.

**ESVCN**—**European society of Veterinary & Comparative Nutrition**

## ESVCN‐P‐3

## Quality of life supported in cats with malignant cancer with a new high calorie, nutrient dense food

### M. Amundson^1^, L. Motsinger^1^, I. Becvarova^1^

#### ^1^Hill's Pet Nutrition, Inc., Topeka, United States

In cancer patients, one of the main goals is to maintain the best possible quality of life, regardless of malignancy type or treatment, especially during palliative care. Poor quality of life perceived by the owner is a common reason for euthanasia in pets with cancer. The study objective was to evaluate feeding a new diet to support cats with malignant cancer.

This single‐arm, non‐controlled, prospective study consisted of 8 cats diagnosed with malignant cancer and fed Hill's Prescription Diet ON‐Care Feline dry for a 70‐day period with a 14‐day transition. Intake (grams and calories), body weight (BW), body condition score (BCS; 1=under ideal, 9=over ideal), muscle condition score (MCS; 1=normal, 4=severe loss), eating enthusiasm (1=lowest, 7=highest), stool quality (1=liquid, 6=firm), grimace score (0=absent, 2=markedly present), and quality of life (QoL; 5‐point scale) were recorded. Average age, BW, BCS, MCS, grimace score, and eating enthusiasm at day 0 were 12.2 y, 4.1 kg, 4.8, 1.9, 1.5, and 4.0, respectively. This study was approved by the Institutional Animal Care and Use Committee.

Gram and caloric intake were unchanged from day 15 to day 70. Average eating enthusiasm numerically increased (*P* > 0.10) from day 0 (4.0) to day 70 (5.3). Numerical changes such as increased BW (0.2 kg, SE=0.2, P<>i>>0.10), decreased BCS (‐0.3, SE=0.4, >0.10), improved MCS (‐0.4, SE=0.2,>*P* > 0.10), and improved grimace score (‐0.5, SE=0>5, *P* > 0.10) were observed from day 0 to day 70. Normal stool score was observed throughout the study. Owners reported cats maintained QoL throughout the study with numerical improvements in healthy appetite (0.5, S>=0.3, *P* > 0.10), appear bright and alert (0.6> SE=0.2, *P* > 0.10), appear to have low‐energy (‐>.5, SE=0.3, *P* > 0.10), and general health> (0.6, SE=0.4, *P* > 0.10) from day 0 to day 70.

Cats fed Hill's Prescription Diet ON‐Care dry maintained BW, BCS, MCS, grimace score, and eating enthusiasm throughout the study. Overall, this study demonstrated acceptance of a new study food and maintained QoL in cats with malignant cancer; however, recruitment of more cats is necessary to assess the true magnitude of the study food benefits.

**Disclosures**

All authors are employees of Hill's Pet Nutrition, Inc.

**Source of Funding**

This study was funded by Hill's Pet Nutrition, Inc.

**ECVIM CSF Funding**

No.

**ESVCN**—**European society of Veterinary & Comparative Nutrition**

## ESVCN‐P‐4

## A novel investigation into the impact of probiotic dietary supplementation on the metabolic activities of the gut microbiota of a healthy canine donor

### G. Pignataro^1^, L. Clerico^2^, B. Belà ^3^, A. Gramenzi^3^

#### ^1^Department of Veterinary Medicine, University of Teramo Department of Veterinary Medicine, University of Teramo, Italy, Teramo, Italy; ^2^Consultant in medical writing, Savona, Italy; ^3^Department of Veterinary Medicine, University of Teramo, Teramo, Italy

The probiotic *Lactobacillus reuteri* NBF 1® (NBF Lanes S.r.l., Milan, Italy) is a dietary supplementation with a proven safety and efficacy profile for dogs. *L. reuteri*'s ability to improve the composition of beneficial species of the intestinal microflora has already been demonstrated.

This study aimed to investigate the impact of *L. reuteri* NBF 1® also at the level of the metabolic activity of canine gut microbiota.

The effect of *L. reuteri* supplementation was evaluated using the microbiota of a healthy ± 20 kg canine donor. The SCIME^TM^platform, which consists of three temperature‐controlled reactors representing the canine gastrointestinal tract, was utilized. The experiment was divided into a three‐week stabilization period, a three‐week control period, and a two‐week treatment period where the regular diet was supplemented with *L.reuteri*. The probiotic impact on the gut microbiota composition was evaluated using the 16S‐targeted Illumina gene sequencing. The metabolic activity changes were displayed by continuously monitoring the acid/base consumption, the concentration of short‐chain fatty acids (SCFA), lactate, ammonium, and branched SCFA of the luminal and mucosal gut microbiota. Shifts in metabolic activity were also investigated through LA‐REIMS‐based analysis (laser‐assisted ambient ionization mass spectrometry) that provides metabolic fingerprints.

Following LA‐REIMS analysis, metabolic fingerprints were generated that comprised 1603 metabolic features. Based on these fingerprints, metabolic differences were observed between the proximal and distal colon regions. *L. reuteri* promoted statistically significant metabolic activity changes in the proximal colon region. The probiotic treatment stimulated lactate production while reducing acetate and propionate levels compared to controls, indicating a relevant shift in microbial fermentation patterns. With respect to markers for proteolytic fermentation, a trend towards increased ammonium and branched SCFA levels was observed in the distal colon following *L. reuteri* supplementation.

Concerning the gut microbiota composition, the probiotic supplementation confirmed the *Limosilactobacillus* genus enrichment, as well as it happened for *Pseudomonas*, *Stenotrophomonas*, and *Faecalibacterium* genera compared to the control.

This study has uncovered the significant ability of *L. reuteri* NBF 1® to modulate the gut microbiota metabolic activity. These findings provide insights into this probiotic's mechanisms of action and suggest potential applications in certain physiological or pathological conditions. Furthermore, they can contribute to the development of effective supplementation treatment combinations.

**Disclosures**

**Source of Funding**

**ECVIM CSF Funding**

No.

**ESVCN**—**European society of Veterinary & Comparative Nutrition**

## ESVCN‐P‐5

## Urine dipstick tests to evaluate urine pH in dogs and cats

### M.E. Gonçalves Tozato^1^, S. de Souza Theodoro^1^, A. de Castro^1^, V.H. Bernardes Nga^1^, A. Cavalieri Carciofi^1^

#### ^1^São Paulo State University, Jaboticabal, Brazil

Assessment of urine pH is part of urine analysis and necessary for the diagnosis and monitoring of urolithiasis in dogs and cats, directing nutritional interventions to modulate urine pH. Despite being common in clinical practice due to their practicality and affordable cost, urine dipstick tests may not be accurate enough to monitor urine pH. We aimed to compare the accuracy and reliability of four strips brands and a portable pH meter with a precision benchtop pH meter.

Four urine pH reagent strips commonly used in Brazil (Veterinary – Uriquest Plus Vet; Human 1 – Uriquest Plus, Human 2 – Uricolor Check; Human 4 – Senesi‐10) and a portable pH meter (AK90, AKSO Electronic) were compared with a benchtop pH meter (Digimed DM‐22, Digicrom Analítica, São Paulo, Brazil). A total of 96 urine samples (48 from cats, 48 from dogs) were evaluated after spontaneous urination. Results were compared by Bland‐Altman and Pearson correlation, using the benchtop pH meter as reference method (P<0.05).

The mean values obtained for all methods was different than the reference benchtop pH meter (P<0.05). Among urine test strips, the veterinary and human 1 strips presented the highest bias, lowest concordance correlation and accuracy (bias correction factor). The Human 2 and Human 3 strips exhibited both lower bias (0.133 ± 0.33), better concordance correlation, and accuracy of 0.98 and 0.97, respectively. Finally, the portable pH meter provided the closest estimation of urine pH in comparison with the reference equipment, with a low bias (0.03 ± 0.08), highest accuracy (0.99), maximum overreading of 0.12 pH, maximum underreading of 0.21 pH and very high Pearson correlation (ρ 0.99; *P*<0.001). Considering that pH is inversely proportional to H ion logarithm concentration, a difference of only 0.1 implies in a variation of 25% on H ion concentration. Therefore, the high mean bias verified for all strips are physiologically important, and do not support their use for urine pH monitoring. Due this, only the portable pH meter was a potential alternative to estimate urine pH of dogs and cats, although it is important to consider that it results was higher than the obtained in the benchtop pH meter (*P*<0.05).

Although unexpensive and easy to use, none of the tested urine strips were precise, accurate or reliable to estimate urine pH. The portable pH meter tested showed adequate precision and accuracy to estimate urine the pH on the clinical practice of dogs and cats.

**Disclosures**

**Source of Funding**

**ECVIM CSF Funding**

No.

**ESVCN**—**European society of Veterinary & Comparative Nutrition**

## ESVCN‐P‐6

## Effects of *Limosilactobacillus reuteri* DSM 32203 consumption on body weight, body condition score, faecal parameters, and intestinal microbiota of healthy dachshund dogs

### B. Bela' 1, D. Di Simone 2, G. Pignataro 1, I. Fusaro 1, A. Gramenzi 1

#### 1 Department of Veterinary Medicine, University of Teramo, Teramo, Italy; 2 Department of Economics and Finance, University of Bari, Bari, Italy

The ability of probiotics to modulate the intestinal microbiota is already well known, however it should be remembered that the effects of a probiotic are species‐specific, therefore, different strains could manifest different effects. In the literature there are several studies on the effects of different species of lactobacilli (*L. acidophilus*, *L. plantarum*) but very few studies examined the behavior of Limosilactobacillus reuteri on a specific breed of dog. The present study analyzed the effectiveness of the probiotic Limosilactobacillus reuteri DSM 32203, on the intestinal health of healthy dachshund adult dogs by analyzing their bodyweight (BW), body condition score (BCS), fecal score (FS) and fecal moisture (FM). In addition, microbiological analyses were carried out to quantify bacterial species such as *E. coli* (for the total coliform count) and Lactobacilli. The research was conducted according to the directive 2010/63/EU and did not imply any form of animal suffering or health risk, since it focused on the administration of a natural substance. Healthy adult male and female non pregnant (age > 1 year) dogs (*n* = 30, dachshund; 15 females, 15 males: 15 + 15 = 30) were selected for the study and were randomly assigned to the control group (*n* = 15; male: female = 7:8) and to the treated group (*n* = 15; male: female = 8:7). The control group fed the standard diet with the addition of a placebo while the treated group received the probiotic. The study lasted 35 days (+ 14 d acclimatation) in line with the time needed to assess any probiotic effects. BW data showed no differences between the two groups of dogs. However, the fecal moisture was significantly lower at the end of the trial in the treated group and the beneficial effect of Limosilactobacillus reuteri DSM 32203 administration was also confirmed by the FS values recorded among the two groups of dogs. Additionally at the end of the study period, there was a significant increase of Lactobacilli in the treated group compared to the control group (*P*< 0.001). The data collected in this study reported the ability of the probiotic strain L. reuteri DSM 32203 to improve fecal quality parameters such as fecal moisture and fecal quality in healthy dachshund adult dogs showing an increase in Lactobacilli count and a little reduction of total coliforms.

**Disclosures**

**Source of Funding**

**ECVIM CSF Funding**

No.

**ESVCN**—**European society of Veterinary & Comparative Nutrition**

## ESVCN‐P‐8

## Lean and fat mass changes with age in dogs and cats fed a controlled protein, amino acid‐supplemented food are comparable to a previously established baseline

### A. McGrath^1^, L. Hancock^1^, C. Stiers^1^, E. Morris^1^

#### ^1^Hill's Pet Nutrition, Topeka, KS, United States

Feeding a restricted protein diet is a frequently recommended nutritional strategy for dogs and cats with chronic kidney disease (CKD). However, CKD is characterized by an upregulation of proteolytic pathways, and diets formulated with low levels of protein can elevate the risk of lean mass loss and its associated adverse health effects, particularly if inadequate amounts of amino acids are consumed. This study aimed to compare the body composition of dogs and cats fed a controlled protein, amino acid‐supplemented diet for at least 56 days to a previously established normal body composition at each age and life stage. 496 observations from 166 colony‐housed cats aged 3 to 18, and 412 observations from 140 colony‐housed dogs aged 2–17 were included in the study. The study population consisted of animals that were healthy along with those diagnosed with renal disease. Dogs consumed diets ranging from 14% to 20% crude protein on a dry matter basis and essential amino acids at levels at least 120% of the FEDIAF minimum for adult dogs based on an MER of 110 kcal/kg^0.75, while cats consumed diets ranging from 28% to 35% crude protein on a dry matter basis and essential amino acids at levels at least 170% of the FEDIAF minimum for adult cats based on an MER of 100 kcal/kg^0.67. Mean lean and fat mass (cats) or percentages (dogs) was compared to those of a previously established baseline at each age. In cats, both lean and fat mass were higher than or similar to the representative baseline for a majority of ages. In dogs, a majority of both lean and fat percentages were higher than or similar to the representative baseline from ages 8 to 13. The maintenance of lean body mass helps reduce the risk of sarcopenia or cachexia, and these data demonstrate that long‐term consumption of a controlled protein food supplemented with key essential amino acids can help support a normal body composition in dogs and cats compared to an established baseline. Therefore, the health outcome of animals can potentially be improved as a result of this nutritional strategy.

**Disclosures**

Allison P. McGrath, Leslie Hancock, Cheryl A. Stiers, and Elizabeth M. Morris are all employees of Hill's Pet Nutrition, Inc.

**Source of Funding**

This study was funded by Hill's Pet Nutrition, Inc.

**ECVIM CSF Funding**

No.

**ESVCN**—**European society of Veterinary & Comparative Nutrition**

## ESVCN‐P‐9

## A cross‐sectional study of feeding practices in lean and heavy/obese cats

### F. Kragh Jørgensen^1^, M. Tams^1^, C. Mortensen^1^, P. Sandøe^2^, C. Bjørnvad^1^

#### ^1^University of Copenhagen Department of Veterinary Clinical Sciences, Copenhagen, Denmark; ^2^University of Copenhagen Department of Veterinary and Animal Sciences, Copenhagen, Denmark

Being obese may affect feline health and quality of life. With an aim of understanding the nature of the problem, this cross‐sectional study investigates feeding practices among cat owners in relation to the cat's body composition.

One hundred and ninety‐nine privately owned cats (>1 year old and reportedly healthy) were examined at home visits. Cats underwent physical examination, weight measurement and body condition scoring (BCS). Owners answered a questionnaire regarding their feeding management.

Three percent of the cats were underweight (BCS3/9), 34% normal weight (BCS4‐5/9), 31% overweight (BCS6/9) and 35% heavy/obese (BCS7‐9/9). Excluding the underweight cats, cats were divided into two groups normal/mildly overweight (normal, BCS4‐6, *n* = 126) or heavy/obese (heavy, BCS7‐9, *n* = 67). Most cats were fed mainly dry food (normal 86% and heavy 85%) and regular diets for adult cats. Ad libitum feeding was practiced for 43% of heavy and 69% of normal cats (*P* = 00007). When asked how they determined amount to feed, 28% fed when the cat begged, 25% never considered it, 20% adjusted feeding based on perceived weight change, 14% followed recommendations from food packaging or veterinarian and 13% from other sources. Treats were given daily, weekly or less often by 82% of owners of heavy and 69% of owners of normal weight cats. Treats were given in addition to the daily diet allocation by 78% of the owners, 9% reduced the diet allocation accordingly, and 4% used part of the normal diet for treats. There was no difference in frequency of giving treats or relation to the daily diet allocation between groups. Among owners of the heavy cats, 1% categorized their cats as underweight, 42% as normal, 49% as obese and 7% as heavy obese. Forty‐two percent had discussed their cat's weight with their veterinarian, but only 10% fed a weight‐loss diet. Ninety‐eight percent of the owners of heavy cats acknowledged overweight as a health problem in cats and claimed to be willing to change their cat's diet (93%), switch to a weight‐loss diet (85%) or increase their cats activity (82%) if their cat was overweight.

This study reveals a lacking accommodation of feeding practices in relation to cat weight status among cat owners. Despite owners being aware of obesity related health risks, diet regimes are seldom changed, and a large proportion are still fed ad libitum. Even when the cat's weight had been discussed during a veterinary consultation, weight loss diets were seldom implemented.

**Disclosures**

**Source of Funding**

No.

**ECVIM CSF Funding**

No.

**ESVCN**—**European society of Veterinary & Comparative Nutrition**

## ESVCN‐P‐10

## Effect of a high‐protein, high‐fiber diet consumption, weight loss, and the engagement of the tutors on blood metabolite profiles of adult dogs

### A. Loste^1,2^, M. Borobia^3,4^, A. García^5^, L. Navarro^3,4^

#### ^1^Facultad de Veterinaria/Universidad de Zaragoza Departamento de Patología Animal, Zaragoza, Spain; ^2^Instituto Agroalimentario de Aragón – IA2 – (CITA‐Universidad de Zaragoza) Departamento de Patología Animal, Zaragoza, Spain; ^3^Facultad de Veterinaria/Universidad de Zaragoza, Zaragoza, Spain; ^4^Hospital Veterinario de la Universidad de Zaragoza, Zaragoza, Spain; ^5^Facultad de Medicina, Universidad de Zaragoza Departamento de Anatomía e Histología Humanas, Zaragoza, Spain

Obesity is the most common nutritional disease in dogs, causing in poorer quality of life and shorter life expectancy, and its trend is similar to that observed in human medicine. The aim of this study was to evaluate the effect of restricted feeding of a high‐protein and high‐fiber (HPHF) diet and weight loss on body composition, blood metabolites and the involvement of the owner in the success of an individualised weight loss plan. Thirty client‐owned dogs were included and divided in 3 groups according to the Body Condition Score (BCS): ideal weight (IW) (6 females, 4 males; BCS: 4.9 ± 0.74), overweight (OW) (5 females, 5 males; BCS: 6.5 ± 0.75) and obese (OB) (3 females, 7 males; BCS: 8.6 ± 0.51). All dogs were considered healthy except for being overweight and were fed a maintenance diet (4‐week baseline phase) to maintain body weight. After baseline, overweight and obese dogs were fed a HPHF diet for 24‐week weight loss phase. For the initial ration calculation (65–75 kcal ME × Kg^0,75^), the recommendations of The European Pet Food Federation (FEDIAF) were followed. Dogs were monitored every 2‐weeks and appropriate dietary adjustments were made according to weight loss and BCS.

After 4‐week baseline phase, serum triglycerides (TG), cholesterol (CHOL) and calcium (Ca) concentrations were significantly increased (*p*<0.01) in overweight and obese dogs compared to dogs with ideal weight [OW (TG: 102,5 mg/dl), CHOL: 218.25 mg/dl; Ca: 9.78 mg/dl); OB (TG: 126.62 mg/dl; CHOL: 217.89; Ca: 10.13 mg/dl; IW (TG: 57.6 mg/dl; CHOL: 174.3 mg/dl; Ca: 9.45 mg/dl)]. In addition, C reactive protein (CRP) was significantly increased (*p*<0.01) in OB (1.04 mg/dl) compared to IW (0.32 mg/dl) and OW (0.31 mg/dl) groups.

After 24‐weeks of restricted feeding and weight loss, 55.6% of the dogs reached the optimal ICC (87.5% OW, 30% OB) with an average weekly weight loss of 0.5%. Regarding to blood metabolites, TG and CHOL serum concentrations in OW and OB dogs, and CRP level in OB dogs decreased significantly (*p*<0.05; *p*<0.01, respectively).

On the other hand, tutors referred an increase in agility and exercise tolerance of their dogs related to weight loss and BCS improvement.

Our results suggest that restricted feeding of a HPHF diet and weight loss promotes fat mass loss, and reduce inflammatory marker, cholesterol and triglycerides serum concentrations in overweight and obese dogs, improving their quality of life.

**Disclosures**

Nestlé Purina PetCare Company partially funded this research by providing the diet for the dogs.

**Source of Funding**

This study was funded by Línea Estratégica de Investigación (LEI) “ONE HEALTH: Investigación interdisciplinar en salud”, Instituto Agroalimentario de Aragón – IA2 – (CITA‐Universidad de Zaragoza). Nestlé Purina PetCare Company partially funded this research by providing the diet for the dogs.

**ECVIM CSF Funding**

No.

**ESVCN**—**European society of Veterinary & Comparative Nutrition**

## ESVCN‐P‐11

## Nutritional studies on the use of legumes, wheat and quinoa in the diet of dogs

### J. Litzenburger^1^, N. Passlack^2^, E. Saliu^1^, J. Schulze‐Holthausen ^1^, J. Zentek^1^

#### ^1^Freie Universität Berlin, Institute of Animal Nutrition, Berlin, Germany; ^2^LMU München, Chair of Animal Nutrition, München, Germany

Plant‐based food and cereals is sometimes very emotionally discussed by dog owners. Particularly critical are aspects of animal welfare as well as increasingly those of CO_2_ footprint. The suitability of cereals is also questioned, with an increasing demand for "pseudo‐cereals," which do not contain gluten. In this study, 10 Beagle dogs were fed in a cross‐over design with diets that contained poultry or legume protein as a mix of peas and bean concentrate with or without the addition of a cereal (processed wheat flakes) or the “pseudo‐cereal” quinoa. The experimental setup was a three‐factorial design where the first factor represents the carbohydrate, the second the protein component and the third the dietary protein concentration. Protein levels were adjusted to fulfil the needs of dogs for essential amino acids or to 1.5 times the amount of protein and amino acids compared to the maintenance requirement. After a three‐week adaptation phase, the collection of faeces, urine and blood samples took place in the fourth week. Statistical evaluation included three‐factorial ANOVA and Tukey tests (*p* < 0.05). The type of protein in the diet variants influenced (*p* < 0.001) the richness of microbial sequences detected in the faeces. The highest values for richness of the faecal microbiota were observed with diets based on animal proteins. Diets based on animal proteins increased the faecal occurrence of Fusobacteria (*p* < 0.001), while dietary variations with plant proteins favoured the relative occurrence of Actinobacteria (*p* = 0.040). The pH values in the urine of dogs on diets containing wheat and poultry protein (5.73–6.30) were lower compared to diets with quinoa (7.20–7.47). An exception was the combination of wheat with the plant protein source, where a higher pH value was observed (7.42) when administering the higher protein dosage. As expected, urinary nitrogen concentrations increased with higher protein intake. The protein sources led to minor changes in haematological parameters, while serum urea and albumin concentration was significantly influenced by the protein content in the experimental feed (*p* < 0.001; *p* = 0.004). With the use of quinoa, the activity of alanine aminotransferase increased compared to wheat‐containing diets (*p* = 0.017), and higher bilirubin concentrations were also observed (*p* = 0.031). The activity of alkaline phosphatase increased with quinoa compared to wheat‐containing diets (*p* = 0.030) and was lower with a higher protein content in the feed than with an adequate supply (*p* < 0.001).

**Disclosures**

**Source of Funding**

Gesellschaft zur Förderung kynologischer Forschung.

**ECVIM CSF Funding**

No.

**ESVCN**—**European society of Veterinary & Comparative Nutrition**

## ESVCN‐P‐12

## Adverse reactions to food in dogs, what are possible (nutritional) risk factors?

### L. van der Ende, R.J. Corbee

#### Faculty of Veterinary Medicine Utrecht University, Utrecht, Netherlands

The aim of this study was to detect potential risk factors for adverse reactions to food (ARF) in dogs, especially looking at early life nutrition.

An online questionnaire has been distributed on social media (Facebook) to dog owners. Consent was required for participation. Questions were asked about breed, if the diagnosis was made by their veterinarian or not, age at diagnosis, diagnostic procedures, diet history during the first 6 months of life, which part of the diet consisted of raw food, if the dogs had experienced a Giardia infection, if their dogs commonly ate from the street, which allergens their dogs were responding to, and the presence of concurrent allergies

In total, 310 dog owners filled in the whole questionnaire and gave their consent. Doodle‐like dogs were overrepresented (147/310). Only 49/310 dogs were diagnosed by their veterinarian, and 19/310 were currently in the diagnostic process, so the majority (242/310) was self‐diagnosed by the owner. Diagnosis included elimination diets in 35/49, followed by allergy tests in blood (10/49), gastroscopy (3/49), and by kinesiology (1/49). In the first 6 months of life, the part of diet coming from raw food varied from none (133/310), <25% (23/310), >25%<50% (14/310), >50%<75% (34/310), >75%<100% (25/310), and 100% (81/310). Raw feeding thus seems common in this group of dogs (most of them were fed these diets prior to the diagnosis). Giardia infection was diagnosed in 44/310 dogs, suspected in 6/310, and not seen in 260/310 dogs. As Giardia infections are common in puppies, (29% of submitted fecal samples of young dogs with similar symptoms contained Giardia), the question is if Giardia is a true risk factor for AFR. Eating from the street is a common undesirable habit of puppies and was reported in 99/310 dogs, which is similar to the 33‐35% reported earlier in a general population of dogs. Only 44 respondents answered the question to which allergens their dogs responded to. Most common reported allergens in this study were chicken (26/44), beef (23/44), and wheat (8/44), which is similar to previous reports.

As Doodle‐like dogs were overrepresented, it seems like these dogs are predisposed for AFR. Self‐diagnosis by owners is very common. Feeding raw food is common in this group and thus might be a risk factor. Most common allergens are beef and chicken, which is in line with previous studies.

**Disclosures**

**Source of Funding**

**ECVIM CSF Funding**

No.

**ESVCN**—**European society of Veterinary & Comparative Nutrition**

## ESVCN‐P‐13

## Use of cannabidiol (CBD) supplements in small animals practice in Portugal

### M.I. Santos^1^, A.L. Lourenço^1^

#### ^1^University of Trás‐os‐Montes and Alto Douro Animal Science, Vila Real, Portugal

An anonymous online survey questioning the frequency of CBD supplements (CBDS) use, its purposes and the outcomes was developed using Microsoft® Forms. This survey targeted veterinarians currently working in small animal clinics in Portugal.

A total of 150 responses were obtained. Sixteen were excluded due to: rejection of the informed consent (1/150) and veterinarians that were not currently working in small animal practice (15/150). From the responses included (*n* = 134), 4% never used CBDS (5/134). From the ones using CBDS (*n* = 129), 24% have been in practice for less than 5 years (31/129), 22% were in practice between 5 and 10 years (28/129), 33% were in practice between 11 and 20 years (43/129), and 21% were in practice for more than 20 years (27/129). From these, 73% (94/129) considered they used it frequently or with high frequency in dogs and 61% in cats (79/129), maybe owing to the prevalence of literature regarding the use of these products in dogs compared to cats. A chi‐square test showed that years of experience tend (*P*<0.105) to affect the frequency of CBDS use. From respondents that never used CBDS, 80% cite lack of knowledge about their properties and applications (4/5), as the main reason for not using them, suggesting a lack of knowledge is preventing 3% (4/134) of the veterinary community from considering the CBDS as a therapeutic tool. Some respondents reported that they never used CBDS for: management of acute pain (54/129; 42%), seizures control (79/129; 61%), or pruritus relief (95/129; 74%). In contrast, many reported to be satisfied or very satisfied using CBDS for: management of chronic pain (118/129; 92%), oncological diseases (89/129; 67%), behaviour changes/stress (74/129;57%), or as an anti‐inflammatory drug (69/129; 53%). Additionally, 22 respondents referred the use of CBDS for other purposes, namely: cognitive dysfunction (5/129; 4%), appetite stimulant (4/129; 3%), feline gingivostomatitis (11/129; 8.5%), geriatric animals (1/129; 1%) and wound healing (1/129; 1%). Regarding side effects, the most emphasised were: sialorrhea (39/173, 23%) and apathy (28/173, 16%). About 26% (45/173) did not identify any side effects.

In conclusion, according to this survey, CBDS are already being used daily by Portuguese veterinarians, despite that most respondents highlighted the lack of scientific evidence regarding these products. These products are more frequently used in dogs than in cats. There is a perceived benefit of CBDS in chronic pain, oncological diseases, behavioural changes/stress, and anti‐inflammatory effects.

**Disclosures**

**Source of Funding**

**ECVIM CSF Funding**

No.

## ECVIM_Research Reports

## New insights into sleep‐disordered breathing and inflammatory response in dogs

### I. Niinikoski^1^, S.‐L. Himanen^2,3^, M. Tenhunen^3^, N. Koho^1^, L. Lilja‐Maula ^1^, M. M. Rajamäki^1^

#### ^1^University of Helsinki, Helsinki Finland; ^2^University of Tampere, Tampere, Finland; ^3^Tampere University Hospital, Tampere Finland

The aims of this PhD thesis were to evaluate the usability of an at‐home sleep apnea neckband for detection of sleep‐disordered breathing (SDB) and to assess risk factors for and signs of SDB in dogs. The low‐grade inflammatory status of brachycephalic dogs (BDs) was evaluated to acquire insight into the consequences of brachycephalic obstructive airway syndrome (BOAS).

63 privately owned pet dogs (28 BDs and 35 non‐brachycephalic dogs (non‐BDs)) were prospectively recruited. Recording with the neckband was done over one night at each dog's home. Obstructive respiratory event index (OREI) was used for evaluation of SDB. The percentage of time spent snoring was assessed. Body condition and BOAS severity were graded.

Sleep recordings from 12 BDs and 12 non‐BDs were included in the first study. BDs had a significantly higher OREI value (*p* < .001) and snore percentage (*p* < .001) than non‐BDs. The proportion of snoring did not correlate with SDB severity in BDs. In the second study comprising 63 dogs, risk factors for high OREI included brachycephaly (*p* < .001) and excess weight (*p* < .001), but not ageing, gender, or neuter status (*P* = .2; *P* = 1.0; *P* = .9). For BDs, BOAS+ class (moderate or severe BOAS signs) was a significant risk factor (*P* = .03). SDB occurred also in non‐BDs. Sleeping position, apneic episodes during sleep, and nighttime restlessness were identified as potential signs of SDB.

In the third study, proinflammatory mediators VEGF‐A and CCL2 were evaluated with a bead‐based immunoassay from serum samples of 114 BDs and 25 non‐BDs, and from bronchoalveolar lavage fluid (BALF) of 23 English Bulldogs and 14 non‐BDs. A proinflammatory condition, seen as an increase in serum VEGF‐A (*P* = .009) and decrease in BALF VEGF‐A (*p* < .001) was discovered in English Bulldogs. Evaluation of breathing during sleep was not performed in these dogs.

Brachycephaly affects welfare in many ways, including the disruption of sleep. Snoring cannot be used as the sole indicator of SDB, and other signs may include changes in sleeping position, apneic episodes during sleep, and nighttime restlessness. Brachycephaly predisposes to SDB, but SDB occurs also in non‐BDs. Obesity should be avoided to improve breathing during sleep in all dogs. The proinflammatory condition in English Bulldogs supports low‐grade inflammation due to brachycephaly and possibly due to SDB.

## Feline hyperaldosteronism: Immunohistochemical, genetic and functional characterisation of the adrenal gland of hypertensive cats

### A. H. Watson^1,2^; M. J. Brown^2^, H. M. Syme^1^

#### ^1^Royal Veterinary College London United Kingdom; ^2^Queen Mary, University of London, London, United Kingdom

Cats are relatively prone to primary hyperaldosteronism (PA) and PA may cause more cases of feline hypertension than are currently recognised. Cats have a single CYP11B enzyme, thought to make aldosterone and cortisol, therefore staining for aldosterone synthase cannot be used to identify aldosterone‐producing areas of the adrenal.

This research aimed to identify immunohistochemical markers of aldosterone producing tissue in cats and indicators of malignancy. We aimed to identify somatic mutations and quantify gene expression within adrenal tumours causing PA. Steroid profiles were generated using mass spectrometry, and aldosterone quantification compared with measurement by radioimmunoassay. We aimed to investigate if the cat CYP11B enzyme can make both aldosterone and cortisol.

Adrenal tissue was obtained at adrenalectomy or post‐mortem examination. Cats with PA had hypokalaemia and unilateral adrenal masses. Control adrenals (*n* = 8) and adrenal tumours (*n* = 30) were stained using immunohistochemistry (for CYP11B, CYP17A1, KCNJ5, VSNL1, NSE, CYB5A, and Ki67), and histochemical staining for reticulin. Whole genome and targeted Sanger sequencing of paired adrenal tumour and non‐tumour tissue (*n* = 13) was used to identify somatic mutations. RNA sequencing compared gene expression in adrenal tumours (*n* = 5) with non‐tumorous adrenals (*n* = 4). Steroids within residual serum or plasma samples were quantified using mass spectrometry (*n* = 27). Epithelial, non‐steroidogenic cell lines were transfected with the cat CYP11B gene, treated with steroid precursors then aldosterone and cortisol measured.

Presence of CYP11B, KCNJ5 and VSNL1 but absence of CYP17A1 was consistent with aldosterone secreting tissue. Function of the zona reticularis is unclear as CYB5A protein was not expressed and neutered cat blood had low androgen concentrations. Neither Ki67 nor reticulin adequately differentiated carcinomas from adenomas. Somatic mutations identified in *GNAQ, CTNNB1* and *CACNA1C* are analogous to mutations identified in human aldosterone producing tumours. Aldosterone measurement by mass spectrometry and radioimmunoassay were strongly correlated. Cats with primary hyperaldosteronism did not have multiple‐steroid excesses. Both cortisol and aldosterone were synthesised by the cat CYP11B gene.

No perfect marker of aldosterone producing tissue exists in cats, however a combination of immunohistochemical stains, in conjunction with looking at tissue architecture may suggest its endocrine function. Spatial gene expression analysis may help clarify the role of the feline zona reticularis. Similar mutations were identified in feline tumours as those found in human tumours which may suggest a common pathogenesis.

